# ﻿Type specimens of non-passerines in Naturalis Biodiversity Center (Animalia, Aves)

**DOI:** 10.3897/zookeys.1155.98097

**Published:** 2023-03-24

**Authors:** Steven D. van der Mije, Pepijn Kamminga, René W. R. J. Dekker

**Affiliations:** 1 Naturalis Biodiversity Center, P.O. Box 9517, 2300 RA, Leiden, Netherlands Naturalis Biodiversity Center Leiden Netherlands

**Keywords:** Aves, birds, Leiden, non-passerines, RMNH, Schlegel, Temminck, types, ZMA

## Abstract

The non-passerine type specimens in Naturalis Biodiversity Center, Leiden are listed as an update to Van den Hoek Ostende et al. (1997) ‘Type-specimens of birds in the National Museum of Natural History, Leiden, Part 1. Non-Passerines’ and Roselaar and Prins (2000) ‘List of type specimens of birds in the Zoological Museum of the University of Amsterdam (ZMA), including taxa described by ZMA staff but without types in the ZMA’. All new names published by Temminck and Schlegel are listed, even when types are not in Naturalis but in other collections. We have added 380 new names and deleted 13 names originally listed in Van den Hoek Ostende et al. (1997).

## ﻿Introduction

In 1997, Van den Hoek Ostende et al. published ‘Type-specimens of birds in the National Museum of Natural History, Leiden, Part 1. Non-Passerines’. Since its publication 26 years ago much has changed, both at an institutional level as well as regarding the collections. The National Museum of Natural History (NNM since 1988), the new name for the Rijksmuseum van Natuurlijke Historie as it was called since 1820, was renamed Naturalis Biodiversity Center (collection acronym: RMNH) in 2011 after the merger with staff and collections of the Zoological Museum of the University of Amsterdam (ZMA) and the Herbaria and staff from the Leiden, Utrecht, and Wageningen Universities.

The merger of the bird collection of Naturalis with that of the ZMA, discussions with colleagues, publications of type catalogues in other museums such as Paris and Vienna, corrections of publication dates of historic literature and hence original descriptions and corrections, and additions found by Naturalis staff and visitors to the collection all made an update of the RMNH catalogue necessary. This work was facilitated by the rapid digitisation of (historical) books and journals (in particular BHL) which enabled quick access and easy checks of the literature. Furthermore, the merged bird collections of the RMNH and ZMA have been fully digitised, making thorough searching and comparison of specimens and their data possible.

### ﻿What is new in this catalogue compared with the 1997 version

Much has changed in this update of Van den Hoek Ostende et al. (1997), who merely compiled the many notes by J. van der Land (curator of invertebrates) and G.F. Mees (former curator of birds) as well as information from specimen labels. Literature research for type specimens not yet listed or labelled as such, which prior to 1997 was time consuming by searching for and flipping through books and journals in the library, was not part of the scope in 1997.

What is new in this 2023 version:

1. We have re-checked all the original descriptions and related literature in this catalogue. We concentrated our efforts on historic works by former RMNH ornithologists such as Temminck’s ‘Planches Coloriées’ (1820–1839) and Schlegel’s monographs (1862–1874) as well as travellers such as Müller, Von Siebold, and others such as Sharpe who were in close contact with them.
2. We added 380 names of taxa described by Temminck, other Leiden curators, and other ornithologists. We also added names with their types in collections other than Leiden or which could not be found despite reference to the type specimens in the RMNH. This is a service to the users of this catalogue to show that we are aware of these names and performed extensive searches in the physical as well as in the digital collection. It will help taxonomists to locate these types in other collections. We have, however, limited this search to published data and have not consulted these collections and/or their curators. We refer to these specimens with their registration numbers as they are listed in published works or (online) databases, we did not check for correctness.
3. We removed the type specimens of 13 names which were listed in Van den Hoek Ostende et al. (1997) as they turned out to be listed erroneously. The 13 names, however, remain in the list and our correction is explained.
4. We listed specimens of which the type status could not be ascertained with certainty as possible types.
5. We included the type specimens formerly in the Zoological Museum of the University of Amsterdam (Roselaar and Prins 2000) which are now part of the collection of Naturalis Biodiversity Center. We also reviewed this catalogue and made corrections and additions where necessary in this update.
6. We added specimen information between square brackets [...] when this information was neither on the label nor written under the stand but found in the original description or secondary sources. An important source of information on collectors and their travels in South-East Asia was the ‘Cyclopaedia of Malesian Collectors’ compiled by M.J. van Steenis-Kruseman (1950), originally published in the Flora Malesiana series, available online ^1^. For additional information on the history of the RMNH (ornithological) collection and its curators, see Gijzen (1938), Holthuis (1995), and the introduction in Van den Hoek Ostende et al. (1997).
7. We corrected, where necessary, years of publication and hence original descriptions of names. This was based, among others, on various works of Edward Dickinson and his co-authors, as e.g., for the ‘Nouveau recueil de planches coloriées’ of Temminck and Laugier (1820–1839) (Dickinson 2001; Dickinson et al. 2022).
8. Van den Hoek Ostende et al. (1997) gave RMNH ##### as format for the registration number. This has been adapted to RMNH.AVES.##### complying with the international standard now in use for the RMNH collections. The numeric part of the unique identifier has remained unchanged.
9. Van den Hoek Ostende et al. (1997) followed the nomenclature in use in the 16 volumes of the ‘Check-List of the Birds of the World’ (Peters 1934–1987). We have updated this to current names as published by the International Ornithological Congress (IOC) starting from v. 10.1. and during the work on this catalogue shifting to the newer v. 11.1. (Gill et al. 2021) (www.worldbirdnames.org).
10. We adjusted the sequence of orders and families as in use by the IOC but listed the types alphabetically under the current IOC names for the genera and within the genera for species.
11. During the process we were confronted with information written on the labels that did not always match information under the stand or the identity of the specimen. Labels are in most cases later additions to the specimen, as almost all original labels have been discarded. Information was also regularly added as handwritings from different persons under the stands show. We noticed discrepancies in collecting date, collectors’ name, location, and gender and age of the specimen. See e.g., *Alcedolugubris* Temminck. An extreme example we encountered was a woodpecker (RMNH.AVES.203484, *Picusleucogaster* Temminck) which was mounted on a stand which originally held a cuckoo from South Africa (*Centropussenegalensis*). In several cases these discrepancies led to doubts about the true information and hence the status of the specimen. Such cases are explained under the taxon involved in which case we added “possible” to the type status of that particular specimen.
12. In some cases we encountered taxonomic issues which need to be solved, see e.g., *Cuculusfasciolatus* Müller, *Centropusmedius* Bonaparte, and *StrixRosenbergii* Schlegel. However, this is beyond the scope of this catalogue and requires a taxonomic review. We merely note the issue.
13. Van den Hoek Ostende et al. (1997) gave specimens originating from Cabinet Temminck and listed in Temminck (1807) the acquisition date of 1807. This is not correct as 1807 is the publication date of the Catalogue and Temminck’s Cabinet goes back to much earlier. Temminck’s collection was one of three founding collections of the RMNH in 1820. An important part was collected by Levaillant in South Africa between 1781 and 1784, an expedition financed by Temminck’s father.
14. Dickinson et al. (2022) discussed the publication of livraisons 1–20 in the ‘Nouveau recueil de Planches Coloriées d’oiseaux’ by Temminck (1820–1821). The first 120 plates and wrappers with scientific names were published often several years before the text appeared. Hence, the specimen on the plate is the holotype and the text becomes irrelevant regarding type status. All specimens referred to in the text should therefore not be regarded as syntypes. However, the problem we were confronted with when selecting the illustrated specimen is twofold. First, the illustrated specimen is without further information, hence it could be any one of the specimens available to Temminck and second, the illustrated specimen could be a composite of more than one specimen, e.g., when not all the characters due to sex, age, or moult were present in a single specimen or could illustrate some artistic licence. Temminck (1820: footnote, text to pl. 3) stated that, to avoid useless repetition, specimens used for the plates are always housed in the first collection mentioned. We have compared specimens in the RMNH with the plates, but were unable to do so for specimens from other collections, in particular the MNHN, in cases where Temminck might have used specimens from there. The MNHN, NMW, and other collections, like the RMNH, have listed syntypes for multiple names published in livraisons 1–20, but this should now be considered an error and we encourage curators to check these specimens against the plates if the holotype is not listed for the RMNH or when Temminck referred to these collections in his text, published later.
15. We have included types for names introduced by B.H. Hodgson. The information linked to the specimens he collected is poor, especially lacking day, month, and year of collecting. As Hodgson stayed in Nepal for a long period (1819–1844), it cannot be ruled out that some of his specimens were collected after the description of the new names.
16. We have listed all primary types with full data, secondary types by registration number only.
17. In the 1830s, Johann Heinrich Frank was a dealer in natural history specimens and antiquities in Leipzig, Germany. His son, Gustav Adolph, educated as a pharmacist, moved to Amsterdam where he started a career as a natural history dealer as well. In 1833, he contacted Temminck for the first time after which many transactions would follow. Gustav Adolph’s son, Gustaaf Adolf junior, followed the career of his father and grandfather and worked as a natural history dealer in London in 1871. Together G.A. Frank senior and junior became important international suppliers for European natural history museums.

Between 1833 and 1883, the Rijksmuseum van Natuurlijke Historie (RMNH) bought and sold to third parties a considerable number of specimens through both Frank senior and Frank junior. Additionally, the Zoological Museum Amsterdam, whose collection is part of Naturalis since 2010, dealt with Frank sr and Frank jr.

From the labels on the specimens in the Leiden collection it is often not clear whether the object was obtained through G.A. Frank sr. or G.A. Frank jr. as only G.A. Frank is mentioned. We have not tried to sort this out any further as it would be largely impossible and beyond the scope of this catalogue.

1. According to Stresemann (1953a: 319), Vieillot (1816–1819) used the ‘Catalogue systematique’ (Temminck 1807) as a basis for many of the new names in his ‘Nouveau dictionnaire d’histoire naturelle’ by slightly modifying the descriptions given by Temminck and ‘translating’ the French name into a scientific binary name.

### ﻿Abbreviations

In addition to the above, the following abbreviations have been used in the specimen information:

“**Loc:**” refers to place, country and, if given on the label, the date the specimen was collected;

“**Leg:**” refers to the field collector and, if given on the label, the date the specimen was received by the museum.

“**Ex**:” refers to the donator, which is not the field collector but a museum, owner of a private collection, or animal dealer and, if given on the label, the date the specimen was received by the museum.

Please note that in the past the date of collection and the date of accession on the labels and in the old catalogues have sometimes been mixed up.

**Acronyms** (in alphabetical order)

**AMNH**American Museum of Natural History, New York, USA.

**ANSP**Academy of Natural Sciences, Philadelphia, USA.

**MCZ**Museum of Comparative Zoology, Cambridge, Massachusetts, USA.

**MfN**Museum fur Naturkunde, Berlin, Germany (with ZMB as registration number acronym).

**MLC** Musée George Sand et de la Vallée Noire (Collection Baillon), La Châtre, France.

**MNHN**Muséum national d’Histoire naturelle, Paris, France.

**MNSL** Naturkundemuseum Leipzig, Leipzig, Germany.

**MRSN**Regional Museum of Natural Sciences of Turin, Turin, Italy.

**MSNG**Museo Civico di Storia Naturale Giacomo Doria, Genova, Italy.

**MTD** Museum für Tierkunde, Dresden, Germany.

**MWNH**Museum Wiesbaden, Hessisches Landesmuseum für Kunst und Natur, Naturhistorische Sammlungen, Wiesbaden, Germany.

**MZB**Museum Zoologicum Bogoriense, Bogor, Indonesia.

**MZUT**Zoological Museum of the University of Turin, now Museo Regionale di Scienze Naturali di Torino, Torino, Italy.

**NAM** Amsterdam Zoo ‘Natura Artis Magistra’, Amsterdam, The Netherlands.

**NHM**Natural History Museum, London, UK.

**NMNH**Smithsonian National Museum of Natural History, Washington, USA. (with USNM as registration number acronym).

**NMP**Národní Museum Prague, Prague, Czech Republic.

**NMS**National Museums Scotland, Edinburgh, Scotland.

**NMW**Naturhistorisches Museum Wien, Vienna, Austria.

**NRM**Naturhistoriska Riksmuseet, Stockholm, Sweden.

**RBINS**Royal Belgian Institute of Natural Sciences, Brussels, Belgium.

**RMNH**Rijksmuseum van Natuurlijke Historie, now Naturalis Biodiversity Center, Leiden, The Netherlands (with RMNH as registration number acronym).

**SMF**Senckenberg Museum Frankfurt, Frankfurt, Germany.

**SMNS**Naturkundemuseum Stuttgart, Stuttgart, Germany.

**UZMC** Universitets Zoologiske Museum, Copenhagen, Denmark.

**ZFMK**Zoologisches Forschungsinstitut und Museum Alexander Koenig, Bonn, Germany.

**ZMA**Zoölogisch Museum Amsterdam, Amsterdam, The Netherlands.

**ZMM**Zoological Museum, Moscow State University, Moscow, Russia.

**ZSM**Zoologische Staatssammlung München, Munich, Germany.

## ﻿Acknowledgements

We wish to thank the following for discussions on a variety of taxa and topics, including literature and archives, listed in alphabetical order: Norbert Bahr, Arnoud van den Berg, Patrick Boussès, Alice Cibois, Wim Dekkers, Edward Dickinson, Robert Dowsett, Angelos Evangelidis, Clem Fisher, Christophe Gouraud, Gradimir Gradev, Hein van Grouw, Justin Jansen, Karien Lahaise, Robert Prÿs-Jones, Christiane Quaisser, Frank Rheindt, Frank Steinheimer, Jan van Tol, Pieter van Wingerden, Konstantinos Vlachopoulos, Ruud Vlek and Claire, and François Voisin.

## ﻿List of type specimens

### ﻿CASUARIIFORMES

**Casuariidae**

***Casuariuspapuanus*** Schlegel, 1871b: 54.

= *Casuariusbennettiwestermanni* Sclater, 1874.

Syntype, RMNH.AVES.87001, adult female, mounted skin. Loc.: Andai, (Berau Peninsula), [Indonesia], 20.iii.1870. Leg.: C.B.H. von Rosenberg.

Syntype, RMNH.AVES.87002, immature male, mounted skin. Loc.: Andai, (Berau Peninsula), [Indonesia], 09.v.1870. Leg.: C.B.H. von Rosenberg.

Schlegel (1871b) published his *C.papuanus* three years prior to Sclater’s *C.westermanni* Sclater, 1874. Hence *C.papuanus* has priority over *C.westermanni*. The IOC listing refers to Perron (2011) and Beehler and Pratt (2016) for usage of *westermanni* for the form from the Vogelkop Peninsula because, as stated, “the provenance of the specimen associated with *C.papuanus* remains unsettled”. Both syntypes of *C.papuanus*, however, have details attached to them about provenance, collector, and date. Dickinson and Remsen (2013) did accept *C.papuanus* in contrast to IOC.

***Casuariusaruensis*** Schlegel, 1866f: 347.

= *Casuariuscasuarius* (Linnaeus, 1758).

Syntype, RMNH.AVES.87003, adult female, mounted skin. Loc.: Wammer [= Warmar], Aru Isles, [Indonesia], 15.iv.1865. Leg.: C.B.H. von Rosenberg.

Syntype, RMNH.AVES.87004, immature female, mounted skin. Loc.: Kobroör, Aru Isles, [Indonesia]. Leg.: C.B.H. von Rosenberg.

***Casuariuscasuariusbistriatus*** van Oort, 1908c: 205.

= *Casuariuscasuarius* (Linnaeus, 1758).

Holotype, RMNH.AVES.850, adult male, mounted skin. Loc.: North coast of New Guinea, [Indonesia], 1904. Ex: Rotterdam Zoo, 03.v.1907.

Holotype by monotypy. Van Oort described a single adult male captured on the “north coast of New Guinea, west of Humboldt Bay, very probably from Tarfia near Matterer Bay”, and sent alive to the zoological garden of Rotterdam, where it died in May 1907. According to the accession books, the skeleton of the holotype is also in the RMNH but was not found.

***CasuariusKaupi*** Von Rosenberg, 1861: 44.

= *Casuariusunappendiculatus* (Blyth, 1860).

Rosenberg referred to his hunters who collected a single male on Salawati in August 1860. No specimen in the RMNH agrees with this. Furthermore, Schlegel (1873c: 12) did not mention this specimen in his letter to Sclater (1866: 168) or in his catalogue of the Struthiones where he discussed the validity of *C.kaupi* Rosenberg.

### ﻿TINAMIFORMES

#### ﻿Tinamidae

***Tinamuserythropus*** Von Pelzeln, 1863: 1127.

= *Crypturelluserythropuserythropus* (Pelzeln, 1863).

Syntype, RMNH.AVES.87005, adult male, mounted skin. Loc.: Barra do Rio Negro [= Manaus], Brazil, 06.xi.1832. Leg.: J. Natterer. Ex: NMW, 1864.

Syntype, RMNH.AVES.87006, adult female, mounted skin. Loc.: Barra do Rio Negro [= Manaus], Brazil, 29.vii.1833. Leg.: J. Natterer. Ex: NMW, 1862.

Other syntypes collected by Natterer at Barra do Rio Negro are in the NMW (NMW 9987 and NMW 20514–20520; Schifter et al. 2007: 34–35) and in the NMNH (USNM 35005; Deignan 1961: 4).

***Tinamusobsoletus*** Temminck, 1815a: 588.

= *Crypturellusobsoletusobsoletus* (Temminck, 1815).

Temminck based this name on 16 specimens described and collected by d’Azara in Paraguay (1781–1801). Temminck specifically referred to two specimens in the MNHN and one specimen in his own collection which could, however, not be found in the RMNH.

***Tinamusstrigulosus*** Temminck, 1815a: 594.

= *Crypturellusstrigulosus* (Temminck, 1815).

Temminck based this name on a single specimen in his own cabinet donated by Hoffmannsegg which is no longer present in the RMNH.

***Tinamustataupatataupa*** Temminck, 1815a: 590.

= *Crypturellustataupatataupa* (Temminck, 1815).

Temminck based this name on the description by De Azara (1802–1805, no. 329) and two specimens in the MNHN.

***CrypturusKerberti*** Büttikofer, 1896a: 1.

= *Crypturellustataupatataupa* (Temminck, 1815).

Holotype, RMNH.AVES.87007, adult female, skin. Loc.: Argentina. Ex: NAM, 02.iv.1896.

Holotype by monotypy. Büttikofer (1896a) described a single adult female sent alive from Argentina and died in the zoological garden of NAM.

In the list of types from the ZMA, Roselaar and Prins (2000: 96) “selected” a paralectotype (ZMA.AVES.4961) because the holotype is in an imperfect state and the taxonomic status of *kerberti* is questionable. However, since the ZMA specimen is not mentioned by Büttikofer and is not part of the type series, it cannot be given paralectotype status.

***Tinamusadspersus*** Temminck, 1815a: 585.

= *Crypturellusundulatusadspersus* (Temminck, 1815).

Temminck based his description on a single specimen in the MfN, collected in Para, Brazil.

***Tinamusundulatus*** Temminck, 1815a: 582.

= *Crypturellusundulatusundulatus* (Temminck, 1815).

Temminck based this name on a description by De Azara (1802–1805) but had not seen any specimens.

***Tinamusvermiculatus*** Temminck, 1825: livr. 62, pl. 369.

= *Crypturellusundulatusvermiculatus* (Temminck, 1825).

Two syntypes from Brazil are presumed to be in the MNHN.

***Tinamusmaculosus*** Temminck, 1815a: 557.

= *Nothuramaculosamaculosa* (Temminck, 1815).

Temminck had seen two specimens in the MNHN and NHM.

***Tinamusrufescens*** Temminck, 1815a: 552.

= *Rhynchotusrufescensrufescens* (Temminck, 1815).

Temminck based his description on a single specimen in the MNHN.

***Tinamusnanus*** Temminck, 1815a: 600.

= *Taoniscusnanus* (Temminck, 1815).

Temminck based this name on a description by De Azara (1802–1805) and indicated that he had not seen any specimens.

***Tinamusguttatus*** Von Pelzeln, 1863: 1126.

= *Tinamusguttatus* Pelzeln, 1863.

Syntype, RMNH.AVES.87008, adult male, mounted skin. Loc.: near Belém do Pará, Brazil, 15.xi.1834. Leg.: J. Natterer. Ex: NMW, 1864.

This is one of eight syntypes. Syntypes collected by Natterer are in the NHM (NHMUK 1892.6.9.131; not listed in Warren 1966), the NMNH (USNM 35004; Deignan 1961: 3) and the NMW (NMW 9988 and 9989, 20524–20526; Schifter et al. 2007: 33). The type locality was restricted to Borba by Hellmayr (1907: 409).

***Tinamustao*** Temminck, 1815a: 569.

= *Tinamustaotao* Temminck, 1815.

Temminck based this name on two specimens, one in a collection in Lisbon, the other in the MfN.

### ﻿GALLIFORMES

#### ﻿Megapodiidae

***Talegalluspyrrhopigius*** Schlegel, 1879f: 159.

= *Aepypodiusarfakianusarfakianus* (Salvadori, 1877).

Holotype, RMNH.AVES.87335, adult, sex unknown, mounted skin. Loc.: [Andai], New Guinea [Indonesia], iv.1878. [Leg.: W.H. Woelders] Ex: G.A. Frank, 1879.

Holotype by monotypy. Schlegel (1879f) based his description on “the skin of one single specimen [...] recently brought to Europe by one of the Dutch missionaries”.

The spelling of *pyrrhopigius* in the title is clearly an error as later in the text it is correctly spelled *pyrrhopygius*.

This specimen was acquired through one of the Frank dealers. The collector must have been missionary Woelders who returned to the Netherlands in February 1879, on leave from Andai, New Guinea, bringing his collection with him (Adriani 1895).

***Macrocephalonmaleo*** Müller, S., 1846: 116.

***Megacephalonmaleo*** Gray, 1846: 849.

= *Macrocephalonmaleo* Müller, S., 1846.

Syntype, RMNH.AVES.226916, adult male, mounted skin. Loc.: Boujat, [Sulawesi, Indonesia], 1840. Leg.: E.A. Forsten.

Syntype, RMNH.AVES.226917, adult female, mounted skin. Loc.: Boujat, [Sulawesi, Indonesia], 1840. Leg.: E.A. Forsten.

Syntype RMNH.AVES.226918, immature, mounted skin. Loc.: Manado, [Sulawesi, Indonesia], 1840. Leg.: E.A. Forsten.

Sal. Müller based his name on *Megachephalonmaleo* Temm. ms. These are also the syntypes of *Megacephalonmaleo* Gray (1846: 849).

Forsten stayed in Celebes twice between March 1840 and April 1842.

***MegapodiusBernsteinii*** Schlegel, 1866d: 261.

= *Megapodiusbernsteinii* Schlegel, 1866.

Syntype, RMNH.AVES.87340, immature female, mounted skin. Loc.: Sula Mangola, [Indonesia]. Leg.: H.A. Bernstein, 1863.

Syntype, RMNH.AVES.227463, adult male, mounted skin. Loc.: Sula Bessie, [Indonesia], 16.xi.1864. Leg.: H.A. Bernstein.

Syntype, RMNH.AVES.227464, adult male, mounted skin. Loc.: Sula Mangola, [Indonesia], 5.xii.1864. Leg.: D.S. Hoedt.

Syntype, ZMA.AVES.25836, adult female, skin. Loc.: Sula isl., 30.xi.1864. Leg.: D.S. Hoedt.

One syntype was sent to the MNHN in July 1875.

***Megapodiussanghirensis*** Schlegel, 1880: 91.

= *Megapodiuscumingiisanghirensis* Schlegel, 1880.

Syntype, RMNH.AVES.87341, adult female, mounted skin. Loc.: Sanghir, [Indonesia], 29.xi.1865. Leg.: D.S. Hoedt.

Syntype, RMNH.AVES.87342, adult male, mounted skin. Loc.: Sanghir, [Indonesia], 23.i.1866. Leg.: D.S. Hoedt.

Syntype, RMNH.AVES.87343, adult, sex unknown, mounted skin. Loc.: Siao-outong, [Indonesia]. Ex: L.D.H.A. Renesse van Duyvenbode, 1866.

Syntype, RMNH.AVES.87344, adult female, mounted skin. Loc.: Siao-outong, [Indonesia]. Ex: L.D.H.A. Renesse van Duyvenbode, 1866.

Syntype, RMNH.AVES.87345, adult male, mounted skin. Loc.: Siao, [Indonesia]. Ex: L.D.H.A. Renesse van Duyvenbode, 1866.

Syntype, RMNH.AVES.87346, adult, sex unknown, mounted skin. Loc.: Siao, [Indonesia]. Ex: L.D.H.A. Renesse van Duyvenbode, 1866.

***Megapodius Forstenii*** Gray, 1847: pl. CXXIV.

***Megapodius Forsteni*** Gray, 1847: 491 (nomen nudum).

= *Megapodiusfreycinetforsteni* Gray, 1847.

Lectotype, RMNH.AVES.87348, adult female, mounted skin. Loc.: Ceram, [Indonesia], 1840. Leg.: E.A. Forsten.

Gray gave no indication of the number of specimens he used for his description: he merely published a manuscript name by Temminck. Therefore, this specimen should be a syntype, but by listing it as holotype Van den Hoek Ostende et al. (1997: 52) designated it the lectotype.

Forsten visited Ceram [= Seram] late 1842, so either he received this specimen from another collector, or the date is an error.

The IOC spelling of *forsteni* (single “i”) is incorrect, based on a nomen nudum, and should follow the spelling (double “ii”) as on the plate.

***Megapodiusfreycinetoustaleti*** Roselaar, 1994: 27.

= *Megapodiusfreycinetoustaleti* Roselaar, 1994.

Holotype, RMNH.AVES.87336, adult male, mounted skin. Loc.: Sorong, [Indonesia], 28.xi.1864. Leg.: H.A. Bernstein.

Paratypes, RMNH.AVES.87337–87339.

There is also a paratype in the NHM (NHMUK 1881.5.1.5666).

***Megapodius Reinwardt*** Dumont, 1823: 416.

***Megapodiusrubripes*** Temminck, 1826: livr. 69, pl. 411.

= *Megapodiusreinwardtreinwardt* Dumont, 1823.

Holotype for *reinwardt*, syntype for *rubripes*, RMNH.AVES.87347, adult, sex unknown, mounted skin. Loc.: Lombok, [Indonesia], 1818 [error?]. Leg.: C.G.C. Reinwardt.

Holotype by monotypy for *M.reinwardt* Dumont, 1823 as he referred to a single individual. He erroneously indicated Amboine [= Ambon] as the type locality. The locality Lombok also seems to be erroneous, since Reinwardt never visited the island; the correct locality is most likely Buma, Sumbawa, where he stayed from 20–23 March 1821 (G.F. Mees, pers. comm.).

In his description of *M.rubripes*, Temminck (1826) indicated that the material was collected by Reinwardt, who collected four specimens. Schifter et al. (2007: 84–85) listed two syntypes received from the RMNH in the NMW (NMW 48139 and 88296).

***Megapodius Tumulus*** Gould, 1842b: 20.

= *Megapodiusreinwardttumulus* Gould, 1842.

Paralectotype, RMNH.AVES.90977, adult male, mounted skin. Loc.: Port essington, Australia. Leg.: -. Ex.: J. Gould.

This specimen was acquired through Gould and possibly collected by his collector Gilbert.

The lectotype was selected by Stone (1913: 132) which is in the ANSP (ANSP 12770, male. Loc.: Port Essington). See also Fisher and Calaby (2009: 77–79).

***Talegallafuscirostrisaruensis*** Roselaar, 1994: 15.

= *Talegallafuscirostris* Salvadori, 1877.

Holotype, RMNH.AVES.87349, adult female, mounted skin. Loc.: Wonumbai [Kobroor], Aru Isl., [Indonesia], 16.v.1865. Leg.: C.B.H. von Rosenberg.

Paratype, RMNH.AVES.87350.

Two paratypes are in the MTD (MTD C7298; Eck and Quaisser 2004: 235) and the MfN (ZMB 51178).

***Talegallusjobiensis*** Meyer, 1874: 74.

= *Talegallajobiensisjobiensis* A.B. Meyer, 1874.

Syntype, RMNH.AVES.87351, adult female, mounted skin. Loc.: Ansous, Jobie [= Japen], [Papua, Indonesia], iv.1873. Leg.: A.B. Meyer. Received 1879.

The species was described from a series of five specimens. Only two seem to have survived: one in the MTD (MTD C3135; Eck and Quaisser 2004: 235) and RMNH.AVES.87351 (Roselaar 1994).

#### ﻿Cracidae

***Craxcarunculata*** Temminck, 1815a: 44.

= *Craxglobulosa* Spix, 1825.

Temminck’s description is based on a single skin in a collection in Lisbon.

***Penelopeparrakoua*** Temminck, 1815a: 85 (nomen novum).

= *Ortalismotmot* (Linnaeus, 1766).

Temminck introduced this name to bring order to the names for this taxon (among others *Phasianusmotmot* Linnaeus, *Phasianusparraqua* Latham, *Phasianusgarrulus* Humboldt and *Phasianusguianensis* Brisson). Although Temminck referred to specimens in his cabinet, there are no types involved here.

***Penelopeobscura*** “Illiger” Temminck, 1815a: 68.

= *Penelopeobscuraobscura* Temminck, 1815.

Temminck based this new species on the description by De Azara (1802–1805). He had not seen any specimens.

***Penelopesuperciliaris*** “Illiger” Temminck, 1815a: 72.

= *Penelopesuperciliarissuperciliaris* Temminck, 1815.

Temminck based this new species on one specimen in his own cabinet and two specimens in the MfN, all collected by Hoffmannsegg in Para, Brazil. The specimen from Cabinet Temminck is no longer in the RMNH.

#### ﻿Numididae

***Agelastesmeleagrides*** Bonaparte, 1850d: 145.

= *Agelastesmeleagrides* Bonaparte, 1850.

Syntype, RMNH.AVES.87414, adult male, mounted skin. Loc.: Dabocrom, Ashantee, Côte d’Or [= Ghana]. Leg.: H.S. Pel.

Syntype, RMNH.AVES.87415, adult male, mounted skin. Loc.: Dabocrom, Ashantee, Côte d’Or [= Ghana]. Leg.: H.S. Pel.

#### ﻿Odontophoridae

***Perdix Sonnini*** Temminck, 1815a: 451.

= *Colinuscristatussonnini* (Temminck, 1815).

Syntype, RMNH.AVES.87357, adult male, mounted skin. Loc.: South America. Leg.: -.

Temminck based his description on specimens in his own cabinet and in the MNHN. Voisin et al. (2015: 8) did not consider their only specimen (MNHN C.G.2013-34) a syntype of *P.sonnini*.

***Odontophoruscubanensis*** Gray, 1846: [no pagination].

= *Colinusvirginianuscubanensis* (G.R. Gray, 1846).

Syntype, RMNH.AVES.224580, adult, sex unknown, mounted skin. Loc.: Cuba. Leg.: -.

This name is listed by G.R. Gray (1846) as a manuscript name by Gould and referring to a description of *Colinusvirginianus* from Cuba by D’Orbigny (1839–1843; no specimens mentioned). Later, Gould (1850) gave a formal description of *O.cubanensis*, mentioning, among others, a specimen in the RMNH. There are no types for *O.cubanensis* listed in Voisin et al. (2015).

***Perdixborealis*** Temminck, 1815a: 436 (nomen novum).

= *Colinusvirginianus* subsp.

Temminck introduced this name as a nomen novum for multiple names being in use for this taxon.

***OdontophorusColumbianus*** Gould, 1850: [30].

= *Odontophoruscolumbianus* Gould, 1850.

Syntype, RMNH.AVES.87399, adult male, mounted skin. Loc.: ‘Colombie’. Leg.: R.F. van Lansberge.

Syntype, RMNH.AVES.87400, adult female, mounted skin. Loc.: ‘Colombie’. Leg.: R.F. van Lansberge.

According to the label and information written under the stand these specimens originate from Colombia, as indeed the name suggests. However, the species is restricted to Venezuela. In his description Gould (1850) mentioned “Caracas” as the type locality and uses the vernacular name “Caraccas Partridge” [sic]. R.F. van Lansberge worked both in Caracas and Santa Fé de Colombia as consul for the Dutch government between 1826 and 1855 and probably these localities got mixed up with the labelling.

***Perdixdentata*** Temminck, 1815a: 419 (nomen novum).

= *Odontophorusgujanensisgujanensis* (Gmelin, 1789).

Temminck introduced this name as a nomen novum for *Perdixguianensis* [sic] Latham.

#### ﻿Phasianidae

***Perdixheyi*** Temminck, 1825: livr. 55, pl. 328 (male) and 329 (female).

= *Ammoperdixheyiheyi* (Temminck, 1825).

Syntype, RMNH.AVES.87352, adult male (error for female), mounted skin. Loc.: [Akaba], Arabia, [1822]. Leg.: [W.P.E.S. Rüppell. Ex: SMF].

Syntype, RMNH.AVES.87353, adult male, mounted skin. Loc.: [Akaba], Arabia, [1822]. Leg.: [W.P.E.S. Rüppell. Ex: SMF].

According to the original description the two syntypes, collected by Hey in the deserts of Aqaba in Jordan, were received from the SMF.

***Anurophasismonorthonyx*** van Oort, 1910e: 212.

= *Anurophasismonorthonyx* van Oort, 1910.

Holotype, RMNH.AVES.87354, adult male, skin. Loc.: Oranje mountains, [Jayawijaya, Papua, Indonesia], 05.xi.1909. Leg.: H.A. Lorentz, Dutch New Guinea Expedition, 1909.

Holotype by monotypy. Van Oort based his description on a single male, field number 254.

***Arborophilajavanicabartelsi*** Siebers, 1929: 149.

= *Arborophilajavanicajavanica* (Gmelin, 1789).

Holotype, RMNH.AVES.14027, adult male, skin. Loc.: Gn. Tjerimai, Cheribon, Java, [Indonesia], 31.v.1928. Leg.: J.J. Menden. Ex: MZB, 12.i.1950 (MZB 5680).

The description was based on ten specimens. The paratypes are in the MZB (MZB 5681–5685, 6916–6919).

***Arborophilabrunneopectuslawuana*** Bartels, 1938: 321.

= *Arborophilajavanicalawuana* Bartels, 1938.

Holotype, RMNH.AVES.29802, adult female, skin. Loc.: Gunung Lawoe, Java, [Indonesia], 24.vi.1936. Leg.: M. Bartels Jr. and P.J. Bouma. Ex: Bartels, 01.vi.1954.

Paratypes, RMNH.AVES.29803–29807.

Bartels (1938) based his description on a series of eight specimens. One of the paratypes is in the MZB (MZB 6843). The eighth specimen was part of the Bouma collection; its whereabouts are now unknown. All paratype labels bear 1938 as the year of collecting which is in error for 1936.

***PerdixVethi*** Snelleman, 1887: 30, pl. III.

= *Arborophilarubrirostris* (Salvadori, 1879).

Holotype, RMNH.AVES.87355, adult female, mounted skin. Loc.: [Alahan Pandjang], W. Sumatra, [Indonesia, 9.viii.1877–29.ix.1877]. Leg.: Sumatra expedition [1877].

Holotype by monotypy: Snelleman (1887: 46) wrote that this female was the only specimen collected. This name is published as a junior synonym of *Arborophilarubrirostris*.

***Perdixmegapodia*** Temminck, 1828: livr. 78, pl. 462 (male) and 463 (female).

= *Arborophilatorqueolatorqueola* (Valenciennes, 1825).

Three syntypes are in the MNHN. One went to the SMF in March 1835.

***Argusgiganteus*** Temminck, 1813b: 410.

= *Argusianusargusargus* (Linnaeus, 1766).

Temminck wrote having seen more than 30 specimens originating from Malacca in his own cabinet and several other collections. No specimens which fit as types are currently present in the RMNH.

***Bambusicolasonorivox*** Gould, 1863: 285.

= *Bambusicolasonorivox* Gould, 1863.

Syntype, RMNH.AVES.87356, adult female, mounted skin. Loc.: N. Formosa [= Taiwan], v.1861. Leg.: R. Swinhoe, 1863.

Syntype, RMNH.AVES.88810, adult male, mounted skin. Loc.: Formosa [= Taiwan], iii.1862. Leg.: R. Swinhoe, 1863.

These are also the types for the genus name *Bambusicola* Gould, 1863. Warren (1966: 276) lists NHMUK 1863.2.16.4 as syntype in the NHM.

***Perdixthoracica*** Temminck, 1815a: 335.

= *Bambusicolathoracicus* (Temminck, 1815).

Temminck (1815a) based his description on a single skin in the collection of Raye van Breukelerwaert. Although some of this collection was bought for the RMNH by Temminck at its sale in 1827, this specimen is not in the RMNH.

***Perdixoculae*** Temminck, 1815a: 408.

= *Caloperdixoculeusoculeus* (Temminck, 1815).

Temminck (1815a) based his description on a single male specimen in the collection of Raye van Breukelerwaert. This specimen was listed in the sales catalogue (1827: 51) and according to annotations by Temminck in the RMNH archive sold for 3 guilders and 15 cents. Although some of the collection was bought for the RMNH, this specimen could not be found. One was sent to the NMW by Temminck in 1823 (NMW 1823.LXXXVIII.88).

***Coturnixtextilis*** Temminck, 1815a: 512.

= *Coturnixcoromandelica* (Gmelin, 1789).

Syntype, RMNH.AVES.87358, adult male, mounted skin. Loc.: ‘Inde Himalaya’. Leg.: -.

According to Temminck this taxon occurred on the Indian subcontinent. He referred specifically to specimens in his collection from Bengal to which no reference is made under the stand. We tentatively list RMNH.AVES.87358 as a syntype. According to Temminck other syntypes (a male and female) are in the MNHN which are not listed by Voisin et al. (2015).

***Coturnixvulgarisafricana*** Temminck & Schlegel, 1849: 103.

= *Coturnixcoturnixafricana* Temminck & Schlegel, 1849.

Syntype, RMNH.AVES.87359, adult male, mounted skin. Loc.: “Cap”, [South Africa]. Leg.: -.

Syntype, RMNH.AVES.87360, adult male, mounted skin. Loc.: “Cap”, [South Africa]. Leg.: -.

Syntype, RMNH.AVES.87361, adult female, mounted skin. Loc.: “Cap”, [South Africa]. Leg.: -.

Syntype, RMNH.AVES.87362, adult male, mounted skin. Loc.: “Cap”, [South Africa]. Leg.: H.B. van Horstok.

Syntype, RMNH.AVES.87363, adult, sex unknown, mounted skin. Loc.: “Cap”, [South Africa]. Leg.: H.B. van Horstok.

Syntype, RMNH.AVES.87364, adult, sex unknown, mounted skin. Loc.: “Cap”, [South Africa]. Leg.: H.B. van Horstok.

***Coturnixvulgarisjaponica*** Temminck & Schlegel, 1849: 103, pl. 61.

= *Coturnixjaponica* Temminck & Schlegel, 1849.

Syntype, RMNH.AVES.87365, adult male, mounted skin. Loc.: Japan. Leg.: Ph.F.B. von Siebold.

Syntype, RMNH.AVES.87366, adult, sex unknown, mounted skin. Loc.: Japan. Leg.: Ph.F.B. von Siebold.

Syntype, RMNH.AVES.87367, adult, sex unknown, mounted skin. Loc.: Japan. Leg.: Ph.F.B. von Siebold.

Syntype, RMNH.AVES.87368, adult, sex unknown, mounted skin. Loc.: Japan. Leg.: Ph.F.B. von Siebold.

Syntype, RMNH.AVES.87369, adult, sex unknown, mounted skin. Loc.: Japan. Leg.: H. Bürger.

Syntype, RMNH.AVES.87370, adult male, mounted skin. Loc.: Japan. Leg.: -.

Syntype, RMNH.AVES.87371, adult male, mounted skin. Loc.: Japan. Leg.: -.

Syntype, RMNH.AVES.87372, adult female, mounted skin. Loc.: Japan. Leg.: -.

Syntype, RMNH.AVES.87373, adult male, mounted skin. Loc.: Japan. Leg.: -, 1844.

Syntype, RMNH.AVES.87374, adult male, mounted skin. Loc.: Japan. Leg.: -, 1841.

Syntype, RMNH.AVES.87375, adult female, mounted skin. Loc.: Japan. Leg.: -, 1841.

Syntype, RMNH.AVES.87376, adult female, mounted skin. Loc.: Japan. Leg.: -, 1844.

Syntype, RMNH.AVES.87377, adult male, mounted skin. Loc.: Japan. Leg.: -, 1841.

Syntype, RMNH.AVES.87378, adult female, mounted skin. Loc.: Japan. Leg.: -, 1844.

Syntype, RMNH.AVES.87379, adult female, mounted skin. Loc.: Japan. Leg.: -.

In this catalogue we follow Holthuis and Sakai (1970) over Mlíkovský (2012b) for the year of publication. This can have consequences on the status and selection of type specimens for this name if the plate was published prior to the description in the livraison. Taxa based upon holotypes are not affected but will differ where the name is based upon a series of specimens.

***Synoicuscervinus*** Gould, 1865: 195.

= *Coturnixypsilophoraaustralis* (Latham, 1802).

Possible paralectotype, RMNH.AVES.224722.

Fisher and Calaby (2009: 80) listed this specimen as possibly from the Gould collection and therefore we consider this to be part of the type series. Stone (1913: 132) selected the lectotype in the NHM.

***PerdixRaaltenii*** Müller, 1842: 158.

= *Coturnixypsilophoraraaltenii* (Müller, 1842).

Syntype, RMNH.AVES.87403, adult male, mounted skin. Loc.: [Babau], Timor, [Indonesia]. Leg.: S. Müller [xi–xii.1828].

Syntype, RMNH.AVES.87404, adult female, mounted skin. Loc.: [Babau], Timor, [Indonesia]. Leg.: S. Müller [xi–xii.1828].

Syntype, RMNH.AVES.87405, adult male, mounted skin. Loc.: [Babau], Timor, [Indonesia]. Leg.: S. Müller [xi–xii.1828].

***Coturnixexcalfactoria*** Temminck, 1815a: 516.

= *Excalfactoriachinensis* subsp.

Syntype, RMNH.AVES.228357, adult male, mounted skin. Loc.: “Moluques” [error for Timor, viii–xi.1801]. Leg. F. Péron [R. Maugé], Baudin expedition.

Temminck (1815a) based his description on more than 20 specimens from the Moluccas and the Philippines and referred to specimens collected during the Baudin Expedition. Only one specimen in the RMNH can be linked to Temminck’s description: it was collected during the Baudin expedition on Timor, which Temminck considered to be part of the Moluccas instead of the Lesser Sundas as it is now.

Two specimens, also from Timor and collected by Baudin, are reported as syntypes in the MNHN (Jansen 2017) and represent, as does RMNH.AVES.228357, *Excalfactoriachinensislineata* (Scopoli, 1786). A specimen from Java was sent to the NMW by Temminck in April 1830. Temminck (1815a) also referred to its occurrence in China, representing the nominate form.

***Perdixgularis*** Temminck, 1815a: 401.

= *Francolinusgularis* (Temminck, 1815).

Syntype, RMNH.AVES.87388, adult male, mounted skin. Loc.: Bengal. Leg.: -.

Syntype, RMNH.AVES.87389, adult male, mounted skin. Loc.: Bengal. Leg.: -.

The description is based on two specimens, one in Temminck’s cabinet and one in the MNHN. The two syntypes listed here are indistinguishable, have the same data and look fully similar. Both specimens have ‘type’ written on their label or under the stand. Based on Temminck’s description, the specimen to which he referred as being the one in his own cabinet, cannot be identified for reasons mentioned above. Voisin et al. (2015) did not list a syntype for *Perdixgularis* but only referred to a specimen which was not considered a syntype.

***Gallusgiganteus*** Temminck, 1813b: 84.

= *Gallusgallus* (Linnaeus, 1758).

Temminck claimed to describe a wild species from Sumatra and Java. He referred to a foot of this taxon in his cabinet which is, however, no longer present in the RMNH.

***GallusMorio*** Temminck, 1813b: 253.

= *Gallusgallus* (Linnaeus, 1758).

Temminck claimed to be describing a wild bird from India.

***Gallusbankiva*** Temminck, 1813b: 87.

= *Gallusgallusbankiva* Temminck, 1813.

Temminck based his name on the description of three specimens in the MNHN by Laischenau (= Leschenault).

***Galluslanatus*** Temminck, 1813b: 256.

= *Gallusgallusdomesticus* (Linnaeus, 1758).

In the catalogue of his cabinet, Temminck (1807: 164, species no. 1047) introduced *G.lanatus* as a nomen nudum, and a formal description followed in 1813.

***Gallusecaudatus*** Temminck, 1813b: 267.

= *Galluslafayettii* Lesson, 1831.

Syntype, RMNH.AVES.224888, adult male, mounted skin. Loc.: Ceylon [= Sri Lanka]. Leg.: Governor of Ceylon. Ex: Cabinet Temminck.

Syntype, RMNH.AVES.224889, immature male, mounted skin. Loc.: Ceylon [= Sri Lanka]. Leg.: Governor of Ceylon. Ex: Cabinet Temminck.

In the catalogue of his cabinet, Temminck (1807: 145, species no. 246) introduced this name as a nomen nudum with a reference to his never published work on Galliformes. A formal description followed in 1813. For a detailed explanation of the taxonomy and history of this taxon and the types see Van Grouw et al. (2017).

Apart from two specimens in his own collection, Temminck referred to a specimen in the collection of Raye van Breukelerwaert. This specimen was sold at the sale of this collection in 1827 (Van Cleef 1827: 49, no 885) for 4 guilders and 15 cents to the RMNH where it seems to be no longer present.

***Gallussonneratii*** Temminck, 1813b: 246.

= *Gallussonneratii* Temminck, 1813.

Temminck described three males and a female without mentioning where he had seen them. The RMNH has no specimens that fit as types.

***GallusFurcatus*** Temminck, 1813b: 261.

= *Gallusvarius* (Shaw, 1798).

In the catalogue of his cabinet, Temminck (1807: 146, species no. 401) introduced this name as a nomen nudum with reference to his much later published work on Galliformes. A formal description followed in 1813 which was based on five specimens, three in the MNHN (from Leschenault) and two in the RMNH, all from Java. Later Temminck (1829: livr. 81, pl. 483) listed Java as well as Sumatra as collecting areas. Schifter et al. (2007: 98) mentioned a possible syntype in the NMW (NMW 13796), received in exchange from the RMNH. One specimen was in the MfN by March 1826. For a detailed explanation of the taxonomy and history of this taxon and the types see Van Grouw and Dekkers (2019).

***TetraoSaliceti*** Temminck, 1815a: 208.

= *Lagopuslagopus* subsp.

Possible syntype, RMNH.AVES.224446, male, adult, mounted skin. Loc.: Lapponie [= northern Scandinavia]. Leg.: -.

Two more specimens in the RMNH from Russia, but without date, might also belong to the type series.

***Lagopusbrachydactylus*** Gould, 1837: pl. 256 and text.

= *Lagopuslagopus* subsp.

Holotype, RMNH.AVES.226893, adult, sex unknown, mounted skin. Loc.: Russia. Ex: C. Fellner von Feldegg.

Holotype by monotypy. Gould wrote: “M. Temminck’s specimen, which we believe to be unique”.

***PerdixLerwa*** Hodgson, 1833: 107.

= *Lerwalerwa* (Hodgson, 1833).

Syntype, RMNH.AVES.227458, adult, sex unknown, mounted skin. Loc.: Nepal. Leg.: B.H. Hodgson.

The Hodgson collection arrived in Leiden in 1845 consisting of four crates with birds and mammals.

***Lophophorusrefulgens*** Temminck, 1813b: 355.

= *Lophophorusimpejanus* (Latham, 1790).

Syntype, RMNH.AVES.87397, adult male, mounted skin. Loc.: Himalayas. Ex: Cabinet Temminck.

Temminck referred to three specimens: one male in his own cabinet (now part of the RMNH), a specimen in the Museum Leverianum in London (a collection which was auctioned in 1806) and a specimen in the MNHN (originating from the collection of the “Stadhouder”). The specimen from the Museum Leverianum went to the NMW where it seems lost (Schifter et al. 2007: 97). Voisin et al. (2015) did not list a syntype for *refulgens* in the MNHN.

***Euplocomusdiardi*** Bonaparte, 1856b: 415.

***Diardigallusprelatus*** Bonaparte, 1856b: 415.

= *Lophuradiardi* (Bonaparte, 1856).

Syntype, RMNH.AVES.87380, adult male, mounted skin. Loc.: Cochinchine [Vietnam]. Leg.: P.M. Diard.

Syntype, RMNH.AVES.87381, adult female, mounted skin. Loc.: Cochinchine [Vietnam]. Leg.: P.M. Diard.

In his description Bonaparte also published the name *Euplocomusdiardi*, a Temminck manuscript name. He intended to substitute this name with his *Diardigallusprelatus*.

***GallusMacartneyi*** Temminck, 1813b: 273.

= *Lophuraignitamacartneyi* (Temminck, 1813).

Syntype, RMNH.AVES.87398, adult male, mounted skin. Loc.: ‘Chine’. Ex: Cabinet Temminck.

The locality ‘Chine’ as written under the stand is obviously erroneous as this taxon occurs in Sumatra as was known to Temminck (1813b: 280). Temminck referred to the first account of this species by G.T. Staunton (1797) based on a specimen presented to Lord Macartney, British Ambassador to China, during his visit to Java in March 1793. This specimen was sent to Shaw in the NHM and is part of the type series. It is not listed in Warren (1966). Temminck described multiple specimens of which only one is still present in the RMNH. Another one was exchanged with the MfN in July 1818.

***Houppiferhoogerwerfi*** Chasen, 1939: 184.

= *Lophurainornatahoogerwerfi* (Chasen, 1939).

Holotype, RMNH.AVES.14026, adult female, skin. Loc.: Telaga Meloewak, Gayu Lues, Sumatra, [Indonesia]. Leg.: A. Hoogerwerf, 24.iv.1937. Ex: MZB, 12.i.1950 (MZB 11744).

Holotype by monotypy. The description was based on a single female. See also Sözer et al. (2006).

***Lophophoruscuvieri*** Temminck, 1820: livr. 1, pl. 1.

= *Lophuraleucomelanoslathami* (J.E. Gray, 1829).

Holotype by monotypy. The specimen collected by Diard and Duvaucel is in the MNHN and confirmed by P. Boussès (in litt., 26 April 2022).

A recommendation to the ICZN is in preparation to suppress *Lophophoruscuvieri* Temminck, 1820, in favour of the junior synonym *PhasianusLathami* J.E. Gray, 1829 (= *Lophuraleucomelanoslathami* (J.E. Gray, 1829), because of prevailing usage. The holotype (see livr. 1, pl. 1), which was historically considered a hybrid in the literature, is here regarded as a composite specimen as (only) the tail does not fit the (adult) Kalij Pheasant *Lophuraleucomelanos*.

***Coturnixperlata*** Temminck, 1815a: 470.

= *Margaroperdixmadagarensis* (Scopoli, 1786).

Possible syntype, RMNH.AVES.224704, adult male, mounted skin. Loc.: Madagascar. Leg.: -.

Temminck based his description on a male in his own cabinet and a similar specimen in the MNHN which is, however, not listed in Voisin et al. (2015).

***Pavocristatusindicus*** Temminck, 1807: 145 (nomen nudum).

***Pavocristatusprimus*** Temminck, 1813b: 26.

***Pavonigripennis*** Sclater, 1860a: 221.

= *Pavocristatus* Linnaeus, 1758.

Syntype, RMNH.AVES.225015, adult male, mounted skin. Ex: Cabinet Temminck.

Sclater based his name on Latham (1823: 114) who based his name on Temminck (1813b). RMNH.AVES.225015 was in Temminck’s collection before 1807 (Jansen 2017) but discussed by Gasso Miracle (2019: 26).

***Francolinuspeli*** Temminck, 1854: 50.

= *Peliperdixlathamilathami* (Hartlaub, 1854).

Syntype, RMNH.AVES.87390, adult male, mounted skin. Loc.: Dabocrom, Côte d’Or [= Ghana]. Leg.: H.S. Pel.

Syntype, RMNH.AVES.87391, adult male, mounted skin. Loc.: Dabocrom, Côte d’Or [= Ghana]. Leg.: H.S. Pel.

Syntype, RMNH.AVES.87392, adult male, mounted skin. Loc.: Sacconde, Côte d’Or [= Sekondi-Takoradi, Ghana], 3 [May] 1841. Leg.: H.S. Pel.

Syntype, RMNH.AVES.87393, immature male, mounted skin. Loc.: Dabocrom, Côte d’Or [= Ghana]. Leg.: H.S. Pel.

Collected during Pel’s stay in Ghana between 1840 and 1855.

***PhasianusDiardi*** Temminck, 1830: livr. 82, text to pl. 486.

= *Phasianusversicolorversicolor* Vieillot, 1825.

Temminck published *Phasianus Diardi* in his resume of *P.versicolor* Vieillot, 1825, based on a specimen sent by Diard to the MNHN. Temminck named it *P. Diardi* in honour of Diard, publishing it as a junior synonym of *P.versicolor* Vieillot, 1825. The holotype is in the MNHN, listed by Voisin et al. (2015: 27) under number C.G. 2013-39.

***PolyplectronChinquis*** Temminck, 1813b: 363.

= *Polyplectronbicalcaratum* (Linnaeus, 1758).

Temminck had seen birds in several menageries prior to the Napoleonic era. He had two males in his own cabinet and saw an immature male in the MNHN. No specimens which fit as types could be found in the RMNH.

***Polyplectronchalcurumscutulatum*** Chasen, 1941: 17.

= *Polyplectronchalcurumscutulatum* Chasen, 1941.

Holotype, RMNH.AVES.14028, adult male, skin. Loc.: Simpang Agoesan, [Gayo Lues], Sumatra, [Indonesia], 07.ii.1937. Ex: MZB, 12.i.1950 (MZB 10176).

***Polyplectronemphanum*** Temminck, 1832: livr. 91, pl. 540.

= *Polyplectronnapoleonis* Lesson, 1831.

The holotype (by monotypy) is in the ANSP (ANSP 12732).

***Francolinusahantensis*** Temminck, 1854: 49.

= *Pternistisahantensis* (Temminck, 1854).

Syntype, RMNH.AVES.87382, adult female, mounted skin. Loc.: Dabocrom, Côte d’Or [= Ghana]. Leg.: H.S. Pel.

Syntype, RMNH.AVES.87383, adult male, mounted skin. Loc.: Dabocrom, Côte d’Or [= Ghana]. Leg.: H.S. Pel.

Syntype, RMNH.AVES.87384, adult male, mounted skin. Loc.: Dabocrom, Côte d’Or [= Ghana]. Leg.: H.S. Pel.

Syntype, RMNH.AVES.87385, immature male, mounted skin. Loc.: Dabocrom, Côte d’Or [= Ghana]. Leg.: H.S. Pel.

***Perdixadansonii*** Temminck, 1815a: 305.

= *Pternistisbicalcaratusbicalcaratus* (Linnaeus, 1766).

Possible syntype, RMNH.AVES.224678, adult male, mounted skin. Loc.: Senegambie. Leg.: -.

Temminck described three specimens: one in the NHM, one in the RMNH and one in Bullock’s collection, one of which was sent by Temminck to the NMW (NMW 1821.LXXIII.56).

***Perdixclamator*** Temminck, 1815a: 298.

= *Pternistiscapensis* (Gmelin, 1789).

Possible syntype, RMNH.AVES.224672, adult male, mounted skin. Loc.: Cap, [South Africa]. Leg.: -.

Temminck (1815a: 304) referred to male specimens in his cabinet, a male in the collection of Raye and a female in the MNHN.

***Perdixerckelii*** Rüppell, 1835: 12, pl. 6.

= *Pternistiserckelii* (Rüppell, 1835).

Paralectotype, RMNH.AVES.90934.

The lectotype was designated by Steinbacher (1949: 105) and is in SMF (SMF 12600). Paralectotypes are in the NHM (Steinheimer 2005a: 238; NHMUK 1837.6.10.699, 1837.6.10.700).

***Perdixrubricollis*** Cretzschmar, 1829: 44, pl. 30.

= *Pternistisleucoscepusleucoscepus* (Gray, 1867).

Possible syntype, RMNH.AVES.148207, adult male, mounted skin. Loc.: Abyssinia. Leg.: W.P.E.S. Rüppell.

Possible syntype, RMNH.AVES.148208, adult female, mounted skin. Loc.: Massawa, Abyssinia [Eritrea]. Leg.: W.P.E.S. Rüppell.

Steinheimer (2005a) listed both as possible syntypes.

***PerdixLongirostris*** Temminck, 1815a: 323.

= *Rhizotheralongirostris* (Temminck, 1815).

Syntype, RMNH.AVES.87401, adult male, mounted skin. Loc.: Sumatra, [Indonesia]. Ex: Museum Batavia.

Syntype, RMNH.AVES.87402, adult female, mounted skin. Loc.: Sumatra, [Indonesia]. Ex: Museum Batavia.

Temminck wrote having received three males and two females and that a male and female were in the collection of Raye van Breukelerwaert. No other specimens in the RMNH collection fit as types. Temminck published this name first as a nomen nudum in his Catalogue systématique (1807: 158, no. 445) based on a male, already referring to the forthcoming formal description (Temminck 1815a).

***Francolinusjugularis*** Büttikofer, 1889b: 76.

= *Scleroptilagutturalisjugularis* (Büttikofer, 1889).

Syntype, RMNH.AVES.87386, adult male, skin. Loc.: Gambos, [Angola], 25.ii.1888. Leg.: P.J. van der Kellen.

Syntype, RMNH.AVES.87387, adult female, skin. Loc.: Gambos, [Angola], 25.ii.1888. Leg.: P.J. van der Kellen.

***Perdix Le Vaillantii*** Temminck, 1823: livr. 7–10, text to pl. 1.

***Perdixvaillantii*** Temminck, 1829: livr. 80, pl. 477.

= *Scleroptilalevaillantiilevaillantii* (Valenciennes, 1825).

Syntype, RMNH.AVES.87394, adult, sex unknown, mounted skin. Loc.: “Cap”, [South Africa]. Leg.: -.

Syntype, RMNH.AVES.87395, adult female, mounted skin. Loc.: “Cap”, [South Africa]. Leg.: -.

Syntype, RMNH.AVES.87396, adult male, mounted skin. Loc.: Palmiet River, Cape, [South Africa]. Leg.: -.

Temminck first mentioned the name *Perdix Le Vaillantii* in a footnote to the text for pl. 1 (1823; see Dickinson 2012: 45) and referred to the description following later (Temminck 1829: pl. 477). In the text and footnote a very short description is given (only collector and locality, and some information on the characteristic morphology of the bill). Following the rule of priority this name should be attributed to Temminck with publication date 1823 and not to Valenciennes (1825).

According to the description with pl. 477 other possible syntypes are in the MNHN, but are not listed in Voisin et al. (2015).

***Phasianusveneratus*** Temminck, 1830: livr. 82, pl. 485.

= *Syrmaticusreevesii* (Gray, 1829).

Possible syntype, RMNH.AVES.224949, adult male, mounted skin. Loc.: China. Leg.: -.

Temminck based his description on two males in his collection of which RMNH.AVES.224949 fits pl. 485. The other male, which Temminck described having a much shorter tail, seems no longer to be present in the RMNH.

***Phasianussœmmerringii*** Temminck, 1830: livr. 82, pl. 487 (male) and pl. 488 (female).

= *Syrmaticussoemmerringiisoemmerringii* (Temminck, 1830).

Syntype, RMNH.AVES.87406, adult female, mounted skin. Loc.: Japan. Leg.: H. Bürger.

Syntype, RMNH.AVES.87407, adult male, mounted skin. Loc.: Japan. Leg.: H. Bürger, “1841”.

Temminck mentioned that he had received several specimens from Von Siebold. There are no mounted specimens in the RMNH referring to Von Siebold on the label or written under the stand. Two specimens refer, in Temminck’s handwriting, to Bürger, the assistant and successor of Von Siebold. Bürger left Japan in 1834. RMNH.AVES.87407 also has “1841” written on the label, which we consider to be an error. For more information on Siebold’s collection, see Holthuis and Sakai (1970), Dekker et al. (2001) and Morioka et al. (2005). Two were sent to the MfN by March 1833 and one to the SMF in August 1830.

Schifter et al. (2007: 99) listed two possible syntypes in the NMW (NMW 1269 and 1270) received from Temminck in 1833.

Dickinson (2001: 44) proposed livraison 82 (January 1830) for both plates.

### ﻿ANSERIFORMES

#### ﻿Anatidae

**Anas (Mareca) gibberifrons** Müller, 1842: 159.

= *Anasgibberifrons* Müller, 1842.

Syntype, RMNH.AVES.87318, adult male, mounted skin. Loc.: Prittie [Pariti], Timor, [Indonesia], xii.1828. Leg.: S. Müller.

Syntype, RMNH.AVES.87319, adult female, mounted skin. Loc.: Atapoepoe, Timor, [Indonesia], iii.1829. Leg.: S. Müller.

Syntype, RMNH.AVES.87320, adult female, mounted skin. Loc.: Makassar, Celebes [= Sulawesi, Indonesia] [iii 1828]. Leg.: S. Müller.

Syntype, RMNH.AVES.87321, adult male, mounted skin. Loc.: Menado, Celebes [= Sulawesi, Indonesia]. Leg.: E.A. Forsten.

Syntype, RMNH.AVES.87322, adult male, mounted skin. Loc.: Gorontalo, Celebes [= Sulawesi, Indonesia]. Leg.: E.A. Forsten.

Syntype, RMNH.AVES.87323, adult male, mounted skin. Loc.: Tondano, Celebes [= Sulawesi, Indonesia], vii.1840. Leg.: E.A. Forsten.

Syntype, RMNH.AVES.87324, adult female, mounted skin. Loc.: Pegoiat [= Paguat], Celebes [= Sulawesi, Indonesia], xi.1841. Leg.: E.A. Forsten.

Syntype, RMNH.AVES.87325, immature, mounted skin. Loc.: Gorontalo, Celebes [= Sulawesi, Indonesia], 1.x.1844. Leg.: E.A. Forsten.

The year 1844 for RMNH.AVES.87325 is obviously an error as Forsten died in 1843. Forsten stayed in Celebes twice between March 1840 and April 1842.

***AnasSalvadorii*** Büttikofer, 1896b: 59.

= *Anasplatyrhynchosplatyrhynchos* Linnaeus, 1758.

Holotype, RMNH.AVES.87326, adult, sex unknown, skin. Loc.: Sumba, [Indonesia]. Leg.: Colfs.

Holoype by monotypy. Büttikofer described how he “found a Duck from the Island of Sumba”. Büttikofer’s description is based on a domestic duck.

***Anserbrachyrhynchus*** Baillon, 1834: 74.

= *Anserbrachyrhynchus* Baillon, 1834.

Syntype, RMNH.AVES.87331, immature, mounted skin. Loc.: Picardie, France. Leg.: L.A.F. Baillon.

The MLC houses a possible syntype (MLC.2011.0.560).

***Ansercygnoidesferus*** Temminck & Schlegel, 1850: 140.

= *Ansercygnoides* (Linnaeus, 1758).

Holotype, RMNH.AVES.87329, adult, sex unknown, mounted skin. Loc.: Japan. Leg.: -, 1844.

Holotype by monotypy. Temminck and Schlegel (1850) wrote that they received a single specimen.

The name *A.ferus* was neither mentioned on pl. 81 published in 1849 (Holthuis and Sakai 1970) or 1850 (Mlíkovský 2012b: 117) nor in the description but only in the index to the plates on page 140 which was published in 1850 (see Morioka et al. 2005).

***Ansermedius*** Temminck, 1840: 519.

= *Ansererythropus* (Linnaeus, 1758).

Holotype, RMNH.AVES.87330, adult, sex unknown, mounted skin. Loc.: Europe, xi.1819. Leg.: C.J. Temminck.

Holotype by monotypy. Temminck described a single bird shot by him.

***Anasscutulata*** Müller, 1842: 159.

= *Asarcornisscutulata* (Müller, 1842).

Syntype, RMNH.AVES.87138, adult male, mounted skin. Loc.: Lake Gorong, Java, [Indonesia]. Leg.: S. Müller.

Syntype, RMNH.AVES.87202, adult male, mounted skin. Loc.: Lake Gorong, Java, [Indonesia]. Leg.: S. Müller.

Syntype, RMNH.AVES.87332, adult male, mounted skin. Loc.: Lake Gorong, Java, [Indonesia]. Leg.: S. Müller.

Since Müller explicitly mentioned a lake at the foot of Mt. Salak, Java as the only collecting locality of this new species, a fourth specimen from “Buitenzorg” is probably not part of the type series even though it was also collected by Müller.

***Nyrocaaustralislebeboeri*** Bartels Jr & Franck, 1938: 337.

= *Aythyaaustralis* (Eyton, 1838).

Holotype, RMNH.AVES.14018, adult male, skin. Loc.: Lake Tundjung, Java, [Indonesia], 3.vi.1937. Leg.: J.M. ten Cate. Ex: MZB, 12.i.1950 (MZB 7791, don.: A.J.M. Ledeboer).

Holotype by monotypy. The name *lebeboeri* is clearly a lapsus, since the species was named after A.J.M. Ledeboer.

***Dendrocygnaguttata*** “Forsten” Schlegel, 1866e: 85.

= *Dendrocygnaguttata* Schlegel, 1866.

Lectotype, RMNH.AVES.87333, adult male, mounted skin. Loc.: Celebes [= Sulawesi], [Indonesia], [iii.1840–iv.1842]. Leg.: E.A. Forsten.

Paralectotypes, RMNH.AVES.88958–88971, 88973–88982, 258109 (formerly 88972).

Van den Hoek Ostende et al. (1997: 49) listed RMNH.AVES.87333 as holotype, the other specimens as paratypes. This was in error, as Schlegel did not nominate a holotype: he referred to RMNH.AVES.87333 as the type of the manuscript name by Forsten. Therefore, all specimens listed in his original description should be considered syntypes. However, by listing RMNH.AVES.87333 as holotype, Van den Hoek Ostende et al. (1997: 49) unintentionally designated it the lectotype.

Forsten visited Celebes twice between March 1840 and April 1842. RMNH.AVES.88962 is erroneously labelled 1860 instead of 1861.

***Nettapuspulchellus*** Gould, 1842a: 89.

= *Nettapuspulchellus* Gould, 1842.

Paralectotypes, RMNH.AVES.90978 and 90979.

The lectotype was selected by Stone (1913: 143) which is in the ANSP (ANSP 5972, male. Loc.: Port Essington).

In the original description, Gould made no reference to specimens. However later Gould (1842c: text to pl. 4) wrote that his description was based on four specimens: two collected by Gilbert, one by Bynoe and one by an unknown collector. The two RMNH specimens came to Leiden prior to Temminck’s death in 1858 as text in his hand on the stand refers to Gilbert as collector. See also Mees (1982: 23) and Fisher and Calaby (2009: 84), who listed these specimens as probable types.

***Anascyanoptera*** Hartlaub, 1855: 357.

***QuerquedulaHartlaubii*** Cassin, 1859: 175 (nomen novum).

***Anascuprea*** Schlegel, 1866e: 62 (nomen novum).

= *Pteronettahartlaubii* (Cassin, 1859).

Holotype, RMNH.AVES.87334, adult female, mounted skin. Loc.: Dabocrom [error for Rio Boutry?], Côte d’Or [= Ghana]. Leg.: H.S. Pel.

Holotype by monotypy. Hartlaub referred to a single male collected at Rio Boutry, Ghana. According to Schlegel and later labels, this specimen is a female collected at Dabocrom, Ghana. Mees (2003) corrected the type locality to Rio Boutry, as referred to by Hartlaub in his original description.

*Quercedulahartlaubii* Cassin, 1859 and *Anascuprea* Schlegel, 1866 were both introduced as nomen novum for *Anascyanoptera* Hartlaub, 1855, which is preoccupied by *Anascyanoptera* Vieillot, 1816.

**Anas (Querquedula) humeralis** Müller, 1842: 159.

= *Spatulaquerquedula* (Linnaeus, 1758).

Syntype, RMNH.AVES.87327, adult male, mounted skin. Loc.: [Pamanukan], Java, [Indonesia], [iv.1832]. Leg.: S. Müller

Syntype, RMNH.AVES.87328, adult female, mounted skin. Loc.: [Pamanukan], Java, [Indonesia], [iv.1832.]. Leg.: S. Müller.

### ﻿CAPRIMULGIFORMES

#### ﻿Podargidae

***Batrachostomusparvulus*** Bonaparte, 1850a: 57.

= *Batrachostomusaffinisaffinis* Blyth, 1847.

Lectotype, RMNH.AVES.88362, adult male, relaxed mount. Loc.: Kapoeas, Borneo, [Indonesia]. Leg.: C.A.L.M. Schwaner.

Paralectotypes, RMNH.AVES.88363 and 88364.

There are two species represented in the type series. The male from Borneo (RMNH.AVES.88362) is *B.affinisaffinis* Blyth, 1847, the other two specimens are *B.stellatus* (Gould, 1837). Stresemann (1937) selected the male from Borneo as lectotype. Mees (1986) argued that this selection was invalid, since Stresemann referred to *B.parvulus* Schlegel, 1857. It is, however, clear that Schlegel’s *parvulus* referred to *B.parvulus* Bonaparte, 1850. We therefore consider Stresemann’s lectotype selection valid, making *B.parvulus* Bonaparte, 1850 a junior synonym of *B.affinis* Blyth, 1847.

***Podarguscornutus*** Temminck, 1822: livr. 27, pl. 157 (nomen novum).

= *Batrachostomuscornutuscornutus* (Temminck, 1822).

Nomen novum for *Podargus Javensis* Horsfield, 1822. Temminck based this name on the description by Horsfield and a single specimen from Sumatra in the MNHN, which is not part of the type series. The holotype for *P. Javensis* and subsequently *P.cornutus* is in the NHM (BMNH 1880.1.1.4764).

***Batrachostomusjavensislongicaudatus*** Hoogerwerf, 1962: 195.

= *Batrachostomuscornutuslongicaudatus* Hoogerwerf, 1962.

Paratype, RMNH.AVES.27880.

The holotype is in the MZB (MZB 23136; Sudaryanti et al. 2006: 7).

***Batrachostomuspoliolophus*** “Temminck” Hartert, 1892a: 63.

= *Batrachostomuspoliolophus* Hartert, 1892.

Holotype, RMNH.AVES.88365, adult female, skin. Loc.: Padang, Sumatra, [Indonesia], 1837. Leg.: L. Hörner.

Holotype by monotypy. Hartert based his description on a single specimen in the RMNH.

***Podargusintermedius*** Hartert, 1895: x.

= *Podargusocellatusintermedius* Hartert, 1895.

Paralectotype, RMNH.AVES.88366.

Hartert nominated specimens from Kiriwina as type. This excludes RMNH.AVES.88367 from Ferguson Island, listed in Van den Hoek Ostende et al. (1997: 162), from the type series. Later Hartert designated the lectotype (Hartert 1925: 157).

#### ﻿Caprimulgidae

***Caprimulgusaegyptius*** Lichtenstein, 1823: 59.

***Caprimulgusisabellinus*** Temminck, 1825: livr. 64, pl. 379 (nomen novum).

= *Caprimulgusaegyptiusaegyptius* Lichtenstein, 1823.

Syntype, RMNH.AVES.88370, adult male, relaxed mount. Loc.: “Nubia” [error for Egypt]. Ex: MfN, 5.iii.1825.

RMNH.AVES.88370 was received on 5 March 1825 from the MfN. Lichtenstein described and only listed specimens from “Aegypt. super.” [Upper Egypt]. RMNH.AVES.88370 has “Nubia” as locality, which might indicate that there has been a mix up with the data either in Berlin or Leiden.

Temminck introduced *C.isabellinus* as an unnecessary nomen novum for *C.aegyptius* Lichtenstein, 1823. He considered the reference to Egypt in the scientific name incorrect as this species also occurs outside Egypt as well as the fact that other species occur in Egypt.

The MfN houses a series of syntypes for *C.aegyptius* (Cleere et al. 2005: 204) which are also the types for *C.isabellinus* Temminck, 1825.

***Caprimulgusconcretus*** Bonaparte, 1850a: 60.

= *Caprimulgusconcretus* Bonaparte, 1850.

Syntype, RMNH.AVES.88372, adult male, relaxed mount. Loc.: Borneo, [Indonesia]. Leg.: P.M. Diard.

Syntype, RMNH.AVES.88373, adult, sex unknown, relaxed mount. Loc.: Borneo, [Indonesia]. Leg.: P.M. Diard.

Syntype, RMNH.AVES.88374, adult female, relaxed mount. Loc.: Borneo, [Indonesia]. Leg.: C.A.L.M. Schwaner.

Syntype, RMNH.AVES.88375, immature female, relaxed mount. Loc.: Borneo, [Indonesia]. Leg.: -.

Bonaparte (1850a) incorrectly gave “Ashantee” (Ghana, West Africa) as the type locality. RMNH.AVES.88375 is a male according to N. Cleere (pers. comm., 2.viii.1993).

***Caprimulgussmithi*** Bonaparte, 1850a: 59.

= *Caprimulguseuropaeuseuropaeus* Linnaeus, 1758.

Syntype, RMNH.AVES.88376, adult female, relaxed mount. Loc.: Algoa Bay, South Africa. Leg.: -.

Syntype, RMNH.AVES.88377, adult female, relaxed mount. Loc.: Cape, South Africa. Leg.: -.

***Caprimulguseximius*** “Rüppell” Temminck, 1826: livr. 67, pl. 398.

= *Caprimulguseximiuseximius* Temminck, 1826.

Holotype, RMNH.AVES.88378, adult male, relaxed mount. Loc.: Sennaar, Ethiopia. Leg.: W.P.E.S. Rüppell. Ex: SMF.

Holotype by monotypy.

Originally ‘Nubie’ was written under the stand, later changed into ‘Sennaar’.

***Caprimulgusjotaka*** Temminck & Schlegel, 1844: pl. 12 and 13, p. 37.

= *Caprimulgusjotakajotaka* Temminck & Schlegel, 1845.

Syntype, RMNH.AVES.88379, adult male, relaxed mount. Loc.: Japan. Leg.: H. Bürger.

Syntype, RMNH.AVES.88380, adult male, relaxed mount. Loc.: Japan. Leg.: H. Bürger.

Syntype, RMNH.AVES.88381, adult female, relaxed mount. Loc.: Japan. Leg.: H. Bürger.

Plates 12 and 13 appeared in 1844, the description on p. 37 one year later (Holthuis and Sakai 1970). For more information on dating and Siebold’s collection see Dekker et al. (2001). Temminck and Schlegel wrote that they had four specimens, two males and two females. The whereabouts of one female is unknown.

In this catalogue we follow Holthuis and Sakai (1970) over Mlíkovský (2012b) for the year of publication. This can have consequences on the status and selection of type specimens for this name if the plate was published prior to the description in the livraison. Taxa based upon holotypes are not affected, but will differ where the name is based upon a series of specimens.

***Caprimulgusinornatus*** Heuglin, 1869: 129.

= *Caprimulgusinornatus* Heuglin, 1869.

Syntype, RMNH.AVES.88382, adult male, skin. Loc.: Keren, Bogosland, N.E. Africa [= Ethiopia], vii.1861. Leg.: T. von Heuglin. Ex: SMNS](Kraus) (4841), 1862.

The remaining two syntypes are in the SMNS (SMNS 4840 and 4842).

***Caprimulgusritae***King et al. 2023 (in press).

= not yet included in IOC 12.2.

Holotype, RMNH.AVES.162472, adult female. Loc.: Wetar, Indonesia, 7.iii.1898. Leg.: C. Schädler (212).

Paratype, RMNH.5070058.

According to G. Sangster (pers. comm., June 2022), this new taxon was considered to belong to *Caprimulgusmacrurusschlegelii* A.B. Meyer, 1874. Other paratypes are in the AMNH (AMNH 632998–632999).

***Caprimulgusmeesi*** Sangster & Rozendaal, 2004: 30.

= *Caprimulgusmeesi* Sangster & Rozendaal, 2004.

Holotype, RMNH.AVES.75297, adult male, skin. Loc.: Nisar, Flores, [Indonesia], 15.ix.1975. Leg.: E. Schmutz.

The paratype, an adult male collected by G. Stein at Mao Marru, Sumba, is in the AMNH (AMNH 346702).

***Caprimulgusnubicus*** Lichtenstein, 1823: 59.

= *Caprimulgusnubicusnubicus* Lichtenstein, 1823.

Syntype, RMNH.AVES.90754, adult, sex unknown, relaxed mount. Loc.: Nubia [= Sudan]. Leg.: [W.F. Hemprich and C.G. Ehrenberg]. Ex: MfN, 05.iii.1824.

According to acquisition lists in the RMNH archives, this specimen was received from the MfN on 5 March 1824. Since this transfer only contained specimens offered by the MfN through Lichtenstein’s “Verzeichnis der Doubletten”, RMNH.AVES.90754 does belong to the type series. At least three syntypes are in the MfN (ZMB 8951–8953; Cleere et al. 2005: 204). Schifter et al. (2007: 206) listed a syntype in the NMW (NMW 42205).

***Antrostomusdominicus*** Bonaparte, 1850a: 61.

= *Caprimulguspectoralispectoralis* Cuvier, 1816.

Syntype, RMNH.AVES.88384, adult male, relaxed mount. Loc.: “Haiti” [error for South Africa]. Leg.: A. Ricord [error?].

Syntype, RMNH.AVES.88385, adult female, relaxed mount. Loc.: “Haiti” [error for South Africa]. Leg.: -.

Haiti is an error since *C.pectoralis* only occurs in Africa. Later ‘South Africa’ was added to the label.

***CaprimulgusBartelsi*** Finsch, 1902: 148.

= *Caprimulguspulchellusbartelsi* Finsch, 1902.

Holotype, RMNH.AVES.65036, adult female, skin. Loc.: Pangerango, Preanger, Java, [Indonesia], 13.x.1901. Ex: M. Bartels (977), 01.vi.1954.

***Caprimulgusruficollis*** Temminck, 1820: 438.

= *Caprimulgusruficollisruficollis* Temminck, 1820.

Temminck based his description on two specimens in the collection of the NMW, where Schifter et al. (2007: 205–206) listed two syntypes collected by Natterer in Algeciras, Spain (NMW 5509 and 42204).

***Caprimulgusdiurnus*** Wied, 1821: 174.

= *Chordeilesnacundanacunda* (Vieillot, 1817).

Syntype, RMNH.AVES.88416, adult, sex unknown, relaxed mount. Loc.: [Inhobim, Bahia], Brazil, [8.ii.1817]. Leg.: A.P.M. zu Wied-Neuwied.

Greenway (1978: 146) listed another syntype in the AMNH (AMNH 6838) as the holotype of *C.diurnus* Wied, 1830, not Wied, 1821. Therefore, this is not a valid lectotype designation. Van den Hoek Ostende et al. (1997: 166) listed RMNH.AVES.88416 along with RMNH.AVES.88414 and 88415 as syntypes for *C.diurnus* Temminck, 1823, a junior primary homonym of Wied’s name. They must have overlooked the earlier publication by Wied as did Greenway. As the latter two specimens were not collected by Wied, they have no type status.

According to Wied he found this species between Vareda (= Inhobim) and Ressaque (= Resacca), 8 February 1817 (Moraes 2009: 30).

***Eurostopus* [sic] *argus*** Hartert, 1892b: 608.

= *Eurostopodusargus* Hartert, 1892.

Paralectotypes, RMNH.AVES.88389 and 88390.

Hartert based his name on a nomen nudum by Von Rosenberg (1867: 37), therefore Rosenberg’s specimens are included in the type series. Listed as *E.guttatusguttatus* (Vigors & Horsfield, 1826) in Peters (1940; Vol. IV: 190), which name was published in 1827 based on a misidentified *E.mystacalis* (Temminck, 1826) (see Holyoak 2001: 292). See Schodde and Mason (1997: 324) for discussion on lectotype selection by Mathews.

***Caprimulgusmystacalis*** Temminck, 1826: livr. 69, pl. 410.

= *Eurostopodusmystacalis* (Temminck, 1826).

Lectotype, RMNH.AVES.88387, adult, sex unknown, relaxed mount. Loc.: Australia. Leg.: -.

Listed as *Eurostopodusalbogularisalbogularis* (Vigors & Horsfield, 1826) in Peters (1940; Vol. IV: 190), which name was published in 1827, one year after Temminck’s publication (Holyoak 2001: 298). Temminck’s description gives no indication about the number of specimens. The listing in Van den Hoek Ostende et al. (1997: 164) as holotype was in error but constituted a lectotype designation.

***Caprimulguspapuensis*** Schlegel, 1866f: 340.

= *Eurostopoduspapuensis* (Schlegel, 1866).

Syntype, RMNH.AVES.88401, adult male, skin. Loc.: Sorong, [Papua, Indonesia], 04.xii.1864. Leg.: H.A. Bernstein.

Syntype, RMNH.AVES.88402, adult male, skin. Loc.: Salawatti, [Indonesia], 25.ii.1865. Leg.: H.A. Bernstein.

***Hydropsalistrifurcatus*** Von Tschudi, 1846: 129.

= *Hydropsalisclimacocercaclimacocerca* (Tschudi, 1844).

Syntype, RMNH.AVES.88405, adult male, skin. Loc.: Baraneira [= Bananeira], Brazil, 08.ix.1821. Leg.: J. Natterer. Ex: NMW, 1864.

***Caprimulguspsalurus*** Temminck, 1822: livr. 27, pl. 157 and 158.

= *Hydropsalistorquatatorquata* (Gmelin, 1789).

Syntype, RMNH.AVES.88403, adult female, skin. Loc.: Brazil. Leg.: -.

Syntype, RMNH.AVES.88404, adult male, skin. Loc.: Brazil. Leg.: -.

Although not mentioned in Schifter et al. (2007), five syntypes are in the NMW (NMW 10173, 41591–41593, 41595).

According to Natterer’s handwritten register cards in the NMW, three specimens were sent to the RMNH, two of which fit as type: one specimen from the third delivery which arrived in the NMW in 1821 and a female collected by Natterer in Ipanema in August 1821 which was probably also sent to the NMW within the third delivery. However, they probably did not belong to the specimens Temminck had seen in the RMNH when he described his new taxon. The female was sent to the RMNH as late as in 1864. According to the original description other syntypes are in the MNHN. For dating of the ‘Planches Coloriées’ and pl. 157 see Dickinson (2001:47).

***CaprimulgusNattererii*** Temminck, 1822: livr. 18, pl. 107.

= *Lurocalissemitorquatusnattererii* (Temminck, 1822).

Holotype, RMNH.AVES.88406, adult male, relaxed mount. Loc.: Brazil. Leg.: -.

Following Dickinson et al. (2022) (see also Introduction of this catalogue under “What is new in this 2023 version”: 14) we have selected the holotype based on the plate since the text that appeared on 22 May 1824 is irrelevant regarding type status. Any listing of syntypes for this name in Van den Hoek Ostende et al. (1997: 165) or any other type catalogue is erroneous. The following specimens have therefore no type status: RMNH.AVES.88407 and 88408 (Van den Hoek Ostende et al. 1997: 165); NMW 41567–41569, NMW 43962 (Schifter et al. 2007: 202–203); ZMB 8922 and 8924 (Cleere et al. 2005: 203) and any specimens in the MNHN referred to by Temminck (1824).

***Lyncornismacrotisjacobsoni*** Junge, 1936: 39.

= *Lyncornismacrotisjacobsoni* Junge, 1936.

Holotype, RMNH.AVES.88391 (6651), adult female, skin. Loc.: Sinabang, Simalur [= Simeulue], [Indonesia], 28.iii.1913. Leg.: E.R. Jacobson and W.C van Heurn.

Paratypes, RMNH.AVES.88392–88398.

***Lyncornismacropterus*** “Temminck” Bonaparte, 1850a: 62.

= *Lyncornismacrotismacropterus* Bonaparte, 1850.

Syntype, RMNH.AVES.88399, adult female, relaxed mount. Loc.: Pagoeat, Celebes [= Sulawesi], [Indonesia], [15.iv.1840–19.vi.1841]. Leg.: E.A. Forsten.

Syntype, RMNH.AVES.88400, adult male, relaxed mount. Loc.: Tondano, Celebes [= Sulawesi], [Indonesia], [15.iv.1840–19.vi.1841]. Leg.: E.A. Forsten.

Forsten visited the Tondano area between 15 April 1840 and 19 June 1841.

***LyncornisTemminckii*** Gould, 1838b: pl. XVI and text.

***Lyncornisimberbis*** “Temminck” Gould, 1838b: pl. XVI and text.

= *Lyncornistemminckii* Gould, 1838.

Syntype, RMNH.AVES.199026, adult male, relaxed mount. Loc.: Borneo, [Indonesia], [28.vii–17.xii.1836]. Leg.: S. Müller.

Gould based his description on several specimens in his own collection and on a bird sent by Temminck under the manuscript name *L.imberbis*. A name Gould published in synonymy of *L.temminckii*. Müller visited Borneo between 28 July and 17 December 1836.

***Hydropsaliscreagra*** Bonaparte, 1850a: 58.

= *Macropsalisforcipata* (Nitsch, 1840).

Syntype, RMNH.AVES.88409, adult male, relaxed mount. Loc.: Brazil. Leg.: -.

Syntype, RMNH.AVES.88410, adult male, relaxed mount. Loc.: Brazil. Leg.: -.

***Nyctidromusgrallarius*** “Wied” Bonaparte, 1850a: 62.

= *Nyctidromusalbicollisalbicollis* (Gmelin, 1789).

Syntype, RMNH.AVES.88411, adult male, relaxed mount. Loc.: Brazil. Leg.: -.

Syntype, RMNH.AVES.88412, adult female, relaxed mount. Loc.: Brazil. Leg.: J. Natterer.

***Caprimulgusminutus*** “Natterer” Bonaparte, 1850a: 63.

= *Nyctiprogneleucopyga* (Spix, 1825).

Possible holotype, RMNH.AVES.88413, adult male, skin. Loc.: Brazil. Leg.: -.

Bonaparte based his description on a manuscript name by Natterer and a specimen in the NMW referred to as “Avis junior?” [immature?]. No types for *C.minutus* are listed in Schifter et al. (2007), but the listing of RMNH.AVES.88413 as holotype by Van den Hoek Ostende et al. (1997: 166) is questionable without more information about its provenance.

Originally this specimen was sexed as female, later changed into male on the label.

***Caprimulgusbinotatus*** “Temminck” Bonaparte, 1850a: 60.

= *Velesbinotatus* (Bonaparte, 1850).

Lectotype, RMNH.AVES.88417, adult male, skin. Loc.: Ashante, Dabocrom, Gold Coast [= Ghana]. Leg.: H.S. Pel, 1841.

Bonaparte erroneously gave “Borneo” as collecting locality and gave no indication of the number of specimens available to him. The listing in Van den Hoek Ostende et al. (1997: 167) as holotype was in error, but constituted a lectotype designation.

### ﻿APODIFORMES

#### ﻿Aegothelidae

***Batrachostomuscrinifrons*** “Temminck” Bonaparte, 1850a: 57.

= *Aegothelescrinifrons* (Bonaparte, 1850).

Lectotype, RMNH.AVES.88368, adult female, relaxed mount. Loc.: Dodinga, Halmahera, [Indonesia], [19.vi–ix], 1841. Leg.: E.A. Forsten.

Bonaparte gave no indication of the number of specimens available to him. The listing in Van den Hoek Ostende et al. (1997: 162) as holotype was in error, but constituted a lectotype designation.

During a forced stay due to illness on Ternate from 19 June 1841 until mid- September 1841, Forsten sent his hunters to Halmahera to collect skins.

***Caprimulgusbrachyurus*** Von Rosenberg, 1866: 143.

= *Aegotheleswallacii* G.R. Gray, 1859.

Holotype, RMNH.AVES.88369, immature, sex unknown, relaxed mount. Loc.: Wonumbai [= Sungai Manumbai], Kobror Island, Aru Islands [Indonesia], v.1865. Leg.: C.B.H. von Rosenberg.

Van den Hoek Ostende et al. (1997: 162) listed Schlegel as the author of *C.brachyurus*. However, according to Mees (1980) this name was published first by Von Rosenberg. This is confirmed by Schlegel (1866f: 340) who referred to Von Rosenberg as the author of *C.brachyurus*.

Von Rosenberg (1866) referred to his specimen as a female and did not indicate the number of specimens. Schlegel (1866f: 34) mentioned that Von Rosenberg applied the name to a single immature specimen collected by himself on the Aru Islands. RMNH.AVES.88369 is therefore holotype by monotypy.

#### ﻿Hemiprocnidae

***Cypseluscomatus*** Temminck, 1824: livr. 45, pl. 268.

= *Hemiprocnecomatacomata* (Temminck, 1824).

Schifter et al. (2007: 216) listed a possible syntype in the NMW. However, lacking collecting data, no final decision could be made whether the specimen indeed belongs to the type series. Temminck also referred to the RMNH but no specimen(s) are present which fit the description.

***Dendrochelidonschisticolor*** Bonaparte, 1850a: 66.

= *Hemiprocnecoronata* (Tickell, 1833).

Syntype, RMNH.AVES.88451, adult male, mounted skin. Loc.: Nepal. Leg.: B.H. Hodgson.

Syntype, RMNH.AVES.88458, adult female, mounted skin. Loc.: Nepal. Leg.: B.H. Hodgson.

***Cypseluslongipennis*** Temminck, 1821: livr. 14, pl. 83.

= *Hemiprocnelongipennislongipennis* (Rafinesque, 1802).

Temminck’s name is considered here to be a younger homonym of *longipennis* Rafinesque as Temminck made no reference to Rafinesque.

Following Dickinson et al. (2022) (see also Introduction of this catalogue under “What is new in this 2023 version”: 14), who did not include *longipennis* Temminck as they considered Rafinesque to be the first describer, the holotype is the specimen illustrated on pl. 83, since the text that appeared later is irrelevant regarding type status. Temminck did not indicate in which collection the illustrated specimen was kept. The RMNH has none that fits the plate. A specimen from Java in the NMW (NMW 66895), received in exchange in 1821 from the RMNH and listed as syntype by Schifter et al. (2007: 216), might prove to be the holotype.

#### ﻿Apodidae

***Collocaliaceramensis*** van Oort, 1911c: 64.

= *Aerodramusceramensis* (van Oort, 1911).

Holotype, RMNH.AVES.14990, adult male, skin. Loc.: Kwalara, Ceram, [Indonesia], 26.iv.1910. Leg.: F.K. van Dedem (330).

***Collocaliafrancicabartelsi*** Stresemann, 1927: 46.

= *Aerodramusfuciphagusfuciphagus* (Thunberg, 1812).

Holotype, RMNH.AVES.88428, adult female, skin. Loc.: [Muara Wettan, Java, Indonesia], [13.ix.1914]. Leg.: [M. Bartels]. Ex: Bartels (9810).

The data listed above have been taken from Stresemann (1927) as they are lacking on the label.

***Collocaliafrancicasororum*** Stresemann, 1931: 12.

= *Aerodramussororum* (Stresemann, 1931).

Paratypes, RMNH.AVES.90897 and 90898.

The holotype is in the AMNH (AMNH 292443) and five paratypes in the MfN (ZMB 34.2250–34.2252, 34.2254 and 34.2255).

***Collocaliabrevirostrisvulcanorum*** Stresemann, 1926: 352.

= *Aerodramusvulcanorum* (Stresemann, 1926).

Holotype, RMNH.AVES.88429, adult female, skin. Loc.: [Gedeh] West Java, [Indonesia]. Leg.: M. Bartels (13273).

The data listed above have been taken from Stresemann (1926). There is no data on the label of this specimen.

***Cypselusbarbatus*** Sclater, 1866: 599.

= *Apusbarbatusbarbatus* (Sclater, 1866).

Syntype, RMNH.AVES.88419, adult, sex unknown, relaxed mount. Loc.: Cap, South Africa. Ex: Cabinet Temminck.

Sclater based this name on two specimens labelled “*Cypselusbarbatus*” in the RMNH. Apart from RMNH.AVES.88419, which is specifically indicated as a type specimen, the museum possesses two *A.barbatus* specimens collected by Van Horstok in South Africa, which were present in the museum at the time of description. Whether or not one of these is the second specimen referred to by Sclater cannot be decided, since these specimens no longer bear the original labels.

***Cypseluspygargus*** Temminck, 1828: livr. 77, pl. 460, fig. 1.

= *Apuscaffer* (Lichtenstein, 1823).

Syntype, RMNH.AVES.88420, adult male, relaxed mount. Loc.: Cap, South Africa. Ex: Cabinet Temminck.

Syntype, RMNH.AVES.88421, adult female, relaxed mount. Loc.: Cap, South Africa. Ex: Cabinet Temminck.

Although not mentioned in the original description, Schifter et al. (2007: 215) listed another syntype in the NMW (NMW 31110).

***Apusaffinisfurcatus*** Brooke, 1971: 101.

= *Apusnipalensisfurcatus* Brooke, 1971.

Holotype, RMNH.AVES.43220, adult male, skin. Loc.: Telar Tjabang Tampajan, Krawang, Java, [Indonesia], 20.viii.1907. Ex: M. Bartels (5004), 01.vi.1954.

Paratypes, RMNH.AVES.43216–43219, 43221–43235.

There are also four paratypes in the MCZ and four in the NMNH.

***Cypselusleucorrhous*** “Müller” Sclater, 1866: 603.

= *Apusnipalensisfurcatus* Brooke, 1971.

Syntype, RMNH.AVES.187909, male, relaxed mount. Loc.: Semarang, Java, Indonesia, 1828. Leg.: S. Müller.

Syntype, RMNH.AVES.187910, male, relaxed mount, 1828. Loc.: Semarang, Java, Indonesia. Leg.: S. Müller.

Syntype, RMNH.AVES.187911, female, relaxed mount, 10.ii.1828. Loc.: Semarang, Java, Indonesia. Leg.: S. Müller.

Sclater published this manuscript name by Müller in the synonymy of *Cypselussubfurcatus* Blyth, 1849. We have found no subsequent use to validate this name which therefore does not seem to be available.

***Cypselusnipalensis*** Hodgson, 1837a: 780.

= *Apusnipalensisnipalensis* Hodgson, 1837.

Syntype, RMNH.AVES.187897, adult, sex unknown, mounted skin. Loc.: Nepal. Leg.: Hodgson.

Syntype, RMNH.AVES.187898, adult, sex unknown, mounted skin. Loc.: Nepal. Leg.: Hodgson.

***Cypseluspoliourus*** Temminck, 1839: 78.

= *Chaeturabrachyurabrachyura* (Jardine, 1846).

Lectotype, RMNH.AVES.88425, adult male, relaxed mount. Loc.: “Louisiana” [error for Brazil]. Ex: Cabinet. Temminck.

Temminck based his description on pl. 726, fig. 2 in Daubenton and gave no indication of the number of specimens available to him. The listing in Van den Hoek Ostende et al. (1997: 169) as holotype was in error, but constituted a lectotype designation. Temminck’s description pre-dates that of Jardine and has been used by Jardine (1846), Bonaparte (1850a), Sclater (1862: 283) and Hartert (1892b: 485).

***Cypselussenex*** Temminck, 1826: livr. 67, pl. 397.

= *Cypseloidessenex* (Temminck, 1826).

Syntype, RMNH.AVES.88418, adult male, relaxed mount. Loc.: Ypanema [= Ipanema], Brazil, 25.vi.1821. Leg.: J. Natterer. Ex: A. de Saint Hilaire.

The data listed here come from a relatively new label. The old label, which is still attached to the specimen, gives no sex. According to this old label the specimen originates from the collection of Auguste de Saint Hilaire.

According to the original description other syntypes are in the MNHN.

***Cypsiurusbatasiensis* [sic] *bartelsorum*** Brooke, 1972: 221.

= *Cypsiurusbalasiensisbartelsorum* Brooke, 1972.

Holotype, RMNH.AVES.88430, adult female, skin. Loc.: Pangerango, Preanger, Java, [Indonesia], 22.viii.1902. Ex: M. Bartels (1332), 01.vi.1954.

Paratypes, RMNH.AVES.88431–88446.

There are also four paratypes in the NMNH.

***Tachornisparvusbrachypterus*** Reichenow, 1903: 386.

= *Cypsiurusparvusbrachypterus* (Reichenow, 1903).

Syntype, RMNH.AVES.90899, adult male, skin. Loc.: Landana, Loango, Angola, [1876–1877]. Leg.: A. Lucan and L. Petit, 1890.

Syntype, RMNH.AVES.90900, adult, sex unknown, skin. Loc.: Elmina, Goldcoast [= Ghana], W Africa. Leg.: C.J.M. Nagtglas, 1862.

Syntype, RMNH.AVES.90901, adult, sex unknown, skin. Loc.: Elmina, Goldcoast [= Ghana]. Leg.: C.J.M. Nagtglas, 1862.

Syntype, RMNH.AVES.90902, adult, sex unknown, skin. Loc.: Elmina, Goldcoast [= Ghana]. Leg.: C.J.M. Nagtglas, 1862.

Another syntype is in the MfN (ZMB 2002.123).

***Cypselusambrosiacus*** Temminck, 1828: livr. 77, pl. 460, fig. 2.

= *Cypsiurusparvusparvus* (Lichtenstein, 1823).

Lectotype, RMNH.AVES.88447, adult, sex unknown, relaxed mount. Loc.: Cape, South Africa [error for “Nubia”]. Leg.: -.

Temminck based his description on Seba and Brisson and gave no indication of the number of specimens available to him. The listing in Van den Hoek Ostende et al. (1997: 171) as holotype was in error, but constituted a lectotype designation.

The origin of Cap de Bonne-Esperance as given by Temminck and on the label is considered to be erroneous as *C.parvus* does not occur in that part of South Africa. On the backside of the label it was later changed into “Nubia” as is also indicated by Sclater (1866: 602).

***Cypselus* (Chætura) *Nudipes*** Hodgson, 1837a: 779.

= *Hirundapuscaudacutusnudipes* (Hodgson, 1837).

Syntype, RMNH.AVES.88450, adult, sex unknown, relaxed mount. Loc.: Nepal. Leg.: B.H. Hodgson.

Hodgson did not give much or any information on the specimen labels such as collecting date. Hence it is in most cases uncertain whether specimens collected and described by him are indeed types. This is also the type species for *Hirundapus* Hodgson, 1837.

Warren (1966: 210) listed several syntypes in the NHM.

**Chaeturagiganteavar.celebensis** Sclater, 1866: 608.

= *Hirundapuscelebensis* (Sclater, 1866).

Syntype, RMNH.AVES.88453, adult, sex unknown, relaxed mount. Loc.: Menado, Celebes [= Sulawesi], [Indonesia], [i-19.vi] 1841. Leg.: ?E.A. Forsten.

Syntype, RMNH.AVES.88454, adult, sex unknown, relaxed mount. Loc.: Menado, N. Celebes [= Sulawesi], [Indonesia]. Leg.: -.

Forsten visited the Tondano area between 15 April 1840 and 19 June 1841.

***HirundinapusKlaesii*** Büttikofer, 1887: 40.

= *Hirundapuscochinchinensis* (Oustalet, 1878).

Syntype, RMNH.AVES.88448, adult male, skin. Loc.: Loeboe [= Lubuk] Gedang, Padang, W. Sumatra, [Indonesia], 18.ii.1885. Leg.: C. Klaesi.

Syntype, RMNH.AVES.88449, adult female, skin. Loc.: Loeboe [= Lubuk] Gedang, Padang, W. Sumatra, [Indonesia], 18.ii.1885. Leg.: C. Klaesi.

***Chaeturaernsti*** M. Bartels jr, 1931: 54.

= *Hirundapuscochinchinensis* (Oustalet, 1878).

Holotype, RMNH.AVES.63745, adult male, skin. Loc.: Mt. Halimun, W. Java, [Indonesia], 27.xi.1925. Leg.: E. Bartels. Ex: Bartels, 01.vi.1954.

***Cypselusgiganteus*** “van Hasselt” Temminck, 1825: livr. 61, pl. 364.

= *Hirundapusgiganteusgiganteus* (Temminck, 1825).

Holotype, RMNH.AVES.88452, adult, sex unknown, relaxed mount. Loc.: Bantam, Java, [Indonesia, 1820–1823]. Leg.: J.C. van Hasselt.

Holotype by monotypy. Van Hasselt visited Java between 1820 and 1823.

***Acanthyliscoracina*** “Müller” Boie, 1844: 167.

= *Rhaphiduraleucopygialis* (Blyth, 1849).

Syntype, RMNH.AVES.186755, adult female, relaxed mount. Loc.: Sumatra, [Indonesia], 1834. Leg. S. Müller.

Usually referred to as *A.coracinus* Bonaparte, 1850. However, the first valid description was given in a letter of Müller, which was published by Boie (1844) . The description by Müller predates that of Blyth.

Boie (1844) referred only to specimens from Sumatra, excluding RMNH.AVES.88427 from the type series, listed by Van den Hoek Ostende et al. (1997: 169) as syntype, who overlooked RMNH.AVES.186755, the only specimen in the RMNH from Sumatra collected by Müller.

Müller visited West Sumatra from June 1833 until late 1835.

***Cypseluscollaris*** Temminck, 1823: livr. 33, pl. 195.

= *Streptoprocnezonariszonaris* (Shaw, 1796).

Van den Hoek Ostende et al. (1997: 172) listed two syntypes (RMNH.AVES.88455–88456) for *C.collaris* Temminck, 1823. However, this name was based on the description of *Hirundocollaris* Wied, (1820: 73); the RMNH specimens are therefore not part of the type series. See also Schifter (1992) and Schifter et al. (2007: 212) who erroneously dated the first publication by Wied from 1831. According to Greenway (1978: 157) the holotype is in the AMNH (AMNH 5865).

***Cypselusalpinusafricanus*** Temminck, 1815b: 270.

= *Tachymarptismelbaafricanus* (Temminck, 1815).

Syntype, RMNH.AVES.88422, adult, sex unknown, relaxed mount. Loc.: Cap, South Africa. Leg.: -.

***Cypselusalpinus*** Temminck, 1815b: 270.

= *Tachymarptismelbamelba* (Linnaeus, 1758).

Syntype, RMNH.AVES.88423, adult, sex unknown, relaxed mount. Loc.: Europe. Ex: Cabinet Temminck.

Syntype, RMNH.AVES.88424, adult female, relaxed mount. Loc.: Switzerland. Ex: Cabinet Temminck.

***CollocaliaCoquerelii*** Schlegel & Pollen, 1868: 65.

= *Zoonavenagrandidierigrandidieri* (Verreaux, 1867).

Lectotype, RMNH.AVES.88457, adult male, skin. Loc.: North-east Madagascar, 21.x.1865. Leg.: F.P.L. Pollen and D.C. van Dam.

Schlegel and Pollen gave no indication of the number of specimens available to them. The listing in Van den Hoek Ostende et al. (1997: 172) as holotype was in error, but constituted a lectotype designation.

#### ﻿Trochilidae

***Aglæactisaliciæ*** Salvin, 1896: 24.

= *Aglaeactisaliciae* Salvin, 1896.

Syntype, RMNH.AVES.90957 (formerly 4873), adult male, skin. Loc.: Suecha [= Succha], N Peru, 10500 ft., 17.ii.1895. Leg.: O.T. Baron. Ex: Schlüter.

Syntype, RMNH.AVES.90958 (formerly 4680), adult female, skin. Loc.: Suecha [= Succha], N Peru, 10000 ft., 21.ii.1895. Leg.: O.T. Baron. Ex: H. van Zanden.

Syntype, RMNH.AVES.90959 (formerly 4680), adult male, skin. Loc.: Suecha [= Succha], N Peru, 10500 ft., 22.ii.1895. Leg.: O.T. Baron. Ex: H. van Zanden.

Syntype, RMNH.AVES.90960 (formerly 4680), adult male, skin. Loc.: Suecha [= Succha], N Peru, 10500 ft., 16.iii.1895. Leg.: O.T. Baron. Ex: H. van Zanden.

Syntype, RMNH.AVES.90961 (formerly 4680), adult male, skin. Loc.: Suecha [= Succha], N Peru, 10600 ft., 16.iii.1895. Leg.: O.T. Baron. Ex: H. van Zanden.

Syntype, RMNH.AVES.90962 (formerly 4897), adult male, skin. Loc.: Suecha [= Succha], N Peru, 10500 ft., 21.iii.1895. Leg.: O.T. Baron. Ex: H. van Zanden.

Other syntypes are in the NHM (Warren 1966: 11). Salvin mentioned March as the collecting date but see Salvin (1895) and Baron (1897) for confirmation that specimens collected in February were also accessible to him and do belong to the type series.

***Uranomitrawhitelyi*** Boucard, 1893: 8.

= *Amaziliabrevirostrisbrevirostris* (Lesson, 1829).

Syntype, RMNH.AVES.88459 (3131), adult female, skin. Loc.: Annai, Guyana, 14.vii.1890. Leg.: H. Whitely. Ex: W.F.H. Rosenberg, 28.ii.1914.

Other syntypes are in the MNHN (MNHN 1989-425, 1989-426 and 1989-427), in the NMNH (USNM 149526 and 149530) and in the AMNH (AMNH 156266).

***UranomitraDerneddei*** Simon, 1911: 129.

= *Amaziliaviolicepsvioliceps* (Gould, 1859).

Possible paralectotype, RMNH.AVES.148250 (formerly 4809).

Since the date of collecting is missing there is no unequivocal evidence that RMNH.AVES.148250 is part of the type series. Simon (1911) indicated that he had more than one specimen. Later he fixed a (lecto)type by noting “type de Puebla dans la collection E. Simon” (Simon 1921: 325).

***Amaziliacupreicauda*** Salvin & Godman, 1884: 452.

= *Amaziliaviridigastercupreicauda* Salvin & Godman, 1884.

Syntype, RMNH.AVES.88460 (formerly 3131), adult female, skin. Loc.: Roraima, Guyana, 12.xii.1881. Leg.: H. Whitely. Ex: W.F.H. Rosenberg, 28.ii.1914.

Syntype, RMNH.AVES.88461 (formerly 3131), adult male, skin. Loc.: Roraima, Guyana, 01.xi.1883. Leg.: H. Whitely. Ex: W.F.H. Rosenberg, 28.ii.1914.

***Trochilusscutatus*** “Natterer” Temminck, 1824: livr. 50, pl. 299, fig. 3.

= *Augastesscutatusscutatus* (Temminck, 1824).

According to the original description, syntypes are in the MNHN and NMW which is confirmed by Jouanin (1950: 25, no number given) and Schifter et al. (2007: 232–233; NMW 2255).

***TrochilusLangsdorffi*** Temminck, 1821: livr. 11, pl. 66.

= *Discosuralangsdorffilangsdorffi* (Temminck, 1821).

According to the original description the depicted specimen was in the collection of Langsdorff and another in the collection of Leadbeater.

***Eriocnemiscatharina*** Salvin, 1897: xxx.

= *Eriocnemislucianicatharina* Salvin, 1897.

Syntype, RMNH.AVES.90956 (formerly 5175), adult male, skin. Loc.: Leimebamba, E Peru, 10000 ft., 14.vii.1894. Leg.: O.T. Baron. Ex: H.van Zanden, 14.iv.1923.

According to Warren (1966: 53) at least one syntype is in the NHM (NHMUK 1912.7.1.414). Warren erroneously spelled this name as *catherina*.

***Trochilusbilophus*** Temminck, 1820: livr. 3, pl. 18, fig. 3.

***Trochiluscornutus*** Zu Wied-Neuwied, 1821: 190.

= *Heliactinbilophus* (Temminck, 1820).

Holotype for *bilophus*, syntype for *cornutus*, RMNH.AVES.88462, adult male, relaxed mount. Loc.: [Campos Gerais], Brazil, [spring 1816]. Leg.: A.P.M. zu Wied-Neuwied.

Temminck’s plate of October 1820 has priority over Wied’s (Zu Wied-Neuwied 1821) description of *T.cornutus*

-*which name was commonly used in the 20^th^ century based on Simon (1921) who erroneously dated Temminck’s publication as 1824.

Following Dickinson et al. (2022) (see also Introduction of this catalogue under “What is new in this 2023 version”: 14) we have selected the holotype based on the plate since the text that appeared later is irrelevant regarding type status. Any listing of syntypes for *T.bilophus* in any other type catalogue is erroneous. The following specimens have therefore no type status for *T.bilophus* Temminck: AMNH 6835 and 6836 (Greenway 1978: 194) and specimens in the MNHN, although they are syntypes for *T.cornutus* Wied.

***Trochilussquamosus*** Temminck, 1823: livr. 34, pl. 203, fig. 1.

= *Heliomastersquamosus* (Temminck, 1823).

Syntype, RMNH.AVES.88463, immature male, relaxed mount. Loc.: Brazil. Leg.: -.

Schifter et al. (2007: 233–234) listed 18 syntypes collected by Natterer in the NMW (NMW 2352–2369, NMW 19787 and 19788).

***Trochilusmesoleucus*** Temminck, 1824: livr. 53, pl. 317, fig. 1–3.

= *Heliomastersquamosus* (Temminck, 1823).

According to Temminck three syntypes are in the MNHN.

***LeucippusBaeri*** Simon, 1901: 202.

= *Leucippusbaeri* Simon, 1901.

Syntype, RMNH.AVES.258661 (4121), adult, sex unknown, skin. Loc.: Gran Tumbes [= Grau de Tumbes], Peru. Leg.: G.A. Baer. Ex: C. Fritsche, 16.iv.1917.

This is one of two syntypes. The whereabouts of the other type are unknown.

***Trochiluschalybeus*** Temminck, 1821: livr. 11, pl. 66, fig. 2.

= *Lophornischalybeus* (Temminck, 1821).

Vieillot (1823) is frequently given as author. However, Temminck’s plate of June 1821 has priority. The name is often incorrectly spelled as *chalybaeus*.

Following Dickinson et al. (2022) (see also Introduction of this catalogue under “What is new in this 2023 version”: 14) the holotype is the specimen illustrated in 1821 on pl. 66, since the text that appeared on 30 August 1823 is irrelevant regarding type status. According to Temminck, the illustrated specimen is in the MNHN. Any listing of syntypes for this name in Van den Hoek Ostende et al. (1997: 174) or any other type catalogue is erroneous. The following specimens have therefore no type status: RMNH.AVES.88466 and 88467.

***Trochilussqualidus*** Temminck, 1822: livr. 20, pl. 120, fig. 1.

= *Phaethornissqualidus* (Temminck, 1822).

Possible holotype, RMNH.AVES.88465, adult male, relaxed mount. Loc.: Brazil. Leg.: J. Natterer.

Following Dickinson et al. (2022) (see also Introduction of this catalogue under “What is new in this 2023 version”: 14) we have preliminary selected RMNH.AVES.88465 as the holotype based on the plate since the text that appeared on 26 June 1824 is irrelevant regarding type status. According to Temminck, the illustrated specimen was collected by Natterer. As Schifter et al. (2007: 218) listed five “syntypes” in the NMW (NMW 2042–2046) collected by Natterer in Paor, Mattodentro and Ipanema, the illustrated specimen could also be one of these. RMNH.AVES.88464 listed in Van den Hoek Ostende et al. (1997: 173) as “syntype” has no type status.

### ﻿MUSOPHAGIFORMES

#### ﻿Musophagidae

***Chizärhisleucogaster*** Rüppell, 1842: 127.

= *Corythaixoidesleucogaster* (Rüppell, 1842).

Paralectotype, RMNH.AVES.90932.

The lectotype was selected by Steinbacher (1949: 107) and is in the SMF (SMF 12615). According to F. Steinheimer (in litt., 17 November 2003) RMNH.AVES.90932 is also part of the type series.

***Chizaerhiszonurus*** Rüppell, 1835: 9, pl. 4.

= *Criniferzonurus* (Rüppell, 1835).

Paralectotype, RMNH.AVES.90918.

The lectotype was designated by Steinbacher (1949: 107) and is in the SMF (SMF 12614). Paralectotypes are in SMF and NHM (Steinheimer 2005a: 243).

***Musophagapaulina*** Temminck, 1820: livr. 4, pl. 23.

= *Tauracoerythrolophus* (Vieillot, 1819).

The holotype (by monotypy) is in the MNHN.

***Corythaixleucotis*** Rüppell, 1835: 8, pl. 3.

= *Tauracoleucotisleucotis* (Rüppell, 1835).

Paralectotype, RMNH.AVES.90933.

The lectotype was selected by Steinbacher (1949: 107) and is in the SMF (SMF 12617). According to F. Steinheimer (in litt., 17 November 2003), RMNH.AVES.90933 does also belong to the type series.

***MusophagaVerreauxii*** Schlegel, 1854a: 462.

= *Tauracomacrorhynchusverreauxii* (Schlegel, 1854).

Syntype, RMNH.AVES.212778, adult male, mounted skin. Loc.: Gabon. Ex: Verreaux, 1858.

Schlegel (1854a: 462) gave this name to specimens from the Gaboon River to clarify confusion about the identification of *M. Persa* (Linn.) by Verreaux and Verreaux (1851a: 257).

***Corythaixschalowi*** Reichenow, 1891: 148.

**Turacusschalowivar.marungensis** Reichenow, 1902: 52.

= *Tauracoschalowi* (Reichenow, 1891).

Syntype, RMNH.AVES.90765, adult female, skin. Loc.: Marungu, Lufuku River, Central German Africa, [Congo-Kinshasa], 21.vii.1883 Leg.: R.J.C. Böhm. Ex: [MfN], A. Reichenow, 1886.

***Turacusemini*** Reichenow, 1893: 30.

= *Tauracoschuettiiemini* Reichenow, 1893.

Syntype, RMNH.AVES.88140, adult female, skin. Loc.: Vundekakare, Central Africa, 24.xii.1891. Leg.: L.J.C. Emin. Ex: MfN, 1893.

***Turacusfinschi*** Reichenow, 1899: 190.

= *Tauracoschuettiiemini* Reichenow, 1893.

Holotype, RMNH.AVES.88141, adult male, skin. Loc.: Ndoruma, Niamniamland, Central Africa, [South Sudan], 01.ix.1883. Leg.: Bohndorff. Ex: W. Schlüter, 1884.

Holotype by monotypy.

### ﻿OTIDIFORMES

#### ﻿Otididae

***Otiscærulescens*** Vieillot, 1821: 334.

= *Eupodotiscaerulescens* (Vieillot, 1821).

Holotype, RMNH.AVES.223279, adult male, mounted skin. Loc.: South Africa. Leg.: F. Levaillant.

Vieillot based his name on the description in Levaillant’s *Voyage* (1790: 226). Levaillant wrote that his dogs caught a bustard, later listed by Temminck (1807: 161) under no. 225, *Otis Coerulescens* Pays des Caffres, which is a nomen nudum.

***Otisscolopacea*** Temminck, 1836: livr. 97, pl. 576.

= *Eupodotisvigorsiivigorsii* (Smith, 1831).

Syntype, RMNH.AVES.87486, adult male, mounted skin. Loc.: Cap, South Africa [1827–1832]. Leg.: J. Smuts.

Syntype, RMNH.AVES.87487, adult female, mounted skin. Loc.: Cap, South Africa. Leg.: H.B. van Horstok.

Syntype, RMNH.AVES.87488, adult female, mounted skin. Loc.: Cap, South Africa. Leg.: H.B. van Horstok.

The year of publication of livraison 97, usually given as 1835 following Sherborn (1898: 488), has been corrected to 1836 by Mees (1994: 51) and confirmed by Dickinson (2001: 45). According to the original description a female is in the MNHN. It is, however, not listed in Voisin and Voisin (2015).

***Otismelanogaster*** Rüppell, 1835: 16, pl. 7.

= *Lissotismelanogastermelanogaster* (Rüppell, 1835).

According to the correspondence in the RMNH archives (letter by Rüppell to Temminck dated 15 October 1837) the RMNH must have received one (type) specimen of this newly described taxon. Since there is no specimen in the collection which fits as type it must be considered lost. For more information see Steinheimer (2005a: 239).

***OtisLudwigii***Rüppell 1837b: 223.

= *Neotisludwigii* (Rüppell 1837).

In his description of *Otisscolopacea*, Temminck (1836) referred to a specimen collected by Ecklon which he identified as *O. Denhamii* (Pl. Col. 97). Rüppell (1837b) included this taxon in his description of *O.ludwigii*, thus adding the Ecklon specimen to the type series of *ludwigii*. However, this specimen is no longer in the RMNH.

### ﻿CUCULIFORMES

#### ﻿Cuculidae

***Cacomantisaeruginosus*** Salvadori, 1878b: 456.

= *Cacomantisaeruginosusaeruginosus* Salvadori, 1878.

Syntype, RMNH.AVES.214221, adult male, relaxed mount. Loc.: Ambon, [Indonesia], 7.i.1867. Leg.: D.S. Hoedt.

Syntype, RMNH.AVES.214223, adult female, relaxed mount. Loc.: Ambon, [Indonesia], 6.iii.1867. Leg.: D.S. Hoedt.

Syntype, RMNH.AVES.214225, immature female, relaxed mount. Loc.: Ambon, [Indonesia], 1863. Leg.: D.S. Hoedt.

Syntype, RMNH.AVES.214231, adult, sex unknown, skin. Loc.: Wahaai, Ceram, [Indonesia]. Leg.: Moens, 1862.

The remaining syntypes are in the MSNG.

***Cacomantisarfakianus*** Salvadori, 1889: 177.

= *Cacomantiscastaneiventris* (Gould, 1867).

Syntype, RMNH.AVES.88142, adult male, relaxed mount. Loc.: Kalwal, Salawatti, [Indonesia], 06.iii.1865. Leg.: H.A. Bernstein.

Syntype, RMNH.AVES.88143, adult male, relaxed mount. Loc.: Kalwal, Salawatti, [Indonesia], 28.iii.1865. Leg.: H.A. Bernstein.

Syntype, RMNH.AVES.88144, adult male, relaxed mount. Loc.: Waigama, Misool, [Indonesia], 18.vii.1867. Leg.: D.S. Hoedt.

Syntype, RMNH.AVES.88145, adult male, relaxed mount. Loc.: Aru Isl., [Indonesia]. Leg.: C.B.H. von Rosenberg.

Syntype, RMNH.AVES.88146, adult male, relaxed mount. Loc.: Aru Isl., [Indonesia]. Leg.: C.B.H. von Rosenberg.

***Cuculusleucolophus*** Müller, 1840: 22.

= *Cacomantisleucolophus* (Müller, 1840).

Syntype, RMNH.AVES.88157, adult male, relaxed mount. Loc.: Lobo Bay [Papua, Indonesia], [vii–viii] 1828. Leg.: S. Müller.

Syntype, RMNH.AVES.88158, adult male, relaxed mount. Loc.: Lobo Bay [Papua, Indonesia], [vii–viii] 1828. Leg.: S. Müller.

Syntype, RMNH.AVES.88159, adult female, relaxed mount. Loc.: Lobo Bay [Papua, Indonesia], [vii–viii] 1828. Leg.: S. Müller.

***Cuculuslanceolatus*** Müller, 1843: 178.

= *Cacomantismerulinuslanceolatus* (Müller, 1843).

Syntype, RMNH.AVES.88147, adult male, relaxed mount. Loc.: Java, [Indonesia], 1826. Leg.: H. Boie.

Syntype, RMNH.AVES.88148, adult male, relaxed mount. Loc.: Java, [Indonesia], 1826. Leg.: H. Boie.

Syntype, RMNH.AVES.88149, adult female, relaxed mount. Loc.: Java, [Indonesia], 1826. Leg.: H. Boie.

Syntype, RMNH.AVES.88150, immature, relaxed mount. Loc.: Java, [Indonesia], 1826. Leg.: H. Boie.

Syntype, RMNH.AVES.88151, immature, relaxed mount. Loc.: Java, [Indonesia], 1826. Leg.: H. Boie.

Syntype, RMNH.AVES.88152, immature, relaxed mount. Loc.: Java, [Indonesia], 1826. Leg.: H. Boie.

***Cuculuspoliogaster*** Müller, 1845: 236.

= *Cacomantispallidus* (Latham, 1801).

Lectotype, RMNH.AVES.88204, immature, relaxed mount. Loc.: Ternate, [Indonesia], [19.vi–ix] 1841. Leg.: E.A. Forsten.

Müller gave no indication of the number of specimens. Van den Hoek Ostende et al. (1997: 143) erroneously listed RMNH.AVES.88204 as holotype and overlooked the lectotype designation by Schlegel (1864b: 22).

Forsten was forced to stay on Ternate due to illness between 19 June 1841 and mid-September 1841.

***Cuculussepulcralis*** Müller, 1843: 177.

= *Cacomantissepulcralissepulcralis* (Müller, 1843).

Syntype, RMNH.AVES.88154, adult male, relaxed mount. Loc.: West Java, [Indonesia], 1827. Leg.: S. Müller.

Syntype, RMNH.AVES.88155, immature female, relaxed mount. Loc.: West Java, [Indonesia], 1827. Leg.: S. Müller.

Syntype, RMNH.AVES.213998, adult male, relaxed mount. Loc.: Java, [Indonesia], 1827. Leg.: S. Müller.

Syntype, RMNH.AVES.214000, adult female, relaxed mount. Loc.: Djassinga [= Jasinga?], Java, [Indonesia], xi.1827. Leg.: S. Müller.

Syntype, RMNH.AVES.214008, adult male, relaxed mount. Loc.: Java, [Indonesia], 1827. Leg.: S. Müller.

Müller described this species as originating from Java and Sumatra, and Schlegel (1864b) listed a specimen collected by Müller in Sumatra. This specimen is no longer in the RMNH.

***Cuculusfasciolatus*** Müller, 1843: 177.

***Penthoceryxsonneratiischlegeli*** Junge, 1948: 322.

= *Cacomantissonneratiifasciolatus* (Müller, 1843).

Syntype for *fasciolatus*, holotype for *schlegeli*, RMNH.AVES.88244, immature male, relaxed mount. Loc.: Padang, Sumatra, [Indonesia], [1833–1835]. Leg.: S. Müller, 1834.

Syntype for *fasciolatus*, RMNH.AVES.88242, adult male, relaxed mount. Loc.: West Java, [Indonesia]. Leg.: S. Müller, 1826 (= *Cacomantissonneratiimusicus* (Ljungh, 1804)).

Syntype for *fasciolatus*, RMNH.AVES.88243, adult female, relaxed mount. Loc.: West Java, [Indonesia]. Leg.: S. Müller, 1826 (= *Cacomantissonneratiimusicus* (Ljungh, 1804)).

The type series also includes specimens from Java, which are *Cacomantissonneratiimusicus* (Ljungh, 1804). Robinson and Kloss (1923: 336, 359) restricted the type locality to Sumatra. However, Junge (1948: 322) pointed out that by only listing the Javan specimens as types for *C.fasciolatus*, Schlegel (1864b: 24) already restricted the type locality to Java, making *C.fasciolatus* a synonym of *musicus*. According to Junge this made it necessary to propose a new name for the Sumatran form which he named *schlegeli*. Following the IOC use of the name *C.fasciolatus* Müller to birds from Sumatra, Borneo, and Palawan, RMNH.AVES.88244 needs to be selected as lectotype to fix *C.fasciolatus* Müller to Sumatra.

Müller visited Sumatra between 1833 and 1835.

***Cuculustymbonomus*** Müller, 1843: 177.

= *Cacomantisvariolosustymbonomus* (Müller, 1843).

Holotype, RMNH.AVES.88156, adult male, relaxed mount. Loc.: Timor, [Indonesia], 1829. Leg.: S. Müller.

Holotype by monotypy: Müller collected a single male.

***Cuculusdumetorum*** Gould, 1845b: text to pl. 87.

= *Cacomantisvariolosusvariolosus* (Vigors & Horsfield, 1827).

Possible paralectotype, RMNH.AVES.214284.

Stone (1913: 152) designated the lectotype in the ANSP (ANSP 20028). Fisher and Calaby (2009: 130) listed RMNH.AVES.214284 as a probable type.

***Calobatesradiceus*** Temminck, 1832: livr. 91, pl. 538.

= *Carpococcyxradiceus* (Temminck, 1832).

Holotype, RMNH.AVES.88160, adult female, mounted skin. Loc.: Pontianak, West Borneo, [Indonesia]. Leg.: P.M. Diard.

Holotype by monotypy. Temminck wrote that this species is known from a single female.

The name *C.radiceus* is considered a lapsus for *radiatus* (Collar and Long 1996).

***Centropusmedius*** “Müller” Bonaparte, 1850a: 108.

= *Centropusbengalensismedius* Bonaparte, 1850.

Syntype, RMNH.AVES.88161, adult male, mounted skin. Loc.: Amboina [= Ambon], [Indonesia], ix.1828. Leg.: S. Müller.

Syntype, RMNH.AVES.88162, adult female, mounted skin. Loc.: Amboina [= Ambon], [Indonesia], ix.1828. Leg.: S. Müller.

Syntype, RMNH.AVES.88163, adult female, mounted skin. Loc.: Amboina [= Ambon], [Indonesia], ix.1828. Leg.: S. Müller.

Syntype, RMNH.AVES.88164, adult female, mounted skin. Loc.: Amboina [= Ambon], [Indonesia], 1842. Leg.: E.A. Forsten.

Syntype, RMNH.AVES.217824, adult female, mounted skin. Loc.: Tjikao, Java, [Indonesia], ii.1827. Leg.: H. Boie (= *Centropusbengalensisjavanensis* (Dumont, 1818)).

Syntype, RMNH.AVES.217832, adult, sex unknown, mounted skin. Loc.: Java, [Indonesia], [1818–1826]. Leg.: Blume, 1828. (= *Centropusbengalensisjavanensis* (Dumont, 1818)).

Syntype, RMNH.AVES.217834, sex unknown, mounted skin. Loc.: Java, [Indonesia], [xii.1820–ix.1821]. Leg.: Kuhl and van Hasselt. (= *Centropusbengalensisjavanensis* (Dumont, 1818)).

Syntype, RMNH.AVES.217836, sex unknown, mounted skin. Loc.: Java, [Indonesia], [xii.1820–ix.1821]. Leg.: Kuhl and van Hasselt. (= *Centropusbengalensisjavanensis* (Dumont, 1818)).

Syntype, RMNH.AVES.217842, immature, sex unknown, mounted skin. Loc.: Tjikao, Java, [Indonesia], 19.iii.1827. Leg.: H. Boie (= *Centropusbengalensisjavanensis* (Dumont, 1818)).

According to Bonaparte this species originated from Ambon and Java, a fact overlooked by Van den Hoek Ostende et al. (1997: 139). The specimens from Java belong to the subspecies *C.b.javanensis*. Following the IOC use of the name *medius* Bonaparte for birds from the Moluccas, a lectotype needs to be selected to fix this name to birds from the Moluccas.

Kuhl and Van Hasselt visited Java between December 1820 and September 1821. Blume stayed in Java from 1818 until 1826.

***Centropusbengalensisphilippinensis*** Mees, 1971a: 190.

= *Centropusbengalensisphilippinensis* Mees, 1971.

Holotype, RMNH.AVES.88165, adult female, skin. Loc.: Calapan, Mindoro, Philippines, 28.v.1888. Leg.: E.L. Moseley, Steere Expedition, 1890.

Paratype, RMNH.AVES.88166.

There are also eight paratypes in the UZMC.

The year of collecting given on the original labels is 1887, but should be 1888 (G.F. Mees, in litt.).

***CentropusBernsteini*** Schlegel, 1866c: 251.

= *Centropusbernsteini* Schlegel, 1866.

Holotype, RMNH.AVES.88167, adult female, skin. Loc.: Sailolo, Salawati, [Indonesia], 28.ii.1865. Leg.: H.A. Bernstein.

Holotype by monotypy. Schlegel wrote he had only seen a female. Salawati is probably incorrect (Hartert 1932).

***Centropuscelebensis*** Quoy & Gaimard, 1832: 230.

= *Centropuscelebensiscelebensis* Quoy & Gaimard, 1832.

Paralectotypes, RMNH.AVES.88168 and 88169.

The lectotype was selected by Voisin and Voisin (1999: 390) and is in the MNHN (MNHN C.G.1997–1193). For the publication dates of the ‘Voyage de découvertes de l’Astrolabe’, see Mlíkovský (2012a).

***Centropusgoliath*** Bonaparte, 1850a: 108.

= *Centropusgoliath* Bonaparte, 1850.

Syntype, RMNH.AVES.88173, adult male, mounted skin. Loc.: Gilolo, [Halmahera], [Indonesia], [19.vi.-ix.1841]. Leg.: E.A. Forsten.

Syntype, RMNH.AVES.88174, adult female, mounted skin. Loc.: Gilolo, [Halmahera], [Indonesia], [19.vi.-ix.1841]. Leg.: E.A. Forsten.

Syntype, RMNH.AVES.95985, adult female, mounted skin. Loc.: Dodingo, Halmahera, [Indonesia], [19.vi.-ix.1841]. Leg.: E.A. Forsten.

Syntype, RMNH.AVES.95986, adult, sex unknown, mounted skin. Loc.: Dodingo, Halmahera, [Indonesia], [19.vi.-ix.1841]. Leg.: E.A. Forsten.

Syntype, RMNH.AVES.217263, adult female, mounted skin. Loc.: Dodingo, Halmahera, [Indonesia], [19.vi.-ix.1841]. Leg.: E.A. Forsten.

Syntype, RMNH.AVES.217268, adult male, mounted skin. Loc.: Halmahera, [Indonesia], [19.vi.-ix.1841]. Leg.: E.A. Forsten.

Syntype, RMNH.AVES.217269, adult male, mounted skin. Loc.: Gilolo, [Halmahera], [Indonesia], [19.vi.-ix.1841]. Leg.: E.A. Forsten.

Van den Hoek Ostende et al. (1997: 140) only listed RMNH.AVES.88173 and 88174 as types. However, there are more specimens from Forsten collected on Halmahera which qualify as types.

During a forced stay on Ternate from 19 June 1841 until mid-September 1841 due to illness, Forsten sent his hunters to Halmahera to collect skins.

***Centropusfrancisci*** Bonaparte, 1850a: 107.

= *Centropusleucogasterleucogaster* (Leach, 1814).

Syntype, RMNH.AVES.88175, adult male, mounted skin. Loc.: Dabocrom, Côte d’Or [= Ghana]. Leg.: H.S. Pel.

Syntype, RMNH.AVES.88176, adult female, mounted skin. Loc.: Dabocrom, Côte d’Or [= Ghana]. Leg.: H.S. Pel.

***Centropusmonachus*** Rüppell, 1837a: 57, pl. 21, fig. 2.

= *Centropusmonachusmonachus* Rüppell, 1837.

Paralectotypes, RMNH.AVES.90915–90917.

The lectotype was designated by Steinbacher (1949: 107) and is in the SMF (SMF 12612). Paralectotypes are in the SMF and NHM (Steinheimer 2005a: 243).

***PolophilusGigas*** Stephens, 1815: 45.

***Corydonixgiganteus*** Vieillot, 1819c: 295.

= *Centropusphasianinusphasianinus* (Latham, 1801).

Holotype, RMNH.AVES.88177, immature, relaxed mount. Loc.: Australia. Ex: Cabinet Temminck.

Both Stephens and Vieillot based their description on a plate by Levaillant. Levaillant (1806–1807: 87) based his *Coucal Géant* on a specimen in the cabinet of Temminck and also referred to a specimen from the Baudin Expedition in the MNHN. According to Jansen (2018: 288), RMNH.AVES.88177 was collected during the Baudin Expedition. Voisin and Voisin (1999) did not list a type in the MNHN.

***Centropusepomidis*** Bonaparte, 1850a: 107.

= *Centropussenegalensissenegalensis* (Linnaeus, 1766).

Syntype, RMNH.AVES.88170, immature male, mounted skin. Loc.: Sacconde, Côte d’Or [= Sekondi-Takoradi, Ghana], 11.x.1846. Leg.: H.S. Pel.

Syntype, RMNH.AVES.88171, immature male, mounted skin. Loc.: Côte d’Or [= Ghana]. Leg.: H.S. Pel.

Syntype, RMNH.AVES.88172, adult female (dark morph), mounted skin. Loc.: Rio Boutry, Côte d’Or [= Ghana]. Leg.: H.S. Pel.

Syntype, RMNH.AVES.218216, adult female, mounted skin. Loc.: Sacconde, Côte d’Or [= Sekondi-Takoradi, Ghana], 20.iii.1841. Leg.: H.S. Pel.

***Centropuskangeangensis*** Vorderman, 1893: 190.

= *Centropussinensiskangeangensis* Vorderman, 1893.

Syntype, RMNH.AVES.88178, adult, sex unknown, skin. Loc.: Ardjaso [= Arjasa], Kangean, [Indonesia], v.1892. Leg.: A.G. Vorderman, 1896.

Vorderman mentioned that he collected two specimens of this taxon together with three specimens of *C.sinensissinensis* Stephens, 1815. The RMNH now possesses three specimens of *C.sinensiskangeangensis* collected by Vorderman. One of these is the type given here, the other two were originally identified as *C. s. sinensis.* The whereabouts of the other syntype is unknown.

***Centropusmadagascariensis*** Schlegel, 1864b: 65.

= *Centropustouloutoulou* (P.L.S. Müller, 1776).

Holotype, RMNH.AVES.90983, immature male, mounted skin. Loc.: Madagascar. Ex: [Maison] Verreaux 1863.

Holotype by monotypy. Schlegel listed only RMNH.AVES.90983 with his description of *C.madagascariensis*.

***Centropusmolkenboeri*** Bonaparte, 1850a: 108.

= *Centropusviridisviridis* (Scopoli, 1786).

Lectotype, RMNH.AVES.88179, adult male, mounted skin. Loc.: Philippines. Leg.: -.

Bonaparte gave no indication of the number of specimens available to him. Schlegel (1864b: 65) designated the lectotype.

***CuculusKlaas*** Stephens, 1815: 128.

***Cuculusklaasii*** Vieillot, 1817b: 230.

= *Chrysococcyxklaas* (Stephens, 1815).

Lectotype, RMNH.AVES.88198, adult male, relaxed mount. Loc.: South Africa. Ex: Cabinet Temminck.

Both Stephens and Vieillot based their description on Levaillant’s ‘Le Coucou de Klaas’ (1806–1807: 53, pl. 212). Levaillant based his description on a specimen in his own collection and a specimen in the MNHN from Senegal, not listed in Voisin and Voisin (1999). As the description by Levaillant is based on two specimens, Van den Hoek Ostende et al. (1997: 143) were in error by listing RMNH.AVES.88198 as holotype for both names. However, this constituted a lectotype designation.

***Cuculuschalcites*** “Illiger” Temminck, 1821: livr. 17, pl. 102, fig. 2.

= *Chrysococcyxlucidusplagosus* (Latham, 1801).

Following Dickinson et al. (2022) (see also Introduction of this catalogue under “What is new in this 2023 version”: 14) the specimen illustrated as fig. 2 on pl. 102 is the holotype. Temminck indicated that it was in the RMNH where no specimen that matches the plate could be found. Other specimens listed by Temminck which could include the holotype are in the MNHN and MfN, although Voisin and Voisin (1999) did not list any types in Paris. In 1823, a bird was sent to the NMW (NMW 1823.LXXXVIII.78) and one was received in exchange by the MfN in July 1818.

***Cuculusneglectus*** Schlegel, 1864b: 35.

= *Chrysococcyxminutillusaheneus* (Junge, 1938).

Holotype, RMNH.AVES.88180, adult, sex unknown, relaxed mount. Loc.: South Borneo, [Indonesia], [28.vii–17.xii.] 1836. Leg.: S. Müller.

Holotype by monotypy. Müller visited Borneo between 28 July 1836 and 17 December 1836.

***Chalcitesmalayanusaheneus*** Junge, 1938: 238.

= *Chrysococcyxminutillusaheneus* (Junge, 1938).

Holotype, RMNH.AVES.88187, adult male, relaxed mount. Loc.: Banjermassing, S. Borneo, [Indonesia], viii.1844. Leg.: C.A.L.M. Schwaner.

Paratypes, RMNH.AVES.88187–88190.

***Chalcitesmalayanusalbifrons*** Junge, 1938: 237.

= *Chrysococcyxminutillusalbifrons* (Junge, 1938).

Holotype, RMNH.AVES.88191 (1371), adult male, skin. Loc.: Batavia [= Jakarta], Java, [Indonesia], 14.x.1908. Leg.: E.R. Jacobson.

Paratypes, RMNH.AVES.88192–88196.

***Lamprococcyxcrassirostris*** Salvadori, 1878b: 456.

= *Chrysococcyxminutilluscrassirostris* (Salvadori, 1878).

Syntype, RMNH.AVES.88181, adult male, relaxed mount. Loc.: Amboine [= Ambon], [Indonesia], [29.iii–20.iv] 1828. Leg.: S. Müller.

Syntype, RMNH.AVES.88182, adult female, relaxed mount. Loc.: Sorong, [Papua, Indonesia], 1864. Leg.: H.A. Bernstein.

Syntype, RMNH.AVES.88183, adult female, relaxed mount. Loc.: Sorong, [Papua, Indonesia], 1865. Leg.: H.A. Bernstein.

Syntype, RMNH.AVES.88184, immature, sex unknown, relaxed mount. Loc.: Ternate, [Indonesia], 1872. Leg.: C.B.H. von Rosenberg.

Syntype, RMNH.AVES.88185, adult male, relaxed mount. Loc.: Halmahera, [Indonesia], 17.vi.1876. Leg.: C.B.H. von Rosenberg.

Syntype, RMNH.AVES.88186, immature female, relaxed mount. Loc.: Little Key, [Indonesia]. Leg.: C.B.H. von Rosenberg.

Syntype, RMNH.AVES.214807, immature female, relaxed mount. Loc.: Goram [= Gorong], [Indonesia]. Leg. C.B.H. von Rosenberg.

Salvadori (1878b: 460) inspected seven specimens in the RMNH. Van den Hoek Ostende et al. (1997: 141) overlooked RMNH.AVES.214807. The remaining syntypes are in the MSNG.

Müller visited Ambon between 29 March and 20 April 1828.

***Chalcitesmalayanusjungei*** Stresemann, 1938: 148.

= *Chrysococcyxminutillusjungei* (Stresemann, 1938).

Paratype, RMNH.AVES.8755.

The holotype is in the AMNH.

***Chalcococcyxinnominatus*** Finsch, 1900: 94.

= *Chrysococcyxminutillusrufomerus* Hartert, 1900.

Holotype, RMNH.AVES.88197, adult male, skin. Loc.: Kisar Island, [Indonesia], 28.vi.1866. Leg.: D.S. Hoedt, 1896.

Holotype by monotypy. Finsch described *C.innominatus* based on a single male.

***Cuculusedolio*** Temminck, 1807: 60.

= *Clamatorjacobinus* subsp.

Possible syntype, RMNH.AVES.212934, adult (pale morph), sex unknown, mounted skin. Loc.: Cap, [South Africa]. Leg.: “Horstok”. (= *Clamatorjacobinusserratus* (Sparrman, 1786).

Temminck based his description on a male and female in his own collection.

Temminck referred to Levaillant’s (1806: vol. 5, pl. 207 and 208) ‘Le Coucou Edolio d’Afrique’. Plate 208 shows, unmistakably, a pale morph *Clamatorjacobinus*, not *C.levaillantii* (Swainson, 1829) as stated by Rookmaaker (1989: 204). Temminck also included *Cuculus Melanoleucos* (Gmelin, 1788: 416) from the Coromandel, viz. India, in the synonymy, which refers to *C.j.jacobinus* (Boddaert, 1783).

Van Horstok is not mentioned under the stand in Temminck’s handwriting but was added later to the label and should be considered an error as he was not in South Africa as early as 1807.

***Coccyzuslansbergi*** Bonaparte, 1850a: 112.

= *Coccyzuslansbergi* Bonaparte, 1850.

Syntype, RMNH.AVES.88200, adult male, relaxed mount. Loc.: Santa Fé de Bogota, Nouvelle Grenade [= Colombia]. Leg.: R.F. van Lansberge.

Syntype, RMNH.AVES.88201, adult female, relaxed mount. Loc.: Santa Fé de Bogota, Nouvelle Grenade [= Colombia]. Leg.: R.F. van Lansberge.

***Coccycus Delalandei*** Temminck, 1827: livr. 74, pl. 440.

= *Couadelalandei* (Temminck, 1827).

The holotype is in the MNHN (Voisin and Voisin 1999: 388; MNHN 1997–1190).

***Cuculuslepidus*** Müller, 1845: 236.

= *Cuculuslepidus* Müller, 1845.

Holotype, RMNH.AVES.88205, immature female, relaxed mount. Loc.: [Lelogama], Timor, [Indonesia], [7–9.x] 1829. Leg.: S. Müller.

Holotype by monotypy. Müller collected a single specimen during his stay in the area of Lelogama from 7 to 9 October 1829 (Müller 1843: 233). Müller identified it as an adult female but it is immature.

***Cuculustenuirostris*** Müller, 1845: 235.

= *Cuculuslepidus* Müller, 1845.

Syntype, RMNH.AVES.88207, adult female, relaxed mount. Loc.: Java, [Indonesia]. Leg.: S. Müller, 1827.

Syntype, RMNH.AVES.88208, adult female, relaxed mount. Loc.: Java, [Indonesia]. Leg.: S. Müller, 1827.

Syntype, RMNH.AVES.88209, adult female, relaxed mount. Loc.: Java, [Indonesia]. Leg.: S. Müller, 1827.

Syntype, RMNH.AVES.88210, adult male, relaxed mount. Loc.: Sumatra, [Indonesia], [vi.1833–1835]. Leg.: S. Müller.

Birds arrived in the NMW in 1821 (no. 1821.LXXIII.50), 1823 (no. 1823.LXXXVIII.77) and April 1830 (no. 1830.VII.36 and 37). Two arrived in the MfN in December 1823. Müller visited West Sumatra from June 1833 until late 1835.

***Cuculuscanoroïdes*** Müller, 1845: 235.

= *Cuculuslepidus* Müller, 1845.

Lectotype, RMNH.AVES.88213, immature, relaxed mount. Loc.: Dusum [= Barito River], Borneo, [Indonesia], viii.1836. Leg.: S. Müller.

Paralectotypes, RMNH.AVES.88199, 88214–88216, 90951–90953.

Junge (1956b: 556) selected the lectotype. He erroneously gave “G[unung.

= Mt.] Doesoen, Poeloe Maja” as collecting locality. This specimen was collected, however, on the Dusun [= Barito] River (Müller 1847a).

***Cuculusconcretus*** Müller, 1845: 236.

= *Cuculusmicropterusconcretus* Müller, 1845.

Syntype, RMNH.AVES.88202, adult male, relaxed mount. Loc.: South Borneo, [Indonesia], 1836. Leg.: S. Müller.

Syntype, RMNH.AVES.88203, adult male, relaxed mount. Loc.: South Borneo, [Indonesia], 1836. Leg.: S. Müller.

***Eudynamismelanorhynchus*** Müller, 1843: 176.

= *Eudynamysmelanorhynchus* Müller, 1843.

Syntype, RMNH.AVES.88218, adult male, relaxed mount. Loc.: Kema, Celebes [= Sulawesi], [Indonesia], 1840. Leg.: E.A. Forsten.

Syntype, RMNH.AVES.88219, adult female, relaxed mount. Loc.: Gorontalo, Celebes [= Sulawesi], [Indonesia], 1840. Leg.: E.A. Forsten.

Syntype, RMNH.AVES.88220, immature male, relaxed mount. Loc.: Tondano, Celebes [= Sulawesi], [Indonesia], 1840. Leg.: E.A. Forsten.

Syntype, RMNH.AVES.88221, adult female, relaxed mount. Loc.: Gorontalo, Celebes [= Sulawesi], [Indonesia], 1840. Leg.: E.A. Forsten.

Forsten stayed in the Tondano area between 22 March 1840 and 19 June 1841.

***Eudynamisransomi*** Bonaparte, 1850a: 101.

**Cuculuspunctatusvar.ceramensis** “Forsten” Bonaparte, 1850a: 101.

= *Eudynamysorientalisorientalis* (Linnaeus, 1766).

Syntype, RMNH.AVES.90971, adult male, relaxed mount. Loc.: Ceram, [Indonesia], 1842. Leg.: E.A. Forsten.

Syntype, RMNH.AVES.90972, adult female, relaxed mount. Loc.: Ceram, [Indonesia], 1842. Leg.: E.A. Forsten.

Bonaparte published Forsten’s manuscript name *C.p.ceramensis* in the synonymy of *E.ransomi*.

Forsten visited Seram late 1842.

***Eudynamispicatus*** Müller, 1843: 176.

= *Eudynamysorientalispicatus* Müller, 1843.

Holotype, RMNH.AVES.88224, immature male, relaxed mount. Loc.: Amboina [= Ambon], [Indonesia], ix.1828. Leg.: S. Müller.

Holotype by monotypy. Müller based his description on a single male in moult.

***Eudynamisminima*** van Oort, 1911b: 54.

= *Eudynamysorientalisrufiventer* (Lesson, 1830).

Syntype, RMNH.AVES.88222, adult male, skin. Loc.: Sabang, [Papua, Indonesia], 03.vii.1907. Leg.: Nieuw Guinea Expeditie 1907.

Syntype, RMNH.AVES.88223, adult male, skin. Loc.: Bivak Island, [Papua, Indonesia], 09.i.1910. Leg.: Dutch New Guinea Expedition 1909.

***Eudynamisscolopaceasimalurensis*** Junge, 1936: 43.

= *Eudynamysscolopaceusmalayanus* Cabanis & Heine, 1863.

Holotype, RMNH.AVES.88225 (6651), adult female, skin. Loc.: Islet in Sibigo Bay, Simalur [= Simeulue], [Indonesia], 28.viii.1913. Leg.: E.R. Jacobson and W.C. van Heurn.

Paratypes, RMNH.AVES.88226–88239.

***Cuculusvagans*** Müller, 1845: 233.

= *Hierococcyxvagans* (Müller, 1845).

Holotype, RMNH.AVES.88217, immature, relaxed mount. Loc.: Java, [Indonesia], [xii.1820–ix.1821]. Leg.: H. Kuhl and J.C. van Hasselt.

Müller described this specimen as an adult male.

Kuhl and Van Hasselt visited Java between December 1820 and September 1821.

***CoccyzusGeoffroyi*** Temminck, 1820: livr. 2, pl. 7.

= *Neomorphusgeoffroyigeoffroyi* (Temminck, 1820).

Following Dickinson et al. (2022) (see also Introduction of this catalogue under “What is new in this 2023 version”: 14) the specimen on pl. 7 is the holotype since the text that was published on or before 25 June 1823, three years after the plate was published in September 1820, is irrelevant regarding type status. According to Temminck the depicted specimen was in the collection of Meiffren-Laugier de Chartrouse and may now be in the MNHN. Any listing of syntypes for this name in Van den Hoek Ostende et al. (1997: 145) or any other type catalogue is erroneous. RMNH.AVES.88240, listed in Van den Hoek Ostende et al. (1997: 145), has therefore no type status.

***CuculusAudeberti*** Schlegel, 1879b: 99.

= *Pachycoccyxaudebertiaudeberti* (Schlegel, 1879).

Holotype, RMNH.AVES.88241, adult female, skin. Loc.: Ambodikila, near Mananara, N.E. Madagascar, 10.vi.1878. Leg.: J.P. Audebert.

Holotype by monotypy. Schlegel referred to RMNH.AVES.88241 as the typical specimen.

***Cuculuscurvirostris*** Shaw, 1810: text to pl. 905.

***Phænicophæustricolor*** Stephens, 1815: 61, pl. 14.

***Phænicophaus* [sic] *viridis*** Vieillot, 1817d: 462.

= *Phaenicophaeuscurvirostriscurvirostris* (Shaw, 1810).

The above names are based on “Le malkoha rouverdin” by Levaillant (1806–1807: 92, pl. 225). Levaillant based his description on a specimen in Temminck’s Cabinet (see Temminck 1807: 59, 209), assuming the bird originated from Africa which it did not. No specimen in the RMNH fits as type.

***Phœnicophaeusviridirufus*** “Levaillant” Müller, 1845: 234.

***Phaenicophaeuserythrognathus*** Bonaparte, 1850a: 98.

= *Phaenicophaeuscurvirostris* subsp.

Syntype (*viridirufus* and *erythrognathus*), RMNH.AVES.88266, adult male, mounted skin. Loc.: Sumatra, [Indonesia], [1835–1838]. Leg.: L. Hörner (= *Phaenicophaeuscurvirostrissingularis* (Parrot, 1907)).

Syntype (*viridirufus* and *erythrognathus*), RMNH.AVES.88267, adult female, mounted skin. Loc.: Sumatra, [Indonesia], [1835–1838]. Leg.: L. Hörner (= *Phaenicophaeuscurvirostrissingularis* (Parrot, 1907)).

Syntype (*viridirufus* and *erythrognathus*), RMNH.AVES.216617, adult female, mounted skin. Loc.: Inderapura, Sumatra, [Indonesia], vii.1835. Leg.: S. Müller (= *Phaenicophaeuscurvirostrissingularis* (Parrot, 1907)).

Syntype (*viridirufus* and *erythrognathus*), RMNH.AVES.216618, adult female, mounted skin. Loc.: Bungus, Sumatra, [Indonesia], iii.1834. Leg.: S. Müller (= *Phaenicophaeuscurvirostrissingularis* (Parrot, 1907)).

Syntype (*viridirufus*), RMNH.AVES.90973, adult male, relaxed mount. Loc.: Java, [Indonesia], [vi–xii] 1826. Leg.: H. Boie (= *Phaenicophaeuscurvirostriscurvirostris* (Shaw, 1810)).

Syntype (*viridirufus*), RMNH.AVES.90974, adult female, relaxed mount. Loc.: Java, [Indonesia], [vi–xii] 1826. Leg.: H. Boie (= *Phaenicophaeuscurvirostriscurvirostris* (Shaw, 1810)).

Possible syntype (*viridirufus*), RMNH.AVES.216854, adult male, mounted skin. Loc.: South Borneo, [Indonesia], ii.1845. Leg.: C.A.L.M. Schwaner (= *Phaenicophaeuscurvirostrismicrorhinus* von Berlepsch, 1895).

Possible syntype (*viridirufus*), RMNH.AVES.216856, adult male, mounted skin. Loc.: Martapoera, Borneo, [Indonesia]. Leg.: C.A.L.M. Schwaner (= *Phaenicophaeuscurvirostrismicrorhinus* von Berlepsch, 1895).

Possible syntype (*viridirufus*), RMNH.AVES.216858, adult male, relaxed mount. Loc.: Martapoera, Borneo, [Indonesia]. Leg.: C.A.L.M. Schwaner (= *Phaenicophaeuscurvirostrismicrorhinus* von Berlepsch, 1895).

Müller applied the name *P.viridirufus* to birds from Java, Sumatra, and Borneo. Hence three subspecies of *Phaenicophaeuscurvirostris* (Shaw, 1810) are involved: nominate *P.c.curvirostris* Shaw, 1810, from Java, *P.c.singularis* Parrot, 1907, from Sumatra and *P.c.microrhinus* von Berlepsch, 1895, from Borneo. Bonaparte applied the name *P.erythrognathus* to birds from Sumatra only.

RMNH.AVES 216854, 216856, and 216858 are listed here as possible syntypes because either the year of collecting is unknown or it is from the same year as publication by Müller, which raises the question whether they were available to him in time. They are, however, the only Bornean specimens in the RMNH collection which might fit as type.

***Phoenicophauselongatus*** Müller, 1836: 342, pl. IV, fig. 5.

= *Phaenicophaeustrististristis* (Lesson, 1830).

Syntype, RMNH.AVES.88268, adult male, mounted skin. Loc.: [Batang Singgalan], Sumatra, [Indonesia], [vi.1833–1835]. Leg.: S. Müller.

Syntype, RMNH.AVES.88269, adult female, mounted skin. Loc.: [Batang Singgalan], Sumatra, [Indonesia], [vi.1833–1835]. Leg.: S. Müller.

The 1835 volume of the Tijdschrift voor Natuurlijke Geschiedenis en Physiologie 2: 315–355 was published in 1836 (Richmond 1926: 141).

Müller, who visited West Sumatra from June 1833 until late 1835, described how he collected an adult and a juvenile male.

***Piayacirce*** Bonaparte, 1850a: 110.

= *Piayacayanacirce* Bonaparte, 1850.

Lectotype, RMNH.AVES.88259, adult male, mounted skin. Loc.: Caracas, Venezuela. Leg.: R.F. van Lansberge.

Schlegel (1864b: 58) designated the lectotype. See also Junge (1937b: 184).

***Piayamehleri*** Bonaparte, 1850a: 110.

= *Piayacayanamehleri* Bonaparte, 1850.

Lectotype, RMNH.AVES.88260, immature, sex unknown, mounted skin. Loc.: Bogota, Colombia. Leg.: R.F. van Lansberge.

Schlegel (1864b: 57) designated the lectotype. See also Junge (1937b: 185).

***Cuculusmelanogaster*** Vieillot, 1817b: 236.

= *Piayamelanogaster* (Vieillot, 1817).

Lectotype, RMNH.AVES.88261, adult, sex unknown, relaxed mount. Loc.: “Java” [error]. Ex: Cabinet Temminck.

Vieillot (1817b) gave no indication of the number of specimens available to him. By listing RMNH.AVES.88261 as holotype, Van den Hoek Ostende et al. (1997: 147) designated it the lectotype.

The locality “Java” is erroneous. Schlegel (1864b: 59) proposed Guyana, while Von Berlepsch and Hartert (1902: 97) substitute Cayenne as locality.

***Phænicophæuscalyorhynchus*** Temminck, 1825: livr. 59, pl. 349.

= *Rhamphococcyxcalyorhynchuscalyorhynchus* (Temminck, 1825).

Syntype, RMNH.AVES.88262, adult male, skin. Loc.: Gorontalo, Celebes [= Sulawesi], [Indonesia], [ix–xii.1820]. Leg.: C.G.C. Reinwardt, 1821.

Syntype, RMNH.AVES.88263, adult female, skin. Loc.: Grorontalo, Celebes [= Sulawesi], [Indonesia], [ix–xii.1820]. Leg.: C.G.C. Reinwardt, 1821.

A third syntype is in the MNHN (Voisin and Voisin 1999: 383; MNHN C.G.1997–1201) where it arrived in 1823. This is also the type species for the genus name *Rhamphococcyx*.

Reinwardt visited North Sulawesi between September and December 1820.

***Scythropsnovaehollandiaefordi*** Mason & Forrester, 1996: 225.

= *Scythropsnovaehollandiaefordi* Mason & Forrester, 1996.

Paratype, RMNH.AVES.215637.

The holotype is in the AMNH (AMNH 110974). RMNH.AVES.215676 (cat. no 37, paratype, adult female from Boni, South Sulawesi, collected by Rookmaker on 28.x.1912) was exchanged with and sent to CSIRO in 1995.

***Scythropsnovaehollandiaeschoddei*** Mason & Forrester, 1996: 226.

= *Scythropsnovaehollandiaeschoddei* Mason & Forrester, 1996.

Paratype, RMNH.AVES.215669.

The holotype is in the AMNH (AMNH 628996).

***Pseudornis Dicruroïdes*** Hodgson, 1839: 136.

= *Surniculusdicruroidesdicruroides* (Hodgson, 1839).

Possible paralectotype, RMNH.AVES.214848.

Hodgson (1839: 136) wrote having sent this form to London although he did not specify the number of specimens. According to the range in measurements and referring to “sexes alike”, more specimens were involved. It is not clear how and when the RMNH acquired its specimen, so we list it here as “possible”. Warren (1966: 81) erroneously listed a specimen in the NHM (NHMUK 1843.11.13.1008) as holotype, thereby designating it the lectotype.

### ﻿PTEROCLIFORMES

#### ﻿Pteroclidae

***Pteroclessetarius*** Temminck, 1815a: 256.

= *Pteroclesalchataalchata* (Linnaeus, 1766).

Temminck (1815a) based this name on specimens in his own cabinet. The RMNH collection holds several mounted specimens which could be syntypes but due to insufficient data cannot be linked with certainty to Temminck’s cabinet.

***Pteroclesbicinctus*** Temminck, 1815a: 247.

= *Pteroclesbicinctusbicinctus* Temminck, 1815.

Syntype, RMNH.AVES.87613, adult male, mounted skin. Loc.: “Groote Vischrivier”, Namibia. Leg.: F. Levaillant.

Syntype, RMNH.AVES.87614, adult female, mounted skin. Loc.: “Groote Vischrivier”, Namibia. Leg.: F. Levaillant.

***Pteroclescoronatus*** Lichtenstein, 1823: 65.

= *Pteroclescoronatuscoronatus* Lichtenstein, 1823.

Syntype, RMNH.AVES.90767, adult male, mounted skin. Loc.: Nubie [= Sudan]. Leg.: W.F. Hemprich and C.G. Ehrenberg.

Syntype, RMNH.AVES.90768, adult female, mounted skin. Loc.: Nubie [= Sudan]. Leg.: W.F. Hemprich and C.G. Ehrenberg.

Three syntypes collected by Hemprich and Ehrenberg in Nubie are in the MfN (ZMB 11428–11439). Schifter et al. (2007: 143) listed two syntypes in the NMW (NMW 572 and 573).

***Pteroclesexustus*** Temminck, 1825: livr. 60, pl. 354 (male) and livr. 61, pl. 360 (female).

= *Pteroclesexustusexustus* Temminck, 1825.

Lectotype, RMNH.AVES.87615, adult male, mounted skin. Loc.: Senegal. Leg.: -.

Paralectotype, RMNH.AVES.87616.

During the preparation of this catalogue the authors were in doubt about the type status of the above listed specimens based on the possibility that Temminck’s *exustus* could be interpreted as a nomen novum for *senegalensis* Lichtenstein, 1823, which is preoccupied by *Tetraosenegalensis* Shaw, 1810, a junior synonym of *Tetraosenegallus* Linnaeus, 1771. After discussions with C. Gouraud and consultation of, among others, F. Rheindt, F. Steinheimer, and J. van Tol, it was concluded that this was not a clear case of a new name and ICZN art.72.7 does not apply. Hence, we follow Gouraud et al. (2016) who selected RMNH.AVES.87615 as lectotype. See also Gouraud (2015: 135).

***Pterocleslichtensteinii*** Temminck, 1825: livr. 60, pl. 355 (male, not pl. 335 as mentioned in the text).

= *Pterocleslichtensteiniilichtensteinii* Temminck, 1825.

Syntype, RMNH.AVES.87617, adult male, mounted skin. Loc.: Nubie [= Sudan]. Leg.: W.F. Hemprich and C.G. Ehrenberg.

Syntype, RMNH.AVES.87618, adult female, mounted skin. Loc.: Nubie [= Sudan]. Leg.: -.

Syntype, RMNH.AVES.90761, adult male, mounted skin. Loc.: Nubie [= Sudan]. Leg.: -.

According to the original description other syntypes are in the MfN and SMF. The female was depicted on pl. 361 which appeared one month after the original description (Dickinson 2001: 51).

***Pteroclestachypetes*** Temminck, 1815a: 274.

= *Pteroclesnamaqua* (Gmelin, 1789).

Syntype, RMNH.AVES.87619, adult female, mounted skin. Loc.: “Cap”, [South Africa]. Leg.: -.

Syntype, RMNH.AVES.87620, adult male, mounted skin. Loc.: “Cap”, [South Africa]. Leg.: -.

Syntype, RNMH.AVES.90762, adult male, mounted skin. Loc.: “Cap”, [South Africa]. Leg.: -.

Syntype, RMNH.AVES.90763, adult female, mounted skin. Loc.: “Cap”, [South Africa]. Leg.: -.

***Pteroclesquadricinctus*** Temminck, 1815a: 252.

= *Pteroclesquadricinctus* Temminck, 1815.

Temminck based his description on a male and female in the collection of Raye van Breukelerwaert. Although some of this collection was bought for the RMNH by Temminck at its sale in 1827, these specimens are not in the RMNH.

***Pteroclesguttatus*** Lichtenstein, 1823: 64.

= *Pteroclessenegallus* (Linnaeus, 1771).

Syntype, RMNH.AVES.90766, adult male, mounted skin. Loc.: Nubie [= Sudan]. Leg.: W.F. Hemprich and C.G. Ehrenberg.

Four syntypes collected by Hemprich and Ehrenberg in Nubie are in the MfN (ZMB 11424–11427). Schifter et al. (2007: 142) listed two syntypes in the NMW (NMW 566 and 567).

***SyrrhaptesPallasii*** Temminck, 1815a: 282.

= *Syrrhaptesparadoxus* (Pallas, 1773).

Temminck based his name on a drawing and description by prof. Fischer (Moscow) and reports of specimens collected by Pallas and Ireskin. Types are presumed to be in the MfN and Moscow.

### ﻿COLUMBIFORMES

#### ﻿Columbidae

***Chalcophapstimorensis*** Bonaparte, 1856d: 948.

***Chalcophapsjavanicoides*** “Temminck” Bonaparte, 1856d: 948.

= *Chalcophapsindicaindica* (Linnaeus, 1758).

Lectotype for *timorensis*, syntype for *javanicoides*, RMNH.AVES.87624, adult male, mounted skin. Loc.: Miromaffo [= Mount Miomaffo], Timor, [Indonesia], [ix.1829]. Leg.: S. Müller.

According to Müller (1844: 196) he visited Mount Miomaffo in September 1829.

Bonaparte (1856d) published Temminck’s manuscript name *javanicoides* as a synonym of *timorensis*. RMNH.AVES.87624 was erroneously listed as holotype for *timorensis* by Van den Hoek Ostende et al. (1997: 83). However, Bonaparte gave no indication of the number of specimens. By listing it as holotype Van den Hoek Ostende et al. (1997: 83) designated the lectotype.

***Peristerabornensis*** “Müller” Bonaparte, 1857: 91.

= *Chalcophapsindicaindica* (Linnaeus, 1758).

Lectotype, RMNH.AVES.87625, adult female, mounted skin. Loc.: Banjermassing, Borneo, [Indonesia], [28.vii.1836–17.xii.1836]. Leg.: S. Müller.

Bonaparte (1857) published Müller’s manuscript name in the synonymy of *Chalcophapsjavanica* Gmelin. RMNH.AVES.87625 was erroneously listed as holotype for *bornensis* by Van den Hoek Ostende et al. (1997: 84). However, Bonaparte gave no indication of the number of specimens. By listing it as holotype Van den Hoek Ostende et al. (1997: 84) designated the lectotype.

Müller visited Borneo from 28 July 1836 to 17 December 1836.

***Peristeraalbifrons*** Bonaparte, 1857: 92.

= *Chalcophapsstephaniwallacei* Brüggemann, 1877.

Syntype, RMNH.AVES.87626, adult male, mounted skin. Loc.: Gorontalo, Celebes [= Sulawesi], [Indonesia], [ix.1841–iv.1842]. Leg.: E.A. Forsten.

Syntype, RMNH.AVES.87627, adult female, mounted skin. Loc.: Gorontalo, Celebes [= Sulawesi], [Indonesia], [ix.1841–iv.1842]. Leg.: E.A. Forsten.

Forsten visited the area near Gorontalo from September 1841 until April 1842.

***ColumbaGodefrida*** Temminck, 1811: livr. 14/15, p. 125, pl. 57.

= *Claravisgeoffroyi* (Temminck, 1811).

Temminck (1811) based his description on a single specimen in the MNHN. For an explanation of the different spellings see David et al. (2010): the case of the spellings *godefrida* in the text and *geoffroyi* on the plate in parts 14/15 cannot be resolved under Article 24.2.4 of the Code, although Temminck used *godefrida* in the Table. This is because, in this case, the Table appeared in the same part. When Temminck (1813a: 297, 476) used *geoffroii*, he introduced an incorrect subsequent spelling. Bonaparte (1857: 75) listed both *godefrida* and *geoffroyi* and used *geoffroyi*, thus being the First Reviser under Article 24.2.3 of the Code.

For dating of this name and those of other pigeons and doves by Temminck in the years 1808–1811, see Dickinson et al. (2010).

***Peristeralansbergii*** Schlegel, 1873b: 139.

= *Claravismondetoura* (Bonaparte, 1856).

Syntype, RMNH.AVES.87629, adult male, mounted skin. Loc.: Caracas, Venezuela. Leg.: R.F. van Lansberge.

Syntype, RMNH.AVES.87630, immature male, mounted skin. Loc.: Caracas, Venezuela. Leg.: R.F. van Lansberge.

Collected between 1842 and 1855 when van Lansberge was governor in Caracas.

***Columbacinerea*** Temminck, 1811: 126, pl. 58 (nec Scopoli, 1786).

***Peristerapretiosa*** Ferrari-Perez, 1886: 175 (nomen novum).

= *Claravispretiosa* (Ferrari-Perez, 1886).

Holotype, RMNH.AVES.87631, adult male, mounted skin. Loc.: Brazil. Ex: Cabinet Temminck.

Holotype by monotypy.

Ferrari-Perez (1886) introduced *pretiosa* as nomen novum for *cinerea* Temminck. RMNH.AVES.87631 is therefore also the holotype of *pretiosa*.

***Columbaalbitorques*** Rüppell, 1837: 63, pl. 22, fig. 1.

= *Columbaalbitorques* Rüppell, 1837.

Paralectotypes, RMNH.AVES.90926–90928.

The lectotype was designated by Steinbacher (1949: 106) and is in SMF (SMF 12605). Other paralectotypes are in SMF and NHM (Steinheimer 2005a: 241). According to correspondence in the RMNH archives (‘Angebotene Vögel aus Abyssinien, abgebildet in meiner Abyssinia Fauna’ [‘Birds offered from Abyssinia, depicted in my Abyssinia Fauna’]), the RMNH specimens were bought from Rüppell between September and October 1837.

***Myristicivoragrisea*** “Gray” Bonaparte, 1854b: 1078.

[***Myristicivora*] *argentea*** “Temminck” Bonaparte, 1854b: 1078

***Columbaargentina*** “Temminck” Bonaparte, 1857: 36.

= *Columbaargentina* Bonaparte, 1855 [error for 1857].

Lectotype for *argentina*, syntype for *grisea* and *argentea*, RMNH.AVES.87634, immature, relaxed mount. Loc.: Pontianak, Borneo, [Indonesia]. Leg.: P.M. Diard.

Gray (1844: 5) published *Columbagrisea* as a nomen nudum. Bonaparte formerly described *Myristicivoragrisea* in 1854 with [*Myristicivora*] *argentea*, a manuscript name by Temminck, in synonymy. In 1857, Bonaparte published the name *Columbaargentina*? “Temm.” in synonymy of *Myristicivoragrisea*. However, *Myristicivoragrisea* Bonaparte, 1854, is preoccupied by *Columbagrisea* Bonnaterre, 1790, making *argentea* Bonaparte, 1854, the first available name, not *argentina* Bonaparte, 1857.

Bonaparte (1854b) gave no specific information about the specimens available to him but only mentioned Borneo as terra typica for *Myristicivoragrisea* Bonaparte, 1854. This excludes the specimen in the NHM from the Indian Archipelago (NHMUK 1845.1838) from the type series which is listed as the (lecto)type by Salvadori (1893: 249).

By erroneously listing RMNH.AVES.87634 as the holotype of *Myristicivoraargentina*Van den Hoek Ostende et al. (1997: 85) selected it as lectotype.

***Colomba Arquatrix*** Temminck, 1808: livr. 3, p. 11, pl. 5.

= *Columbaarquatrix* Temminck, 1808.

Holotype, RMNH.AVES.87637, adult, sex unknown, relaxed mount. Loc.: “Pays Auteniquoi” [South Africa]. Leg.: ?F. Levaillant. Ex: Cabinet Temminck.

Holotype by monotypy.

***Stictoenasarquatricula*** Bonaparte, 1854c: 1105.

= *Columbaarquatrix* Temminck, 1808.

Possible syntype, RMNH.AVES.87638, immature, relaxed mount. Loc.: “Cap”, [South Africa]. Leg.: -.

According to the original label this specimen was collected in South Africa (“Cap”), while Bonaparte (1854c) gave Abyssinia as its origin. However, according to Schlegel (1873b: 72) RMNH.AVES.87638 is one of the types of *arquatricula* Bonaparte.

***Columbajanthina*** Temminck, 1830: livr. 86 [85], pl. 503.

= *Columbajanthinajanthina* Temminck, 1830.

Lectotype, RMNH.AVES.87640, adult male, mounted skin. Loc.: Japan. Leg.: H. Bürger.

Paralectotype, RMNH.AVES.87660.

No livraison number is given under the text. According to Dekker et al. (2001: 203) and Dickinson (2001: 45) this name was published in livraison 85, not 86 as mentioned by Sherborn (1898: 488) and Peters (1937: 69).

Schlegel (1873b: 75) selected the lectotype. Voisin et al. (2005: 845) listed a paralectotype in the MNHN (MNHN 2002-543), received in exchange from Temminck in March 1836. One went to Frankfurt in March 1835.

The word “Usinato” written under the stand of RMNH.AVES.87640 does not refer to a locality as indicated in the first edition of this catalogue, but to the Japanese name for the species “Usibato”.

***ColumbaLarvata*** Temminck, 1809: livr. 7, p. 71, pl. 31.

= *Columbalarvata* Temminck, 1809.

Holotype, RMNH.AVES.87621, adult, sex unknown, relaxed mount. Loc.: “Pays Auteniquoi” [South Africa]. Leg.: ?F. Levaillant. Ex: Cabinet Temminck.

Holotype by monotypy. Temminck wrote that Levaillant donated him a male of this species.

***ColumbaErythrotorax*** Temminck, 1809: livr. 7, p. 15, pl. 7.

= *Columbalarvata* Temminck, 1809.

Temminck (1809) referred to two specimens in the MNHN and two other specimens in the collection of Dufresne (depicted specimens).

Junior synonym of *ColumbaLarvata* Temminck, 1809 which was published in the same work, though slightly later. For dating of this name and those of other pigeons and doves by Temminck in the years 1808–1811, see Dickinson et al. (2010).

***Columbaleucomela*** Temminck, 1821: 126.

= *Columbaleucomela* Temminck, 1821.

Holotype by monotypy. Collected by Westall in “New Holland” [= Australia]. Formerly in the collection of the Linnean Society, now in the NHM (NHMUK 1863.7.6.12).

***Palumbuscasiotis*** Bonaparte, 1854c: 1103.

***Columbapalumbushimalayana*** Schlegel, 1873b: 66.

= *Columbapalumbuscasiotis* (Bonaparte, 1854).

Syntype, RMNH.AVES.87643, adult female, mounted skin. Loc.: “Tartarie chinoise”, Himalaya. Leg.: -. (Syntype of *Palumbuscasiotis* and *Columbapalumbushimalayana*).

Syntype, RMNH.AVES.87642, adult, sex unknown, mounted skin. Loc.: Himalaya. Leg.: -. (Syntype of *Columbapalumbushimalayana*).

Syntype, RMNH.AVES.87644, adult, sex unknown, mounted skin. Loc.: Nepal. Ex: G.A Frank, 1860. (Syntype of *Columbapalumbushimalayana*).

Syntype, RMNH.AVES.87645, adult, sex unknown, mounted skin. Loc.: Nepal. Ex: G.A. Frank, 1860. (Syntype of *Columbapalumbushimalayana*).

Voisin et al. (2005: 843) incorrectly listed a specimen in the MNHN as holotype (by monotypy) of *Palumbuscasiotis* Bonaparte, 1854 as they incorrectly referred to Bonaparte (1857: 42) ‘Conspectus Generum Avium II’ as the original description. However, Bonaparte published *casiotis* three years earlier in 1854(c). He gave no indication of the (number of) specimens he had seen and referred to two additional specimens in the Calcutta Museum (Blyth 1849: 233, B and C). The locality given is “Himal. s. occ. Tataria chinensi” [Northwestern Himalaya and Chinese Tartary] (Bonaparte 1854a) and “Tartarie chinoise” (Bonaparte 1854d), which agrees with the locality on the label of RMNH.AVES.87643.

***Columbapollenii*** Schlegel, 1865c: 87.

= *Columbapollenii* Schlegel, 1865.

Holotype, RMNH.AVES.87646, adult, sex unknown, mounted skin. Loc.: Mayotte, Comoro Archipelago, 10.v.1864. Leg.: F.P.L. Pollen and D.C. van Dam.

Holotype by monotypy. Schlegel wrote that Pollen and Van Dam collected a single specimen.

Pages 1–180 of Vol. 3, ‘Nederlandsch Tijdschrift voor de Dierkunde’ were published in 1865 (Pieters and Dickinson 2005: 107).

***Columbakitlitzii*** Temminck, 1836: livr. 98, no plate.

= *Columbaversicolor* Kittlitz, 1832.

Extinct. According to the original description, syntypes from Bonin and Japan are in St. Petersburg and in the SMF.

***Janthoenasluzoniensis*** Schlegel, 1873b: 75.

= *Columbavitiensisgriseogularis* (Walden & Layard, 1872).

Holotype, RMNH.AVES.87647, adult, sex unknown, mounted skin. Loc.: Luzon, Philippines. Leg.: J.H. Gevers, 1862.

Holotype by monotypy. Schlegel listed only a single specimen.

***Janthœnasalbigularis*** “Temminck” Bonaparte, 1854: 1105.

***Janthaenashalmaheira*** Bonaparte, 1857: 44.

= *Columbavitiensishalmaheira* (Bonaparte, 1857).

Syntype, RMNH.AVES.87648, adult male, mounted skin. Loc.: Gilolo, [Halmahera], [Indonesia], [vi.1841–ix.1841]. Leg.: E.A. Forsten.

Syntype, RMNH.AVES.87649, immature male, mounted skin. Loc.: Gilolo, [Halmahera], [Indonesia], [vi.1841–ix.1841]. Leg.: E.A. Forsten.

Syntype, RMNH.AVES.87650, immature, sex unknown, mounted skin. Loc.: Gilolo, [Halmahera], [Indonesia], [vi.1841–ix.1841]. Leg.: E.A. Forsten.

Syntype, RMNH.AVES.87651, adult female, mounted skin. Loc.: Ceram, [Indonesia], [1842]. Leg.: E.A. Forsten.

*Janthoenasalbigularis* Bonaparte, 1854 is a nomen nudum and replaced by *halmaheira* Bonaparte, 1857 (not 1855 as in IOC 11.1).

During a forced stay due to illness on Ternate from 19 June 1841 until mid September 1841, Forsten sent his hunters to Halmahera to collect skins. Forsten visited Seram late 1842.

***Columbametallica*** Temminck, 1835: livr. 95, pl. 562.

= *Columbavitiensismetallica* Temminck, 1835.

Holotype, RMNH.AVES.87652, adult male, mounted skin. Loc.: Miromaffo [= Mount Miomaffo], Timor, [Indonesia], ix.1829. Leg.: S. Müller.

Holotype by monotypy.

***ColumbaCruziana*** Prevost, 1842: 89, pl. 48.

= *Columbinacruziana* (Prevost, 1842).

Syntype, RMNH.AVES.219062, adult male, mounted skin. Loc.: Bolivia [error]. Leg.: A. d’Orbigny.

Syntype, RMNH.AVES.219063, adult female, mounted skin. Loc.: Bolivia [error]. Leg.: A. d’Orbigny.

According to Voisin et al. (2005: 854) the locality Bolivia must be an error as the species does not occur there, Tacna in Peru is the correct locality.

***ColumbaPicui*** Temminck, 1813a: 435.

= *Columbinapicuipicui* (Temminck, 1813).

In Temminck (1810: 29) the vernacular name used by De Azara (1802–1805) was mentioned in the text of *Columbaminuta* Latham. In Temminck (1813a) it was introduced as a new species.

***Columbasquammata*** Lesson, 1831: 474.

***ColumbaSquamosa*** Temminck, 1811: 127, pl. 59 (nec Bonnaterre, 1790).

= *Columbinasquammatasquammata* (Lesson, 1831).

Lectotype, RMNH.AVES.87875, adult, sex unknown, mounted skin. Loc.: Brazil. Leg.: -.

Lesson (1831) based his description on *Columbasquamosa* Temminck, 1811. Temminck (1811) based his description on a specimen in the MNHN and a male in his own cabinet from Hoffmannsegg. Temminck wrote that the male specimen in his cabinet resembled the specimens in the museum in Paris. Voisin et al. (2005: 853) claimed, however, the MNHN specimen to be the holotype by monotypy. That is not correct. Because Van den Hoek Ostende et al. (1997: 109) also erroneously listed RMNH.AVES.87875 as holotype, they by doing so designated it as lectotype. The specimen in the MNHN (MNHN C.G.2002-5270) is therefore paralectotype.

*ColumbaSquamosa* is also the type of the genus name *Scardafella* Bonaparte, 1855.

***ColumbaTalpacoti*** Temminck, 1810: livr. 12/13, p. 22, pl. 12.

= *Columbinatalpacotitalpacoti* (Temminck, 1810).

Temminck (1810) based his description on a specimen in his own cabinet and in several other collections, including the MNHN where syntypes are confirmed by Voisin et al. (2005: 856).

For dating of this name and those of other pigeons and doves by Temminck in the years 1808–1811, see Dickinson et al. (2010).

***ColumbaHolosericea*** Temminck, 1809: livr. 8, p. 73, pl. 32.

= *Drepanoptilaholosericea* (Temminck, 1809).

Holotype, RMNH.AVES.87653, adult, sex unknown, skin. Loc.: Îles des Pins, New Caledonia. Ex: Cabinet Temminck.

Holotype by monotypy. Temminck (1809) wrote that he only knew the specimen which was used as a model here.

Temminck described the species as originating from the Sandwich Islands [= Hawaii] which is clearly erroneous, since it does not occur there. According to Schlegel (1873b: 41) this specimen originated from Île des Pins, New Caledonia.

***CarpophagaVandepolli*** Büttikofer, 1896c: 190.

= *Duculaaeneaconsobrina* (Salvadori, 1887).

Holotype, RMNH.AVES.87654, adult female, skin. Loc.: Hili Madjeio, Nias, [Indonesia], 19.xi.1895. Leg.: J.Z. Kannegieter. Ex: J.R.H. Neervoort van de Poll, 1896.

Holotype by monotypy. Büttikofer (1896c) based his description on a single female.

***Carpophaganuchalis*** Cabanis, 1882: 126.

= *Duculaaeneanuchalis* (Cabanis, 1882).

Syntype, RMNH.AVES.87655, adult, sex unknown, mounted skin. Loc.: Luçon (= Luzon), Philippines. Leg.: J.H. Gevers, 1862.

The other syntype is in the MfN.

***Duculapaulina*** Bonaparte, 1854b: 1076.

= *Duculaaeneapaulina* Bonaparte, 1854.

Syntype, RMNH.AVES.87656, adult male, mounted skin. Loc.: Tondano, Celebes [= Sulawesi], [Indonesia], vii.1840. Leg.: E.A. Forsten.

Syntype, RMNH.AVES.87657, adult male, mounted skin. Loc.: Tondano, Celebes [= Sulawesi], [Indonesia], 1841. Leg.: E.A. Forsten.

Forsten visited the Tondano area from 22 March 1840 to 19 June 1841.

***Duculaaeneasulana*** Siebers, 1929: 152.

= *Duculaaeneapaulina* Bonaparte, 1854.

Holotype, RMNH.AVES.14023, adult female, skin. Loc.: Sula-Besi, [Indonesia], 1913. Leg.: Tarip. Ex: MZB, 12.i.1950 (MZB 5893).

There are seven paratypes in the MZB (MZB 5888–5892, 5894 and 5895).

***Columbamuscadivora*** “Temminck” Bonaparte, 1850a: 32.

= *Duculaaeneapolia* (Oberholser, 1917).

Possible syntype, RMNH.AVES.215540, adult male, mounted skin. Loc.: Bima, Sumbawa [Indonesia]. Leg.: E.A. Forsten.

Bonaparte (1850a) published this manuscript name by Temminck in the synonymy of *Carpophagaaenea* (Linnaeus, 1766).

RMNH.AVES.215540 is listed here as a possible syntype because of its reference to *muscadivora* written in Temminck’s handwriting under the stand.

***Duculaproblematica*** Rensch, 1931: 372.

= *Duculaaeneapolia* (Oberholser, 1917).

Holotype, RMNH.AVES.14094, adult male, skin. Loc.: Laora, Sumba, [Indonesia], 13.iv.1925. Leg.: K.W. Dammerman. Ex: MZB, 12.i.1950 (MZB 5019).

Rensch (1931) probably erred when he stated that the holotype was in the MfN where it is not (S. Frahnert, in litt. 27 October 2020).

***Columbacapistrata*** Temminck: 1822: livr. 28, pl. 165.

= *Duculabadiabadia* (Raffles, 1822).

Schifter et al. (2007: 150–151) listed a syntype from Java in the NMW (NMW 66058), received in exchange from the RMNH in 1823. One arrived in the MfN in March 1833.

***Duculabasilica*** Bonaparte, 1854b: 1076.

= *Duculabasilicabasilica* Bonaparte, 1854.

Syntype, RMNH.AVES.87714, adult male, mounted skin. Loc.: Dodingo, Halmahera, [Indonesia]. Leg.: E.A. Forsten.

Syntype, RMNH.AVES.87715, adult female, mounted skin. Loc.: Dodingo, Halmahera, [Indonesia]. Leg.: E.A. Forsten.

During a forced stay due to illness on Ternate from 19 June 1841 until mid-September 1841, Forsten sent his hunters to Halmahera to collect skins.

***ColumbaLittoralis*** Temminck, 1808: 15, pl.7.

= *Duculabicolor* (Scopoli, 1786).

Temminck based his description primarily on reports by Leschenault. Specimens in the MNHN collected by Leschenault before the publication could be considered types.

***Duculamelanurasiebersi*** van Bemmel, 1940: 335.

= *Duculabicolor* (Scopoli, 1786).

Holotype, RMNH.AVES.14022, adult female, skin. Loc.: En’botit, Buru, [Indonesia], 03.iii.1921. Leg.: L.J. Toxopeus. Ex: MZB, 12.i.1950 (MZB 13566).

There are three paratypes in the MZB (MZB 15640–15642).

***Columbacineracea*** Temminck, 1835: livr. 95, pl. 563.

= *Duculacineracea* (Temminck, 1835).

Lectotype, RMNH.AVES.87658, adult, sex unknown, mounted skin. Loc.: Timor, [Indonesia], [x.1828–xii.1829]. Leg.: S. Müller.

Paralectotype, RMNH.AVES.87922.

Schlegel (1873b: 91) selected the lectotype.

Müller visited Timor from October 1828 until December 1829, the area of Mount Miomaffo between 7 and 30 September 1829.

***Carpophagaconcinna*** Wallace, 1865: 383.

= *Duculaconcinna* (Wallace, 1865).

Syntype, RMNH.AVES.87659, adult, sex unknown, mounted skin. Loc.: Matabello, [Indonesia], 1860. Leg.: A.R. Wallace. Ex: G.A. Frank.

According to Peters (1937: 45) the type locality Matabello is based on an error and should be Watubela (Moluccas).

RMNH.AVES.87659 is also a syntype for *Carpophagaroseinucha* Schlegel, 1866.

***ColumbaForsterii*** “Temminck” Prévost, 1843: 87, pl. 47.

***Hemiphagaforsteni*** Bonaparte, 1854b (nomen novum).

= *Duculaforsteni* (Bonaparte, 1854).

Syntype, RMNH.AVES.216523, male, mounted skin. Loc.: Manado, Celebes, [Indonesia], [16.v.1840]. Leg.: E.A. Forsten.

Syntype, RMNH.AVES.216524, female, mounted skin. Loc.: Manado, Tondano, [Indonesia], [16.v.1840]. Leg.: E.A. Forsten.

Syntype, RMNH.AVES.216525, male, mounted skin. Loc.: [Manado], Celebes, [Indonesia], [16.v.1840]. Leg.: E.A. Forsten.

Prévost (1842) dedicated this species to Forster, clearly a lapsus for E.A. Forsten. This was corrected by Bonaparte, who introduced *Hemiphagaforsteni* as a nomen novum.

According to field notes and collection lists by Forsten in the Naturalis archives, he collected eight specimens on 16 May 1840 near and between Tondano and Tomohon (P. van Wingerden, June 2022, pers. comm.). Three listed here as syntype are still in Leiden, the whereabouts of the remaining five specimens is not known to us. None are in the MNHN (Patrick Boussès, MNHN, pers. comm., 8 July 2022).

***Columbalacernulata*** Temminck, 1822: livr. 28, pl. 164.

= *Duculalacernulatalacernulata* (Temminck, 1822).

Lectotype, RMNH.AVES.87663, adult female, mounted skin. Loc.: Java, [Indonesia], [1816–1822]. Leg.: C.G.C. Reinwardt.

Schlegel (1873b: 96) selected the lectotype. According to the original description paralectotypes are in the MNHN although no specimens are listed in Voisin et al. (2004). One paralectotype is in the NMW (NMW 66057; Schifter et al. 2007: 150) which arrived there in 1823. One arrived in Berlin in March 1833, one in Leuven in August 1829, and one in Groningen in May 1845.

Reinwardt visited Java several times between April 1816 and March 1822.

***Columbaluctuosa*** Temminck, 1824: livr. 42, pl. 247.

= *Duculaluctuosa* (Temminck, 1824).

Syntype, RMNH.AVES.87664, adult, sex unknown, mounted skin. Loc.: Celebes [= Sulawesi], [Indonesia], [ix–xii.1821]. Leg.: C.G.C. Reinwardt.

Syntype, RMNH.AVES.87665, adult, sex unknown, mounted skin. Loc.: Celebes [= Sulawesi], [Indonesia], [ix–xii.1821]. Leg.: C.G.C. Reinwardt.

According to the original description other type specimens are in the MNHN. See Voisin et al. (2004: 124), who confirmed a syntype collected by Reinwardt on Celebes (MNHN 2002-148). Schifter et al. (2007: 149) listed another syntype in the NMW (NMW 48428) which arrived in exchange in 1823. For dating see Dickinson (2001: 46).

Reinwardt visited North Sulawesi between September and December 1821.

***Columbamullerii*** Temminck, 1835: livr. 96, pl. 566.

= *Duculamullerii* (Temminck, 1835).

Holotype, RMNH.AVES.87666, adult female, mounted skin. Loc.: Dourga River ou détroit de la Princesse Marianne. [Papua], [Indonesia], v.1829 [error for 21–27.v.1828]. Leg.: S. Müller.

Holotype by monotypy. Temminck (1835) wrote that his travellers collected a female.

The label indicates that the skin was collected in May 1829. This must be an error as Müller visited the Dourga River between 21 and 27 May 1828.

***Carpophagageelvinkiana*** Schlegel, 1873b: 86.

= *Duculamyristicivorageelvinkiana* (Schlegel, 1873).

Syntype, RMNH.AVES.88983, adult male, mounted skin. Loc.: Mefor [= Numfor], [Indonesia], 21.i.1869. Leg.: C.B.H. von Rosenberg.

Syntype, RMNH.AVES.88984, adult female, mounted skin. Loc.: Mefor [= Numfor], [Indonesia], 24.i.1869. Leg.: C.B.H. von Rosenberg.

Syntype, RMNH.AVES.88985, adult male, mounted skin. Loc.: Mefor [= Numfor], [Indonesia], 27.i.1869. Leg.: C.B.H. von Rosenberg.

Syntype, RMNH.AVES.88986, adult male, mounted skin. Loc.: Mefor [= Numfor], [Indonesia], 28.i.1869. Leg.: C.B.H. von Rosenberg.

Syntype, RMNH.AVES.88987, adult male, mounted skin. Loc.: Soëk [= Biak], [Indonesia], 23.iii.1869. Leg.: C.B.H. von Rosenberg.

Syntype, RMNH.AVES.88988, adult female, mounted skin. Loc.: Soëk [= Biak], [Indonesia], 11.iii.1869. Leg.: C.B.H. von Rosenberg.

Syntype, RMNH.AVES.88989, adult male, mounted skin. Loc.: Meosnoum [= Mios Num], [Indonesia], 09.v.1869. Leg.: C.B.H. von Rosenberg.

RMNH.AVES.88984 is given as a female on the label, but as a male under the stand.

***Carpophagaroseinucha*** Schlegel, 1866b: 197.

= *Duculamyristicivoramyristicivora* (Scopoli, 1786) but see below.

Syntype, RMNH.AVES.87659, adult, sex unknown, mounted skin. Loc.: Matabello, [Indonesia]. Leg.: A.R. Wallace, 1860 (= *Duculaconcinna* (Wallace, 1865)).

Syntype, RMNH.AVES.87667, adult male, mounted skin. Loc.: Rawak [= Luwak, off Waigeu], [Indonesia], [16.xii.1818–05.i.1819]. Leg.: J.R.C. Quoy and J.P. Gaimard, Expedition de l’Uranie. Ex: Le Prevost, MNHN, 1836 (= *Duculamyristicivoramyristicivora* (Scopoli, 1786)).

Syntype, RMNH.AVES.87668, adult female, mounted skin. Loc.: Sanghir, [Indonesia], 30.x.1865 or 1.xi.1865. Leg.: C.B.H. von Rosenberg (= *Duculaconcinna* (Wallace, 1865)).

Syntype, RMNH.AVES.87669, adult male, mounted skin. Loc.: Gebe, [Indonesia], 22.ii.1863. Leg.: H.A. Bernstein (= *Duculamyristicivoramyristicivora* (Scopoli, 1786)).

Syntype, RMNH.AVES.87670, adult female, mounted skin. Loc.: Gebe, [Indonesia], 15.ii.1863. Leg.: H.A. Bernstein (= *Duculamyristicivoramyristicivora* (Scopoli, 1786)).

Syntype, RMNH.AVES.87671, adult female, mounted skin. Loc.: Gebe, [Indonesia], 02.ii.1863. Leg.: H.A. Bernstein (= *Duculamyristicivoramyristicivora* (Scopoli, 1786)).

Syntype, RMNH.AVES.87672, adult male, mounted skin. Loc.: Gebe, [Indonesia], 05.ii.1863. Leg.: H.A. Bernstein (= *Duculamyristicivoramyristicivora* (Scopoli, 1786)).

Syntype, RMNH.AVES.87673, adult male, mounted skin. Loc.: Gebe, [Indonesia], 07.ii.1863. Leg.: H.A. Bernstein (= *Duculamyristicivoramyristicivora* (Scopoli, 1786)).

Syntype, RMNH.AVES.87674, adult male, mounted skin. Loc.: Gebe, [Indonesia], 23.ii.1863. Leg.: H.A. Bernstein (= *Duculamyristicivoramyristicivora* (Scopoli, 1786)).

Syntype, RMNH.AVES.87675, adult female, mounted skin. Loc.: Gebe, [Indonesia], 15 or 25.ii.1863. Leg.: H.A. Bernstein (= *Duculamyristicivoramyristicivora* (Scopoli, 1786)).

Syntype, RMNH.AVES.87676, adult female, mounted skin. Loc.: Gagie [= Gag], [Indonesia], 04.iii.1863. Leg.: H.A. Bernstein (= *Duculamyristicivoramyristicivora* (Scopoli, 1786)).

Syntype, RMNH.AVES.87677, adult male, mounted skin. Loc.: Gagie [= Gag], [Indonesia], 05.iii.1863. Leg.: H.A. Bernstein (= *Duculamyristicivoramyristicivora* (Scopoli, 1786)).

Syntype, RMNH.AVES.87678, adult female, mounted skin. Loc.: Waigeu, [Indonesia], 17.iii.1863. Leg.: H.A. Bernstein. (= *Duculamyristicivoramyristicivora* (Scopoli, 1786)).

Syntype, RMNH.AVES.87679, adult male, mounted skin. Loc.: Waigeu, [Indonesia], 17.iii.1863. Leg.: H.A. Bernstein (= *Duculamyristicivoramyristicivora* (Scopoli, 1786)).

Syntype, RMNH.AVES.87680, adult male, mounted skin. Loc.: Waigeu, [Indonesia], 18.iii.1863. Leg.: H.A. Bernstein (= *Duculamyristicivoramyristicivora* (Scopoli, 1786)).

Syntype, RMNH.AVES.87681, adult female, mounted skin. Loc.: Waigeu, [Indonesia], 25.iii.1863. Leg.: H.A. Bernstein (= *Duculamyristicivoramyristicivora* (Scopoli, 1786)).

Syntype, RMNH.AVES.87682, adult female, mounted skin. Loc.: Gemien, [Indonesia], 09.v.1863. Leg.: H.A. Bernstein (= *Duculamyristicivoramyristicivora* (Scopoli, 1786)).

Syntype, RMNH.AVES.87686, adult male, mounted skin. Loc.: Sanghir, [Indonesia], 30.x.1865. Leg.: C.B.H. von Rosenberg (= *Duculaconcinna* (Wallace, 1865)).

Syntype, RMNH.AVES.87687, adult female, mounted skin. Loc.: Sanghir, [Indonesia], 30.x.1865. Leg.: C.B.H. von Rosenberg (= *Duculaconcinna* (Wallace, 1865)).

Although Schlegel (1866b) gave a detailed list of his type specimens, there is inconsistency between the specimens mentioned in the original description and the specimens he listed later (Schlegel 1873b: 81) and those which could be traced in the collection. In the original description, Schlegel listed 12 specimens from Gebe collected by Bernstein, four from Waigeu (Bernstein), two from Gagie (Bernstein), one from Gemien (Bernstein), one from Rawak collected during the expedition of the ‘Uranie’, one from Matabello (Wallace), one from New Guinea reported by Quoy and Gaimard from the expedition of the ‘Uranie’, and three from Sanghir (Von Rosenberg). Nineteen specimens fit this type series. Three specimens collected by Bernstein near Sorong in New Guinea (RMNH.AVES.87683–87685) listed as types by Van den Hoek Ostende et al. (1997: 91) are not part of the type series. Neither is a third specimen collected by Bernstein on Gagie in November 1864, as Schlegel explicitly mentioned two specimens. Five syntypes from Gebe are missing. Two specimens with ‘Gebe, 25. and 27. vii. 1863, leg.: D.S. Hoedt’ on their labels might fit as syntypes. However, there is no evidence of errors either on label data or in Schlegel’s list of specimens. All missing type specimens must have been exchanged shortly after the description since Schlegel (1873b) did not list them seven years later. There is no trace yet where these specimens currently are.

Voisin et al. (2004: 122) listed a type specimen (MNHN C.G.2002-141) for the name *Globicerapacifica* Bonaparte, 1854b (nec Gmelin) which might fit as syntype. It was collected in New Guinea and reported by Quoy and Gaimard from the expedition of the ‘Uranie’, but the label data is not detailed enough.

**Taxonomy**: In the second edition of his catalogue, Schlegel (1873b: 81–82) split *Carpophagaroseinucha* into two taxa: *Carpophagatumida* Wallace, 1865 (a younger synonym of *myristicivora* Scopoli, 1786) and *C.concinna* Wallace, 1865, which is still supported by current taxonomy.

*Duculamyristicivora* occurs on the West Papuan Islands (nominate race: Batanta, Gebe, Gag, Gam, Misool, Salawati, Schildpad, Sinapang, Waigeo, and Widis) and the islands in the Geelvink Bay (race geelvinkiana). It has also been recorded as a vagrant from New Guinea. *Duculaconcinna* is resident on countless small islands of the Sanghir and Talaud group, the Central and South Moluccan Islands and the islands south of Sulawesi. Birds from the Aru, Kei and Talaud Islands are sometimes treated as separate taxa: *D.c.aru* Salomonsen, 1934, *D.c.separata* (Hartert, 1896), and *D.c.intermedia* (Meyer & Wiglesworth, 1894), respectively. Dickinson (2003: 178) listed *D.concinna* as monotypic.

Consequently, the type series is composed of two taxa, *Duculamyristicivoramyristicivora* (Scopoli, 1786) and *Duculaconcinna* (Wallace, 1865). RMNH.AVES.87659 is also one of the syntypes of *Carpophagaconcinna* Wallace, 1865. Additionally, specimens collected by Von Rosenberg on Sanghir (RMNH.AVES.87686–87688) belong to this taxon.

**Nomenclature**: To exclude specimens which do not belong to the same taxon from being name-bearing types, it is necessary to designate a lectotype. Taking Schlegel’s description into account, the name has to be applied to birds known as *myristicivora*. However, since the type specimens identified as *myristicivora* originate from different West Papuan islands and Schlegel did not prefer one of them, the fixation of the name *roseinucha* to a single specimen and thus to one of these islands would be an arbitrary selection. In order to keep the name applicable to different islands and because *roseinucha* as a younger synonym does not (yet) affect current taxonomy, we do not propose or select a lectotype here.

***Carpophaganeglecta*** Schlegel, 1866b: 194.

= *Duculaneglecta* (Schlegel, 1866).

Syntype, RMNH.AVES.87690, adult male, mounted skin. Loc.: Amboina [= Ambon], [Indonesia], 10.iv.1827. Leg.: S. Müller.

Syntype, RMNH.AVES.87691, adult male, mounted skin. Loc.: Amboina [= Ambon], [Indonesia], ix.1828. Leg.: S. Müller.

Syntype, RMNH.AVES.87692, adult, sex unknown, mounted skin. Loc.: Amboina [= Ambon], [Indonesia]. Leg.: W.H. de Vries, 1862.

Syntype, RMNH.AVES.87693, adult male, mounted skin. Loc.: Amboina [= Ambon], [Indonesia], 08.iii.1863. Leg.: D.S. Hoedt.

Syntype, RMNH.AVES.87694, adult male, mounted skin. Loc.: Amboina [= Ambon], [Indonesia], 08.iii.1863. Leg.: D.S. Hoedt.

Syntype, RMNH.AVES.87695, adult female, mounted skin. Loc.: Ceram, [Indonesia], 04.iv.1863. Leg.: D.S. Hoedt.

Syntype, RMNH.AVES.87696, adult female, mounted skin. Loc.: Ceram, [Indonesia], 11.iv.1863. Leg.: D.S. Hoedt.

Syntype, RMNH.AVES.87697, adult male, mounted skin. Loc.: Ceram, [Indonesia], 13.iv.1863. Leg.: D.S. Hoedt.

Syntype, RMNH.AVES.87698, adult male, mounted skin. Loc.: Ceram, [Indonesia], 19.iv.1863. Leg.: D.S. Hoedt.

Syntype, RMNH.AVES.87699, adult male, mounted skin. Loc.: Ceram, [Indonesia], 19.iv.1863. Leg.: D.S. Hoedt.

Syntype, RMNH.AVES.87700, adult female, mounted skin. Loc.: Ceram, [Indonesia], 19.iv.1863. Leg.: D.S. Hoedt.

Syntype, RMNH.AVES.87701, adult male, mounted skin. Loc.: Boano, [Indonesia]. Leg.: D.S. Hoedt, 1863.

Syntype, RMNH.AVES.87702, adult female, mounted skin. Loc.: Boano, [Indonesia]. Leg.: D.S. Hoedt, 1863.

Syntype, RMNH.AVES.87703, adult, sex unknown, skeleton. Loc.: Amboina [= Ambon], [Indonesia], [iv.1827]. Leg.: S. Müller.

The sex of RMNH.AVES.87694 is not clear. According to the label it is a male; according to the stand it is a female.

***Columbaoceanica*** “Lesson and Garnot” Desmarest, 1826: 316.

= *Duculaoceanicaoceanica* (Desmarest, 1826).

Syntype, RMNH.AVES.87688, adult female, relaxed mount. Loc.: Kosuhai (Kusaie) [= Kosrae], [E. Carolina], 1823 [error = 5.vi.1824–15.vi.1824]. Leg.: R.P. Lesson (‘Voyage Coquille’).

The publication of the name *Columbaoceanica* by Desmarest in June 1826 predated the publication in Lesson and Garnot (1826b: pl. 41) of July 1826.

According to Lesson and Garnot (1826a: 423), the island Oualan (Kosrae) was visited by the ‘Coquille’ between 5 and 15 June 1824. The date of 1823 as mentioned on the label by Finsch must therefore be an error. According to this label the original data on the original label by Temminck was: “*Columbaoceanica* ♀ voy. Lesson Ualan”.

According to Voisin et al. (2004: 121) two syntypes collected on the Marianas Islands are in the MNHN (MNHN 2002-113 and 2002-114).

***Columbaperspicillata*** Temminck, 1824: livr. 42, pl. 246.

= *Duculaperspicillata* (Temminck, 1824).

Lectotype, RMNH.AVES.87689, adult male, mounted skin. Loc.: Dodingo, Halmahera, [Indonesia]. Leg.: ?E.A. Forsten.

Schlegel (1873b: 89) selected the lectotype.

The label mentions Forsten as the collector. This must be erroneous since Forsten arrived in Indonesia after the name was published in 1824. The specimen was probably collected by Reinwardt who visited Halmahera briefly in 1821. A similar mix-up is found in the type specimens of *Columbahyogastra* Temminck, 1824, which were presumably collected by Reinwardt and are also labelled ‘Forsten’.

According to the original description paratypes are in the MNHN but not listed in Voisin et al. (2004).

***Carpophagapinonjobiensis*** Schlegel, 1871a: 26.

***CarpophagaWestermanii*** “von Rosenberg” Schlegel, 1871a: 27.

= *Duculapinonjobiensis* (Schlegel, 1871).

Syntype, RMNH.AVES.87704, adult male, mounted skin. Loc.: Jobi Island, [Indonesia], 24.iv.1869. Leg.: C.B.H. von Rosenberg.

Syntype, RMNH.AVES.87705, adult male, mounted skin. Loc.: Jobi Island, [Indonesia], 9.iv.1869. Leg.: C.B.H. von Rosenberg.

Syntype, RMNH.AVES.87706, adult female, mounted skin. Loc.: Jobi Island, [Indonesia], 15.iv.1869. Leg.: C.B.H. von Rosenberg.

Syntype, RMNH.AVES.87707, adult female, mounted skin. Loc.: Jobi Island, [Indonesia], 16.iv.1869. Leg.: C.B.H. von Rosenberg.

Syntype, RMNH.AVES.87708, adult male, mounted skin. Loc.: Jobi Island, [Indonesia], 16.iv.1869. Leg.: C.B.H. von Rosenberg.

Syntype, RMNH.AVES.87709, adult female, mounted skin. Loc.: Jobi Island, [Indonesia], 21.iv.1869. Leg.: C.B.H. von Rosenberg.

Syntype, RMNH.AVES.87710, adult female, mounted skin. Loc.: Jobi Island, [Indonesia], 22.iv.1869. Leg.: C.B.H. von Rosenberg.

Schlegel (1871a) mentioned in his description that he received this series from Von Rosenberg under the manuscript name *Carpophaga Westermanii*. Published as a junior synonym, this is not an available name; however, subsequent use as a valid name (see for instance Schlegel 1873b: 95 and Salvadori 1893: 224) made this name available (ICZN 11.6.1).

***Columbarosacea*** Temminck, 1836: livr. 98, pl. 578.

= *Ducularosacea* (Temminck, 1836).

Lectotype, RMNH.AVES.87711, adult male, mounted skin. Loc.: Timor, [Indonesia], 1829. Leg.: S. Müller.

Paralectotypes, RMNH.AVES.87712, 255162.

The year of publication of livraison 97, usually given as 1835 following Sherborn (1898: 488), has been corrected to 1836 by Mees (1994: 51) and confirmed by Dickinson (2001: 47). The year of publication of livraisons 98 and 99 was subsequently corrected to 1836 by Dickinson (2001: 47), contra Sherborn (1898: 488) who gave 1835 for both livraisons.

Müller (1843: 159) only mentioned this species once in his account of Timor, when he visited the area of Prittie [= Pariti].

Schlegel (1873b: 88) selected the lectotype. According to Voisin et al. (2004: 125) another possible type is in the MNHN (MNHN 2002-143) where it arrived in May 1836.

***Globicerarubricera*** Bonaparte, 1854b: 1073.

= *Ducularubricerarubricera* (Bonaparte, 1854).

Syntype, RMNH.AVES.87713, adult, sex unknown, mounted skin. Loc.: New Ireland [Bismarck Archipelago]. Ex: MNHN.

***Ducularufigasterpallida*** Junge, 1952: 248.

= *Ducularufigasterrufigaster* (Quoy & Gaimard, 1832).

Holotype, RMNH.AVES.87716, adult male, skin. Loc.: Bivak Island, [Papua], [Indonesia], 25.i.1910. Leg.: H.A. Lorentz, Dutch New Guinea Expedition 1909.

Paratypes, RMNH.AVES.87717–87731.

***Ptilopushelviventris*** Von Rosenberg, 1866: 144.

= *Gallicolumbarufigulahelviventris* (von Rosenberg, 1866).

Holotype, RMNH.AVES.87734, adult male, skin. Loc.: Wokam, Aru Islands, [Indonesia], 13.iii.1865. Leg.: C.B.H. von Rosenberg.

Holotype by monotypy, Von Rosenberg (1866) based his description on a single male specimen. Although given as 1867 in Van den Hoek Ostende et al. (1997: 94), volume 29 of the ‘Natuurkundig Tijdschrift voor Nederlandsch Indië’ was already published in 1866 (Mees 1980).

***Columbatristigmata*** Bonaparte, 1855a: 207.

= *Gallicolumbatristigmatatristigmata* (Bonaparte, 1855).

Lectotype, RMNH.AVES.87736, adult male, skin. Loc.: Tondano, Celebes [= Sulawesi], [Indonesia], v.1841. Leg.: E.A. Forsten.

Bonaparte (1855a) gave no indication of the number of specimens available to him. Schlegel (1873b: 158) selected the lectotype.

***Columbahumeralis*** Temminck, 1821: 128.

= *Geopeliahumeralishumeralis* (Temminck, 1821).

Temminck (1821) referred to two specimens from “Broad-Sound, Cote Orientale, New Holland” [= Australia], one in the collection of the Linnean Society, now in the NHM (NHMUK 1863.7.6.14). The second one was in the NHM, but is no longer present (Van Grouw, pers. comm., 2022).

***ColumbaMaugeus*** Temminck, 1809: livr. 10/11, p. 11, pl. 52.

= *Geopeliamaugeus* (Temminck, 1809).

Temminck (1809) referred to two specimens in the MNHN where they are confirmed by Voisin et al. (2005: 852) and two other specimens in the collection of Dufresne (one ended up in Edinburgh, but is now lost (Jansen 2018: 275).

For dating of this name and those of other pigeons and doves by Temminck in the years 1808–1811, see Dickinson et al. (2010).

***Geopeliaplacida*** Gould, 1844: 55.

= *Geopeliaplacidaplacida* Gould, 1844.

Possible paralectotype, RMNH.AVES.218935.

Fisher and Calaby (2009: 118) listed this specimen as from the Gould collection and possibly part of the type series. Stone (1913: 134) selected the lectotype in the ANSP (ANSP 13437).

***Columbascripta*** Temminck, 1821: 127.

= *Geophapsscriptascripta* (Temminck, 1821).

Lectotype, RMNH.AVES.87737, adult, sex unknown, mounted skin. Loc.: Australia. Leg.: -.

It is clear that Temminck (1821) used two specimens for his description, an old male (without indicating where this specimen was seen) and a female or young male in the collection of the Linnean Society, London. Temminck gave no details for the old male specimen, but according to Schlegel (1873b: 155) RMNH.AVES.87737 is the type, hereby designating it the lectotype. The type from the Linnean Society is now in the NHM (NHMUK 1863.7.6.13) and listed by Warren (1966: 266) as holotype. Warren must have overlooked that the type series consists of two specimens and the lectotypification by Schlegel.

***ColumbaMystacea*** Temminck, 1811: livr. 14/15, p. 124, pl. 56.

= *Geotrygonmystacea* (Temminck, 1811).

Holotype, RMNH.AVES.87781, adult, sex unknown, mounted skin. Loc.: San Domingo [error]. Leg.: -.

Holotype by monotypy, as Temminck (1811) wrote the only specimen he had seen was part of his cabinet. The locality San Domingo must be an error, as this species does not occur there. It is found on the neighbouring island of Puerto Rico and San Domingo could be the port of shipment.

***Columbacristata*** Temminck, 1809: 20, pl. 9 (nec Gmelin, 1788).

= *Geotrygonversicolor* (Lafresnaye, 1846).

Syntype, RMNH.AVES.87738, adult, sex unknown, skin. Loc.: Jamaica. Ex: Cabinet Temminck.

According to the original description, another syntype was in London (collection not mentioned) and a third in the collection of Raye van Breukelerwaert.

***ColumbaViolacea*** Temminck, 1809: livr. 7, p. 67, pl. 29.

= *Geotrygonviolaceaviolacea* (Temminck, 1809).

Temminck (1809) based his description on a single specimen in the MNHN which is still there (Voisin et al. 2005: 861).

For dating of this name and those of other pigeons and doves by Temminck in the years 1808–1811, see Dickinson et al. (2010).

***Gouracoronataminor*** Schlegel, 1864a: 208.

= *Gouracristata* (Pallas, 1764).

Syntype, RMNH.AVES.87739, adult male, mounted skin. Loc.: Waigeu, [Indonesia], 18.iii.1863. Leg.: H.A. Bernstein.

Syntype, RMNH.AVES.87740, adult male, mounted skin. Loc.: Waigeu, [Indonesia], 19.iii.1863. Leg.: H.A. Bernstein.

Syntype, RMNH.AVES.87741, adult female, mounted skin. Loc.: Waigeu, [Indonesia], 02.iv.1863. Leg.: H.A. Bernstein.

Syntype, RMNH.AVES.87742, immature male, mounted skin. Loc.: Waigeu, [Indonesia], 13.iii.1863. Leg.: H.A. Bernstein.

A syntype was sent in exchange to the NMW in 1865 but could not be traced in 2002 (NMW 1865.IV.30; Schifter et al. 2007: 158).

***Gouracristatapygmaea*** Mees, 1965: 160.

= *Gouracristata* (Pallas, 1764).

Holotype, RMNH.AVES.87743, adult male, mounted skin. Loc.: Waigama, Misool, [Indonesia], 11.vii.1867. Leg.: D.S. Hoedt.

Paratypes, RMNH.AVES.87744–87747.

***GouraSteursii*** “Temminck” Gray, G.R., 1845: pl. CXX, p. 479.

= *Gouravictoriavictoria* (Fraser, 1844).

Syntype, RMNH.AVES.219710, adult male, mounted skin. Loc. Jobi [= Yapen], [Indonesia]. Leg.: [F.V.H.A. Ridder de Stuers, 1844].

Syntype, RMNH.AVES.219711, adult female, mounted skin. Loc. Jobi [= Yapen], [Indonesia]. Leg.: [F.V.H.A. Ridder de Stuers, 1844].

According to Mees (1994: 25) these are the oldest *Goura* specimens in the RMNH, received in 1844 from “Kolonel Steurs” (= probably F.V.H.A. Ridder de Stuers, Governor of the Moluccas in the 1840s). The locality is a later addition by Schlegel (1873b: 169).

The plate in Gray (1845) was drawn after a living bird in the aviary of the Earl of Derby, so if preserved, this specimen is also part of the type series.

***Columbamada*** Hartert, 1899: 33.

= *Gymnophapsmada* (Hartert, 1899).

Paralectotype, RMNH.AVES.87748.

Hartert gave no indication of the specimens available to him. Later Hartert (1927: 6) designated a type, but failed to give specific details to distinguish it. RMNH.AVES.87748 and two specimens in the AMNH (AMNH 611809 and 611810) fit the Harter’s “type” from which Greenway (1978: 50) selected AMNH 611809 as the lectotype.

***Columbagigas*** Ranzani, 1821: 223 (nomen novum).

= *Hemiphaganovaeseelandiaespadicea* (Latham, 1801).

Van den Hoek Ostende et al. (1979) erroneously listed RMNH.AVES.87749 as holotype for *Columbagigas* Ranzani, 1821. Schodde, in Schodde and Mason (1997: 53) indicated that *gigas* is a nomen novum for *Columbaspadicea* Latham, 1801.

***RynchaenasSchlegeli*** Von Rosenberg, 1866: 143.

= *Henicophapsalbifronsschlegeli* (von Rosenberg, 1866).

Syntype, RMNH.AVES.87750, adult male, mounted skin. Loc.: Wokam, Aru Islands, [Indonesia], 21.iii.1865. Leg.: C.B.H. von Rosenberg.

Syntype, RMNH.AVES.89421, adult female, mounted skin. Loc.: Wonoumbai, Aru Islands, [Indonesia], 29.vi.1865. Leg.: C.B.H. von Rosenberg.

Syntype, RMNH.AVES.89420, adult female, mounted skin. Loc.: Wonoumbai, Aru Islands, [Indonesia], 1.vii.1865. Leg.: C.B.H. von Rosenberg.

Although given as 1867 in Van den Hoek Ostende et al. (1997: 96), volume 29 of the ‘Natuurkundig Tijdschrift voor Nederlandsch Indië’ was published in 1866 (Mees 1980). Later in the same year, Schlegel (1866f: 345) listed *schlegeli*, with reference to Von Rosenberg as the original author, in synonymy of *Henicophapsalbifrons*.

***ColumbaArmillaris*** Temminck, 1808: 13, pl. 6.

= *Leucosarciamelanoleuca* (Latham, 1801).

Syntype, RMNH.AVES.87753, adult, sex unknown, skin. Loc.: Australia. Ex: Cabinet Temminck.

The depicted specimen is from Temminck’s Cabinet, who also referred to two specimens in London.

***Columbadilopha*** Temminck, 1821: 124.

= *Lopholaimusantarcticus* (Shaw, 1793).

Paralectotype, RMNH.AVES.87762.

Salvadori (1893: 236) listed NHMUK 1863.7.6.10 from the Linnean Society, London, as the type of *C.dilopha*, thereby designating it the lectotype. Warren (1966: 82) listed this specimen as syntype. Brookes owned the Brookesian Museum of Comparative Anatomy and sold his collection in 1826 of which 72 specimens were purchased by the RMNH.

***Macropygiagriseinucha*** Salvadori, 1876: 204.

***Macropygiamaforensis*** Salvadori, 1878a: 429.

= *Macropygiaamboinensisgriseinucha* Salvadori, 1876 and *Macropygiaamboinensismaforensis* Salvadori, 1878.

Syntype for *griseinucha* only, RMNH.AVES.217857, adult female, mounted skin. Loc.: Soëk [= Biak], [Indonesia], 16.iii.1869. Leg.: C.H.B. von Rosenberg.

Syntype for *griseinucha* only, RMNH.AVES.217859, immature male, mounted skin. Loc.: Soëk [= Biak], [Indonesia], 18.iii.1869. Leg.: C.H.B. von Rosenberg.

Syntype, RMNH.AVES.217879, adult male, mounted skin. Loc.: Mafoor [= Numfor], [Indonesia], i.1869. Leg.: C.B.H. von Rosenberg.

Syntype, RMNH.AVES.217881, adult male, mounted skin. Loc.: Mafoor [= Numfor], [Indonesia], i.1869. Leg.: C.B.H. von Rosenberg.

Syntype, RMNH.AVES.217883, adult male, mounted skin. Loc.: Mafoor [= Numfor], [Indonesia], i.1869. Leg.: C.B.H. von Rosenberg.

Syntype, RMNH.AVES.217885, adult male, mounted skin. Loc.: Mafoor [= Numfor], [Indonesia], 01.ii.1869. Leg.: C.B.H. von Rosenberg.

Salvadori (1876) described birds from Mafor [= Numfor], Miosnom [= Mios Num], Misori [= Biak] and Jobi [= Yapen] as *griseinucha*. Two years later he restricted *griseinucha* to Miosnom (Salvadori, 1878a: 431) and introduced *maforensis* as a new name for the birds from Mafor [= Numfor] (Salvadori, 1878a: 429).

Two or three taxa are involved: *Macropygiaamboinensismaforensis* Salvadori, 1878 from Numfor and *Macropygiaamboinensisgriseinucha* Salvadori, 1876 from Mios Num. IOC 11.1 is not clear about the subspecific status on Biak. Beehler and Pratt (2016) consider birds from Biak as *M.a.cinereiceps* Tristram, 1889 but Avibase considers the population on Biak as “not being identified to subspecies, but vocally similar to *maforensis*” (https://avibase.bsc-eoc.org).

***Macropygiakeyensis*** Salvadori, 1876: 204.

= *Macropygiaamboinensiskeyensis* Salvadori, 1876.

Syntype, RMNH.AVES.87764, adult male, mounted skin. Loc.: Groot Kei [= Kay Besar], [Indonesia], 30.iii.1865. Leg.: D.S. Hoedt.

Syntype, RMNH.AVES.217818, adult female, mounted skin. Loc.: Indonesia, 21.viii.1865. Leg.: C.B.H. von Rosenberg.

Salvadori (1876) described this species based on immature specimens collected by Beccari and descriptions by Von Rosenberg (1867: 81) and Schlegel (1873b: 114; RMNH.AVES.87764). We agree with Warren (1966: 151) that the specimen later listed by Salvadori (1893: 353) as “type of male” is not part of the type series.

***Macropygiaalbicapilla*** Bonaparte, 1854c: 1111.

= *Macropygiadoreyaalbicapilla* Bonaparte, 1854.

Lectotype, RMNH.AVES.87763, adult, sex unknown, mounted skin. Loc.: Celebes [= Sulawesi], [Indonesia], [iii.1840–iv.1842]. Leg.: E.A. Forsten.

Schlegel (1873b: 111) selected the lectotype.

Forsten stayed in Celebes twice between March 1840 and April 1842.

***Macropygiaalbiceps*** “Temminck” Bonaparte, 1854c: 1111.

= *Macropygiadoreyaalbiceps* Bonaparte, 1856.

Syntype, RMNH.AVES.90980, adult, sex unknown, mounted skin. Loc.: Ternate, [Indonesia], [19.vi.1841–ix.1841]. Leg.: E.A. Forsten.

Although Bonaparte (1854c) initially considered *albiceps* to be a junior synonym of *amboinensis* (Bonaparte, 1854c: 1111), he later (1856c: 839) distinguished this bird as a separate race. According to ICZN (art. 11.6.1) the first publication as a synonym counts as the date of publication, so it should be *Macropygiaalbiceps* Bonaparte, 1854.

Forsten visited Ternate from 19 June 1841 until mid-September 1841.

***Macropygiaphasianellabarussa*** Siebers, 1929: 152.

= *Macropygiaemilianaemiliana* Bonaparte, 1854.

Holotype, RMNH.AVES.15342, adult male, skin. Loc.: Palembang, Sumatra, [Indonesia], 09.viii.1918. Ex: MZB, 12.i.1950 (MZB 6066).

***Macropygiaphasianellamegala*** Siebers, 1929: 151.

= *Macropygiaemilianamegala* Siebers, 1929.

Holotype, RMNH.AVES.14024, adult male, skin. Loc.: Kangean Island, [Indonesia], v.1892. Leg.: A.G. Vorderman. Ex: MZB, 12.i.1950 (MZB 6068).

***Macropygianigrirostrismajor*** van Oort, 1908a: 174.

= *Macropygianigrirostris* Salvadori, 1876.

Syntype, RMNH.AVES.87765, adult male, skin. Loc.: Duke of York Island, [Papua New Guinea], 04.x.1880. [Leg.: Th. Kleinschmidt]. Ex: Museum Godeffroy, v.1882.

Syntype, RMNH.AVES.87766, adult male, skin. Loc.: New Britain, [Papua New Guinea], 26.xi.1880. [Leg.: Th. Kleinschmidt]. Ex: Museum Godeffroy, v.1882.

***Macropygiaphasianella*** Temminck, 1821: 129.

= *Macropygiaphasianellaphasianella* (Temminck, 1821).

Temminck (1821) based his description on a single specimen in the collection of the Linnean Society London from New South Wales. This holotype is now in the NHM (NHMUK 1863.7.6.15; Warren 1966: 226).

***Columbaruficeps*** Temminck, 1835: livr. 95, pl. 561.

= *Macropygiaruficepsruficeps* (Temminck, 1835).

Syntype, RMNH.AVES.87767, adult male, mounted skin. Loc.: Java, [Indonesia]. Leg.: S. Müller.

Syntype, RMNH.AVES.87768, adult male, skin. Loc.: Java, [Indonesia], [iv.1816–iii.1822]. Leg.: C.G.C. Reinwardt.

Syntype, RMNH.AVES.87803, adult, sex unknown, skeleton. Loc.: [West] Sumatra, [Indonesia], [vii.1833–1835]. Leg.: S. Müller (= *Macropygiaruficepssumatrana* Robinson & Kloss, 1919).

According to Schlegel (1873b: 110) RMNH.AVES.87767 is the depicted specimen on pl. 561.

Although not mentioned in the original description, Schifter et al. (2007: 153) listed two syntypes in the NMW (NMW 66076 and 66077), received in exchange with the RMNH in April 1830. One arrived in 1824 in the MfN, already documented as *Columbaruficeps* with reference to the ‘Planches Coloriées’. One was sent to Groningen in 1847.

Reinwardt visited Java several times between April 1816 and March 1822. Müller visited West Sumatra from July 1833 until late1835.

***ColumbaUnchall*** Wagler, 1827: no. 38.

***Columbaleptogrammica*** Temminck, 1835: livr. 95, pl. 560.

= *Macropygiaunchallunchall* (Wagler, 1827).

Syntype, RMNH.AVES.87770, adult male, mounted skin. Loc.: Java, [Indonesia], [iv.1816–iii.1822]. Leg.: C.G.C. Reinwardt.

Syntype only for *leptogrammica*, RMNH.AVES.87769, adult, sex unknown, mounted skin. Loc.: Java, [Indonesia], [vi.1826–ix.1827]. Leg.: H. Boie, [vi.1826–ix.1827].

According to Schlegel (1873b: 108) RMNH.AVES.87770 is the depicted specimen on pl. 560.

Reinwardt visited Java several times between April 1816 and March 1822. Boie visited Java between June 1826 and September 1827.

***ColumbaPicturata*** Temminck, 1813: page 315.

= *Nesoenaspicturatuspicturatus* (Temminck, 1813).

Syntype, RMNH.AVES.87888, adult, sex unknown, mounted skin. Loc.: Mauritius. Leg.: -.

Dowsett (pers. comm.) assumed that Réunion is the type locality instead of Mauritius, where the species had been introduced prior to 1813 when the specimen was described. The native distribution of *Nesoenaspicturatus* is on islands in the Indian Ocean (Seychelles, Amirantes, Aldabra, Glorieuse, and Comoro islands) with the nominate *picturatus* Temminck on Madagascar.

***Columbalophotes*** Temminck, 1822: livr. 24, pl. 142.

= *Ocyphapslophoteslophotes* (Temminck, 1822).

Lectotype, RMNH.AVES.87780, adult, sex unknown, mounted skin. Loc.: [Blue Mountains], Australia. Leg.: - [1821–1822].

The original description gives no indication about the number of type specimens. However, since Temminck (1822) described a single “male” there was probably only one specimen available at that time. As Van den Hoek Ostende et al. (1997: 100) referred to RMNH.AVES.87780 as holotype, this constitutes a lectotype designation.

This specimen must have been received in the RMNH between 1821 and 1822, as Temminck wrote that it had not arrived yet when he published a previous paper in the Transactions of the Linnean Society of London in 1821.

***Columbaerythroptera*** Gmelin, 1789: 254.

***Columbaleucophrys*** Wagler, 1829: 743.

= *Pampusanaerythropteraerythroptera* (Gmelin, 1789).

Possible syntype, RMNH.AVES.90752, adult male, mounted skin. Loc.: O. Taiti [= Tahiti]. Leg.: Voyage de Cook. Ex: Cab. Bullock [= Bullock Museum].

The origin, collector, and history and therefore the true identity of this specimen was and is still open to much debate. Up to now it is assumed to be the only surviving specimen out of at least four collected on Tahiti and Moorea between 17 August and 1 September 1773 and between 22 April and 14 May 1774 during Captain James Cook’s second voyage. It is and long has been considered one of the syntypes. However, only if it is one of the specimens collected during Capt. Cook’s second voyage, is it one of the syntypes.

Temminck (1811: 123) wrote that the specimen Knip illustrated in ‘Les Pigeons’ was from the private collection of Mr. Gevers from Rotterdam. Gevers’ collection was sold in 1787 (Gevers and Mensema 2000: 14), well before it was illustrated by Knip. One could therefore assume that Gevers’ specimen had become part of Temminck’s private collection which, in 1820, became part of the RMNH. Comparison of RMNH.AVES.90752 with the illustrated specimen does not, however, provide convincing evidence that the same individual is involved. Hence, RMNH.AVES.90752 might have come to the RMNH via a different route. One option is that it might have been purchased by Temminck at Bullock’s Auction in London in 1819, which did include part of Cook’s collection. No direct reference is given to this specimen in either the Sales Catalogue or in Temminck’s list of objects bought there, unless it refers to an ‘unknown pigeon’ on day 16, lot 3.

Another possibility currently examined by J. Jansen and J. Hume is that, because of a lack of evidence due to the absence of original labels as well as morphological differences with true *P.erythroptera*, RMNH.AVES.90752 might not originate from Tahiti and was not collected during Cook’s voyage or bought at Bullock’s auction. DNA analysis is scheduled to try to ascertain its identity and accept or reject historic or current theories.

***LeptoptilaHoedtii*** Schlegel, 1871a: 30.

= *Pampusanahoedtii* (Schlegel, 1871).

Syntype, RMNH.AVES.87732, adult male, skin. Loc.: Wetar Island, [Indonesia], 07.v.1866. Leg.: D.S. Hoedt.

Syntype, RMNH.AVES.87733, adult female, skin. Loc.: Wetar Island, [Indonesia], 09.v.1866. Leg.: D.S. Hoedt.

***Phlegoenasvitiensis*** Finsch, 1872: 50.

= *Pampusanastairi* (Gray, 1856).

Syntype, RMNH.AVES.87735, adult male, skin. Loc.: Vitu Levu, Fiji. Leg.: E.H. Gräffe. Ex: Museum Godeffroy, 1864.

Gräffe (1868) visited Viti Levu once prior to 1864, sometime during 1862. He described (1868: 11) how he received during this trip a living pair of *Peristeraerythroptera* in Vaitop upstream the Rewa River. The male of this pair could well be RMNH.AVES.87735.

***Columbaxanthonura*** Temminck, 1823: pl. 190.

= *Pampusanaxanthonura* (Temminck, 1823).

Temminck (1823) based this name on two specimens in the MNHN collected by Quoy and Gaimard (MNHN C.G.2003-2661 and C.G.2003-2662; Voisin et al. 2005: 862).

***Columbadenisea*** Temminck, 1830: livr. 86 [= 85], pl. 502.

= *Patagioenasaraucana* (Lesson & Garnot, 1827).

Holotype, RMNH.AVES.87632, adult, sex unknown, mounted skin. Loc.: Chile. Leg.: -.

Contrary to Schlegel (1873b: 68) and Van den Hoek Ostende et al. (1997: 85), who listed two syntypes, Temminck (1830) referred to a single specimen from Chile in the RMNH which is the holotype by monotypy. It was identified based on comparison with the illustrated specimen.

According to Dickinson (2001: 46) this name was published in livraison 85, not 86 as printed in the footnote of the text and mentioned by Sherborn (1898: 488).

***ColumbaRufina*** Temminck, 1809: livr. 6, p. 59, pl. 24.

= *Patagioenascayennensiscayennensis* Bonnaterre, 1792.

Temminck (1809) based his description on a specimen in the MNHN where it is confirmed by Voisin et al. (2005: 846; MNHN C.G. 2002-529) and specimens in several other collections. One was sent to Leuven in 1822.

For dating of this name and those of other pigeons and doves by Temminck in the years 1808–1811, see Dickinson et al. (2010).

***ColumbaGymnophtalmos*** Temminck, 1809: livr. 5, p. 48, pl. 18.

= *Patagioenascorensis* (Jacquin, 1784).

Temminck (1809) based his description on a male in the MNHN where it is confirmed by Voisin et al. (2005: 846; MNHN C.G.2002-531) and a female in the collection of Raye van Breukelerwaert. The latter was sold at the auction of this collection for 2 guilders and 50 cents (Catalogue: 52). Its current whereabouts are unknown.

***Columbafasciatavioscae*** Brewster, 1888: 86.

= *Patagioenasfasciatavioscae* (Brewster, 1888).

Syntype, RMNH.AVES.87639, adult female, skin. Loc.: Piercio Ranch, Lower California, United States, 5.vii.1887. Leg.: A. Frazar. Ex: MCZ, 1902.

***Columbaleucocapilla*** Temminck, 1807: 142, no. 777.

= *Patagioenasleucocephala* (Linnaeus, 1758).

Syntype, RMNH.AVES.90966, adult male, relaxed mount. Loc.: Trinidad. Ex: Cabinet Temminck.

Temminck (1807) did not indicate the number of specimens available to him but referred to male(s) only.

***ColumbaMaculosa*** Temminck, 1813a: 113.

= *Patagioenasmaculosamaculosa* (Temminck, 1813).

Temminck (1813a) based his description on d’Azara and did not refer to any specimens.

***Chloroenasfallax*** Schlegel, 1873b: 80.

= *Patagioenasmaculosamaculosa* (Temminck, 1813).

Syntype, RMNH.AVES.87641, adult female, mounted skin. Loc.: Rio Negro, Argentina, vi.1871. Ex: G.A. Frank, 1873.

Syntype, RMNH.AVES.89418, adult, sex unknown, mounted skin. Loc.: “Mexico”. Leg.: -.

***ColumbaPicazuro*** Temminck, 1813a: 111.

= *Patagioenaspicazuropicazuro* (Temminck, 1813).

Temminck (1813a) based his description on d’Azara and did not refer to any specimens.

***ColumbaPortoricensis*** Temminck, 1809: 41, pl. 15.

= *Patagioenassquamosa* (Bonnaterre, 1792).

Lectotype, RMNH.AVES.90967, adult, sex unknown, relaxed mount. Loc.: Antilles “Haiti”. Ex: Cabinet Temminck.

See Stresemann (1953b: 103) for dating of Temminck and Knip’s ‘Les Pigeons’ (1808–1811). According to the original description other type specimens are in the MNHN. Voisin et al. (2005: 843) confirmed two paralectotypes in the MNHN (MNHN 2002-540 and 2002-541) collected by Maugé in Puerto Rico. Jansen and Fuchs (2019: 46) listed additional specimens in the NMS (NMS 1819.20.1.72) and NMW (NMW 44588).

Schlegel (1873b: 68) selected the lectotype.

***ColumbaVinacea*** Temminck, 1809: livr. 9, p. 87, pl. 41 (nec Gmelin, 1789).

= *Patagioenassubvinaceapurpureotincta* (Ridgway, 1888).

Temminck (1809) reused a specific epithet for this new species already occupied by *Columbavinacea* Gmelin, 1789 (= *Streptopeliavinacea*). Temminck based his description on a single specimen in MNHN which is still present (Voisin et al. 2005: 847; MNHN C.G. 2002-553).

For dating of this name and those of other pigeons and doves by Temminck in the years 1808–1811, see Dickinson et al. (2010).

***Columbaleucotis*** Temminck, 1823: livr. 32, pl. 189.

= *Phapitreronleucotisleucotis* (Temminck, 1823).

The holotype is in the MNHN (MNHN 2002-139; Voisin et al. 2004: 113).

***ColumbaElegans*** Temminck, 1809: livr. 6, p. 56, pl. 22.

= *Phapseleganselegans* (Temminck, 1809).

Temminck (1809) based his description on two specimens in the MNHN collected during the Baudin expedition where they still are (Voisin et al. 2005: 858; MNHN C.G.2003-2671 and C.G.2003-2656) and a specimen in London in the collection of J. Banks. Van den Hoek Ostende et al. (1997: 101) listed a syntype in the RMNH collection (RMNH.AVES.87782); this must be an error.

***Peristerahistrionica*** Gould, 1841b: pl. 66.

= *Phapshistrionica* (Gould, 1841).

Syntype, RMNH.AVES.87751, adult male, mounted skin. Loc.: [Namori Plains], Australia, [xii.1839]. Leg.: J. Gould.

Syntype, RMNH.AVES.87752, adult female, mounted skin. Loc.: [Namori Plains], Australia, [xii.1839]. Leg.: J. Gould.

These are two of the 12 specimens Gould collected on the Namori Plains in December 1839. RMNH.AVES.87752 is labelled “Verreaux, 1867”, which cannot be correct as “Gould” is written under the stand in Temminck’s handwriting. As Temminck died in 1858, the specimen could not have been purchased from Verreaux in 1867.

***Ptilopusbernsteinii*** Schlegel, 1863c: 59.

= *Ptilinopusbernsteiniibernsteinii* Schlegel, 1863.

Holotype for *bernsteinii*, syntype for *ochrogaster*, RMNH.AVES.87771, adult female, mounted skin. Loc.: [Gunung Sibela], Batjan, [Indonesia], 19.i.1861. Leg.: H.A. Bernstein.

RMNH.AVES.87771 is the holotype by monotypy for *bernsteinii*. Schlegel (1863c) based his description on this single specimen sent by Bernstein.

***Ptilopusochrogaster*** Bernstein, 1864: 86.

= *Ptilinopusbernsteiniibernsteinii* Schlegel, 1863.

Syntype for *ochrogaster*, holotype for *bernsteinii*, RMNH.AVES.87771, adult female, mounted skin. Loc.: Batjan, [Indonesia], 19.i.1861. Leg.: H.A. Bernstein.

Syntype, RMNH.AVES.87772, adult male, mounted skin. Loc.: Kaou, Halmahera, [Indonesia], 27.xi.1861. Leg.: H.A. Bernstein.

Syntype, RMNH.AVES.87773, adult male, mounted skin. Loc.: Obi-Lattou [= Obilatu], [Indonesia], 25.viii.1862. Leg.: H.A. Bernstein. (= *Ptilinopusbernsteiniimicrus* (Jany, 1955)).

Syntype, RMNH.AVES.87774, adult male, mounted skin. Loc.: Dodingo, Halmahera, [Indonesia], 22.x.1862. Leg.: H.A. Bernstein.

Syntype, RMNH.AVES.87775, adult male, mounted skin. Loc.: Weda, Halmahera, [Indonesia], 10.iii.1863. Leg.: H.A. Bernstein.

Syntype, RMNH.AVES.87776, adult female, mounted skin. Loc.: Weda, Halmahera, [Indonesia], 11.vi.1863. Leg.: H.A. Bernstein.

Syntype, RMNH.AVES.87777, adult female, mounted skin. Loc.: Ternate, [Indonesia], 14.viii.1863. Leg.: H.A. Bernstein.

Syntype, RMNH.AVES.87778, adult male, mounted skin. Loc.: Weda, Halmahera, [Indonesia], 01.xi.1864. Leg.: H.A. Bernstein.

***PtilopuscinctusFlorensis*** Schlegel, 1871a: 20 (nomen novum).

= *Ptilinopuscinctusalbocinctus* Wallace, 1864.

Schlegel (1871a) described *Ptilopuscinctus Florensis* as an unnecessary nomen novum for *Ptilopusalbocinctus* Wallace, 1864.

***ColumbaCincta*** Temminck, 1809: livr. 6, p. 58, pl. 23.

= *Ptilinopuscinctuscinctus* (Temminck, 1809).

Temminck (1809) based his description on a single specimen in his own cabinet, which could not be found in the RMNH. One was sent to Berlin in March 1833 and one to Frankfurt in March 1835 (J. Jansen, pers. comm., 2022). As Temminck’s description is based on a single specimen, one of the two is the holotype.

For dating of this name and those of other pigeons and doves by Temminck in the years 1808–1811, see Dickinson et al. (2010).

***PtilopuscinctusLettiensis*** Schlegel, 1871a: 20.

= *Ptilinopuscinctuslettiensis* Schlegel, 1871.

Syntype, RMNH.AVES.87754, adult male, mounted skin. Loc.: Letti, [Indonesia], 30.v.1866. Leg.: D.S. Hoedt.

Syntype, RMNH.AVES.87755, adult female, mounted skin. Loc.: Letti, [Indonesia], 05.vii.1866. Leg.: D.S. Hoedt.

Syntype, RMNH.AVES.213745, immature male, relaxed mount. Loc.: Letti, [Indonesia], v.1866. Leg.: D.S. Hoedt.

Syntype, RMNH.AVES.213746, immature female, relaxed mount. Loc.: Letti, [Indonesia], v.1866. Leg.: D.S. Hoedt.

Van den Hoek Ostende et al. (1997: 97) must have overseen the two immature specimens, already listed in Schlegel (1873b: 35).

***Columbahyogastra*** “Reinwardt” Temminck, 1824: livr. 43, pl. 252.

= *Ptilinopushyogastrus* (Temminck, 1824).

Syntype, RMNH.AVES.87786, adult male, mounted skin. Loc.: Gilolo, [Halmahera], [Indonesia]. Leg.: ?E.A. Forsten.

Syntype, RMNH.AVES.87787, adult female, mounted skin. Loc.: Gilolo, [Halmahera], [Indonesia]. Leg.: ?E.A. Forsten.

The stand gives Forsten as collector in Temminck’s handwriting, which must be erroneous since Forsten arrived in Indonesia after the species was published. The specimens were probably collected by Reinwardt who visited Halmahera briefly in 1821. A similar mix-up is found in a type specimen of *Columbaperspicillata* Temminck, 1824, which was presumably collected by Reinwardt and is also labelled ‘Forsten’. In addition to this Temminck wrote on the stand of RMNH.AVES.87786 “ind. type”.

***Ptilopusinsolitus*** Schlegel, 1863c: 61, pl. Vogels 3, fig. 3.

= *Ptilinopusinsolitusinsolitus* Schlegel, 1863.

Holotype, RMNH.AVES.87788, adult, sex unknown, skin. Loc.: New Caledonia [error]. Leg.: - [1862].

Holotype by monotypy. The bird was acquired in 1862, the collector is not known. The tail and primaries are lacking. This was already mentioned by Schlegel in the description and most probably the reason why the bird in the plate is partly hidden behind a plant. The type locality was given as New Ireland by Hartert (1924: 197).

***Ptilopushumeralisiobiensis*** Schlegel, 1873b: 16.

= *Ptilinopusiozonusiobiensis* Schlegel, 1873.

Lectotype, RMNH.AVES.87792, adult male, skin. Loc.: Jobi, [Indonesia], 28.iv.1869. Leg.: C.B.H. von Rosenberg.

Paralectotypes, RMNH.AVES.87788 (= *Ptilinopusinsolitusinsolitus* Schlegel, 1863), 87789–87791.

In his description Schlegel (1873b) explicitly includes the type of *Ptilopusinsolitus* Schlegel, 1863 (RMNH.AVES.87788) in the type series. Since this name has priority, Schlegel should have placed all specimens from Jobi in this taxon. Instead, he incorrectly introduced a new name. Since *insolitus* is a different taxon, the name *P.humeralisiobiensis* is available for the race from Jobi. Quaisser and Dekker (2008: 412) designated the lectotype.

***PtilopusHugoniana*** Schlegel, 1863c: 60, pl. 3 fig. 2.

= *Ptilinopusleclancherileclancheri* (Bonaparte, 1855).

Lectotype, RMNH.AVES.87756, adult, sex unknown, skin. Loc.: Luzon, Philippines. Leg.: H. Gevers, 1862.

Paralectotype, RMNH.AVES.87793.

Schlegel (1873b: 36) selected the lectotype.

***Columbaluteovirens*** Hombron & Jacquinot, 1841: 315.

= *Ptilinopusluteovirens* (Hombron & Jacquinot, 1841).

Syntype, RMNH.AVES.87628, adult male, skin. Loc.: Vanua Valavo [= Ovalau], [Fiji], [x.1838]. Leg.: [J.B. Hombron and H. Jacquinot, Expedition of L’Astrolabe and La Zelée.] Ex: MNHN.

According to Voisin et al. (2004: 118) two other syntypes are in the MNHN (MNHN C.G.2002-129 and C.G.2002-130).

***Columbamagnifica*** Temminck, 1821: 125.

= *Ptilinopusmagnificusmagnificus* (Temminck, 1821).

Holotype, RMNH.AVES.87779, adult, sex unknown, mounted skin. Loc.: Red Point, S.E. Australia. Ex: Linnean Society London, A. MacLeay.

Temminck (1821) wrote that he received a specimen from Alexander MacLeay during his visit to London in 1819. This is the only specimen mentioned in his description, so we consider it to be a holotype by monotypy. The specimen listed by Warren (1966: 173; NHMUK 1863.7.6.11) as holotype is therefore not part of the type series. The year indicated on the label is 1868. This must be a mistake and probably indicates the year the specimen was remounted (G.F. Mees, in litt.).

***Jotreronchrysorrhoa*** Salvadori, 1875a: 671.

***Ptilinopussulaënsis*** Brüggemann, 1876: 81.

= *Ptilinopusmelanospiluschrysorrhous* (Salvadori, 1875).

Syntype, RMNH.AVES.87794, adult male, mounted skin. Loc.: Wahaai, Ceram, [Indonesia], 1862. Leg.: J.C.B. Bernelot Moens.

Syntype, RMNH.AVES.87795, adult female, mounted skin. Loc.: Ceram, [Indonesia], [x.1859–vi.1860]. Leg.: A.R. Wallace. Ex: Frank, 1862.

Syntype, RMNH.AVES.87796, adult male, mounted skin. Loc.: Sula Bessie, [Sanana], [Indonesia], i.1864. Leg.: H.A. Bernstein.

Syntype, RMNH.AVES.87797, adult male, mounted skin. Loc.: Sula Bessie, [Sanana], [Indonesia], i.1864. Leg.: H.A. Bernstein.

Syntype, RMNH.AVES.87798, adult male, mounted skin. Loc.: Sula Bessie, [Sanana], [Indonesia], i.1864. Leg.: H.A. Bernstein.

Syntype, RMNH.AVES.87799, adult male, mounted skin. Loc.: Sula Bessie, [Sanana], [Indonesia], i.1864. Leg.: H.A. Bernstein.

Syntype, RMNH.AVES.87800, immature female, mounted skin. Loc.: Sula Bessie, [Sanana], [Indonesia], i.1864. Leg.: H.A. Bernstein.

Syntype, RMNH.AVES.87801, immature female, mounted skin. Loc.: Sula Bessie, [Sanana], [Indonesia], i.1864. Leg.: H.A. Bernstein.

Syntype, RMNH.AVES.87802, adult male, mounted skin. Loc.: Sula Mangola, [Indonesia], ii.1864. Leg.: H.A. Bernstein.

Syntype, RMNH.AVES.87923, adult male, mounted skin. Loc.: Sula Mangola, [Indonesia], ii.1864. Leg.: H.A. Bernstein.

Syntype, RMNH.AVES.87804, adult male, mounted skin. Loc.: Sula Mangola, [Indonesia], ii.1864. Leg.: H.A. Bernstein.

Syntype, RMNH.AVES.87805, adult male, mounted skin. Loc.: Sula Bessie, [Sanana], [Indonesia], 15.xi.1864. Leg.: D.S. Hoedt.

Syntype, RMNH.AVES.87806, adult female, mounted skin. Loc.: Sula Bessie, [Sanana], [Indonesia], 16.xi.1864. Leg.: D.S. Hoedt.

Syntype, RMNH.AVES.87807, adult female, mounted skin. Loc.: Sula Bessie, [Sanana], [Indonesia], 17.xi.1864. Leg.: D.S. Hoedt.

Syntype, RMNH.AVES.87808, adult male, mounted skin. Loc.: Sula Bessie, [Sanana], [Indonesia], 19.xi.1864. Leg.: D.S. Hoedt.

The collecting date of RMNH.AVES.87808 is not clear. According to the label it was collected on 19.xi.1864, but the information on the stand states that it was collected on 19.xii.1864.

Wallace visited Ceram [= Seram] twice between October 1859 and June 1860 (Baker 2001).

***Jotreronmelanauchen*** Salvadori, 1875a: 671.

= *Ptilinopusmelanospilusmelanauchen* (Salvadori, 1875).

Holotype, RMNH.AVES.87809, adult male, mounted skin. Loc.: Larantoeka, Flores, [Indonesia], 13.iv.1862. Leg.: J. Semmelink.

Holotype by monotypy. The description by Salvadori (1875a) was based on Schlegel (1873b), where only RMNH.AVES.87809 was listed.

***Jotreronmelanospila*** Salvadori, 1875a: 670.

= *Ptilinopusmelanospilusmelanospilus* (Salvadori, 1875).

Syntype, RMNH.AVES.214772, adult male, mounted skin. Loc.: Menado, Celebes [= Sulawesi], [Indonesia]. Leg.: J.G.F. Riedel, 1870.

Syntype, RMNH.AVES.214773, adult female, mounted skin. Loc.: between Katapara and Alep, Celebes [= Sulawesi], [Indonesia], v.1840. Leg.: E.A. Forsten.

Syntype, RMNH.AVES.214774, adult male, mounted skin. Loc.: Soumalatta, Celebes [= Sulawesi], [Indonesia], 2.xi.1863. Leg.: C.B.H. von Rosenberg.

Syntype, RMNH.AVES.214775, adult male, mounted skin. Loc.: Kwandang, Celebes [= Sulawesi], [Indonesia], 28.x.1863. Leg.: C.B.H. von Rosenberg.

Syntype, RMNH.AVES.214776, adult female, mounted skin. Loc.: Modelido, Celebes [= Sulawesi], [Indonesia], v.1863. Leg.: C.B.H. von Rosenberg.

Syntype, RMNH.AVES.214777, adult female, mounted skin. Loc.: Modelido, Celebes [= Sulawesi], [Indonesia], v. 1863. Leg.: C.B.H. von Rosenberg.

Syntype, RMNH.AVES.214778, adult male, mounted skin. Loc.: Bone, Celebes [= Sulawesi], [Indonesia] 27.xi.1863. Leg.: C.B.H. von Rosenberg.

Syntype, RMNH.AVES.214779, adult female, mounted skin. Loc.: Bone, Celebes [= Sulawesi], [Indonesia], 18.xi.1863. Leg.: C.B.H. von Rosenberg.

Salvadori (1875a) described *melanospilus* based on six specimens collected by Bruijn and Beccari, but in the description he also referred to Schlegel (1873b: 29). Therefore, the specimens listed by Schlegel with the description are part of the type series.

***Jotreronxanthorrhoa*** Salvadori, 1875a: 671.

= *Ptilinopusmelanospilusxanthorrhous* (Salvadori, 1875).

Syntype, RMNH.AVES.87810, adult male, mounted skin. Loc.: Sanghir, [Indonesia], 24.x.1864. Leg.: C.B.H. von Rosenberg.

Syntype, RMNH.AVES.87811, adult male, mounted skin. Loc.: Sanghir, [Indonesia], 28.x.1864. Leg.: C.B.H. von Rosenberg.

Syntype, RMNH.AVES.87812, adult female, mounted skin. Loc.: Sanghir, [Indonesia], 28.x.1864. Leg.: C.B.H. von Rosenberg.

Syntype, RMNH.AVES.87813, adult male, mounted skin. Loc.: Sanghir, [Indonesia], 02.i.1866. Leg.: D.S. Hoedt.

Syntype, RMNH.AVES.87814, adult female, mounted skin. Loc.: Sanghir, [Indonesia], 02.i.1866. Leg.: D.S. Hoedt.

Syntype, RMNH.AVES.87815, adult male, mounted skin. Loc.: Sanghir, [Indonesia], 29.i.1866. Leg.: D.S. Hoedt.

Syntype, RMNH.AVES.87816, adult male, mounted skin. Loc.: Siao, Sanghir, [Indonesia], 24.x.1865. Leg.: D.S. Hoedt.

Syntype, RMNH.AVES.87817, adult male, mounted skin. Loc.: Siao, Sanghir, [Indonesia], 24.x.1865. Leg.: D.S. Hoedt.

Syntype, RMNH.AVES.87818, adult female, mounted skin. Loc.: Siao, Sanghir, [Indonesia], 28.x.1865. Leg.: D.S. Hoedt.

Syntype, RMNH.AVES.87819, immature female, mounted skin. Loc.: Siao, Sanghir, [Indonesia], 28.x.1865. Leg.: D.S. Hoedt.

Syntype, RMNH.AVES.87820, adult female, mounted skin. Loc.: Siao, Sanghir, [Indonesia], 04.xi.1865. Leg.: D.S. Hoedt.

Syntype, RMNH.AVES.87821, adult male, mounted skin. Loc.: Siao, Sanghir, [Indonesia]. Ex: L.D.H.A. Renesse van Duijvenbode, 1866.

On the stand of RMNH.AVES.87813 and RMNH.AVES.87815 the date 1866 is written while the label gives 1865 as the year of collection. RMNH.AVES.87818 and RMNH.AVES.87819 give 1865 on the stand and 1864 on the label (later changed into 1865).

***Columbamonacha*** Temminck, 1824: livr. 43, pl. 253.

= *Ptilinopusmonacha* (Temminck, 1824).

Holotype, RMNH.AVES.87830, adult, sex unknown, mounted skin. Loc.: Ternate, [Indonesia]. Leg.: C.G.C. Reinwardt [viii–ix.1821].

Holotype by monotypy. According to Temminck (1824) this species is from Celebes. This is an error since *P.monacha* is restricted to Halmahera and surrounding islands. Schlegel (1873b: 13) corrected the collecting location to Ternate.

Reinwardt visited Ternate (and very briefly Halmahera) twice between 15 August 1821 and 4 September 1821.

***Columbanaina*** Temminck, 1835: livr. 95, pl. 565.

= *Ptilinopusnainus* (Temminck, 1835).

Lectotype, RMNH.AVES.87831, adult male, mounted skin. Loc.: Lobo Bay, [Papua], [Indonesia], [4.vii -29.viii.]1828. Leg.: S. Müller.

See Mees (1982) for year of publication. The original description gives no indication about the number of type specimens other than Temminck had not yet seen the female. However, Van den Hoek-Ostende et al. (1997: 105) erroneously listed it as holotype, which constitutes a lectotype designation.

***Ptilopusornatus*** Schlegel, 1871b: 52.

= *Ptilinopusornatusornatus* Schlegel, 1871.

Syntype, RMNH.AVES.87832, adult male, mounted skin. Loc.: Haddam, [Papua], [Indonesia], 09.iv.1870. Leg.: C.B.H. von Rosenberg.

Syntype, RMNH.AVES.87833, adult female, mounted skin. Loc.: Haddam, [Papua], [Indonesia], 10.iv.1870. Leg.: C.B.H. von Rosenberg.

Syntype, RMNH.AVES.87834, adult male, mounted skin. Loc.: Haddam, [Papua], [Indonesia], 13.iv.1870. Leg.: C.B.H. von Rosenberg.

Syntype, RMNH.AVES.87835, adult male, mounted skin. Loc.: Haddam, [Papua], [Indonesia], 19.iv.1870. Leg.: C.B.H. von Rosenberg.

***Columbaperlata*** Temminck, 1835: livr. 94, pl. 559.

= *Ptilinopusperlatusperlatus* (Temminck, 1835).

Holotype, RMNH.AVES.87836, adult male, mounted skin. Loc.: Lobo Bay, [Papua], [Indonesia], [4.vii–29.viii.] 1828. Leg.: S. Müller.

Holotype by monotypy. See Mees (1982) for the year of publication.

***Ptilopuszonurus*** Salvadori, 1876: 197.

= *Ptilinopusperlatuszonurus* Salvadori, 1876.

Syntype, RMNH.AVES.87837, adult female, mounted skin. Loc.: Wonumbai [= Sungai Manumbai], Aru Islands, [Indonesia], 15.v.1865. Leg.: C.B.H. von Rosenberg.

Syntype, RMNH.AVES.87838, adult female, mounted skin. Loc.: Wonumbai [= Sungai Manumbai], Aru Islands, [Indonesia], 25.v.1865. Leg.: C.B.H. von Rosenberg.

***Columbaporphyracea*** Temminck, 1821: 130.

***ColumbaForsteri*** Desmarest, 1826: 340.

***Columbaviridissima*** Temminck, 1835: text for pl. 254 (2^nd^ ed. livr. 95).

= *Ptilinopusporphyraceusporphyraceus* (Temminck, 1821).

Syntype for *porphyracea* and *Forsteri*, lectotype for *viridissima*, RMNH.AVES.213951, adult male, relaxed mount. Loc.: Tonga Tabu [= Tongatapu]. Ex: [MNHN?].

According to Jansen (2018: 284), RMNH.AVES.213951 is one of the two doves collected by G. Bass in September or October 1802 and brought to Paris by the Baudin expedition.

Desmarest (1826) based his *Forsteri* on one of the unpublished plates by Forster, but also on the description by Temminck (1821) of *porphyracea*. According to Desmarest this new name is necessary because *porphyracea* was preoccupied by *Columbaporphyrea* Temminck, 1822.

Schlegel (1873b: 8) selected RMNH.AVES.213951 as lectotype of *Columbaviridissima*.

***Columbaporphyrea*** “Reinwardt” Temminck, 1822: livr. 18, pl. 106.

***Columbaroseicollis*** Wagler, 1827: no. 27 (nomen novum).

= *Ptilinopusporphyreus* (Temminck, 1822).

Holotype, RMNH 87757, adult male, mounted skin. Loc.: Java, [Indonesia], [iv.1816–iii.1822]. Leg.: C.G.C. Reinwardt.

Following Dickinson et al. (2022) (see also Introduction of this catalogue under “What is new in this 2023 version”: 14) we have selected the holotype based on the plate since the text that appeared on 22 May 1824 is irrelevant regarding type status. Any listing of syntypes for this name in Van den Hoek Ostende et al. (1997: 97) or any other type catalogue is erroneous. RMNH.AVES.87758–87761 listed in Van den Hoek Ostende et al. (1997: 97) and NMW 66002 (Schifter et al. 2007: 146) have therefore no type status. One collected by Reinwardt was sent to the NMW in March 1822 (1822.LXXX.42) and another arrived there in April 1830. One was sent to the MfN in May 1827 and one went to the SMF in March 1835.

Reinwardt visited Java several times between April 1816 and March 1822.

***Columbapulchella*** Temminck, 1835: livr. 95, pl. 564.

= *Ptilinopuspulchellus* (Temminck, 1835).

Syntype, RMNH.AVES.87839, adult male, mounted skin. Loc.: Lobo Bay, [Papua], [Indonesia], [4.vii–29.viii.] 1828. Leg.: S. Müller.

Syntype, RMNH.AVES.87840, adult male, mounted skin. Loc.: Lobo Bay, [Papua], [Indonesia], [4.vii–29.viii.] 1828. Leg.: S. Müller.

***Ptilopusneglectus*** Schlegel, 1873b: 7.

= *Ptilinopusrarotongensisrarotongensis* Hartlaub & Finsch, 1871.

Holotype, RMNH.AVES.87841, adult, sex unknown, relaxed mount. Loc.: Océanie [= Rarotonga Island], [Cook Islands]. Leg.: -.

***ColumbaXanthogaster*** Wagler, 1827: no. 29 [237].

= *Ptilinopusreginaxanthogaster* (Wagler, 1827).

Syntype, RMNH.AVES.87842, adult male, skin. Loc.: Banda, [Indonesia], [18.v.-25.vi.1821]. Leg.: [C.G.C. Reinwardt].

Wagler (1827) based this name on pl. 254 and the first edition of the accompanying text in Temminck (1820–1839). Therefore, the other three specimens in Temminck’s description are also part of the type series. The whereabouts of these two syntypes is unknown.

Temminck described this specimen as a female collected by Reinwardt in Celebes, which was later corrected to a male from Banda by Schlegel (1873b: 11).

Reinwardt visited the Banda Archipelago from 18 May to 25 June 1821.

***Columbadiademata*** Temminck, 1835: text for 254 (2^nd^ ed. livr. 95).

= *Ptilinopusreginaxanthogaster* (Wagler, 1827).

Syntype, RMNH.AVES.87842, adult male, skin. Loc.: Banda, [Indonesia], [18.v.-25.vi.1821]. Leg.: [C.G.C. Reinwardt].

Syntype, RMNH.AVES.87843, adult male, skin. Loc.: Banda Neira, [Indonesia], [25–29.iv.] 1828. Leg.: S. Müller.

Temminck (1835) referred to six specimens: three collected by Reinwardt from Celebes [error for Banda] and three by Müller and Macklot from Banda. The whereabouts of the other four syntypes is unknown. The original text accompanying pl. 254 was part of livr. 43 (1824). However, the description of *diademata* was published in a corrected new issue of this text in livr. 95 (1835).

Müller stayed on Banda-Neira from 25 to 29 April 1828. In the report of his visit, he mentioned collecting this new species (Müller 1841: 119).

***Ptilopusaurantiventris*** Von Rosenberg, 1866: 144.

= *Ptilinopusreginaxanthogaster* (Wagler, 1827).

Syntype, RMNH.AVES.87844, immature male, mounted skin. Loc.: Little Key, [Indonesia], 14.viii.1865. Leg.: C.B.H. von Rosenberg.

Syntype, RMNH.AVES.87845, immature male, mounted skin. Loc.: Little Key, [Indonesia], 24.viii.1865. Leg.: C.B.H. von Rosenberg.

Syntype, RMNH.AVES.87846, adult male, mounted skin. Loc.: Koor, Key Islands, [Indonesia], 02.ix.1865. Leg.: C.B.H. von Rosenberg.

Syntype, RMNH.AVES.87847, adult female, mounted skin. Loc.: Koor, Key Islands, [Indonesia], 02.ix.1865. Leg.: C.B.H. von Rosenberg.

Syntype, RMNH.AVES.87848, adult male, skin. Loc.: Great Key, [Indonesia], 05.vi.1865. Leg.: C.B.H. von Rosenberg.

Syntype, RMNH.AVES.87849, adult female, skin. Loc.: Great Key, [Indonesia], 02.viii.1865. Leg.: C.B.H. von Rosenberg.

Syntype, RMNH.AVES.87850, immature male, skin. Loc.: Great Key, [Indonesia], 02.viii.1865. Leg.: C.B.H. von Rosenberg.

Syntype, RMNH.AVES.87851, immature female, skin. Loc.: Great Key, [Indonesia], 03.viii.1865. Leg.: C.B.H. von Rosenberg.

Syntype, RMNH.AVES.87852, adult female, skin. Loc.: Little Key, [Indonesia], 18.viii.1865. Leg.: C.B.H. von Rosenberg.

Syntype, RMNH.AVES.87853, immature male, skin. Loc.: Little Key, [Indonesia], viii.1865. Leg.: C.B.H. von Rosenberg.

Although given as 1867 in Van den Hoek Ostende et al. (1997: 106), volume 29 of the ‘Natuurkundig Tijdschrift voor Nederlandsch Indië’ was already published in 1866 (Mees 1980). Later in the same year, Schlegel (1866f: 346) listed *aurantiventris*, with reference to Von Rosenberg as the original author, in synonymy of *P.diadematus* (Temminck, 1835).

***PtilopusMiquelii*** Schlegel, 1871a: 22.

= *Ptilinopusrivolimiquelii* Schlegel, 1871.

Syntype, RMNH.AVES.87822, adult female, mounted skin. Loc.: Jobi, [Indonesia], 29.iv.1869. Leg.: C.B.H. von Rosenberg.

Syntype, RMNH.AVES.87823, adult female, mounted skin. Loc.: Jobi, [Indonesia], 03.v.1869. Leg.: C.B.H. von Rosenberg.

Syntype, RMNH.AVES.87824, adult male, mounted skin. Loc.: Jobi, [Indonesia], 03.v.1869. Leg.: C.B.H. von Rosenberg.

Syntype, RMNH.AVES.87825, adult male, mounted skin. Loc.: Jobi, [Indonesia], 03.v.1869. Leg.: C.B.H. von Rosenberg.

Syntype, RMNH.AVES.87826, adult male, mounted skin. Loc.: Miosnom [= Mios Num], [Indonesia], 08.v.1869. Leg.: C.B.H. von Rosenberg.

Syntype, RMNH.AVES.87827, adult male, mounted skin. Loc.: Miosnom [= Mios Num], [Indonesia], 10.v.1869. Leg.: C.B.H. von Rosenberg.

Syntype, RMNH.AVES.87828, adult female, mounted skin. Loc.: Miosnom [= Mios Num], [Indonesia], 11.v.1869. Leg.: C.B.H. von Rosenberg.

Syntype, RMNH.AVES.87829, adult male, mounted skin. Loc.: Miosnom [= Mios Num], [Indonesia], 14.v.1869. Leg.: C.B.H. von Rosenberg.

***Columbaroseicapilla*** Lesson, 1831: 472.

= *Ptilinopusroseicapilla* (Lesson, 1831).

Syntype, RMNH.AVES.87854, adult male, relaxed mount. Loc.: Guam, Marianne Islands. Leg.:.

Syntype, RMNH.AVES.87855, adult female, relaxed mount. Loc.: Guam, Marianne Islands. Leg.:. Ex: MNHN.

Two other syntypes are in the MNHN (MNHN C.G.2002-123 and C.G.2002-124; Voisin et al. 2004: 115).

The original data was lost when these specimens were removed from their stands. The more recent labels give “Guam, “Lesson” Schleg.” and additionally “Mus. Paris” for RMNH.AVES.87855. It is unclear what is meant by “Lesson”. It could be a reference to the author or collector. If Lesson is the collector, then these specimens are not part of the type series as Lesson (1831) specifically refers to Freycinet (‘Uranie’ expedition) for the type series. However, we expect that Lesson would have mentioned any specimens collected by himself, so we assume “Lesson” on the more recent labels refers to the author, not the collector, of *roseicapilla*.

***Ptilopusspeciosus*** “von Rosenberg” Schlegel, 1871a: 22.

= *Ptilinopussolomonensisspeciosus* Schlegel, 1871.

Syntype, RMNH.AVES.87856, adult male, mounted skin. Loc.: Mefor [= Numfor], [Indonesia], 30.i.1869. Leg.: C.B.H. von Rosenberg.

Syntype, RMNH.AVES.87857, adult male, mounted skin. Loc.: Mefor [= Numfor], [Indonesia], 30.i.1869. Leg.: C.B.H. von Rosenberg.

Syntype, RMNH.AVES.87858, adult male, mounted skin. Loc.: Mefor [= Numfor] I[ndonesia], 31.i.1869. Leg.: C.B.H. von Rosenberg.

Syntype, RMNH.AVES.87859, adult female, mounted skin. Loc.: Mefor [= Numfor], [Indonesia], 31.i.1869. Leg.: C.B.H. von Rosenberg.

Syntype, RMNH.AVES.87860, adult male, mounted skin. Loc.: Mefor [= Numfor], [Indonesia], 01.ii.1869. Leg.: C.B.H. von Rosenberg.

Syntype, RMNH.AVES.87861, adult male, mounted skin. Loc.: Mefor [= Numfor], [Indonesia], 01.iii.1869. Leg.: C.B.H. von Rosenberg.

Syntype, RMNH.AVES.87862, adult male, mounted skin. Loc.: Soëk [= Biak], [Indonesia], 15.iii.1869. Leg.: C.B.H. von Rosenberg.

Syntype, RMNH.AVES.87863, adult male, mounted skin. Loc.: Soëk [= Biak], [Indonesia], 23.iii.1869. Leg.: C.B.H. von Rosenberg.

Syntype, RMNH.AVES.87864, adult female, mounted skin. Loc.: Soëk [= Biak], [Indonesia], 23.iii.1869. Leg.: C.B.H. von Rosenberg.

Syntype, RMNH.AVES.87865, adult female, mounted skin. Loc.: Soëk [= Biak, Indonesia], 23.iii.1869. Leg.: C.B.H. von Rosenberg.

Syntype, RMNH.AVES.87866, adult male, mounted skin. Loc.: Soëk [= Biak], [Indonesia], 24.iii.1869. Leg.: C.B.H. von Rosenberg.

Syntype, RMNH.AVES.87867, adult male, mounted skin. Loc.: Soëk [= Biak], [Indonesia], 24.iii.1869. Leg.: C.B.H. von Rosenberg.

Syntype, RMNH.AVES.87868, adult male, mounted skin. Loc.: Soëk [= Biak], [Indonesia], 27.iii.1869. Leg.: C.B.H. von Rosenberg.

***ColumbaSuperba*** Temminck, 1809: livr. 8, p. 75, pl. 33.

= *Ptilinopussuperbussuperbus* (Temminck, 1809).

Temminck (1809) based his description on a single specimen in his cabinet (Temminck 1807: 143, no. 978) which could not be found in the RMNH. For dating of this name and those of other pigeons and doves by Temminck in the years 1808–1811, see Dickinson et al. (2010). One female was sent to the SMF in March 1835.

***PtilopusviridisGeelvinkiana*** Schlegel, 1871a: 23.

***CarpophagaMusschenbroekii*** “von Rosenberg” Schlegel, 1871a: 23.

= *Ptilinopusviridisgeelvinkianus* Schlegel, 1871.

Syntype, RMNH.AVES.87783, adult male, mounted skin. Loc.: Mefor, [Indonesia], 22.i.1869. Leg.: C.B.H. von Rosenberg.

Syntype, RMNH.AVES.87784, adult male, mounted skin. Loc.: Mefor, [Indonesia], 02.ii.1869. Leg.: C.B.H. von Rosenberg.

Syntype, RMNH.AVES.87785, immature male, mounted skin. Loc.: Mefor, [Indonesia], 16.ii.1869. Leg.: C.B.H. von Rosenberg.

Syntype, RMNH.AVES.87869, adult male, mounted skin. Loc.: Miosnoum [= Mios Num], [Indonesia], 11.v.1869. Leg.: C.B.H. von Rosenberg.

Syntype, RMNH.AVES.87870, adult male, mounted skin. Loc.: Miosnoum [= Mios Num], [Indonesia], 08.v.1869. Leg.: C.B.H. von Rosenberg.

Syntype, RMNH.AVES.87871, adult male, mounted skin. Loc.: Miosnoum [= Mios Num], [Indonesia], 11.v.1869. Leg.: C.B.H. von Rosenberg.

Schlegel (1871a) mentioned that he received this series from Von Rosenberg under the manuscript name *Carpophaga Musschenbroekii*. Published as a junior synonym this is not an available name. However, subsequent use as a valid name by Salvadori (1893: 152) made this name available (ICZN 11.6.1).

RMNH.AVES.89422 from Andai, listed as syntype in Van den Hoek Ostende et al. (1997: 101) is no longer considered part of the type series because New Guinea itself is not mentioned by Schlegel as type locality.

***Ptilinopusviridispseudogeelvinkianus*** Junge, 1952: 247.

= *Ptilinopusviridisgeelvinkianus* Schlegel, 1871.

Holotype, RMNH.AVES.87869, adult male, mounted skin. Loc.: Miosnoum [= Mios Num], [Indonesia], 11.v.1869. Leg.: C.B.H. von Rosenberg.

Paratypes, RMNH.AVES.87870 and 87871.

Junge (1952) introduced the name *pseudogeelvinkianus* for the birds from Mios Num and restricted *geelvinkianus* to Numfor.

***Macropygiareinwardtiiminor*** Schlegel, 1873b: 106.

***Reinwardtoenareinwardtsibrevis*** Peters, 1937: xiii (nomen novum).

= *Reinwardtoenareinwardtibrevis* Peters, 1937.

Syntype, RMNH.AVES.87874, adult male, mounted skin. Loc.: Soëk [= Biak], [Indonesia], 28.iii.1869. Leg.: C.B.H. von Rosenberg.

Syntype, RMNH.AVES.88926, adult female, mounted skin. Loc.: Soëk [= Biak], [Indonesia], 26.iii.1869. Leg.: C.B.H. von Rosenberg.

Peters (1937) introduced *brevis* as a nomen novum for *Macropygiareinwardtiiminor* Schlegel, 1873 because the latter was preoccupied by *Macropygiaunchallminor* Swinhoe, 1870.

***Columbareinwardtsi*** Temminck, 1824: livr. 42, pl. 248.

***Reinwardtoenatypica*** Bonaparte, 1857: 59.

= *Reinwardtoenareinwardtireinwardti* (Temminck, 1824).

Holotype, RMNH.AVES.87872, adult, sex unknown, mounted skin. Loc.: Amboina [= Ambon], [Indonesia], [26.vi–12.viii.1821]. Leg.: C.G.C. Reinwardt.

The spelling of the specific name *reinwardtsi* is a lapsus (Mees 1964c). Holotype by monotypy.

Temminck (1824) gave Celebes as the origin of the specimen which is obviously an error as the species does not occur there. Reinwardt visited Ambon twice between 26 June 1821 and 12 August 1821.

***Columbaminiata*** Temminck, 1813: 460.

= ?*Spilopeliachinensischinensis* (Scopoli, 1786).

Temminck (1813a) based his name on his “Colombe mordorée” (1813: 369) and on the “Grande tourterelle de la Chine” by Sonnerat (1782: 178). The description, including translations in other languages, does not give us conclusive evidence as to the species involved. Schlegel (1873b: 127) placed both Sonnerat’s “Grande tourterelle de la Chine” and “Tourterelle grise de la Chine”, which is illustrated in Sonnerat (1782: 176) and is indeed *S.chinensis*, in the synonymy of *Turtur* (now *Spilopelia*) *chinensis*, a decision we tentatively follow here.

Temminck (1813a) listed names in the index for species in collections he had not seen himself.

***ColumbaTigrina*** Temminck, 1809: livr. 9, p. 94, pl. 43.

= *Spilopeliachinensistigrina* (Temminck, 1809).

Temminck’s description (1809) is based on a specimen in his own collection and two in the MNHN, one from Timor (Baudin expedition) and a specimen from Java reported by Leschenault. One of the MNHN specimens was later sent to the NMW (Jansen 2018: 270). Hence, Voisin et al. (2005: 851) listed a single specimen collected by Dumont (crew member of the Baudin expedition) as syntype (MNHN C.G.2002-532, now MNHN-ZO-MO-2002-532; Jansen 2018: 270), which could well have been seen by Temminck. The specimen in Temminck’s own collection seems no longer to be present in the RMNH. One was sent by Temminck to Leuven in 1822 and one to the MfN in 1824.

For dating of this name and those of other pigeons and doves by Temminck in the years 1808–1811, see Dickinson et al. (2010).

***ColumbaBitorquata*** Temminck, 1809: livr. 9, p. 86, pl. 40.

= *Streptopeliabitorquatabitorquata* (Temminck, 1809).

Temminck (1809) based his description on a single specimen in the MNHN collected by Maugé which is still there (Voisin et al. 2005: 850; MNHN C.G. 2002-536).

For dating of this name and those of other pigeons and doves by Temminck in the years 1808–1811, see Dickinson et al. (2010).

***Columbadusumieri*** Temminck, 1823: livr. 32, pl. 188.

= *Streptopeliabitorquatadusumieri* (Temminck, 1823).

Syntype, RMNH.AVES.87884, adult, sex unknown, mounted skin. Loc.: “Mariannes”, Philippines. Ex: MNHN.

The spelling of the specific name *dusumieri* is clearly a lapsus. Temminck (1823) named this species in honour of Dusumier, a miss-spelling for Jean-Jacques Dussumier.

Schlegel (1873b: 120) suggested the specimen originated from the Mariannas, which is an error added to the label.

According to the original description other syntypes are in the MNHN, Voisin et al. (2005: 851) listed one syntype (MNHN C.G.2002-537).

***Turturneglectus*** Schlegel, 1873b: 122.

= *Streptopeliadecipiensdecipiens* (Hartlaub & Finsch, 1870).

Syntype, RMNH.AVES.87885, adult, sex unknown, mounted skin. Loc.: North-east Africa. Leg.: A.B. Clot-Bey.

Syntype, RMNH.AVES.87886, adult, sex unknown, mounted skin. Loc.: North-east Africa. Leg.: A.B. Clot-Bey.

Syntype, RMNH.AVES.87887, adult, sex unknown, mounted skin. Loc.: North-east Africa. Leg.: A.B. Clot-Bey.

Syntype, RMNH.AVES.89423, adult male, mounted skin. Loc.: India [error]. Leg.: -.

Syntype, RMNH.AVES.89424, adult female, mounted skin. Loc.: India [error]. Leg.: -.

As the exact type locality is unknown other than “NE Africa”, the name *Turturneglectus* Schlegel, 1873, is linked to either *Streptopeliadecipiensdecipiens* (Hartlaub & Finsch, 1870) or *Streptopeliadecipienselegans* (Zedlitz, 1913). Further study is needed into the subspecific characters of the holotype and/or the travel reports of Clot to determine where the doves have been collected. See also *Turturfallax* Schlegel, 1873, collected during the same expedition.

***Columbalugens*** Rüppell, 1837: 64, pl. 22, fig. 2.

= *Streptopelialugens* (Rüppell, 1837).

Paralectotype, RMNH.AVES.90925.

The lectotype was designated by Steinbacher (1949: 106) and is in SMF (SMF 12603). Paralectotypes are in SMF and NHM (Steinheimer 2005a: 241).

***Columbagelastis*** Temminck, 1835: livr. 93, pl. 550.

= *Streptopeliaorientalisorientalis* (Latham, 1790).

Syntype, RMNH.AVES.87882, adult male, mounted skin. Loc.: Japan. Leg.: H. Bürger.

Syntype, RMNH.AVES.87883, adult female, mounted skin. Loc.: Japan. Leg.: H. Bürger.

Schifter et al. (2007: 153–154) listed two syntypes, received in exchange from the RMNH, in the NMW (NMW 3781 and 37872). One was sent to the SMF in March 1835.

***ColumbaAlba*** Temminck, 1809: livr. 9: 102, pl. 46.

= *Streptopeliaroseogrisea* (Sundevall, 1857).

Temminck (1809) does not refer to specimens of this white form of a *Streptopeliaroseogrisea* (see Van Grouw 2018). For dating of this name and those of other pigeons and doves by Temminck in the years 1808–1811, see Dickinson et al. (2010).

***Turturfallax*** Schlegel, 1873b: 124.

= *Streptopeliaroseogrisearoseogrisea* (Sundevall, 1857).

Holotype, RMNH.AVES.87889, adult, sex unknown, mounted skin. Loc.: North-east Africa. Leg.: A.B. Clot-Bey.

As the exact type locality is unknown other than “NE Africa”, the name *Turturfallax* Schlegel, 1873 is linked to either *Streptopeliaroseogrisearoseogrisea* (Sundevall, 1857) or *Streptopeliaroseogriseaarabica* (Neumann, 1904). Further study is needed into the subspecific characters of the holotype and/or the travel reports of Clot to determine where the doves have been collected. See also *Turturneglectus* Schlegel, 1873.

***Columbahumilis*** Temminck, 1824: livr. 44, pls 258 and 259.

= *Streptopeliatranquebaricahumilis* (Temminck, 1824).

Temminck (1824) referred to specimens from Manilla, Luzon, and “Bengale” in the MNHN and RMNH. No specimens could, however, be found in the RMNH.

***ColumbaCalva*** Temminck, 1811: livr. 14/15, p. 35, pl. 7.

= *Treroncalvuscalvus* (Temminck, 1811).

Holotype, RMNH.AVES.87890, adult, sex unknown, skin. Loc.: Coast of Angola. Ex: Cabinet Temminck.

Holotype by monotypy, as Temminck (1811) wrote that he was only aware of the specimen in his cabinet.

***Phalacrotrerondelalandii*** Bonaparte, 1854d: 873.

= *Treroncalvusdelalandii* (Bonaparte, 1854).

Possible syntype, RMNH.AVES.87894, adult male, skin. Loc.: Natal, South Africa. Ex: Maison Verreaux, 1867.

Bonaparte (1854d) pointed out that Verreaux and Verreaux (1851c: 423) applied the name *Vinagocalva* to a different taxon than Temminck did. According to Art. 49, ICZN (1999) and contrary to Voisin et al. (2004: 111–112) this name is not available and has therefore no types. Bonaparte gave the specimen(s) described in Verreaux and Verreaux the new name *delalandii* which became available under Art. 12.2. of the Code (ICZN 1999).

RMNH.AVES.87894 is listed here as a possible syntype as the year of collection is missing making it possible that it was collected later than 1854.

Another syntype is in the MNHN (MNHN C.G.1851-175). It was erroneously considered to be the holotype by Voisin et al. (2004: 112).

***Columbacapellei*** Temminck, 1822: livr. 24, pl. 143.

= *Treroncapelleicapellei* (Temminck, 1822).

Syntype, RMNH.AVES.87622, adult male, mounted skin. Loc.: Java, [Indonesia], [iv.1816–iii.1822]. Leg.: C.G.C. Reinwardt.

Syntype, RMNH.AVES.87623, adult female, mounted skin. Loc.: Java, [Indonesia], [iv.1816–iii.1822]. Leg.: C.G.C. Reinwardt.

According to the original description another syntype (a male) is in the MNHN but is not listed in Voisin et al. (2004). Schifter et al. (2007: 144) listed a syntype in the NMW which they received in 1823 in exchange with the RMNH. One was sent to the MfN in May 1827.

Reinwardt visited Java several times between April 1816 and March 1822.

***Treronnasica*** Schlegel, 1863d: 67.

= *Treroncurvirostranasica* Schlegel, 1863.

Syntype, RMNH.AVES.89431, adult male, mounted skin. Loc.: Martapoera, [Borneo], [Indonesia], x.1836. Leg.: S. Müller.

Syntype, RMNH.AVES.89432, adult male, mounted skin. Loc.: Martapoera, [Borneo], [Indonesia], [28.vii–17.xii.1836]. Leg.: S. Müller.

Syntype, RMNH.AVES.87891, adult male, mounted skin. Loc.: Borneo, [Indonesia], [1843–1848]. Leg.: C.A.L.M. Schwaner.

Syntype, RMNH.AVES.87892, adult male, mounted skin. Loc.: Borneo, [Indonesia], [1851–1858]. Leg.: J.H. Croockewit.

Syntype, RMNH.AVES.87893, adult female, mounted skin. Loc.: Borneo, [Indonesia], [1851–1858]. Leg.: J.H. Croockewit.

According to Schlegel (1863d), RMNH.AVES.89431 and 89432 were collected in Martapura, Sumatra. This must be an error as Martapura in Borneo is the island where Müller stayed in 1836.

Müller visited Borneo from 28 July 1836 to 17 December 1836, Schwaner from 1843 to 1848, and Crookewit from 1851 to 1858.

***Columbacinnamomea*** Temminck, 1835: livr. 93.

= *Treronfulvicollisfulvicollis* (Wagler, 1827).

Syntype, RMNH.AVES.87895, adult male, mounted skin. Loc.: South Borneo, [Indonesia]. Leg.: S. Müller [error].

Syntype, RMNH.AVES.87896, adult male, mounted skin. Loc.: South Borneo, [Indonesia]. Leg.: S. Müller [error].

*Columbacinnamomea* is not illustrated in the ‘Planches Coloriées’. Temminck (1835) only gave a description.

Müller, whose name occurs on the labels but not on the stand, cannot be the collector, since he did not visit Borneo until 1836. These specimens are presumed to have been collected by either Diard or Henrici (G.F. Mees, in litt.).

***Columbafulvicollis*** Wagler, 1827: [229].

***ColumbaAromatica*** Temminck, 1813a: 53.

= *Treronfulvicollisfulvicollis* (Wagler, 1827).

Wagler (1827) based his name on *Columbaaromatica Var.* en *Var. d* by Temminck (1813a: 53, pl. 6; Index 442) and gave Java as its origin. This is not correct. Temminck (1813a) wrote that a specimen of *aromatica* as well as related doves were sent to him from “Batavia” [= Jakarta]. Temminck referred to this as the port of shipment, not the origin of the specimen or species. Nominate *fulvicollis* occurs in Malaysia and Sumatra.

Three specimens were sent to Leuven in 1822 and 1829 and a skeleton was sent to the SMF in 1827.

*Columbaaromatica* Temminck is preoccupied by *Columbaaromatica* Gmelin, 1789 (= *Treronaromaticus* (Gmelin, 1789).

***Treronsangirensis*** Brüggemann, 1876: 79.

= *Trerongriseicaudasangirensis* Brüggemann, 1876.

Syntype, RMNH.AVES.87897, adult male, mounted skin. Loc.: Sanghir, [Indonesia], 02.i.1866. Leg.: D.S. Hoedt.

Syntype, RMNH.AVES.87898, adult male, mounted skin. Loc.: Sanghir, [Indonesia], 02.i.1866. Leg.: D.S. Hoedt.

Syntype, RMNH.AVES.87899, adult male, mounted skin. Loc.: Sanghir, [Indonesia], 28.x.1864. Leg.: C.B.H. von Rosenberg.

Syntype, RMNH.AVES.87900, adult male, mounted skin. Loc.: Sanghir, [Indonesia], 24.x.1864. Leg.: C.B.H. von Rosenberg.

Syntype, RMNH.AVES.87901, adult female, mounted skin. Loc.: Sanghir, [Indonesia], 26.x.1864. Leg.: C.B.H. von Rosenberg.

Syntype, RMNH.AVES.87902, adult female, mounted skin. Loc.: Siao, Sanghir, [Indonesia], 30.x.1865. Leg.: D.S. Hoedt.

Syntype, RMNH.AVES.87903, adult male, mounted skin. Loc.: Siao, Sanghir, [Indonesia], 31.x.1865. Leg.: D.S. Hoedt.

Syntype, RMNH.AVES.87904, adult female, mounted skin. Loc.: Siao, Sanghir, [Indonesia], 26.x.1865. Leg.: D.S. Hoedt.

Syntype, RMNH.AVES.87905, adult male, mounted skin. Loc.: Siao, Sanghir, [Indonesia], 07.xi.1865. Leg.: D.S. Hoedt.

Syntype, RMNH.AVES.87906, adult female, mounted skin. Loc.: Siao Oudong, Sanghir, [Indonesia]. Ex: L.D.H.A. Renesse van Duijvenbode, 1866.

Syntype, RMNH.AVES.87907, adult male, mounted skin. Loc.: Siao Luwan, Sanghir, [Indonesia]. Ex: L.D.H.A. Renesse van Duijvenbode, 1866.

***TreronVordermani*** Finsch, 1901: 162.

= *Trerongriseicaudavordermani* Finsch, 1901.

Syntype, RMNH.AVES.87910, adult male, skin. Loc.: Ardjasa, Kangean Island, [Indonesia], 02.v.1892. Leg.: A.G. Vorderman, 1896.

Syntype, RMNH.AVES.87911, adult female, skin. Loc.: Kangean Island, [Indonesia], v.1892. Leg.: A.G. Vorderman, 1896.

***Treronpompadoridehaani*** Voous, 1951: 97. In: Van Bemmel and Voous 1951: 97.

= *Trerongriseicaudawallacei* (Salvadori, 1893).

Syntype, ZMA.AVES.8703, adult male, skin. Loc.: Butung I. [= Buton], [Indonesia], viii.1909. Leg.: M. Mohari (75). Ex: J. Elbert.

Syntype, ZMA.AVES.8704, adult female, skin. Loc.: Butung I. [= Buton], [Indonesia], viii.1909. Leg.: M. Mohari (73). Ex: J. Elbert.

Voous (in Van Bemmel and Voous 1951) nominated ZMA.AVES.8703 and 8704 as the types, thereby excluding all other specimens from the type series. Roselaar and Prins (2000: 101) listed these syntypes as holo- and co-type, respectively. As this publication is after 1999, this does not constitute a valid lectotypification (ICZN 74.7).

***Columbaolax*** Temminck, 1823: livr. 41, pl. 241.

= *Treronolax* (Temminck, 1823).

According to the original description, syntypes are in the MNHN and RMNH. No specimens could, however, be found in the RMNH. Schifter et al. (2007: 145; NMW 65884) listed a syntype in the NMW which came in exchange from the RMNH in September 1833.

***Columbaoxyura*** “Reinwardt” Temminck, 1823: livr. 41, pl. 240.

= *Treronoxyurus* (Temminck, 1823).

Lectotype, RMNH.AVES.212751, adult male, mounted skin. Loc.: Java, [Indonesia], [1820–1823]. Leg.: G. van Raalten.

Paralectotype, RMNH.AVES.212752.

According to the original description the types, collected by Reinwardt and Diard, are in the RMNH and MNHN (MNHN-ZO-2005-2536). There is, however, no specimen in the RMNH from these collectors. We follow, however, Schlegel (1873b: 63) who listed RMNH.AVES.212751 as the type and so designated it the lectotype.

According to a note attached to RMNH.AVES.212752 by G.F. Mees, this specimen agrees more with the plate than RMNH.AVES.212751 and Mees questioned the collecting data given by Schlegel. On the bottom of the stand the original text is very vague, but with certainty does not refer to Boie or Bogor. We follow Mees and list this specimen as a paralectotype. Schifter et al. (2007: 144) listed two possible paralectotypes in the NMW (NMW 64788 and 64789) received from the RMNH in exchange in April 1830. One was sent to Leuven in August 1829.

Van Raalten stayed in Java from 1820 until 1827.

***ColumbaMilitaris*** Temminck, 1809: 23, pl.1.

= *Treronphoenicopterusphoenicopterus* (Latham, 1790).

Temminck (1809) referred to a male in the museum Raye de Breukelerwaert and a female in the MNHN (illustrated specimen). In the sale of the former collection, this species is not listed and this specimen could not be found in the RMNH. According to Voisin et al. (2004: 112) there is no specimen in the MNHN which could fit as a type either.

***ColumbaPsittacea*** Temminck, 1808: livr. 1, p. 28, pl. 4.

= *Treronpsittaceus* (Temminck, 1808).

Temminck (1808) based his description on more than 30 specimens from Indonesia. The depicted specimen was from his own cabinet, but is no longer present in the RMNH. One was sent to the MfN in March 1833 and one went to the SMF in March 1835.

***Columbasieboldii*** Temminck, 1835: livr. 93, pl. 549.

= *Treronsieboldiisieboldii* (Temminck, 1835).

Syntype, RMNH.AVES.87879, adult male, mounted skin. Loc.: Japan. Leg.: Ph.F.B. von Siebold.

Syntype, RMNH.AVES.87880, adult male, mounted skin. Loc.: Japan. Leg.: H. Bürger.

Syntype, RMNH.AVES.87881, adult female, mounted skin. Loc.: Japan. Leg.: H. Bürger.

One specimen was sent to the SMF in March 1835.

***Sphenocercuskorthalsi*** Bonaparte, 1857: 9.

= *Treronsphenuruskorthalsi* (Bonaparte, 1855) [error for 1857].

Syntype, RMNH.AVES.87876, adult male, mounted skin. Loc.: Mt. Gedeh, Java [Indonesia]. Leg.: S. Müller.

Syntype, RMNH.AVES.87877, adult female, mounted skin. Loc.: Mt. Gedeh, Java [Indonesia]. Leg.: S. Müller.

***TreronTeysmannii*** Schlegel, 1879d: 103.

= *Treronteysmannii* Schlegel, 1879.

Syntype, RMNH.AVES.87908, adult male, skin. Loc.: Sumba, [Indonesia]. Leg: J.E. Teysmann [error for J.E. Teijsmann]. Ex: Koloniaal Museum Haarlem, 1875.

Syntype, RMNH.AVES.87909, adult male, skin. Loc.: Sumba, [Indonesia]. Leg: J.E. Teysmann [error for J.E. Teijsmann]. Ex: Koloniaal Museum Haarlem, 1875.

***Treronchlorops*** Salvadori, 1874: 288.

= *Treronvernans* (Linnaeus, 1771).

Syntype, RMNH.AVES.87912, adult male, mounted skin. Loc.: Gorontalo, Celebes [= Sulawesi], [Indonesia], x.1841. Leg.: E.A. Forsten.

Syntype, RMNH.AVES.87913, adult male, mounted skin. Loc.: Tjikao, Java, [Indonesia], i.1827. Leg.: H. Boie.

Syntype, RMNH.AVES.87914, adult female, mounted skin. Loc.: Tjikao, Java, [Indonesia], i.1827. Leg.: H. Boie.

***Treronvernansgriseicapilla*** Schlegel, 1863d: 71.

= *Treronvernans* (Linnaeus, 1771).

Lectotype, RMNH.AVES.87915, adult male, mounted skin. Loc.: Southwest coast of Sumatra, [Indonesia], 1835. Leg.: S. Müller.

Paralectotypes, RMNH.AVES.88927–88942.

According to Peters (1937), Schlegel (1863d) described this taxon as *Trerongriseicapilla*. However, in his discussion on *Treronvernans*, Schlegel made the following remark on birds from Bangka and Sumatra: “Nous les désignerons sous l‘épithète supplémentaire de *griseicapilla*” [We classify them under the addition of the epithet *griseicapilla*]. The original combination of the taxon is therefore *Treronvernansgriseicapilla*. The lectotype was selected by Junge (1936: 6, ft 2). The year in which the specimen was collected given by Junge (1936) as 1853 is an error for 1835.

Ten years after his description, Schlegel (1873b: 49–51) considered *T.vernans* monotypic and “withdrew” his *griseicapilla*. In the second edition of his catalogue, he listed the type specimens of *griseicapilla* under *T.vernans*. See also under *Treronchlorops* Salvadori, 1874.

Van den Bossche visited Bangka from May 1859 until July 1861.

***Treronvernanskarimuniensis*** Hoogerwerf, 1962: 196.

= *Treronvernans* (Linnaeus, 1771).

Hoogerwerf based his description on a series of 14 specimens, two of which he nominated as “types” (MZB 23918 and 23922), hereby excluding all other specimens from the type series. This also applies to RMNH.AVES.27869 and 27870 listed in Van den Hoek Ostende et al. (1997: 113) as syntypes.

***Treronvernansparva*** Kloss, 1931: 308.

= *Treronvernans* (Linnaeus, 1771).

Holotype, RMNH.AVES.14025, adult male, skin. Loc.: Deli, Sumatra, [Indonesia]. Ex: MZB, 12.i.1950 (MZB 5978).

Kloss (1931) gave measurements of six males and four females collected by Van Heyst in Deli. One paratype is the MZB (MZB 5979).

For recognition of subspecies, see under *Treronchlorops* Salvadori, 1874.

***Columbamodesta*** Temminck, 1835: livr. 93, pl. 552.

= *Turacoenamodesta* (Temminck, 1835).

Lectotype, RMNH.AVES.87917, adult male, mounted skin. Loc.: Miromaffo [= Mount Miomaffo], Timor, [Indonesia], ix.1829. Leg.: S. Müller.

Paralectotype, RMNH.AVES.87916.

Schlegel (1873b: 107) designated the lectotype.

Müller (1842: 154) described how he ‘observed’ this new species on 3 November 1828, on his way to the Radja of Amabie, but apparently did not collect a specimen. The date with RMNH.AVES.87916 must be wrong, as Müller arrived in Timor in October 1828. One was sent to the SMF in March 1835.

**Columba (Peristera) puella** Schlegel, 1848: 17 (nec Lesson, 1827).

***Turturbrehmeriinfelix*** Peters, 1937: xiii, 113 (nomen novum).

= *Turturbrehmeriinfelix* Peters, 1937.

Syntype, RMNH.AVES.87918, adult male, mounted skin. Loc.: Dabocrom, Côte d’Or [= Ghana]. Leg.: H.S. Pel.

Syntype, RMNH.AVES.87919, adult male, mounted skin. Loc.: Dabocrom, Côte d’Or [= Ghana]. Leg.: H.S. Pel.

Syntype, RMNH.AVES.87920, adult female, mounted skin. Loc.: Dabocrom, Côte d’Or [= Ghana]. Leg.: H.S. Pel.

Possible syntype, ZMA.AVES.3420, adult, sex unknown, relaxed mount. Loc.: Gold Coast [= Ghana]. Leg.: -.

Schlegel (1848) based his description on three specimens, two collected by Pel and one in the zoological garden of NAM. There are, however, three specimens from Pel in the RMNH with the same data so we list all three as syntype. The bird from NAM could well be ZMA.AVES.3420.

Peters (1937: 113) introduced *Turturbrehmeriinfelix* as a nomen novum for Columba (Peristera) puella Schlegel, 1848.

***ColumbaTympanistria*** Temminck, 1809: livr. 8, p. 80, pl. 36.

= *Turturtympanistria* (Temminck, 1809).

Holotype, RMNH.AVES.87921, adult, [female], skin. Loc.: “Pays Auteniquoi” [= South Africa]. Leg.: ?F. Levaillant. Ex: Cabinet Temminck.

Holotype by monotypy. Temminck (1809) wrote having received a single specimen from Levaillant.

Van den Hoek Ostende et al. (1997: 114) listed two syntypes. We have listed RMNH.AVES.87921 as holotype based on comparison with pl. 36. Although the RMNH has a second specimen with the same data from Levaillant, we follow Temminck who referred to a single specimen.

***Columbavenusta*** Temminck, 1825: livr. 57, pl. 341, fig. 1.

= *Uropeliacampestris* (Spix, 1825).

Temminck (1825) gave no indication about the number of type specimens or their whereabouts. Two syntypes from the province of Goyas, Brazil, are in the MNHN (Voisin et al. 2005: 854; MNHN CG.2002-523 and 2002-524).

***ColumbaAurita*** Temminck, 1809: livr. 7, p. 60, pl. 25.

= *Zenaidaauritaaurita* (Temminck, 1809).

Temminck (1809) based his description on several specimens. The one illustrated on pl. 25 is from the collection of Raye van Breukelerwaert. Its whereabouts are unknown.

For dating of this name and those of other pigeons and doves by Temminck in the years 1808–1811, see Dickinson et al. (2010).

***ColumbaCarunculata*** Temminck, 1809: 19, pl. 11 is not applicable, artefact.

The name was based on an artefact. Temminck (1809) referred to a single specimen in the collection of Levaillant.

***ColumbaCaerulea*** Temminck, 1809: livr. 8, p. 82, pl. 37 is not applicable, artefact.

Temminck (1809) based his description on a single specimen in the collection of Holthuizen, which was an artefact.

For dating of this name and those of other pigeons and doves by Temminck in the years 1808–1811, see Dickinson et al. (2010).

***ColumbaHottentotta*** Temminck, 1809: 26, pl. 15 is not applicable, artefact.

Temminck (1809) based the name on a fictional description by Levaillant, therefore this name is excluded as a scientific name.

***ColumbaAuricularis*** Temminck, 1809: livr. 6, p. 54, pl. 21.

***C[olumba]Temminckii*** Wagler, 1827: 241 (is not applicable, artefact).

Syntype, RMNH.AVES.87635, adult, sex unknown, mounted skin. Loc.: “Îles de la mer Pacifique”. Ex: Museum Raye van Breukelerwaert.

Syntype, RMNH.AVES.87636, adult, sex unknown, mounted skin. Loc.: “Îles de la mer Pacifique”. Ex: Museum Raye van Breukelerwaert.

The description is based on artefacts. According to Schlegel (1873b: 65) these artefacts are modified domestic pigeons.

Van den Hoek Ostende et al. (1997: 85) listed this name as Temminck, 1813: 236. However, *auricularis* was already described on p. 54 and pl. 21 of livraison 6 (1809) of Temminck and Knip ‘Les Pigeons’ (1808–1811).

### ﻿GRUIFORMES

#### ﻿Sarothruridae

***Rallicularubra*** Schlegel, 1871b: 55.

= *Rallicularubrarubra* Schlegel, 1871.

Syntype, RMNH.AVES.87477, adult male, skin. Loc.: Hattam, Arfak, [Papua], [Indonesia], 13.iv.1870. Leg.: C.B.H. von Rosenberg, 1870.

Syntype, RMNH.AVES.87478, adult female, skin. Loc.: Hattam, Arfak, [Papua], [Indonesia], 13.iv.1870. Leg.: C.B.H. von Rosenberg, 1870.

***Rallusrufus*** Vieillot, 1819e: 564.

***Rallusdimidiatus*** Lesson, 1831: 537.

= *Sarothrurarufarufa* (Vieillot, 1819).

Syntype, RMNH.AVES.87483, adult male, mounted skin. Loc.: “Kaap” [South Africa]. Leg.: F. Levaillant (?).

Syntype, RMNH.AVES.87484, adult female, mounted skin. Loc.: “Kaap” [South Africa]. Leg.: F. Levaillant (?).

Vieillot (1819e) based this species on Temminck’s description of ‘Le Marouette Piqueté’ in the ‘Catalogue Systematique’ (1807: 266).

#### ﻿Rallidae

***Rallinaisabellina*** Schlegel, 1865b: 16.

= *Amaurornisisabellina* (Schlegel, 1865).

Syntype, RMNH.AVES.87431, adult male, mounted skin. Loc.: Gorontalo, Celebes [= Sulawesi], [Indonesia]. Leg.: E.A. Forsten.

Syntype, RMNH.AVES.87432, adult female, mounted skin. Loc.: Gorontalo, Celebes [= Sulawesi], [Indonesia]. Leg.: E.A. Forsten.

Schlegel (1865b) nominated two of the five specimens available to him as “type”, thereby excluding all other specimens from the type series.

Forsten stayed in Celebes twice between March 1840 and April 1842.

***GallinulaFrankii*** Schlegel, 1879g: 163.

= *Amaurornismoluccanamoluccana* (Wallace, 1865).

Holotype, RMNH.AVES.87433, adult, sex unknown, mounted skin. Loc.: [Andai], N.W. New Guinea, [Indonesia], [1868–1879]. Leg.: [W.H. Woelders]. Ex: G.A. Frank, 1879.

Holotype by monotypy. Schlegel (1879g) described how he received a single skin of this new species from the dealer Frank “brought to Europe by one of the Dutch missionaries residing on the coast of the North-West peninsula of New-Guinea”. This must have been missionary Woelders, who returned to the Netherlands in February 1879 on leave from Andai, New Guinea, bringing his collection with him.

***Gallinulaleucomelana*** Müller, 1842: 158.

= *Amaurornisphoenicurusleucomelana* (Müller, 1842).

Syntype, RMNH.AVES.87434, adult male, mounted skin. Loc.: Pritie [= Pariti], Timor, [Indonesia], xii.1828. Leg.: S. Müller.

Syntype, RMNH.AVES.87435, adult male, mounted skin. Loc.: Timor, [Indonesia], 1828. Leg.: S. Müller.

***DidusBrouckei*** Schlegel, 1854b: 345.

= *Aphanapteryxbonasia* (Sélys-Longchamps, 1848).

Extinct Red Rail from Mauritius. Based on a plate by Van den Broucke. Spelt *Broeckei* in Schlegel (1854c: 256).

***Gallinulaoculea*** Hartlaub, 1855: 357.

= *Canirallusoculeus* (Hartlaub, 1855).

Syntype, RMNH.AVES.87436, adult male, mounted skin. Loc.: Dabocrom, Côte d’Or [= Ghana], ii.1843. Leg.: H.S. Pel.

Syntype, RMNH.AVES.87437, adult female, mounted skin. Loc.: Dabocrom, Côte d’Or [= Ghana]. Leg.: H.S. Pel.

Syntype, RMNH.AVES.87438, adult male, mounted skin. Loc.: Dabocrom, Côte d’Or [= Ghana], iii.1850. Leg.: H.S. Pel.

***DidusHerbertii*** Schlegel, 1854b: 346.

= *Erythromachusleguati* (Milne-Edwards, 1874).

Extinct Leguat’s Rail from Rodrigues Island. Based on a plate by Herbert. Schlegel’s name from 1854 should have priority over Milne-Edwards’ name *leguati* that was published 20 years later.

***Fulicaatrajaponica*** Temminck & Schlegel, 1849: 120, pl. 77.

= *Fulicaatraatra* Linnaeus, 1758.

Syntype, RMNH.AVES.87439, adult, sex unknown, mounted skin. Loc.: Japan. Leg.: Ph.F.B. von Siebold.

Syntype, RMNH.AVES.87440, adult, sex unknown, mounted skin. Loc.: Japan. Leg.: H. Bürger.

Page 120 as well as pl. 77 of the ‘Fauna Japonica’ appeared in 1849 (Holthuis and Sakai 1970). See also Mlíkovský (2012b) and Dekker et al. (2001).

According to Schlegel (1865b: 61) RMNH.AVES.87440 is the depicted specimen.

***Fulicalugubris*** Müller, 1847b: 454.

= *Fulicaatralugubris* Müller, 1847.

Syntype, RMNH.AVES.87441, adult male, mounted skin. Loc.: Tjimahi [= Lake Cimahi], West Java, [Indonesia]. Leg.: S. Müller.

Syntype, RMNH.AVES.87442, adult male, mounted skin. Loc.: Tjimahi [= Lake Cimahi], West Java, [Indonesia]. Leg.: S. Müller.

Müller’s work was published in parts, with pp 281–472 published in 1847.

***Gallinulahaematopus*** Schlegel, 1865b: 44.

= *Gallinulatenebrosafrontata* Wallace, 1863.

Holotype, RMNH.AVES.87443, adult, sex unknown, mounted skin. Loc.: Celebes [= Sulawesi], [Indonesia], [ix–xi.1821]. Leg.: C.G.C. Reinwardt.

Paratypes, RMNH.AVES.88943–88957.

There is also a paratype in the NMW (NMW 48690; see Schifter et al. 2007: 117).

Reinwardt visited North Celebes from September 1821 until November 1821.

***Rallusetorques*** “Temminck” Schlegel, 1865: 23.

= *Gallirallusphilippensisphilippensis* (Linnaeus, 1766).

Syntype, RMNH.AVES.225199, adult male, mounted skin. Loc.: Celebes [= Sulawesi], [Indonesia]. Leg.: -.

Syntype, RMNH.AVES.226924, adult female, mounted skin. Loc.: Tondano, Celebes [= Sulawesi], [Indonesia], [22.iii.1840–19.vi.1841]. Leg.: E.A. Forsten.

Schlegel (1865b) published this manuscript name by Temminck in synonymy of *Gallirallusphilippensis*.

Forsten visited the Tondano area from 22 March 1840 to 19 June 1841.

***Hypotaenidiaphilippensisxerophila*** van Bemmel & Hoogerwerf, 1940: 470.

= *Gallirallusphilippensisxerophilus* (van Bemmel & Hoogerwerf, 1940).

Holotype, RMNH.AVES.14030, adult female, skin. Loc.: Gunung Api, Banda Sea, [Indonesia], 09.viii.1938. Ex: MZB, 12.i.1950 (MZB 12364).

There are two paratypes in the MZB (MZB 12365 and 12366).

***StictolimnasSharpei*** Büttikofer, 1893: 274.

= *Gallirallusphilippensis* subsp.

Holotype, RMNH.AVES.87485, adult, sex unknown, mounted skin. Loc.: South America [error]. Leg.: G.A. Frank, 1865.

This species is only known from the holotype. Olson (1986) placed it in the genus *Gallirallus*. The locality is clearly an error. Olson speculated that it may well have been collected on one of the Greater Sunda Islands. This is also the type species for the genus name *Stictolimnas* Büttikofer, 1893.

It is now considered a melanistic colour morph of the Buff-banded Rail *Gallirallusphilippensis* after genetic studies revealed that it is “genetically indistinguishable” from *G.philippensis* (den Tex and Dekker; Sharpe’s Rail’s Riddle, unpubl.). See Hume and Van Grouw (2014: 172–173) for further details.

***HypotaenidiaJentinki*** Sharpe, 1893: 268.

= *Gallirallustorquatussulcirostris* (Wallace, 1863).

Holotype, RMNH.AVES.87482, adult female, mounted skin. Loc.: Soela Mangoli, [Indonesia], 27.xi.1864. Leg.: D.S. Hoedt.

***RallusHoeveni*** Von Rosenberg, 1866: 144.

= *Gymnocrexplumbeiventrisplumbeiventris* (Gray, 1862).

Holotype, RMNH.AVES.87444, adult female, mounted skin. Loc.: Wonoembai, [Kobroor], Aru Isl., [Indonesia], 03.vi.1865. Leg.: C.B.H. von Rosenberg.

Holotype by monotypy.

Although given as 1867 by Van den Hoek Ostende et al. (1997: 63), vol. 29 of the “Natuurkundig Tijdschrift voor Nederlandsch Indië” was published in 1866.

In the same year, *hoeveni* was described again by Schlegel (1866f: 349) who referred to Von Rosenberg as the original author of *hoeveni*. Von Rosenberg indicated the specimen as being a male. The stand and label give “female” in Schlegels’ handwriting. Von Rosenberg also described the egg of this species in the RMNH, but no egg has been found collected by Von Rosenberg.

***Rallinarosenbergii*** Schlegel, 1866b: 212.

= *Gymnocrexrosenbergii* (Schlegel, 1866).

Holotype, RMNH.AVES.87445, adult male, skin. Loc.: Kema, Celebes [= Sulawesi], [Indonesia], 06.x.1864. Leg.: C.B.H. von Rosenberg.

Holotype by monotypy. Schlegel wrote having received “un individu”.

***Himantornishaematopus*** Hartlaub, 1855: 357.

= *Himantornishaematopus* Hartlaub, 1855.

Syntype, RMNH.AVES.87446, adult male, mounted skin. Loc.: Elmina, Côte d’Or [= Ghana]. Leg.: H.S. Pel.

Syntype, RMNH.AVES.87447, adult female, mounted skin. Loc.: Dabocrom, Côte d’Or [= Ghana]. Leg.: H.S. Pel.

Syntype, RMNH.AVES.87448, adult female, mounted skin. Loc.: Dabocrom, Côte d’Or [= Ghana], ii.1843. Leg.: H.S. Pel.

Hartlaub (1855) only mentioned Dabocrom in his description, but also described a male specimen. Therefore, the only male specimen, although from Elmina, is also included in the type series.

***Rallusexilis*** Temminck, 1831: livr. 88, pl. 523.

= *Laterallusexilis* (Temminck, 1831).

The holotype (by monotypy) is in the MNHN (Voisin and Voisin 2015: 67; MNHN C.G.2011-539).

***Corethrurarubra*** Sclater & Salvin, 1860: 300.

= *Laterallusruber* (Sclater & Salvin, 1860).

Paralectotype, RMNH.AVES.90930.

Sclater and Salvin (1860) gave no indication of the number of specimens available to them. Warren (1966: 249) listed a specimen in the NHM (NHMUK 1889.11.20.124) as holotype, thereby selecting it as lectotype.

***Ralluspectoralisconnectens*** Junge, 1952: 247.

= *Lewiniapectoralismayri* (Hartert, 1930).

Holotype, RMNH.AVES.18761, adult male, skin. Loc.: Paniai, [Papua], [Indonesia], 06.xi.1939. Leg.: Dutch New Guinea Expedition 1939, x.1951.

Junge (1952: 247) erroneously nominated the same specimen (RMNH.AVES.19267) as the type for two new taxa: *Porzanapusillamayri* and *Ralluspectoralisconnectens*. As RMNH.AVES.19267 is a specimen of *Porzanapusilla*, the error is made in refering to this specimen as the type of *connectens.* We have corrected this by listing RMNH.AVES.18761 here instead as the holotype of *connectens.* Its label has “Type” written on it in Junge’s handwriting, indicating he intended this specimen to be the (holo)type.

***Ralluspectoralis*** Temminck, 1831: livr. 88, text to pl. 523.

= *Lewiniapectoralispectoralis* (Temminck, 1831).

The type specimen from Australia, collected by Lesueur in 1801, is in the MNHN (MNHN-ZO-2011-539). See Jansen (2018: 296) for details.

***Gallinulaangulata*** Sundevall, 1850: 454.

= *Paragallinulaangulata* (Sundevall, 1850).

Paralectotypes, RMNH.AVES.229709–229710.

Gyldenstolpe (1926: 107) selected the lectotype by listing an adult male (NRM 569826) in the NRM as type.

***RallusRicordi*** Schlegel, 1865b: 8.

***RallusRicordi*** Bonaparte, 1856b: 598.

= *Pardirallussanguinolentus* subsp.

Holotype, RMNH.AVES.87449, adult, sex unknown, mounted specimen. Loc.: St. Domingue [error?]. Leg.: A. Ricord.

Bonaparte (1856b) published this name as a nomen nudum in the index only, without any further description. Schlegel (1865b: 8, 9) published it later in the synonymy of *Ralluscaesius* Tschudi (= *Pardirallussanguinolentus* subsp.). The locality Santo Domingo [Dominican Republic] is considered an error as this species does not occur there. Santo Domingo might therefore refer to places of similar name in either Chili or Ecuador.

***Gallinulaporphyrio*** Hartlaub, 1855: 357.

= *Porphyrioalleni* Thompson, 1842.

Syntype, RMNH.AVES.87469, adult male, mounted skin. Loc.: Elmina, Côte d’Or [= Ghana]. Leg.: H.S. Pel.

***Porphyriominutus*** von Heuglin, 1863a: 169.

= *Porphyrioalleni* Thomson, 1842.

Syntype, RMNH.AVES.148249, adult male, mounted skin. Loc.: Upper Nil [Sudan], iii.1863. Leg.: Th. von Heuglin, 1865.

Syntype, RMNH.AVES.227331, adult male, mounted skin. Loc.: Upper Nil [Sudan], iii.1863. Leg.: Th. von Heuglin, 1866.

***Porphyriobemmeleni*** Büttikofer, 1889c: 192.

= *Porphyrioindicus* Horsfield, 1821.

Holotype, RMNH.AVES.87458, adult female, mounted skin. Loc.: Lake Toba, Sumatra, [Indonesia]. Ex: Rotterdam Zoo, 29.v.1889.

Holotype by monotypy. Büttikofer (1889c) based his description on a single specimen: “Some days ago our Museum received a probably fully adult female of *Porphyrio*”.

***Porphyriosmaragdinus*** Temminck, 1827: livr. 71, pl. 421 (nomen novum).

= *Porphyrioindicus* Horsfield, 1821.

Temminck (1827) published *smaragdinus* as an unnecessary nomen novum for *Porphyrioindicus* Horsfield, 1821.

***Porphyriosmaragnotus*** Temminck, 1820: 700.

= *Porphyriomadagascariensis* (Latham, 1801).

Syntype, RMNH.AVES.87452, adult male, mounted skin. Loc.: “Kaap” [South Africa]. Ex: Cabinet Temminck.

Syntype, RMNH.AVES.87453, adult female, mounted skin. Loc.: “Kaap” [South Africa]. Ex: Cabinet Temminck.

RMNH.AVES.87454, an immature from South Africa, listed in Van den Hoek Ostende et al. (1997: 64) as syntype is no longer considered to be part of the type series as any evidence is lacking. One arrived in the MfN in April 1825 and one in Groningen in May 1847.

***Porphyriochloronotus*** Brehm, 1855: 293.

= *Porphyriomadagascariensis* (Latham, 1801).

Syntype, RMNH.AVES.226479, adult male, mounted skin. Loc.: Egypt. Leg. A.E. Brehm.

According to Schlegel (1865b: 54) this is one of the types of *chloronotus*.

***Porphyriobellus*** Gould, 1841a: 176.

= *Porphyriomelanotusbellus* Gould, 1841.

Paralectotypes, RMNH.AVES.227274–227275.

Stone (1913:136) selected the lectotype in the collection of the ANSP (ANSP 6401). Van den Hoek Ostende et al. (1997: 64) erroneously referred to RMNH.AVES.87457 as a paralectotype which is not part of the type series.

***Porphyriomelanopterus*** Bonaparte, 1856b: 599.

= *Porphyriomelanotusmelanopterus* Bonaparte, 1856.

Syntype, RMNH.AVES.87461, adult male, mounted skin. Loc.: Kaibobo, Ceram, [Indonesia]. Leg.: E.A. Forsten.

Syntype, RMNH.AVES.87462, adult male, mounted skin. Loc.: Kaibobo, Ceram, [Indonesia]. Leg.: E.A. Forsten.

Syntype, RMNH.AVES.87463, adult female, mounted skin. Loc.: Kaibobo, Ceram, [Indonesia]. Leg.: E.A. Forsten.

Forsten visited Seram late 1842.

***Porphyrioporphyrioplessenorum*** Neumann, 1941: 109.

= *Porphyriomelanotusmelanopterus* Bonaparte, 1856.

Holotype, RMNH.AVES.9862, adult male, skin. Loc.: Bratan, Bali, [Indonesia], 19.i.1938. Leg.: V. von Plessen, 15.xi.1941.

There are three paratypes in the MTD (MTD C44724, C45528, C45529; Eck and Quaisser 2004: 243).

***Porphyriomelanotus*** Temminck, 1820: 701.

= *Porphyriomelanotusmelanotus* Temminck, 1820.

Temminck gave no reference to the specimens he described.

***Porphyrioneglectus*** Schlegel, 1865b: 53.

= *Porphyriopoliocephaluspoliocephalus* (Latham, 1801).

Syntype, RMNH.AVES.87455, adult, sex unknown, mounted skin. Loc.: Travancore, near Cape Comorin [Kanniyakumari], Hindustan, India. Leg.: -.

Syntype, RMNH.AVES.87456, adult, sex unknown, mounted skin. Loc.: Hindustan, India. Leg.: Schaufuß, 1863.

In the original description, Schlegel (1865b) gave Nepal as the origin for RMNH.AVES.87456. However, on the label of this specimen (Schlegel’s catalogue no. 2) Hindustan is written.

***Porphyriohyacinthinus*** Temminck, 1820: 698.

= *Porphyrioporphyrio* (Linnaeus, 1758).

Temminck (1820) gave no reference to the specimens he described: the RMNH does not hold specimens from Europe dated prior to 1820.

***Porphyriocaesius*** Schlegel, 1865b: 52.

= *Porphyrioporphyrio* (Linnaeus, 1758).

Syntype, RMNH.AVES.87464, adult, sex unknown, mounted skin. Loc.: Sicily, Italy. Leg.: F. Cantraine.

Syntype, RMNH.AVES.87465, adult, sex unknown, mounted skin. Loc.: Sicily, Italy, [1833]. Leg.: F. Cantraine.

Syntype, RMNH.AVES.87466, adult, sex unknown, mounted skin. Loc.: Sicily, Italy, [1833]. Leg.: F. Cantraine.

Syntype, RMNH.AVES.87467, immature, mounted skin. Loc.: Sicily, Italy, [1833]. Leg.: F. Cantraine.

Cantraine visited Sicily in 1833.

***Porphyriopulverulentus*** Temminck, 1826: livr. 68, pl. 405.

= *Porphyriopulverulentus* Temminck, 1826.

Syntype, RMNH.AVES.87468, adult, sex unknown, mounted skin. Loc.: Philippines. Leg.: -.

Temminck (1826) described this species as occurring from Senegal to South Africa, which is erroneous as this species is from the Philippines. According to the original description other syntypes are in the MNHN, but are not listed in Voisin and Voisin (2015). RMNH.AVES.87468 is the depicted specimen on pl. 405.

***Rallusolivaceus*** Vieillot, 1819e: 561.

= *Porzanaalbicollistyphoeca* Peters, 1932.

According to Stresemann (1953a: 328) Vieillot (1819e) based this species on the description of “Le Rale d’eau de St. Domingue” in Temminck’s catalogue (1807: 177, 265). This specimen is not found in the RMNH. Stresemann restricted the type locality to Guiana. Hellmayr and Conover (1942: 364) did not consider *olivaceus* to belong to *albicollis*, giving priority to *typhoeca* Peters, 1932 as does the IOC.

***Erythracinereamedia*** Schlegel, 1865b: 33.

= *Porzanacinerea* (Vieillot, 1819).

Holotype, RMNH.AVES.90976, adult male, mounted skin. Loc.: Borneo, [Indonesia]. Leg.: -.

Holotype by monotypy. Schlegel (1865b) published this manuscript name given by Bonaparte and listed only RMNH.AVES.90976 as the type.

***Erythracinereaminima*** Schlegel, 1865b: 34.

= *Porzanacinerea* (Vieillot, 1819).

Holotype, RMNH.AVES.87451, adult female, mounted skin. Loc.: Utanata, [Papua], [Indonesia], vi.1828. Leg.: S. Müller.

Holotype by monotypy. Schlegel (1865b) published this manuscript name by Bonaparte and listed only RMNH.AVES.87451 as the type.

***Gallinulaerythrothorax*** Temminck & Schlegel, 1849: 121, pl. 78.

= *Porzanafuscaerythrothorax* (Temminck & Schlegel, 1849).

Syntype, RMNH.AVES.87471, adult, sex unknown, mounted skin. Loc.: Japan. Leg.: Ph.F.B. von Siebold.

Syntype, RMNH.AVES.87472, adult female, mounted skin. Loc.: Japan. Leg.: Ph.F.B. von Siebold.

Syntype, RMNH.AVES.87473, adult female, mounted skin. Loc.: Japan. Leg.: H. Bürger.

Syntype, RMNH.AVES.87474, adult male, mounted skin. Loc.: Japan. Leg.: H. Bürger.

Syntype, RMNH.AVES.87475, adult, sex unknown, mounted skin. Loc.: Japan. Leg.: H. Bürger.

Syntype, RMNH.AVES.87476, adult, sex unknown, mounted skin. Loc.: Japan. Leg.: H. Bürger.

In this catalogue we follow Holthuis and Sakai (1970) over Mlíkovský (2012b) for the year of publication. This can have consequences on the status and selection of type specimens for this name if the plate was published prior to the description in the livraison. Taxa based upon holotypes are not affected but will differ where the name is based upon a series of specimens.

***Gallinularubiginosa*** Temminck, 1825: livr. 60, pl. 357.

= *Porzanafuscafusca* (Linnaeus, 1766).

Syntype, RMNH.AVES.87470, adult male, mounted skin. Loc.: Java, [Indonesia]. Leg.: C.G.C. Reinwardt.

***Porzanapusillamayri*** Junge, 1952: 247.

= *Porzanapusillamayri* Junge, 1952.

Holotype, RMNH.AVES.19267, adult male, skin. Loc.: Paniai, [Papua], [Indonesia], 04.xi.1939. Leg.: Dutch New Guinea Expedition 1939.

Junge (1952: 247) erroneously nominated the same specimen (RMNH.AVES.19267) as the type for two new taxa: *Porzanapusillamayri* and *Ralluspectoralisconnectens*. As RMNH.AVES.19267 is a specimen of *Porzanapusilla*, the error is made in the nomination of the type of *connectens*, which we further discuss under that name.

***RallusSandwichensis*** Gmelin, 1789: 717.

***Rallusobscurus*** Gmelin, 1789: 718.

***PennulaWilsoni*** Finsch, 1898: 77.

= *Porzanasandwichensis* (Gmelin, 1789).

Lectotype for *sandwichensis*, syntype for *obscurus* and *Wilsoni*, RMNH.AVES.87450, adult, sex unknown, mounted skin. Loc.: Sandwich Islands [= Hawaii]. Leg.: - [Capt. J. Cook’s voyage?].

Sharpe (1893: 269) selected the Leiden specimen as the lectotype for *sandwichensis*, by naming it “the typical specimen”. This lectotypification has been overlooked by Olson (1994) and Jansen and Roe (2013), see below.

Jansen and Roe (2013) summarised their paper on the RMNH specimen as follows: “The provenance of the two types in Leiden and Vienna” [ed.: NMW 50.728, received from Temminck in 1821] “is shrouded in mystery, as their early history is incomplete, and both changed hands before reaching their current destinations. Furthermore, one or both specimens were originally described as *Rallusobscurus* Gmelin, 1789, a synonym of *Porzanasandwichensis*”. They concluded that “Due to the lack of proper documentation and hence our inability to definitively link either of Latham’s original descriptions to a specific specimen, the RMNH and NMW specimens should henceforth be regarded as syntypes of *Rallussandwichensis* Gmelin, 1789 and *R.obscurus* Gmelin, 1789”.

Olson (1994) reached the same conclusion as Jansen and Roe (2013): “One or both of these are types of *Rallussandwichensis* Gmelin…” (Olson, 1994).

Finsch (1898) started his discussion on the “Sandwich rail” in the RMNH introducing a new name for *Crexsandwichensis*Schlegel (1865b: 25) and referring to “Schlegel’s type”. Introducing a new name was necessary according to Finsch because he questioned the validity of the origin of this specimen and thus the validity of the epithet *sandwichensis*, following Newton in the opinion that none of Cook’s specimens had survived.

***Rallinaminahasa*** Wallace, 1863a: 346.

= *Rallinaeurizonoidesminahasa* Wallace, 1863.

Syntype, RMNH.AVES.87479, adult male, mounted skin. Loc.: Minahassa, Celebes [= Sulawesi], [Indonesia]. Leg.: A.R. Wallace.

Another syntype is in the NHM (NHMUK 1873.5.12.2359).

***Gallinulaeurizona*** Temminck, 1826: livr. 70, pl. 417.

= *Rallinafasciata* (Raffles, 1822).

Syntype, RMNH.AVES.87480, adult male, mounted skin. Loc.: Java, [Indonesia], [vi.1826–ix.1827]. Leg.: [H. Boie].

Syntype, RMNH.AVES.87481, adult female, mounted skin. Loc.: Java, [Indonesia], [vi.1826–ix.1827]. Leg.: [H. Boie].

In the index of the ‘Planches Coloriées’ Temminck refers to this bird as *Gallinulaeuryzonia*. Schifter et al. (2007: 114) listed a possible syntype in the NMW (NMW 71129). According to Schlegel (1865b: 19) these specimens were collected by Boie, who visited Java from June 1826 until September 1827.

***Rallusabyssinicus*** Rüppell, 1845: 127, pl. 46.

= *Rougetiusrougetii* (Guérin-Méneville, 1843).

Paralectotype, RMNH.AVES.90931.

The lectotype was designated by Steinbacher (1949: 105) and is in the SMF (SMF 12711). Paralectotypes are in the SMF and NHM (Steinheimer 2005a: 239). Steinheimer (2005a: 239) mentioned another possible paralectotype in the RMNH, but there is no other specimen which could fit as a type.

#### ﻿Gruidae

***Gruscinerealongirostris*** Temminck & Schlegel, 1849: 117.

***GrusSchlegelii*** Blyth, 1873: 419.

= *Antigonecanadensiscanadensis* (Linnaeus, 1758).

Holotype, RMNH.AVES.87425, adult male, mounted skin. Loc.: Japan. Leg.: H. Bürger.

Holotype by monotypy. Temminck and Schlegel (1849: 118) received a single specimen from Japan. Plate 72 illustrating the holotype was published after the text (Mlíkovský 2012b).

Blyth (1873) based his description on the plate of *Gruscinerealongirostris* of Temminck and Schlegel (1850).

***Grusleucauchen*** Temminck, 1828: livr. 76, pl. 449.

= *Antigonevipio* (Pallas, 1811).

Syntype, RMNH.AVES.87429, adult male, mounted skin. Loc.: Japan. Leg.: Ph.F.B. von Siebold.

Schifter et al. (2007: 109) listed a possible syntype collected by Von Siebold which arrived in the NMW (NMW 70930) in September 1833. For more information on Siebold’s collection see Holthuis and Sakai (1970), Dekker et al. (2001), and Morioka et al. (2005). A specimen was exchanged with the MNHN in March 1836. One was sent to the MfN in March 1833.

***Grusmonacha*** Temminck, 1835: livr. 94, pl. 555.

= *Grusmonacha* Temminck, 1835.

Syntype, RMNH.AVES.87426, adult male, mounted skin. Loc.: Japan. Leg.: H. Bürger.

Temminck (1835) referred to three specimens collected by Von Siebold and Bürger of which RMNH.AVES.87426 is the only one still present in the RMNH. One was sent to the SMF in March 1835 and one to the MNHN in April 1835.

### ﻿PODICIPEDIFORMES

#### ﻿Podicipedidae

***Podicepsrubricollismajor*** Temminck & Schlegel, 1844: 122, pl. 78B.

= *Podicepsgrisegenaholbollii* Reinhardt, 1853.

Syntype, RMNH.AVES.107498, adult female, mounted skin. Loc.: Japan. Leg.: Ph.F.B. von Siebold.

Syntype, RMNH.AVES.107499, adult, sex unknown, mounted skin. Loc.: Japan. Leg.: Ph.F.B. von Siebold.

Syntype, RMNH.AVES.107500, adult female, mounted skin. Loc.: Japan. Leg.: Ph.F.B. von Siebold.

### ﻿PHOENICOPTERIFORMES

#### ﻿Phoenicopteridae

***Phoenicopterusantiquorum*** Temminck, 1820: Man. d’Orn. 2: 587.

= *Phoenicopterusroseus* Pallas, 1811.

Nomen nudum (Grant and Mackworth-Praed 1933: 17).

### ﻿CHARADRIIFORMES

#### ﻿Turnicidae

***Turnixhottentottus*** Temminck, 1815a: 636.

= *Turnixhottentottus* Temminck, 1815.

Possible syntype, RMNH.AVES.87416, adult, sex unknown, mounted skin. Loc.: “Cap”, [South Africa]. Leg.: -.

Possible syntype, RMNH.AVES.87417, adult, sex unknown, mounted skin. Loc.: “Cap”, [South Africa]. Leg.: -.

Possible syntype, RMNH.AVES.87418, adult, female, mounted skin. Loc.: “Cap”, [South Africa]. Leg.: -.

Temminck (1815a) based his description on two specimens in his own cabinet collected by Levaillant (Temminck, 1807: 160). The RMNH houses three instead of two specimens with no other data than “Cap” and reference to their type status. Based on this limited information we list them here as types. RMNH.AVES.87418 is added here tentatively as it is the only specimen of which the sex, which is not referred to by Temminck, is indicated under the stand and as it differs in plumage, being much more orange on the breast, that is not described either.

***Hemipodiusmaculosus*** Temminck, 1815a: 631.

***TurnixMaculatus*** Vieillot, 1823: 330.

= *Turnixmaculosusmaculosus* (Temminck, 1815).

Syntype, RMNH.AVES.87424, adult female, mounted skin. Loc.: “Oceania” [= Kupang Bay, West Timor, Indonesia]. Leg.: [Baudin Expedition, R. Maugé, 22.viii.1801–13.xi.1801].

According to the label this specimen is a male from ‘Oceania’. However, in his description Temminck (1815a) refers to ‘Nouvelle Hollande’ as the type locality. According to Peters (1934: 144) this should be Timor, Indonesia. The specimen was later identified as a female, which is correct.

Jansen (2017: 476) identified this specimen as part of the material gathered by René Maugé during the Baudin Expedition (1801–1804). It was collected near Kupang, West-Timor, between 22 August and 13 November 1801. Temminck based his description on one specimen in his own collection and three syntypes in the MNHN of which only one can be found in the database (MNHN-ZO-2011-543: photo in Jansen 2018: 354).

***Hemipodiusnanus*** Sundevall, 1850: 110.

= *Turnixnanus* (Sundevall, 1850).

Syntype, RMNH.AVES.229704, adult male, mounted skin. Loc.: South Africa. Leg.: J.A. Wahlberg.

***Hemipodiusthoracicus*** Temminck, 1815a: 622.

= *Turnixocellatus* subsp.

Temminck (1815a) based his name on a single specimen from Luzon in the MNHN, but according to him it is no longer there. It is not listed in Voisin and Voisin (2015).

As two subspecies occur on Luzon, *T.o.ocellatus* (Scopoli, 1786) and *T.o.benguetensis* Parkes, 1968, it is unclear to which of these two *thoracicus* referred.

***Hemipodiusfasciatus*** Temminck, 1815a: 634.

= *Turnixsuscitatorfasciatus* (Temminck, 1815).

According to Temminck (1815a) he had seen a single specimen in the MNHN. No type for *fasciatus* is listed in Voisin and Voisin (2015).

***Hemipodiuspugnax*** Temminck, 1815a: 612.

= *Turnixsuscitatorsuscitator* (Gmelin, 1789).

Syntype, RMNH.AVES.87419, adult male, mounted skin. Loc.: Java, [Indonesia]. Leg.: -.

Syntype, RMNH.AVES.87420, adult female, mounted skin. Loc.: Java, [Indonesia]. Leg.: -.

Syntype, RMNH.AVES.87421, adult female, mounted skin. Loc.: Java, [Indonesia]. Leg.: -.

Temminck (1815a) also mentions a syntype in the MNHN.

***Turnixsuscitatorbaweanus*** Hoogerwerf, 1962: 199.

= *Turnixsuscitatorsuscitator* (Gmelin, 1789).

Hoogerwerf (1962) nominated a male and a female in the collection in MZB as “types” thereby excluding all other specimens from the type series, including RMNH.AVES.27865 and 27866, listed in Van den Hoek Ostende et al. (1997: 59) as syntypes.

Sudaryanti et al. (2006) designated a lectotype (MZB 22783) from the two syntypes in the MZB. However, the mere fact that Hoogerwerf did not select a holotype in his description is not a valid reason for a lectotype designation after 1999 (ICZN Recommendation 74G).

***Turnixsuscitatorkuiperi*** Chasen, 1937: 208.

= *Turnixsuscitatorsuscitator* (Gmelin, 1789).

Holotype, RMNH.AVES.14029, adult female, skin. Loc.: Gunung Liang, Billiton, [Indonesia], 09.ii.1936. Leg.: F.J. Kuiper. (292). Ex: MZB, 12.i.1950 (MZB 16329).

Paratypes, RMNH.AVES.25422, ZMA.AVES.47874–47875, ZMA.AVES.47929.

Fourteen specimens were examined by Chasen (1937).

***Hemipodiusdussumier*** Temminck, 1828: livr. 76, pl. 454, fig. 2.

= *Turnixsylvaticusdussumier* (Temminck, 1828).

Syntype, RMNH.AVES.87422, adult female, mounted skin. Loc.: Bengal. Leg.: J.-J. Dussumier.

Syntype, RMNH.AVES.87423, adult female, mounted skin. Loc.: Bengal. Leg.: J.-J. Dussumier.

According to the original description other syntypes are in the MNHN.

***Hemipodiustachydromus*** Temminck, 1815a: 626.

= *Turnixsylvaticussylvaticus* (Temminck, 1815).

Temminck (1815a) referred to specimens in his cabinet, which no longer seem to be present in the RMNH.

***Hemipodiuslunatus*** Temminck, 1815a: 629.

= *Turnixsylvaticussylvaticus* (Temminck, 1815).

Temminck (1815a) referred to a single specimen in the Leverian museum.

***Turnixjoudera*** Gray, 1846: 129.

= *Turnixtankitanki* Blyth, 1843.

Syntype, RMNH.AVES.227458, adult sex unknown, mounted skin. Loc.: Nepal. Leg.: B.H. Hodgson.

Syntype, RMNH.AVES.227459, adult sex unknown, mounted skin. Loc.: Nepal. Leg.: B.H. Hodgson.

Syntype, RMNH.AVES.227460, adult sex unknown, mounted skin. Loc.: Nepal. Leg.: B.H. Hodgson.

Gray (1846) published this name based on a description and plate by Hodgson (1838), in which Hodgson did not provide his description with a scientific name. According to Gray (1846: preface) his catalogue described the collection of Hodgson and “the types of the specimens described in that gentleman’s various scientific papers”. A series of specimens was selected for the NHM and duplicates were distributed to various other museums, including the RMNH (erroneously called “Museum of the University of Leyden”).

***Hemipodiusnigrifrons*** Temminck, 1815a: 610.

= Turnixcf.tanki Blyth, 1843.

According to Temminck (1815a) the holotype (by monotypy) is in the MNHN. It is not listed in Voisin and Voisin (2015) and has not been noticed during recent visits or found in register books by J. Jansen (pers. comm., 24 Nov 2020). However, Van Grouw (pers. comm., 2022) has found the alleged specimen (MNHN A.C.2012-159) and considers it to be an artefact.

Its identification is a mystery. According to Temminck’s description it originated from India. Vieillot and Oudart (1843) pl. 218 show a *Turnix* with a remarkably bold black-and-white forehead (as in *Pteroclesquadricinctus*), as in Temminck’s description and in Stephens (1819: 388–389). This pattern cannot be linked to any *Turnix* species, be it from India where only three species occur, or elsewhere. As the body parts most closely resemble *T.tanki* Blyth, 1843, we have listed it here provisionally under that name.

#### ﻿Burhinidae

***Oedicnemusmaculosus*** Temminck, 1824: livr. 49, pl. 292.

= *Burhinuscapensismaculosus* (Temminck, 1824).

Syntype, RMNH.AVES.87559, adult, sex unknown, mounted skin. Loc.: Senegambie. Leg.: -.

According to the original description other syntypes are in the MNHN, MfN and SMF.

***Oedicnemusaffinis*** Rüppell, 1837b: 210.

= *Burhinuscapensismaculosus* (Temminck, 1824).

According to the correspondence in the RMNH archives (letter by Rüppell to Temminck dated 15 October 1837) the RMNH must have received one (type) specimen of this newly described taxon. Since there is no specimen in the collection which fits as type it must be considered lost.

***Œdicnemuscrepitans*** Temminck, 1815b: 322.

= *Burhinusoedicnemusoedicnemus* (Linnaeus, 1758).

Possible syntype, RMNH.AVES.258648, adult, sex unknown, mounted skin. Loc.: Europe. Leg.: -.

Although any information about the origin or location of the specimen is lacking, we list it here as a possible syntype because of Temminck’s handwriting and Schlegels’ confirmation to *Oedicnemuscrepitans* on the stand and in Schlegel (1865a: 20).

***Oedicnemusbüttikoferi*** Reichenow, 1898: 182.

= *Burhinusvermiculatusbuettikoferi* (Reichenow, 1898).

Syntype, RMNH.AVES.87560, adult male, skin. Loc.: Fisherman Lake, Liberia, 15.iii.1881. Leg.: J. Büttikofer and C.F. Sala.

Syntype, RMNH.AVES.87561, adult male, skin. Loc.: Marfa [= Mafa] River, Grand Cape Mountain, Liberia, 18.viii.1881. Leg.: J. Büttikofer and C.F. Sala.

Although Sala is also mentioned on the label of RMNH.AVES.87561, he cannot have been the collector as he died two months earlier on 10 June 1881.

#### ﻿Chionidae

***Chionisminor*** Hartlaub, 1841: 5.

= *Chionisminorminor* Hartlaub, 1841.

Holotype, RMNH.AVES.87568, adult, sex unknown, skin. Loc.: Kerguelen Islands. Ex: G.A. Frank.

Holotype by monotypy. Hartlaub (1841) wrote that he found a single specimen in the Leiden collection.

#### ﻿Haematopodidae

***Hæmatopusniger*** “Cuvier” Temminck, 1820: 131 (nec Pallas).

***Haematopusmoquini*** Bonaparte, 1856a: 1020 (nomen novum).

= *Haematopusmoquini* Bonaparte, 1856.

*Haematopusmoquini* Bonaparte is a nomen novum for *Haematopusniger* Temminck. Both names are based on “black oystercatchers” referred to by Cuvier without specifically referring to specimens. Hence Temminck’s and Bonaparte’s names do not have types.

***Haematopuspalliatus*** Temminck, 1820: 532.

= *Haematopuspalliatuspalliatus* Temminck, 1820.

Syntype, RMNH.AVES.87491, adult, sex unknown, mounted skin. Loc.: Brazil. Leg.: -.

Syntype, RMNH.AVES.87492, adult, sex unknown, mounted skin. Loc.: Brazil. Leg.: -.

The type locality was restricted to Rio de Janeiro by Von Berlepsch (1908: 304).

#### ﻿Recurvirostridae

***Recurvirostrarubricollis*** Temminck, 1820: 592 (nomen novum).

= *Recurvirostranovaehollandiae* Vieillot, 1816.

Nomen novum for “*R.americana*”. According to Voisin & Voisin (2012: 49) Temminck (1820) based *R.rubricollis* on the same specimen (MNHN C.G. 2012-182) as Vieillot did for *R.novaehollandiae*.

#### ﻿Charadriidae

***Charadriusazarai*** Temminck, 1823: livr. 31, pl. 184.

= *Charadriuscollaris* Vieillot, 1818.

We have no indication of the whereabouts of the type(s) of *Charadriusazarai* Temminck, 1823.

***Charadriuspyrrhothorax*** Gould, 1837: pl. 299.

= *Charadriusmongolus* subsp.

Syntype, RMNH.AVES.87493, adult male, mounted skin. Loc.: St. Petersburg, Russia. Leg.: J.F. Brandt.

It is not clear which subspecies of *C.mongolus* is involved here.

***Charadriuspecuarius*** Temminck, 1823: livr. 31, pl. 183.

= *Charadriuspecuarius* Temminck, 1823.

Temminck (1823) referred to Levaillant’s travels in South Africa. We have no indication of the whereabouts of the type specimens.

***Charadriusperonii*** Schlegel, 1865a: 33.

= *Charadriusperonii* Schlegel, 1865.

Lectotype, RMNH.AVES.87494, adult male, skin. Loc.: Samau, [Indonesia], iii.1828. Leg.: S. Müller.

Paralectotypes, RMNH.AVES.87495–87502, 87503–87506 (= *Charadriusjavanicus* Chasen, 1938).

The specimens from Samau were designated lectotype and allotype by Junge (1936). Müller stayed on Borneo from 28 July to 17 December 1836, Kuhl and Van Hasselt travelled together on Java between December 1820 and September 1821. Crookewit visited Borneo between 1851 and 1856.

***Charadriusperoniichaseni*** Junge, 1939: 120.

= *Charadriusperonii* Schlegel, 1865.

Holotype, RMNH.AVES.87507 (6651), adult female, skin. Loc.: Lasikin, Simalur [= Simeulue], [Indonesia], 06.iv.1913. Leg.: E.R. Jacobson and W.C. van Heurn.

Paratypes, RMNH.AVES.87508–87512.

***Charadriusruficapillus*** Temminck, 1821: livr. 8, pl. 47, fig. 2.

= *Charadriusruficapillus* Temminck, 1821.

Following Dickinson et al. (2022) (see also Introduction of this catalogue under “What is new in this 2023 version”: 14) the specimen illustrated on pl. 47, fig. 2 is the holotype, since the text that appeared later is irrelevant regarding type status. Any listing of syntypes for this name in any type catalogue is erroneous. According to Temminck, the illustrated specimen is in the MNHN where Voisin and Voisin (2012: 38) reported two syntypes, one of which will likely be the holotype. See also Jansen (2018: 344).

***Charadriusnigrifrons*** “Cuvier” Temminck, 1821: livr. 8, pl. 47, fig. 1.

= *Elseyornismelanops* (Vieillot, 1818).

Following Dickinson et al. (2022) (see also Introduction of this catalogue under “What is new in this 2023 version”: 14) the specimen illustrated on pl. 47, fig. 1 is the holotype, since the text that appeared later is irrelevant regarding type status. Any listing of syntypes for this name in any type catalogue is erroneous. According to Temminck, the illustrated specimen is in the MNHN (MNHN-ZO-2012-142 which is confirmed by Voisin and Voisin (2012: 40). See also Jansen (2018: 346).

***Charadriuspluvialisorientalis*** Temminck & Schlegel, 1849: 104, pl. 62.

***Charadriusauratuslongipes*** Schlegel, 1858: 411.

= *Pluvialisfulva* (Gmelin, 1789).

Lectotype for *orientalis*, syntype for *longipes*, RMNH.AVES.87515, adult, sex unknown, mounted skin. Loc.: Japan. Leg.: Ph.F.B. von Siebold, 31.viii.1829, Dernière Expedition.

Syntype for *longipes*, paralectotype for *orientalis*, RMNH.AVES.87516, adult male, mounted skin. Loc.: Borneo, [Indonesia], [28.vii.1836–17.xii.1836]. Leg.: S. Müller.

Syntype for *longipes*, paralectotype for *orientalis*, RMNH.AVES.87517, adult male, mounted skin. Loc.: Pagouat, N Celebes [= Sulawesi], [Indonesia]. Leg.: E.A. Forsten.

Syntype for *longipes*, paralectotype for *orientalis*, RMNH.AVES.90649, adult female, mounted skin. Loc.: Borneo, [Indonesia], [28.vii.1836–17.xii.1836]. Leg.: S. Müller.

Syntype for *longipes*, paralectotype for *orientalis*, RMNH.AVES.90650, adult male, mounted skin. Loc.: Borneo, [Indonesia]. Leg.: C.A.L.M. Schwaner.

Syntype for *longipes*, paralectotype for *orientalis*, RMNH.AVES.90651, adult male, mounted skin. Loc.: Pagattan, Borneo, [Indonesia], 2.ix.1844. Leg.: C.A.L.M. Schwaner.

Syntype for *longipes*, paralectotype for *orientalis*, RMNH.AVES.90653, adult male, mounted skin. Loc.: Java, [Indonesia]. Leg.: H. Kuhl and J.C. van Hasselt.

Syntype for *longipes*, paralectotype for *orientalis*, RMNH.AVES.90654, adult male, mounted skin. Loc.: Gorontalo, Celebes [= Sulawesi], [Indonesia], 24.ix.1842. Leg.: E.A. Forsten.

Syntype for *longipes*, paralectotype for *orientalis*, RMNH.AVES.90655, adult male, mounted skin. Loc.: Pagouat, N Celebes [= Sulawesi], [Indonesia]. Leg.: E.A. Forsten.

Syntype for *longipes*, paralectotype for *orientalis*, RMNH.AVES.90656, adult female, mounted skin. Loc.: Timor, [Indonesia], x.1829. Leg.: S. Müller.

Syntype for *longipes*, paralectotype for *orientalis*, RMNH.AVES.90657, adult female, mounted skin. Loc.: Timor, [Indonesia], x.1829. Leg.: S. Müller.

Syntype for *longipes*, paralectotype for *orientalis*, RMNH.AVES.90658, adult male, mounted skin. Loc.: Latakou [Dithakong], Cap, South Africa. Leg.: Verreaux.

Temminck and Schlegel (1849) mentioned having received specimens from South Africa, Java, Timor, Borneo, Celebes, and Japan. Dekker et al. (2001: 209–210) designated the lectotype to fix *orientalis* to Japan.

The so-called ‘Dernière Expedition’ (see lectotype) refers to a shipment which arrived in Leiden on 31 August 1829 and contained Von Siebold’s main collection: 827 specimens of 188 species (Dekker et al. 2001: 202).

Kuhl and Van Hasselt collected together on Java from December 1820 until September 1821. Forsten stayed in the Pagouat area between September and November 1841 and in the area around Gorontalo from September 1841 until April 1842. Müller visited Borneo from 28 July 1836 to 17 December 1836.

In this catalogue we follow Holthuis and Sakai (1970) over Mlíkovský (2012b) for the year of publication. This can have consequences on the status and selection of type specimens for this name if the plate was published prior to the description in the livraison. Taxa based upon holotypes are not affected but will differ where the name is based upon a series of specimens.

***Charadriusalbiceps*** Temminck, 1832: livr. 89, pl. 526.

= *Vanellusarmatus* (Burchell, 1822).

Holotype, RMNH.AVES.87513, adult male, mounted skin. Loc.: “Cafrerie” [= South Africa]. Leg.: -.

Holotype by monotypy.

***Lobi-vanellusinornatus*** Temminck & Schlegel, 1849: 106, pl. 63.

= *Vanelluscinereus* (Blyth, 1842).

Holotype, RMNH.AVES.87514, immature, mounted skin. Loc.: Japan. Leg.: Ph.F.B. von Siebold.

Holotype by monotypy. The description on page 106 appeared in 1849, the publication date of pl. 63 is unknown (Holthuis and Sakai 1970). For more information on Siebold’s collection see also Dekker et al. (2001).

In this catalogue we follow Holthuis and Sakai (1970) over Mlíkovský (2012b) for the year of publication. This can have consequences on the status and selection of type specimens for this name if the plate was published prior to the description in the livraison. Taxa based upon holotypes are not affected but will differ where the name is based upon a series of specimens.

***Charadriusmelanopteroides*** “Temminck” Schlegel, 1865a: 63.

= *Vanelluslugubris* (Lesson, 1826).

Syntype, RMNH.AVES.87521, adult female, mounted skin. Loc.: Elmina, Côte d’Or [= Ghana]. Leg.: H.S. Pel.

Syntype, RMNH.AVES.87522, adult female, mounted skin. Loc.: Elmina, Côte d’Or [= Ghana]. Leg.: H.S. Pel.

Syntype, RMNH.AVES.87523, adult, sex unknown, mounted skin. Loc.: Elmina, Côte d’Or [= Ghana]. Leg.: C.J.M. Nagtglas, 1862.

Syntype, RMNH.AVES.87524, adult, sex unknown, mounted skin. Loc.: Elmina, Côte d’Or [= Ghana]. Leg.: C.J.M. Nagtglas, 1862.

Schlegel (1865a) published this manuscript name by Temminck in the synonymy of *Vanellusinornatus* Swaison. Therefore, only the specimens available to Temminck are considered part of the type series.

***Vanelluscucullatus*** Temminck, 1830: livr. 85, pl. 505.

= *Vanellusmacropterus* (Wagler, 1827).

Syntype, RMNH.AVES.87518, adult female, mounted skin. Loc.: Sumatra, [Indonesia]. Leg.: -.

Syntype, RMNH.AVES.87519, adult male, mounted skin. Loc.: Timor, [Indonesia], [14.x.1828–1829]. Leg.: S. Müller.

Syntype, RMNH.AVES.87520, adult, sex unknown, skeleton. Loc.: Java, [Indonesia], [xii.1820–ix.1821]. Leg.: H. Kuhl and J.C. van Hasselt.

Kuhl and Van Hasselt collected on Java from December 1820 until September 1821. Müller visited Timor from 14 October 1828 until late 1829.

#### ﻿Jacanidae

***Parragallinacea*** Temminck, 1828: livr. 78, pl. 464.

= *Irediparragallinacea* (Temminck, 1828).

Syntype, RMNH.AVES.87489, adult male, mounted skin. Loc.: Menado, Celebes [= Sulawesi], [Indonesia], [ix–xi.1841]. Leg.: C.G.C. Reinwardt.

Syntype, RMNH.AVES.87490, adult, sex unknown, mounted skin. Loc: Menado, Celebes [= Sulawesi], [Indonesia], [ix–xi.1841]. Leg.: C.G.C. Reinwardt.

Reinwardt visited the Menado area between 11 October and 11 November 1821.

#### ﻿Scolopacidae

***Tringaplatyrhincha*** Temminck, 1815b: 616.

= *Calidrisfalcinellus* (Pontoppidan, 1763).

Temminck (1815b) based his description on a single specimen in the MNHN, which is not mentioned by Voisin and Voisin (2012).

***TringaBonapartei*** Schlegel, 1844: 89.

= *Calidrisfuscicollis* (Vieillot, 1819).

Holotype, RMNH.AVES.87546, adult, sex unknown, mounted skin. Loc.: ‘Amerique’. Leg.: C.L. Bonaparte.

***Tringaalbescens*** Temminck, 1821: livr. 7, pl. 41, fig. 2.

= *Calidrisruficollis* (Pallas, 1776).

Following Dickinson et al. (2022) (see also Introduction of this catalogue under “What is new in this 2023 version”: 14) the specimen on pl. 41, fig. 2 is the holotype since the text that appeared later is irrelevant regarding type status. Temminck (1821) did not indicate in which collection the illustrated specimen was housed. Jansen (2018: 350) listed MNHN-ZO-2014-473 and MNHN-ZO-2014-474 from Western Australia collected by Levillain in 1801 as syntypes, one of which is assumed to be the holotype. Temminck published this name in 1821, not 1824 as in Jansen (2018: 350).

***Tringacrassirostris*** Temminck & Schlegel, 1849: 107, pl. 64.

= *Calidristenuirostris* (Horsfield, 1821).

Syntype, RMNH.AVES.87526, adult male, mounted skin. Loc.: Japan. Leg.: Ph.F.B. von Siebold.

Syntype, RMNH.AVES.87527, adult, sex unknown, mounted skin. Loc.: Japan. Leg.: Ph.F.B. von Siebold.

Syntype, RMNH.AVES.87528, adult, sex unknown, mounted skin. Loc.: Japan. Leg.: H. Bürger.

Syntype, RMNH.AVES.87529, adult male, mounted skin. Loc.: Japan. Leg.: H. Bürger.

Syntype, RMNH.AVES.87530, adult female, mounted skin. Loc.: Pontianak, Borneo, [Indonesia]. Leg.: P.M. Diard.

Syntype, RMNH.AVES.87531, adult female, mounted skin. Loc.: Java, [Indonesia]. Leg.: H. Kuhl and J.C. van Hasselt.

Syntype, RMNH.AVES.87532, adult male, mounted skin. Loc.: Java, [Indonesia]. Leg.: H. Kuhl and J.C. van Hasselt.

Syntype, RMNH.AVES.87533, adult, sex unknown, skeleton. Loc.: Java, [Indonesia]. Leg.: H. Kuhl and J.C. van Hasselt.

Syntype, RMNH.AVES.87534, adult, sex unknown, skeleton. Loc.: Java, [Indonesia]. Leg.: H. Kuhl and J.C. van Hasselt.

According to Schlegel (1864d), RMNH.AVES.87526 (fig. 1), RMNH.AVES.87529 (fig. 2) and RMNH.AVES.87528 (fig. 3) are the depicted specimens of pl. 64.

In this catalogue we follow Holthuis and Sakai (1970) over Mlíkovský (2012b) for the year of publication. This can have consequences on the status and selection of type specimens for this name if the plate was published prior to the description in the livraison. Taxa based upon holotypes are not affected, but will differ where the name is based upon a series of specimens.

***ScolopaxWilsonii*** Temminck, 1826: livr. 68, text to pl. 403.

= *Gallinagodelicata* (Ord, 1825).

Temminck (1826) published this name in a footnote to the text with pl. 403. His description was based on the description of the “Snipe” by Wilson (1812: 18; pl. 47, fig. 1). There were no specimens in the RMNH available to Temminck at that time.

***Scolopaxgallinagoides*** Schlegel, 1864d: 6.

= *Gallinagodelicata* (Ord, 1825).

Holotype, RMNH.AVES.87535, adult, sex unknown, mounted skin. Loc.: Mexico. Leg.: 1857.

***ScolopaxLamotti*** Baillon, 1834: 71.

= *Gallinagogallinagogallinago* (Linnaeus, 1758).

Syntype, RMNH.AVES.258639, adult male, mounted skin. Loc.: Abbeville, France. Leg.: J.F.E. Baillon.

Syntype, RMNH.AVES.258640, adult female, mounted skin. Loc.: Abbeville, France. Leg.: J.F.E. Baillon.

Syntype, RMNH.AVES.258641, adult male, mounted skin. Loc.: Abbeville, France. Leg.: J.F.E. Baillon.

Gouraud (2015: 149) mentioned a single specimen sent to Temminck, although Schlegel (1864d: 5) listed three specimens as types of *lamotti*.

***ScolopaxPygmaea*** Baillon, 1834: 71.

= *Gallinagogallinagogallinago* (Linnaeus, 1758).

Syntype, RMNH.AVES.258642, adult male, mounted skin. Loc.: Abbeville, France. Leg.: J.F.E. Baillon.

According to Gouraud (2015: 149), Baillon (1834) based his description on two specimens. Schlegel (1864d: 6) listed RMNH.AVES.258642 as one of the types of *Pygmaea*.

***Gallinagomegala*** Swinhoe, 1861: 343.

= *Gallinagomegala* Swinhoe, 1861.

Paralectotype, RMNH.AVES.87536.

Warren (1966: 181) incorrectly referred to a specimen in the NHM (NHMUK 1896.1.1.142) as the holotype. Swinhoe refers to a specimen sent to and described by Blyth and to “*a* specimen” (not “*the* specimen”) he will send to the NHM (which is the specimen listed by Warren as holotype). The listing by Warren as holotype does, however, constitute a lectotype designation.

***Gallinagonemoricola*** Hodgson, 1836a: 8.

= *Gallinagonemoricola* Hodgson, 1836.

Syntype, RMNH.AVES.258643, adult, sex unknown, mounted skin. Loc.: Nepal. Leg.: B.H. Hodgson.

Hodgson (1836a) did not give much or any information on the specimen labels such as collecting date. Hence it is in most cases uncertain whether specimens collected and described by him are indeed types. The NHM has four syntypes (Warren 1966: 201).

***Gallinagonobilis*** P.L. Sclater, 1856b: 31.

= *Gallinagonobilis* P.L. Sclater, 1856.

Possible syntype, RMNH.AVES.258647, adult male, mounted skin. Loc.: Bogota, Colombia. Leg.: Verreaux, 1863.

According to Schlegel (1864d: 9) RMNH.AVES.258647 is one of the types of *Gallinagonobilis*, but this seems very unlikely. Sclater based his description on two specimens: one in the NHM collected by Stevens and a specimen sent to Sclater by Verreaux. Warren (1966: 208) listed the Verreaux specimen (NHMUK 1891.10.20.547) as syntype and the specimen by Stevens as missing.

***Spilurasolitariajaponica*** Bonaparte, 1856b: 579, 1024.

= *Gallinagosolitariajaponica* (Bonaparte, 1856).

Holotype, RMNH.AVES.87545, adult, sex unknown, mounted skin. Loc.: Japan. Leg.: H. Bürger.

Holotype by monotypy. Bonaparte (1856b) gave this new name to a single specimen listed in Temminck and Schlegel (1849: 112, pl. 64) as *Scolopaxsolitaria*.

***Gallinagosolitaria*** Hodgson, 1831: 238.

= *Gallinagosolitariasolitaria* Hodgson, 1831.

Possible syntype, RMNH.AVES.258646, adult male, mounted skin. Loc.: Nepal. Leg.: -.

Hodgson (1831) did not give much or any information on the specimen labels such as collecting date. Hence it is often uncertain whether specimens collected and described by him are indeed types. According to Schlegel (1864d: 16) RMNH.AVES.258646 is one of the types of *Gallinagosolitaria*. At least nine other syntypes are in the NHM (Warren 1966: 275).

***Scolopaxstenura*** Bonaparte, 1831: 335.

= *Gallinagostenura* (Bonaparte, 1831).

Syntype, RMNH.AVES.87537, adult male, mounted skin. Loc.: Buitenzorg [= Bogor], Java, [Indonesia], xi.1826. Leg.: H. Boie.

Syntype, RMNH.AVES.87538, adult male, mounted skin. Loc.: Buitenzorg [= Bogor], Java, [Indonesia], 10.xii.1826. Leg.: H. Boie.

Syntype, RMNH.AVES.87539, adult, sex unknown, mounted skin. Loc.: Buitenzorg [= Bogor], Java, [Indonesia], xii.1826. Leg.: H. Boie.

Syntype, RMNH.AVES.87540, adult, sex unknown, mounted skin. Loc.: Buitenzorg [= Bogor], Java, [Indonesia], xii.1826. Leg.: H. Boie.

Syntype, RMNH.AVES.87541, adult, sex unknown, skeleton. Loc.: Java, [Indonesia]. Leg.: H. Kuhl and J.C. van Hasselt.

Syntype, RMNH.AVES.87542, adult, sex unknown, skeleton. Loc.: Java, [Indonesia]. Leg.: H. Kuhl and J.C. van Hasselt.

Bonaparte (1831) listed *Scolopaxstenura* twice, both as the MS name of Kuhl as well as of Temminck. The cover page of volume 4 of the ‘Annali di Storia Naturale Bologna’ gives 1830, but publication of pp. 303–390 is generally referred to as 1831.

Aimassi et al. (2020: 81) listed a possible syntype in MZUT (MZUT Av3021).

***GallinagoBiclavus*** Hodgson, 1837b: 490.

***Gallinagoheterura*** Hodgson, 1836a: 8.

= *Gallinagostenura* (Bonaparte, 1831).

Syntype, RMNH.AVES.258644, adult, sex unknown, mounted skin. Loc.: Nepal. Leg.: B.H. Hodgson.

Syntype, RMNH.AVES.258645, adult, sex unknown, mounted skin. Loc.: Nepal. Leg.: B.H. Hodgson.

Hodgson did not give much or any information on the specimen labels such as collecting date. Hence it is in most cases uncertain whether specimens collected and described by him are indeed types. The NHM has five syntypes of *Gallinago Biclavus* and *heterura* (Warren 1966: 128).

***Scolopaxgigantea*** Temminck, 1826: livr. 68, pl. 403.

= *Gallinagoundulatagigantea* (Temminck, 1826).

Syntype, RMNH.AVES.87543, adult male, mounted skin. Loc.: [Itararé, São Paulo], Brazil. Leg.: J. Natterer, [1820].

Syntype, RMNH.AVES.87544, adult [female], sex unknown, mounted skin. Loc.: [Itararé, São Paulo], Brazil. Leg.: J. Natterer, [ii.1821].

See Schifter et al. (2007: 128) for collecting data. Schifter et al. (2007: 128) also stated that Natterer collected a total of 13 specimens. They confirmed the presence of five syntypes in the NMW as mentioned in the original description (NMW 39316–39319, 65865), and mentioned that other syntypes went to the museums in Bremen and the NHM.

According to Schlegel (1864d: 8) RMNH.AVES.87543 is the depicted specimen on pl. 403.

***Numeniusnasicus*** Temminck, 1840: 393.

***Numeniusmajor*** Temminck & Schlegel, 1844: 110.

= *Numeniusarquataorientalis* Brehm, 1831.

Syntype for *nasicus* and *major*, RMNH.AVES.87552, adult, sex unknown, mounted skin. Loc.: [West-]Sumatra, [Indonesia]. Leg.: S. Müller, [1833–1835].

Syntype for *nasicus*, RMNH.AVES.87553, adult, sex unknown, mounted skin. Loc.: Sumatra, [Indonesia]. Leg.: A.H. von Henrici, [1820–1826].

Temminck (1840) described skins from Sumatra and Borneo. No specimens from Borneo collected prior to 1840 are present in the RMNH. Müller visited West-Sumatra between 1833 and 1835. H.A. von Henrici arrived in Indonesia in October 1820 and returned to The Netherlands in 1838 in which year (part of) his collection (mainly from Borneo) entered the RMNH. He served in Sumatra sometime between 1820 and 1826.

There are also several specimens in the RMNH collected by Von Siebold and Bürger which were present at the time of description of *major*. But although Temminck and Schlegel refer to it as the “courlis du Japon” [Curlew from Japan], they don’t specifically mention any material from Japan. So, these specimens are not part of the type series.

***Numeniusbrevirostris***Lichtenstein 1823: 75.

***Numeniushemirhynchus*** “Temminck” Schlegel, 1864b: 101.

= *Numeniusborealis* (Forster, 1772).

Syntype, RMNH.AVES.87554, adult male, mounted skin. Loc.: Brazil. Leg.: J. Natterer.

This specimen was erroneously listed under “*Numeniushemirhynchus*Bonaparte (1856b: 597) in Van den Hoek Ostende et al. (1997: 76), a name which is more frequently referred to in the literature but is not listed in the index on p. 597 or anywhere else. The quotation on p. 597 refers to *Numeniusmelanorhynchus* Bonaparte, a nomen nudum.

It was Schlegel (1864b: 101) who introduced *hemirhynchus* “Temm”.

***Numeniusminor*** Müller, 1841: 110.

= *Numeniusminutus* Gould, 1841.

Holotype, RMNH.AVES.87555, adult male, mounted skin. Loc.: Amboine [= Ambon], [Indonesia]. Leg.: S. Müller [iii.1828–20.iv.1828].

Holotype by monotypy. Müller (1841) wrote only having collected a single male. Müller visited Ambon from late March until 20 April 1828.

***Phalaropusfimbriatus*** Temminck, 1825 livr. 62, pl. 370.

= *Phalaropustricolor* (Vieillot, 1819).

According to Temminck (1825), two syntypes are in the MNHN (not listed in Voisin and Voisin 2012).

***Tringaleucoptera*** Gmelin, 1789: 678.

***Tringapyrrhetraea*** Forster & Lichtenstein, 1844: 174.

= *Prosobonialeucoptera* (Gmelin, 1789).

Syntype, RMNH.AVES.87556, adult, sex unknown, mounted skin. Loc.: Tahiti, [French Polynesia], [1772–1775]. Leg.: -. [mount attributed to A. Sparrman, J. Cook’s second voyage].

Extinct. The origin of the only known specimen of this species in the RMNH was traced back until 1848 but based on its taxidermy could be attributed to the 2^nd^ circumnavigation commanded by James Cook (1772–1775) (Jansen et al. 2021a, 2021b). See also Cibois et al. (2012).

***ScolopaxRochussenii*** Schlegel, 1866c: 254.

= *Scolopaxrochussenii* Schlegel, 1866.

Holotype, RMNH.AVES.87557, adult male, relaxed mount. Loc.: Obi [Indonesia], 19.viii.1862. Leg.: H.A. Bernstein.

***ScolopaxRosenbergii*** Schlegel, 1871b: 54.

= *Scolopaxrosenbergii* Schlegel, 1871.

Holotype, RMNH.AVES.87558, adult female, relaxed mount. Loc.: Hattam, Arfak mountains, [Papua], [Indonesia], 20.iv.1870. Leg.: C.B.H. von Rosenberg.

***Totanuspulverulentus*** Müller, 1842: 153.

= *Tringabrevipes* (Vieillot, 1816).

Syntype, RMNH.AVES.87547, adult, sex unknown, mounted skin. Loc.: Timor, [Indonesia]. Leg.: S. Müller.

Syntype, RMNH.AVES.87548, adult male, mounted skin. Loc.: Timor, [Indonesia]. Leg.: S. Müller.

Syntype, RMNH.AVES.87549, adult male, mounted skin. Loc.: Timor, [Indonesia], ii.1829. Leg.: S. Müller.

Syntype, RMNH.AVES.87550, adult female, mounted skin. Loc.: Atapoepoe, Timor, [Indonesia], iii.1829. Leg.: S. Müller.

Syntype, RMNH.AVES.87551, adult female, mounted skin. Loc.: Atapoepoe, Timor, [Indonesia], iii.1829. Leg.: S. Müller.

Müller (1842) described this new species in a footnote to his report of a collecting trip in November 1828 from Kupang to Manikie (Timor). Strictly speaking, only the specimens collected during this trip should be types. However, as the report was not published until 1842 all material collected and seen by the author before 1842 is considered part of the type series. A specimen collected by Müller on Borneo is excluded because there is no reference to Borneo material.

***Falcinelluscursorius*** Temminck, 1830: livr. 86, pl. 510.

= not applicable, artefact.

Lectotype, RMNH.AVES.87525, adult, sex unknown, relaxed mount. Loc.: “River Gamtos”, [South Africa]. Leg.: Levaillant? Ex: Museum Raye van Breukelerwaert.

This name is based on an artefact which is a modified *Calidrisferruginea* (Pontoppidan, 1763) (Schlegel 1844: 98).

Temminck (1830) based his description on two specimens, RMNH.AVES.87525 and an immature specimen described as Ærolia varia by Vieillot and Oudart (1843: 89, pl. 231) in the MNHN. RMNH.AVES.87525 is therefore not the holotype as listed in Van den Hoek Ostende et al. (1997: 73) but has now to be considered lectotype.

#### ﻿Glareolidae

***Glareolalactea*** Temminck, 1820: 503.

= *Glareolalactea* Temminck, 1820.

Possible syntype, RMNH.AVES.87562, adult, sex unknown, mounted skin. Loc.: “Ganges”, Bengale. Leg.: -.

Possible syntype, RMNH.AVES.87563, adult, sex unknown, mounted skin. Loc.: Bengale. Leg.: -.

Temminck (1820) based his description on two specimens in the MNHN, which are not listed by Voisin and Voisin (2012). As the information on the RMNH specimens fits Temminck’s description, both MNHN specimens might have been exchanged with the RMNH. However, Gouraud (in litt., 28 and 29 October 2020) claims a possible syntype in the MLC (MLC.2011.0.918). Hence, we list the specimens here as possible syntypes.

***Glareolanuchalisliberiae*** Schlegel, 1881: 58.

***Glareolamegapoda*** Büttikofer, 1885: 233.

= *Glareolanuchalisliberiae* Schlegel, 1881.

Lectotype for *liberiae*, syntype for *megapoda*, RMNH.AVES.87564, adult female, skin. Loc.: Marfa River, Liberia, 28.i.1881. Leg.: J. Büttikofer and C.F. Sala (syntype for *megapoda*).

Syntype for *megapoda*, paralectotype for *liberiae*, RMNH.AVES.258649, adult female, skin. Loc.: Bavia, St. Paul’s River, Liberia, 26.i.1880. Leg.: J. Büttikofer and C.F. Sala (10).

Syntype for *megapoda*, paralectotype for *liberiae*, RMNH.AVES.258650, adult female, skin. Loc.: Bavia, St. Paul’s River, Liberia, 30.i.1880. Leg.: J. Büttikofer and C.F. Sala (23).

Syntype for *megapoda*, paralectotype for *liberiae*, RMNH.AVES.258651, adult male, skin. Loc.: Bavia, St. Paul’s River, Liberia, 15.iii.1880. Leg.: J. Büttikofer and C.F. Sala (no. 71).

Syntype for *megapoda*, paralectotype for *liberiae*, RMNH.AVES.258652, adult male, skin. Loc.: Bendo, Fisherman’s Lake, Liberia, 01.xii.1880. Leg.: J. Büttikofer and C.F. Sala (no. 213).

Syntype for *megapoda*, paralectotype for *liberiae*, RMNH.AVES.258654, adult female, skin. Loc.: Bavia, St. Paul’s River, Liberia, 30.i.1880. Leg.: J. Büttikofer and C.F. Sala (21).

Syntype for *megapoda*, paralectotype for *liberiae*, RMNH.AVES.258655, adult female, skin. Loc.: Bavia, St. Paul’s River, Liberia, 30.i.1880. Leg.: J. Büttikofer and C.F. Sala (22).

Syntype for *megapoda*, paralectotype for *liberiae*, RMNH.AVES.258656, adult male, skin. Loc.: Bavia, St. Paul’s River, Liberia, 30.i..1880. Leg.: J. Büttikofer and C.F. Sala (no. 20).

Syntype for *megapoda*, paralectotype for *liberiae*, RMNH.AVES.258657, adult male, skin. Loc.: Bavia, St. Paul’s River, Liberia, 20.iii.1880. Leg.: J. Büttikofer and C.F. Sala (no. 196).

Syntype for *megapoda*, paralectotype for *liberiae*, RMNH.AVES.258658, adult female, skin. Loc.: Bavia, St. Paul’s River, Liberia, 21.iii.1880. Leg.: J. Büttikofer and C.F. Sala (no. 198).

Syntype for *megapoda*, paralectotype for *liberiae*, RMNH.AVES.258659, adult male, skin. Loc.: Bendo, Fisherman’s Lake, Liberia, 04.xii.1880. Leg.: J. Büttikofer and C.F. Sala (no. 218).

Syntype for *megapoda*, paralectotype for *liberiae*, RMNH.AVES.258660, adult female, skin. Loc.: Bendo, Fisherman’s Lake, Liberia, 04.xii.1880. Leg.: J. Büttikofer and C.F. Sala (no. 219).

Syntype for *megapoda*, paralectotype for *liberiae*, ZMA.AVES.24122, adult female, skin. Loc.: Hotehu(?), Fisherman’s Lake, Liberia, 01.xii.1880 [not 1886]. Leg.: J. Büttikofer and C.F. Sala. (no. 3).

Syntype only for *megapoda*, RMNH.AVES.180164, eggs (2). Loc.: Bavia, St. Paul’s River, Liberia, 15.iii.1880. Leg.: J. Büttikofer and C.F. Sala.

Schlegel (1881) had a small series available but did not designate a (holo)type. Van den Hoek Ostende et al. (1997: 77) were in error to list RMNH.AVES.87564 as holotype. By doing so they unintentionally selected a lectotype for *liberiae* Schlegel.

The name *Glareolamegapoda* was first introduced by G.R. Gray (1844: 62) as a nomen nudum, used subsequently as *Glareolamegapodia* by Finsch and Hartlaub (1870: 636) also lacking a description. The first formal description was given by Büttikofer (1885: 233), based on his own specimens collected in Liberia.

Sharpe (1896: 64) listed Gray’s specimen in the NHM (cat. *e*) as type. However, as this specimen is not part of Büttikofer’s type series, and Gray’s name is a nomen nudum as stated above, we do not consider Sharpe’s (lecto)type selection valid. Hence, the types for *megapoda* Büttikofer are listed here as syntypes instead of paralectotypes.

***CursoriusAfricanus*** Temminck, 1807: 175, description: 263.

= *Rhinoptilusafricanusafricanus* (Temminck, 1807).

Holotype, RMNH.AVES.87565, adult male, relaxed mount. Loc.: Namaqualand [South Africa]. Leg.: -.

In his description, Temminck (1807) does not refer to a collector. The original stand is lost but the replacement label written by Finsch mentions “probl. coll. by Levaillant (1783–1785)”. According to Rookmaaker (1989: 316) and Irwin (1963: 2; who restricted the type locality to “Pofadder, Great Bushmanland”) this specimen was collected by Levaillant.

***Cursoriusbicinctus*** Temminck, 1820: 515.

= *Rhinoptilusafricanusafricanus* (Temminck, 1807).

Possible holotype, RMNH.AVES.87566, adult, sex unknown, relaxed mount. Loc.: “Cap”, [South Africa]. Leg.: -.

Temminck (1820) based his description on a specimen collected by Levaillant “dans l’intérieur de l’Afrique”. The labels by Finsch which replace the original labels do neither mention Levaillant nor “l’intérieur de l’Afrique” but give “Cap” instead. Finsch refers to it as the “Type of *bicinctus*”.

***Cursoriuschalcopterus*** Temminck, 1824: livr. 50, pl. 298.

= *Rhinoptiluschalcopterus* (Temminck, 1824).

According to Temminck (1824) the holotype (by monotypy) is in the MNHN.

***Glareolaisabella*** Vieillot, 1816a: 69.

***Glareolagrallaria*** Temminck, 1820: 503.

= *Stiltiaisabella* (Vieillot, 1816).

Syntype, RMNH.AVES.87567, adult female, mounted skin. Loc.: “Australia” [error for Kupang, Timor, Indonesia]. Leg.: [Maugé, R., Baudin Expedition]. Ex: MNHN.

Jansen (2017: 476) identified this specimen as part of the material collected by René Maugé during the Baudin Expedition (1801–1804). It was collected near Kupang, West-Timor between 22 August and 13 November 1801.

Vieillot (1816a) based his description on specimens brought back from the Baudin’s expedition in the MNHN. It is highly likely that RMNH.AVES.87567 was still in Paris when Vieillot described *isabella*. Jansen (2017: 476) listed two specimens in the MNHN as part of the type series (A.C.13083 and 13084). So Voisin and Voisin (2012: 51) erred in listing MNHN A.C.13084 as holotype by monotypy. We do not consider this a valid lectotype designation.

Schifter et al. (2007: 133) listed a possible syntype for *grallaria* Temminck in the NMW (NMW 49262) from Banda, received from the RMNH in 1823. However, Temminck most likely based his *grallaria* on the same specimens as *isabella* Vieillot when they were still in MNHN (Voisin and Voisin 2012: 51). The specimen in the NMW is therefore not part of the type series.

#### ﻿Laridae

***Sternatenuirostris*** Temminck, 1823: livr. 34, pl. 202.

= *Anoustenuirostristenuirostris* (Temminck, 1823).

Syntype, RMNH.AVES.87575, adult, sex unknown, mounted skin. Loc.: ‘Mer de Australie’. Leg.: -.

According to the original description other syntypes are in the MNHN but are not listed in Voisin and Voisin (2011). This species breeds in the Seychelles. The indication ‘Mer de Australie’ written under the stand is an error.

***Sternaleucopareia*** Temminck, 1820: 746.

= *Chlidoniashybridahybrida* (Pallas, 1811).

Syntype, RMNH.AVES.87576, adult, sex unknown, mounted skin. Loc.: Hungary. Leg.: J. Natterer.

Schifter et al. (2007: 135–136) confirmed the presence of syntypes, also collected by Natterer in Hungary, in the NMW (NMW 9422, 20759, 53464, 53465). Natterer collected for the NMW throughout Europe between 1806 and 1817, before he left for Brazil.

***Hydrochelidonfluviatilis*** Gould, 1843: 140.

= *Chlidoniashybridajavanicus* (Horsfield, 1821).

Paralectotypes, RMNH.AVES.87577 and 87578.

Stone (1913: 138) designated the lectotype in the ANSP (ANSP 5004).

***Sternaleucoptera*** Temminck, 1815b: 483.

= *Chlidoniasleucopterus* (Temminck, 1815).

Syntype, RMNH.AVES.87579, adult, sex unknown, mounted skin. Loc.: Lake Geneva, Switzerland. Leg.: -.

Syntype, RMNH.AVES.87580, adult, sex unknown, mounted skin. Loc.: Lake Geneva, Switzerland. Leg.: -.

Aimassi et al. (2020: 82) listed a possible syntype in the MZUT (MZUT Av3166).

***Laruspoliocephalus*** Temminck, 1820: 780.

= *Chroicocephaluscirrocephaluscirrocephalus* (Vieillot, 1818).

Syntype, RMNH.AVES.207793, adult, sex unknown, mounted skin. Loc.: Brazil.

Syntype, RMNH.AVES.207795, adult, sex unknown, mounted skin. Loc.: Brazil.

Temminck (1820) published this name with a description in a footnote to *Larusridibundus*.

***Larustenuirostris*** Temminck, 1840: 478.

= *Chroicocephalusgenei* (Breme, 1839).

Lectotype, RMNH.AVES.87587, adult male, mounted skin. Loc.: Sicily, Italy, [ii.1830]. Leg.: F. Cantraine.

Temminck (1840) described two specimens. Only one could be found. Schlegel (1863i: 28) selected the lectotype.

***Larusmelanorhinchus*** Temminck, 1830: livr. 85, pl. 504.

= *Chroicocephalusphiladelphia* (Ord, 1815).

Holotype, RMNH.AVES.87593, adult, sex unknown, mounted skin. Loc.: Chile. Leg.: -.

Holotype by monotypy.

***Laruscapistratus*** Temminck, 1820: 785.

= *Chroicocephalusridibundus* (Linnaeus, 1766).

Lectotype, RMNH.AVES.87594, adult, sex unknown, mounted skin. Loc.: ‘Mer du Nord’, [Orkney], [United Kingdom]. Leg.: -.

Temminck (1820) based his description on several specimens (“mes individus”) from Orkney. Schlegel (1863i: 39) listed RMNH.AVES.87594 as the type for *capistratus*, hereby designating it the lectotype.

Both Orkney and Shetland are mentioned on the labels. As Temminck gave Orkney as provenance for his specimen, we follow this.

***LarusKittlitzii*** Schlegel, 1863i: 40.

= *Chroicocephalusridibundus* (Linnaeus, 1766).

Holotype, RMNH.AVES.207979, adult, sex unknown, mounted skin. Loc.: Macao, China. Leg.: R. Swinhoe.

Swinhoe (1860: 60; 1861: 345) published this name twice as a nomen nudum while describing his collections from China. The first publication of this name with a description is by Schlegel in the synonymy of *Larusschimperi* (= *ridibundus*) and was based on a single specimen. This name was published in the same year by Swinhoe with a description (1863: 428), but as Vol. 5, issue 4 of ‘Ibis’ was issued in October 1863 and the publication by Schlegel in August 1863, the latter has priority.

***Sylochelidonstrenuus*** Gould, 1846: 21.

= *Hydroprognecaspia* (Pallas, 1770).

Paralectotype, RMNH.AVES.209461.

According to Schlegel (1863i: 14) this is one of the types of *strenuus* Gould. Schlegel stated that RMNH.AVES.209461 was collected in New South Wales; however, the label gives Tasmania. Stone (1913: 138) selected the lectotype in the ANSP (ANSP 5037).

***LarusAudouinii*** Payraudeau, 1826: 462.

= *Ichthyaetusaudouinii* (Payraudeau, 1826).

Lectotype, RMNH.AVES.87581, adult, sex unknown, mounted skin. Loc.: Corsica [= Corse], [France], [1824–1826]. Leg.: B.C.M. Payraudeau.

The lectotype was selected by Voisin et al. (1998: 65).

***Larusichthyaëtusminor*** Schlegel, 1863i: 34.

= *Ichthyaetusichthyaetus* (Pallas, 1773).

Syntype, RMNH.AVES.87588, adult, sex unknown, mounted skin. Loc.: Bengal, India. Leg.: P.M. Diard and A. Duvaucel. Ex: MNHN.

Syntype, RMNH.AVES.87589, adult female, mounted skin. Loc.: Ganges, India. Ex: G.A. Frank.

***Larusleucophthalmus*** Temminck, 1825: livr. 62, pl. 366.

= *Ichthyaetusleucophthalmus* (Temminck, 1825).

Syntype, RMNH.AVES.87590, adult male, mounted skin. Loc.: Red Sea. Leg.: W.P.E.S. Rüppell.

Syntype, RMNH.AVES.87591, adult female, mounted skin. Loc.: Red Sea Leg.: W.P.E.S. Rüppell.

Syntype, RMNH.AVES.87592, adult, sex unknown, mounted skin. Loc.: Red Sea. Leg.: W.P.E.S. Rüppell.

Syntype, RMNH.AVES.206177, adult, sex unknown, mounted skin. Loc. Red Sea, [1823–1825]. Leg.: W.F. Hemprich and C. Ehrenberg. Ex: MfN, 1859.

Another syntype is in the NMW (NMW 894; Schifter et al. 2007: 133).

***Larusmelanocephalus*** Temminck, 1820: Man. D’Orn., ed. 2, II: 777.

= *Ichthyaetusmelanocephalus* (Temminck, 1820).

The original description was based on specimens in the NMW collected by Natterer (Schifter et al. 2007: 134; NMW 20804 and 20805).

***Larusmelanurus*** Temminck, 1828: livr. 77, pl. 459.

= *Laruscrassirostris* Vieillot, 1818.

Lectotype, RMNH.AVES.87582, adult female, mounted skin. Loc.: Japan. Leg.: Ph.F.B. von Siebold.

Paralectotypes, RMNH.AVES.87583–87586.

Schlegel (1863i: 8) selected the lectotype. For information on dating and on Siebold’s collection, see Holthuis and Sakai (1970), Dekker et al. (2001), and Morioka et al. (2005). Two birds (juvenile and adult) collected by Von Siebold were sent to the NMW in 1841.

***Sternafuligula*** Lichtenstein (in Forster and Lichtenstein) 1844: 276.

= *Onychoprionanaethetusantarcticus* (Lesson, R, 1831).

Syntype, RMNH.AVES.210496, adult male, mounted skin. Loc.: Red Sea, [1823–1825]. Leg.: W.F. Hemprich and C. Ehrenberg. Ex: MfN.

Lichtenstein (in Forster and Lichtenstein 1844) mentioned that *Sternafuliginosa* Linnaeus consisted of two species. He applied his new name *fuligula* to the taxon from the Red Sea based on specimens in the MfN.

***Sternamelanogaster*** Temminck, 1827: livr. 73, pl. 434.

= *Sternaacuticauda* Gray, 1831.

Lectotype, RMNH.AVES.87603, adult, sex unknown, mounted skin. Loc.: “Mer des Indes”. Leg.: -.

Paralectotype, RMNH.AVES.87604.

Schlegel (1863j: 21) selected the lectotype. According to the original description other paralectotypes are in the MNHN (not listed in Voisin and Voisin 2011) and London (collection unknown). Plate 434 was published in livraison 73 (Dickinson 2001). The text, however, does not give the livraison number and is printed on the backside of *Sternamelanauchen* which was published in livraison 72.

The name *Sternamelanogaster* Temminck, 1827, is preoccupied by its prior use as *Sternamelanogaster* “T[emminck]” Horsfield, 1824, as a substitute name for *SternaJavanica* Horsfield, 1821 (Dickinson 2001: 37). However, in our opinion, *SternaJavanica* Horsfield, 1821 is a nomen nudum. Hence its substitute name *Sternamelanogaster* “T[emminck]” Horsfield, 1824 is also a nomen nudum, making *Sternamelanogaster* Temminck, 1827 an available name with priority over *acuticauda* Gray, 1831.

***Sternaarctica*** Temminck, 1820: 742.

= *Sternaparadisaea* Pontoppidan, 1763.

Syntype, RMNH.AVES.210049, adult, sex unknown, mounted skin. Loc.: Europe. Leg.: -.

***Sternamelanauchen*** Temminck, 1827: livr. 72, pl. 427.

= *Sternasumatranasumatrana* Raffles, 1822.

Lectotype, RMNH.AVES.87605, adult, sex unknown, mounted skin. Loc.: Celebes [= Sulawesi], [Indonesia], [ix.1821–xii.1821]. Leg.: [C.G.C. Reinwardt].

According to the original description other syntypes are in the MNHN but not listed in Voisin and Voisin (2011). Schlegel (1863j: 28) selected the lectotype.

Reinwardt visited Sulawesi from September until December 1821.

***Sternanatalensis*** “Verreaux” Schlegel, 1863j: 22.

= *Sternulaalbifronsalbifrons* (Pallas, 1764).

Schlegel (1863j) published this manuscript name by Verreaux, associated with RMNH.AVES.210701 (ex Verreaux, from South Africa), in the synonymy of *Sternaminuta* (= *Sternulaalbifrons*). No subsequent use to validate this name has been found.

***SternaPusilla*** Temminck, 1840: 465.

= *Sternulaalbifronssinensis* (Gmelin, 1789).

Syntype, RMNH.AVES.87595, adult, sex unknown, mounted skin. Loc.: Java, [Indonesia]. Leg.: H. Kuhl and J.C. van Hasselt.

Syntype, RMNH.AVES.87596, adult male, mounted skin. Loc.: Borneo, [Indonesia], [28.vii.1836–17.xii.1836]. Leg.: S. Müller.

Syntype, RMNH.AVES.87597, adult female, mounted skin. Loc.: Borneo, [Indonesia], [28.vii.1836–17.xii.1836]]. Leg.: S. Müller.

Syntype, RMNH.AVES.87598, adult, sex unknown, mounted skin. Loc.: Pontianak, Borneo, [Indonesia]. Leg.: P.M. Diard.

Syntype, RMNH.AVES.87599, adult, sex unknown, mounted skin. Loc.: Pontianak, Borneo, [Indonesia]. Leg.: P.M. Diard.

Syntype, RMNH.AVES.87600, adult, mounted skin. Loc.: Timor, [Indonesia], xi.1829. Leg.: S. Müller.

Syntype, RMNH.AVES.87601, adult, sex unknown, mounted skin. Loc.: Timor, [Indonesia]. Leg.: S. Müller.

Müller visited Borneo between 28 July 1836 and 17 December 1836. Müller (1841:125, 373) found these birds in September 1836 between Tanjung-jawa and Kampong Rioeng on a sandbank in the river Barito where the Madoeit enters the Barito.

***Sternaaffinis*** Cretzschmar, 1827: 23, pl. 14.

= *Thalasseusbengalensisbengalensis* (Lesson, 1831).

Possible paralectotypes, RMNH.AVES.150650 and 150651.

Steinheimer (2005a: 241) listed both as possible paralectotypes. The lectotype was designated by Steinbacher (1949) and is in the SMF (SMF 12705). Paralectotypes are in the SMF and probably in the NHM (Steinheimer 2005a: 241).

***SternaBergii*** Lichtenstein, 1823: 80.

= *Thalasseusbergiibergii* (Lichtenstein, 1823).

Syntype, RMNH.AVES.87606, adult, sex unknown, mounted skin. Loc.: “Cap”, [South Africa]. Ex: MfN.

***Sternaressa*** “Müller” Schlegel, 1863j: 9.

= *Thalasseusbergiicristatus* (Stephens, 1826).

Syntype, RMNH.AVES.210960, adult female, mounted skin. Loc.: Timor, [Indonesia], xi.1828. Leg.: S. Müller.

Syntype, RMNH.AVES.210961, adult male, mounted skin. Loc.: Oetanata river, New Guinea, [Indonesia], [11–22] vi.1828. Leg.: S. Müller [Triton Expedition 1828].

Syntype, RMNH.AVES.210963, adult male, mounted skin. Loc.: Oetanata river, New Guinea, [Indonesia], [11–22] vi.1828. Leg.: S. Müller [Triton Expedition 1828].

Syntype, RMNH.AVES.210965, immature female, mounted skin. Loc.: [Oetanata river], New Guinea [Indonesia], [11–22.vi.1828]. Leg.: S. Müller [Triton Expedition 1828].

Syntype, RMNH.AVES.255183, skeleton, sex unknown. Loc.: Timor, [Indonesia, i.1829]. Leg.: S. Müller.

Syntype, RMNH.AVES.255973, skeleton, sex unknown. Loc.: New Guinea, [Indonesia], [v.-viii.1828]. Leg.: S. Müller [Triton Expedition 1828].

Schlegel (1863j) published this manuscript name by Müller as a synonym of *Sternapelecanoides* King, 1827. Müller (1841: 125) gave the name *Ressa* as the vernacular name used by the local people.

***SternaVelox*** Cretzschmar, 1827: 21.

= *Thalasseusbergiivelox* (Cretzschmar, 1827).

Possible syntype, RMNH.AVES.87607, adult male, mounted skin. Loc.: Red Sea. Leg.: W.P.E.S. Rüppell (= *T.b.velox*).

Syntype, RMNH.AVES.210918. adult female, mounted skin. Loc.: Cape, South Africa. Leg.: Van Horstock (= *T.b.bergii*).

Although listed as a syntype by Van den Hoek Ostende et al. (1997: 82), RMNH.AVES.87607 may not be a type since relevant collecting or acquisition dates are missing. See Steinheimer (2005a: 241).

As Cretzschmar (1827: 22) also referred to South African specimens in the RMNH collection, we have added RMNH.AVES.210918 from the Cape here.

***Sternabernsteini*** Schlegel, 1863j: 9.

= *Thalasseusbernsteini* (Schlegel, 1863).

Holotype, RMNH.AVES.87602, adult male, mounted skin. Loc.: Kaou, Halmahera, [Indonesia], 22.xi.1861. Leg.: H.A. Bernstein.

***Sternagalericulata*** Lichtenstein, 1823: 81.

= *Thalasseusmaximus* (Boddaert, 1783).

Syntype, RMNH.AVES.87608, adult male, mounted skin. Loc.: Brazil. Ex: MfN.

#### ﻿Stercorariidae

***Lestrisspinicauda*** Hardy, 1854: 657.

= *Stercorariuslongicauduslongicaudus* Vieillot, 1819.

Syntype, RMNH.AVES.87569, adult, sex unknown, mounted skin. Loc.: St. Helena. Ex: J. Hardy.

***Lestrispomarinus*** Temminck, 1815b: 514.

= *Stercorariuspomarinus* (Temminck, 1815).

Syntype, RMNH.AVES.87570, adult, sex unknown, mounted skin. Loc.: North Sea. Leg.: -.

Syntype, RMNH.AVES.87571, adult, sex unknown, mounted skin. Loc.: North Sea. Leg.: -.

Syntype, RMNH.AVES.87572, adult, sex unknown, mounted skin. Loc.: North Sea. Leg.: -.

Syntype, RMNH.AVES.87573, adult, sex unknown, mounted skin. Loc.: North Sea. Leg.: -.

Syntype, RMNH.AVES.87574, immature, mounted skin. Loc.: North Sea. Leg.: -.

#### ﻿Alcidae

***UriaeMandtii*** Lichtenstein in Mandt, 1822: 30.

= *Cepphusgryllemandtii* (Lichtenstein, 1822).

Syntype, RMNH.AVES.91009, adult, sex unknown, mounted skin. Loc.: Spitsbergen, [1821]. Leg.: W.M. Mandt. Ex: MfN.

According to J. Mlíkovský (in litt., 21 February 2008), RMNH.AVES.91009 was collected by G.M. Mandt on Spitsbergen [Svalbard] in 1821 and is one of three specimens listed under the name *mandtii* in Lichtenstein’s catalogue of the MfN (Lichtenstein 1854: 105). Two syntypes remained in the MfN of which one is still present (ZMB 14416). The third, RMNH.AVES.91009, went to the RMNH between 1854 and 1867. It is mentioned by Schlegel (1867a: 19) as specimen no. 9: “un des individus types de l’Uria Mandtii Lichtenstein, obtenu du Musée de Berlin”.

One year after the name was mentioned for the first time in Mandt’s dissertation, Lichtenstein gave another description of *mandtii* with reference to Mandt in his “Doublettenverzeichnis” (Lichtenstein 1823: 88). There is no indication whether the specimen listed there had indeed been sold or not. The total number of syntypes therefore remains unclear.

***Uriawumizusume*** Temminck, 1836: livr. 98, pl. 579.

***SynthliboramphusTemminckii*** Brandt, 1837: 347.

= *Synthliboramphuswumizusume* (Temminck, 1836).

Syntype, RMNH.AVES.87609, adult male, mounted skin. Loc.: Japan. Leg.: Ph.F.B. von Siebold.

Syntype, RMNH.AVES.87610, adult male, mounted skin. Loc.: Japan. Leg.: Ph.F.B. von Siebold.

Syntype, RMNH.AVES.87611, immature, sex unknown, mounted skin. Loc.: Japan. Leg.: Ph.F.B. von Siebold.

Syntype, RMNH.AVES.87612, adult, sex unknown, skeleton. Loc.: Japan. Leg.: Ph.F.B. von Siebold.

The year of publication of livraison 97, usually given as 1835 following Sherborn (1898: 488), has been corrected to 1836 by Mees (1994: 51) and confirmed by Dickinson (2001: 47). The year of publication of livraison 98 and 99 was subsequently corrected to 1836 by Dickinson (2001: 47), contra Sherborn, (1898), who gave 1835 for both livraisons.

Schifter et al. (2007: 141) listed another syntype for *wumizusume* in the NMW (NMW 69597), collected by Von Siebold, which was received in 1841.

Brandt’s name was published without description but with reference to “Temm. Planch. Col. tab. 579”. The same specimens are therefore types of both names.

### ﻿SPHENISCIFORMES

#### ﻿Spheniscidae

***Eudyptesschlegeli*** Finsch, 1876: 204.

= *Eudyptesschlegeli* Finsch, 1876.

Holotype, RMNH.AVES.87035, adult, sex unknown, mounted skin. Loc.: Macquarie Island, New Zealand. Leg.: -.

Holotype by monotypy. Finsch’s description (1876) is based on a single specimen (“indiv. No. 3, Schleg. in Mus. P.B.”).

### ﻿PROCELLARIIFORMES

#### ﻿Diomedeidae

***Diomedeabrachiura*** Temminck, 1828: livr. 75.

= *Phoebastriaalbatrus* (Pallas, 1769).

This name was first published by Temminck (1828) in the introduction to the genus *Albatros* (livr. 75). A full description followed in the text to pl. 456. Syntypes are in the MNHN and Japan.

For the date of publication of livraison 75, see Dickinson (2001: 46).

***Diomedeacauta*** Gould, 1841a: 177.

= *Thalassarchecautacauta* (Gould, 1841).

Paralectotype, RMNH.AVES.87009.

According to an acquisition list in the RMNH archives, Temminck received the specimen from Gould between 1 December 1840 and 1 March 1841. The lectotype was selected by Stone (1913: 138) and is in the ANSP (ANSP 4518).

***Diomedeamelanophris*** Temminck, 1828: livr. 77, pl. 456.

= *Thalassarchemelanophris* (Temminck, 1828).

Syntype, RMNH.AVES.87010, adult, sex unknown, mounted skin. Loc.: Pacific. Leg.: -.

Syntype, RMNH.AVES.87011, adult male, mounted skin. Loc.: St. Paul, Pacific, 19.v.1826. Leg.: H. Boie.

Syntype, RMNH.AVES.87012, adult male, mounted skin. Loc.: St. Paul, Pacific, 19.v.1826. Leg.: H. Boie.

Syntype, RMNH.AVES.87013, immature male, mounted skin. Loc.: St. Paul, Pacific, 19.v.1826. Leg.: -.

RMNH.AVES.87014 (cranium) and RMNH.AVES.88756 (skeleton), listed as types in Van den Hoek Ostende et al. (1997: 12), do not belong to the type series since Temminck did not mention any skeletal material.

The subsequent spelling *melanophrys* is an “unjustified emendation” (Art. 33.2.3., ICZN 1999), but was judged to be in prevailing usage e.g., by Dickinson (2003: 72) who therefore deemed *melanophrys* to be a justified emendation (Art. 33.2.3.1., ICZN 1999). Carlos and Voisin (2008: 130) proposed the conservation of the original spelling *Diomedeamelanophris*.

According to Temminck there should also be specimens from the Cape and Australia in the RMNH which are no longer present. Based on the collecting locality and date, RMNH.AVES.87013 must also have been collected by H. Boie.

Von Berlepsch (1908: 293) designated a specimen from the Cape, South Africa as lectotype. However, he did not specify a particular specimen making this lectotypification invalid as it does not conform to the regulations of the ICZN.

#### ﻿Hydrobatidae

***Procellarialeachii*** Temminck, 1820: 812.

= *Oceanodromaleucorhoaleucorhoa* (Vieillot, 1818).

Temminck (1820) had seen specimens in the NHM (from the Bullock Museum) and the collection of Baillon (now MLC) and knew of specimens in the MNHN and Laugier collection. The specimen in the MLC could not be found (Gouraud 2015: 147). The specimen in the NHM (NHMUK 1964.23.2) was erroneously listed by Warren (1966: 156) as holotype, which constitutes a lectotype designation.

#### ﻿Procellariidae

***Nectrischilensis*** Bonaparte, 1857: 202.

= *Ardennagrisea* (Gmelin, 1789).

Syntype, RMNH.AVES.87030, adult male, mounted skin. Loc.: Chile. Leg.: -.

***Procellariatenuirostris*** Temminck, 1836: livr. 99.

= *Ardennatenuirostris* (Temminck, 1836).

Syntype, RMNH.AVES.87033, adult male, mounted skin. Loc.: Japan. Leg.: Ph.F.B. von Siebold.

Syntype, RMNH.AVES.87034, adult, sex unknown, mounted skin. Loc.: Japan. Leg.: Ph.F.B. von Siebold.

There is no plate related to the description nor is the livraison mentioned underneath the text, which is generally considered to be livraison 99, following the description and illustration of *Procellarialeucomelas* Temminck, 1836: livr. 99, pl. 587.

The year of publication of livraison 98 and 99 was corrected to 1836 by Dickinson (2001: 46), contra Sherborn (1898) who gave 1835 for both livraisons.

***Procellarialeucomelas*** Temminck, 1836: livr. 99, pl. 587.

= *Calonectrisleucomelas* (Temminck, 1836).

Paralectotypes, RMNH.AVES.87016–87018.

The year of publication of livraison 98 and 99 was corrected to 1836 by Dickinson (2001), contra Sherborn (1898) who gave 1835 for both livraisons.

Voisin et al. (1997: 763) erroneously considered a syntype in the collection of the MNHN the holotype by monotypy (MNHN C.G.1996-1039). As this should be considered a lectotype selection, the RMNH specimens become paralectotypes. Another type specimen is in the NMW (NMW 53301; Schifter et al. 2007: 40).

Two crania (RMNH.AVES.87019 and 87020), listed as types in Van den Hoek Ostende et al. (1997: 13), do not belong to the type series since Temminck did not mention any skeletal material.

***Procellarianiveamajor*** Schlegel, 1863h: 15.

= *Pagodromaniveamajor* (Schlegel, 1863).

Syntype, RMNH.AVES.107018, mounted skin. Loc.: “South Pole”. Leg: J.S.C. Dumont d’Urville, Astrolabe and Zélée Expedition 1837–1842.

Syntype, RMNH.AVES.107020, mounted skin. Loc.: Antarctic Sea. Ex: G.A. Frank, 1863.

Bonaparte (1857: 192) published *Procellarianiveamajor* earlier without description making it a nomen nudum.

***Procellarianiveaminor*** Schlegel, 1863h: 16.

= *Pagodromaniveanivea* (Forster, 1777).

Syntype, RMNH.AVES.90981, mounted skin. Loc.: “South Pole”. Leg.-.

Syntype, RMNH.AVES.90982, mounted skin. Loc.: Antarctic Sea. Ex: G.A. Frank, 1863.

Bonaparte (1857: 192) published *Procellarianiveaminor* earlier without description making it a nomen nudum.

***Procellariaaterrima*** Bonaparte, 1857: 191.

= *Pseudobulweriaaterrima* (Bonaparte, 1857).

Paralectotype, RMNH.AVES.87021.

The lectotype was designated by Jouanin (1970: 55) and is in the MNHN (MNHN C.G.1995-263; Voisin et al. 1997: 761).

Bonaparte based his description on specimens in the MNHN and RMNH and referred to a MS name given by Verreaux. According to handwritten notes in his private copy of the ‘Conspectus Avium’, Verreaux considered *P.aterrima* and *Puffinuspacificus* to be the same species and applied the names to the wrong specimens (Jouanin 1970). To avoid further confusion, Jouanin (1970: 55) selected MNHN C.G.1995-263 as lectotype. Consequently, RMNH.AVES.87021, in Van den Hoek Ostende et al. (1997: 13) listed as syntype becomes a paralectotype.

According to Jouanin (1970: 55), MNHN C.G.1995-263 and RMNH.AVES.87021 are two of only four known specimens of this species.

***Procellariahasitata*** Kuhl, 1820a: 142.

***Procellarialeucocephalae*** Kuhl, 1820a: 142.

= *Pterodromahasitata* (Kuhl, 1820).

Holotype, RMNH.AVES.87022, adult, sex unknown, mounted skin. Loc.: Indian Ocean. Leg.: -.

Holotype by monotypy. Kuhl (1820a) described this species from a single specimen in the Bullock Museum bought by Temminck for the RMNH. Kuhl attributed this name to Forster, based on a manuscript name on the original drawing (“tab. 97”) of this specimen. He synonymised *Procellarialeucocephalae*, another Forster manuscript name (“tab. 98”), with *P.hasitata*. By doing so Kuhl inadvertently also published this manuscript name for the first time, not Forster and Lichtenstein (1844) as is commonly believed.

See also Schlegel (1863h: 13) and Hellmayr and Conover (1948: 76) who confirmed the presence of the type specimen in the RMNH.

***Procellariaincerta*** Schlegel, 1863h: 9.

= *Pterodromaincerta* (Schlegel, 1863).

Syntype, RMNH.AVES.87023, adult, sex unknown, mounted skin. Loc.: “Mers australes” [error]. Leg.: -.

Syntype, RMNH.AVES.87024, adult, sex unknown, mounted skin. Loc.: “New Zealand” [error]. Leg.: M. de Beligny.

Syntype, RMNH.AVES.87025, adult male, mounted skin. Loc.: “Australia” [error]. Ex: Maison Verreaux, 1863.

Syntype, RMNH.AVES.87026, chick, mounted skin. Loc.: “New Caledonia” [error]. Ex: Maison Verreaux, 1863.

All specimens Schlegel (1863h) listed in his description are still available. The type localities “Mers australes, côtes de la Nouvelle Zéelande, Mers de l’Australie, Nouvelle Calédonie” must be an error since *P.incerta* does not occur there but ranges through the South Atlantic Ocean, off the east coast of South America to the west coast of Africa, and breeds only on Tristan and Gough Islands of Tristan da Cunha. De Beligny possibly collected his specimen on the journey to or from New Zealand (in 1840 or 1845).

Some doubt remains about the taxonomic identity of the downy chick (G.O. Keijl, pers. comm., 31 October 2007).

***Procellarianeglecta*** Schlegel, 1863h: 10.

= *Pterodromaneglectaneglecta* (Schlegel, 1863).

Syntype, RMNH.AVES.87027, adult male, mounted skin. Loc.: Kermadec Islands [error for Meyer Islets] [Leg.: J. MacGillivray.] Ex: Maison Verreaux.

Syntype, RMNH.AVES.87028, adult female, mounted skin. Loc.: Sunday Island [error for Meyer Islets], vii.1854. [Leg.: J. MacGillivray.] Ex: Maison Verreaux, 1863.

Syntype, RMNH.AVES.87029, adult, sex unknown, mounted skin. Loc.: Kermadec Island [error for Meyer Islets], vii.1854. [Leg.: J. MacGillivray.] Ex: Maison Verreaux, 1863.

All specimens listed by Schlegel (1863h) are still available. Apparently, all of them were collected by John MacGillivray in July 1854. MacGillivray’s original journal shows that these specimens were collected on the Meyer Islets and not, as is commonly believed, on Raoul Island.

***Procellariaanglorum*** Temminck, 1820: 806.

= *Puffinuspuffinus* (Brünnich, 1764).

Syntype, RMNH.AVES.87031, adult, sex unknown, mounted skin. Loc.: Europe. Leg.: -.

Syntype, RMNH.AVES.87032, adult, sex unknown, mounted skin. Loc.: Europe. Leg.: -.

### ﻿CICONIIFORMES

#### ﻿Ciconiidae

***Anastomuslamelligerus*** Temminck, 1823: livr. 40, pl. 236.

= *Anastomuslamelligeruslamelligerus* Temminck, 1823.

Syntype, RMNH.AVES.87081, adult, sex unknown, mounted skin. Loc.: Senegal. Leg.: -.

According to the original description the second syntype, reported by Delalande from South Africa, is in the MNHN.

***CiconiaAbdimii*** Lichtenstein, 1823: 76.

= *Ciconiaabdimii* Lichtenstein, 1823.

Syntype, RMNH.AVES.87082, adult male, mounted skin. Loc.: Nubia. Leg.: Ehrenberg. Ex: M.H.C. Lichtenstein, MfN.

The specimen was received in exchange with the MfN in 1823.

***Dissouraneglecta*** Finsch, 1904: 94.

= *Ciconiaepiscopusneglecta* (Finsch, 1904).

Syntype, RMNH.AVES.87083, adult, sex unknown, mounted skin. Loc.: Java, [Indonesia]. Ex: Cabinet Temminck.

Syntype, RMNH.AVES.87084, adult, sex unknown, mounted skin. Loc.: Java, [Indonesia]. Ex: Cabinet Temminck.

Syntype, RMNH.AVES.87085, adult male, mounted skin. Loc.: Buitenzorg [= Bogor], Java, [Indonesia], xii.1826. Leg.: H. Boie.

Syntype, RMNH.AVES.87086, adult male, mounted skin. Loc.: Java, [Indonesia]. Leg.: 1870.

Syntype, RMNH.AVES.87087, adult, sex unknown, mounted skin. Loc.: Makassar, Celebes [= Sulawesi], [Indonesia]. Leg.: J.E. Teysmann [error for J.E. Teijsman], 1878.

Syntype, RMNH.AVES.87088, adult, sex unknown, mounted skin. Loc.: Minahassa, Celebes [= Sulawesi], [Indonesia]. Leg.: S.C.J.W. van Musschenbroek, 1878.

Syntype, RMNH.AVES.87089, immature, mounted skin. Loc.: Bugo, Soembawa [= Sumbawa], [Indonesia], xii.1879. Leg.: J.W. van Lansberge, v.1882.

Syntype, RMNH.AVES.87090, adult female, mounted skin. Loc.: Soewassoe, Celebes [= Sulawesi], [Indonesia], 16.vi.1863. Leg.: C.B.H. von Rosenberg.

Syntype, RMNH.AVES.87091, adult, sex unknown, mounted skin. Loc.: N. Celebes [= Sulawesi], [Indonesia]. Leg.: F. von Faber, 1883.

Syntype, RMNH.AVES.87092, adult, sex unknown, mounted skin. Loc.: Menado [= Manado], Celebes [= Sulawesi], [Indonesia]. Leg.: F. von Faber, 1883.

The species was first published in a determination key. Later, Finsch (1905) gave a full description according to which he had five specimens from Java, five from Celebes, and one from Sumbawa; one of the Java specimens seems to have been lost since.

Teijsmann’s specimen was most likely collected during the Celebes expedition of 1877, during which he stayed in the Makassar area in June, July, October, and December.

Two skeletons (RMNH.AVES.87093 and 87094) listed by Van den Hoek Ostende et al. (1997: 20) do not belong to the type series as Finsch did not mention any skeletal material.

***Ciconiaargala*** Temminck, 1824: livr. 51, pl. 301, nec Latham, 1790.

= *Leptoptiloscrumenifer* (Lesson, 1831).

Possible syntype, RMNH.AVES.234677, adult, sex unknown, mounted skin. Loc.: Africa. Ex: Cabinet Temminck.

Temminck (1824) caused confusion by naming this taxon from Africa *argala* and the one from Asia (see next entry) *marabou* which are both local names. However, Temminck mixed them up as he should have given *argala* to the Asian taxon and *marabou* to the African. See e.g., Bennett (1835: 274–276).

The name *argala* Temminck, 1824 (Africa) is preoccupied by *argala* Latham, 1790 (Africa and Asia), the latter being misapplied; *crumenifer* Lesson, 1831 is the next available name for the taxon from Africa.

Temminck referred to specimens from various parts of Africa in the collections of the MNHN, NMW, MfN, SMF, and RMNH. RMNH.AVES.234677 is listed here as a possible syntype as it originates from Temminck’s Cabinet which was established well before 1824. The name *argala* is, however, not mentioned under the stand. No type specimens are listed in Schifter et al. (2007).

***Ciconiamarabou*** Temminck, 1824: livr. 51, pl. 300.

= *Leptoptilosdubius* (Gmelin, 1789).

Syntype, RMNH.AVES.87095, adult, sex unknown, mounted skin. Loc.: Ganges, India. Leg.: -.

According to the original description other syntypes are in the MNHN. See also under *Ciconiaargala* Temminck, 1824.

***Ciconiacapillata*** Temminck, 1824: livr. 53, pl. 312.

= *Leptoptilosjavanicus* (Horsfield, 1821).

Syntype, RMNH.AVES.87096, adult male, mounted skin. Loc.: Java, [Indonesia]. Leg.: -.

Syntype, RMNH.AVES.87097, adult, sex unknown, mounted skin. Loc.: Java, [Indonesia]. Leg.: -.

According to the original description two syntypes (immature birds) are in the MNHN. Schifter et al. (2007: 51) listed a possible syntype in the NMW (NMW 47612) which arrived in April 1830.

***Tantaluslacteus*** Temminck, 1825: livr. 59, pl. 352.

= *Mycteriacinerea* (Raffles, 1822).

Syntype, RMNH.AVES.87098, adult male, mounted skin. Loc.: Java, [Indonesia]. Leg.: H. Kuhl and J.C. van Hasselt.

Syntype, RMNH.AVES.87099, adult female, mounted skin. Loc.: Java, [Indonesia]. Leg.: H. Kuhl and J.C. van Hasselt.

Syntype, RMNH.AVES.87100, immature male, mounted skin. Loc.: Java, [Indonesia]. Leg.: H. Kuhl and J.C. van Hasselt.

Kuhl and Van Hasselt collected on Java from December 1820 until September 1821. Two skeletons also collected by Kuhl and Van Hasselt are not included in the type series, since Temminck (1825) made no reference to skeletal material. One was sent to the NMW in September 1833.

### ﻿SULIFORMES

#### ﻿Anhingidae

***PlotusLevaillantii*** Temminck, 1807: 196.

= *Anhingarufarufa* (Daudin, 1802).

Syntype, RMNH.AVES.87036, immature female, mounted skin. Loc.: “Cap”, [South Africa]. Leg.: F. Levaillant.

Syntype, RMNH.AVES.87037, adult male, mounted skin. Loc.: “Cap”, [South Africa]. Leg.: F. Levaillant.

One specimen was sent to the NMW in 1821 (NMW 1821.LXXIII.60) and one in April 1830 (NMW 1830.VII.104).

Phalacrocoracidae

***CarboDesmarestii*** Payraudeau, 1826: 464.

= *Phalacrocoraxaristotelisdesmarestii* (Payraudeau, 1826).

Paralectotype, RMNH.AVES.87038.

The lectotype was selected by Voisin et al. (1998: 65) and is in the Museé Ornithologique Ch. Payraudeau, La Chaize-le-Vicomte, France (no. 00049). Another paralectotype is in the MLC (MLC.2010.0.73).

***Carbocapillatus*** Temminck & Schlegel, 1849: pl. 83.

***Carbofilamentosus*** Temminck & Schlegel, 1850: 129.

= *Phalacrocoraxcapillatus* (Temminck & Schlegel, 1849).

Lectotype, RMNH.AVES.87039, adult male, mounted skin. Loc.: Japan. Leg.: Ph.F.B. von Siebold.

Paralectotypes, RMNH.AVES.87040–87044, RMNH.AVES.107933 (= *Phalacrocoraxcarbohanedae* Kuroda, 1925).

This taxon was erroneously given two different names: on pls 83 and 83B it was referred to as *Carbocapillatus*, but in the text on p. 129 as *C.filamentosus*. According to Holthuis and Sakai (1970) both plates appeared in 1849 (not 1848 as erroneously stated by Dekker et al. 2001: 205), whereas the text was published in 1850. The priority of *C.capillatus* was first accepted by Dorst and Mougin (1979), who footnoted that Reichenbach (1850) and Bonaparte (1857) had both selected *capillatus*. See also Dekker et al. (2001: 205), Morioka et al. (2005), and Mlíkovský (2012b: 111).

Schlegel (1863k: 10) listed eight specimens: seven from Von Siebold and/or Bürger (of which six are still present in the RMNH), while the collector of the 8^th^ specimen is unknown. He referred to RMNH.AVES.87039 as “type du *Carbocapillatus*” and as the depicted specimen, hereby designating it as lectotype. RMNH.AVES.107933 has been identified as *Phalacrocoraxcarbohanedae* Kuroda, 1925. Two specimens collected by Von Siebold arrived in the NMW in 1841.

In this catalogue we follow Holthuis and Sakai (1970) over Mlíkovský (2012b) for the year of publication. This can have consequences on the status and selection of type specimens for this name if the plate was published prior to the description in the livraison. Taxa based upon holotypes are not affected but will differ where the name is based upon a series of specimens.

***Phalacrocoraxlugubris*** Rüppell, 1845: 134.

= *Phalacrocoraxlucidus* (Lichtenstein, 1823).

Paralectotype, RMNH.AVES.87045.

Steinbacher (1949: 103) designated the lectotype in the SMF (SMF 12 698).

***Phalacrocoraxmentalis*** Bonaparte, 1857: 175.

= *Phalacrocoraxmagellanicus* (Gmelin, 1789).

Holotype, RMNH.AVES.87046, immature, mounted skin. Loc.: Ile Magdalena, Malowines. Ex: G.A. Frank.

The locality under the stand is given as Ile Magdalena, with Malowines (= Falklands) added to it. However, Ile Magdalena is located in the Strait of Magellan, not in the Falklands (R. Woods, pers. comm., 26 February 2004).

***Carbograculus*** Temminck, 1820: 897.

= not applicable, see below.

Van den Hoek Ostende et al. (1997: 16) listed three syntypes which could be identified as *Phalacrocoraxsulcirostris* (Brandt, 1837) (RMNH.AVES. 87047 and 87048) and *P.olivaceusmexicanus* (Brandt, 1837) (RMNH.AVES.87049). However:

1. Temminck had already mentioned *Carbograculus* in the first edition of his ‘Manuel d’ornithologie’ (Temminck 1815b: 589);
2. In both editions the name is listed as given by Meyer. According to Ogilvie-Grant (1898: 364), *Carbograculus* Meyer & Wolf, 1810 is used in the way Linnaeus (1766: 217) did, as a younger synonym of *Pelecanusaristotelis* Linnaeus, 1761 (now *Phalacrocoraxaristotelis*).
3. Temminck referred in both editions to *C.graculus* Gmelin, 1788 (in 1815 for the immature bird, in 1820 for all specimens), which is the same taxon, *Phalacrocoraxaristotelis* (Linnaeus, 1761).

Considering this, it was obviously not Temminck’s intention to describe a new species. He applied the name *graculus* to the wrong specimens and listed the birds known as *P.aristotelis* under the name *Carbocristatus*. According to the Code (ICZN 1999, Art. 49) a name wrongly applied through misidentification is not available and therefore cannot have type specimens.

### ﻿PELECANIFORMES

#### ﻿Threskiornithidae

***Ibiscarunculata*** Rüppell, 1837: 49, pl. 19.

= *Bostrychiacarunculata* (Rüppell, 1837).

Paralectotypes, RMNH.AVES.90939–90941.

The lectotype was designated by Steinbacher (1949: 103) and is in SMF (SMF 12597). Paralectotypes are in SMF and NHM (Steinheimer 2005a: 236). According to correspondence in the RMNH archives (‘Angebotene Vögel aus Abyssinien, abgebildet in meiner Abyssinia-Fauna’), the RMNH specimens were sold by Rüppell to the museum between September and October 1837.

***Lampribissplendida*** Salvadori, 1903: 185.

= *Bostrychiaolivaceaolivacea* (Du Bus de Gisignies, 1838).

Holotype, RMNH.AVES.87101, adult male, skin. Loc.: Sofore-Place, Liberia, 11.vi.1880. Leg.: J. Büttikofer and C.F. Sala.

Holotype by monotypy.

***Ibisnippon*** Temminck, 1835: livr. 93, pl. 551.

***NipponiaTemminckii*** Reichenbach, 1850: xiv.

= *Nipponianippon* (Temminck, 1835).

Holotype for *nippon*, syntype for *Temminckii*, RMNH.AVES.87102, adult male, mounted skin. Loc.: Japan. Leg.: Ph.F.B. von Siebold.

Holotype by monotypy, as Temminck (1835) refers to a unique specimen. Temminck mentioned Von Siebold and a single specimen from Japan in the RMNH. RMNH.AVES.87102 fits best the bird depicted on pl. 551. A second specimen (RMNH.AVES.195123) was collected by Bürger and can therefore not be part of the type series. Neither are two skulls, listed as syntypes in Van den Hoek Ostende et al. (1997: 21).

Reichenbach (1850) did not give a full description on page xiv but referred to two plates in his “Iconographie” (Reichenbach 1836): “Ic. Av. t. 141. ic. 538” and “t. 149. ic. 2569”. The bird depicted in figure 538 of pl. 141 was obviously copied from Temminck’s ‘Planches Coloriées’. RMNH.AVES.87102 is therefore also a syntype of *NipponiaTemminckii* Reichenbach, 1850. The second syntype, the specimen depicted in figure 2569 of pl. 149, could not be traced (Dekker et al. 2001: 208–209). For more information on dating of texts and plates see, Dekker et al. (2001).

Van den Hoek Ostende et al. (1997: 22) erred in suggesting that the type material of *Ibisnippon* Temminck was also type material for *Ibistemmincki* Reichenow, 1877. Reichenow´s name is a new combination of Reichenbach´s name listed above (see Dekker et al. 2001).

***Plataleamajor*** Temminck & Schlegel, 1849: 119, pl. 75.

***Plataleajaponica*** Reichenow, 1877: 159 (nomen novum, in part).

= *Platalealeucorodialeucorodia* Linnaeus, 1758.

Holotype for *major*, syntype for *japonica*, RMNH.AVES.87105, adult male, mounted skin. Loc.: Japan. Leg.: H. Bürger.

Holotype by monotypy. Temminck and Schlegel (1849: 119) state that they received only one specimen of the two new Japanese species of *Platalea*.

Reichenow (1877) proposed the name *japonica* as a nomen novum for *P.major* Temminck & Schlegel, 1849 and *P.minor* Temminck & Schlegel, 1849, which he considered as a single species. Previously *japonica* was listed in synonymy of *minor* (e.g., Sharpe 1898: 51), but description and measurements comprise both taxa.

The description on page 119 as well as the plate appeared in 1849 (Holthuis and Sakai 1970). See also Mlíkovský (2012b) and Dekker et al. (2001).

***Plataleaminor*** Temminck & Schlegel, 1849: 120, pl. 76

***Plataleajaponica*** Reichenow, 1877: 159 (nomen novum, in part).

= *Plataleaminor* Temminck & Schlegel, 1849.

Holotype for *minor*, syntype for *japonica*, RMNH.AVES.87107, adult male, mounted skin. Loc.: Japan. Leg.: H. Bürger.

Holotype by monotypy, see also *Plataleamajor*. The description on page 120 as well as the plate appeared in 1849 (Holthuis and Sakai 1970). See also Mlíkovský (2012b) and Dekker et al. (2001).

Reichenow proposed the name *japonica* as a nomen novum for *P.major* Temminck & Schlegel, 1849 and *P.minor* Temminck & Schlegel, 1849, which he considered as a single species. Previously *japonica* was listed in synonymy of *minor* (e.g., Sharpe 1898: 51), but description and measurements comprise both taxa.

***Tantaluschalcopterus*** Temminck, 1830: livr. 86, pl. 511.

= *Plegadischihi* (Vieillot, 1817).

Lectotype, RMNH.AVES.87108, adult, sex unknown, mounted skin. Loc.: Chile. Leg.: -.

Schlegel (1863n: 5) listed RMNH.AVES.87108 as “type de *l’Ibis chalcoptera*, Temminck”, hereby designating it lectotype.

According to the original description paralectotypes from Chile are in the MNHN. However, Voisin (1993) did not list any specimens there which could fit as a type.

This species is depicted on pl. 511, not 515 as erroneously given in Van den Hoek Ostende et al. (1997: 22).

***Ibisperegrina*** Bonaparte, 1857: 159.

= *Plegadisfalcinellus* (Linnaeus, 1766).

Syntype, RMNH.AVES.87109, immature female, mounted skin. Loc.: Makassar, Celebes [= Sulawesi], [Indonesia], iii.1828. Leg.: S. Müller.

Bonaparte (1857) referred to specimens in the RMNH from Java collected by Korthals, from Makassar collected by Müller and from “Menado” [= Manado] collected by Forsten. We assume Bonaparte confused his data as there are specimens from Java collected by Müller and from Lake Gorontalo (Sulawesi) collected by Forsten. No specimens collected by Korthals could be found or are mentioned in the catalogue by Schlegel (1863n: 3–4).

There are also specimens collected by Kuhl and Van Hasselt from Java which must have been present at the time of Bonaparte’s visit. Two early specimens from Java have no collector indicated on the label and two specimens collected by Forsten are labelled ‘Celebes’. These have not been listed here, since they do not fit the provenance or collector indicated by Bonaparte, but all these specimens could be part of the type series.

***Ibispapillosa*** Temminck, 1824: livr. 51, pl. 304.

= *Pseudibispapillosa* (Temminck, 1824).

Paralectotypes, RMNH.AVES.87110 and 87111.

Voisin (1993: 46) selected the lectotype in the MNHN (MNHN C.G.1992-376) and listed a paralectotype (MNHN C.G.1992-375). The MLC holds another paralectotype (MLC.2011.0.503).

According to Schlegel (1863n: 11) RMNH.AVES.87110 is the depicted specimen.

***Ibisplumbeus*** Temminck, 1823: livr. 40, pl. 235.

= *Theristicuscaerulescens* (Vieillot, 1817).

Paralectotypes, RMNH.AVES.90999 and 91000.

According to the original description other type specimens are in the MNHN and NMW. Voisin (1993) listed a specimen in the MNHN (MNHN C.G.1992-374) and declared it erroneously as holotype by monotypy, which must be considered as lectotype selection. All other type specimens therefore become paralectotypes.

Schifter et al. (2007: 51–52) mentioned two types collected by Natterer in Ipanema in the NMW (both erroneously under NMW 47467, but second specimen: NMW 47474, E. Bauernfeind, pers. comm., September 2007). Both specimens were identified as *Mesembrinibiscayennensis* (Gmelin, 1789). However, Temminck described and depicted specimens of *Theristicuscaerulescens*. Consequently, there remains some doubt about the type status of these specimens.

***Theristicuscolumbianus*** Finsch, 1899b: 23.

= *Theristicuscaudatuscaudatus* (Boddaert, 1783).

Holotype, RMNH.AVES.87112, immature, sex unknown, mounted skin. Loc.: Colombia. Ex: Deyrolle, 1867.

Holotype by monotypy. Finsch (1899b) mentioned a single specimen from Colombia, received from Deyrolle, Paris, in 1867 and described it as an adult bird. Based on its label however, it is immature.

***Ibisleucon*** Temminck, 1829: livr. 81, pl. 481.

= *Threskiornismelanocephalus* (Latham, 1790).

Paralectotypes, RMNH.AVES.87113, 91001–91004.

According to Dickinson (2001: 46), livraison 81 was published in 1829 and not in 1824 as given by Voisin (1993).

Temminck (1829) mentioned a series of specimens in the RMNH, including all ages and both sexes. Voisin (1993: 49) listed two type specimens in the MNHN and selected one as the lectotype of Temminck´s name and of *Ibisbengala* Cuvier, 1829 (MNHN C.G.1992-373) and the second paralectotype (MNHN C.G.1992-366) of *leucon*, as the lectotype of *Ibismacei* Wagler, 1827. Having the same collecting data, MNHN C.G.1992-367, listed as a paralectotype of *Ibismacei* by Voisin (1993), might even be a third paralectotype of *leucon*.

Schifter et al. (2007: 52–53) listed a possible type specimen in the NMW (NMW 63273).

#### ﻿Ardeidae

***Ardeaflavirostris*** “Temminck” Wagler, 1827: spec. 9, [p. 177].

= *Ardeaalba* subsp.

A specimen from Java in the RMNH (RMNH.AVES.108220) does not refer to Prevost as indicated by Wagler, but to Boie. We therefore do not list this specimen as syntype of *Ardeaflavirostris* Wagler. Schifter et al. (2007: 46) refer to a specimen from South Africa received from Temminck in 1830 (NMW 47.453) which does not refer to Prevost either. According to J. Jansen (pers. comm., 2022) another specimen was sent by Temminck in 1823 to the MfN.

***Ardeacinereaaltirostris*** Mees, 1971b: 225.

= *Ardeacinereajouyi* Clark, 1907.

Holotype, RMNH.AVES.28454, adult male, skin. Loc.: Sedari, Java, [Indonesia], 12.iii.1918. Ex: M.E.G. Bartels, 01.vi.1954.

Paratypes, RMNH.AVES.28443–28453, 28455–28463, 87052–87056, 252791, 252915.

***Ardeagoliat*** Temminck, 1829: livr. 80, pl. 474.

= *Ardeagoliath* Cretzschmar, 1829.

Paralectotype, RMNH.AVES.90748.

According to Dickinson (2001: 46) pl. 474 was published in livraison 80 on 5 September 1829. Cretzschmar (1827–1830: 39, pl. 26) published *ArdeaGoliath* also in 1829 (Steinbacher 1949: 103). Since no exact date is given the publication must be adopted to 31 December 1829 (Art. 21.3.; ICZN 1999). Although Temminck’s *Ardeagoliat* would have priority over Cretzschmar’s *A.goliath*, Steinheimer (2005b: 168–170) gave evidence that *A.goliat* Temminck has not been used as a valid name since 1899, whereas the younger name *A.goliath* Cretzschmar is in prevailing usage. In accordance with Art. 23.9.2. (ICZN 1999) he declared *A.goliath* Cretzschmar as a nomen protectum giving precedence over *A.goliat* Temminck, thus as nomen oblitum making *A.goliat* Temminck invalid but available and linked to its type specimens.

Temminck referred to a specimen depicted by Rüppell in his ‘Atlas zu der Reise im nördlichen Afrika’ (between immature and adult) and to an adult in the RMNH.

Steinbacher (1949: 103) designated the lectotype in the SMF (SMF 12598).

***Ardeapurpureamadagascariensis*** van Oort, 1910c: 83.

= *Ardeapurpureamadagascariensis* van Oort, 1910.

Syntype, RMNH.AVES.87057, adult male, mounted skin. Loc.: N.W. Madagascar. Leg.: D.C. van Dam.

Syntype, RMNH.AVES.87058, adult female, mounted skin. Loc.: Foulpoint, Madagascar, 02.xi.1875. Leg.: J.P. Audebert.

***Ardeatyphon*** Temminck, 1829: livr. 80, pl. 475.

***ArdeaTemminckii*** Reichenbach, 1852: XVI (nomen novum) (see t. 159, ic. 466).

***Ardearobusta*** Bonaparte,1855: 110 (ex MS Muller) (nomen novum).

= *Ardeasumatrana* Raffles, 1822.

Holotype, RMNH.AVES.87059, adult, sex unknown, mounted skin. Loc.: “Inde Continentale” [written under the stand], “Engelsch Indie” [on the label]. Leg.: -.

Holotype by monotypy. Temminck (1829) referred to a single object from Africa (Gambia, Galam) in the RMNH. This was obviously an error since he described and depicted a specimen of *A.sumatrana* Raffles, 1822. The locality “Indonesia” given by Van den Hoek Ostende et al. (1997: 18) was also wrong. The label reads “Engelsch Indie” [British India] while the stand gives “Inde Continentale”: both are erroneous as the species does not occur in India.

***Ardeasemirufa*** Schlegel, 1863g: 35.

= *Ardeolarufiventris* (Sundevall, 1850).

Syntype, RMNH.AVES.87060, adult, sex unknown, mounted skin. Loc.: South Africa. Ex: G.A. Frank, 1862.

Syntype, RMNH.AVES.87061, immature, mounted skin. Loc.: South Africa. Ex: G.A. Frank, 1862.

According to the labels these specimens were received in 1862. Schlegel (1863g), however, gave 1861 as the acquisition date.

***Ardeastellariscapensis*** Schlegel, 1863g: 48.

= *Botaurusstellariscapensis* (Schlegel, 1863).

Syntype, RMNH.AVES.87062, adult female, mounted skin. Loc.: “Cap”, [South Africa]. Leg.: H.B. van Horstok.

Syntype, RMNH.AVES.87063, adult male, mounted skin. Loc.: Latakou [= Dithakong], South Africa, x.1834. Leg.: J.P. Verreaux. Ex: Maison Verreaux, 1858.

***Ardeanigripes*** Temminck, 1840: 376.

= *Egrettagarzettanigripes* (Temminck, 1840).

Syntype, RMNH.AVES.87064, adult male, mounted skin. Loc.: Java, [Indonesia], [ix.1821–ix.1823]. Leg.: J.C. van Hasselt.

Syntype, RMNH.AVES.87065, adult male, mounted skin. Loc.: Surabaja, Java, [Indonesia]. Leg.: S. Müller.

Syntype, RMNH.AVES.87066, adult female, mounted skin. Loc.: Surabaja, Java, [Indonesia], ii.1827. Leg.: S. Müller.

Syntype, RMNH.AVES.87067, immature, mounted skin. Loc.: Java, [Indonesia]. Leg.: -.

Syntype, RMNH.AVES.87068, adult, sex unknown, skeleton. Loc.: Java, [Indonesia], [vi.1826–ix.1827]. Leg.: H. Boie.

Schifter et al. (2007: 47) listed another syntype in the NMW (NMW 47482) received in April 1830. Aimassi et al. (2020: 97) listed a syntype in the MZUT (MZUT Av15625). Another arrived in the MfN in December 1824.

***ArdeaLansbergei*** Schlegel, 1879e: 113.

= *Egrettapicata* (Gould, 1845).

Syntype, RMNH.AVES.87069, immature, mounted skin. Loc.: Makassar, Celebes [= Sulawesi], [Indonesia]. Leg.: J.E. Teysmann [J.E. Teijsmann], 1878.

Syntype, RMNH.AVES.87070, immature, mounted skin. Loc.: Makassar, Celebes [= Sulawesi], [Indonesia]. Leg.: J.E. Teysmann [J.E. Teijsmann], 1878.

Syntype, RMNH.AVES.87071, immature, mounted skin. Loc.: Makassar, Celebes [= Sulawesi], [Indonesia]. Leg.: J.E. Teysmann [J.E. Teijsmann], 1878.

Syntype, RMNH.AVES.87072, immature, mounted skin. Loc.: Makassar, Celebes [= Sulawesi], [Indonesia]. Leg.: J.E. Teysmann [J.E. Teijsmann], 1878.

Syntype, RMNH.AVES.87073, immature, mounted skin. Loc.: Makassar, Celebes [= Sulawesi], [Indonesia]. Leg.: J.E. Teysmann [J.E. Teijsmann], 1878.

The specimens are most likely collected during the Celebes expedition of 1877, during which Teijsmann stayed in the Makassar area in June, July, October, and December. The year 1878 on the label is probably the year they arrived in Leiden.

**Ardea (Herodias) picata** Gould, 1845a: 62.

= *Egrettapicata* (Gould, 1845).

Possible paralectotype, RMNH.AVES.233489.

Fisher and Calaby (2009: 90) listed this specimen as a possible type. The lectotype was selected by Stone (1913: 143) and is in the ANSP (ANSP 6668).

***Nycticoraxgoisagi*** Temminck, 1836: livr. 98, pl. 582.

= *Gorsachiusgoisagi* (Temminck, 1836).

Paralectotypes, RMNH.AVES.87074–87077.

By mistake, Voisin and Voisin (1996: 597) considered the only specimen in the MNHN as holotype (MNHN C.G.1995-241), which is therefore a lectotype selection.

The year of publication of livraison 97, usually given as 1835 following Sherborn (1898: 488), has been corrected to 1836 by Mees (1994: 51) and confirmed by Dickinson (2001: 47). The year of publication of livraison 98 and 99 was subsequently corrected to 1836 by Dickinson (2001: 47), contra Sherborn (1898: 488) who gave 1835 for both livraisons.

According to Schlegel (1863g: 55) the two females were collected by Von Siebold, although on the labels Bürger is given as collector.

A skull (RMNH.AVES.87078) listed as paralectotype by Van den Hoek Ostende et al. (1997: 19) does not belong to the type series since Temminck did not mention any skeletal material. This skull has later been identified as *Nycticoraxnycticorax*.

***Nycticoraxlimnophilax*** Temminck, 1836: livr. 98, pl. 581.

= *Gorsachiusmelanolophus* (Raffles, 1822).

Syntype, RMNH.AVES.87079, immature, mounted skin. Loc.: Java, [Indonesia], [xii.1820–ix.1821]. Leg.: H. Kuhl and J.C. van Hasselt.

Syntype, RMNH.AVES.108662, immature, mounted skin. Loc.: Java, [Indonesia], [xii.1820–ix.1821]. Leg.: H. Kuhl and J.C. van Hasselt.

The year of publication of livraison 97, usually given as 1835 following Sherborn (1898: 488), has been corrected to 1836 by Mees (1994: 51) and confirmed by Dickinson (2001: 47). The year of publication of livraison 98 and 99 was subsequently corrected to 1836 by Dickinson (2001: 47), contra Sherborn (1898: 488) who gave 1835 for both livraisons.

Temminck (1836) gave no indication how many specimens he had and where he had seen them, but he mentioned that both sexes were alike, so he had more than one specimen.

Kuhl and Van Hasselt collected on Java from December 1820 until September 1821.

***Ardeasibilatrix*** Temminck, 1824: livr. 46, pl. 271.

= *Syrigmasibilatrixsibilatrix* (Temminck, 1824).

Syntype, RMNH.AVES.87080, adult, sex unknown, mounted skin. Loc.: Brazil. Leg.: Bonjour.

According to the original description other syntypes are in the MNHN. However, Voisin and Voisin (1996) did not list any specimen in the MNHN which could fit as type. According to Gouraud (2015: 138) the specimen Temminck saw in the MNHN is now in the MLC (MLC.2010.0.77).

#### ﻿Pelecanidae

***Pelecanusconspicillatus*** Temminck, 1824: livr. 47, pl. 276.

= *Pelecanusconspicillatus* Temminck, 1824.

According to Voisin (1992: 170), the holotype is in the MNHN (MNHN C.G.1991-1129).

***Pelecanuscrispus*** Bruch, 1832: 1109.

= *Pelecanuscrispus* Bruch, 1832.

Syntype, RMNH.AVES.87051, adult female, mounted skin. Loc.: Dalmatia, [Croatia], [iii.1831]. Ex: C.F. Bruch.

Bruch (1832) based his description on an adult female in his own collection collected in Dalmatia in March 1831 and on an illustration of an immature from the same date from Cairo by Von Kittlitz (1833).

***Pelecanusminor*** Rüppell, 1837a: 186.

= *Pelecanusonocrotalus* Linnaeus, 1758.

Syntype, RMNH.AVES.87050, adult, sex unknown, mounted skin. Loc.: “Egypte inferior” [Egypt]. Leg.: W.P.E.S. Rüppell.

### ﻿ACCIPITRIFORMES

#### ﻿Cathartidae

***Cathartesurubutinga*** Von Pelzeln, 1861: 7.

= *Cathartesburrovianusurubutinga* Pelzeln, 1861.

Syntype, RMNH.AVES.87114, adult male, mounted skin. Loc.: Forte [de São Joaquim] do Rio Branco, Brazil, 12.ii.1832. Leg.: J. Natterer. Ex: NMW, 1862.

Syntype, RMNH.AVES.87115, adult female, mounted skin. Loc.: Forte [de São Joaquim] do Rio Branco, Brazil, 21.iv.1832. Leg.: J. Natterer. Ex: NMW, 1862.

Both the collecting locality and collecting date of RMNH.AVES.87115 given by Van den Hoek Ostende et al. (1997: 24) are incorrect. According to Schifter et al. (2007: 60–61) the original description was based on nine specimens collected by Natterer in Forte do Rio Branco, Sapitiba, and Irisanga. Five syntypes from these localities are held by the NMW (NMW 39872, 40083–40085, 44259). Another syntype is in the NMNH (USNM 34984; Deignan 1961: 39).

***Cathartesvulturinus*** Temminck, 1821: livr. 6, pl. 31.

= *Gymnogypscalifornianus* (Shaw, 1797).

According to Temminck (1821) the holotype (by monotypy) is in the NHM (NHMUK Old Vellum Cat. 5.2).

#### ﻿Pandionidae

***Pandionhaliaëtusorientalis*** Temminck & Schlegel, 1844: 13.

= *Pandionhaliaetushaliaetus* (Linnaeus, 1758).

Lectotype, RMNH.AVES.90642, adult female, mounted skin. Loc.: Nagasaki, Japan. Leg.: Ph.F.B. von Siebold.

Paralectotypes, RMNH.AVES.90643–90645.

The lectotype was designated by Dekker et al. (2001: 209) fixing *orientalis* to the nominate *haliaetus*. Specimens from Bawean might belong to *P.h.melvillensis* Mathews, 1912. The name *orientalis* is not listed in early synonymies, e.g., Sharpe (1874) and seems to have been overlooked.

#### ﻿Accipitridae

***Falcodussumieri*** Temminck, 1824: livr. 52, pl. 308.

= *Accipiterbadiusdussumieri* (Temminck, 1824).

According to the original description, the type specimens are in the MNHN where Voisin and Voisin (2001a: 184) confirmed three types (MNHN C.G.1999-2133–C.G.1999-2135) and selected MNHN C.G.1999-2133 as lectotype. See also Dickinson (2001: 51).

***Falcopileatus*** Temminck, 1823: livr. 35, pl. 205.

= *Accipiterbicolorpileatus* (Temminck, 1823).

Paralectotypes, RMNH.AVES.87120, 190049–190052.

Temminck (1823) based his description on specimens in the MNHN, RMNH and NMW and in the collection of Wied. According to Schlegel (1873a: 71), RMNH.AVES.87120 was collected by Natterer. However, Schifter et al. (2007: 69–70) contradicted Schlegel by stating that RMNH.AVES.87120 was collected by A.P.M. zu Wied-Neuwied, based on the locality Rio Belmonte, which Natterer never visited. Wied mentioned having a pair of this species in his collection (Zu Wied-Neuwied 1830: 111). One of the alleged Wied specimens (in total three in RMNH and AMNH) must therefore be from another collector or the data got mixed up.

Greenway (1973: 266) designated the lectotype in the AMNH (AMNH 6386). He attributed the lectotype designation to Allen (1889: 267), but Allen did not use the term type for the AMNH specimen. The lectotype designation was overlooked by Van den Hoek Ostende et al. (1997: 24), who listed RMNH.AVES.87120 as a syntype, as well as by Schifter et al. (2007: 69) for NMW 40066, collected by Natterer in Murungaba. Voisin and Voisin (2001a: 187) listed a specimen in the MNHN which might also fit as type (MNHN C.G.1999-2161). It was collected in “l’Amerique méridionale’’ and acquired by the MNHN in 1808. It does, however, not give any reference to Temminck on the label (as do most of Temminck’s type specimens in the MNHN).

***Accipiterchilensis*** Philippi & Landbeck, 1864: 43.

= *Accipiterchilensis* Philippi & Landbeck, 1864.

Syntype, RMNH.AVES.87116, adult male, mounted skin. Loc.: Santiago, Chile. Ex: 1862.

Syntype, RMNH.AVES.87117, immature male, mounted skin. Loc.: Santiago, Chile, vi.1863. Ex: Museum Santiago, 1864.

Syntype, RMNH.AVES.87118, immature female, mounted skin. Loc.: Santiago, Chile, vii.1863. Ex: Museum Santiago, 1864.

Syntype, RMNH.AVES.87119, immature female, mounted skin. Loc.: Santiago, Chile, vi.1863. Leg.: Philippi, 1864.

***Nisuscirrhocephalusceramensis*** Schlegel, 1862c: 39.

= *Accipitererythrauchenceramensis* (Schlegel, 1862).

Holotype, RMNH.AVES.87121, adult female, mounted skin. Loc.: Ceram, [Indonesia]. Leg.: E.A. Forsten.

Holotype by monotypy. Schlegel stated that Forsten only collected a single female. Forsten visited Seram late 1842.

***Nisuserythropus*** Hartlaub, 1855: 354.

= *Accipitererythropuserythropus* (Hartlaub, 1855).

Holotype, RMNH.AVES.87122, adult male, skin. Loc.: Rio Boutry, Côte d’Or [= Ghana]. Leg.: H.S. Pel, 1842.

Holotype by monotypy. Hartlaub did not mention the number of specimens he had seen, but only described a male and gave a single set of measurements.

***Accipiterbüttikoferi*** Sharpe, 1888: 200.

= *Accipitererythropuserythropus* (Hartlaub, 1855).

Syntype, RMNH.AVES.87140, adult male, skin. Loc.: Schiffelinsville, Liberia, 10.i.1887. Leg.: J. Büttikofer and F.X. Stämpfli.

Syntype, RMNH.AVES.87141, adult female, skin. Loc.: Sofore, Liberia, 12.iv.1880. Leg.: J. Büttikofer and C.F. Sala.

Syntype, RMNH.AVES.91010, adult female, skin. Loc.: Du Queah River, Liberia, 19.xii.1885. Leg.: F.X. Stampfli.

Erroneously identified as *Accipiterminullus* (Daudin, 1800) by Van den Hoek Ostende et al. (1997: 27), but later corrected by B. Clark (pers. comm., 11 August 2004). Syntype RMNH.AVES.91010 was not listed in Van den Hoek Ostende et al. (1997: 27).

***Nisusbrutus*** Schlegel, 1865c: 80.

= *Accipiterfrancesiaebrutus* (Schlegel, 1865).

Syntype, RMNH.AVES.87123, adult male, mounted skin. Loc.: Mayotte, Comoro Archipelago, 09.v.1864. Leg.: F.P.L. Pollen and D.C. van Dam.

Syntype, RMNH.AVES.87124, adult female, mounted skin. Loc.: Mayotte, Comoro Archipelago, 09.v.1864. Leg.: F.P.L. Pollen and D.C. van Dam.

Syntype, RMNH.AVES.87125, adult male, mounted skin. Loc.: Mayotte, Comoro Archipelago, 04.v.1864. Leg.: F.P.L. Pollen and D.C. van Dam.

Syntype, RMNH.AVES.87126, adult female, mounted skin. Loc.: Mayotte, Comoro Archipelago, 04.vi.1864. Leg.: F.P.L. Pollen and D.C. van Dam.

Pages 1–180 of vol. 3 of the ‘Nederlandsch Tijdschrift voor de Dierkunde’ were published in 1865 (Pieters and Dickinson 2005: 107).

In his description Schlegel mentioned two males and three females. One female could not be located.

***NisuoïdesMorelii*** Pollen, 1866: 62.

= *Accipiterfrancesiaefrancesiae* Smith, 1834.

Syntype, RMNH.AVES.87127, adult male, mounted skin. Loc.: Parages de Tintingue, Madagascar. Leg.: Lantz, 1866. Ex: Musée de St. Denis, 1868.

This specimen is one of a series of eight syntypes. The others are in the natural history museum of St. Denis (Réunion).

***Falcoregalis*** Temminck, 1830: livr. 84, pl. 495.

= *Accipitergentilisatricapillus* (Wilson, 1812).

Van den Hoek Ostende et al. (1997: 25) listed three syntypes (RMNH.AVES.87128–87130). However, according to the original description the holotype (by monotypy) is in the MNHN. Voisin and Voisin (2001a: 186) did not list the holotype, but discussed the holotype of *Doedalionpictum* Lesson, 1830, which they identified as *A.g.atricapillus* under current taxonomy. This specimen, MNHN C.G.1999-2156, collected by Lesueur in North America and received by the MNHN in 1825, could have been available to Temminck and might be the holotype of *Falcoregalis*.

***Asturgriseiceps*** Kaup, 1848: 774.

= *Accipitergriseiceps* (Kaup, 1848).

Syntype, RMNH.AVES.88990, adult male, mounted skin. Loc.: near beach at Atep, Celebes [= Sulawesi], [Indonesia]. Leg.: E.A. Forsten.

Syntype, RMNH.AVES.88991, adult female, mounted skin. Loc.: Gorontalo, Celebes [= Sulawesi], [Indonesia]. Leg.: E.A. Forsten.

Syntype, RMNH.AVES.88992, immature female, mounted skin. Loc.: Gorontalo, Celebes [= Sulawesi], [Indonesia]. Leg.: E.A. Forsten.

This name is usually, but incorrectly, attributed to Schlegel (1862c). Forsten stayed in Celebes twice between March 1840 and April 1842.

**Astur (Nisus) gularis** Temminck & Schlegel, 1844: 5, pl. 2.

= *Accipitergularisgularis* (Temminck & Schlegel, 1844).

Syntype, RMNH.AVES.87131, adult male, mounted skin. Loc.: Japan. Leg.: -.

Syntype, RMNH.AVES.87132, adult female, mounted skin. Loc.: Japan. Leg.: -.

***Asturhenstii*** Schlegel, 1873a: 62.

= *Accipiterhenstii* (Schlegel, 1873).

Syntype, RMNH.AVES.87133, adult female, mounted skin. Loc.: Mouroundava [= Morondova], Madagascar, 1870. Leg.: D.C. van Dam.

Syntype, RMNH.AVES.87134, adult male, mounted skin. Loc.: Mouroundava [= Morondova], Madagascar, 1870. Leg.: D.C. van Dam.

***Falcohiogaster*** Müller, 1841: 110.

= *Accipiterhiogasterhiogaster* (Müller, 1841).

Syntype, RMNH.AVES.87142, adult male, mounted skin. Loc.: Ambon, [Indonesia], [iv.1828]. Leg.: S. Müller.

Syntype, RMNH.AVES.87143, adult female, mounted skin. Loc.: Ambon, [Indonesia], [iv.1828]. Leg.: S. Müller.

Müller collected on Ambon in April 1828.

***Asturnovaehollandiaeleucosomus*** Sharpe, 1874: 94.

= *Accipiterhiogasterleucosomus* (Sharpe, 1874).

Holotype, RMNH.AVES.87144, adult male, skin. Loc.: Aidoema, Lobo Bay, [Papua], [Indonesia], [vi–viii] 1828. Leg.: S. Müller.

Holotype by monotypy. Sharpe (1874) gave measurements of a single male in the RMNH.

***Urospiziaspallidiceps*** Salvadori, 1879: 474.

= *Accipiterhiogasterpallidiceps* (Salvadori, 1879).

Syntype, RMNH.AVES.87145, adult male, mounted skin. Loc.: Buru, [Indonesia], 06.xi.1873. Leg.: S.C.J.W. van Musschenbroek, 1878.

***Asturhypoxanthus*** Schlegel, 1862c: 16.

= *Accipitermelanoleucusmelanoleucus* Smith, 1830.

Syntype, RMNH.AVES.87135, adult male, mounted skin. Loc.: Zondagsrivier, [South Africa]. Ex: MfN (ZMB 772), 1863.

In discussing *Asturmelanoleucus* Smith, 1830, Schlegel (1862c) split the taxon into two species. He described the adult specimens of the dark morph under *Asturapoxypterus* (holotype in the MfN) and the rufous coloured immature birds as *Asturhypoxanthus*. For *A.hypoxanthus* he listed specimens in the collections of the MfN and MNSL. According to Mauersberger and Neumann (1986: 140) there is no specimen in the MNSL which could fit as a type. The collection catalogue of the MfN gives three specimens (ZMB 770–772) collected by G.L.E. Krebs in the Sundays River area, South Africa, in 1822. One of them later came to the RMNH, a second is still available (ZMB 771; see www.gbif-vertebrata.de), but the third syntype cannot be traced. For more background information on the types see Mauersberger and Neumann (1986: 140).

***AsturTemminckii*** “Pel” Hartlaub, 1855: 353.

= *Accipitermelanoleucustemminckii* (Hartlaub, 1855).

Syntype, RMNH.AVES.87136, adult male, mounted skin. Loc.: Rio Boutry, Côte d’Or [= Ghana]. Leg.: H.S. Pel.

Hartlaub (1855) mentioned a male and female. The whereabouts of the female are unknown.

***Nisusverreauxii*** Schlegel, 1862c: 37.

= *Accipitermelanoleucus* subsp.

Syntype, RMNH.AVES.87136, adult male, mounted skin. Loc.: Rio Boutry, Côte d’Or [= Ghana]. Leg.: H.S. Pel (= *Accipitermelanoleucustemminckii* (Hartlaub, 1855)).

Syntype, RMNH.AVES.87139, adult female, mounted skin. Loc.: Kaiskama, [South Africa]. Leg.: J. Verreaux (= *Accipitermelanoleucusmelanoleucus* Smith, 1830).

The two syntypes of *Nisusverreauxii* belong to different subspecies of *Accipitermelanoleucus*. RMNH.AVES.87136 is also syntype of *Asturtemminckii* Hartlaub, 1855 (see above).

Based on the different coloration of morphs, adults, and immature birds, Schlegel (1862c) split *A.melanoleucus* into three species: *Asturapoxypterus* for the dark morph of the nominate race, *A.hypoxanthus* for the rufous coloured immature (see also *A.hypoxanthus*), and *Nisusverreauxii* for adult birds. As long as *A.melanoleucus* was considered to be monotypic all three names were listed in the synonymy of this name (e.g., Sharpe 1874: 156; Reichenow 1901: 551). Only when the smaller west-African race *temminckii* was separated from the larger nominate race from eastern and southern Africa, the type series became composite.

Since Schlegel’s description includes both taxa, the name *verreauxii* cannot unambiguously be applied to one of them. A fixation of *verreauxii* either to *temminckii* or *melanoleucus* by lectotypification would be an arbitrary selection. Furthermore, there is no taxonomic purpose of a lectotype selection since none of the current names is antedated by Schlegel’s *verreauxii*. Therefore no lectotype needs to be designated here.

***Falcopoliogaster*** Temminck, 1824: livr. 45, pl. 264.

= *Accipiterpoliogaster* (Temminck, 1824).

Lectotype, RMNH.AVES.87146, adult male, mounted skin. Loc.: [Ipanema], [São Paulo], Brazil, [vi.1819]. Leg.: J. Natterer (395).

According to Dickinson (2001: 50) the text was included in livraison 45. It mentioned two plates: 264 (adult male) and 295 (immature), although the latter only appeared in livraison 50, six months after livraison 45 was published. As given by the original description, another paralectotype is in the NMW (NMW 48082; Schifter et al. 2007: 66).

According to Von Pelzeln (1870: 8) Natterer collected these specimens in March and June 1819 in Ipanema. As the specimen in the NMW is from March, the RMNH specimen was probably collected in June 1819.

Hellmayr and Conover (1949: 68), by referring to the Leiden specimen as “the type”, designated it the lectotype.

***Accipiterrhodogasterbutonensis*** Voous, 1951: 82 (in Van Bemmel and Voous 1951).

= *Accipiterrhodogasterrhodogaster* (Schlegel, 1862).

Holotype, ZMA.AVES.8702, adult female, skin. Loc.: Buton, Celebes [Sulawesi], [Indonesia], viii.1909. Leg.: M. Mohari. Ex: J. Elbert [Sunda Expedition 1909–1910; #175].

Paratypes, ZMA.AVES.47902 and 47903.

According to Van Bemmel and Voous (1951) there are two paratypes in the Elbert collection in the SMF and probably three in the MZB.

***Nisusvirgatusrhodogaster*** Schlegel, 1862c: 32.

= *Accipiterrhodogasterrhodogaster* (Schlegel, 1862).

Syntype, RMNH.AVES.87147, adult male, mounted skin. Loc.: Gorontalo, Celebes [= Sulawesi], [Indonesia]. Leg.: E.A. Forsten.

Syntype, RMNH.AVES.87148, adult female, mounted skin. Loc.: Gorontalo, Celebes [= Sulawesi], [Indonesia]. Leg.: E.A. Forsten.

Forsten visited the area near Gorontalo from September 1841 until April 1842.

***Nisussulaënsis*** Schlegel, 1866a: 26.

= *Accipiterrhodogastersulaensis* (Schlegel, 1866).

Holotype, RMNH.AVES.87149, adult female, mounted skin. Loc.: Sula-Besi, [Indonesia], i.1864. Leg.: H.A. Bernstein.

Holotype by monotypy, as Schlegel (1866a) wrote that Bernstein sent a single adult specimen.

***Falcoexilis*** Temminck, 1830: livr. 84, pl. 496.

= *Accipiterrufiventrisrufiventris* Smith, 1830.

Syntype, RMNH.AVES.87150, adult male, mounted skin. Loc.: “Afrique Australe” [= South Africa]. Leg.: -.

Syntype, RMNH.AVES.87151, adult male, mounted skin. Loc.: “Afrique Australe” [= South Africa]. Leg.: -.

Syntype, RMNH.AVES.87152, adult female, mounted skin. Loc.: “Afrique Australe” [= South Africa]. Leg.: -.

According to Schlegel (1862c: 30) RMNH.AVES.87150 is the depicted specimen of pl. 496.

In reaction to Mees (1967: 144), who believed that the birds until then identified as *rufiventris* Smith, 1830 should be called *exilis* Temminck, 1830, the ICZN ruled that the name *exilis* Temminck, 1830 was suppressed in favour of *rufiventris* Smith, 1830 (ICZN 1974: 186), even though Smith had no intention of describing a new species and the priority was questionable.

***Falcocuculoides*** Temminck, 1822: livr. 19, pl. 110.

= *Accipitersoloensis* (Horsfield, 1821).

According to Dickinson (2001: 48), pl. 110 (immature female) appeared in February 1822, followed three months later by livr. 22 containing the text and pl. 129 (adult male). Based on the text, this name was published as a nomen novum for *Falco soloënsis* (Horsfield, 1821). However, as the plate was published prior to the text, we treat this name as validly published and the depicted specimen as the holotype (see Dickinson et al. 2022). According to Temminck the holotype is an immature female, collected by Reinwardt. As the two types listed by Van den Hoek Ostende et al. (1997: 28; RMNH.AVES.87153 and 87154) are both male, they are excluded from the type series. According to Schifter et al. (2007: 67) at least two specimens went in exchange to the NMW (NMW 71223 and 71225) (one in February 1822 and one in September 1833), although they have no reference to Reinwardt. One adult was sent to the MfN in December 1823. None of these qualify as type.

Reinwardt collected in Indonesia from April 1816 until June 1822. Temminck mentioned several specimens of all plumages in the RMNH which were collected by Reinwardt.

***Asturmacrocelides*** Hartlaub, 1855: 354.

= *Accipitertousseneliimacroscelides* (Hartlaub, 1855).

Syntype, RMNH.AVES.87155, adult male, mounted skin. Loc.: Rio Boutry, Côte d’Or [= Ghana]. Leg.: H.S. Pel.

Syntype, RMNH.AVES.87156, adult female, mounted skin. Loc.: Rio Boutry, Côte d’Or [= Ghana], ii.1843. Leg.: H.S. Pel.

Syntype, RMNH.AVES.87157, adult female, mounted skin. Loc.: Saccondee, Côte d’Or [= Sekondi-Takoradi, Ghana]. Leg.: H.S. Pel.

Syntype, RMNH.AVES.87158, adult male, mounted skin. Loc.: Rio Boutry, Côte d’Or [= Ghana]. Leg.: H.S. Pel.

Syntype, RMNH.AVES.87159, adult female, mounted skin. Loc.: Saccondee, Côte d’Or [= Sekondi-Takoradi, Ghana], iv.1840. Leg.: H.S. Pel.

***Accipitertrinotatus*** Bonaparte, 1850a: 33.

= *Accipitertrinotatus* Bonaparte, 1850.

Syntype, RMNH.AVES.87160, adult female, mounted skin. Loc.: Celebes [= Sulawesi], [Indonesia]. Leg.: E.A. Forsten.

Syntype, RMNH.AVES.87161, immature male, mounted skin. Loc.: Gorontalo, Celebes [= Sulawesi], [Indonesia]. Leg.: E.A. Forsten.

Forsten stayed in Celebes twice between March 1840 and April 1842 and in the area near Gorontalo between September 1841 and April 1842.

***Falcotrivirgatus*** Temminck, 1824: livr. 51, pl. 303.

= *Accipitertrivirgatustrivirgatus* (Temminck, 1824).

According to the original description, the type specimens are in the MNHN, where Voisin and Voisin (2001a: 185) listed a specimen as holotype (MNHN C.G.1999-2167).

***Accipitervirgatusfuscipectus*** Mees, 1970: 286.

= *Accipitervirgatusfuscipectus* Mees, 1970.

Holotype, RMNH.AVES.59023, adult male, skin. Loc.: Wanta River, Taiwan, 04.iii.1969. Leg.: K.H. Chen, 8.v.1969.

Paratypes, RMNH.AVES.49280–49282, 52698, 52755 and 52756, 52885, 53049, 53197, 53405, 54089, 58873, 59024 and 59025.

Two paratypes are in the AMNH and three in the NHM.

***Accipitervirgatusquinquefasciatus*** Mees, 1984: 314.

= *Accipitervirgatusquinquefasciatus* Mees, 1984.

Holotype, RMNH.AVES.81024, adult male, skin. Loc.: Ruteng, Flores, Indonesia, 26.iv.1978. Leg.: E. Schmutz.

A paratype is in the AMNH.

***Accipitervirgatusvanbemmeli*** Voous, 1950: 99.

= *Accipitervirgatusvanbemmeli* Voous, 1950.

Holotype, ZMA.AVES.2589, adult male, skin. Loc.: Berastagi, [N-Sumatra], [Indonesia], 28.iii.1914. Ex: L.P. le Cosquino de Bussy (110a).

Paratypes, ZMA.AVES.2590, RMNH.AVES.190673, 190676–190678, 194204.

Voous (1950) examined 12 birds from the RMNH, MZB, AMNH, and MCZ and private collections (now mostly in RMNH) for his description. The RMNH specimens listed above could be identified as part of the type series based on the localities mentioned by Voous.

***Falcovirgatus*** Temminck, 1822: livr. 19, pl. 109.

= *Accipitervirgatusvirgatus* (Temminck, 1822).

Holotype, RMNH.AVES.87162, adult male, mounted skin. Loc.: Java, [Indonesia]. Leg.: C.G.C. Reinwardt.

Livraison 19 was published in February 1822, the text to the plate appeared on 26 June 1824.

Holotype by monotypy. Reinwardt visited Java several times between April 1816 and March 1822.

***Falcofucosa*** ‘Cuvier’ Temminck, 1821: livr. 6, pl. 32.

= *Aquilaaudaxaudax* (Latham, 1801).

Following Dickinson et al. (2022) (see also Introduction of this catalogue under “What is new in this 2023 version”: 14) the specimen illustrated on pl. 32 is the holotype since the text that appeared later is irrelevant regarding type status. In the text, however, Temminck (1821) referred to one specimen in the RMNH and two in the MNHN, none have been reported in Van den Hoek Ostende et al. (1997) or any of the MNHN type catalogues.

**Falco (Aquila) albicans** Rüppell, 1835: 34.

= *Aquilarapaxbelisarius* (Levaillant, 1850).

Paralectotypes, RMNH.AVES.87166–87167.

The lectotype was designated by Steinbacher (1949: 104) and is in the SMF (SMF 12609). Paralectotypes are in the SMF and NHM (Steinheimer 2005a: 237).

According to correspondence in the RMNH archives (‘Angebotene Vögel aus Abyssinien, abgebildet in meiner Abyssinia Fauna’ [‘Birds offered from Abyssinia, depicted in my Abyssinia Fauna’]), the RMNH specimens were part of a collection Rüppell sold to the museum between September and October 1837.

Rüppell’s name is preoccupied (and not a nomen oblitum as stated by Van den Hoek Ostende et al. 1997: 31) by *Falcoalbicans* Gmelin, 1788, now considered a white morph of gyrfalcon. Next available name is *Falco Belisarius* Levaillant, 1850. For more information on different usage of names and nomenclatural problems see Sclater and Mackworth-Praed (1920: 854) and Steinheimer (2005b: 173–174).

***Falcorapax*** Temminck, 1828: livr. 76 (error for livr. 77), pl. 455.

= *Aquilarapaxrapax* (Temminck, 1828).

Syntype, RMNH.AVES.87165, adult female, mounted skin. Loc.: “Cap”, [South Africa]. Leg.: H.B. van Horstok.

Syntype, RMNH.AVES.190771, adult male, mounted skin. Loc.: “Cap”, [South Africa]. Leg.: H.B. van Horstok.

Syntype, RMNH.AVES.190772, juvenile male, mounted skin. Loc.: “Cap”, [South Africa]. Leg.: H.B. van Horstok.

For correction to livraison 77, see Dickinson (2001: 45).

Temminck (1828) mentioned “meridionale de l’Afrique” as type locality and several specimens of both sexes, including a specimen in moult in the MNHN.

***Falcoobsoletus*** Gloger, 1833: 141.

= *Aquilarapaxrapax* (Temminck, 1828).

Syntype, RMNH.AVES.90757, adult female, mounted skin. Loc.: Kafferland [= Cafrérie], South Africa. Leg.: Krebs. Ex: MfN, 1842.

Four syntypes are in the MfN (ZMB 525–527, 529), of which three were collected by Krebs in “Kafferland”. RMNH.AVES.90757 must have been part of Krebs’ series.

***Spizaetusspilogaster*** Bonaparte, 1850c: 487.

= *Aquilaspilogaster* (Bonaparte, 1850).

Syntype, RMNH.AVES.87229, adult male, mounted skin. Loc.: Sennaar, Ethiopia. Leg.: L.W. Ruyssenaars.

According to Bonaparte (1850c)a female syntype is in the RBINS.

***Bazaborneensis*** Brüggemann, 1876: 47.

= *Avicedajerdoniborneensis* (Brüggemann, 1876).

Holotype, RMNH.AVES.87168, adult female, skin. Loc.: Pontianak, Borneo, [Indonesia]. Leg.: P.M. Diard, 1826.

Holotype by monotypy. Brüggemann (1876) renamed “a bird from Borneo’’ mentioned in Schlegel (1873a: 135) as *Bazamagninostris* [sic].

***Bazacelebensis*** Schlegel, 1873a: 135.

= *Avicedajerdonicelebensis* (Schlegel, 1873).

Syntype, RMNH.AVES.87169, adult female, skin. Loc.: Tondano, Celebes [= Sulawesi], [Indonesia]. Leg.: E.A. Forsten, 1840.

Syntype, RMNH.AVES.88917, adult male, mounted skin. Loc.: Menado, N Celebes [= Sulawesi], [Indonesia]. Leg.: L.D.H.A. Renesse van Duyvenbode, 1866.

Syntype, RMNH.AVES.88918, adult male, skin. Loc.: Sula-bessi, [Indonesia], i.1864. Leg.: H.A. Bernstein.

Syntype, RMNH.AVES.88919, adult male, skin. Loc.: Sula-mangoli, [Indonesia], 3.xii.1864. Leg.: H.A. Bernstein.

***Avicedasubcristataobscura*** Junge, 1956a: 231.

= *Avicedasubcristataobscura* Junge, 1956.

Holotype, RMNH.AVES.20985, adult male, skin. Loc.: Biak, Indonesia, 22.viii.1953. Leg.: C. Hoogerheide, 29.ix.1953.

Paratypes, RMNH.AVES.20986, 21433 and 21434, 22118, 87170.

***Falco (Lophotes) Reinwardtii*** Schlegel & Müller, 1841: 37, pl. 5, figs 1 and 2.

= *Avicedasubcristatareinwardtii* (Schlegel & Müller, 1841).

Lectotype, RMNH.AVES.87171, adult male, relaxed mount. Loc.: Amboine [= Ambon], [Indonesia], 1821. Leg.: C.G.C. Reinwardt.

Paralectotypes, RMNH.AVES.88925, RMNH.AVES.87168–87169 (= *Avicedajerdonicelebensis* (Schlegel, 1873), RMNH.AVES.87172 (= *Avicedasubcristatarufa* (Schlegel, 1866).

Different taxa are represented in the type series. RMNH.AVES.87168 is also the holotype of *Bazaborneensis* Brüggemann, 1876. RMNH.AVES.87169 and 87172 are syntypes of *Bazacelebensis* Schlegel, 1873 and *Bazarufa* Schlegel, 1866 respectively. Quaisser and Dekker (2008: 409) designated the lectotype.

***Bazarufa*** Schlegel, 1866a: 40, 41, pl. 27, fig. 4; pl. 28, figs 1–3.

= *Avicedasubcristatarufa* (Schlegel, 1866).

Syntype, RMNH.AVES.87172, adult male, relaxed mount. Loc.: Ternate, [Indonesia], 1821. Leg.: C.G.C. Reinwardt.

Syntype, RMNH.AVES.87173, adult female, relaxed mount. Loc.: Batjan, [Indonesia], ii.1861. Leg.: H.A. Bernstein.

Syntype, RMNH.AVES.87174, adult male, relaxed mount. Loc.: Batjan, [Indonesia], 18.ix.1862. Leg.: H.A. Bernstein.

Syntype, RMNH.AVES.87175, immature female, relaxed mount. Loc.: Batjan, [Indonesia], 11.ix.1862. Leg.: H.A. Bernstein.

Syntype, RMNH.AVES.87176, adult male, relaxed mount. Loc.: Galela, Halmahera, [Indonesia], 14.viii.1861. Leg.: H.A. Bernstein.

Syntype, RMNH.AVES.87177, adult female (male according to Schlegel, an error), relaxed mount. Loc.: Ternate, [Indonesia], 16.v.1861. Leg.: H.A. Bernstein.

Syntype, RMNH.AVES.87178, adult female, relaxed mount. Loc.: Ternate, [Indonesia], 04.viii.1863. Leg.: H.A. Bernstein.

Syntype, RMNH.AVES.87179, immature male, relaxed mount. Loc.: Ternate, [Indonesia], 07.viii.1863. Leg.: H.A. Bernstein.

Syntype, RMNH.AVES.87180, adult female, mounted skin. Loc.: Tidore, [Indonesia], 22.xi.1863. Leg.: H.A. Bernstein.

Syntype, RMNH.AVES.87181, adult female, relaxed mount. Loc.: Morotai, [Indonesia], 19.ix.1861. Leg.: H.A. Bernstein.

Syntype, RMNH.AVES.87182, adult female, relaxed mount. Loc.: Morotai, [Indonesia], 29.viii.1861. Leg.: H.A. Bernstein.

According to the labels RMNH.AVES.87173 was depicted on pl. 28, fig. 1, RMNH.AVES.87174 on pl. 28, fig. 2, RMNH.AVES.87179 on pl. 28, fig. 3 and RMNH.AVES.87175 on pl. 27 fig. 4.

***Bazastresemanni*** Siebers, 1930: 243.

= *Avicedasubcristatastresemanni* (Siebers, 1930).

Holotype, RMNH.AVES.14019, adult male, skin. Loc.: Nal’Besi, Buru [Indonesia], 17.vi.1921. Leg.: L.J. Toxopeus (346). Ex: MZB (MZB 5534).

Siebers (1930) described this taxon as “*Bazastresemanni* subsp. nov.”. He considered this form a subspecies of *Baza* (= *Aviceda*) *subcristata*.

Siebers referred to 15 specimens collected by Toxopeus (Buru Expedition, 11 specimens) and Denin (local collector, 4 specimens). He referred to RMNH.AVES.14019 as “*Typus*”, thereby nominating it the holotype.

***Falcopoliogenys*** Temminck, 1825: livr. 55, pl. 325.

= *Butasturindicus* (Gmelin, 1788).

Syntype, RMNH.AVES.87183, adult male, mounted skin. Loc.: Philippines. Leg.: J.-J. Dussumier.

The syntype which is illustrated on pl. 325 is in the MNHN.

***Falcoliventer*** Temminck, 1827: livr. 74, pl. 438.

= *Butasturliventer* (Temminck, 1827).

Syntype, RMNH.AVES.87184, adult female, mounted skin. Loc.: Java, [Indonesia]. Leg.: C.G.C. Reinwardt.

Temminck (1827) illustrated a male and mentioned other specimens of both sexes from Celebes [= Sulawesi], Sumatra, Java, and the Malayan Peninsula. According to the original description, other syntype(s) are in the MNHN. Schifter et al. (2007: 71) listed two possible syntypes from Celebes received from the RMNH in December 1830 and September 1833 (NMW 44303 and 49653).

Reinwardt visited Java several times between April 1816 and March 1822.

***Falco (Buteo) Augur*** Rüppell, 1836: 38, pl. 16.

= *Buteoaugur* (Rüppell, 1836).

Paralectotype, RMNH.AVES.90935.

The lectotype was designated by Steinbacher (1949: 104) and is in the SMF (SMF 12610). Paralectotypes are in the SMF and NHM (Steinheimer 2005a: 237). According to correspondence in the RMNH archives (‘Angebotene Vögel aus Abyssinien, abgebildet in meiner Abyssinia-Fauna’ [‘Birds offered from Abyssinia, depicted in my Abyssinia-Fauna’]), the RMNH specimen was part of a collection Rüppell sold to the museum between September and October 1837.

**Falco (Buteo) hydrophilus** Rüppell, 1836: 39, pl. 17.

= *Buteoaugur* (Rüppell, 1836).

Syntype, RMNH.AVES.90936, adult male, mounted skin. Loc.: Abyssinia. Leg.: W.P.E.S. Rüppell.

Besides the RMNH specimen, Steinheimer (2005a: 237) listed syntypes in the SMF and NHM (NHMUK 1837.6.10.684). According to correspondence in the RMNH archives (‘Angebotene Vögel aus Abyssinien, abgebildet in meiner Abyssinia-Fauna’) the RMNH specimen was part of a collection Rüppell sold to the museum between September and October 1837.

***Faclo* [sic] *buteocapensis*** Temminck & Schlegel, 1844: 16.

= *Buteobuteovulpinus* (Gloger, 1833).

Lectotype, RMNH.AVES.87194, adult male, mounted skin. Loc.: “Afrique australe” [= South Africa]. Leg.: J. Verreaux.

Paralectotype, RMNH.AVES.87195 (= *Buteotrizonatus* Rudebeck, 1957).

Quaisser and Dekker (2008: 411) designated the lectotype.

***Buteohemilasius*** Temminck & Schlegel, 1844: 18.

= *Buteohemilasius* Temminck & Schlegel, 1844.

Holotype, RMNH.AVES.87196, adult, sex unknown, mounted skin. Loc.: Japan. Leg.: Ph.F.B. von Siebold.

Holotype by monotypy. Temminck and Schlegel (1844: 19) wrote that they had a single specimen. Plate 7 illustrating the holotype was published after the text (Mlíkovský 2012b: 110).

***Faclo* [sic] *buteojaponicus*** Temminck & Schlegel, 1844: 16, pls 6 and 6B.

= *Buteojaponicusjaponicus* Temminck & Schlegel, 1844.

Syntype, RMNH.AVES.87185, adult female, mounted skin. Loc.: Japan. Leg.: Ph.F.B. von Siebold.

Syntype, RMNH.AVES.87186, adult male, mounted skin. Loc.: Japan. Leg.: Ph.F.B. von Siebold.

Syntype, RMNH.AVES.87187, adult male, mounted skin. Loc.: Japan. Leg.: Ph.F.B. von Siebold.

Syntype, RMNH.AVES.87188, adult male, mounted skin. Loc.: Japan. Leg.: Ph.F.B. von Siebold.

Syntype, RMNH.AVES.87189, adult female, mounted skin. Loc.: Japan. Leg.: Ph.F.B. von Siebold.

Syntype, RMNH.AVES.87190, adult female, mounted skin. Loc.: Japan. Leg.: Ph.F.B. von Siebold.

Syntype, RMNH.AVES.87191, adult male, mounted skin. Loc.: Japan. Leg.: H. Bürger.

Syntype, RMNH.AVES.87192, adult, sex unknown, mounted skin. Loc.: Japan. Leg.: H. Bürger.

Syntype, RMNH.AVES.87193, adult female, mounted skin. Loc.: Japan. Leg.: H. Bürger.

Syntype, RMNH.AVES.90746, adult female, mounted skin. Loc.: Japan. Leg.: H. Bürger.

RMNH.AVES.87189 and RMNH.AVES.87193 are the specimens illustrated in the Fauna Japonica. The description on p. 16 and pl. 6 were both part of fascicle 1 which was published in 1844. The date of publication of pl. 6B is unknown (Holthuis and Sakai 1970). See also Dekker et al. (2001).

***Falcostriolatus*** Temminck, 1821: livr. 15, pl. 87.

= *Buteonitidus* (Latham, 1790).

Following Dickinson et al. (2022) (see also Introduction of this catalogue under “What is new in this 2023 version”: 14) the specimen illustrated on pl. 87 is the holotype since the text that appeared later is irrelevant regarding type status. According to Temminck (1821), the illustrated specimen was in the MNHN.

***Falcocirtensis*** Levaillant, 1850: pl. 3.

= *Buteorufinuscirtensis* (Levaillant, 1850).

Paralectotype, RMNH.AVES.87199 (= *Buteobuteovulpinus* (Gloger, 1833).

According to Schlegel (1862b: 5–6) RMNH.AVES.87199 is one of the types. However, according to descriptions and measurements given by Ferguson-Lees and Christie (2001: 700) and specimens in the RMNH and as indicated by G.F. Mees on the label, RMNH.AVES.87199 does not belong to *cirtensis*, but is *Buteobuteovulpinus* (Gloger, 1833), a taxon which is easily confused with *Buteorufinuscirtensis* (Ferguson-Lees and Christie 2001: 700).

Voisin and Voisin (2001b: 625) listed another type specimen in the MNHN (MNHN C.G.2000-1627). Since this specimen was identified as *cirtensis*, the type series is composed of two taxa. Consequently, Voisin and Voisin (2001b: 625) designated MNHN C.G.2000-1627 as lectotype of *Falcocirtensis* Levaillant, 1850.

***Falcolacernulatus*** Temminck, 1827: livr. 74, pl. 437.

= *Buteogalluslacernulatus* (Temminck, 1827).

According to Voisin and Voisin (2001b: 621), the holotype (by monotypy) is in the MNHN (MNHN C.G.2000-1621).

***Falcorutilans*** Temminck, 1820: livr. 5, pl. 25.

= *Buteogallusmeridionalis* (Latham, 1790).

Following Dickinson et al. (2022) (see also Introduction of this catalogue under “What is new in this 2023 version”: 14) the specimen illustrated on pl. 25 is the holotype since the text that appeared later is irrelevant regarding type status. According to Dickinson (2001: 46) pl. 25 appeared in December 1820, whereas the text was published on 25 June 1823. According to Temminck (1823), the illustrated specimen was in the MNHN, however Voisin and Voisin (2001b) do not mention the name *rutilans*. C. Gouraud and J. Jansen (in litt., 2023) indicated MLC.2011.0.400 (collected by Auguste Saint-Hilaire in Brazil between 1816 and 1822) could be the holotype as shipments from Brazil reached Paris as early as 1818.

***Falcoriocourii*** Temminck, 1821: livr. 15, pl. 85.

= *Chelictiniariocourii* (Temminck, 1821).

Following Dickinson et al. (2022) (see also Introduction of this catalogue under “What is new in this 2023 version”: 14) the specimen on pl. 85 is the holotype since the text that appeared in February 1824 is irrelevant regarding type status. Temminck (1821) indicated that the specimens he had seen were in private collections in France and the holotype might therefore now be in the MNHN.

***Falcouncinatus*** “Illiger” Temminck, 1822: livr. 18, pls 103 (male), 104 (female).

= *Chondrohieraxuncinatusuncinatus* (Temminck, 1822).

Syntype, RMNH.AVES.87200, adult male, mounted skin. Loc.: Brazil. Leg.: -.

Syntype, RMNH.AVES.87201, adult male, mounted skin. Loc.: Brazil. Leg.: A.P.M. zu Wied-Neuwied.

Following Dickinson et al. (2022) (see also Introduction of this catalogue under “What is new in this 2023 version”: 14) we have selected two syntypes based on pls 103 and 104, since the text that appeared on 22 May 1824 and pl. 115 were published later and are irrelevant regarding type status. According to Temminck (1822) the depicted specimens were in the RMNH. RMNH.AVES.87201 matches the bird on pl. 103 and RMNH.AVES.87200 the bird on pl. 104. These findings agree with Schlegel (1862j: 8). Any listing of syntypes for this name in other type catalogue is erroneous. The following specimens in the MfN therefore have no type status: ZMB 720, 721, and 723.

Von Berlepsch (1908: 293) designated a lectotype in the RMNH but failed to specify which specimen. His lectotypification therefore does not conform to the regulations by the ICZN.

This name is thought not to have been published by Illiger (Stresemann and Amadon 1979: 285).

***Falcovitticaudus*** Zu Wied-Neuwied, 1830: 178.

= *Chondrohieraxuncinatusuncinatus* (Temminck, 1822).

Paralectotype, RMNH.AVES.87201.

Van den Hoek Ostende et al. (1997: 34) erroneously listed this specimen as the holotype. However, Wied (1830) based his description on three specimens: a “pair” of immature birds in his own collection and the bird depicted on pl. 103 of the ‘Planches Colorieés’, an adult of the dark morph with a single white band on the tail. One of Wied’s own specimens, an immature female, is in the AMNH (AMNH 6362) and was selected as lectotype by Allen (1889: 269). Since RMNH.AVES.87201, a syntype of *Falcouncinatus* Temminck, 1822, was probably the bird depicted on pl. 103, it also belongs to the type series of *F.vitticaudus* and is now considered a paralectotype.

***Circusgouldi*** Bonaparte, 1850a: 34.

= *Circusapproximans* Peale, 1849.

Syntype, RMNH.AVES.87203, adult male, mounted skin. Loc.: “Patagonia” [= Australia]. Leg.: -.

Syntype, RMNH.AVES.87204, adult female, mounted skin. Loc.: “Patagonia” [= Australia]. Leg.: -.

The two types of *Circusgouldi* are labelled and listed in Schlegel (1862c: 9) as originating from Patagonia. Obviously, this is a mistake since the species is only known from Australia.

***Falcogularis*** Temminck, 1820: livr. 4, pl. 22.

= *Circusbuffoni* (Gmelin, 1788).

Following Dickinson et al. (2022) (see also Introduction of this catalogue under “What is new in this 2023 version”: 14) the specimen illustrated on pl. 22 is the holotype since the text that appeared later is irrelevant regarding type status. According to Temminck (1820), the illustrated specimen was in the MNHN.

***Falcotorquatus*** “Cuvier” Temminck, 1821: livr. 8, pl. 43.

= *Accipitercirrocephalus* (Vieillot, 1817).

Following Dickinson et al. (2022) (see also Introduction of this catalogue under “What is new in this 2023 version”: 14) the specimen illustrated on pl. 43 is the holotype since the text that appeared later is irrelevant regarding type status. According to Temminck (1821), the illustrated specimen was in the MNHN, where MNHN-ZO-MO-1999-2128, listed as syntype for *Falcotorquatus* Cuvier (error for *Falcotorquatus* Temminck) fits pl. 43.

***Falcohistrionicus*** Quoy & Gaimard, 1824: 93, pl. 15 and 16.

= *Circuscinereus* Vieillot, 1816.

Possible syntype, RMNH.AVES.191469, adult male, mounted skin. Loc.: Malouines [= Falkland Islands]. Leg.: J.R.C. Quoy and J.P. Gaimard. Ex: MNHN.

According to Voisin and Voisin (2001a: 179), *Falcohistrionicus* was described on the basis of two males: an adult (pl. 15) and immature (pl. 16), both of them still available in the MNHN (MNHN C.G. 199-2130 and 1999-2131). Although providing correct collecting data, there is no unequivocal evidence whether RMNH.AVES.191469, a third specimen, was also part of the type series. It might have come from the MNHN to the RMNH before it was described by Quoy and Gaimard in 1824.

Voisin and Voisin (2001a: 179) selected the adult male (MNHN C.G.1999-2130) as lectotype. However, neither using the wording as required by the Code (ICZN 1999) nor giving any taxonomic purposes, their statement did not fulfil the requirements of the Code for lectotype designations after 1999 (Art. 74.7.3 ICZN 1999 and Recommendation 74 G of ICZN Declaration 44, 2005) and must be considered invalid. Consequently, all type specimens remain to be syntypes.

After having been shipwrecked in East Falkland, the crew of the L’Uranie remained in the area around Berkeley Sound between 15 February and 27 April 1820.

***Falcomaurus*** Temminck, 1828: livr. 78, pl. 461.

= *Circusmaurus* (Temminck, 1828).

Syntype, RMNH.AVES.87205, adult female, mounted skin. Loc.: Cape, [South Africa]. Leg.: H.B. van Horstok.

Syntype, RMNH.AVES.87206, adult female, mounted skin. Loc.: Cape, [South Africa]. Leg.: H.B. van Horstok.

Syntype, RMNH.AVES.87207, adult male, mounted skin. Loc.: Cape, [South Africa]. Leg.: H.B. van Horstok.

Syntype, RMNH.AVES.87208, adult male, mounted skin. Loc.: Cape, [South Africa]. Leg.: H.B. van Horstok.

Syntype, RMNH.AVES.87209, immature male, mounted skin. Loc.: Cape, [South Africa]. Leg.: H.B. van Horstok.

RMNH.AVES.87205 is the depicted specimen of pl. 461. Schifter et al. (2007: 64) discussed a possible syntype in the NMW received in exchange with the RMNH in 1830 (NMW 3516).

Van Horstok arrived 30 March 1826 in Cape Town, South Africa.

***Asturspectabilis*** Schlegel, 1863e: 131, pl. 6.

= *Circaetusspectabilisspectabilis* (Schlegel, 1863).

Holotype, RMNH.AVES.87210, adult female, skin. Loc.: Elmina, Côte d’Or [= Ghana], spring 1861. Leg.: C.J.M. Nagtglas.

Holotype by monotypy.

***Elanusintermedius*** Schlegel, 1862f: 7.

= *Elanuscaeruleushypoleucus* Gould, 1859.

Syntype, RMNH.AVES.87211, adult, sex unknown, mounted skin. Loc.: Java, [Indonesia]. Leg.: S. Müller.

Syntype, RMNH.AVES.87212, adult, sex unknown, mounted skin. Loc.: Java, [Indonesia]. Leg.: -, 1858.

Syntype, RMNH.AVES.87213, adult male, mounted skin. Loc.: Banjermassing, Borneo, [Indonesia]. Leg.: C.A.L.M. Schwaner.

Syntype, RMNH.AVES.87214, adult, sex unknown, mounted skin. Loc.: Celebes [= Sulawesi], [Indonesia]. Leg.: E.A. Forsten.

Syntype, RMNH.AVES.89419, adult, sex unknown, cranium. Loc.: Celebes [= Sulawesi], [Indonesia]. Leg.: E.A. Forsten.

Forsten stayed in Celebes twice between March 1840 and April 1842.

***Elanusminor*** Bonaparte, 1850a: 22.

= *Elanuscaeruleusvociferus* (Latham, 1790).

Syntype, RMNH.AVES.87215, adult male, mounted skin. Loc.: Nepal. Leg.: B.H. Hodgson.

Syntype, RMNH.AVES.87216, adult male, mounted skin. Loc.: Nepal. Leg.: B.H. Hodgson.

Syntype, RMNH.AVES.87217, adult female, mounted skin. Loc.: Nepal. Leg.: B.H. Hodgson.

***Falcodispar*** Temminck, 1825: livr. 54, pl. 319.

= *Elanusleucurusleucurus* (Vieillot, 1818).

According to Temminck (1825), the type specimens are in the MNHN although they are not listed in Voisin and Voisin (2001a).

***Falcopterocles*** Temminck, 1821: livr. 10, pl. 56.

= *Geranoaetusalbicaudatusalbicaudatus* (Vieillot, 1816).

Following Dickinson et al. (2022) (see also Introduction of this catalogue under “What is new in this 2023 version”: 14) the specimen illustrated on pl. 56 is the holotype since the text that appeared January 1823 is irrelevant regarding type status. According to Temminck (1821) the illustrated specimen was in the MNHN. Hellmayr and Conover (1949: 149) reported about “Temminck’s type” in the MNHN. This specimen, collected by A. Saint-Hilaire in August 1822 (MNHN C.G.2000-1637), is of a too recent date to be the illustrated specimen. There seems to be no other specimen in the MNHN which could fit as a type (Voisin and Voisin 2001b: 631). Any listing of syntypes for this name in any type catalogue is erroneous, although the holotype might be one of them. The following specimens therefore need to be checked against pl. 56: NMW 35243 and 40092 (Schifter et al. 2007: 75–76) and ZMB 667–670 and ZMB 672 in the MfN.

***Falcoaguia*** Temminck, 1824: livr. 51, pl. 302.

= *Geranoaetusmelanoleucusmelanoleucus* (Vieillot, 1819).

Syntype, RMNH.AVES.90741, adult, sex unknown, mounted skin. Loc.: Brazil. Leg.: -.

Temminck (1824) refers in the original description to specimens from Brazil in the collections in the RMNH and MNHN. A single specimen in the RMNH from Brazil without further data as to collector and year and with incorrect reference to pl. 301 in Temminck’s handwriting under the stand, could fit as syntype. The text of livr. 51 also refers incorrectly to pl. 301, as the plate is numbered 302: this fits with the original manuscript by Temminck of the contents of the plates in the archives of the RMNH.

Voisin and Voisin (2001b) did not list a specimen in the MNHN which could fit as a type of *Falcoaguia*.

Incorrectly referred to as *aguja* in Peters’ Checklist vol. I, second edition (1979: 359).

***Falcohemidactylus*** Temminck, 1820: livr. 1, pl. 3.

= *Geranospizacaerulescenscaerulescens* (Vieillot, 1817).

Following Dickinson et al. (2022) (see also Introduction of this catalogue under “What is new in this 2023 version”: 14) the specimen illustrated on pl. 3 is the holotype since the text that appeared later is irrelevant regarding type status. According to Temminck (1820), specimens were in the collections of Laugier de Chartrouse, Prince de Neuwied (= A.P.M. zu Wied-Neuwied) and the MNHN. Voisin and Voisin (2001b: 621) listed (MNHN C.G.2000-1622) as holotype which needs to be checked against pl. 3. Any listing of syntypes for this name in other type catalogues is erroneous.

***Falcogracilis*** Temminck, 1821: livr. 16, pl. 91.

= *Geranospizacaerulescensgracilis* (Temminck, 1821).

Holotype, RMNH.AVES.87218, adult female, mounted skin. Loc.: Brazil. Leg.: -.

Following Dickinson et al. (2022) (see also Introduction of this catalogue under “What is new in this 2023 version”: 14) the illustrated specimen on pl. 91 is the holotype since the text that appeared on 27 March 1824 is irrelevant regarding type status. Temminck (1821) mentioned having seen two specimens, both adult males sexed by Wied and Freyreis (“Treyreis” in Temminck). However, according to the label, RMNH.AVES.87218 is a female. Hellmayer and Conover (1949: 231) referred to the above specimen (“spec. 3” in Schlegel 1862e: 54) as “type”, thus unintentionally and unnecessarily selecting a lectotype.

Freyreis worked near Colonia (Bahia, Brazil). Hellmayer and Conover (1949: 231) restricted the type locality to that area.

***Vulturindus*** Forster, 1798: 40.

***Vulturchaugoun*** Daudin, 1800: 14.

***Vulturindou*** Rüppell, 1830: 382–383.

= *Gypsbengalensis* (Gmelin, 1788).

Holotype, RMNH.AVES.87220, adult, sex unknown, mounted skin. Loc.: “Hindoustan”, [India]. Leg.: -.

Holotype by monotypy. Both authors based their name on Levaillant’s description of “Le Chaugoun” (1799: 50) in which Levaillant wrote about this specimen in singular “d’ou je l’ai reçu”. On the label is added: ‘ind. fig. par Le Vaill. Ois. Afr. Pl. 11’.

***VulturCoprotheres*** Forster, 1798: 35.

***VulturKolbii*** Daudin, 1800: 15.

***Vulturvulgaris*** Vieillot, 1819b: 262.

***Vulturchassefiente*** Ruppel [sic], 1830: 382.

= *Gypscoprotheres* (Forster, 1798).

Syntype, RMNH.AVES.87221, adult, sex unknown, mounted skin. Loc.: South Africa. Leg.: F. Levaillant.

According to Rookmaaker (1989), Levaillant saw “Le Chasse-Fiente” “in the mountains between Cape Town and False Bay, as well as in other places near the ocean”. Levaillant’s description (1799: 45, pl. 10) formed the basis for *Vultur Coprotheres* Forster, 1798, *Vultur Kolbii* Daudin, 1800, *Vulturvulgaris* Vieillot, 1819, and *Vulturchassefiente* Ruppel [sic], 1830. It is a second calendar year bird (P. Mundy, pers. comm., 16 June 1995).

***Vulturfulvusoccidentalis*** Schlegel, 1844: 12.

= *Gypsfulvusfulvus* (Hablizl, 1783).

Syntype, RMNH.AVES.87222, adult female, mounted skin. Loc.: Sardegna, Italy. Leg.: F. Cantraine.

Syntype, RMNH.AVES.87223, adult male, mounted skin. Loc.: Sardegna, Italy. Leg.: F. Cantraine.

In ‘Die Europäischen Tag-Raubvögel’ Schlegel (1844) split the griffon vultures from Europe into two races, an eastern and a western form, but failed to provide them with scientific names. The western form is properly named in Schlegel (1844), the eastern race in Schlegel (1862i).

Cantraine collected in Sardinia between 29 October 1829 and 5 June 1830 (De Koninck 1869).

***Vulturfulvusorientalis*** Schlegel, 1862i: 6.

= *Gypsfulvusfulvus* (Hablizl, 1783).

Syntype, RMNH.AVES.192568, adult sex unknown, mounted skin. Loc.: Eastern Europe. Ex: Cabinet Temminck.

Syntype, RMNH.AVES.192569, adult, sex unknown, mounted skin. Loc.: Spalatro [= Split], Dalmatia, [Croatia], [1831–1833]. Leg.: F. Cantraine.

Syntype, RMNH.AVES.192570, adult, sex unknown, mounted skin. Loc.: Dalmatia, [Croatia], [1831–1833]. Leg.: F. Cantraine.

Syntype, RMNH.AVES.192571, juvenile, sex unknown, mounted skin. Loc.: Dalmatia, [Croatia], [1831–1833]. Leg. F. Cantraine.

Syntype, RMNH.AVES.192572, juvenile, sex unknown, mounted skin. Loc.: Eastern Europe. Leg.: -.

Syntype, RMNH.AVES.192577, adult male. Loc.: Eastern Europe (lived in captivity for 20 years). Leg.: -.

See *Vulturfulvusoccidentalis* Schlegel, 1844. Cantraine collected in Dalmatia between 1831 and 1833.

***Falcohumilis*** Müller & Schlegel, 1841: 44.

= *Haliaeetushumilis* (Müller & Schlegel, 1841).

Syntype, RMNH.AVES.87230, immature, mounted skin. Loc.: Liemonanis [= Limau Manis], Padang-Bessie, Sumatra, [Indonesia]. Leg.: S. Müller, iii.1835.

Syntype, RMNH.AVES.192869, adult female, mounted skin. Loc.: Bengale (= Malaysia). Ex: Frank, 1840.

Müller and Schlegel (1841) mentioned that one young male was collected, but later wrote that they received two additional specimens, presumably from India (Müller and Schlegel 1841: 47). Of these two, only one is still present in the RMNH. The label originally referred to “Bengale”, which was later changed into Malaysia.

***Falcomacei*** Temminck, 1820 (ex Cuvier MS): livr. 2, pl. 8 (ad.).

= *Haliaeetusleucoryphus* (Pallas, 1771).

Following Dickinson et al. (2022) (see also Introduction of this catalogue under “What is new in this 2023 version”: 14) the illustrated specimen on pl. 8 is the holotype since the text that appeared in June 1823 and pl. 223 (immature) which also appeared later are irrelevant regarding type status. Temminck (1820) referred to specimens from “Bengale” in the MNHN. However, Voisin and Voisin (2001a: 186–187) stated that three specimens in the MNHN cannot have type status since they were collected after pl. 8 was published in 1820.

***Falcoleucopterus*** Temminck, 1830: livr. 83, pl. 489.

= *Haliaeetuspelagicus* (Pallas, 1811).

There is no specimen in the RMNH which could fit as a type (Dekker et al. 2001: 204–205), nor did Voisin and Voisin (2001a) list any type specimens in the MNHN.

***Falcodiodon*** Temminck, 1823: livr. 34, pl. 198.

= *Harpagusdiodon* (Temminck, 1823).

Lectotype, RMNH.AVES.87225, adult male, mounted skin. Loc.: Brazil, [1817–iv.1819]. Leg.: J. Natterer [and J. Kammerlacher].

RMNH.AVES.87225 was erroneously listed as holotype by Van den Hoek Ostende et al. (1997: 37). However, in his description Temminck (1823) mentioned specimens (plural) in the collections of Wied, in the NMW, RMNH, and MNHN. By doing so Van den Hoek Ostende et al. (1997: 37) selected a lectotype.

Schifter et al. (2007: 61) mentioned two paralectotypes collected by Natterer in the NMW (NMW 44241 and 40307). A third specimen went to the ZSM. Although not listed by Deignan (1961), one of Wied’s specimens collected in Brazil between 1815 and 1817 is registered in the NMNH (USNM 76818) and might be another paralectotype. Voisin and Voisin (2001a) did not list a type specimen in the MNHN which Temminck referred to in his description.

The type locality was restricted to Ipanema, São Paolo, Brazil by Von Berlepsch (1908: 293).

***Falcohamatus*** Temminck, 1821: livr. 11, pl. 61.

= *Helicolesteshamatus* (Temminck, 1821).

Holotype, RMNH.AVES.87241, adult male, mounted skin. Loc.: Brazil. Leg.: -.

Following Dickinson et al. (2022) (see also Introduction of this catalogue under “What is new in this 2023 version”: 14) we have selected the holotype based on the pl. 61, since the text that appeared later is irrelevant regarding type status. Any listing of syntypes for this name in other type catalogues is erroneous. Five specimens in the MfN therefore have no type status.

***Henicopernislongicaudaminima*** Junge, 1937a: 150.

= *Henicopernislongicauda* (Lesson & Garnot, 1828).

Holotype, RMNH.AVES.87226, adult male, mounted skin. Loc.: Wokam, Aru Islands, [Indonesia], 07.iv.1865. Leg.: C.B.H. von Rosenberg.

Paratypes, RMNH.AVES.87227 and 87228.

***Aquilamorphnoïdes*** Gould 1841: 161.

= *Hieraaetusmorphnoides* (Gould, 1841).

Paralectotype, RMNH.AVES.192843.

Stone (1913: 145) designated the lectotype in the ANSP (ANSP 1733).

***Falcobonelli*** Temminck, 1824: livr. 49, pl. 288.

= *Hieraaetuspennatus* (Gmelin, 1788).

Temminck (1824) did not specify where he had seen the four type specimens. One is in the MRSN (Aimassi et al. 2020: 83; MZUT Av96). There are no specimens in the RMNH which could fit as types, nor did Voisin and Voisin (2001b) list any in the MNHN.

***Falcomalaiensis*** Temminck, 1822: livr. 20, pl. 117.

= *Ictinaetusmalaiensis* (Temminck, 1822).

Following Dickinson et al. (2022) (see also Introduction of this catalogue under “What is new in this 2023 version”: 14) the illustrated specimen on pl. 117 is the holotype since the text that appeared on 26 June 1824 is irrelevant regarding type status. According to Temminck (1822), the illustrated specimen was in the RMNH but is no longer present. Temminck also referred to specimens in the MNHN, but none are listed in Voisin and Voisin (2001a, b).

The spelling of the specific epithet on the wrapper of the plate is *malaiensis*, in the text *malayensis*. The latter spelling has long been followed by different authors. However, the spelling with the plate has priority and should be used.

***Falcomonogrammicus*** Temminck, 1824: livr. 53, pl. 314.

= *Kaupifalcomonogrammicusmonogrammicus* (Temminck, 1824).

Syntype, RMNH.AVES.87231, adult male, mounted skin. Loc.: Senegal. Leg.: -.

Another syntype is in the MNHN (MNHN C.G.2000-1620; Voisin and Voisin 2001b: 621).

***Falcopalliatus*** “Wied” Temminck, 1822: livr. 35, pl. 204.

***Cymindesbuteonidis*** Lesson, 1830 (nomen novum).

= *Leptodoncayanensismonachus* (Vieillot, 1817).

Syntype, RMNH.AVES.192934, [adult], male, mounted skin. Loc.: [Rio Peruhype], [Viçosa], [Bahia], Brazil, [1815–1817]. Leg.: A.P.M. zu Wied-Neuwied.

Syntype, RMNH.AVES.192931, [adult], female, mounted skin. Loc.: Brazil. Leg.: J. Natterer.

There is much confusion regarding the specimens Temminck (1822) used in his description. He described a female but figured a male. Schlegel (1862j: 9) listed a female (cat. 4) as the figured specimen. Hellmayr and Conover (1949: 22) stated that a specimen (RMNH.AVES.192934) in “Leyden’’ is the bird figured and described by Temminck and is the “type” which constitutes a lectotypification. However, in the footnote they refer to a specimen now registered as RMNH.AVES.192931, following Schlegel. Due to this ambiguity, we consider the lectotypification invalid and list both specimens as syntype. Greenway (1973: 258) listed a paralectotype in the AMNH (AMNH 9670), which cannot be correct, because according to Wied (1830: 148) only one specimen was collected.

In the RMNH there are two specimens (RMNH.AVES.192928 and 192929) from Cayenne and one collected by Natterer (RMNH.AVES.192930) which we do not consider to be part of the type series due to lack of information linking it to the original description.

Lesson introduced *Cymindisbuteonidis* as a nomen novum for *Falcopalliatus* Temminck, 1822.

***Leucopternissuperciliaris*** Von Pelzeln, 1861: 10.

= *Leucopterniskuhli* Bonaparte, 1850.

Syntype, RMNH.AVES.90963, adult female, mounted skin. Loc.: Para, Brazil. Leg.: J. Natterer. Ex: NMW, 1862.

According to Schifter et al. (2007: 72) Natterer collected only three specimens, two of which are in the NMW (NMW 40231 and 40232). A third was sent in exchange to the RMNH. Schifter (in litt., 18 January 2000) gave 1835 as the possible year of collection.

***Macheiramphusalcinus*** Bonaparte, 1850c: 482.

***Machaerhamphusalcinus*** Westerman, 1851: 29.

= *Macheiramphusalcinusalcinus* Bonaparte, 1850.

Holotype, RMNH.AVES.87232, adult, sex unknown, mounted skin. Loc.: Malacca [= Malaysia]. Ex: G.A. Frank.

The spelling of the genus name and authorship has long been debated. Bonaparte (1850c) described it in 1850 as *Macheiramphusalcinus*. Westerman (1851: 29) one year later as *Machaerhamphusalcinus*. Westerman’s name was used for more than a century because it was believed to have been published in 1848. Deignan (1960: 121) pointed out that Bonaparte had priority and all major works after that date have followed Deignan by using Bonaparte’s name.

**Falco (Nisus) polyzonus** Rüppell, 1836: 36, pl. 15.

= *Melieraxmetabatesmetabates* Heuglin, 1861.

Paralectotypes, RMNH.AVES.90937–90938.

The lectotype was designated by Steinbacher (1949: 104) and is in the SMF (SMF 12606). Paralectotypes are in the SMF, NMW, and NHM (see Steinheimer 2005a: 236; Schifter et al. 2007: 65–66). See Steinheimer (2005b: 171–172) for more information about nomenclature and taxonomy of Rüppell’s name.

***Milvusmelanoti****s* Temminck & Schlegel, 1844: 14, pl. 5, pl. 5B.

= *Milvusmigranslineatus* (Gray, 1831).

Syntype, RMNH.AVES.87233, adult male, mounted skin. Loc.: Japan. Leg.: -, 1844.

Syntype, RMNH.AVES.87234, adult female, mounted skin. Loc.: Japan. Leg.: Ph.F.B. von Siebold.

Syntype, RMNH.AVES.87235, adult, sex unknown, mounted skin. Loc.: Japan. Leg.: Ph.F.B. von Siebold.

Syntype, RMNH.AVES.87236, adult, sex unknown, mounted skin. Loc.: Japan. Leg.: H. Bürger.

Syntype, RMNH.AVES.87237, adult, sex unknown, mounted skin. Loc.: Japan. Leg.: H. Bürger.

Syntype, RMNH.AVES.87238, adult, sex unknown, mounted skin. Loc.: Japan. Leg.: Ph.F.B. von Siebold.

According to Holthuis and Sakai (1970) the text on page 14 as well as pl. 5 appeared in 1844. In the text adult birds of both sexes and a young male are mentioned. See also Dekker et al. (2001).

***Cathartesmonachus*** Temminck, 1823: livr. 38, pl. 222.

= *Necrosyrtesmonachus* (Temminck, 1823).

Although the RMNH and MNHN are mentioned in the original description, no specimens from Senegal collected before 1823 are in the RMNH or MNHN. See Voisin and Voisin (2001b: 629) who discussed a specimen considered as type of *Carthartesmonachus* but was collected in Sennar and given to the museum as late as 1834.

***Spizaëtusnipalensisbartelsi*** Stresemann, 1924: 431.

= *Nisaetusbartelsi* (Stresemann, 1924).

Holotype, RMNH.AVES.88921, adult male, skin. Loc.: Gunung Melattie, Java, [Indonesia], 30.iv.1907. Ex: M. Bartels (no. 4790)

***Falcocristatellus*** Temminck, 1824: livr. 48, pl. 282.

= *Nisaetuscirrhatuscirrhatus* (Gmelin, 1788).

According to the original description, the type specimens collected by Leschenault in “l’Inde et à Ceylan” are in the MNHN. See Voisin and Voisin (2001b: 628) who confirmed two type specimens (MNHN C.G.2000-1614 and C.G.2000-1615) and selected C.G.2000-1614 as lectotype.

***Falconiveus*** Temminck, 1822: livr. 22, pl. 127.

= *Nisaetuscirrhatuslimnaeetus* (Horsfield, 1821).

Syntype, RMNH.AVES.87254, adult female, mounted skin. Loc.: Java, [Indonesia], [1816–1822]. Leg.: C.G.C. Reinwardt.

Syntype, RMNH.AVES.87255, immature, mounted skin. Loc.: Java, [Indonesia], [1816–1822]. Leg.: C.G.C. Reinwardt.

Temminck (1822) mentioned several specimens from Java sent to him by Reinwardt. Two specimens in the collection, an adult female, and an immature, both listed here, are labelled “Reinwardt. Java”. The adult specimen RMNH.AVES.87254 is not the specimen illustrated by Temminck. We therefore assume that the illustrated specimen is no longer in the RMNH. Another syntype, received in exchange in March 1822, is in the NMW (NMW 85036; Schifter et al. 2007: 77).

***Spizaëtuscirrhatusvanheurni*** Junge, 1936: 24.

= *Nisaetuscirrhatusvanheurni* (Junge, 1936).

Holotype, RMNH.AVES.87256 (6651), adult female, skin. Loc.: Lasikin, Simalur, [Indonesia], 06.iv.1913. Leg.: E.R. Jacobson and W.C.van Heurn.

Paratypes, RMNH.AVES.87257–87260.

***Spizaëtoslanceolatus*** Temminck & Schlegel, 1844: 7.

= *Nisaetuslanceolatus* (Temminck & Schlegel, 1844).

Syntype, RMNH.AVES.87261, adult female, mounted skin. Loc.: Celebes [= Sulawesi], [Indonesia]. Leg.: C.G.C. Reinwardt.

Syntype, RMNH.AVES.87262, adult male, mounted skin. Loc.: Tondano, Celebes [= Sulawesi], [Indonesia]. Leg.: E.A. Forsten.

Syntype, RMNH.AVES.87263, immature male, mounted skin. Loc.: Tondano, Celebes [= Sulawesi], [Indonesia]. Leg.: E.A. Forsten.

Forsten stayed in Celebes twice between March 1840 and April 1842.

***Nisactus* [sic] *Nipalensis*** Hodgson, 1836b: 229, pl. 7.

= *Nisaetusnipalensisnipalensis* Hodgson, 1836.

Syntype, RMNH.AVES.193547, adult female, mounted skin. Loc.: Nepal. Leg.: B.H. Hodgson.

Syntype, RMNH.AVES.193548, adult, sex unknown, mounted skin. Loc.: Nepal. Leg.: B.H. Hodgson.

Syntype, RMNH.AVES.193551, adult, sex unknown, mounted skin. Loc.: Nepal. Leg.: B.H. Hodgson.

***Spizaëtosorientalis*** Temminck & Schlegel, 1844: 7, pl. 3.

= *Nisaetusnipalensisorientalis* (Temminck & Schlegel, 1844).

Holotype, RMNH.AVES.87264, immature female, mounted skin. Loc.: Japan. Leg.: H. Bürger.

Holotype by monotypy. Temminck and Schlegel (1844: 8) referred to this specimen as “the specimen of this species…”.

***Falcounicinctus*** Temminck, 1824: livr. 53, pl. 313.

= *Parabuteounicinctusunicinctus* (Temminck, 1824).

According to Voisin and Voisin (2001b: 623) the holotype is in the MNHN (MNHN C.G.2000-1618).

**Perniscristatusvar.celebensis** Wallace, 1868: 17.

= *Perniscelebensis* Wallace, 1868.

Holotype, RMNH.AVES.87239, adult female, mounted skin. Loc.: Bone, Celebes [= Sulawesi], [Indonesia], 18.xi.1863. Leg.: C.B.H. von Rosenberg (754).

Holotype by monotypy. Wallace (1868) based this name on a single bird illustrated by Schlegel (1866a, pl. 26, fig. 3). According to the index this is a female from Java. From the descriptions of both Schlegel and Wallace, Wallace meant fig. 4. The index states it is a male from Celebes. However, the specimen collected by Von Rosenberg is a female.

The date of collection given by Schlegel (1873a: 132) as “16 Mai 1867” is an error.

***Falcoptilorhynchus*** Temminck, 1821: livr. 8, pl. 44.

= *Pernisptilorhynchusptilorhynchus* (Temminck, 1821).

Following Dickinson et al. (2022) (see also Introduction of this catalogue under “What is new in this 2023 version”: 14) the specimen illustrated on pl. 44 is the holotype since the text that appeared on 29 July 1823 is irrelevant regarding type status. According to Temminck (1821), the illustrated specimen was in the MNHN, which is confirmed by Voisin and Voisin (2001a: 175) who listed MNHN C.G.1999-2141, collected by Leschenault de la Tour on Java in 1807, as the holotype.

***Falcogymnogenys*** Temminck, 1824: livr. 52, pl. 307.

= *Polyboroidesradiatus* (Scopoli, 1786).

Syntype, RMNH.AVES.87240, adult female, mounted skin. Loc.: Madagascar. Leg.: [P.B. Milius, Baudin expedition]. Ex: MNHN.

Voisin and Voisin (2001a) did not list a type specimen in the MNHN.

According to Schlegel (1862c: 54) RMNH.AVES.87240 was obtained from the MNHN and depicted on pl. 307. Temminck stated that it was collected by P.B. Milius, who took over command of the *Géographe* after the death of Baudin in 1803. Temminck mentioned that the specimens in the RMNH were collected in the interior of South Africa, but no matching specimens could be found.

***Falcopoecilonotus*** Temminck, 1820 (ex Cuvier MS): livr. 1, pl. 9.

= *Pseudasturalbicollisalbicollis* (Latham, 1790).

Following Dickinson et al. (2022) (see also Introduction of this catalogue under “What is new in this 2023 version”: 14) the specimen illustrated on pl. 9 is the holotype since the text that appeared on or before 25 June 1823 is irrelevant regarding type status. According to Temminck (1820), the illustrated specimen was in the MNHN. Voisin and Voisin (2001b: 622) selected MNHN C.G.2000-1629 as lectotype. However, Following Dickinson et al. (2022) there is no longer any reason to designate a lectotype as the illustrated specimen is the holotype.

***Cymindisleucopygus*** Spix, 1824: 7, pl. II.

= *Rostrhamussociabilissociabilis* (Vieillot, 1817).

Syntype, RMNH.AVES.87242, adult, sex unknown, mounted skin. Loc.: Brazil. Leg.: J.B. Spix.

Syntype, RMNH.AVES.87243, adult, sex unknown, mounted skin. Loc.: Brazil. Leg.: J.B. Spix.

***Asturinagularis*** Schlegel, 1862d: 4.

= *Rupornismagnirostrispucherani* (Verreaux & Verreaux, 1855).

Syntype, RMNH.AVES.87197, adult female, mounted skin. Loc.: Buenos Aires, Argentina. Ex: Maison Verreaux, 1858.

Syntype, RMNH.AVES.87198, immature female, mounted skin. Loc.: Rio de la Plata, Argentina. Leg.: -.

***Circaëtusbachacelebensis*** Schlegel, 1862e: 27.

= *Spilornisrufipectusrufipectus* Gould, 1858.

Syntype, RMNH.AVES.87251, adult female, mounted skin. Loc.: Tondano, Celebes [= Sulawesi], [Indonesia]. Leg.: E.A. Forsten.

Syntype, RMNH.AVES.87252, immature female, mounted skin. Loc.: Gorontalo, Celebes [= Sulawesi], [Indonesia]. Leg.: E.A. Forsten.

Syntype, RMNH.AVES.88920, adult male, cranium. Loc.: Celebes [= Sulawesi], [Indonesia]. Leg.: E.A. Forsten.

Forsten stayed in Celebes twice between March 1840 and April 1842.

***Circaëtussulaënsis*** Schlegel, 1866a: 38, pl. 23 figs 4, 5, 6.

= *Spilornisrufipectussulaensis* (Schlegel, 1866).

Syntype, RMNH.AVES.87244, adult male, mounted skin. Loc.: Sula Bessie, [Indonesia], i.1864. Leg.: H.A. Bernstein.

Syntype, RMNH.AVES.87245, adult female, mounted skin. Loc.: Sula Bessie, [Indonesia], i.1864. Leg.: H.A. Bernstein

Syntype, RMNH.AVES.87246, adult female, mounted skin. Loc.: Sula Bessie, [Indonesia], i.1864. Leg.: H.A. Bernstein.

Syntype, RMNH.AVES.87247, adult female, mounted skin. Loc.: Sula Bessie, [Indonesia], 24.xi.1864. Leg.: D.S. Hoedt.

Syntype, RMNH.AVES.87248, adult male, mounted skin. Loc.: Sula Mangole, [Indonesia], ii.1864. Leg.: H.A. Bernstein.

Syntype, RMNH.AVES.87249, adult male, mounted skin. Loc.: Sula Mangole, [Indonesia], 03.xii.1864. Leg.: D.S. Hoedt.

Syntype, RMNH.AVES.87250, immature female, mounted skin. Loc.: Sula Bessie, [Indonesia], i.1864. Leg.: H.A. Bernstein.

***Falcotyrannus*** Wied, 1820: 357.

= *Spizaetustyrannustyrannus* (Wied, 1820).

Van den Hoek Ostende et al. (1997: 41) erroneously listed RMNH.AVES.87265 (adult female, mounted skin. Loc.: Brazil. Leg.: A.P.M. zu Wied-Neuwied) as holotype (LeCroy et al. 2014: 311). See also Hoffman and Geller-Grimm (2013: 87).

***Falcoalbescens*** Daudin, 1800: 45.

***Falcolongicaudus*** Wilkes, 1810: 180.

= *Stephanoaetuscoronatus* (Linnaeus, 1766).

Syntype, RMNH.AVES.87266, adult male, mounted skin. Loc.: ‘Afrique australe, Pays d’Auteniquoi’ [South Africa]. Leg.: F. Levaillant.

According to the label this is the ”ind. tué et figu. par Le Vaill. Ois. d’Afr. 1 pl. 3”. Levaillant named this bird “Le Blanchard” and described how he, after three weeks of observing the behaviour of a pair, was able to collect both. His description formed the basis for both *Falcoalbescens* Daudin, 1800, and *Falcolongicaudus* Wilkes, 1810. Both authors translated the description of Levaillant.

***Vulturimperialis*** Temminck, 1827: livr. 72, pl. 426.

= *Torgostracheliotus* (Forster, 1791).

Holotype, RMNH.AVES.87163, adult, sex unknown, mounted skin. Loc.: Africa. Leg.: -.

Holotype by monotypy. Temminck (1827) wrote: “the subject of our illustration…”. He also referred to Levaillant’s “Le Chincou” (1796: pl. 12) but claimed that the depicted bird in Levaillant was from a specimen in captivity, which was not preserved. According to Temminck the specimen in the RMNH originated from Bengal, but also indicated that this is uncertain.

***Vulturægypius*** Rüppell, 1830: 377 (not Temminck, 1826: livr. 69, pl. 407).

= *Torgostracheliotus* (Forster, 1791).

Syntype, RMNH.AVES.87164, adult, sex unknown, mounted skin. Loc.: Nubia. Leg.: W.P.E.S. Rüppell.

Van den Hoek Ostende et al. (1997: 30) listed *Vulturaegypius* Temminck (1826) as a scientific name. Temminck (1826) did, however, not use *aegypius* as a specific epithet but as a vernacular name used by Savigny. This is evident from its placement next to the vernacular name used by Levaillant, later in the text confirmed by Temminck, and by the scientific name *Vulturauricularis* (Daud.) in the subheading and the ‘Tableau Méthodique’. Rüppell (1830: 377; 1835: 45) was under the impression that he was dealing with a scientific name and used æ*gypius* in combination with *Vultur* in that sense. In his reply to Rüppell, Temminck (1832: supplement to text on genus *Vultur*, livr. 89) also used this combination.

***Asturmacrourus*** Hartlaub, 1855: 353.

**Accipiter (Urotriorchis) amadoni** Wolters, 1979: 440 (nomen novum).

= *Urotriorchismacrourus* (Hartlaub, 1855).

Holotype, RMNH.AVES.87267, “male” (error: immature female), relaxed mount. Loc.: Dabocrom, Côte d’Or [= Ghana]. Leg.: H.S. Pel, 1842.

Holotype by monotypy. Hartlaub (1855) published a manuscript name by Temminck. The description is based on a single specimen.

Wolters (1979) created his nomen novum for *Asturmacrourus* Hartlaub, 1855, which was incorrectly believed to be pre-occupied by *Accipitermacrourus* Gmelin, 1771 (= *Circusmacrourus* (Gmelin, 1771)).

### ﻿STRIGIFORMES

#### ﻿Tytonidae Mathews, 1912

***Phodilusbadiusparvus*** Chasen, 1937: 216.

= *Phodilusbadiusparvus* Chasen, 1937.

Holotype, RMNH.AVES.14021, adult female, skin. Loc.: Kampong Ajer Saga, Billiton, [Indonesia], 05.xi.1935. Leg.: F.J. Kuiper. Ex: MZB, 1952 (MZB 16315).

Paratype, ZMA.AVES.47867.

***Strixfurcata*** Temminck, 1827: livr. 73, pl. 432.

= *Tytofurcatafurcata* (Temminck, 1827).

Holotype, RMNH.AVES.88272, adult, sex unknown, mounted skin. Loc.: Cuba, [1822–1824]. Leg.: E. Pöppig.

Holotype by monotypy.

Pöppig visited Cuba between 1822 and 1824 (Stafleu 1969).

***Strixinexspectata*** Schlegel, 1879a: 50.

= *Tytoinexspectata* (Schlegel, 1879).

Lectotype, RMNH.AVES.88273, adult female, mounted skin. Loc.: Minahassa, Celebes [= Sulawesi], [Indonesia], 1878. Leg.: S.C.J.W. van Musschenbroek.

Schlegel (1879a) did not specify how many specimens were available to him. Van den Hoek Ostende et al. (1997: 148) erroneously listed this specimen as holotype, thereby designating it the lectotype. No other type specimens are present in the RMNH.

***Tytonovaehollandiaecalabyi*** Mason, 1983: 126.

= *Tytonovaehollandiaecalabyi* Mason, 1983.

Holotype, RMNH.AVES.42474, adult male, skin. Loc.: Paal Poetih near Merauke, [Papua], [Indonesia], 22.iv.1960. Leg.: A. Hoogerwerf.

Paratype, RMNH.AVES.42475.

***StrixRosenbergii*** Schlegel, 1866b: 181.

= *Tytorosenbergiirosenbergii* (Schlegel, 1866).

Syntype, RMNH.AVES.88274, adult male, mounted skin. Loc.: Modelido, Celebes [= Sulawesi], [Indonesia], 15.v.1863. Leg.: C.B.H. von Rosenberg.

Syntype, RMNH.AVES.88275, adult male, mounted skin. Loc.: Bone, Celebes [= Sulawesi], [Indonesia], 16.xi.1863. Leg.: C.B.H. von Rosenberg.

Syntype, RMNH.AVES.88276, adult female, mounted skin. Loc.: Gorontalo, Celebes [= Sulawesi], [Indonesia], 28.iii.1864. Leg.: C.B.H. von Rosenberg.

Syntype, RMNH.AVES.89433, adult, sex unknown, cranium. Loc.: Celebes [= Sulawesi], [Indonesia], 1840. Leg.: E.A. Forsten.

Syntype, RMNH.AVES.89434, adult, sex unknown, cranium. Loc.: Celebes [= Sulawesi], [Indonesia], 1840. Leg.: E.A. Forsten.

Possible syntype, RMNH.AVES.195735, juvenile female, mounted skin. Loc.: Philippines. Ex: Verreaux, 1863 (= *Tytolongimembrisamauronota* (Cabanis, 1872).

Schlegel (1866b) based this new species on specimens collected by Von Rosenberg, two skulls collected by Forsten and immatures received from Verreaux. RMNH.AVES.195735 from Verreaux from the Philippines represents a different species and was overlooked by Van den Hoek Ostende et al. (1997: 149). A lectotype from Von Rosenberg’s series needs to be selected.

Forsten stayed in Celebes twice between March 1840 and April 1842.

***StrixtenebricosaArfaki*** Schlegel, 1879c: 101.

= *Tytotenebricosaarfaki* (Schlegel, 1879).

Holotype, RMNH.AVES.88277, adult male, mounted skin. Loc.: Hattam, [Papua], [Indonesia], 06.xii.1875. Leg.: [W.H. Woelders].

The collector most likely was missionary Woelders, who returned to the Netherlands in February 1879, on leave from Andai, New Guinea, bringing his collection with him (Adriani 1895).

#### ﻿Strigidae

***Ciccabagisella*** Bonaparte, 1850a: 44.

= *Aegoliusharrisiiharrisii* (Cassin, 1849).

Lectotype, RMNH.AVES.88278, adult, sex unknown, mounted skin. Loc.: Colombia. Ex: G.A. Frank.

Schlegel (1862h: 44) designated the lectotype.

***Otuscapensismajor*** Schlegel, 1873a: 3.

***Asiohelvolahova*** Stresemann, 1922: 64 (nomen novum).

= *Asiocapensishova* Stresemann, 1922.

Holotype, RMNH.AVES.88279, adult male, mounted skin. Loc.: Bombetok Bay, Madagascar, 1870. Leg.: D.C. van Dam.

Stresemann (1922) introduced *Asiohelvolahova* as nomen novum for *Otuscapensismajor* Schlegel, preoccupied by *Otusmajor* C.L. Brehm (= *Asioo.otus*).

***Strixbrama*** Temminck, 1821: livr. 12, pl. 68.

= *Athenebramabrama* (Temminck, 1821).

Following Dickinson et al. (2022) (see also Introduction of this catalogue under “What is new in this 2023 version”: 14) the specimen illustrated on pl. 68 is the holotype since the text that appeared on 27 September 1823 is irrelevant regarding type status. According to Temminck (1821) the holotype is in the MNHN.

***NoctuaTarayensis*** Hodgson, 1836c: 175.

= *Athenebramaindica* (Franklin, 1831).

Syntype, RMNH.AVES.197743, adult, sex unknown, mounted skin. Loc.: “Indostan”, Nepal. Leg.: B.H. Hodgson.

Possible syntype, RMNH.AVES.197745, adult, sex unknown, mounted skin. Loc.: Nepal. Leg.: B.H. Hodgson.

RMNH.AVES.197743 has “Indostan” written under the stand in Temminck’s handwriting, which refers to the lowlands, not the Himalayas, and therefore fits Hodgsons’ description of *Tarayensis*. RMNH.AVES.197745, however, only refers to Nepal and might therefore have its origin in either the lowlands or the Himalayas from where Hodgson described *Noctuatubiger* Hodgson, 1836c: 175. As Hodgson did not give any clear differences between *Tarayensis* and *tubiger*, we have listed it here as a possible syntype for *Tarayensis*. Both names are senior synonyms of *Athenebramaindica* (Franklin, 1831).

Another syntype is in the NHM (Warren 1966: 291; NHMUK 1843.1.13.154).

***Strixgrallaria*** Temminck, 1822: livr. 25, pl. 146.

= *Athenecuniculariagrallaria* (Temminck, 1822).

Syntype, RMNH.AVES.88349, adult, sex unknown [male], mounted skin. Loc.: [Ytararé], Brazil, [13.viii.1820]. Leg.: [J. Natterer].

Temminck (1822) described only the male of this species and depicted RMNH.AVES.88349. Schifter et al. (2007: 197) confirmed that RMNH.AVES.88349 is a male, collected by Natterer in Ytararé on 13 August 1820 (Von Pelzeln 1870). They also listed three additional syntypes in the NMW (NMW 40517, 40518, and 44031). According to the original description other syntypes are located in the NHM, although the name is not mentioned in Warren (1966).

***Strixsonnerati*** Temminck, 1820: livr. 4, pl. 21.

= *Athenesuperciliaris* (Vieillot, 1817).

Following Dickinson et al. (2022) (see also Introduction of this catalogue under “What is new in this 2023 version”: 14) the specimen illustrated on pl. 21 is the holotype since the text that appeared later is irrelevant regarding type status. According to Temminck (1820) the illustrated specimen was in the MNHN where it is listed under MNHN-ZO-2010-265.

***Noctuapollenii*** Schlegel, 1865c: 81.

= *Athenesuperciliaris* (Vieillot, 1817).

Holotype, RMNH.AVES.88333, adult female [male], mounted skin. Loc.: Syrangene, N.W. Madagascar, 18.x.1864. Leg.: F.P.L. Pollen and D.C. van Dam.

Pages 1 to 180 of Vol. 3, ‘Nederlandsch Tijdschrift voor de Dierkunde’ were published in 1865 (Pieters and Dickinson 2005: 107).

***Strixafricana*** Temminck, 1821: livr. 9, pl. 50.

= *Buboafricanusafricanus* (Temminck, 1821).

Following Dickinson et al. (2022) (see also Introduction of this catalogue under “What is new in this 2023 version”: 14) the specimen illustrated on pl. 50 is the holotype since the text that appeared later is irrelevant regarding type status. According to Temminck (1821) the illustrated specimen was in the MNHN, where MNHN-ZO-2010-258, listed as syntype, might prove to be the illustrated specimen and hence the holotype.

***Strixlactea*** Temminck, 1820: livr. 1, pl. 4.

= *Bubolacteus* (Temminck, 1820).

Livraison 1 was published in August 1820, the text appeared on 25 December 1824. The holotype (by monotypy) is in the MNHN.

***Buboverreauxi*** Bonaparte, 1850a: 49.

= *Bubolacteus* (Temminck, 1820).

Syntype, RMNH.AVES.88281, adult male, mounted skin. Loc.: Cap, [South Africa]. Leg.: H.B. van Horstok.

Syntype, RMNH.AVES.88282, adult female, mounted skin. Loc.: Cap, [South Africa]. Leg.: H.B. van Horstok.

Syntype, RMNH.AVES.88283, immature, mounted skin. Loc.: Cap, [South Africa]. Leg.: H.B. van Horstok.

***Buboleucostictus*** Hartlaub, 1855: 354.

= *Buboleucostictus* Hartlaub, 1855.

Syntype, RMNH.AVES.88284, adult male, mounted skin. Loc.: Dabocrom, Côte d’Or [= Ghana]. Leg.: H.S. Pel.

Syntype, RMNH.AVES.88285, adult female, mounted skin. Loc.: Dabocrom, Côte d’Or [= Ghana]. Leg.: H.S. Pel.

***Bubofasciolatus*** Hartlaub, 1855: 354.

= *Bubopoensis* Fraser, 1854.

Syntype, RMNH.AVES.88286, adult male, mounted skin. Loc.: Dabocrom, Côte d’Or [= Ghana]. Leg.: H.S. Pel.

Syntype, RMNH.AVES.88287, adult female, mounted skin. Loc.: Dabocrom, Côte d’Or [= Ghana]. Leg.: H.S. Pel.

Syntype, RMNH.AVES.88288, adult male, mounted skin. Loc.: Dabocrom, Côte d’Or [= Ghana]. Leg.: H.S. Pel.

***Strixstrepitans*** Temminck, 1823: livr. 30, pl. 174.

= *Bubosumatranusstrepitans* (Temminck, 1823).

Lectotype, RMNH.AVES.88291, adult male, mounted skin. Loc.: Java, [Indonesia]. Leg.: -.

RMNH.AVES.88291 is not the holotype as erroneously listed in Van den Hoek Ostende et al. (1997: 151) but has therefore now to be considered lectotype. According to the original description, paralectotypes are in the MNHN.

***Buboorientalisminor*** Schlegel, 1862g: 13.

= *Bubosumatranussumatranus* (Raffles, 1822).

Syntype, RMNH.AVES.88289, adult female, mounted skin. Loc.: Banka, [Indonesia]. Leg.: J.F.R.S. van den Bossche, 1861.

Syntype, RMNH.AVES.88290, adult male, mounted skin. Loc.: Banka, [Indonesia]. Leg.: J.F.R.S. van den Bossche, 1861.

***Bubosumatranustenuifasciatus*** Mees, 1964a: 116.

= *Bubosumatranustenuifasciatus* Mees, 1964.

Holotype, RMNH.AVES.35476 (4617), adult male, skin. Loc.: Rantau, S.E. Borneo, [Indonesia], 22.ii.1916. Leg.: F.C.E. van der Putten, 7.x.1919.

Paratypes, RMNH.AVES.88292–88298.

***Strixmacrorhyncha*** Temminck, 1821: livr. 11, pl. 62.

= *Bubovirginianus* subsp.

Following Dickinson et al. (2022) (see also Introduction of this catalogue under “What is new in this 2023 version”: 14) the specimen illustrated on pl. 62 is the holotype since the text that appeared later is irrelevant regarding type status. Any listing of syntypes for this name in Van den Hoek Ostende et al. (1997: 151) or any other type catalogue is erroneous. The specimens listed in Van den Hoek Ostende et al. (1997: 151; RMNH.AVES.88299 and 88300) have therefore no type status. According to Temminck (1821), the illustrated specimen is in the MNHN, where MNHN-ZO-2010-256 had been selected as lectotype; the specimen needs confirmation as being the illustrated specimen and hence the holotype.

***Strixferruginea*** Wied, 1820: 104.

= *Glaucidiumbrasilianumbrasilianum* (Gmelin, 1788).

Syntype, RMNH.AVES.88304, adult, sex unknown, mounted skin. Loc.: [Paulista], [Bahia], Brazil, [ix.1815]. Leg.: A.P.M. zu Wied-Neuwied.

Wied (1820) described this species in a footnote. He wrote that several small owls, called “cabure” by natives, were shot late September 1815 at Paulista, ca. 15 miles northeast of Macahe, Bahia, Brazil.

Other syntypes are in the AMNH (AMNH 6895 and 6343; Greenway 1978: 127).

***Strixinfuscata*** Temminck, 1820: 97.

***Strixpasserinoides*** Temminck, 1825: livr. 58, pl. 344.

= *Glaucidiumbrasilianumbrasilianum* (Gmelin, 1788).

Possible holotype for *infuscata*, syntype for *passerinoides*, RMNH.AVES.88306, adult, sex unknown, mounted skin. Loc.: Brazil. Leg.: J. Natterer.

Schifter et al. (2007: 196) had doubts about the type status of RMNH.AVES.88306 for *infuscata* Temminck. According to Natterer’s handwritten record cards, it was part of the third delivery which came to the NMW in 1821, one year after the description of *infuscata*. There is no other specimen in the collection which could fit as a type.

According to the original description other syntypes of *passerinoides* Temminck are in the MNHN and in the collection of Wied.

Peters’ name for *passerinoides* Temminck was erroneously given as *G.b.phaloenoides* (Daudin, 1800) by Van den Hoek Ostende et al. (1997: 152).

***Strixspadicea*** Temminck, 1821: livr. 17, pl. 98.

= *Glaucidiumcastanopterum* (Horsfield, 1821).

Holotype, RMNH.AVES.197111, adult, sex unknown, mounted skin. Loc.: Java, [Indonesia], [iv.1816–iii.1822]. Leg.: C.G.C. Reinwardt.

Following Dickinson et al. (2022) (see also Introduction of this catalogue under “What is new in this 2023 version”: 14) we have selected the holotype based on pl. 98, since the text that appeared later is irrelevant regarding type status. Any listing of syntypes for this name in the MNHN and/or NHM is erroneous. A specimen was sent to the NMW in 1823 (1823.LXXXVIII.5) and in April 1830 (1830.II.5).

Reinwardt visited Java several times between April 1816 and March 1822.

***Strixpumila*** “Illiger” Temminck, 1821: livr. 7, pl. 39.

***Strixminutissima*** Wied, 1830: 242.

= *Glaucidiumminutissimum* (Wied, 1830).

Holotype for *pumila*, syntype for *minutissima*, RMNH.AVES.88309, adult female, mounted skin. Loc.: Brazil. Leg.: -.

Temminck (1821) published pl. 39 with the scientific name on the wrapper in 1821, well before the text was published in 1823 (Dickinson 2001: 45–46), making the depicted specimen, which agrees with RMNH.AVES.88309, the holotype of *Strixpumila* Temminck, 1821.

A recommendation to the ICZN is in preparation to suppress *Strixpumila* Temminck, 1821, in favour *Strixminutissima* Wied, 1830 (= *Glaucidiumminutissimum* (Wied, 1830) because of prevailing usage unless the available evidence changes and shows that the name *pumila* has been used as valid since 1899.

***Strixlicua*** Lichtenstein, 1842: 12.

***Noctuaperlatacapensis*** Schlegel, 1862h: 37.

= *Glaucidiumperlatumlicua* (Lichtenstein, 1842).

Syntype, RMNH.AVES.88314, adult, sex unknown, mounted skin. Loc.: “Cafrerie”, [South Africa]. Ex: MfN.

Syntype, RMNH.AVES.88315, adult, sex unknown, mounted skin. Loc.: “Cafrerie”, [South Africa]. Leg.: G.L.E. Krebs. Ex: MfN, 1842.

***Strixperlata*** Vieillot, 1818d: 26.

= *Glaucidiumperlatumperlatum* (Vieillot, 1818).

Lectotype, RMNH.AVES.88312, adult male, mounted skin. Loc.: Senegal. Ex: Raye van Breukelerwaert.

Vieillot (1818d) based his description on “La Chouette Chevêchette Perlée” by Levaillant (1806–1807: pl. 284). Levaillant had seen this species in the collection of Raye van Breukelerwaert but doesn’t specify the number of specimens. The listing as holotype in Van den Hoek Ostende et al. (1997: 154) is therefore erroneous but constituted a lectotype designation. In the catalogue accompanying the sale of this collection the species is not listed (Van Cleef 1827: 5).

***Strixoccipitalis*** Temminck, 1821: livr. 6, pl. 34.

= *Glaucidiumperlatumperlatum* (Vieillot, 1818).

Holotype, RMNH.AVES.88313, adult female, mounted skin. Loc.: Senegal. Leg.: -.

Following Dickinson et al. (2022) (see also Introduction of this catalogue under “What is new in this 2023 version”: 14) we have selected the holotype based on the plate since the text that appeared in 1823 is irrelevant regarding type status. Also, according to Schlegel (1862g: 37) RMNH.AVES.88313 is depicted on pl. 34. Any listing of syntypes for this name in Van den Hoek Ostende et al. (1997: 154) or any other type catalogue is erroneous. RMNH.AVES.88312 has therefore no type status for *occipitalis* Temminck. Temminck (1821) also referred to specimens in the MNHN which no longer have type status.

***Strixsylvatica*** “Müller” Bonaparte, 1850a: 40.

***Glaucidiumbrodieiperitum*** Peters, 1940: 133 (nomen novum).

= *Taenioptynxsylvaticussylvaticus* (Bonaparte, 1850).

Lectotype, RMNH.AVES.88307, adult male, relaxed mount. Loc.: Batang Singalang, W. Sumatra, [Indonesia], 1834. Leg.: S. Müller.

Bonaparte (1850a) gave no indication of the number of specimens available to him. The listing as holotype in Van den Hoek Ostende et al. (1997: 153) is therefore erroneous but constituted a lectotype designation.

Peters (1940) introduced *peritum* as an unnecessary nomen novum for *Strixsylvatica* Bonaparte, 1850.

***Bubolettii*** Büttikofer, 1889a: 34.

= *Jubulalettii* (Büttikofer, 1889).

Lectotype, RMNH.AVES.88316, adult, sex unknown, skin. Loc.: Du Queah River, Pessy Country, Liberia, 03.xii.1887. Leg.: Lett. Ex: F.X. Stämpfli, 1888.

Büttikofer (1889a) gave no indication of the number of specimens available to him. The listing as holotype in Van den Hoek Ostende et al. (1997: 154) is therefore erroneous but constituted a lectotype designation.

***Cultrunguis Flavipes*** Hodgson, 1836d: 364 pl. 26.

= *Ketupaflavipes* (Hodgson, 1836).

Syntype, RMNH.AVES.196655, sex unknown, mounted skin. Loc.: Nepal. Leg. B.H. Hodgson.

***Ketupaminor*** Büttikofer, 1896c: 165.

***Buboketupubüttikoferi*** Chasen, 1935: 84 (nomen novum).

= *Ketupaketupuminor* Büttikofer, 1896.

Syntype, RMNH.AVES.88317, adult male, skin. Loc.: Gunung Sitolie, Nias, [Indonesia], 17.i.1896. Leg.: J.Z. Kannegieter. Ex: J.R.H. Neervoort van der Poll, vii.1896.

Syntype, RMNH.AVES.88318, adult female, skin. Loc.: Lahagu, Nias, [Indonesia], 14.ii.1896. Leg.: J.Z. Kannegieter. Ex: J.R.H. Neervoort van der Poll, vii.1896.

Chasen (1935) introduced *büttikoferi* as a nomen novum for *Ketupaminor* Büttikofer, 1896, because he regarded *Ketupa* synonymous with *Bubo*, for which the specific epithet *minor* was already in use.

***StrixLeschenaulti*** Temminck, 1820: livr. 4, pl. 20.

= *Ketupazeylonensisleschenaulti* (Temminck, 1820).

Following Dickinson et al. (2022) (see also Introduction of this catalogue under “What is new in this 2023 version”: 14) the specimen illustrated on pl. 20 is the holotype since the text that appeared on 25 December 1824 is irrelevant regarding type status. According to Temminck (1820), the illustrated specimen is in the MNHN from where it has not yet been reported.

***Syrniummacabrum*** Bonaparte, 1850a: 53.

= *Megascopsalbogularismacabrus* (Bonaparte, 1850).

Lectotype, RMNH.AVES.88335, adult, sex unknown, mounted skin. Loc.: Colombia. Ex: Maison Verreaux.

Bonaparte (1850a) gave no indication of the number of specimens available to him. The listing as holotype in Van den Hoek Ostende et al. (1997: 157) is therefore erroneous but constituted a lectotype designation.

***Strixatricapilla*** “Natterer” Temminck, 1822: livr. 25, pl. 145.

= *Megascopsatricapilla* (Temminck, 1822).

Van den Hoek Ostende et al. (1997: 157) erroneously listed the holotype as present in the RMNH. Temminck (1822) mentioned a single specimen collected by Natterer in Brazil in the NMW, where it is still available (NMW 40468; Schifter et al. 2007: 192). According to Natterer’s handwritten register cards in the NMW, this is the only specimen which was available at the time of description. Eight other specimens, currently in the NMW, were collected later and received by the museum after 1830. The specimen listed in Schlegel (1862g: 22) and in Van den Hoek Ostende et al. (1997: 157), RMNH.AVES.88336 and 196291 (a male collected by J. Natterer, Ipanema, Brazil, 17 February 1819) were received under the name *Otusdecussatus* and do not belong to this series of nine. They therefore have no type status. See Dantas et al. (2021) for taxonomic review of the *Megascopsatricapilla* - *M.watsonii* complex.

***Otuscholibacaucae*** Hekstra, 1982: 60.

= *Megascopscholibacruciger* (von Spix, 1824).

Holotype, RMNH.AVES.8091, adult male, skin. Loc.: El Tambo, Cauca, Colombia, 25.vi.1938. Leg.: [K. von Schneidern], 30.xi.1938.

***Ephialtesargentina*** “Lichtenstein” Schlegel, 1862g: 21.

= *Megascopssanctacatarinae* (Salvin, 1897).

In a footnote to his entry of *Scopsbrasiliensis*, Schlegel (1862g: 21) tentatively referred Lichtenstein’s *Ephialtesargentina* (1854: 7) to this species. Lichtentstein published this name as a nomen nudum. The description by Schlegel is the first valid use of this name and therefore Schlegel is the author of *Ephialtesargentina*.

The taxonomic status of this taxon is currently under review (Dickinson, pers. comm., 2022) and is listed here, following IOC 11.2, tentatively as *Megascopssanctacatarinae* (Salvin, 1897), a name published 35 years after *argentina* Schlegel.

The two syntypes of *Ephialtesargentina* Schlegel are in the MfN (MZB 1204 and 1205).

***Scopsnovae-zelandiae*** Bonaparte, 1850a: 47.

= *Megascopswatsoniiwatsonii* (Cassin, 1849).

Lectotype, RMNH.AVES.88346, adult, sex unknown, mounted skin. Loc.: ? New Zealand. Leg.: -.

The locality of New Zealand is an error. *Megascopswatsonii* is a South American species. Bonaparte (1850a) gave no indication of the number of specimens available to him. Schlegel (1862g: 28) designated the lectotype. See Dantas et al. (2021) for taxonomic review of the *Megascopsatricapilla* - *M.watsonii* complex.

**Strix (Athene) guteruhi** Müller, 1845: 279.

= *Ninoxfusca* (Vieillot, 1817).

Syntype, RMNH.AVES.88319, adult male, relaxed mount. Loc.: Banny, Timor, [Indonesia], v.1829. Leg.: S. Müller.

Syntype, RMNH.AVES.88320, adult female, relaxed mount. Loc.: Banny, Timor, [Indonesia], x.1829. Leg.: S. Müller.

Syntype, RMNH.AVES.88321, adult female, relaxed mount. Loc.: Lelogama, Timor, [Indonesia], x.1829. Leg.: S. Müller.

***Strixmaugei*** Temminck, 1821: livr. 8, pl. 46.

= *Ninoxfusca* (Vieillot, 1817).

The holotype (by monotypy) is in the MNHN.

***Ninoxios*** Rasmussen, 1999: 458.

= *Ninoxios* Rasmussen, 1999.

Holotype, RMNH.AVES.84701, adult male, skin. Loc.: “Hill 1440”, Dumoga-Bone National Park, N. Sulawesi, Indonesia, 07.iv.1985. Leg.: F.G. and C.M. Rozendaal, xi.1985.

***Strixhirsutajaponica*** Temminck & Schlegel, 1845: 28, pl. 9B.

= *Ninoxjaponicajaponica* (Temminck & Schlegel, 1844) (error for 1845).

Syntype, RMNH.AVES.88329, adult, sex unknown, mounted skin. Loc.: Japan. Leg.: Ph.F.B. von Siebold.

Syntype, RMNH.AVES.88330, adult female, mounted skin. Loc.: Japan. Leg.: H. Bürger.

According to the data under the stand RMNH.AVES.88329 was collected by H. Bürger.

In this catalogue we follow Holthuis and Sakai (1970) over Mlíkovský (2012b) for the year of publication. This can have consequences on the status and selection of type specimens for this name if the plate was published prior to the description in the livraison. Taxa based upon holotypes are not affected but will differ where the name is based upon a series of specimens.

***Noctuaochracea*** Schlegel, 1866b: 183.

***Ninoxperversa*** Stresemann, 1938: 149 (nomen novum).

= *Ninoxochracea* (Schlegel, 1866).

Holotype, RMNH.AVES.88322, adult female, mounted skin. Loc.: Negri-Lama, Celebes [= Sulawesi], [Indonesia], 22.ix.1863. Leg.: C.B.H. von Rosenberg.

Holotype by monotypy. Schlegel (1866b) based his name on a single specimen. Stresemann (1938) introduced *Ninoxperversa* as an unnecessary nomen novum for *Noctuaochracea* Schlegel, 1866, which he considered preoccupied by *Noctuaochracea* Haworth, 1809, which is in fact a moth (Noctuidae, Lepidoptera).

***Noctuahirsutaphilippensis*** Schlegel, 1862h: 26.

= *Ninoxphilippensisphilippensis* Bonaparte, 1855.

Syntype, RMNH.AVES.88323, adult, sex unknown, mounted skin. Loc.: Lucon, Philippines. Leg.: H. Gevers, 1862.

Syntype, RMNH.AVES.88324, adult, sex unknown, mounted skin. Loc.: Philippines. Ex: G.A. Frank, 1861.

Syntype, RMNH.AVES.88325, adult, sex unknown, mounted skin. Loc.: Philippines. Leg.: -, 1861.

Schlegel (1862h) introduced this name, obviously unaware of the earlier identical name by Bonaparte (1855b: 655).

***Noctuaaruensis*** Schlegel, 1866f: 329.

= *Ninoxrufahumeralis* (Bonaparte, 1850).

Holotype, RMNH.AVES.88326, adult female, mounted skin. Loc.: Wokam, Aru Islands, [Indonesia], 12.iv.1865. Leg.: C.B.H. von Rosenberg.

Holotype by monotypy. Schlegel (1866f) based his name on a single specimen.

***Noctuafransenii*** Schlegel, 1866c: 256.

= *Ninoxrufahumeralis* (Bonaparte, 1850).

Holotype, RMNH.AVES.88327, adult female, mounted skin. Loc.: Waigeu, [Indonesia], 04.v.1863. Leg.: H.A. Bernstein.

Holotype by monotypy. Schlegel (1866c) based his name on a single specimen.

***Strixhirsutaminor*** Schlegel, 1873a: 24.

= *Ninoxscutulata* (Raffles, 1822).

Syntype, RMNH.AVES.197274, adult, sex unknown, mounted skin. Loc.: Banka, [Indonesia]. Leg.: J.E. Teysmann [error for J.E. Teijsmann], 1862 (= *Ninoxscutulatascutulata* (Raffles, 1822)).

Syntype, RMNH.AVES.197275, adult, sex unknown, mounted skin. Loc.: Banka, [Indonesia]. Leg.: J.E. Teysmann [error for J.E. Teijsmann]. 1862 (= *Ninoxscutulatascutulata* (Raffles, 1822)).

Syntype, RMNH.AVES.197276, adult, sex unknown, mounted skin. Loc.: Malacca. Ex: Deyrolle, 1867 (= *Ninoxscutulatascutulata* (Raffles, 1822)).

Syntype, RMNH.AVES.197310, adult female, mounted skin. Loc.: Banjermassing, Borneo, [Indonesia]. Leg.: C.A.L.M. Schwaner (= *Ninoxscutulataborneensis* (Bonaparte, 1850)).

Syntype, RMNH.AVES.197311, adult, sex unknown, mounted skin. Loc.: Banjermassing, Borneo, [Indonesia]. Leg.: C.A.L.M. Schwaner (= *Ninoxscutulataborneensis* (Bonaparte, 1850)).

Syntype, RMNH.AVES.197312, adult, sex unknown, mounted skin. Loc.: Borneo, [Indonesia]. Ex: F.P.L. Pollen, 1862 (= *Ninoxscutulataborneensis* (Bonaparte, 1850)).

Syntype, RMNH.AVES.88328, adult female, mounted skin. Loc.: Pontianak, Borneo, [Indonesia]. Leg.: P.M. Diard (= *Ninoxscutulataborneensis* (Bonaparte, 1850)).

***Strixhirsutaborneensis*** Bonaparte, 1850a: 41.

= *Ninoxscutulataborneensis* (Bonaparte, 1850).

Syntype, RMNH.AVES.88328, adult female, mounted skin. Loc.: Pontianak, Borneo, [Indonesia]. Leg.: P.M. Diard.

***Strixhirsuta*** Temminck, 1824: livr. 49, pl. 289.

= *Ninoxscutulatahirsuta* (Temminck, 1824).

Temminck (1824) reported syntypes in the MNHN and RMNH. No specimen(s) which could fit as type are in the RMNH.

***Ninoxnipalensis***Hodgson: 1837c: 23, pl. XIV.

= *Ninoxscutulatalugubris* (Tickell, 1833).

Syntype, RMNH.AVES.197263, adult, sex unknown, mounted skin. Loc.: Nepal. Leg.: B.H. Hodgson.

Syntype, RMNH.AVES.197265, adult, sex unknown, mounted skin. Loc.: Nepal. Leg.: B.H. Hodgson.

Syntype, RMNH.AVES.197267, adult, sex unknown, mounted skin. Loc.: Nepal. Leg.: B.H. Hodgson.

Other syntypes are in the NHM (Warren 1966: 206). This is also the type species for the genus *Ninox*.

***Athenesquamipila*** Bonaparte, 1850a: 41.

= *Ninoxsquamipila* (Bonaparte, 1850).

Syntype, RMNH.AVES.88331, adult, sex unknown, mounted skin. Loc.: Ceram, [Indonesia], [1842]. Leg.: E.A. Forsten.

Syntype, RMNH.AVES.88332, adult, sex unknown, mounted skin. Loc.: Ceram, [Indonesia], [1842]. Leg.: E.A. Forsten.

Another syntype is in the NMW (NMW 49751; Schifter et al. 2007: 196). Forsten visited Seram late 1842.

***NoctuaHoedtii*** Schlegel, 1871a: 3.

= *Ninoxtheomachatheomacha* (Bonaparte, 1855).

Holotype, RMNH.AVES.88334, adult female, skin. Loc.: Misool, [Indonesia], 16.v.1867. Leg.: D.S. Hoedt.

Holotype by monotypy. Schlegel (1871a) based his name on a single specimen.

***Pisorhinaangelinae*** Finsch, 1912: 156.

= *Otusangelinae* (Finsch, 1912).

Holotype, RMNH.AVES.45833, adult female, skin. Loc.: Pangerango, Preanger, Java, [Indonesia], 25.viii.1911. Ex: Bartels, 01.vi.1954.

***Otuscollari*** Lambert & Rasmussen, 1998: 207.

= *Otuscollari* Lambert & Rasmussen, 1998.

Paratypes, RMNH.AVES.84653 and 90749.

The holotype is in the SNMB, Braunschweig (Lambert and Rasmussen 1998). Another paratype is in the MTD (C2446).

***Strixnoctula*** “Reinwardt” Temminck, 1821: livr. 17, pl. 99.

= *Otuslempijilempiji* (Horsfield, 1821).

Syntype, RMNH.AVES.88341, adult male, mounted skin. Loc.: Java, [Indonesia]. Leg.: C.G.C. Reinwardt.

Syntype, RMNH.AVES.88342, adult female, mounted skin. Loc.: Java, [Indonesia]. Leg.: C.G.C. Reinwardt.

Following Dickinson et al. (2022) (see also Introduction of this catalogue under “What is new in this 2023 version”: 14) the specimen illustrated on pl. 99 is the holotype since the text that appeared later is irrelevant regarding type status. According to Temminck (1821) the illustrated specimen was in the RMNH. However, none of the RMNH specimens are a perfect fit with pl. 99 and we consider pl. 99 to be a composite of two specimens, listed here as syntypes. Temminck also referred to specimens in the NHM but any specimens there have no type status. Schifter et al. (2007: 193) listed a possible syntype in the NMW (NMW 66.696) which they received through an exchange with the RMNH in 1823. It is worth comparing this specimen with pl. 99. One was sent to the MfN in May 1827.

***ScopsLettia*** Hodgson, 1836c: 176.

= *Otuslettialettia* (Hodgson, 1836).

Syntype, RMNH.AVES.196093, adult, sex unknown, mounted skin. Loc.: Nepal. Leg.: B.H. Hodgson.

Syntype, RMNH.AVES.196095, adult, sex unknown, mounted skin. Loc.: Nepal. Leg.: B.H. Hodgson.

Syntype, RMNH.AVES.196097, adult, sex unknown, mounted skin. Loc.: Nepal. Leg.: B.H. Hodgson.

***Strixmagica*** Müller, 1841: 110.

= *Otusmagicusmagicus* (Müller, 1841).

Syntype, RMNH.AVES.88343, adult female, relaxed mount. Loc.: Amboina [= Ambon], [Indonesia], iv.1828. Leg.: S. Müller.

Syntype, RMNH.AVES.88344, adult male, relaxed mount. Loc.: Amboina [= Ambon], [Indonesia], iv.1828. Leg.: S. Müller.

***Otusmantis*** Temminck & Schlegel, 1844: 25.

= *Otusrufescensrufescens* (Horsfield, 1821).

Lectotype, RMNH.AVES.88347, adult male, mounted skin. Loc.: Pontianak, Borneo, [Indonesia]. Leg.: P.M. Diard.

Sharpe (1875: 103) designated the lectotype.

***Scopslongipennis*** “Lichtenstein” Kaup, 1853: 110.

= *Otusscopscycladum* (Tschusi, 1904).

Syntype, RMNH.AVES.90964, adult, mounted skin. Loc.: Syria, [vii.1824]. Leg.: W.F. Hemprich and C.G. Ehrenberg. Ex: MfN, 1863.

According to Stresemann (1962: 388) Hemprich and Ehrenberg collected three specimens in “Syria, Julio 1824” and sent them under Hemprich’s MS name *Strixstriolata* to the MfN. Two syntypes are still there (ZMB 1199 and 1201), whereas the third specimen was exchanged with the RMNH in 1863 (formerly ZMB 1200, now RMNH.AVES.90964). Later Kaup (1862: 223) restricted the type locality to Syria.

Apparently, Kaup’s name was never used as a valid name as it was questionable which subspecies the birds from this region belonged to. Vaurie (1965), Roselaar (1985), Dickinson (2003), and others considered the breeding birds from this area to be *O.s.cycladum* (Tschusi zu Schmidhoffen, 1904) and so did Roselaar after studying the type specimens of *Scopslongipennis* Kaup, 1853 (C. Roselaar, in litt., September 2005).

*Otuss.cycladum* is in prevailing usage, but to be qualified by the term nomen protectum the conditions of “the Code” (ICZN 1999), Art. 23.9.1. need to be met.

***Otussemitorques*** Temminck & Schlegel, 1844: 24, pl. 8.

= *Otussemitorquessemitorques* Temminck & Schlegel, 1844.

Syntype, RMNH.AVES.88337, adult female, mounted skin. Loc.: Japan. Leg.: Ph.F.B. von Siebold.

Syntype, RMNH.AVES.88338, adult male, mounted skin. Loc.: Japan. Leg.: Ph.F.B. von Siebold.

Syntype, RMNH.AVES.88339, adult female, mounted skin. Loc.: Japan. Leg.: Ph.F.B. von Siebold.

Syntype, RMNH.AVES.88340, adult female, mounted skin. Loc.: Japan. Leg.: Ph.F.B. von Siebold.

Syntype, RMNH.AVES.88305, adult, sex unknown, skeleton. Loc.: Japan. Leg.: Ph.F.B. von Siebold.

Temminck and Schlegel (1844) examined six specimens of which the above five are still present in the RMNH. Schifter et al. (2007: 192) listed a specimen in the NMW (NMW 53.849) but due to a presumed collecting date of 1862, the type status of this specimen is unclear. According to the data under the stand of RMNH.AVES.88337, it was collected by H. Bürger.

***Otusscopsafricana*** Temminck & Schlegel, 1844: 27.

= *Otussenegalensissenegalensis* (Swainson, 1837).

Syntype, RMNH.AVES.195821, adult, sex unknown, mounted skin. Loc.: South Africa. Leg.: -.

***Scopssiaoënsis*** Schlegel, 1873a: 13.

= *Otussiaoensis* (Schlegel, 1873).

Holotype, RMNH.AVES.88345, adult, sex unknown, skin. Loc.: Oudang [= Ondong], Siao [= Pulau Siau], [Indonesia]. Leg.: L.D.H. Renesse van Duivenbode, 1866.

Holotype by monotypy. Schlegel (1873a) listed a single specimen.

***Otusscopsjaponicus*** Temminck & Schlegel, 1844: 27, pl. 9.

= *Otussuniajaponicus* Temminck & Schlegel, 1844.

Syntype, RMNH.AVES.195812, adult, sex unknown, mounted skin. Loc.: Japan. Leg.: Ph.F.B. von Siebold.

Syntype, RMNH.AVES.195813, adult, sex unknown, mounted skin. Loc.: Japan. Leg.: Ph.F.B. von Siebold.

This name was published in 1844 (or 1845 according to Mlíkovský 2012b: 116), not 1850 as in Peters (Vol. IV: 1940: 91). These specimens are also the types for *Scopszorcaasiaticus* Schlegel, 1862.

***Scopszorcaasiaticus*** Schlegel, 1862g: 20.

= *Otussunia* (Hodgson, 1836).

Syntype, RMNH.AVES.195812, adult, sex unkown, mounted skin. Loc.: Japan. Leg.: Ph.F.B. von Siebold (= *Otussuniajaponicus* Temminck & Schlegel, 1844).

Syntype, RMNH.AVES.195813, adult, sex unkown, mounted skin. Loc.: Japan. Leg.: Ph.F.B. von Siebold (= *Otussuniajaponicus* Temminck & Schlegel, 1844).

Syntype, RMNH.AVES.195818, adult, sex unknown, mounted skin. Loc.: Nepal. Ex: Frank (= *Otussuniasunia* (Hodgson, 1836)).

Syntype, RMNH.AVES.195819, adult, sex unknown, mounted skin. Loc.: Nepal. Ex: Frank (= *Otussuniasunia* (Hodgson, 1836)).

***Strixleucotis*** Temminck, 1820: livr. 3, pl. 16.

= *Ptilopsisleucotis* (Temminck, 1820).

Following Dickinson et al. (2022) (see also Introduction of this catalogue under “What is new in this 2023 version”: 14) the specimen illustrated on pl. 16 is the holotype since the text that appeared on 25 December 1824 is irrelevant regarding type status. According to Temminck (1820) the specimen on pl. 16 was in the collection of Meiffren-Laugier de Chartrouse. Other specimens were said to be in the RMNH and MNHN. The holotype might now be in the MNHN from where it has not yet been reported.

***Strixpeli*** “Temminck” Bonaparte, 1850a: 44.

= *Scotopeliapeli* (Bonaparte, 1850).

Lectotype, RMNH.AVES.88348, adult male, mounted skin. Loc.: Rio Boutry, Ashantee, Côte d’Or [= Ghana]. Leg.: H.S. Pel.

Bonaparte (1850a) gave no indication of the number of specimens available to him. Schlegel (1862h: 24) designated the lectotype. Van den Hoek Ostende et al. (1997: 159) erroneously listed RMNH.AVES.88348 as holotype.

***Syrniumfulvescens*** Sclater & Salvin, 1868: 58.

= *Strixfulvescens* (Sclater and Salvin,1868).

Syntype, RMNH.AVES.88361, adult, sex unknown, mounted skin. Loc.: Guatemala. Leg.: O. Salvin. Ex: G.A. Frank.

Warren (1966: 105) listed another syntype in the NHM (NHMUK 1874.7.4.5).

***Strixhuhula*** Daudin, 1800: 190.

= *Strixhuhulahuhula* Daudin, 1800.

Syntype, RMNH.AVES.88301, adult, sex unknown, mounted skin. Loc.: Cayennes, [French Guiana]. Leg.: -.

Syntype, RMNH.AVES.88302, adult, sex unknown, mounted skin. Loc.: Cayennes, [French Guiana]. Leg.: -.

Daudin (1800) based his description on the description of ‘Le Huhul’ by Levaillant (Rookmaaker 1989). Levaillant had seen several specimens, both from his own collection as well as from the collections of J. Raye van Breukelerwaert and the painter Desmoulins. RMNH.AVES.88301 agrees well with the plate of Levaillant. This specimen probably originates from the collection of Raye van Breukelerwaert. RMNH.AVES.88302 is listed tentatively as a syntype. The data on the label of both specimens are the same.

Von Berlepsch (1908: 288) designated a specimen in the MNHN the lectotype, without specifying which specimen he selected which is not in accordance with the regulations of the ICZN and therefore invalid.

***Strixhylophila*** Temminck, 1825: livr. 63, pl. 373.

= *Strixhylophila* Temminck, 1825.

Lectotype, RMNH.AVES.88350, adult male, mounted skin. Loc.: [Ipanema], Brazil, [26-ix-1821]. Leg.: J. Natterer.

Von Berlepsch (1908: 288) designated the lectotype. Schifter et al. (2007: 198) listed four paralectotypes collected by Natterer in Ipanema in the NMW (NMW 40487–40490). According to the original description other paralectotypes are in the MNHN.

***Syrniumbartelsi*** Finsch, 1906: 63.

= *Strixleptogrammicabartelsi* (Finsch, 1906).

Holotype, RMNH.AVES.46109, adult female, skin. Loc.: Pangerango, Preanger, Java, [Indonesia], 03.xi.1902. Ex: Bartels, 01.vi.1954.

Holotype by monotypy.

***Strixleptogrammicachaseni*** Hoogerwerf & de Boer, 1947: 140.

= *Strixleptogrammicachaseni* Hoogerwerf & de Boer, 1947.

Holotype, RMNH.AVES.14020, adult male, skin. Loc.: Penjabin Mine, W. Billiton [= Belitung], [Indonesia], 30.iv.1936. Leg.: F.J. Kuiper. Ex: MZB, 12.i.1950 (MZB 16324).

Possible paratype, ZMA.AVES.47766.

Roselaar and Prins (2000: 106) tentatively listed a specimen formerly in the ZMA but now in the RMNH as a possible paratype (ZMA.AVES.47766).

***Strixleptogrammica*** Temminck, 1832: livr. 89, pl. 525.

= *Strixleptogrammicaleptogrammica* Temminck, 1832.

Holotype, RMNH.AVES.88352, immature, sex unknown, mounted skin. Loc.: Pontianak, Borneo, [Indonesia]. Leg.: P.M. Diard.

Van den Hoek Ostende et al. (1997: 147) listed two syntypes of *Strixleptogrammica*. However, Temminck (1832) described his new taxon based on a single specimen sent by Diard, which is therefore the holotype by monotypy. RMNH.AVES.88352 refers to pl. 525 on the label and fits the bird depicted on that plate. Also according to Schlegel (1862g: 21) it is the depicted specimen and type. RMNH.AVES.88351 listed in Van den Hoek Ostende et al. (1997: 147) has no type status.

Although the text to the plate suggests that this plate was published in livr. 88, it was actually part of livr. 89 (see Dickinson 2001: 45), dating this name not from 1831 as listed in Van den Hoek Ostende et al. (1997: 159), but from 1832.

***Ciccabamyrtha*** Bonaparte, 1850a: 44.

= *Strixleptogrammicamyrtha* (Bonaparte, 1850).

Syntype, RMNH.AVES.88353, adult male, mounted skin. Loc.: Sumatra, [Indonesia], [vii.1833–1835]. Leg.: S. Müller.

Syntype, RMNH.AVES.88354, adult female, mounted skin. Loc.: Sumatra, [Indonesia], [vii.1833–1835]. Leg.: S. Müller.

Syntype, RMNH.AVES.88355, adult female, mounted skin. Loc.: Sumatra, [Indonesia], [1835–1838]. Leg.: L. Hörner.

Syntype, RMNH.AVES.88356, immature, mounted skin. Loc.: Batang Singalang, Sumatra, [Indonesia], x.1834. Leg.: S. Müller.

According to the data under the stand RMNH.AVES.88354 is a male collected by Hörner. Müller visited West Sumatra from July 1833 until late 1835.

***Strixpagodarum*** Temminck, 1823: livr. 39, pl. 230.

= *Strixseloputoseloputo* Horsfield, 1821.

Syntype, RMNH.AVES.87878, immature, sex unknown, mounted skin. Loc.: Java, [Indonesia], [xii.1820–ix.1821]. Leg.: H. Kuhl and J.C. van Hasselt.

Other syntypes are in the MNHN. Schlegel (1862g: 22) restricted the type locality to Java.

Kuhl and van Hasselt collected together on Java from December 1820 until September 1821.

***Strixfuscescens*** Temminck & Schlegel, 1850: pl. 10.

***Strixrufescens*** Temminck & Schlegel, 1845: 30.

= *Strixuralensisfuscescens* Temminck & Schlegel, 1850 (not 1845).

Syntype, RMNH.AVES.88357, adult male, mounted skin. Loc.: Japan. Leg.: Ph.F.B. von Siebold.

Syntype, RMNH.AVES.88358, adult male, mounted skin. Loc.: Japan. Leg.: Ph.F.B. von Siebold.

Syntype, RMNH.AVES.88359, adult male, mounted skin. Loc.: Japan. Leg.: H. Bürger.

Syntype, RMNH.AVES.88360, adult female, mounted skin. Loc.: Japan. Leg.: H. Bürger.

Syntype, RMNH.AVES.88153, adult, sex unknown, skeleton. Loc.: Japan. Leg.: Ph.F.B. von Siebold.

This taxon was named in two different ways by Temminck and Schlegel. In the text of the Fauna Japonica (1845; 30) it was described as *Strixrufescens*, but on pl. 10 as *S.fuscescens*. The exact publication date of pl. 10 is unknown (Holthuis and Sakai 1970). It was not in fascicle 2 (1845) where the text appeared. The earliest possible date is 1847, but it could have been as late as 1850 (Dekker et al. 2001: 206). According to the Code, 1850 must therefore be used as the year of publication until there is evidence to the contrary (ICZN 1999, Art. 21.7.). The name *rufescens* was therefore published first, but is preoccupied by *Strixrufescens* Horsfield, 1821. The next available name is *fuscescens* which has been in use at least since Hartert (1913: 1021), who selected Kiuschin [= Kyushu] as type locality. For more information on dating and Siebold’s collection see Dekker et al. (2001) and Holthuis and Sakai (1970). See also Mlíkovský (2012b).

RMNH.AVES.88358 is labelled as male, but according to the stand it is a female. According to Schlegel (1862g: 11) RMNH.AVES.88359 is the depicted specimen on pl. X.

***Syrniumsquamulatum*** “Lichtenstein” Bonaparte, 1850a: 53.

= *Strixvirgatasquamulata* (Bonaparte, 1850).

Lectotype, RMNH.AVES.88303, immature, sex unknown, mounted skin. Loc.: Mexico, [1824–1829]. Leg.: F. Deppe. Ex: MfN.

Bonaparte (1850a) gave no indication of the number of specimens available to him. Van den Hoek Ostende et al. (1997: 152) erroneously listed RMNH.AVES.88303 as holotype, thereby designating it the lectotype.

### ﻿COLIIFORMES

#### ﻿Coliidae Sundevall, 1836

***Coliusnigricollis*** Vieillot, 1817a: 378.

= *Coliusstriatusnigricollis* Vieillot, 1817.

Holotype, RMNH.AVES.88469, adult, sex unknown, relaxed mount. Loc.: [Malymbe = Malembo], West Africa. Ex: Cabinet Temminck.

Vieillot (1817a) based his description on Levaillant’s “Coliou Rayé à Gorge Noire” (1807: pl. 259). According to Rookmaaker (1989: 206) Levaillant had seen six specimens from Angola and Malymbe and bought one from each provenance. He donated one to Temminck. This specimen is listed in Temminck (1807: 97) as no. 780 “Coliou à gorge noir de Malymbe - non décrit”. As Vieillot only mentioned Malymbe as locality in his description we consider this a case of holotype by monotypy.

***Coliuserythromelon*** Vieillot, 1817a: 378.

***Coliusguiriva*** Hartlaub, 1849: 3.

= *Urocoliusindicusindicus* (Latham, 1790).

Lectotype, RMNH.AVES.88468, adult, sex unknown, skin. Loc.: “Pays des Caffres” [South Africa]. Ex: Cabinet Temminck.

According to Rookmaaker (1989: 206), Vieillot (1817a) based his description on Levaillant’s “Coliou Quiriwa” (1807: 42, pl. 258). Hartlaub (1849: 3) published the name *Coliusguiriva* also based on Levaillant and on Temminck’s “Le coliou jou-nue d’Afrique pays des caffres - non decrit” (1807: 97).

Neither Levaillant nor Vieillot mentioned the number of specimens available to them. The listing in Van den Hoek Ostende et al. (1997: 174) as holotype was in error, but constituted a lectotype designation.

### ﻿TROGONIFORMES

#### ﻿Trogonidae

***TrogonMackloti*** Müller, 1836: 336, pl. IV, fig. 1.

= *Apalharpactesmackloti* (Müller, 1836).

Syntype, RMNH.AVES.88483, adult female, mounted skin. Loc.: Mt. Singgalang, Sumatra, [Indonesia], [v–xi.1834]. Leg.: S. Müller.

Syntype, RMNH.AVES.88484, adult female, mounted skin. Loc.: Sumatra, [Indonesia], [vi.1833–1835]. Leg.: S. Müller.

Syntype, RMNH.AVES.88485, adult male, mounted skin. Loc.: Sumatra, [Indonesia], [vi.1833–1835]. Leg.: S. Müller.

Not 1835 as in Peters’ Checklist. The 1835 volume was published in 1836 (see Richmond 1926: 141).

Müller visited West Sumatra from June 1833 until late 1835 and stayed in the area around Mt. Singgalang between May and November 1834.

***Trogonreinwardtii*** Temminck, 1822: livr. 21, pl. 124.

= *Apalharpactesreinwardtii* (Temminck, 1822).

Syntype, RMNH.AVES.88481, adult, sex unknown, mounted skin. Loc.: Java, [Indonesia]. Leg.: -.

Syntype, RMNH.AVES.88482, immature, sex unknown, mounted skin. Loc.: Java, [Indonesia]. Leg.: -.

Schifter et al. (2007: 240) listed a syntype in the NMW (NMW 65407). According to the original description other syntypes are in the MNHN (not listed in Voisin and Voisin 2009).

***TrogonNarina*** Stephens, 1815: 14.

= *Apalodermanarinanarina* (Stephens, 1815).

Syntype, RMNH.AVES.88470, adult male, relaxed mount. Loc.: “Cap”, [South Africa]. Ex: Cabinet Temminck.

Syntype, RMNH.AVES.88471, adult female, relaxed mount. Loc.: “Cap”, [South Africa]. Ex: Cabinet Temminck.

Stephens (1815) based his description on Levaillant’s (1806: 104) description of “Le Couroucou Narina”.

***Trogonardens*** Temminck, 1826: livr. 68, pl. 404.

= *Harpactesardensardens* (Temminck, 1826).

The holotype (by monotypy) was in the collection of Laugier, now in NHM (NHMUK 1837.6.10.603; pers. comm. Van Grouw, 19.i.2023).

***Trogondiardii*** Temminck, 1832: livr. 91, pl. 541.

= *Harpactesdiardiidiardii* (Temminck, 1832).

Syntype, RMNH.AVES.88472, adult male, mounted skin. Loc.: Sumatra [error for Borneo], [Indonesia]. Leg.: S. Müller [error for Diard].

There is confusion about the type specimens for this name. Van den Hoek Ostende et al. (1997: 175) listed three syntypes collected by Müller (including RMNH.AVES.87662 and 88264). However, Müller visited West Sumatra from June 1833 until late 1835 and Borneo not until 1836, so these specimens cannot be part of the type series. RMNH.AVES.88472 is labelled ‘Sumatra, Müller’, whereas RMNH.AVES.87661 is labelled ‘Borneo, Diard’. Apparently there has been a mix-up, since RMNH.AVES.88472 belongs to the Bornean race and RMNH.AVES.87661 to the Sumatran (G.F. Mees, 26.ii.1968 on label). We therefore only consider RMNH.AVES.88472 to be part of the type series of *diardii* Temminck, 1832.

Schifter et al. (2007: 240) listed a syntype in the NMW (NMW 43937) collected in Pandang, Sumatra (which is specifically mentioned by Temminck) by Henrici and received from the RMNH in 1833.

***Trogonduvaucelii*** Temminck, 1824: livr. 49, pl. 291.

= *Harpactesduvaucelii* (Temminck, 1824).

Syntype, RMNH.AVES.88473, adult male, mounted skin. Loc.: Sumatra, [Indonesia]. Leg.: S. Müller [error].

Müller is given as the collector. This must be an error, since he left the Netherlands for Indonesia on 21 December 1825. Temminck (1824) mentioned that he had only received male specimens. RMNH.AVES.88474, a female listed by Van den Hoek Ostende et al. (1997: 175) is therefore not part of the type series. In his original description Temminck referred to Duvaucel and specimens from Sumatra in the RMNH and MNHN (not listed in Voisin and Voisin 2009).

Schifter et al. (2007: 241) listed two possible syntypes in the NMW (NMW 65396 and 65397), but their status is questionable. Both specimens have Borneo as collecting locality which is not mentioned by Temminck and NMW 65396 is a female.

***Trogonflagrans*** Müller, 1836: 338, pl. 4, fig. 2.

= *Harpacteserythrocephalusflagrans* (Müller, 1836).

Syntype, RMNH.AVES.88475, adult male, mounted skin. Loc.: Mt. Singgalang, Sumatra [Indonesia, vii.1834]. Leg.: S. Müller.

Syntype, RMNH.AVES.88476, adult male, mounted skin. Loc.: Sumatra [Indonesia]. Leg.: -.

Syntype, RMNH.AVES.88477, adult female, mounted skin. Loc.: Mt. Singgalang, Sumatra [Indonesia, vii.1834]. Leg.: S. Müller.

Syntype, RMNH.AVES.88478, immature, sex unknown, mounted skin. Loc.: Sumatra [Indonesia, 1833–1835]. Leg.: S. Müller.

According to the data on the stand, RMNH.AVES.88475 and 88477 have been collected in July, RMNH.AVES.88478 in June. The year is not indicated. Müller made a trip to West Sumatra in the years 1833–1835, visiting the area of Mt. Singgalang from May until November 1834.

Schifter et al. (2007: 241) listed a possible syntype collected by Müller on Sumatra in the NMW (NMW 35218).

Not 1835 as in Peters’ Checklist. The 1835 volume was published in 1836 (see Richmond 1926: 141).

***TrogonTemminckii*** Gould, 1835: 29.

= *Harpacteskasumbakasumba* (Raffles, 1822) (if from Sumatra; see below).

Holotype, RMNH.AVES.88479, adult male, mounted skin. Loc.: Borneo? [error for Sumatra], [Indonesia]. Leg.: ?Diard [error for Van den Berg].

Gould (1835) introduced this new name for *Trogonfasciatus* as described and illustrated by Temminck (1825: pl. 321). The history of the RMNH specimens is confusing. Temminck (1825: pl. 321) identified them incorrectly as *Trogonfasciatus* Pennant, 1769 (from India and Sri Lanka) and gave Van den Berg as collector and Sumatra as type locality. These data were originally written under the stands. Later this was changed, in Temminck’s handwriting, in “Diard, Borneo”. Since Diard arrived in Borneo after 1825, this is clearly incorrect and G.F. Mees (26 February 1968) summarised this discrepancy on the labels of RMNH.AVES.88479 and 88480 and considered Van den Berg as collector and Sumatra as origin.

Since it was assumed that the birds came from Diard from Borneo, they were identified as *Harpacteskasumbaimpavidus* (Chasen & Kloss, 1931), an identification which seemed to be corroborated by their somewhat smaller size. However, if these birds were indeed collected by Van den Berg on Sumatra, as is most likely, they belong to the nominate race.

***Trogonoreskios*** Temminck, 1823: livr, 31, pl. 181.

= *Harpactesoreskiosoreskios* (Temminck, 1823).

Syntype, RMNH.AVES.88265, adult female, skin. Loc.: Java, [Indonesia], 1821. Leg.: H. Kuhl and J.C. van Hasselt.

According to the original description other syntypes are in the MNHN (not listed in Voisin and Voisin 2009). Schifter et al. (2007: 241) listed two possible syntypes in the NMW (NMW 65402 and 65403).

***Trogontemnurus*** Temminck, 1825: livr. 55, pl. 326.

= *Priotelustemnurustemnurus* (Temminck, 1825).

Syntype, RMNH.AVES.88486, adult, sex unknown, relaxed mount. Loc.: Cuba. Ex: Cabinet Temminck.

Temminck also referred to type material in the MNHN (not listed in Voisin and Voisin 2009) and in the collection of Drapiez (Brussels).

***Trogonmassena*** Gould, 1838a: III, pl. 16d.

= *Trogonmassenamassena* Gould, 1838.

Syntype, RMNH.AVES.88487, adult male, mounted skin. Loc.: Mexico. Ex: MNHN, 1838.

According to Gould (1838a) other types are in the MNHN (not listed in Voisin and Voisin 2009) and in the NMW (not listed in Schifter et al. 2007).

### ﻿BUCEROTIFORMES

#### ﻿Phoeniculidae

***Falcinelluscyanomelas*** Vieillot, 1819a: 165.

= *Rhinopomastuscyanomelascyanomelas* (Vieillot, 1819).

Syntype, RMNH.AVES.90968, adult female, relaxed mount. Loc.: “Pays des grands Namaquois” [= Namaqualand], [South Africa], [1781–1784]. Leg.: F. Levaillant. Ex: Cabinet Temminck.

According to the description, Vieillot (1819a) based his name on Levaillant’s plates (Levaillant, 1807: Promerops, pls 5 and 6, pp. 15–17, see Rookmaaker 1989: 209) and, consequently, specimens collected by Levaillant in Namaqualand. Vieillot might have copied his text from Temminck’s description of “Le promerops namaquoi” (Temminck 1807: 74, 217) as he did in other cases (see Hartlaub 1849; Stresemann 1953a). See Rookmaaker (1989) for more information about Levaillant’s relationship to Temminck and the RMNH.

#### ﻿Bucorvidae

***Buceroscarunculatusguineensis*** Schlegel, 1862a: 20.

= *Bucorvusabyssinicus* (Boddaert, 1783).

Syntype, RMNH.AVES.22162, adult female, mounted skin. Loc.: Accra, Ashanti, Côte d’Or [= Ghana], 1844. Leg.: H.S. Pel.

Syntype, RMNH.AVES.89436, adult male, skeleton. Loc.: Goudkust [= Ghana], 1844. Leg.: H.S. Pel.

***Buceroscarunculatuscafer*** Schlegel, 1862a: 20.

***Bucorvusschlegeli*** Roberts, 1926: 219 (nomen novum).

= *Bucorvusleadbeateri* (Vigors, 1825).

Syntype, RMNH.AVES.88652, adult male, mounted skin. Loc.: “Interieur Cafrérie”, [South Africa]. Ex: G.A. Frank, 1857.

Syntype, RMNH.AVES.22163, adult male, mounted skin. Loc.: Cafrérie Cap, [South Africa]. Ex: Bullock Museum, 1819.

Syntype, RMNH.AVES.22164, adult, sex unknown, mounted skin. Loc.: Africa. Ex: Cabinet Temminck.

Roberts introduced *schlegeli* as an unnecessary nomen novum for *Buceroscarunculatuscafer* Schlegel, 1862.

#### ﻿Bucerotidae

***Bucerosgaleritus*** Temminck, 1831: livr. 88, pl. 520.

= *Anorrhinusgaleritus* (Temminck, 1831).

Syntype, RMNH.AVES.22159, adult male, mounted skin. Loc.: Pontianak, Borneo, [Indonesia]. Leg.: P.M. Diard.

Possible syntype, RMNH.AVES.88643, adult male, mounted skin. Loc.: Sumatra, [Indonesia]. Leg.: S. Müller [?].

Possible syntype, RMNH.AVES.88644, adult female, mounted skin. Loc.: Sumatra, [Indonesia]. Leg.: S. Müller [?].

Possible syntype, RMNH.AVES.88645, immature female, mounted skin. Loc.: Batang Singalang, Sumatra, [Indonesia]. Leg.: S. Müller [?].

According to Schlegel (1862a: 8), RMNH.AVES.22159 is the specimen depicted on pl. 520. According to a later label by Schlegel, RMNH.AVES.88643–88645 were collected by Müller on Sumatra. That would mean that these specimens are not part of the type series, as Müller arrived in Sumatra in June 1833, two years after publication. However, the data on the bottom of the stand in Temminck’s handwriting does not mention Müller, so we list them here as possible syntypes.

***Bucerosconvexus*** Temminck, 1832: livr. 89, pl. 530.

= *Anthracocerosalbirostrisconvexus* (Temminck, 1832).

Lectotype, RMNH.AVES.22160, adult male [female], mounted skin. Loc.: Sumatra, [Indonesia]. Leg.: ?S. Müller.

As Müller did not arrive in Sumatra before June 1833 and Temminck published this name in 1832, there is doubt about the correctness of Müller as collector. Schlegel (1862a: 7) listed it as the type and as the figured specimen, which suggests it was not collected by Müller. This constituted, however, a lectotype designation.

See Dickinson (2001: 46) for dating: not 1831 as listed in Van den Hoek Ostende et al. (1997: 190). Skeleton RMNH.AVES.88648 listed in Van den Hoek Ostende et al. (1997: 190) is not part of the type series as Temminck made no reference to skeletal material.

***Bucerosviolaceus*** Wilkes, 1808: 479.

= *Anthracoceroscoronatus* (Boddaert, 1783).

Wilkes (1808) based his name on “Le Calao violet” by Levaillant (1801a: 39, pl. 19). Levaillant saw a bill of this new species in the cabinet of Temminck which is no longer present in the RMNH.

***Bucerosantracicus*** Temminck, 1832: livr. 89, pl. 529.

= *Anthracocerosmalayanus* (Raffles, 1822).

Syntype, RMNH.AVES.197861, adult female, mounted skin. Loc.: Borneo, [Indonesia], [1826]. Leg.: P.M. Diard.

Temminck (1832) referred to specimens from Sumatra and Borneo but failed to indicate in which collections the type specimens were kept. RMNH.AVES.197861 is the only specimen in the RMNH which qualifies as type since Diard visited Borneo in 1826. This is a female, therefore not the depicted specimen, which is a male.

***AnthracocerosMarchei*** Oustalet, 1885: 108.

= *Anthracocerosmarchei* Oustalet, 1885.

Syntype, RMNH.AVES.22181, adult male, skin. Loc.: Busuanga Island, Calamianes, [Philippines]. Leg.: A. Marche. Ex: MNHN, 1893.

***Bucerosrhinoceros* var. *Borneoensis*** Schlegel & Müller, 1845: 22.

***Bucerosrhinoceroides*** “Temminck” Bonaparte, 1850a: 89 (nomen novum).

= *Bucerosrhinocerosborneoensis* Schlegel & Müller, 1845.

Syntype, RMNH.AVES.22154, adult female, mounted skin. Loc.: Pontianak, Borneo, [Indonesia]. Leg.: P.M. Diard.

Syntype, RMNH.AVES.88650, immature female, mounted skin. Loc.: Pontianak, Borneo, [Indonesia]. Leg.: P.M. Diard.

The type locality was restricted to the “Doeson or Barito River” by Chasen and Kloss (1930: 25). Page 22 of the bird section of the Verhandelingen was published in livraison 11 on 26 June 1845 (Husson and Holthuis 1955: 23). One was sent to the MfN in 1833.

Bonaparte introduced *rhinoceroides* as an unnecessary nomen novum for *Bucerosrhinoceros* var. *Borneoensis* Schlegel & Müller, 1845.

***Bucerosrhinoceros* var. *Sumatrana*** Schlegel & Müller, 1845: 22.

= *Bucerosrhinocerosrhinoceros* Linnaeus, 1758.

Syntype, RMNH.AVES.22152, adult male, mounted skin. Loc.: Batang Singalang, Sumatra, [Indonesia], [v.-viii.1834]. Leg.: S. Müller.

Syntype, RMNH.AVES.22153, adult, sex unknown, mounted skin. Loc.: Batang Singalang, Padang, Sumatra, [Indonesia], [v.-viii.1834]. Leg.: S. Müller.

Page 22 of the bird section of the ‘Verhandelingen’ was published in livraison 11 on 26 June 1845 (Husson and Holthuis 1955: 23).

Müller visited the area of Batang Singgalan between May and August 1834.

***Buceroscristatus*** Vieillot, 1816c: 591.

= *Bucerosrhinocerossilvestris* Vieillot, 1816.

Vieillot (1816c) based this name on “Le calao a casque concave” by Levaillant (1801a: 10, pls 3 and 4). Levaillant based his description on a specimen in the cabinet of Temminck received from Java, which is no longer present in the RMNH.

***Bucerossilvestris*** Vieillot, 1816c: 592.

= *Bucerosrhinocerossilvestris* Vieillot, 1816.

Vieillot (1816c) based this name on “Le calao a casque en croissant’’ by Levaillant (1801a: 26, pl. 13). Levaillant based his description on a specimen in the cabinet of Temminck from Java and mentioned the Moluccas as a probable origin. Java must have been overlooked by Vieillot, who only mentioned the Moluccas as type locality. This specimen is no longer present in the RMNH.

***Bucerosrhinoceros* var. *Indica*** Schlegel & Müller, 1845: 22.

***Bucerossublunatus*** “Temminck” Bonaparte, 1850a: 90 (nomen novum).

= *Bucerosrhinocerossilvestris* Vieillot, 1816.

Holotype, RMNH.AVES.22180, adult female, mounted skin. Loc.: “Seringapatam’, [India]. Ex: G.A. Frank, 1838.

Holotype by monotypy. Schlegel and Müller (1845) only mentioned the specimen from Seringapatam, India, that is considered an error. Sanft (1960) designated Java as the type locality. Page 22 of the bird section of the ‘Verhandelingen’ was published in livraison 11 on 26 June 1845 (Husson and Holthuis 1955: 23).

Bonaparte introduced *sublunatus* as an unnecessary nomen novum for *Bucerosrhinoceros* var. *Indica* Schlegel & Müller, 1840.

***Buceroslunatus*** Temminck, 1834: livr. 92, pl. 546.

***Bucerosrhinoceros* var. *Javanica*** Schlegel & Müller, 1845: 22.

= *Bucerosrhinocerossilvestris* Vieillot, 1816.

Syntype, RMNH.AVES.198090, adult, sex unknown, mounted skin. Loc.: Java, [Indonesia], [vi.1826–viii.1827]. Leg.: H. Boie.

Syntype, RMNH.AVES.198092, adult, sex unknown, mounted skin. Loc.: Java, [Indonesia], [vi.1826–viii.1827]. Leg.: H. Boie.

Syntype, RMNH.AVES.158244, adult, sex unknown, skull. Loc.: Java, [Indonesia]. Ex: -, 1828.

Temminck (1834) mentioned that at the time of the publication of his introduction to the genus *Buceros* (Temminck, 1823: livr. 36) this species was only known from a mutilated specimen and a living bird, both in his possession. These specimens are no longer in the RMNH. During the publication of the description of *lunatus* in 1834 the specimens listed above were available to Temminck, therefore listed here as syntypes. Temminck gave as type locality for *lunatus* Java and Banda, Bonaparte (1850a: 90) restricted the type locality to Java.

Boie visited Java between June 1826 and September 1827.

***Bucerosbucinator*** Temminck, 1824: livr. 48, pl. 284.

= *Bycanistesbucinator* (Temminck, 1824).

Temminck (1824) based his description on a skull in his possession (no longer in the RMNH) and a specimen collected by Delalande in the MNHN (MNHN C.G.2007-216), erroneously listed by Voisin and Voisin (2008b: 21) as holotype.

***Buceroscylindricus*** Temminck, 1831: livr. 88, pl. 521, fig. 2.

= *Bycanistescylindricus* (Temminck, 1831).

Lectotype, RMNH.AVES.22174, adult male, skull. Loc.: Goldcoast [= Ghana]. Ex: Brooke’s Museum, 1828.

Temminck (1831) based his description on two skulls bought at the auction of the Brooke’s collection in 1828 (Gijzen 1938: 162): an adult male and an immature (Schlegel 1862a: 18). The whereabouts of the immature skull are unknown.

Van Oort (1907: 176) designated the lectotype.

***Bucerosatratus*** Temminck, 1835: livr. 94, pl. 558.

= *Ceratogymnaatrata* (Temminck, 1835).

Holotype by monotypy. Formerly in the collection of Meiffren-Laugier de Chartrouse, now probably in the MNHN, though not listed in Voisin and Voisin (2008b).

***Buceroselatus*** Temminck, 1831: livr. 88, pl. 521, fig. 1.

= *Ceratogymnaelata* (Temminck, 1831).

Lectotype, RMNH.AVES.22161, adult male, cranium. Loc.: “Goudkust” [= Ghana]. Ex: C.J. Temminck.

Erroneously given as RMNH.AVES.22164 in Van den Hoek Ostende et al. (1997: 193). Schlegel (1862a: 18) designated the lectotype.

Temminck (1831) described this species based on two skulls, one in the RMNH, obtained from Prof. N.C. de Fremery in Utrecht and one he had seen in Brooke’s collection. The whereabouts of the syntype from Brooke’s collection are unknown.

***OrtholophusCassini*** Finsch, 1903b: 196.

= *Horizocerusalbocristatuscassini* (Finsch, 1903).

Syntype, RMNH.AVES.22155, adult male, skin. Loc.: Victoria, Cameroon, 05.xi.1898. Leg.: P. Preuss. Ex: MfN, 1903.

**Berenicornis (Buceros) macrourus** Bonaparte, 1850a: 91.

= *Horizocerusalbocristatusmacrourus* (Bonaparte, 1850).

Syntype, RMNH.AVES.22156, adult male, mounted skin. Loc.: Rio Boutry, Ashantee, Goldcoast [= Ghana]. Leg.: H.S. Pel, 1842.

Syntype, RMNH.AVES.22157, adult male, mounted skin. Loc.: Goldcoast [= Ghana]. Leg.: H.S. Pel.

Syntype, RMNH.AVES.22158, adult female, mounted skin. Loc.: Dabocrom, Goldcoast [= Ghana]. Leg.: H.S. Pel.

***BucerosNagtglasii*** Schlegel, 1862a: 16.

= *Horizocerushartlaubihartlaubi* (Gould, 1861).

Holotype, RMNH.AVES.22178, adult, sex unknown, skin. Loc.: St. George d’Elmina, Goudkust [= Ghana], 1861. Leg.: C.J.M. Nagtglas.

Holotype by monotypy as Schlegel (1862a) referred to a single specimen.

Van den Hoek Ostende et al. (1997: 195) dated this name from Schlegel (1863a). However, the description was published in another work which came out a few months earlier in 1862 (see Pieters and Dickinson 2005; Dickinson and Pieters 2011).

***Lophocerosalboterminatus*** Büttikofer, 1889b: 67.

= *Lophocerosalboterminatus* Büttikofer, 1889.

Syntype, RMNH.AVES.22173, adult female, skin. Loc.: Gambos, Angola, 08.ii.1888. Leg.: P.J. van der Kellen.

Syntype, RMNH.AVES.22172, adult female, skin. Loc.: Gambos, Angola, 11.ii.1888. Leg.: P.J. van der Kellen.

Syntype, RMNH.AVES.22171, adult male, skin. Loc.: Gambos, Upper Cunene, Angola, 20.ii.1888. Leg.: P.J. van der Kellen.

***Bucerospulchrirostris*** Schlegel, 1862a: 15.

= *Lophoceroscamurus* (Cassin, 1857).

Syntype, RMNH.AVES.22168, adult, sex unknown, skin. Loc.: St. George d’Elmina, Goldcoast [= Ghana], 1861. Leg.: C.J.M. Nagtglas.

Syntype, RMNH.AVES.22169, adult, sex unknown, skin. Loc.: St. George d’Elmina, Goldcoast [= Ghana], 1861. Leg.: C.J.M. Nagtglas.

Syntype, RMNH.AVES.22170, adult, sex unknown, skin. Loc.: St. George d’Elmina, Goldcoast [= Ghana], 1861. Leg.: C.J.M. Nagtglas.

Van den Hoek Ostende et al. (1997: 195) dated this name from Schlegel (1863b). However, the description was published in another work which was published a few months earlier in 1862 (see Pieters and Dickinson 2005; Dickinson and Pieters 2011).

***Bucerosfasciatus*** Shaw, 1812: 34.

= *Lophocerosfasciatusfasciatus* (Shaw, 1812).

Lectotype, RMNH.AVES.22179, adult male, relaxed mount. Loc.: Angola. Ex: Cabinet Temminck.

Shaw (1812) based his name on Levaillant’s description of the “Calao Longibandes” (1806–1807: pl. 233) which Levaillant based on three specimens of which one was send to Temminck (Temminck, 1807: 38, cat. no. 778). By listing one of these syntypes as holotype, Van den Hoek Ostende et al. (1997: 194) unintentionally designated RMNH.AVES.22179 as lectotype.

***Bucerossemifasciatus*** “Temminck” Hartlaub, 1855: 356.

= *Lophocerosfasciatussemifasciatus* (Hartlaub, 1855).

Syntype, RMNH.AVES.22288, adult male, mounted skin. Loc.: Rio Boutry, Côte d’Or [= Ghana]. Leg.: H.S. Pel

Syntype, RMNH.AVES.197313, adult male, mounted skin. Loc.: Senegal. Ex: MNHN.

Syntype, RMNH.AVES.197315, adult female, mounted skin. Loc.: Rio Boutry, Côte d’Or [= Ghana], viii.1842. Leg.: H.S. Pel.

Syntype, RMNH.AVES.197319, immature male, mounted skin. Loc.: Rio Boutry, Côte d’Or [= Ghana], xii.1842. Leg.: H.S. Pel.

Syntype, RMNH.AVES.157136, adult, sex unknown, skull. Loc.: Côte d’Or [= Ghana]. Leg.: H.S. Pel.

Syntype, RMNH.AVES.157142, adult, sex unknown, skull. Loc.: Côte d’Or [= Ghana]. Leg.: H.S. Pel.

Syntype, RMNH.AVES.254333, adult female, skeleton. Loc.: Côte d’Or [= Ghana]. Leg.: H.S. Pel.

Although the article by Hartlaub (1855) deals with the specimens collected by Pel, Hartlaub specifically mentioned the specimen from Senegal in his description of *semifasciatus*. Van den Hoek Ostende et al. (1997: 194) only listed RMNH.AVES.22288 as syntype.

***Buceroslimbatus*** Rüppell, 1835: 5, pl. 2 fig. 1.

= *Lophoceroshemprichii* (Ehrenberg, 1833).

Paralectotype, RMNH.AVES.197402.

Steinbacher (1949: 108) designated the lectotype in the SMF (SMF 12623). For more information see Steinheimer (2005a: 239).

According to Rüppell (1835–1840) he collected this species in Temben province in June (1832).

***Bucerosepirhinus*** Sundevall, 1850: 108.

**Bucerosnasutusvar.caffer** Sundevall, 1850: 108.

= *Lophocerosnasutusepirhinus* (Sundevall, 1850).

Syntype, RMNH.AVES.197367, adult male, mounted skin. Loc.: Makkalis [= Magaliesberg], South Africa. Leg.: J.A. Wahlberg, 26.xi.1841.

Sundevall (1850) did not intend to publish var. caffer as a new name, so even though it is mentioned earlier in the text, *epirhinus* is the available name. Two other syntypes are in the NRM (NRM 569870 and 569871).

***Buceroscineraceus*** Temminck, 1832: livr. 89, no plate.

= *Ocycerosgriseus* (Latham, 1790).

Temminck (1832) did not give an indication where he had seen the specimen(s) on which he based his description.

***Penelopidestalisi*** Finsch, 1903a: 190.

= *Penelopidesmanillaemanillae* (Boddaert, 1783).

Syntype, RMNH.AVES.22175, adult male, skin. Loc.: Prov. Cagayan, N. Luzon, Philippines, 23.ii.1892. Leg.: A. van der Valk, 1893.

***Buceroscorrugatus*** Temminck, 1832: livr. 90, pl. 531.

= *Rhabdotorrhinuscorrugatuscorrugatus* (Temminck, 1832).

Syntype, RMNH.AVES.22166, adult male, mounted skin. Loc.: Pontianak, Borneo, [Indonesia], [1826]. Leg.: P.M. Diard.

Temminck (1832) wrote having received three specimens: an adult male and an immature from Borneo collected by Diard and a male from the Indian continent. The latter is either a different species or the locality is erroneous. Schlegel (1862a: 9–10) did not list specimens other than RMNH.AVES.22166 that fit as syntype.

***Bucerosgracilis*** Temminck, 1832: livr. 90, pl. 535.

= *Rhabdotorrhinuscorrugatuscorrugatus* (Temminck, 1832).

Holotype by monotypy. According to Temminck (1832) he received a single specimen from Borneo. Schlegel (1862a: 9) listed a specimen collected by Schwaner (RMNH.AVES.197627) as the depicted specimen on pl. 535. Schwaner visited Borneo after the publication so either the collector is incorrect, or Schlegel is in error.

***Bucerosexarhatus*** “Reinwardt” Temminck, 1823: livr. 36, pl. 211.

= *Rhabdotorhinusexarhatusexarhatus* (Temminck, 1823).

Possible holotype, RMNH.AVES.22165, adult female, cranium. Loc.: Celebes [= Sulawesi], [Indonesia], [ix–xii.1821]. Leg.: C.G.C. Reinwardt.

Holotype by monotypy. Temminck (1823) based his description on a single specimen collected by Reinwardt. Judging from Temminck’s illustration this bird was a female. There is no longer a mounted skin collected by Reinwardt in the RMNH which fits the illustration. However, there is a skull of a female collected by Reinwardt which, judging from the damage to the back of the skull, might be the leftover from a specimen that was previously mounted (but disintegrated). The details of the casque and bill agree very well with the specimen illustrated on pl. 211. We therefore assume that this is the skull which belonged to the skin Temminck illustrated.

Reinwardt visited North Sulawesi between September and December 1821.

***Bucerosleucocephalus*** Vieillot, 1816c: 592.

***Bucerossulcatus*** Temminck, 1821: livr. 12, pl. 69.

= *Rhabdotorrhinusleucocephalus* (Vieillot, 1816).

Holotype for *leucocephalus*, RMNH.AVES.24056, adult male, mounted skin. Loc.: Mindanao, Philippines. Leg.: -.

Vieillot (1816c) based his description on a single specimen in Temminck’s collection (see Temminck 1807: 37).

Following Dickinson et al. (2022) (see also Introduction of this catalogue under “What is new in this 2023 version”: 14) the specimen illustrated on pl. 69 is the holotype since the text that appeared later is irrelevant regarding type status. Any listing of syntypes for this name in Van den Hoek Ostende et al. (1997: 190) or any other type catalogue is erroneous. RMNH.AVES.24056 is the holotype for *leucocephalus* Vieillot, but not for *sulcatus* Temminck. According to Temminck (1821), the illustrated specimen is in the MNHN. Based on Van den Hoek Ostende et al. (1997 190), who erroneously listed RMNH.AVES.24056 as holotype for *sulcatus* Temminck that was in fact a lectotype selection, Voisin and Voisin (2008b: 21) listed MNHN C.G.2007-217 as a paralectotype. They assumed it to be the specimen on pl. 69 in which they are correct, and MNHN C.G.2007-217 therefore is the holotype.

***Buceroscassidix*** Temminck, 1823: livr. 36, pl. 210.

= *Rhyticeroscassidix* (Temminck, 1823).

Syntype, RMNH.AVES.22167, adult male, mounted skin. Loc.: Tondano, Celebes [= Sulawesi], [Indonesia]. Leg.: C.G.C. Reinwardt [20–24.x.1821].

According to the description the RMNH received two specimens from Reinwardt. The whereabouts of the second specimen are unknown. Schlegel (1862a: 9) did not list it in his catalogue.

Reinwardt visited the Tondano area between 20 and 24 September 1821.

***Aceroscassidixbrevirostris*** Van Bemmel, 1951: 56, in: Van Bemmel and Voous 1951.

= *Rhyticeroscassidix* (Temminck, 1823).

Holotype, ZMA.AVES.8701, adult male, skin. Loc.: Labasa, [Muna Island], [Indonesia], 6.x.1948. Leg.: G.A.L. de Haan (625).

Paratypes, ZMA.AVES.9275–9277.

There might be other paratypes in the MZB.

***Buceroscrispatus*** Wilkes, 1808: 479.

***Bucerosundulatus*** Shaw, 1812: 26.

= *Rhyticerosundulatus* (Shaw, 1812).

Wilkes (1808) and Shaw (1812) based their name on “Le Calao à casque festonné” by Levaillant (1801a: 41, pls 20 and 21). Levaillant saw this new species in the cabinet of Temminck (see Temminck 1807: 37). It is no longer present in the RMNH.

***BucerosErythrorhynchus*** “Brisson” Kuhl, 1820: 522 (not Temminck, 1823. livr. 36, sp. 19 (text).

= *Tockuserythrorhynchus* (Temminck, 1823).

Kuhl (1820, Buffoni et Daubentoni: 5, 22) published *Buceros Erythrorhynchus* as a scientific name for “Le Calao a bec rouge de Sénégal’’ described and depicted in Brisson (1760:, t. 4: 575, pl. 46, fig. 2) and depicted on pl. 260 in Daubenton (Planches enluminées). Although Kuhl did not give a proper description (his work was intended to be an index to the plates by Buffon and Daubenton), by referring to the plate this is an available name. Therefore, *Tockuserythrorhynchus* should be attributed to Kuhl, 1820 and not to Temminck, 1823.

The specimen Brisson had seen was in the collection of R.-A. de Reaumur and was collected by M. Adanson in Senegal.

***Bucerosrufirostris*** Sundevall, 1850: 108.

**Buceroserythrorhynchosvar.caffer** Sundevall, 1850: 108.

= *Tockusrufirostris* (Sundevall, 1850).

Paralectotype, RMNH.AVES.197484.

Sundevall (1850) did not intend to publish var. caffer as a new name, so even though it is mentioned earlier in the text, *rufirostris* is the available name. Gyldenstolpe (1926: 107) selected the lectotype by listing an adult male (NRM 569872) in the NRM as type.

### ﻿CORACIIFORMES

#### ﻿Coraciidae

***Coraciascyanogaster*** Cuvier, 1816: 401.

= *Coraciascyanogaster* Cuvier, 1816.

Possible holotype, RMNH.AVES.88625, adult, sex unknown, relaxed mount. Loc.: “Senegambie”. Ex: Cabinet Temminck.

Cuvier (1816) based his name on the description of “Le Rollier à ventre bleu” by Levaillant (1806: 78, pl. 26). Levaillant based his description on a specimen he received from Temminck. Van den Hoek Ostende et al. (1997: 188) listed this specimen as holotype which we follow here, although the provenance of RMNH.AVES.88625 is uncertain.

***GarrulusTemminckii*** Vieillot, 1819d: 435.

= *Coraciastemminckii* (Vieillot, 1819).

Holotype, RMNH.AVES.88626, adult male, relaxed mount. Loc.: “Otaheite” [error for Sulawesi], [Indonesia]. Ex: Cabinet Temminck.

According to Schlegel (1867b: 138) this specimen was collected by Reinwardt [in 1821]. If correct, RMNH.AVES.88626 cannot be the holotype. Vieillot refers to pl. 9 in Levaillant (1807: 46, pl. 9). In 1823 a bird was sent to the NMW (1823.LXXXVIII.22).

***CoraciasViolacea*** Temminck, 1807: 45.

= *Eurystomusglaucurusafer* (Latham, 1790).

Syntype, RMNH.AVES.88627, adult, sex unknown, relaxed mount. Loc.: “Senegambie”. Ex: Cabinet Temminck.

***Eurystomusorientalisoberholseri*** Junge, 1936: 30.

= *Eurystomusorientalisoberholseri* Junge, 1936.

Holotype, RMNH.AVES.88628 (6651), adult male, skin. Loc.: Sinabang, Simalur, [Indonesia], 11.vii.1913. Leg.: E.R. Jacobson and W.C. van Heurn.

Paratypes, RMNH.AVES.88629–88642.

#### ﻿Alcedinidae

***Daceloconcreta*** Temminck, 1825: livr. 58, pl. 346.

= *Actenoidesconcretusconcretus* (Temminck, 1825).

Holotype, RMNH.AVES.88523, adult male, relaxed mount. Loc.: Sumatra, [Indonesia]. Leg.: -.

Holotype by monotypy. Temminck’s description (1825) was based on the only specimen known. According to Schlegel (1863f: 26) this specimen was illustrated on pl. 346 and collected by Müller, which must be an error as Müller did not visit Sumatra until 1833. One specimen from Borneo was donated to the NMW in February 1833 (1833.VII.19) and one in 1841. One arrived in the MfN in 1824.

***Dacelomonachus*** Bonaparte, 1850a: 154.

***Dacelocyanocephala*** “Forsten” Bonaparte, 1850a: 154.

= *Actenoidesmonachusmonachus* (Bonaparte, 1850).

Syntype, RMNH.AVES.88543, adult male, mounted skin. Loc.: Kota-Boema, Kema, Celebes [= Sulawesi], [Indonesia]. Leg.: E.A. Forsten.

Syntype, RMNH.AVES.88544, adult male, mounted skin. Loc.: Kema, Celebes [= Sulawesi], [Indonesia]. Leg.: E.A. Forsten.

Syntype, RMNH.AVES.88545, adult female, mounted skin. Loc.: Kota-Boema, Celebes [= Sulawesi], [Indonesia]. Leg.: E.A. Forsten.

Bonaparte (1850a) published *cyanocephala* and *princeps* (see below), both manuscript names by Forsten, in the synonymy of *monachus*.

Forsten visited North Sulawesi from 22 March to 19 June 1841.

***Daceloprinceps*** “Forsten” Bonaparte, 1850a: 154.

***Monachalcyonprinceps*** Reichenbach, 1851: iv, 38.

= *Actenoidesprincepsprinceps* (Reichenbach, 1851).

Syntype, RMNH.AVES.204978, adult male, mounted skin. Loc.: Menado, Celebes [= Sulawesi], [Indonesia], [22.iii–19.vi.1841]. Leg.: E.A. Forsten.

Syntype, RMNH.AVES.204980, adult female, mounted skin. Loc.: Menado, Celebes [= Sulawesi], [Indonesia]. Leg.: E.A. Forsten.

Reichenbach (1851) based his *princeps* on the description in Bonaparte (1850a: 154) and Forsten’s manuscript name published there.

Forsten visited North Sulawesi from 22 March to 19 June 1841.

***Alcedojaponica*** Bonaparte, 1854a: 320.

= *Alcedoatthisbengalensis* Gmelin, 1788.

Syntype, RMNH.AVES.88488, adult male, mounted skin. Loc.: Japan. Leg.: Ph.F.B. von Siebold.

Syntype, RMNH.AVES.88489, adult male, mounted skin. Loc.: Japan. Leg.: Ph.F.B. von Siebold.

Syntype, RMNH.AVES.88490, immature male, mounted skin. Loc.: Japan. Leg.: Ph.F.B. von Siebold.

Syntype, RMNH.AVES.88491, adult female, mounted skin. Loc.: Japan. Leg.: Ph.F.B. von Siebold.

Syntype, RMNH.AVES.88492, adult, sex unknown, mounted skin. Loc.: Japan. Leg.: Ph.F.B. von Siebold.

Syntype, RMNH.AVES.88493, adult, sex unknown, mounted skin. Loc.: Japan. Leg.: Ph.F.B. von Siebold.

Syntype, RMNH.AVES.88494, adult, sex unknown, mounted skin. Loc.: Japan. Leg.: Ph.F.B. von Siebold.

Syntype, RMNH.AVES.88495, adult, sex unknown, mounted skin. Loc.: Japan. Leg.: Ph.F.B. von Siebold.

RMNH.AVES.8488 is labelled “male”, but according to the stand as “female”. For a discussion on problems and recognition of the name *Alcedojaponica*, see Morioka et al. (2005).

***Alcedoberyllina*** Vieillot, 1818a: 414.

= *Alcedocoerulescens* Vieillot, 1818.

Possible syntype, RMNH.AVES.88496, adult male, relaxed mount. Loc.: Java, [Indonesia]. Ex: Cabinet Temminck.

According to Voisin and Voisin (2008b: 9), the MNHN holds possible holotypes (by monotypy) for *beryllina* (MNHN C.G.2006-559) and for *coerulescens* (MNHN C.G.2006-560), see also Jansen (2018: 388). However, as Vieillot (1818a) based many of his descriptions on Temminck (1807), see introduction, RMNH.AVES.88496 is listed here as (possible) syntype for *beryllina*. RMNH.AVES.88497, listed in Van den Hoek Ostende et al. (1997: 178), is no longer considered to be a type due to lack of conclusive information.

***Alcedocryzona* [sic**] Temminck, 1830: livr. 86, text with pl. 508.

= *Alcedoeuryzonaeuryzona* Temminck, 1830.

Lectotype, RMNH.AVES.88498, adult male, mounted skin. Loc.: Jasinga, Java, [Indonesia], xi.1827. Leg.: S. Müller.

Not illustrated; the description was published in the text of *A.lazuli* Temminck (1830) who gave no indication of the number of specimens, so the listing by Van den Hoek Ostende et al. (1997: 178) as holotype is an error but constituted a lectotype designation.

***Alcedoquadribrachys*** “Temminck” Bonaparte, 1850a: 158.

= *Alcedoquadribrachysquadribrachys* Bonaparte, 1850.

Lectotype, RMNH.AVES.88499, adult male, relaxed mount. Loc.: Rio Boutry [= Butre], Goldcoast [= Ghana]. Leg.: H.S. Pel, 1841.

Schlegel (1863f: 10) designated the lectotype.

***Halcyonfulgidus*** Gould, 1857: 65.

= *Caridonaxfulgidus* (Gould, 1857).

Paralectotype, RMNH.AVES.88539.

Gould (1857) based his description on specimens he received from A.R. Wallace. The label gives “Wallace 1866” as collector: the date, not the collector, is an error as the specimen was collected in 1856 and received in 1857 through the dealer Frank with whom Gould was in contact about this new kingfisher.

Warren (1966: 104) erroneously listed a specimen in the NHM (NHMUK 1881.5.1.2952) as holotype, thereby designating it the lectotype.

Wallace stayed in Lombok from 17 June to 30 August 1856 and his sales catalogue listed *Halcyonfulgidus*, collected at Ampanam and Gunong Sari (Baker 2001: 267).

***Cerylerudissyriaca*** Roselaar, 1995: 22.

= *Cerylerudissyriacus* Roselaar, 1995.

Holotype, RMNH.AVES.88859, adult female, mounted skin. Loc.: Syria. Ex: Maison Verreaux, 1863.

Paratype, RMNH.AVES.88860.

Other paratypes are in the ZFMK, ZMM, and NHM.

***Alcyonediemenensis*** Gould, 1846: 19.

= *Ceyxazureusdiemenensis* (Gould, 1846).

Possible paralectotype, RMNH.AVES.202146.

Stone (1913: 151) designated the lectotype in the ANSP (ANSP 21232). Fisher and Calaby (2009: 138) listed this specimen as a possible type.

Gould travelled through Australia between September 1838 and May 1840, visiting Tasmania several times.

***Alcyonepulchra*** Gould, 1846: 19.

= *Ceyxazureusruficollaris* (Bankier, 1841).

Possible paralectotype, RMNH.AVES.202150.

Stone (1913: 151) designated the lectotype in the ANSP (ANSP 21237). Fisher and Calaby (2009: 138) listed this specimen as a possible type.

***Ceyxinnominata*** Salvadori, 1869: 446.

= *Ceyxerithaca* (Linnaeus, 1758).

Syntype, RMNH.AVES.202173, adult male, relaxed mount. Loc.: Sumatra, [Indonesia], [vii.1833–1835]. Leg.: S. Müller (= *Ceyxerithacaerithaca* (Linnaeus, 1758).

Syntype, RMNH.AVES.202219, adult male, relaxed mount. Loc.: Sumbawa, [Indonesia], 1842. Leg.: E.A. Forsten (= *Ceyxerithacamotleyi* Kloss, 1929).

Syntype, RMNH.AVES.254518, sex unknown, skeleton. Loc.: Java, [Indonesia], [xii.1820–ix.1821]. Leg.: Kuhl and van Hasselt (= *Ceyxerithacamotleyi* Kloss, 1929).

Syntype, RMNH.AVES.255870, sex unknown, skeleton. Loc.: Java, [Indonesia], [xii.1820–ix.1821]. Leg.: Kuhl and van Hasselt (= *Ceyxerithacamotleyi* Kloss, 1929).

Syntype, RMNH.AVES.256609, sex unknown, skeleton. Loc.: Java, [Indonesia], [xii.1820–ix.1821]. Leg.: Kuhl and van Hasselt (= *Ceyxerithacamotleyi* Kloss, 1929).

Salvadori (1869) referred to specimens in the RMNH from Sumatra and Bangka (= *Ceyxerithacaerithaca* (Linnaeus, 1758) and specimens collected by Boie and Kuhl and van Hasselt on Java, and Forsten on Sumbawa (= *Ceyxerithacamotleyi* Kloss, 1929), listed in Schlegel (1863f: 49). Other syntypes are in the MZUT (MZUT Av15267; Aimassi et al. 2020: 89) and other collections.

Kuhl and van Hasselt collected together on Java between December 1820 and September 1821. Müller visited West Sumatra from July 1833 until late 1835.

***Dacelofallax*** Schlegel, 1866b: 187.

= *Ceyxfallaxfallax* (Schlegel, 1866).

Syntype, RMNH.AVES.88501, adult male, mounted skin. Loc.: Bone [= Gorontalo], Celebes [= Sulawesi], [Indonesia], 24.xi.1863. Leg.: C.B.H. von Rosenberg.

Syntype, RMNH.AVES.88502, adult male, mounted skin. Loc.: Bone [= Gorontalo], Celebes [= Sulawesi], [Indonesia], 25.xi.1863. Leg.: C.B.H. von Rosenberg.

Syntype, RMNH.AVES.88503, adult female, mounted skin. Loc.: Gorontalo, Celebes [= Sulawesi], [Indonesia], 07.iii.1864. Leg.: C.B.H. von Rosenberg.

Syntype, RMNH.AVES.88504, adult female, mounted skin. Loc.: Toula-bello [= Tulabolo], Celebes [= Sulawesi], [Indonesia], 26.iv.1864. Leg.: C.B.H. von Rosenberg.

Syntype, RMNH.AVES.88505, immature female, mounted skin. Loc.: Toula-bello [= Tulabolo], Celebes [= Sulawesi], [Indonesia], 04.iv.1864. Leg.: C.B.H. von Rosenberg.

Syntype, RMNH.AVES.88506, immature male, mounted skin. Loc.: Toula-bello [= Tulabolo], Celebes [= Sulawesi], [Indonesia], 06.v.1864. Leg.: C.B.H. von Rosenberg.

Syntype, RMNH.AVES.88507, adult female, mounted skin. Loc.: Toula-bello [= Tulabolo], Celebes [= Sulawesi], [Indonesia], 10.v.1864. Leg.: C.B.H. von Rosenberg.

Syntype, RMNH.AVES.88508, adult male, mounted skin. Loc.: Menado, Celebes [= Sulawesi], [Indonesia], 1865. Ex: L.D.H. Renesse van Duivenbode.

In some cases, the date of collection on the stand differs from that on the label. According to the data on the stands, and according to Schlegel (1863f: 33) RMNH.AVES.88504 was collected on 26 April 1864 and RMNH.AVES.88505 and 88506 on 29 April 1864 and 7 May 1864 respectively.

Rosenberg stayed in the area of Tulabolo between 14 April and 21 May 1864 (see Von Rosenberg 1865: 106–117).

***Ceyxlepida*** Temminck, 1836: livr. 100, pl. 595, fig. 1.

= *Ceyxlepiduslepidus* Temminck, 1836.

Syntype, RMNH.AVES.88509, adult, sex unknown, mounted skin. Loc.: Amboina [= Ambon], [Indonesia], [29.iii–20.iv.1828]. Leg.: S. Müller.

Müller visited Ambon between 29 March and 20 April 1828.

***Ceyxpusilla*** Temminck, 1836: livr. 100, pl. 595, fig. 3.

= *Ceyxpusilluspusillus* Temminck, 1836.

Lectotype, RMNH.AVES.88511, adult male, mounted skin. Loc.: [Lobo Bay], “Nouveau Guinee” [= Papua], [Indonesia], [4.vii–29.viii], 1828. Leg.: S. Müller.

Temminck (1836) gave no indication of the number of specimens available to him. Schlegel (1863f: 18) designated the lectotype, which must have been overlooked by Van den Hoek Ostende et al. (1997: 179), who listed this specimen as holotype.

According to Temminck this specimen was collected “dans les mêmes localités que la précédente” i.e., *Ceyxsolitaria*.

***Ceyxsolitaria*** Temminck, 1836: livr. 100, pl. 595, fig. 2.

= *Ceyxsolitarius* Temminck, 1836.

Lectotype, RMNH.AVES.88510, adult male, mounted skin. Loc.: Lobo Bay, [Papua], [Indonesia] [4.vii–29.viii.1828]. Leg.: S. Müller.

Temminck (1836) gave no indication of the number of specimens available to him. Schlegel (1874b: 9) designated the lectotype, which must have been overlooked by Van den Hoek Ostende et al. (1997: 179), who listed this specimen as a holotype.

***Dacelocyanotis*** Temminck, 1824: livr. 44, pl. 262.

= *Citturacyanotiscyanotis* (Temminck, 1824).

Lectotype, RMNH.AVES.88512, adult, sex unknown, mounted skin. Loc.: Celebes [= Sulawesi], [Indonesia]. Leg.: -.

Temminck (1824) gave no indication of the number of specimens available to him. Schlegel (1863f: 23) designated the lectotype, which must have been overlooked by Van den Hoek Ostende et al. (1997: 180), who listed this specimen as a holotype.

The type locality in the original description is erroneously given as Sumatra, as this species is endemic to Sulawesi.

***Clytoceyxreximperator*** van Oort, 1909a: 79.

= *Clytoceyxrex* Sharpe, 1880.

Lectotype, RMNH.AVES.88513, adult male, skin. Loc.: Alkmaar [Papua], [Indonesia], 17.ix.1907. Leg.: Dutch New Guinea Expedition 1907 (449).

Van Oort (1909a) did not nominate any type specimens, but listed only one specimen in the RMNH, treated by Van den Hoek Ostende et al. (1997: 180) as holotype by monotypy. However, Van Oort also referred to two specimens in the Rothschild collection in Tring (a male and a female, bought from Boucard; now in AMNH or NHM). Warren (1966) and Greenway (1978) did, however, not list any type(s) in the NHM or AMNH.

The listing as holotype by Van den Hoek Ostende et al. (1997: 180) constitutes a lectotype designation, making the other two specimens paralectotypes.

***DaceloGaudichaud*** Gaimard, 1823: 52.

= *Dacelogaudichaud* Gaimard, 1823.

Syntype, RMNH.AVES.88514, adult male, mounted skin. Loc.: Waigeu, [Papua], [Indonesia], [16.xii.1818–5.i.1819]. Leg.: Voyage de l’Uranie.

Syntype, RMNH.AVES.88515, adult female, mounted skin. Loc.: Waigeu, [Papua], [Indonesia], [16.xii.1818–5.i.1819]. Leg.: Voyage de l’Uranie.

Original description was by Gaimard (1823), not Quoy and Gaimard (1824) as frequently referred to, including by Van den Hoek Ostende et al. (1997: 180). In contrast to Quoy and Gaimard (1824) who referred to three specimens (two males and one female), Gaimard (1823) neither mentioned the number of specimens nor the sex. Therefore, we also include RMNH.AVES.88515 in the type series. Voisin and Voisin (2008b: 4) listed two syntypes in the MNHN (MNHN C.G.2006-536, male, and C.G.2006-537, female) and, based on Quoy and Gaimard, excluded RMNH.AVES.88515 from the type series.

The ‘Uranie’ visited Waigeo between 16 December 1818 and 5 January 1819.

***Dacelopygmaeus*** Cretzschmar, 1829: 42, pl. 28 b.

= *Halcyonchelicutichelicuti* (Stanley, 1814).

Paralectotypes, RMNH.AVES.90906, 90909.

The lectotype was designated by Steinbacher (1949: 107) and is in SMF (SMF 12728). Paralectotypes are in SMF, NMH (Steinheimer 2005a: 244) and NMW (Schifter et al. 2007: 243–244).

***Halcyonrufigularis*** Sharpe, 1892: 215.

= *Halcyoncinnamomina* subsp.

Van den Hoek Ostende et al. (1997: 181) incorrectly listed RMNH.AVES.88522 as syntype, as Sharpe (1892) listed a single type at the Zoological Society, now in the NHM (NHMUK 1855.12.19.239; Warren 1966: 253).

**Alcedo (Halcyon) coromanda major** Temminck & Schlegel, 1848: 75, pl. 39.

***Halcyonschlegeli*** Bonaparte, 1850a: 156.

= *Halcyoncoromandamajor* (Temminck & Schlegel, 1848).

Syntype, RMNH.AVES.88524, adult male, mounted skin. Loc.: Japan. Leg.: H. Bürger.

Syntype, RMNH.AVES.88525, immature, sex unknown, mounted skin. Loc.: Japan. Leg.: H. Bürger.

The word “Janasemi” under the stand of both specimens does not refer to a locality (as presumed in the first edition of this catalogue) but to the Japanese name for the species “Yamasemi”.

Bonaparte (1850a) introduced *schlegeli* as an unnecessary nomen novum for *Alcedocoromandamajor* Temminck & Schlegel, 1848.

**Alcedo (Halcyon) coromanda minor** Temminck & Schlegel, 1848: 76.

***Halcyonlilacina*** Bonaparte, 1850a: 156 (nomen novum).

= *Halcyoncoromandaminor* (Temminck & Schlegel, 1848).

Lectotype for *minor*, syntype for *lilacina*, RMNH.AVES.88526, adult male, mounted skin. Loc.: Pontianak, Borneo, [Indonesia]. Leg.: P.M. Diard.

Paralectotype for *minor*, syntype for *lilacina*, RMNH.AVES.88528, adult male, mounted skin. Loc.: Padang, Sumatra, [Indonesia], [vi.1833–1835]. Leg.: S. Müller.

The lectotype for *minor* was selected by Oberholser (1915: 649), who restricted the type locality to Pontianak. Diard travelled to Pontianak in 1826.

RMNH.AVES.88527, listed by Van den Hoek Ostende et al. (1997: 182) as paralectotype, is not part of the type series, as Crookewit arrived in Borneo 1851.

Bonaparte (1850a) introduced *lilacina* as an unnecessary nomen novum for *Alcedocoromandaminor* Temminck & Schlegel, 1848.

Müller visited West Sumatra from June 1833 until late1835.

***Halcyoncoromandasulana*** Mees, 1970: 299.

= *Halcyoncoromandasulana* Mees, 1970.

Holotype, RMNH.AVES.88529, adult female, mounted skin. Loc.: Soela-Bessi [= Sanana], [Indonesia], 17.xi.1864. Leg.: H. Bernstein.

Paratypes, RMNH.AVES.88530–88535.

According to the label, RMNH.AVES.88529 was collected by Bernstein (see also Schlegel 1863f: 17), but on the stand Hoedt is given as collector.

***Alcedoomnicolor*** Temminck, 1822: livr. 23, pl. 135.

= *Halcyoncyanoventris* (Vieillot, 1818).

Syntype, RMNH.AVES.88536, adult male, mounted skin. Loc.: Java, [Indonesia]. Leg.: [C.L. Blume].

According to Schlegel (1863f: 28) this specimen was collected by C.L. Blume. According to the original description another syntype is in the MNHN, but not listed in Voisin and Voisin (2008b).

***Alcedocyanoleuca*** Vieillot, 1818a: 401.

= *Halcyonsenegalensiscyanoleuca* (Vieillot, 1818).

Lectotype, RMNH.AVES.88547, adult, sex unknown, skin. Loc.: Angola. Ex: Cabinet Temminck.

Vieillot (1818a) gave no indication of the number of specimens available to him. The listing of RMNH.AVES.88547 as holotype by Van den Hoek Ostende et al. (1997: 184) constituted a lectotype designation.

***Alcedopicturata*** Schlegel, 1863f: 16.

= *Ispidinapictanatalensis* (Smith, 1832).

Syntype, RMNH.AVES.88549, adult, sex unknown, mounted skin. Loc.: “Cap”, South Africa. Leg.: -.

Syntype, RMNH.AVES.88548, adult, sex unknown, mounted skin. Loc.: Port Natal, South Africa. Leg.: -.

Syntype, RMNH.AVES.89435, adult, sex unknown, mounted skin. Loc.: Port Natal, South Africa. Leg.: -.

***Dacelomelanops*** “Temminck” Bonaparte, 1850a: 154.

= *Lacedopulchellamelanops* (Bonaparte, 1850).

Syntype, RMNH.AVES.88386, immature male, relaxed mount. Loc.: Pontianak, Borneo, [Indonesia], 1826. Leg.: P.M. Diard.

Syntype, RMNH.AVES.88550, adult male, relaxed mount. Loc.: Banjermassing, Borneo, [Indonesia], 1843. Leg.: C.A.L.M. Schwaner.

***Dacelobuccoides*** Temminck, 1836: livr. 99, pl. 586.

= *Lacedopulchellapulchella* (Horsfield, 1821).

Lectotype, RMNH.AVES.88653, adult female, relaxed mount. Loc.: Java, [Indonesia], [xii.1820–ix.1821]. Leg.: [H.Kuhl and J.C. van Hasselt].

Paralectotypes, RMNH.AVES.202630–202633.

Schlegel (1863f: 21) selected the lectotype and attributed this specimen to Kuhl and Van Hasselt. Finsch, however, on a note attached to the specimen, showed doubts about the origin.

Kuhl and Van Hasselt collected on Java from December 1820 until September 1821. Müller visited the Indrapoera area (west Sumatra) in July 1835.

For dating of Temminck’s livraison 99, pl. 586, see Dickinson (2001: 47).

***Alcedolugubris*** Temminck, 1834: livr. 92, pl. 548.

= *Megacerylelugubrislugubris* (Temminck, 1834).

Holotype, RMNH.251781, adult male, mounted skin. Loc.: Japan. Leg.: H. Burger [error for Von Siebold?].

Holotype by monotypy. Temminck (1834) based his description on a unique sample and described and illustrated a specimen with a “belt” formed by black and red spots. Since only males have red (rusty) spots, Temminck must have based his description on a male, although he did not give the sex of the specimen and was probably unaware of the difference.

Van den Hoek Ostende et al. (1997: 178) followed the information under the stand of RMNH.AVES.88500 which was collected by Von Siebold but is a female without any red (rusty) markings. Hence it neither fits the description nor the plate. G.F. Mees had added a note on the label of RMNH.AVES.251781, the male, that this specimen was collected by Burger and therefore could not be the type. We are, however, under the impression that either the stands of the two specimens have been mixed up (which could easily be done and has occurred in other instances, see Introduction) or that the label information has been mixed up as there has been confusion over the sexes on both labels. We also noticed that the names of both collectors have been added later and are not in Temminck’s handwriting, which has added to the confusion. By correcting the holotype to RMNH.AVES.251781, the plate and description agree with the male specimen.

For more information on dating and Siebold’s collection see Holthuis and Sakai (1970) and Dekker et al. (2001).

***Ramphalcyoncapensisinnominata*** van Oort, 1910d: 126.

= *Pelargopsiscapensisinnominata* (van Oort, 1910).

Syntype, RMNH.AVES.88551, adult male, mounted skin. Loc.: Banjermassing, Borneo, [Indonesia]. Leg.: C.A.L.M. Schwaner.

Syntype, RMNH.AVES.88552, adult female, mounted skin. Loc.: Banjermassing, Borneo, [Indonesia]. Leg.: C.A.L.M. Schwaner.

Syntype, RMNH.AVES.88553, adult male, mounted skin. Loc.: Pontianak, Borneo, [Indonesia]. Leg.: P.M. Diard.

Syntype, RMNH.AVES.88554, adult, sex unknown, mounted skin. Loc.: Pontianak, Borneo, [Indonesia]. Leg.: P.M. Diard.

Syntype, RMNH.AVES.88555, adult male, mounted skin. Loc.: Pleyharie, Borneo, [Indonesia]. 03.viii.1866. Leg.: J. Semmelink.

Syntype, RMNH.AVES.88556, immature, mounted skin. Loc.: Pleyharie, Borneo, [Indonesia], 25.iv.1867. Leg.: J. Semmelink.

Syntype, RMNH.AVES.88557, adult, sex unknown, mounted skin. Loc.: Pleyharie, Borneo, [Indonesia], 1867. Leg.: J. Semmelink.

Syntype, RMNH.AVES.88558, adult, sex unknown, mounted skin. Loc.: Pleyharie, Borneo, [Indonesia], 1867. Leg.: J. Semmelink.

Syntype, RMNH.AVES.88559, adult male, skin. Loc.: Poetoes Siban, Upper Kapoeas, Central Borneo, [Indonesia], 19.v.1896. Leg.: A.W. Nieuwenhuis, 1898.

Syntype, RMNH.AVES.88560, adult male, skin. Loc.: Poetoes Siban, Upper Kapoeas, Central Borneo, [Indonesia], 27.v.1896. Leg.: A.W. Nieuwenhuis, 1898.

Syntype, RMNH.AVES.88561, adult, sex unknown, skin. Loc.: Sandakan, N.E. Borneo, [Sabah], [Malaysia]. Leg.: J.C. Prakke, v.1891.

***Alcedomelanorhyncha*** Temminck, 1826: livr. 66, pl. 391.

= *Pelargopsismelanorhynchamelanorhyncha* (Temminck, 1826).

Lectotype, RMNH.AVES.88562, adult, sex unknown, mounted skin. Loc.: Celebes [= Sulawesi], [Indonesia], [ix–xii.1821]. Leg.: C.G.C. Reinwardt.

Schlegel (1863f: 15) designated the lectotype. According to the original description other paralectotypes are in the MNHN but not listed in Voisin and Voisin (2008b). Schifter et al. (2007: 243) listed a paralectotype in the NMW (NMW 8905).

Reinwardt visited north Sulawesi between September and December 1821.

***TanysipteraCarolinae*** “von Rosenberg” Schlegel, 1871a: 13.

= *Tanysipteracarolinae* Schlegel, 1871.

Syntype, RMNH.AVES.88563, adult male, mounted skin. Loc.: Mefor [= Numfor], [Papua], [Indonesia], 21.i.1869. Leg.: C.B.H. von Rosenberg.

Syntype, RMNH.AVES.88564, adult female, mounted skin. Loc.: Mefor [= Numfor], [Papua], [Indonesia], 22.i.1869. Leg.: C.B.H. von Rosenberg.

Syntype, RMNH.AVES.88565, adult female, mounted skin. Loc.: Mefor [= Numfor], [Papua], [Indonesia], 22.i.1869. Leg.: C.B.H. von Rosenberg.

Syntype, Cat no 11, adult female, mounted skin. Loc.: Mefor [= Numfor], [Papua], [Indonesia], 23.i.1869. Leg.: C.B.H. von Rosenberg.

Syntype, RMNH.AVES.88567, adult male, mounted skin. Loc.: Mefor [= Numfor], [Papua], [Indonesia], 24.i.1869. Leg.: C.B.H. von Rosenberg.

Syntype, RMNH.AVES.88568, adult female, mounted skin. Loc.: Mefor [= Numfor], [Papua], [Indonesia], 25.i.1869. Leg.: C.B.H. von Rosenberg.

Syntype, RMNH.AVES.88569, adult female, mounted skin. Loc.: Mefor [= Numfor], [Papua], [Indonesia], 25.i.1869. Leg.: C.B.H. von Rosenberg.

Syntype, RMNH.AVES.88570, adult female, mounted skin. Loc.: Mefor [= Numfor], [Papua], [Indonesia], 26.i.1869. Leg.: C.B.H. von Rosenberg.

Syntype, RMNH.AVES.88571, adult female, mounted skin. Loc.: Mefor [= Numfor], [Papua], [Indonesia], 27.i.1869. Leg.: C.B.H. von Rosenberg.

Syntype, RMNH.AVES.88572, immature female, mounted skin. Loc.: Mefor [= Numfor], [Papua], [Indonesia], 27.i.1869. Leg.: C.B.H. von Rosenberg.

Syntype, RMNH.AVES.88573, adult male, mounted skin. Loc.: Mefor [= Numfor], [Papua], [Indonesia], 27.i.1869. Leg.: C.B.H. von Rosenberg.

Syntype, RMNH.AVES.88574, adult male, mounted skin. Loc.: Mefor [= Numfor], [Papua], [Indonesia], 28.i.1869. Leg.: C.B.H. von Rosenberg.

Syntype, RMNH.AVES.88575, adult male, mounted skin. Loc.: Mefor [= Numfor], [Papua], [Indonesia], 28.i.1869. Leg.: C.B.H. von Rosenberg.

Syntype, RMNH.AVES.88576, adult female, mounted skin. Loc.: Mefor [= Numfor], [Papua], [Indonesia], 28.i.1869. Leg.: C.B.H. von Rosenberg.

Syntype, RMNH.AVES.88577, immature male, mounted skin. Loc.: Mefor [= Numfor], [Papua], [Indonesia], 30.i.1869. Leg.: C.B.H. von Rosenberg.

Syntype, RMNH.AVES.88578, immature male, mounted skin. Loc.: Mefor [= Numfor], [Papua], [Indonesia], 01.ii.1869. Leg.: C.B.H. von Rosenberg.

Syntype, RMNH.AVES.88579, adult male, mounted skin. Loc.: Mefor [= Numfor], [Papua], [Indonesia], 04.ii.1869. Leg.: C.B.H. von Rosenberg.

Syntype, RMNH.AVES.88580, immature female, mounted skin. Loc.: Mefor [= Numfor], [Papua], [Indonesia], 10.ii.1869. Leg.: C.B.H. von Rosenberg.

Syntype, RMNH.AVES.88581, adult male, mounted skin. Loc.: Mefor [= Numfor], [Papua], [Indonesia], 11.ii.1869. Leg.: C.B.H. von Rosenberg.

Syntype, Cat no 6, adult male, mounted skin. Loc.: Mefor [= Numfor], [Papua], [Indonesia], 11.ii.1869. Leg.: C.B.H. von Rosenberg.

Syntype, RMNH.AVES.88583, adult female, mounted skin. Loc.: Mefor [= Numfor], [Papua], [Indonesia], 18.ii.1869. Leg.: C.B.H. von Rosenberg.

Syntype, RMNH.AVES.88584, immature female, mounted skin. Loc.: Mefor [= Numfor], [Papua], [Indonesia], 21.ii.1869. Leg.: C.B.H. von Rosenberg.

Syntype, RMNH.AVES.88585, immature, mounted skin. Loc.: Mefor [= Numfor], [Papua], [Indonesia], 24.ii.1869. Leg.: C.B.H. von Rosenberg.

Syntype, RMNH.AVES.88586, adult female, mounted skin. Loc.: Mefor [= Numfor], [Papua], [Indonesia], 24.ii.1869. Leg.: C.B.H. von Rosenberg.

Syntype, RMNH.AVES.88587, adult male, mounted skin. Loc.: Mefor [= Numfor], [Papua], [Indonesia], 01.iii.1869. Leg.: C.B.H. von Rosenberg.

Syntype, RMNH.AVES.88588, adult male, mounted skin. Loc.: Mefor [= Numfor], [Papua], [Indonesia], 01.iii.1869. Leg.: C.B.H. von Rosenberg.

Cat. No. 6 and cat. No. 11 (Rosenberg no. 119) are registered to be in the RMNH but could not be located. According to Roselaar and Prins (2000: 106) the latter specimen was exchanged with the ZMA, where it subsequently disappeared before 1950. Roselaar and Prins are in error when they placed the origin of this specimen in Cabinet Temminck as the private collection of Temminck was incorporated in the newly formed RMNH in 1820 and no longer existed at the time of description.

RMNH.AVES.88580 was collected on 27 January 1869 according to the label, but the stand gives 10 February 1869 as the collecting date.

***Tanysipteragalateaboanensis*** Mees, 1964b: 125.

= *Tanysipteragalateaboanensis* Mees, 1964.

Holotype, RMNH.AVES.35579, adult male, mounted skin. Loc.: Boano [Indonesia], 1863. Leg.: D.S. Hoedt.

Paratypes, RMNH.AVES.88589 and 88590.

***Tanysipteraobiensis*** Salvadori, 1877b: 302.

= *Tanysipteragalateaobiensis* Salvadori, 1877.

Syntype, RMNH.AVES.88591, adult female, mounted skin. Loc.: Obi Major, [Indonesia], 14.vii.1862. Leg.: H.A. Bernstein.

Syntype, RMNH.AVES.88592, immature male, mounted skin. Loc.: Obi Major, [Indonesia], 14.vii.1862. Leg.: H.A. Bernstein.

Syntype, RMNH.AVES.88593, immature male, mounted skin. Loc.: Obi Major, [Indonesia], 15.vii.1862. Leg.: H.A. Bernstein.

Syntype, RMNH.AVES.88594, adult male, mounted skin. Loc.: Obi Major, [Indonesia], 16.vii.1862. Leg.: H.A. Bernstein.

Syntype, RMNH.AVES.88597, adult male, mounted skin. Loc.: Obi Major, [Indonesia], 20.vii.1862. Leg.: H.A. Bernstein.

Syntype, RMNH.AVES.88595, adult female, mounted skin. Loc.: Obi Major, [Indonesia], 24.vii.1862. Leg.: H.A. Bernstein.

Syntype, RMNH.AVES.88596, adult female, mounted skin. Loc.: Obi Major, [Indonesia], 29.vii.1862. Leg.: H.A. Bernstein.

Syntype, RMNH.AVES.88598, immature female, mounted skin. Loc.: Obi Lattau [= Obilatu], [Indonesia], 07.viii.1862. Leg.: H.A. Bernstein.

Syntype, RMNH.AVES.88599, immature male, mounted skin. Loc.: Obi Lattau [= Obilatu], [Indonesia], 13.viii.1862. Leg.: H.A. Bernstein.

Syntype, RMNH.AVES.88600, adult male, mounted skin. Loc.: Obi Lattau [= Obilatu], [Indonesia], 14.viii.1862. Leg.: H.A. Bernstein.

Syntype, RMNH.AVES.88601, immature female, mounted skin. Loc.: Obi Lattau [= Obilatu], [Indonesia], 19.viii.1862. Leg.: H.A. Bernstein.

Syntype, RMNH.AVES.88602, immature female, mounted skin. Loc.: Obi Lattau [= Obilatu], [Indonesia], 21.viii.1862. Leg.: H.A. Bernstein.

Syntype, RMNH.AVES.88603, immature female, mounted skin. Loc.: Obi Lattau [= Obilatu], [Indonesia], 21.viii.1862. Leg.: H.A. Bernstein.

Syntype, RMNH.AVES.88604, immature female, mounted skin. Loc.: Obi Lattau [= Obilatu], [Indonesia], 23.viii.1862. Leg.: H.A. Bernstein.

Syntype, RMNH.AVES.88605, adult female, mounted skin. Loc.: Obi Lattau [= Obilatu], [Indonesia], 26.viii.1862. Leg.: H.A. Bernstein.

Syntype, RMNH.AVES.88606, immature female, mounted skin. Loc.: Obi Lattau [= Obilatu], [Indonesia], 27.viii.1862. Leg.: H.A. Bernstein.

Another syntype is in the NHM (Günther 1892: 307, cat. a), but not listed in Warren (1966).

***TanysipteraSchlegelii*** “von Rosenberg” Schlegel, 1871a: 12.

= *Tanysipterariedelii* Verreaux, 1866.

Syntype, RMNH.AVES.88607, adult female, mounted skin. Loc.: Soek [= Biak], [Indonesia], 14.iii.1869. Leg.: C.B.H. von Rosenberg.

Syntype, RMNH.AVES.88608, adult female, mounted skin. Loc.: Soek [= Biak], [Indonesia], 14.iii.1869. Leg.: C.B.H. von Rosenberg.

Syntype, RMNH.AVES.88609, immature female, mounted skin. Loc.: Soek [= Biak], [Indonesia], 14.iii.1869. Leg.: C.B.H. von Rosenberg.

Syntype, RMNH.AVES.88610, adult female, mounted skin. Loc.: Soek [= Biak], [Indonesia], 15.iii.1869. Leg.: C.B.H. von Rosenberg.

Syntype, RMNH.AVES.88611, adult male, mounted skin. Loc.: Soek [= Biak], [Indonesia], 18.iii.1869. Leg.: C.B.H. von Rosenberg.

Syntype, RMNH.AVES.88612, adult male, mounted skin. Loc.: Soek [= Biak], [Indonesia], 18.iii.1869. Leg.: C.B.H. von Rosenberg.

Syntype, Cat. no 5, adult male, mounted skin. Loc.: Soek [= Biak], [Indonesia], 18.iii.1869. Leg.: C.B.H. von Rosenberg.

Syntype, RMNH.AVES.88613, adult male, mounted skin. Loc.: Soek [= Biak], [Indonesia], 22.iii.1869. Leg.: C.B.H. von Rosenberg.

Syntype, RMNH.AVES.88614, adult male, mounted skin. Loc.: Soek [= Biak], [Indonesia], 24.iii.1869. Leg.: C.B.H. von Rosenberg.

Syntype, RMNH.AVES.88615, adult female, mounted skin. Loc.: Soek [= Biak], [Indonesia], 24.iii.1869. Leg.: C.B.H. von Rosenberg.

Syntype, RMNH.AVES.88616, immature female, mounted skin. Loc.: Soek [= Biak], [Indonesia], 24.iii.1869. Leg.: C.B.H. von Rosenberg.

Syntype, RMNH.AVES.88617, adult male, mounted skin. Loc.: Soek [= Biak], [Indonesia], 26.iii.1869. Leg.: C.B.H. von Rosenberg.

Syntype, RMNH.AVES.88618, adult male, mounted skin. Loc.: Soek [= Biak], [Indonesia], 28.iii.1869. Leg.: C.B.H. von Rosenberg.

Syntype, RMNH.AVES.88619, adult female, mounted skin. Loc.: Soek [= Biak], [Indonesia], 28.iii.1869. Leg.: C.B.H. von Rosenberg.

Syntype, RMNH.AVES.88620, adult female, mounted skin. Loc.: Soek [= Biak], [Indonesia], iii.1869. Leg.: C.B.H. von Rosenberg.

Cat. no. 5 could not be located.

***Halcyoncoronatus*** Müller, 1843: 175.

= *Todiramphusaustralasiaaustralasia* (Vieillot, 1818).

Syntype, RMNH.AVES.88516, adult male, mounted skin. Loc.: Timor, [Indonesia], [x.1828–xii.1829]. Leg.: S. Müller.

Syntype, RMNH.AVES.88517, adult female, mounted skin. Loc.: Timor, [Indonesia], [x.1828–xii.1829]. Leg.: S. Müller.

Müller visited Timor between October 1828 and December 1829.

***Todiramphusforsteni*** “Temminck” Bonaparte, 1850a: 157.

= *Todiramphuschlorischloris* (Boddaert, 1783).

Lectotype, RMNH.AVES.88518, adult female, mounted skin. Loc.: Gorontalo, Celebes [= Sulawesi], [Indonesia], [ix.1841–iv.1842]. Leg.: E.A. Forsten.

Bonaparte (1850a) gave no indication of the number of specimens. Schlegel (1863f: 37) only listed the above specimen (without mentioning its type status) which Günther (1892: 280) designated as lectotype.

Forsten visited the area near Gorontalo between September 1841 and April 1842.

***Alcedodiops*** Temminck, 1824: livr. 46, pl. 272.

= *Todiramphusdiops* (Temminck, 1824).

Syntype, RMNH.AVES.88537, adult female, mounted skin. Loc.: Ternate, [Indonesia]. Leg.: -.

Although Temminck (1824) did not refer to Ternate as type locality, Schlegel (1863f: 41) referred to RMNH.AVES.88537 as the specimen on pl. 272.

***Todiramphusfunebris*** “Forsten” Bonaparte, 1850a: 157.

= *Todiramphusfunebris* Bonaparte, 1850.

Syntype, RMNH.AVES.88540, adult male, mounted skin. Loc.: Dodinga, Halmahera, [Indonesia]. Leg.: E.A. Forsten.

Syntype, RMNH.AVES.88541, adult male, mounted skin. Loc.: Dodinga, Halmahera, [Indonesia]. Leg.: E.A. Forsten.

Syntype, RMNH.AVES.88542, adult female, mounted skin. Loc.: Dodinga, Halmahera, [Indonesia]. Leg.: E.A. Forsten.

During a forced stay due to illness on Ternate from 19 June 1841 to mid September 1841, Forsten sent his hunters to Halmahera to collect skins.

***Alcedolazuli*** Temminck, 1830: livr. 86, pl. 508.

= *Todiramphuslazuli* (Temminck, 1830).

Syntype, RMNH.AVES.88538, adult, sex unknown, mounted skin. Loc.: Amboina [= Ambon], [Indonesia]. Leg.: -.

Temminck (1830) referred to two specimens from Sumatra in the RMNH where this south Moluccan species does not, however, occur. The syntype listed above refers to the illustrated specimen on pl. 508, both written under the stand as well as in Schlegel (1863f: 42). We notice differences between the illustrated specimen and RMNH.AVES.88538, as the plate lacks the white spot under the eye evident in the specimen.

***Dacelograyi*** Schlegel, 1863f: 37.

= *Todiramphussacererromangae* (Mayr, 1938).

Syntype, RMNH.AVES.88519, adult female, skin. Loc.: Aneiteum [= Anatom Isl.], New Hebrides [= Vanuatu], 15.xi.1853. Leg.: “Voyage Herald” [B. Seeman].

Syntype, RMNH.AVES.88520, adult, sex unknown, skin. Loc.: Aneiteum [= Anatom Isl.], New Hebrides [= Vanuatu], 15.xi.1853. Leg.: “Voyage Herald” [B. Seeman].

Syntype, RMNH.AVES.88521, adult, sex unknown, skin. Loc.: New Hebrides [= Vanuatu]. Ex: G.A. Frank, 1862.

Both specimens from Aneiteum (= Anatom Island) refer to *H. s. erromangae* (Mayr, 1938). RMNH.AVES.88521 is of unknown origin within the group of islands and could be any of five subspecies occurring in Vanuatu.

***Alcedoalbicilla*** Lesson & Garnot, 1826a: 338 (nec Dumont, 1823).

= *Todiramphussaurophagussaurophagus* (Gould, 1843).

Lectotype, RMNH.AVES.88546, adult male, mounted skin. Loc.: Dorey Bay, [Papua], [Indonesia], [26.vii–9.ix.1824]. Leg.: Voyage La Coquille.

Lesson and Garnot (1826a: 338) gave no indication of the number of specimens available to them. The listing as holotype by Van den Hoek Ostende et al. (1997: 183) is therefore an error but constitutes a lectotype designation. According to Lesson and Garnot (1826a: 338, 343) they encountered this species during their stay on the island Lambonne and near Port Praslin (New Ireland, Papua New Guinea, 12–20 August 1823). Dorey (= Manokwari) is not mentioned as type locality and in the description of their stay at Manokwari no mention is made of this species (Lesson and Garnot 1826b). The ship ‘La Coquille’ stayed in Manokwari from 26 July to 9 September 1824.

***Alcedotuta*** Gmelin, 1788: 453.

= *Todiramphustutustutus* (Gmelin, 1788).

Possible lectotype, RMNH.AVES.204880, adult, sex unknown, relaxed mount. Loc.: Otaiti [= Tahiti], [French Polynesia]. Leg.: [J. Cook?]. Ex: Bullock Museum, 1819.

Possible paralectotype, RMNH.AVES.204878.

Gmelin (1788) formally described this new species based on the description by Latham (1782: 624) of specimens collected during the voyages of Captain Cook. Lysaght (1959) stated that a bird in the collection of the RMNH received through the auctions of the former Bullock Museum in 1819 is the type of *Todiramphustutus*. RMNH.AVES.204878 dates to Cabinet Temminck (acquired between 1805 and 1807 and listed in the ‘Catalogue Systematique’ of 1807) and has “Type” written on a label which was added later. RMNH.AVES.204880, however, is from the Bullock auction (lot 5, 19 May 1819) and labelled as “Type” by Finsch more than half a century later. However, a link to the voyage of Cook is not proven for either of these specimens. See the contradicting conclusions of Jansen and Van der Vliet (2015), Lee and Holyoak (2017), and Van der Vliet and Jansen (2017) on the presence of the species on Tahiti.

#### ﻿Meropidae

***Meropogonforsteni*** “Temminck” Bonaparte, 1850a: 164.

= *Meropogonforsteni* Bonaparte, 1850.

Lectotype, RMNH.AVES.88623, adult male, relaxed mount. Loc.: Tondano, Celebes [= Sulawesi], [Indonesia], [15.iv.-xii], 1840. Leg.: E.A. Forsten.

Bonaparte (1850a) gave no indication of the number of specimens available to him. Schlegel (1863o: 8) designated the lectotype, a fact overlooked by Van den Hoek Ostende et al. (1997: 188), who listed RMNH.AVES.88623 as holotype.

Forsten visited the Tondano area between 15 April 1840 and 19 June 1841.

***Meropshirundinaceus*** Vieillot, 1817c: 21.

***Meropstavva*** “Levaillant” Boie, 1828: 316.

***Meropstaiva*** Cuvier, 1829: 271.

= *Meropshirundineushirundineus* A.A.H. Lichtenstein, 1793.

Syntype, RMNH.AVES.195239, adult, sex unknown, relaxed mount. Loc.: [Senegal]. Ex: Cabinet Temminck.

All three authors based their name on the description of “Le Guépier à queue fourchue” by Levaillant (1807: 35). Levaillant mentioned having collected 85 specimens.

***Meropsvariegatus*** Vieillot, 1817c: 25.

***MeropsSonninii*** “Levaillant” Boie, 1828: 316.

= *Meropsvariegatusvariegatus* Vieillot, 1817.

Lectotype of *variegatus*, syntype of *Sonninii*, RMNH.AVES.88622, adult, sex unknown, relaxed mount. Loc.: [Malimbe], [Angola]. Leg.: [Perrin]. Ex: Cabinet Temminck.

Vieillot (1817c) gave no indication of the number of specimens available to him but described a male and female. Levaillant mentioned in his description of his “Guépier à collier gros blue” (1807: 34) that he had seen five specimens, among them one in the collection of Vieillot and one in Temminck’s cabinet.

Van den Hoek Ostende et al. (1997: 188) erroneously listed RMNH.AVES.88622 as holotype for *variegatus*, thereby designating it lectotype.

***Meropsamictus*** Temminck, 1824: livr. 52, pl. 310.

= *Nyctyornisamictus* (Temminck, 1824).

Syntype, RMNH.AVES.88624, adult male, relaxed mount. Loc.: Padang, Sumatra, [Indonesia]. Leg.: [A.F.] van den Berg.

Temminck (1824) wrote having received two specimens from Van den Berg, of which only one could be found in the RMNH.

According to the original description other syntypes are in the MNHN, but not listed in Voisin and Voisin (2008b).

### ﻿PICIFORMES

#### ﻿Bucconidae

***Buccopulmentum*** Sclater, 1856a: 194.

= *Buccotamatiapulmentum* Sclater, PL, 1856.

Paralectotypes, RMNH.AVES.198437, 198439.

Sclater (1856a) based his new species on a manuscript name by Bonaparte and Verreaux. Sclater (in Sclater and Shelly 1891: 189) designated the lectotype.

***Monasatenebrio*** Temminck, 1825: livr. 54, pl. 323, fig. 1.

= *Chelidopteratenebrosatenebrosa* (Pallas, 1782).

Syntype, RMNH.AVES.198580, adult, sex unknown, mounted skin. Loc.: Cayenne.

Temminck (1825) gave no indication of specimens or their whereabouts. RMNH.AVES.198580 is marked on the stand with *Monasatenebrio* in Temminck’s hand (later overwritten with *C.tenebrosa*). We therefore consider it part of the type series.

***Monaca* [sic] *phaioleucos*** Temminck, 1825: pl. 323, fig. 2.

= *Nonnularubecularubecula* (Spix, 1824).

Syntype, RMNH.AVES.88654, adult female, mounted skin. Loc.: Brazil. Leg.: -.

Syntype, RMNH.AVES.88655, adult male, mounted skin. Loc.: Brazil. Leg.: -.

Syntype, RMNH.AVES.198529, adult female, mounted skin. Loc.: Ypanema [= Ipanema], Brazil. Leg.: J. Natterer. Ex: NMW 1863.

No livraison is mentioned, but according to Dickinson (2001: 47), pl. 323 was part of livr. 54. The specimen illustrated by Temminck is probably the female listed above as RMNH.AVES.88654. It is not clear how many specimens were available to Temminck. Since the label of the male is similar to that of the female, we assume that both specimens arrived in the museum simultaneously.

According to Schifter et al. (2007: 256) the type material was probably collected by Natterer. Six possible syntypes are in the NMW (NMW 56329–56335).

***Nonnularubeculatapanahoniensis*** Mees, 1968: 102.

= *Nonnularubeculatapanahoniensis* Mees, 1968.

Holotype, RMNH.AVES.38138, adult male, skin. Loc.: Paloemeu, Suriname, 19.xi.1965. Leg.: G.F. Mees, 28.iii.1966.

Paratypes, RMNH.AVES.38148 and 39194.

***Capitomelanotis*** Temminck, 1821: livr. 16, pl. 94.

= *Nystaluschacuruchacuru* (Vieillot, 1816).

Possible holotype, RMNH.AVES.88656, adult male, mounted skin. Loc.: Brazil. Leg.: -.

Following Dickinson et al. (2022) (see also introduction of this catalogue under “What is new in this 2023 version”: 14) the specimen illustrated on pl. 94 is the holotype, since the text that appeared later is irrelevant regarding type status. Any listing of other specimens as syntypes for this name in Van den Hoek Ostende et al. (1997: 195) and type catalogues of the MfN and NMW is erroneous. Therefore, neither RMNH.AVES.88657 and 88658, nor ZMB 10323 and 15153, nor NMW 57299–57305 or NMW 57307 and 57308 have type status.

According to Temminck (1821), the illustrated specimen is in the MNHN. However, according to Goffin (1863: 83), RMNH.AVES.88656 is the specimen illustrated on pl. 94. Hence it is listed here as “possible holotype”. Indeed, it is a perfect match with the specimen illustrated on pl. 94, also concluded by G.F. Mees and stated on the label. We advise the MNHN to check their specimens as they might have a better claim.

***Buccostriolatus*** Von Pelzeln, 1856: 500.

= *Nystalusstriolatusstriolatus* (Pelzeln, 1856).

Syntype, RMNH.AVES.88659, adult female, mounted skin. Loc.: [Engenho do Capitão Gama], [Mato Grosso], [Brazil], [03.viii.1826]. Leg.: [J. Natterer]. Ex: NMW, 1864.

This is one out of eight syntypes. Schifter et al. (2007: 254) listed five syntypes collected by Natterer in Engenho do Capitao Gama in the NMW (NMW 40756–40758, NMW 57295 and 57296) and mentioned that, according to Natterer’s handwritten record cards, RMNH.AVES.88659 was collected by Natterer at the same location on 03 August 1826.

#### ﻿Megalaimidae

***Mycropogonfuliginosus*** Temminck, 1830: livr. 83.

= *Caloramphusfuliginosusfuliginosus* (Temminck, 1830).

Syntype, RMNH.AVES.88660, adult male, mounted skin. Loc.: Borneo, [Indonesia]. Leg.: P.M. Diard.

Temminck (1830) described male(s) and female(s) but only RMNH.AVES.88660 could be found in the RMNH collection.

***Buccoarmillaris*** Temminck, 1821: livr. 15, pl. 89, fig. 1.

= *Psilopogonarmillarisarmillaris* (Temminck, 1821).

Holotype, RMNH.AVES.88662, adult male, relaxed mount. Loc.: Java, [Indonesia], [iv.1816–iii.1822]. Leg.: C.G.C. Reinwardt.

Following Dickinson et al. (2022) (see also Introduction of this catalogue under “What is new in this 2023 version”: 14) we have selected the holotype based on pl. 89, fig. 1, since the text that appeared on 28 February 1824 is irrelevant regarding type status. Goffin (1863: 23) already referred to RMNH.AVES.88662 being the specimen illustrated on pl. 89. Any listing of syntypes for this name in Van den Hoek Ostende et al. (1997: 196) or any other type catalogue is erroneous. The following specimens therefore have no type status: NMW 65570 (Schifter et al. 2007: 260) (arrived in March 1822 and the other in April 1830); MLC.2011.0.1252 (Gouraud 2015: 144) and MZUT Av1862 (Aimassi et al. 2020: 87). One specimen arrived in December 1823 in the MfN, one in Leuven in 1822.

The type locality was restricted to the Province of Batam, West Java (Chasen 1935: 136). Reinwardt visited Java several times between April 1816 and March 1822.

***Buccogularis*** “Reinwardt” Temminck, 1821: livr. 15, pl. 89, fig. 2.

***Buccocyanocephalus*** Reinwardt, 1820: Bataviasche Courant, febr. 1820 (not seen).

= *Psilopogonaustralis* (Horsfield, 1821).

Holotype, RMNH.AVES.88663, adult male, mounted skin. Loc.: Java, [Indonesia], [iv.1816–iii.1822]. Leg.: C.G.C. Reinwardt.

Following Dickinson et al. (2022) (see also Introduction of this catalogue under “What is new in this 2023 version”: 14) we have selected the holotype based on pl. 89, fig. 2, since the text that appeared later is irrelevant regarding type status. Any listing of syntypes for this name in Van den Hoek Ostende et al. (1997: 196) or any other type catalogue is erroneous. The following specimens have therefore no type status: RMNH.AVES.88664 (Van den Hoek Ostende et al. 1997: 196) and MZUT Av14884 (Aimassi et al. 2020: 87). One specimen arrived in the MfN in December 1823.

Reinwardt visited Java several times between April 1816 and March 1822.

***Buccochrysopogon*** Temminck, 1824: livr. 48, pl. 285.

= *Psilopogonchrysopogonchrysopogon* (Temminck, 1824).

Syntype, RMNH.AVES.88665, adult male, relaxed mount. Loc.: Padang, Sumatra, [Indonesia]. Leg.: [A.F.] van den Berg.

Temminck (1824) listed three specimens: RMNH.AVES.88665 in the RMNH and two syntypes from Diard and Duvaucel in the MNHN. One was sent to the NMW in February 1833 (no. 1833.VII.13).

***Megalaimachrysopsis*** Goffin, 1863: 15.

= *Psilopogonchrysopogonchrysopsis* (Goffin, 1863).

Syntype, RMNH.AVES.88666, adult male, skin. Loc.: Borneo, [Indonesia], ix.1846. Leg.: C.A.L.M. Schwaner.

Syntype, RMNH.AVES.88667, adult male, mounted skin. Loc.: Borneo, [Indonesia], [1843–1848]. Leg.: C.A.L.M. Schwaner.

Syntype, RMNH.AVES.88668, adult female, skin. Loc.: Borneo, [Indonesia], [1843–1848]. Leg.: C.A.L.M. Schwaner.

Syntype, RMNH.AVES.88669, adult female, skin. Loc.: Borneo, [Indonesia], [1843–1848]. Leg.: C.A.L.M. Schwaner.

Schwaner visited Borneo between 1843 and 1848.

***Buccocorvinus*** Temminck, 1831: livr. 88, pl. 522.

= *Psilopogoncorvinus* (Temminck, 1831).

Syntype, RMNH.AVES.88670, adult male, mounted skin. Loc.: Java, [Indonesia], [vi.1826–ix.1827]. Leg.: H. Boie.

Syntype, RMNH.AVES.198919, adult male, mounted skin. Loc.: Buitenzorg [= Bogor], Java, [Indonesia], viii.1826. Leg.: H. Boie.

Syntype, RMNH.AVES.198920, adult male, relaxed mount. Loc.: Buitenzorg [= Bogor], Java, [Indonesia], viii.1826. Leg.: H. Boie.

Syntype, RMNH.AVES.198921, adult, sex unknown, relaxed mount. Loc.: Mt. Salak, Java, [Indonesia], vi.1826. Leg.: H. Boie.

Syntype, RMNH.AVES.198922, adult female, relaxed mount. Loc.: Buitenzorg [= Bogor], Java, [Indonesia], xii.1826. Leg.: H. Boie.

Schifter et al. (2007: 259) listed a syntype in the NMW (NMW 65429). Aimassi et al. (2020: 87) listed two syntypes in MZUT (MZUT Av1858 and 1859). One was sent to the MfN in 1824, one to Leuven in August 1829.

Boie visited Java between June 1826 and September 1827.

***Buccofrontalis*** Temminck, 1831: livr. 88, text “Genre Barbu”.

= *Psilopogonduvauceliiduvaucelii* (Lesson, 1830).

According to the original description, type specimens are in the MNHN, NHM, and RMNH. Schifter et al. (2007: 261) listed a syntype from Borneo in the NMW (NMW 65621) which arrived in February 1833 (no. 1833.VII.10). No specimens from Sumatra are present in the RMNH which could be types.

***Buccofaiostriatus*** Temminck, 1831: livr. 88, text “Genre Barbu”.

***Buccofaiostrictus*** Temminck, 1832: livr. 89, pl. 527.

= *Psilopogonfaiostrictusfaiostrictus* (Temminck, 1832).

Syntype, RMNH.AVES.88671, adult, sex unknown, relaxed mount. Loc.: Cochinchina [= Vietnam]. Leg.: P.M. Diard.

According to the original description other syntypes are in the MNHN.

No livraison number is given underneath the text. According to Dickinson (2001: 38) pl. 527 was published in livraison 89 in 1832. Temminck’s name *faiostriatus* which was published one year earlier without description is considered a typing error for *faiostrictus*.

***Buccoaurifrons*** Temminck, 1831: livr. 88, text “Genre Barbu”.

= *Psilopogonflavifrons* (Cuvier, 1816).

Syntype, RMNH.AVES.88649, adult, sex unknown, relaxed mount. Loc.: Ceylon [= Sri Lanka]. Leg.: -.

Temminck (1831) first published this name in the introduction to the genus *Bucco* as species no. 12. He referred to Levaillant’s “Le Barbu à Front d’Or” (1806: II, pl. 55), which Temminck, in the text to *Buccoarmillaris* (Temminck, 1821), considered to be an immature specimen of this latter species. In livraison 88, Temminck described it as a separate species.

According to the original description other syntypes were present in the MNHN and in the collection of Levaillant.

***Buccoroseus*** Dumont, 1805: 52.

= *Psilopogonhaemacephalusroseus* (Dumont, 1805).

Holotype, RMNH.AVES.88672, adult, sex unknown, relaxed mount. Loc.: Java, [Indonesia]. Ex: Cabinet Temminck.

Dumont (1805) based his description on “Le Barbu Rose Gorge” by Levaillant (1806: pl. 33). Levaillant had seen this bird in the collection of Temminck. It is listed in his catalogue as a male from Java, no. 467 (Temminck 1807: 56). One specimen was sent to the NMW in 1821 (no. 1821.LXXIII.47).

Dumont’s name is frequently referred to as published in 1816, but Vol. IV and V and a few copies of VI were published in 1805 and 1806, after which publication was suspended until 1816 when volumes were brought up to date by Supplements (www.zoonomen.net).

***Buccohenricii*** Temminck, 1831: livr. 88, pl. 524.

= *Psilopogonhenriciihenricii* (Temminck, 1831).

Syntype, RMNH.AVES.88673, adult male, relaxed mount. Loc.: Padang, Sumatra, [Indonesia]. Leg.: A.H. Henrici.

One was sent to the MNHN in February 1843.

***BuccoKotoreas*** “Temminck” Horsfield, 1824: general catalogue (no pagination) (nomen novum).

= *Psilopogonjavensis* (Horsfield, 1821).

Horsfield (1824) introduced *Kotoreas* as a nomen novum for *Buccojavensis* Horsfield, 1821. The type series for *kotoreas* is therefore the same as for *javensis*, then in the collection of the East India Company, now in the NHM (Warren 1966: 147). His publication also predated *Buccokotorea* Temminck, 1831 (text to the genus *Barbu*), a fact overlooked by Aimassi et al. (2020: 87), who erroneously listed two syntypes for this name in the MZUT (MZUT Av1860 and 1861).

***Megalaimushodgsoni*** Bonaparte, 1850a: 144.

= *Psilopogonlineatushodgsoni* (Bonaparte, 1850).

Lectotype, RMNH.AVES.88679, adult, sex unknown, mounted skin. Loc.: Nepal. Leg.: B.H. Hodgson.

Paralectotype, RMNH.AVES.88680.

Goffin (1863: 34) designated the lectotype.

***Buccomystacophanos*** Temminck, 1824: livr. 53, pl. 315.

= *Psilopogonmystacophanosmystacophanos* (Temminck, 1824).

Syntype, RMNH.AVES.88674, adult male, mounted skin. Loc.: Sumatra, [Indonesia]. Leg.: P.M. Diard.

Syntype, RMNH.AVES.199171, adult male, relaxed mount. Loc.: Sumatra, [Indonesia]. Leg.: P.M. Diard.

According to the original description other syntypes collected by Diard and Duvaucel are in the MNHN. Schifter et al. (2007: 260) listed a possible syntype in the NMW (NMW 44795), but as no data link NMW 44795 to either Sumatra (type locality from original description) or Diard, its type status is doubtful.

***Megalæmanuchalis*** Gould, 1863: 283.

***Megalaimacalauchenia*** Goffin, 1863: 94.

= *Psilopogonnuchalis* (Gould, 1863).

Paralectotype for *nuchalis*, holotype for *calauchenia*, RMNH.AVES.88675, adult, sex unknown, mounted skin. Loc.: Formosa [= Taiwan], iv.1862. Leg.: R. Swinhoe, 1863.

Paralectotypes for *nuchalis*, RMNH.AVES.199468, 199470.

Holotype by monotypy for *calauchenia* as Goffin (1863) referred to RMNH.AVES.88675 only.

Shelley (Sclater and Shelley 1891: 75) designated the lectotype for *nuchalis* in the NHM (NHMUK 1889.2.1.10; Warren 1966: 210).

***BuccoOorti*** Müller, 1836: 341, pl. IV, fig. 4.

= *Psilopogonoorti* (Müller, 1836).

Holotype, RMNH.AVES.88676, adult male, mounted skin. Loc.: Sumatra, [Indonesia], [vi.1833–vii.1834]. Leg.: S. Müller.

Holotype by monotypy. Müller (1836) wrote having collected a single male.

Not 1835 as in Peters’ Checklist. The 1835 volume was published in 1836 (Richmond 1926: 141).

***Psilopogonpyrolophus*** Müller, 1836: 339, pl. IV, fig. 3.

= *Psilopogonpyrolophus* Müller, 1836.

Syntype, RMNH.AVES.88687, adult male, mounted skin. Loc.: Sumatra, [Indonesia], [vi.1833–1835]. Leg.: S. Müller.

Syntype, RMNH.AVES.88688, adult female, mounted skin. Loc.: Sumatra, [Indonesia], [vi.1833–1835]. Leg.: S. Müller.

Syntype, RMNH.AVES.88689, adult, sex unknown, skeleton. Loc.: Sumatra, [Indonesia], [vi.1833–1835]. Leg.: S. Müller.

Syntype, RMNH.AVES.88690, adult, sex unknown, skeleton. Loc.: Sumatra, [Indonesia], [vi.1833–1835]. Leg.: S. Müller.

Schifter et al. (2007: 258) listed a possible syntype in the NMW (NMW 67124).

Not dated 1835 as in Peters’ Checklist. The 1835 volume was published in 1836 (Richmond 1926: 141).

Müller visited west Sumatra from June 1833 until late 1835.

#### ﻿Lybiidae

***GymnobuccoPeli*** Hartlaub, 1857: 175.

= *Gymnobuccopeli* Hartlaub, 1857.

Lectotype, RMNH.AVES.88661, adult male, relaxed mount. Loc.: Ashantee, Dabocrom, Ghana, 1841. Leg.: H.S. Pel.

The lectotype was selected by Mees (2003: 247) because the alleged type series was composed of two taxa, RMNH.AVES.89442 being *Gymnobuccocalvus* (Lafresnaye, 1841). This was, however, unnecessary since Hartlaub based his description on Pel’s specimen from Dabocrom and on a specimen in the MNHN collected by M. Aubry-Lecomte in Gabon. This excludes RMNH.AVES.89442, listed in Van den Hoek Ostende et al. (1997: 196) as syntype from the type series as it is from Butre. The specimen in the MNHN was overlooked by Mees, who only referred to two syntypes in the RMNH.

***PogoniasBrucii*** Rüppell, 1837: 50, pl. 20, fig. 1.

= *Lybiusguifsobalito* Hermann, 1783.

Paralectotype, RMNH.AVES.90914.

The lectotype was designated by Steinbacher (1949: 108) and is in the SMF (SMF 12703). Paralectotypes are in the SMF and NHM (Steinheimer 2005a: 245).

According to the correspondence in the RMNH archives (‘Angebotene Vögel aus Abyssinien, abgebildet in meiner Abyssinia Fauna’ [‘Birds offered from Abyssinia, depicted in my Abyssinia Fauna’]), the RMNH specimen was one of the birds Rüppell sold to the museum between September and October 1837.

***Pogoniaspersonatus*** Temminck, 1823: livr. 34, pl. 201.

= *Lybiustorquatustorquatus* (Dumont, 1805).

Syntype, RMNH.AVES.200556, adult, sex unknown, relaxed mount. Loc.: South Africa. Leg.: -.

Temminck reported syntypes in the MNHN (Voisin and Voisin 2009: 134; MNHN-ZO-2008-756) and RMNH. Goffin (1863: 5) listed RMNH.AVES.200558, collected by H.B. van Horstok, as the specimen depicted on pl. 201. According to the label this specimen was received in 1833, so could not have been available at the time of description.

***Pogoniasundatus*** Rüppell, 1837: 52, pl. 20.

= *Lybiusundatusundatus* (Rüppell, 1837).

Paralectotype, RMNH.AVES.90913.

The lectotype was designated by Steinbacher (1949: 109) and is in the SMF (SMF 12616). Paralectotypes are in the SMF and NHM (Steinheimer 2005a: 245). According to Steinheimer (2005b: 181–182), the specimen in the NHM shows intermediate characters of *Lybiusu.undatus* and *L.u.thiogaster* Neumann, 1903, whereas all other type specimens (including the lectotype) belong to the nominate race.

According to the correspondence in the RMNH archives (‘Angebotene Vögel aus Abyssinien, abgebildet in meiner Abyssinia Fauna’ [‘Birds offered from Abyssinia, depicted in my Abyssinia Fauna’]), the RMNH specimen was one of the birds Rüppell sold to the museum between September and October 1837.

***Pogoniasrubescens*** Temminck, 1823: livr. 34, text “Genre Pogonias”.

***P[ogonias]Rubicon*** Cuvier, 1829: 457.

= *Lybiusvieillotirubescens* (Temminck, 1823).

Syntype, RMNH.AVES.200526, adult male, mounted skin. Loc.: Senegal. Leg.: -.

Temminck (1823, in the introduction to the genus) and Cuvier (1829) based their name on “Le Barbu Rubicon” by Levaillant (1807: 43, pl. D) who referred to three specimens in Cabinet Temminck, Dufresne’s collection and the MNHN.

***Megalæmabilineata*** Sundevall, 1850: 109.

= *Pogoniulusbilineatusbilineatus* (Sundevall, 1850).

Paralectotype, RMNH.AVES.200461.

Gyldenstolpe (1926: 78) selected the lectotype by listing an adult male (NRM 569758) in the NRM as type.

***Buccochrysoconus*** Temminck, 1832: pl. 536, fig. 2.

= *Pogoniuluschrysoconuschrysoconus* (Temminck, 1832).

Syntype, RMNH.AVES.88681, adult, sex unknown, relaxed mount. Loc.: Galam, Senegal. Leg.: -.

No livraison is mentioned in the text. According to Dickinson (2001: 47) pl. 536 is part of livraison 90, which fits with *Buccofrontalis* Temminck, 1832.

According to the original description other syntypes are in the MNHN.

***Barbatulauropygialis*** Heuglin, 1862: 37.

= *Pogoniuluschrysoconuschrysoconus* (Temminck, 1832).

Syntype, RMNH.AVES.200435, adult male, skin. Loc.: Bongo, [South Sudan], ix.1861. Leg.: T. von Heuglin.

***Buccopusillus*** Dumont, 1805: 50.

***BuccoRubrifrons*** Vieillot, 1816b: 497.

***BuccoParvus*** Cuvier, 1817: 428.

***Buccobarbatula*** Temminck, 1831: livr. 88, text to “Genre Barbu”.

= *Pogoniuluspusilluspusillus* (Dumont, 1805).

Syntype, RMNH.AVES.88682, adult, sex unknown, relaxed mount. Loc.: “Pays de Caffres”, [South Africa]. Leg.: ?F. Levaillant.

All authors based their description on Levaillant’s “Barbion” (1806: 73, pl. 32). Levaillant brought 14 specimens to Europe, one of which he gave to Temminck (Rookmaaker 1989: 256). This is probably the specimen listed here, according to Goffin (1863: 41) and the label that was added later. It is listed in Temminck’s catalogue as no. 89 (Temminck 1807: 56).

Dumont’s name is frequently referred to as published in 1816, but Vols IV and V and a few copies of Vol. VI were published in 1805 and 1806 after which publication was suspended until 1816 when volumes were brought up to date by Supplements (www.zoonomen.net).

RMNH.AVES.88682 is also syntype for *Buccominutus* Bonaparte, 1850.

***Buccominutus*** Bonaparte, 1850a: 144.

= *Pogoniuluspusilluspusillus* (Dumont, 1805).

Syntype, RMNH.AVES.88682, adult, sex unknown, relaxed mount. Loc.: “Pays de Caffres” [South Africa]. Leg.: ?F. Levaillant.

Syntype, RMNH.AVES.88683, adult, sex unknown, relaxed mount. Loc.: “Senegal” [error]. Leg.: -

Syntype, RMNH.AVES.88684, adult, sex unknown, relaxed mount. Loc.: “Africa/Caffrerie” [South Africa]. Leg.: -.

Syntype, RMNH.AVES.200403, adult, sex unknown, relaxed mount. Loc.: ”Caffrerie” [South Africa]. Leg.: H.B. van Horstok.

The locality Senegal for RMNH.AVES.88683 must be incorrect, since *P.pusillus* is an (south-)east African species. Bonaparte gave Sennaar and South Africa as the type locality.

***Xylobuccoscolopaceus*** “Temminck” Bonaparte, 1850a: 141.

= *Pogoniulusscolopaceusscolopaceus* (Bonaparte, 1850).

Lectotype, RMNH.AVES.88685, adult male, relaxed mount. Loc.: Ashantee, Dabocrom, Ghana, 1842. Leg.: H.S. Pel.

Paralectotype, RMNH.AVES.88686.

Goffin (1863: 48) designated the lectotype.

***Megalæmaleucotis*** Sundevall, 1850: 109.

= *Stactolaemaleucotisleucotis* (Sundevall, 1850).

Paralectotype, RMNH.AVES.200366.

Gyldenstolpe (1926: 78) selected the lectotype by listing an adult male (NRM 569757) in the NRM as type.

***Buccomargaritatus*** Cretzschmar, 1828: 30, pl. 20.

***Micropogonmargaritaceus*** Temminck, 1838: tabl. meth. 55.

= *Trachyphonusmargaritatusmargaritatus* (Cretzschmar, 1828).

Paralectotype for *margaritatus*, syntype for *margaritaceus*, RMNH.AVES.90910, adult male, mounted skin. Loc.: Sennaar, [Ethiopia]. Leg.: W.P.E.S. Rüppell.

Possible paralectotype for *margaritatus*, syntype for *margaritaceus*, RMNH.AVES.150507, adult male, mounted skin. Loc.: Abyssinia. Leg.: W.P.E.S. Rüppell.

Possible paralectotype for *margaritatus*, syntype for *margaritaceus*, RMNH.AVES.150508, adult female, mounted skin. Loc.: Abyssinia. Leg.: W.P.E.S. Rüppell.

The lectotype was designated by Steinbacher (1949: 109) and is in the SMF (SMF 12618). Paralectotypes are in the SMF and possibly in the NHM (Steinheimer 2005a: 245). Schifter et al. (2007: 267) listed two paralectotypes in the NMW (NMW 24539 and 24540).

Besides RMNH.AVES.90910, the RMNH holds two specimens that according to Steinheimer (2005a: 244) could also belong to the type series, but evidence is lacking.

***CapitoGoffinii*** “Schlegel” Goffin, 1863: 72.

= *Trachyphonuspurpuratusgoffinii* (Goffin, 1863).

Syntype, RMNH.AVES.88691, adult, sex unknown, relaxed mount. Loc.: Goudkust [= Ghana], iv.1860. Leg.: C.J.M. Nagtglas.

Syntype, RMNH.AVES.88692, adult, sex unknown, relaxed mount. Loc.: Goudkust [= Ghana], ix.1860. Leg.: C.J.M. Nagtglas.

Syntype, RMNH.AVES.88693, adult, sex unknown, relaxed mount. Loc.: Goudkust [= Ghana], vii.1861. Leg.: C.J.M. Nagtglas.

Peters (1948) mentioned Schlegel as the author. However, the catalogue of the Buccones in the RMNH was not written by Schlegel but by Goffin, who published this manuscript name by Schlegel.

Schifter et al. (2007: 266) listed another syntype in the NMW (NMW 44820) that arrived there in 1863. This is incorrect, as the type series consists of the specimens listed by Goffin in the original description (all still present in the RMNH), not the specimens indicated by Schlegel’s manuscript name.

***Trachyphonuslurpuratus* [sic**] Verreaux & Verreaux, 1851a: 260.

= *Trachyphonuspurpuratuspurpuratus* Verreaux & Verreaux, 1851.

Syntype, RMNH.AVES.200651, adult male, skin. Loc.: Gabon. Ex: Verreaux.

***PolysticteQuopopa*** Smith, 1836: 54.

= *Trachyphonusvaillantiivaillantii* (Ranzani, 1821).

Syntype, RMNH.AVES.200667, adult male, relaxed mount. Loc.: Kurrichaine [= Enzelsberg], [South Africa], [12.viii.1834–ii.1836]. Leg.: A. Smith.

Syntype, RMNH.AVES.200668, adult male, relaxed mount. Loc.: Kurrichaine [= Enzelsberg], [South Africa], [12.viii.1834–ii.1836]. Leg.: A. Smith.

***Pogoniasmelanocephala*** Cretzschmar, 1829: 41, pl. 28a.

= *Tricholaemamelanocephalamelanocephala* (Cretzschmar, 1829).

Paralectotypes, RMNH.AVES.90911–90912.

The lectotype was designated by Steinbacher (1949: 109: 263) and is in the SMF (SMF 12619). Paralectotypes are in the SMF, the NHM (Steinheimer 2005a: 244) and in the NMW (NMW 44830; Schifter et al. 2007: 263). The birds were collected by W.P.E.S. Rüppell during his first collecting trip to Kordofan between 1823 and 1825 (Steinheimer 2005a: 244).

#### ﻿Indicatoridae

***Indicatorarchipelagicus*** Temminck, 1832: livr. 91, pl. 542, fig. 1.

= *Indicatorarchipelagicus* Temminck, 1832.

Holotype, RMNH.AVES.88694, adult male, relaxed mount. Loc.: Pontianak, Borneo, [Indonesia], 1826. Leg.: P.M. Diard.

Holotype by monotypy. The original name was erroneously given as *archipelagus* in Van den Hoek Ostende et al. (1997: 200).

***Indicatormajor*** Stephens, 1815: 139.

= *Indicatorindicator* (Sparrman, 1777).

Syntype, RMNH.AVES.148252, adult, sex unknown, relaxed mount. Loc.: Cap, South Africa, [1781–1784]. Leg.: F. Levaillant. Ex: Cabinet Temminck.

Stephens (1815) based his name on “Le Grand Indicateur” by Levaillant (1807: 135–136, pl. 241; see Rookmaaker 1989). The specimen was listed by Temminck (1807: 57, no. 91).

***Indicatoralbirostris*** Temminck, 1825: livr. 62, pl. 367.

= *Indicatorindicator* (Sparrman, 1777).

Syntype, RMNH.AVES.88695, adult male, relaxed mount. Loc.: Senegal. Leg.: -.

Schlegel (1864b: 2) erroneously listed RMNH.AVES.200799 from South Africa as the depicted specimen on pl. 367. According to the original description other syntypes are in the MNHN and MfN. A bird from *Terracaffrorum* was sent to the NMW in September 1833 (1833.IX.7).

***Indicatorminimus*** “Vieillot” Temminck, 1832: livr. 91, pl. 542, fig. 1.

= *Indicatorminorminor* Stephens, 1815.

Possible lectotype, RMNH.AVES.200757, adult, sexe unknown, relaxed mount. Loc.: Cape [South Africa]. Leg.: -.

Temminck (1832) based this name on the description by Levaillant (1807: 137, pl. 242, not pl. 240 as stated by Temminck) but did not give any indication that specimens were available to him. According to Schlegel (1864b: 2) RMNH.AVES.200757 is the type of *minimus*. This is questionable as data with the specimen is lacking, but if correct constitutes a lectotype designation.

***Melignothespachyrhynchus*** Heuglin, 1864: 266.

= *Indicatorminordiadematus* Rüppell, 1837.

Holotype, RMNH.AVES.200759, adult, male, relaxed mount. Loc.: Bongo, [South Sudan], xi.1863. Leg.: Th. von Heuglin, 1865.

Holotype by monotypy. Von Heuglin (1864) described how he collected a single male near Bongo.

***IndicatorLevaillantii*** Temminck, 1825: livr. 62, text to “Genre Indicateur”.

= *Indicatorvariegatus* Lesson, 1830.

Temminck (1825) based this name on the description by Levaillant (1806: 139) of a bird that was observed, but not collected. Schlegel (1864b: 2) erroneously listed two specimens (RMNH.AVES.200778–200779) as types of *Levaillantii*.

***Prodotiscusregulus*** Sundevall, 1850: 109.

= *Prodotiscusregulusregulus* Sundevall, 1850.

Paralectotype, RMNH.AVES.88696.

Gyldenstolpe (1926: 79) selected the lectotype by listing an adult male (NRM 569751) in the NRM as type.

#### ﻿Picidae

***Dryocopuspercoccineus*** Bonaparte, 1850b: 134.

= *Campephilusrobustus* (Lichtenstein, 1819).

Syntype, RMNH.AVES.88731, adult female, mounted skin. Loc.: Buenos Aires, Argentina. Leg.: -.

Syntype, RMNH.AVES.88732, adult male, mounted skin. Loc.: Buenos Aires, Argentina. Leg.: -.

***Picusbalius*** Heuglin, 1871: 810.

= *Campetherapunctuligerabalia* (Heuglin, 1871).

Von Heuglin (1871) referred to a specimen in Stuttgart, incorrectly referred to as holotype (SMNS-5396) on the website of the German GBIF portal, and an immature in Leiden, which is no longer present.

***Picusgaleatus*** Temminck, 1822 (ex Natterer MS): livr. 29, pl. 171.

= *Celeusgaleatus* (Temminck, 1822).

The holotype (by monotypy), a single male collected by Natterer in Ipanema, Brazil, is in the NMW (NMW 57892; Schifter et al. 2007: 293).

***PicusSultaneus*** Hodgson, 1837d: 105.

= *Chrysocolaptesguttacristatussultaneus* (Hodgson, 1837).

Syntype, RMNH.AVES.205479, male, mounted skin. Loc.: Nepal. Leg.: B.H. Hodgson.

Warren (1966: 287) listed two syntypes in the NHM (NHMUK 1880.1.1.94 and a female without a registration number).

***DryotomusFlavigula*** Hodgson, 1837d: 106.

= *Chrysophlegmaflavinuchaflavinucha* (Gould, 1834).

Syntype, RMNH.AVES.202323, male, mounted skin. Loc.: Nepal. Leg.: B.H. Hodgson.

Warren (1966: 98) listed two syntypes in the NHM (NHMUK 1843.1.13.939 and another without a registration number).

***Picusmentalis*** Temminck, 1826: livr. 65, pl. 384.

***Picusgularis*** Wagler, 1827: no 89.

= *Chrysophlegmamentalementale* (Temminck, 1826).

Syntype, RMNH.AVES.88741, immature male, mounted skin. Loc.: Java, [Indonesia]. Leg.: -.

Syntype, RMNH.AVES.88742, adult male, mounted skin. Loc.: Java, [Indonesia]. Leg.: -.

Syntype, RMNH.AVES.88743, adult male, mounted skin. Loc.: Java, [Indonesia]. Leg.: -.

According to the original description other syntypes are in the MNHN.

Schifter et al. (2007: 291) listed two syntypes collected by Reinwardt on Java in the NMW (NMW 1379 and 37902). Both specimens (male and female) were received in exchange with the RMNH in 1823, three years prior to its description, and are not listed in the original description. They might therefore not be part of the type series. One specimen, a male, arrived in Berlin in December 1823.

*Picusgularis* Wagler, 1827 is based on the same series as *Picusmentalis* Temminck, 1826 as is a female specimen in the MNHN.

***Chrysophlegmaniasense*** Büttikofer, 1896c: 169.

= *Chrysophlegmaminiaceumniasense* Büttikofer, 1896.

Syntype, RMNH.AVES.88744, adult male, skin. Loc.: Hili Madjeio, Nias, [Indonesia], 16.xi.1895. Leg.: J.Z. Kannegieter.

Syntype, RMNH.AVES.88745, adult female, skin. Loc.: Hili Madjeio, Nias, [Indonesia], 17.xi.1895. Leg.: J.Z. Kannegieter.

Syntype, RMNH.AVES.88747, adult female, skin. Loc.: Hili Madjeio, Nias, [Indonesia], 29.xi.1895. Leg.: J.Z. Kannegieter.

Syntype, RMNH.AVES.88746, adult female, skin. Loc.: Gunung Sitolie, Nias, [Indonesia], 28.xii.1895. Leg.: J.Z. Kannegieter.

***Piculusrubiginosusfortirostris*** Mees, 1974: 57.

= *Colaptesrubiginosusnigriceps* (Blake, 1941).

Holotype, RMNH.AVES.72619, adult male, skin. Loc.: Nassau Mountains, Suriname, 09.viii.1972. Leg.: G.F. Mees.

Paratypes, RMNH.AVES.72620–72622.

***Piculusrubiginosuspoliocephalus*** Mees, 1974: 56.

= *Colaptesrubiginosusnigriceps* (Blake, 1941).

Holotype, RMNH.AVES.72616, adult male, skin. Loc.: Brownsberg, Suriname, 22.i.1972. Leg.: G.F. Mees.

Paratypes, RMNH.AVES.72617 and 72618.

***Picusanalis*** “Temminck” Bonaparte, 1850a: 137.

= *Dendrocoposanalisanalis* (Bonaparte, 1850).

Lectotype, RMNH.AVES.88706, adult male, relaxed mount. Loc.: Java, [Indonesia], [xii.1820–ix.1821]. Leg.: H. Kuhl and J.C. van Hasselt.

*Picusanalis* was first published by Horsfield (1824: general catalogue) as a nomen novum for *Picusminor* Linnaeus, 1758. Bonaparte (1850a) based his description on specimens in the RMNH labelled by Temminck with *analis* and specimens listed as *minor* by Horsfield (1822: 177) in the East India Company Museum. Warren (1966: 13) listed three syntypes in the NHM which were received in 1860 from the Honourable East India Company Museum. Two birds labelled by Temminck as *Picusanalis* arrived in the MfN in December 1823.

Van den Hoek Ostende et al. (1997: 201) erroneously listed RMNH.AVES.88706 as a holotype, hereby designating it the lectotype.

Kuhl and van Hasselt collected on Java from December 1820 until September 1821.

***Picusinsularis*** Gould, 1863: 283.

= *Dendrocoposleucotosinsularis* (Gould, 1863).

Syntype, RMNH.AVES.204366, adult male, mounted skin. Loc.: North Formosa [= Taiwan], iv.1862. Leg.: R. Swinhoe, 1863.

Syntype, RMNH.AVES.204370, adult male, mounted skin. Loc.: Campa Hill, Formosa [= Taiwan], iii.1862. Leg.: R. Swinhoe. Ex: G.A. Frank, 1867.

Of the six specimens in the RMNH collected by Swinhoe in Taiwan only the syntypes listed above have a collecting date and are unquestionably collected before the publication. Warren (1966: 141) listed a single syntype in the NHM (NHMUK 1863.2.16.19).

***PicusWestermani*** Blyth, 1870: 163.

= *Dendrocoposmaceiwestermani* (Blyth, 1870).

Holotype, ZMA.AVES.1940, adult male, relaxed mount. Loc.: Himalayas. Ex: NAM.

***Picusmelanauchen*** Heuglin, 1871: 808.

= *Dendropicosabyssinicus* (Stanley, 1814).

Syntype, RMNH.AVES.90755, adult male, mounted skin. Loc.: Waggera, Haute Abyssinia, [Ethiopia], i.1862. Leg.: Heuglin, 1864.

Syntype, RMNH.AVES.90756, immature, sex unknown, mounted skin. Loc.: Waggera, [Ethiopia], i.1862. Leg.: Heuglin, 1864.

***Picusminutus*** Temminck, 1823: livr. 33, pl. 197, fig. 2.

***Dendropicoselachus*** Oberholser, 1919: 8 (nomen novum).

= *Dendropicoselachus* Oberholser, 1919.

Syntype, RMNH.AVES.88709, adult male, mounted skin. Loc.: Senegal. Leg.: -.

Syntype, RMNH.AVES.88710, adult female, mounted skin. Loc.: Senegal. Leg.: -.

According to the original description other syntypes are in the MNHN.

Oberholser (1919) introduced *elachus* as a nomen novum for *Picusminutus* Temminck, 1823, preoccupied by *Picusminutus* Latham, 1790 (= *Picumnusspilogaster* Sundevall, 1866).

***Picusfulviscapus*** “Illiger” Lichtenstein, 1823: 11.

= *Dendropicosfuscescensfuscescens* (Vieillot, 1818).

Syntype, RMNH.AVES.88711, adult male, mounted skin. Loc.: Cap, South Africa. Ex: MfN, M.H.C. Lichtenstein.

Syntype, RMNH.AVES.88712, adult female, mounted skin. Loc.: Cap, South Africa. Leg.: -.

Syntype, RMNH.AVES.90753, adult female, mounted skin. Loc.: -. Ex: MfN, M.H.C. Lichtenstein.

Lichtenstein (1823) based his name on the description of “Le Pic à baguettes d’or” in Temminck (1807: 212). Schifter et al. (2007: 300) listed two possible syntypes in the NMW (NMW 24.724 and 24.725).

***Picusfuscescens*** Vieillot, 1818c: 86.

= *Dendropicosfuscescensfuscescens* (Vieillot, 1818).

Vieillot (1818c) based his description on “Le Pic à baguettes d’or” by Levaillant (1808: 25). Temminck (1807: 65) listed a specimen in his own collection under this vernacular name, which is no longer present in the RMNH.

***DendrobatesGabonensis*** Verreaux & Verreaux, 1851b: 513.

= *Dendropicosgabonensisgabonensis* (Verreaux & Verreaux, 1851).

Possible holotype, RMNH.AVES.204109, adult, sex unknown, mounted skin. Loc.: Gabon. Ex: Verreaux.

Verreaux and Verreaux (1851b) based their description on a single male from Gabon. Unequivocal data is lacking to link RMNH.AVES.204109 to the description.

***Picuserithronothos*** Vieillot, 1818c: 73.

***Picusneglectus*** Wagler, 1827: no. 29.

= *Dinopiumpsarodes* (Lichtenstein, 1793).

Holotype, RMNH.AVES.88713, adult, sex unknown, mounted skin. Loc.: Ceylon [= Sri Lanka]. Ex: Cabinet Temminck.

Holotype by monotypy. Temminck (1807) listed only a single male in his catalogue. Vieillot based his name on “Le pic à dos rouge” in the cabinet of Temminck (1807: 63, 212).

***Picusleucogaster*** Temminck, 1830: livr. 85, pl. 501.

= *Dryocopusjavensisjavensis* (Horsfield, 1821).

Syntype, RMNH.AVES.203481, adult, sex unknown, mounted skin. Loc.: Java, [Indonesia]. Leg.: -.

Syntype, RMNH.AVES.203482, adult female, mounted skin. Loc.: Java, [Indonesia]. Leg.: -.

Syntype, RMNH.AVES.203483, adult male, sex unknown, mounted skin. Loc.: Java, [Indonesia]. Leg.: -.

Syntype, RMNH.AVES.203484, adult, female, mounted skin. Loc.: Java, [Indonesia]. Leg.: -.

Syntype, RMNH.AVES.254589, adult, sex unknown, skeleton. Loc.: Java, [Indonesia], [iv.1816–iii.1822]. Leg.: C.G.C. Reinwardt.

Syntype, RMNH.AVES.255879, adult, sex unknown, skeleton. Loc.: Java, [Indonesia], [xii.1820–ix.1821]. Leg.: H. Kuhl and J.C. van Hasselt.

Temminck (1830) did not give an indication in which collection(s) the specimens, both male and female from Java, were kept. All specimens above refer to the original description in Temminck’s handwriting. Two arrived in the MfN in May 1827.

Reinwardt visited Java several times between April 1816 and March 1822. Kuhl and van Hasselt collected together on Java from December 1820 until September 1821.

***Picusconcretus*** Temminck, 1821: livr. 15, pl. 90, fig. 1 and 2.

= *Hemicircusconcretusconcretus* (Temminck, 1821).

Syntype, RMNH.AVES.88714, immature male, mounted skin. Loc.: Java, [Indonesia]. Leg.: -.

Syntype, RMNH.AVES.88715, adult female, mounted skin. Loc.: Java, [Indonesia]. Leg.: -.

Following Dickinson et al. (2022) (see also Introduction of this catalogue under “What is new in this 2023 version”: 14) the two specimens illustrated on pl. 90, figs 1 and 2, are the only two syntypes since the text that appeared on 28 February 1824 is irrelevant regarding type status. Any listing of other syntypes for this name in Van den Hoek Ostende et al. (1997: 202) or other type catalogues is erroneous. According to Temminck (1821), the illustrated specimens were in the RMNH. The following specimens have no type status: RMNH.AVES.88716 (Van den Hoek Ostende et al. 1997: 202), NMW 1568 and 44754 (Schifter et al. 2007: 303), and MLC.2011.0.1337 (Gouraud 2015: 144).

***Yunxjaponica*** Bonaparte, 1850a: 112.

= *Jynxtorquillachinensis* Hesse, 1911.

Lectotype, RMNH.AVES.88717, adult, sex unknown, mounted skin. Loc.: Japan. Leg.: - .

Bonaparte (1850a) gave no indication of the number of specimens available to him. Van den Hoek Ostende et al. (1997: 202) erroneously listed RMNH.AVES.88717 as holotype, thereby designating it the lectotype.

***Picuspoicilophos*** Temminck, 1823: livr. 33, pl. 197, fig. 1.

= *Meiglyptestrististristis* (Horsfield, 1821).

Syntype, RMNH.AVES.88718, adult male, mounted skin. Loc.: Java, [Indonesia]. Leg.: -.

Syntype, RMNH.AVES.88719, adult male, mounted skin. Loc.: Java, [Indonesia]. Leg.: -.

According to the original description, other syntypes are located in the MNHN and NMW (Schifter et al. 2007: 292; NMW 1460 and 44701).

***Picusmelanopogon*** “Lichtenstein” Temminck, 1828: livr. 76, pl. 451.

= *Melanerpesformicivorusformicivorus* (Swainson, 1827).

Syntype, RMNH.AVES.88720, adult male, mounted skin. Loc.: Mexico. Leg.: -.

Syntype, RMNH.AVES.88721, adult male, mounted skin. Loc.: Mexico. Leg.: -.

The original description is based on “a” male from Mexico received from Lichtenstein (MfN) in the RMNH. Temminck (1828) mentioned that the female is not yet known to him, but that specimens of both sexes can probably be found in the MfN where it was described under the name *Picusmelampogon* Deppe, 1830, two years later.

***Picuslarvatus*** Temminck, 1827: livr. 73, text to pl. 433.

= *Melanerpesradiolatus* (Wagler, 1827).

Syntype, RMNH.AVES.88722, adult male, skin. Loc.: Jamaica. Ex: Bullock Museum, 18.v.1819.

Syntype, RMNH.AVES.88723, adult male, skin. Loc.: Jamaica. Ex: Bullock Museum, 18.v.1819.

***Centuruscanescens*** Salvin, 1889: 370.

= *Melanerpessantacruzicanescens* (Salvin, 1889).

Syntype, RMNH.AVES.90975, adult male, skin. Loc.: Ruatan Island, Honduras, 1886. Leg.: G.F. Gaumer. Ex: O. Salvin, 1889.

Warren (1966: 50) listed several syntypes in the NHM (among others NHMUK 1898.3.14.358).

***Picussuperciliaris*** Temminck, 1827: livr. 73, pl. 433.

= *Melanerpessuperciliarissuperciliaris* (Temminck, 1827).

Lectotype, RMNH.AVES.88724, adult male, relaxed mount. Loc.: Cuba. Leg.: -.

The description gives no indication about the number of specimens. By listing it as holotype, Van den Hoek Ostende et al. (1997: 203) inadvertently designated RMNH.AVES.88724 the lectotype.

***Meiglyptesbadiosus*** “Temminck” Bonaparte, 1850a: 113.

= *Micropternusbrachyurusbadiosus* (Bonaparte, 1850).

Syntype, RMNH.AVES.88725, adult male, mounted skin. Loc.: Pontianak, Borneo, [Indonesia]. Leg.: P.M. Diard.

Syntype, RMNH.AVES.88726, adult female, mounted skin. Loc.: Pontianak, Borneo, [Indonesia]. Leg.: P.M. Diard.

Syntype, RMNH.AVES.88727, adult female, mounted skin. Loc.: Borneo, [Indonesia]. Leg.: -.

***Picuspulverulentus*** Temminck, 1826: livr. 66, pl. 389.

= *Mulleripicuspulverulentuspulverulentus* (Temminck, 1826).

Syntype, RMNH.AVES.88729, adult male, mounted skin. Loc.: Java, [Indonesia]. Leg.: -.

Syntype, RMNH.AVES.88730, adult, sex unknown, skeleton. Loc.: Java, [Indonesia]. Leg.: H. Kuhl and J.C. van Hasselt.

Syntype, RMNH.AVES.203338, adult male, mounted skin. Loc.: Java, [Indonesia]. Leg.: -.

Schifter et al. (2007: 293) listed another syntype (NMW 1.579) from Banda [Bangka] received in exchange from the RMNH in 1823. However, since Temminck (1826) did not mention the NMW in the original description and referred only to specimens from Java and Sumatra in the RMNH, evidence is lacking on whether this specimen belongs to the type series. One was sent to the MfN in May 1827.

***Hemilophusmülleri*** Bonaparte, 1850b: 131.

= *Mulleripicuspulverulentuspulverulentus* (Temminck, 1826).

Lectotype, RMNH.AVES.88728, immature, mounted skin. Loc.: Borneo, [Indonesia]. Leg.: C.A.L.M. Schwaner.

The description gives no indication about the number of specimens. By listing it as holotype, Van den Hoek Ostende et al. (1997: 203) inadvertently designated RMNH.AVES.88728 the lectotype.

***Picusaurulentus*** “Illiger” Temminck, 1821: livr. 10, pl. 59, fig. 1.

= *Piculusaurulentus* (Temminck, 1821).

Holotype, RMNH.AVES.88733, adult male, mounted skin. Loc.: Brazil. Leg.: -.

Following Dickinson et al. (2022) (see also Introduction of this catalogue under “What is new in this 2023 version”: 14) we have selected the holotype based on pl. 59, fig. 1, since the text that appeared later is irrelevant regarding type status. Any listing of syntypes for this name in Van den Hoek Ostende et al. (1997: 204) or any other type catalogue is erroneous. The following specimens have therefore no type status: RMNH.AVES.88734 (Van den Hoek Ostende et al. 1997: 204), ZMB 214 and 10585, and NMW 32948, 44703, and 44704.

***Picumnusasterias*** Sundevall, 1866: 97.

= *Picumnusalbosquamatusguttifer* Sundevall, 1866.

Syntype, RMNH.AVES.88735, adult male, mounted skin. Loc.: Brazil. Leg.: -.

Syntype, RMNH.AVES.88736, adult female, mounted skin. Loc.: Brazil. Leg.: -.

According to Peters (1948: 96), who listed *P.asterias* Sundevall, 1866 as a distinct species, it was only known from the “unique type”. However, Sundevall (1866) described a male and female (or immature) specimen in the RMNH. Later it was synonymized with *P.albosquamatusguttifer* Sundevall, 1866 (Short, 1982).

***Picumnuscirratus*** Temminck, 1825: livr. 62, pl. 371, fig. 1.

= *Picumnuscirratuscirratus* Temminck, 1825.

Syntype, RMNH.AVES.88737, adult male, mounted skin. Loc.: Brazil. Leg.: -.

***PicumnusTemminckii*** De Lafresnaye, 1845: 6.

= *Picumnustemminckii* Lafresnaye, 1845.

De Lafresnaye (1845) based his description on *Picumnusexilis* Lichtenstein as illustrated in Temminck (1825: pl. 371, fig. 2).

***Picusawokera*** Temminck, 1836: livr. 99, pl. 585.

= *Picusawokeraawokera* Temminck, 1836.

Syntype, RMNH.AVES.202200, adult, sex unknown [male], mounted skin. Loc.: Japan. Leg.: H. Bürger.

Syntype, RMNH.AVES.202202, adult male, mounted skin. Loc.: Japan. Leg.: H. Bürger.

Temminck (1836) described male(s) and female(s) and illustrated a male. The plate was erroneously labelled “kizuki” instead of “awokera”. Schifter et al. (2007: 290) listed a syntype collected by Siebold in the NMW (NMW57688) which arrived there in 1841.

Three races are recognized by IOC 11.1, but variation might “reflect a cline of decreasing size and increasing plumage darkness from N to S and a division of geographical races seems unjustified”, subsequently treated as monotypic (Winkler and Christie 2002: 541; Del Hoyo 2020: 338).

***Gecinusdedemi*** van Oort, 1911c: 59.

= *Picuscanusdedemi* (van Oort, 1911).

Holotype, RMNH.AVES.15058, adult male, skin. Loc.: Mt. Sibayak, [Sumatra], [Indonesia], 13.x.1909. Leg.: F.K. van Dedem.

***Gecinustancolo*** Gould, 1863: 283.

= *Picuscanustancolo* (Gould, 1863).

Syntype, RMNH.AVES.88739, adult male, mounted skin. Loc.: North Formosa [= Taiwan], iii.1862. Leg.: R. Swinhoe, 1863.

Syntype, RMNH.AVES.88740, adult female, mounted skin. Loc.: North Formosa [= Taiwan], iii.1862. Leg.: R. Swinhoe, 1863.

Warren (1866: 291) listed two syntypes in the NHM (NHMUK 1863.2.16.21 and another not specified).

***GecinusWeberi*** A. Müller, 1882: 421.

= *Picusviridanus* Blyth, 1843.

Syntype, RMNH.AVES.88748, adult male, mounted skin. Loc.: Salanga [= Phuket], Malacca [= Thailand]. Ex: Linnaea, vi.1886.

Syntype, RMNH.AVES.88749, adult female, mounted skin. Loc.: Salanga [= Phuket], Malacca [= Thailand]. Ex: Linnaea, vi.1886.

These are two of a series of 20 syntypes. Deignan (1961: 213) listed two syntypes in the NMNH (USNM 112665, 112666). Warren (1966: 311) listed two syntypes in the NHM (NHMUK 1884.2.12.1 and the other not specified).

***Picusdimidiatus*** Temminck, 1830: livr. 85, text to pl. 501.

= *Picusvittatus* Vieillot, 1818.

Syntype, RMNH.AVES.202288, adult male, mounted skin. Loc.: Java, [Indonesia], [xii.1820–ix.1821]. Leg.: H. Kuhl and J.C. van Hasselt.

Syntype, RMNH.AVES.254468, adult male, skeleton. Loc.: Java, [Indonesia], [xii.1820–ix.1821]. Leg.: H. Kuhl and J.C. van Hasselt.

Syntype, RMNH.AVES.255878, adult, sex unknown, skeleton. Loc.: Java, [Indonesia], [xii.1820–ix.1821]. Leg.: H. Kuhl and J.C. van Hasselt.

Syntype, RMNH.AVES.256572, adult female, skeleton. Loc.: Java, [Indonesia], [1818–1826]. Leg.: C.L. Blume.

Syntype, RMNH.AVES.256919, adult female, skeleton. Loc.: Java, [Indonesia], [1818–1826]. Leg.: C.L. Blume.

Syntype, RMNH.AVES.257114, adult female, skull. Loc. Java, [Indonesia], [1818–1826]. Leg.: C.L. Blume.

Syntype, RMNH.AVES.88750, immature male, mounted skin. Loc.: “Java”. Leg.: -. (= *Picusxanthopygaeus* (Gray & Gray, 1847)).

Temminck (1830) referred to Java, Sumatra, and the “Indian Continent” as collecting areas but gave no indication in which collection(s) he had seen the specimens. Schifter et al. (2007: 291) listed three syntypes from Java in the NMW (NMW 1308, 1309, and 1341). RMNH.AVES.88750 is also the syntype for *Gecinusxanthopygius* Bonaparte, 1850. A male and female were sent to the MfN in 1833.

Kuhl and van Hasselt collected together on Java from December 1820 until September 1821.

***Gecinusxanthopygius*** “Hodgson” Bonaparte, 1850a: 127.

= *Picusxanthopygaeus* (Gray & Gray, 1847).

Lectotype, RMNH.AVES.88750, immature male, mounted skin. Loc.: “Java”. Leg.: -.

Bonaparte (1850a) listed this species as “*Gecinusxanthopygius* Hodgs.”, using a different spelling from that of Hodgson. By 1850, however, Hodgson’s manuscript name had been validated by Gray and Gray (1847).

The locality Java is obviously in error, as the species does not occur there.

Bonaparte gave no indication of the number of specimens available to him. RMNH.AVES.88750 is the specimen referred to by Bonaparte as “*dimidiatus*, jun. Mus. Lugd.”. The listing as holotype in Van den Hoek Ostende et al. (1997: 206) is an error, but constitutes a lectotype designation.

***Picusvalidus*** Temminck, 1825: livr. 64, pl. 378 (male), pl. 402 (female).

= *Reinwardtipicusvalidusvalidus* (Temminck, 1825).

Syntype, RMNH.AVES.88697, adult male, relaxed mount. Loc.: Java, [Indonesia], [iv.1816–iii.1822]. Leg.: C.G.C. Reinwardt.

Syntype, RMNH.AVES.88698, adult male, relaxed mount. Loc.: Java, [Indonesia], [iv.1816–iii.1822]. Leg.: C.G.C. Reinwardt.

Syntype, RMNH.AVES.205437, adult female, relaxed mount. Loc.: Java, [Indonesia], [iv.1816–iii.1822]. Leg.: C.G.C. Reinwardt.

Syntype, RMNH.AVES.88699, adult, sex unknown, skeleton. Loc.: Java, [Indonesia], [xii.1820–ix.1821]. Leg.: H. Kuhl and J.C. van Hasselt.

According to the original description other syntypes are in the MNHN. Schifter et al. (2007: 304) listed two syntypes collected by Reinwardt on Java in the NMW (NMW 1556 and 1564).

Reinwardt visited Java several times between April 1816 and March 1822. Kuhl and van Hasselt collected together on Java from December 1820 until September 1821.

***Chrysocolaptesxanthopygius*** Finsch, 1905: 34.

= *Reinwardtipicusvalidusxanthopygius* (Finsch, 1905).

Syntype, RMNH.AVES.88701, adult male, skin. Loc.: Blue River, Upper Mahakan, C. Borneo, [Indonesia], xi.1898. Leg.: A.W. Nieuwenhuis.

Syntype, RMNH.AVES.88702, adult female, skin. Loc.: Blue River, Upper Mahakan, C. Borneo, [Indonesia], xi.1898. Leg.: A.W. Nieuwenhuis.

Syntype, RMNH.AVES.205388, adult male, mounted skin. Loc.: Liang Kubung, C. Borneo, [Indonesia], 16.iv.1894. Leg.: J. Büttikofer.

Syntype, RMNH.AVES.205389, adult male, mounted skin. Loc.: Upper Kapuas, C. Borneo, [Indonesia], 01.xii.1893. Leg.: J. Büttikofer.

The description was based on five specimens, three males and two females. One female specimen is no longer in the RMNH. Finsch (1905) also referred to specimens listed by Büttikofer (1887: 18) and Hargitt (1890: 459) for *Xylolepusvalidus* and *Chrysocolaptesvalidus*, respectively.

Skeletons RMNH.AVES.88699 and 88700 listed in Van den Hoek Ostende et al. (1997: 200) as syntypes are no longer considered part of the type series.

***Picumnusabnormis*** Temminck, 1825: livr. 62, pl. 371, fig. 3.

= *Sasiaabnormisabnormis* (Temminck, 1825).

Holotype, RMNH.AVES.88751, adult female, relaxed mount. Loc.: W. Java, [Indonesia], [xii.1820–ix.1821]. Leg.: H. Kuhl and J.C. van Hasselt.

Holotype by monotypy.

***Veniliaalbertuli*** Bonaparte, 1850a: 129.

= *Veniliornissanguineus* (Lichtenstein, 1793).

Syntype, RMNH.AVES.88752, adult female, mounted skin. Loc.: “Celebes” [error]. Leg.: “E.A. Forsten” [error].

Syntype, RMNH.AVES.88753, adult male, mounted skin. Loc.: “Celebes” [error]. Leg.: “E.A. Forsten” [error].

Bonaparte (1850a) described this species based on an unspecified number of specimens from “Celebes” in the “NAM” which must be erroneous as *Veniliornissanguineus* is only known from South America. RMNH.AVES.88752 and 88753 are the only two specimens in the RMNH collection with this locality data.

***Picuspercussus*** Temminck, 1826: livr. 66, pl. 390.

= *Xiphidiopicuspercussuspercussus* (Temminck, 1826).

Syntype, RMNH.AVES.88754, adult male, mounted skin. Loc.: Cuba, [1823–1824]. Leg.: E.F. Pöppig.

Syntype, RMNH.AVES.88755, adult female, mounted skin. Loc.: Cuba, [1823–1824]. Leg.: E.F. Pöppig.

The female was later depicted on pl. 424 (livr. 71, 1827). Both sexes are described in the text.

***Picuskaleënsis*** Swinhoe, 1863: 390.

= *Yungipicuscanicapilluskaleensis* (Swinhoe, 1863).

Syntype, RMNH.AVES.204735, adult female, mounted skin. Loc.: Formosa [= Taiwan], iv.1862. Leg.: R. Swinhoe, 1863.

Of the nine specimens in the RMNH collected by Swinhoe in Taiwan, the collecting date is only known for RMNH.AVES.204735 which is prior to the date of description.

***Picuskizuki*** Temminck, 1836: livr. 99, text to pl. 585.

= *Yungipicuskizukikizuki* (Temminck, 1836).

Syntype, RMNH.AVES.88703, adult female, mounted skin. Loc.: Japan. Leg.: -.

Syntype, RMNH.AVES.88704, adult male, mounted skin. Loc.: Japan. Leg.: -.

Syntype, RMNH.AVES.88705, adult female, mounted skin. Loc.: Japan. Leg.: -.

No plate is mentioned in the original description, although the name is erroneously added under the illustration of *Picusawokera* (pl. 585). The year of publication of livraisons 98 and 99 was corrected to 1836 by Dickinson (2001: 47), contrary to Sherborn, (1898: 488), who gave 1835 for both livraisons. Seebohm (1884) restricted the type locality to Kyushu (see Dekker et al. 2001: 207).

Schifter et al. (2007: 302) listed a syntype in the NMW (NMW 44761), collected by Siebold in Japan and received in 1841 in exchange with the RMNH.

***PicusTemminckii*** Malherbe, 1849: 529.

= *Yungipicustemminckii* (Malherbe, 1849).

Holotype, RMNH.AVES.88708, adult female, mounted skin. Loc.: Gorontalo, Celebes [= Sulawesi], [Indonesia], [ix.1841–iv.1842]. Leg.: E.A. Forsten.

Holotype by monotypy. Malherbe (1849) based his description on a single female in the RMNH.

Forsten visited the area around Gorontalo between September 1841 and April 1842.

***Picusboie*** Temminck, 1829: livr. 80, pl. 473.

= not applicable.

Holotype, RMNH.AVES.88738, adult, sex unknown, relaxed mount. Loc.: -. Ex: J. Raye van Breukelerwaert, 1827.

*Picusboie* is based on an artefact; a specimen composed of parts of various species.

### ﻿FALCONIFORMES

#### ﻿Falconidae

***Falcoaterrimus*** Temminck, 1821: livr. 7, pl. 37.

= *Daptriusater* Vieillot, 1816.

Following Dickinson et al. (2022) (see also Introduction of this catalogue under “What is new in this 2023 version”: 14) the specimen illustrated on pl. 37 is the holotype since the text that appeared later is irrelevant regarding type status. Any listing of syntypes for this name in any type catalogue is erroneous. According to Temminck (1821), the illustrated specimen is in the MNHN, where Voisin and Voisin (2002: 473–474) confirm the presence of MNHN C.G.2001-398 said to be the specimen illustrated on pl. 37 which should therefore be listed as holotype, not syntype. MNHN C.G.2001-398 is also the holotype of *Daptriusater* Vieillot, 1816 (Voisin and Voisin 2002: 473).

***Daptriusniger*** “Vieillot” Temminck, 1821: livr. 7, pl. 37.

= *Daptriusater* Vieillot, 1816.

Following Dickinson et al. (2022) (see also Introduction of this catalogue under “What is new in this 2023 version”: 14) the specimen illustrated on pl. 37 is the holotype since the text that appeared two years later is irrelevant regarding type status.

In the text to pl. 37, Temminck (1823) referred to a name supposedly given by Vieillot, *Daptriusniger*. Vieillot’s use of “*niger*” is, however, part of the description of the plumage of the bird, not the introduction of a new name. The use as name by Temminck constitutes a valid publication of this name.

***Falcobiarmicus*** Temminck, 1825: livr. 55, pl. 324.

= *Falcobiarmicusbiarmicus* Temminck, 1825.

Syntype, RMNH.AVES.87268, adult male, mounted skin. Loc.: “Cap”, [South Africa]. Leg.: -.

According to the original description, other syntypes are present in the MNHN but this is not confirmed by Voisin and Voisin (2002). Gouraud (2015: 145) referred to another syntype in the MLC (MLC.2011.0.395) and located two syntypes in the MNHN (MNHN-ZO-2012-737 and MNHN-ZO-2012-738; pers. comm., 2023).

***Falcolanariuscapensis*** Schlegel, 1862b: 16.

= *Falcobiarmicusbiarmicus* Temminck, 1825.

Syntype, RMNH.AVES.87269, adult male, mounted skin. Loc.: “Cap”, [South Africa]. Leg.: [H.B. van Horstok].

Syntype, RMNH.AVES.87270, adult male, mounted skin. Loc.: “Cap”, [South Africa]. Leg.: -.

Syntype, RMNH.AVES.87271, adult female, mounted skin. Loc.: Grote Vischrivier, [South Africa]. Leg.: J. Verreaux.

Syntype, RMNH.AVES.87268, adult male, mounted skin. Loc.: “Cap”, [South Africa]. Leg.: [H.B. van Horstok].

RMNH.AVES.87268, erroneously given as RMNH.AVES.87272 in Van den Hoek Ostende et al. (1997: 42–43), is also the syntype of *Falcobiarmicus* Temminck, 1825.

***FalcoFeldeggii*** Schlegel, 1843: 2, pl. 10 (ad. male) and 11 (imm. male).

= *Falcobiarmicusfeldeggii* Schlegel, 1843.

Syntype, RMNH.AVES.87273, adult female, mounted skin. Loc.: Dalmatia, [Croatia]. Leg.: C.F.F. von Feldegg [1829].

Schlegel (1843) based his description on three specimens collected by Feldegg in Dalmatia in 1829: an adult and an immature male in Feldegg’s private collection (now in the NMP; see Mlíkovský 2005: 115–116) and a female which was sent by Feldegg to the RMNH. Furthermore, Schlegel mentioned a specimen in the Mainzer Museum, collected near Hainau, Germany. The whereabouts of this specimen are unknown.

Mlíkovský (2005: 115–116) selected the adult male as lectotype. However, his statement did not fulfil the requirements of the Code for lectotype designations after 1999 (Art. 74.7.3 ICZN 1999 and Recommendation 74 G of ICZN Declaration 44, 2005). Neither using the wording as required by the Code nor giving any taxonomic purposes, this designation must be considered invalid. Consequently, all type specimens remain syntypes.

***Falcolanariusgraecus*** Schlegel, 1862b: 6, 15.

= *Falcobiarmicus* subsp.

Syntype, RMNH.AVES.191799, adult male, mounted skin. Loc.: Greece. Ex: F. Schulz, Dresden. (= *Falcobiarmicusfeldeggii* Schlegel, 1843).

Syntype, RMNH.AVES.191800, female, mounted skin. Loc.: Greece. Ex: F. Schulz, Dresden. (= *Falcocherrug* Gray, 1834).

Syntype, RMNH.AVES.191801, adult male, mounted skin. Loc.: Cairo, Egypt. Ex: Parzudacki, 1860. (= *Falcobiarmicustanypterus* Schlegel, 1843).

Syntype, RMNH.AVES.191802, adult female, mounted skin. Loc.: Egypt. Ex: Ruhl, 1842. (= *Falcobiarmicusfeldeggii* Schlegel, 1843).

Syntype, RMNH.AVES.191803, adult male, mounted skin. Loc.: Egypt. Leg. T. von Heuglin, 1861. (= *Falcobiarmicustanypterus* Schlegel, 1843).

Syntype, RMNH.AVES.191804, [imm.] male, mounted skin. Loc.: Suez, Egypt. Ex: E. Parzudaki, 1860. (= *Falcobiarmicustanypterus* Schlegel, 1843).

Syntype, RMNH.AVES.191808, adult male, mounted skin. Loc.: Chartum, Sudan. Leg.: L.W. Ruyssenaars, 1858. (= *Falcobiarmicustanypterus* Schlegel, 1843 or *F.b.abyssinicus* Neumann, 1904).

As Hartert (1915) had written, the type series of *Falcolanariusgraecus* Schlegel, 1862, includes two subspecies: *Falcobiarmicusfeldeggii* Schlegel, 1843 and *F.b.tanypterus* Schlegel, 1843. However, we do not exclude RMNH.AVES.191808 to represent a third subspecies: *F.b.abyssinicus* Neumann, 1904.

We identified RMNH.AVES.191800 as a male (not female as on the label) *Falcocherrug* Gray, 1834, based on measurements, structure of wing, toes, and claws and identification by an ornithologist with field experience with the species in Eastern Europe. This makes the type series of *Falcolanariusgraecus* Schlegel, 1862 a composite series of two species, including two or three subspecies of *F.biarmicus*. A lectotype selection is in place here which we suggest to publish appropriately at a later date and link the name *graecus* to Greece and hence select RMNH.AVES.191799 as lectotype. making *Falcolanariusgraecus* Schlegel, 1862, a senior synonym of *F.b.feldeggii* Schlegel, 1843.

***Falcotanypterus*** Schlegel, 1843: 2, pl. 12 and 13.

***Falcolanariusnubicus*** Schlegel, 1862b: 15.

= *Falcobiarmicustanypterus* Schlegel, 1843.

Syntype, RMNH.AVES.87274, adult female, mounted skin. Loc.: Nubia. Leg.: W.F. Hemprich and C.G. Ehrenberg. Ex: MfN, 1863.

Syntype, RMNH.AVES.87275, adult female, mounted skin. Loc.: Nubia. Leg.: W.F. Hemprich and C.G. Ehrenberg.

Syntype, RMNH.AVES.87276, adult female, mounted skin. Loc.: Nubia. Leg.: W.F. Hemprich and C.G. Ehrenberg.

*Falcotanypterus* was described by Schlegel in 1843, not 1862 as mentioned in Van den Hoek Ostende et al. (1997: 43). Six syntypes collected in Nubia by Hemprich and Ehrenberg are in the MfN (ZMB 998-1003).

According to Neumann (1904: 370), ZMB 998 was depicted on pl. 12 and ZMB 1002 probably on pl. 13. Schlegel (1862b: 16), however, stated that his cat. no. 1 is the specimen on pl. 12 and cat. no. 2 is depicted on pl. 13.

In his description of *Falcolanariusnubicus* Schlegel listed three specimens in the RMNH and referred to the specimens in Mainz and the MfN labelled *tanypterus*. Hence, these specimens in Mainz and MfN (ZMB 998-1003) are also part of the type series for *nubicus*. RMNH.AVES.87274, studied by Schlegel in the MfN, came to the RMNH in 1863. However, only the three syntypes listed above are still present in the RMNH: cat. 1 from the 1862 catalogue (1862b: 15) is missing.

***Falcoconcolor*** Temminck, 1825: livr. 56, pl. 330.

= *Falcoconcolor* Temminck, 1825.

Syntype, RMNH.AVES.87277, adult male, mounted skin. Loc.: Barakan, Red Sea. Leg.: W.P.E.S. Rüppell.

Syntype, RMNH.AVES.87278, adult female, mounted skin. Loc.: Barakan, Red Sea. Leg.: W.P.E.S. Rüppell.

According to the original description, other syntypes are present in the MNHN and MfN. Voisin and Voisin (2002) do, however, not mention *concolor* syntype(s) in the MNHN.

***FalcoBosschii*** Schlegel, 1863m: 123, pl. 5.

= *Falcocuvierii* Smith, 1830.

Holotype, RMNH.AVES.87279, adult, sex unknown, mounted skin. Loc.: [Elmina], Côte d’Or [= Ghana]. Leg.: C.J.M. Nagtglas, 1861.

Holotype by monotypy.

***Falcodeiroleucus*** Temminck, 1825: livr. 59, pl. 348.

= *Falcodeiroleucus* Temminck, 1825.

According to the original description, the holotype is in the MNHN, which is confirmed by Voisin and Voisin (2002: 478–479; MNHN C.G.2001-401).

***Falcofemoralis*** Temminck, 1822: livr. 21, pl. 121.

= *Falcofemoralisfemoralis* Temminck, 1822.

Syntype, RMNH.AVES.87280, male, mounted skin. Loc.: Ytararé, Brazil, [7.iii.1821]. Leg.: J. Natterer.

Syntype, RMNH.AVES.90744, male [= immature], mounted skin. Loc.: Ytararé, Brazil, [30.i.1821]. Leg.: J. Natterer.

Syntype, RMNH.AVES.90745, male, mounted skin. Loc.: Brazil. Leg.: J. Natterer.

Temminck published the description of *Falcofemoralis* twice: in livr. 21, pl. 121, in 1822 and in livr. 58, pl. 343, in 1825 (Dickinson 2001). Only the specimens available to Temminck in 1822 can belong to the type series. According to Schlegel (1862b: 21), Temminck based his description on three males.

According to the original description, other type specimens are in the NMW. Schifter et al. (2007: 82) listed two syntypes (male and female) collected by Natterer in Ytararé and Ipanema (NMW 40344 and 40345). However, Temminck explicitly wrote that no females had been sent by the Viennese travellers. Hence, the female listed by Schifter in Vienna is not part of the type series.

Voisin and Voisin (2002: 480) mentioned a syntype in the MNHN (MNHN C.G.2001-400). It was collected by A. Saint-Hilaire in August 1822 and refers to pl. 343 on its label (“type of plate”). As pl. 343 is not part of the original description (see previous paragraph) and because the original description in livr. 21, pl. 121, was published in April 1822 (Dickinson 2001: 46), four months before the MNHN specimen was collected, MNHN 2001-400 is not part of the type series.

***Falcoreligiosus*** Bonaparte, 1850: 25.

= *Falcolongipennislongipennis* Swainson, 1837.

Lectotype, RMNH.AVES.87281, adult [= immature] male, relaxed mount. Loc.: Ceram, [Indonesia], 1842. Leg.: E.A. Forsten.

Van den Hoek Ostende et al. (1997: 44) attributed this name to Sharpe (1874: 397). However, this manuscript name by Temminck was first published by Bonaparte in the synonymy of *Falcoseverus*. He considered this form a dark morph (“var. nigra”) of *severus.* As Bonaparte (1850) gave Ceram [= Seram] as the locality, only RMNH.AVES.87281 is part of the type series as no other specimens from Ceram [= Seram] from before 1850 are present in the RMNH.

Sharpe (1874: 398) referred to RMNH.AVES.87281 as “type of the species”, hereby designating it the lectotype.

***Tinnunculusmoluccensisorientalis*** Meyer & Wiglesworth, 1898: 79 (nec Brehm, 1851).

***Falcomoluccensisbernsteini*** Stresemann, 1919: 8 (nomen novum).

= *Falcomoluccensismoluccensis* (Bonaparte, 1850).

Syntype, RMNH.AVES.87283, adult male, mounted skin. Loc.: Gilolo, Halmahera, [Indonesia]. Leg.: A.R. Wallace, 1860.

Syntype, RMNH.AVES.90984, adult female, mounted skin, Loc.: Ternate, [Indonesia]. Leg.: E.A. Forsten (no. 19).

Syntype, RMNH.AVES.90985, immature male, mounted skin. Loc.: Ternate, [Indonesia], 17.xii.1860. Leg.: H.A. Bernstein.

Syntype, RMNH.AVES.90986, immature female, mounted skin. Loc.: Ternate, [Indonesia], 19.iv.1861. Leg.: H.A. Bernstein.

Syntype, RMNH.AVES.90987, immature male, mounted skin. Loc.: Galela, Halmahera, [Indonesia], 25.vii.1861. Leg.: H.A. Bernstein.

Syntype, RMNH.AVES.90988, adult male, mounted skin. Loc.: Galela, Halmahera, [Indonesia], 17.vii.1861. Leg.: H.A. Bernstein.

Syntype, RMNH.AVES.90989, adult female, mounted skin. Loc.: Galela, Halmahera, [Indonesia], 24.vii.1861. Leg.: H.A. Bernstein.

Syntype, RMNH.AVES.90990, immature male, mounted skin. Loc.: Dodingo, Halmahera, [Indonesia], 19.iv.1862. Leg.: H.A. Bernstein.

Syntype, RMNH.AVES.90991, adult female, mounted skin. Loc.: Batjan, [Indonesia], i.1861. Leg.: H.A. Bernstein.

Syntype, RMNH.AVES.90992, adult male, mounted skin. Loc.: Ternate, [Indonesia], 12.x.1862. Leg.: H.A. Bernstein.

Syntype, RMNH.AVES.90993, immature male, mounted skin. Loc.: Mareh, [Indonesia], 11.ix.1863. Leg.: H.A. Bernstein.

Syntype, RMNH.AVES.90994, immature male, mounted skin. Loc.: Tidore, [Indonesia], 01.iv.1862. Leg.: H.A. Bernstein.

Syntype, RMNH.AVES.90995, adult male, mounted skin. Loc.: Morotai, [Indonesia], 21.ix.1861. Leg.: H.A. Bernstein.

In their description, Meyer and Wiglesworth (1898) refer to Schlegel (1866a: 47, pl. 1, fig. 3). By doing so, they included all specimens from Halmahera and the surrounding islands that Schlegel mentioned in his work.

Wallace visited Halmahera for the first time in February 1858 and made several subsequent visits. Forsten visited Ternate from 19 June 1841 until mid September 1841.

Stresemann (1919) introduced *Falcomoluccensisbernsteini* as a nomen novum for *Tinnunculusmoluccensisorientalis* Meyer & Wiglesworth, 1898, preoccupied by *Cerchneisorientalis* C.L. Brehm (1851: 75).

***Falcoperegrinusharterti*** Buturlin, 1907: 99.

= *Falcoperegrinusjaponensis* Gmelin, 1788.

Syntype, ZMA.AVES.43803, adult male, skin. Loc.: Alazeya Delta, [NE Siberia], [Russia], 21.vii.1905. Coll.: S.A. Buturlin. Ex: Snouckaert/van Marle (3803).

Syntype, ZMA.AVES.43804, adult female, skin. Loc.: Kolyma Delta, [NE Siberia], [Russia], vii.1905. Coll.: S.A. Buturlin. Ex: Snouckaert/van Marle (3804).

The name was based on a series of skins. Sudilovskaya (1962: 436) referred to a “type” from the Kolyma delta (ZMM R-5119, male, spring 1905) and a “co-type” from Abai (at 63°N on the Indigirka R.; ZMM R-6369, female, spring 1905), both collected by Buturlin. According to Roselaar and Prins (2000: 99) this constituted a lectotype selection. However, in using the term “type” and “co-type” (= syntype) Sudilovskaya only listed two specimens with equal type status (ICZN 73.2.1.) and failed to unambiguously select a particular syntype as lectotype (ICZN 74.5).

***Falcominor*** Bonaparte, 1850a: 23.

= *Falcoperegrinusminor* Bonaparte, 1850.

Syntype, RMNH.AVES.87285, adult male, mounted skin. Loc.: “Cap”, [South Africa]. Leg.: H.B. van Horstok.

Syntype, RMNH.AVES.87286, adult male, mounted skin. Loc.: “Cap”, [South Africa]. Leg.: H.B. van Horstok.

Syntype, RMNH.AVES.87287, adult male, mounted skin. Loc.: “Cap”, [South Africa]. Leg.: H.B. van Horstok.

Syntype, RMNH.AVES.87288, adult female, mounted skin. Loc.: “Cap”, [South Africa], 19.iv.1828. Leg.: H.B. van Horstok.

Syntype, RMNH.AVES.87289, immature male, mounted skin. Loc.: “Cap”, [South Africa]. Leg.: H.B. van Horstok.

RMNH.AVES.87285 was later labelled “male”. There remains some doubt whether all specimens belong to the same race.

***Falcopelegrinoides*** Temminck, 1829: livr. 81, pl. 479.

= *Falcoperegrinuspelegrinoides* Temminck, 1829.

Syntype, RMNH.AVES.87284, adult male, mounted skin. Loc.: Dongola, Nubia, [Sudan]. Leg.: [Rüppell].

In the original description, Temminck (1829) referred to specimens reported by Rüppell from Nubia and in the RMNH from Baie d’Algoa. These South African specimens are no longer in the RMNH. According to Schlegel (1862b: 6) RMNH.AVES.87284 is the depicted specimen on pl. 479.

***Falcopunctatus*** “Cuvier” Temminck, 1821: livr. 8, pl. 45.

= *Falcopunctatus* Temminck, 1821.

Possible holotype, RMNH.AVES.87290, adult male, mounted skin. Loc.: Mauritius. Ex: MNHN.

Following Dickinson et al. (2022) (see also Introduction of this catalogue under “What is new in this 2023 version”: 14) the specimen illustrated on pl, 45 is the holotype since the text that appeared later is irrelevant regarding type status. According to Temminck (1821), the illustrated specimen was in the MNHN. However, RMNH.AVES.87290, erroneously listed as syntype in Van den Hoek Ostende et al. (1997: 45), seems to fit pl. 45 better than the two “syntypes” in the MNHN based on online digital photos (MNHN 2001-388 and 2001-389; Voisin and Voisin 2002: 479), hence it has been listed here as “possible holotype” until proven otherwise. RMNH.AVES.87290 has been donated by the MNHN to the RMNH at an unknown date and might have been part of the MNHN collection when the plate was prepared in 1821.

***Falcosmithii*** Schlegel, 1873a: 43 (nomen novum).

= *Falcorupicoloidesrupicoloides* A. Smith, 1829.

Schlegel (1873a) introduced *smithii* as an unnecessary nomen novum. Sharpe (1874: 432) claimed the type (adult female. Loc.: South Africa) to be in the South African Museum. Warren (1966: 256) listed a specimen in the NHM (NHMUK 1843.2.28.85, adult female. Loc.: South Africa. Leg.: A. Smith) as “? Holotype”.

***Falcogyrfalcogroenlandicus*** Schlegel, 1862b: 13.

***Falcoholbœlli*** Sharpe, 1873: 415.

= *Falcorusticolus* Linnaeus, 1758.

Syntype, RMNH.AVES.192229, adult male, mounted skin. Loc.: Godthaab, Greenland. Ex: R. Conradsen, 1859.

Syntype, RMNH.AVES.192230, adult male, mounted skin. Loc.: Godthaab, Greenland. Ex: Herrnhutters, 1858.

Syntype, RMNH.AVES.192238, adult male, mounted skin. Loc.: Godthaab, Greenland. Ex: Hernhutters, 1858.

Syntype, RMNH.AVES.192239, immature male, mounted skin. Loc.: Juliaanshaab, Greenland. Leg.: -, winter 1858–1859. Ex: R. Conradsen, 1859–1860.

Syntype, RMNH.AVES.192240, immature male, mounted skin. Loc.: Juliaanshaab, Greenland. Ex: R. Conradsen, 1859–1860.

Syntype, RMNH.AVES.192241, immature male, mounted skin. Loc.: Juliaanshaab, Greenland. Ex: R. Conradsen. 1859–1860.

Syntype, RMNH.AVES.192242, immature female, mounted skin. Loc.: Juliaanshaab, Greenland. Ex: R. Conradsen, 1859–1860.

Syntype, RMNH.AVES.192243, immature female, mounted skin. Loc.: Juliaanshaab, Greenland. Ex: R. Conradsen, 1859–1860.

Syntype, RMNH.AVES.192244, juvenile female, mounted skin. Loc.: Greenland. Leg.: C.P. Hollböll.

Syntype, RMNH.AVES.192245, adult female, mounted skin. Loc.: Godthaab, Greenland. Ex: R. Conradsen, 1859–1860.

Syntype, RMNH.AVES.192246, adult female, mounted skin. Loc.: Juliaanshaab, Greenland. Ex: R. Conradsen, 1859–1860.

Syntype, RMNH.AVES.192247, immature female, mounted skin. Loc.: Juliaanshaab, Greenland. Ex: R. Conradsen, 1859–1860.

The labels of the specimens received from Conradsen give 1859 as date while on the stand 1860 is given. Probably all these Conradsen specimens were collected in the winter of 1859–1860. Only in the case of RMNH.AVES.192229 this date is given on the stand of the specimen.

Van den Hoek Ostende et al. (1997: 45) selected the wrong specimens as types, probably due to mix up of the old catalogue numbers.

Sharpe (1873) introduced *Falco holbœlli* to clarify the nomenclature of the Greenland gyrfalcons. In his opinion there are two taxa in Greenland: *candicans* and a Greenland form of the Islandic gyrfalcon *islandus*. Earlier Hollböll (1854) described a form of Greenland gyrfalcon as *Falcoarcticus.* Sharpe identified this form as similar to *islandus* and included it as well as *Falcogyrfalcogroenlandicus* Schlegel, 1862 in *Falco holbœlli*. A syntype for *Falco holbœlli* is in the NHM (NHMUK 1872.11.8.13; Warren 1966: 129).

***Falcogyrfalconorwegicus*** Schlegel, 1862b: 12.

= *Falcorusticolus* Linnaeus, 1758.

Syntype, RMNH.AVES.87303, adult female, mounted skin. Loc.: Plateau de Dorrefield, Norway, 1842. Ex: Prins Alexander van Oranje-Nassau.

Syntype, RMNH.AVES.87304, immature female, mounted skin. Loc.: Plateau de Dorrefield, Norway, ix.1842. Ex: Prins Alexander van Oranje-Nassau.

Syntype, RMNH.AVES.87305, immature female, mounted skin. Loc.: Plateau de Dorrefield, Norway, ix.1842. Ex: Prins Alexander van Oranje-Nassau.

Syntype, RMNH.AVES.87306, immature male, mounted skin. Loc.: Bergen, Norway. Leg.: Hög, 1848.

Syntype, RMNH.AVES.87307, immature male, mounted skin. Loc.: Noordwijk, The Netherlands, 16.x.1849. Leg.: F.A. Verster van Wulverhorst.

RMNH.AVES.87303–87305 were caught for the falconry of H.R.H. Prince W. Alexander C.H.F. van Oranje-Nassau.

***Falcoaldrovandii*** Temminck, 1822: livr. 22, pl. 128.

= *Falcoseverus* Horsfield, 1821.

Syntype, RMNH.AVES.87308, adult female, mounted skin. Loc.: Java, [Indonesia]. Leg.: C.G.C. Reinwardt.

Temminck (1822) mentioned having two specimens: only RMNH.AVES.87308 is now present in the RMNH. The other specimen was sent to the MfN in May 1827.

***Falcotinnunculusjaponicus*** Temminck & Schlegel, 1844: 2, pl. 1.

***Cerchneistinnunculusmanchuricus*** Stuart Baker, 1930: vol. 7 (2^nd^ ed.): 403 (nomen novum).

= *Falcotinnunculusinterstinctus* McClelland, 1840.

Syntype, RMNH.AVES.87310, adult female [immature male], mounted skin. Loc.: Japan. Leg.: Ph.F.B. von Siebold.

Syntype, RMNH.AVES.87311, adult male [immature female], mounted skin. Loc.: Japan. Leg.: Ph.F.B. von Siebold.

Syntype, RMNH.AVES.87312, adult male, mounted skin. Loc.: Nagasaki, Japan. Leg.: Ph.F.B. von Siebold.

Syntype, RMNH.AVES.87313, immature female, mounted skin. Loc.: Nagasaki, Japan. Leg.: Ph.F.B. von Siebold.

RMNH.AVES.87310 is not an adult female as written under the stand, but an immature male. RMNH.AVES.87311 is not an adult male as written under the stand and on the label, but an immature female. According to Schlegel (1862b: 27) RMNH.AVES.87310 is the depicted specimen on pl. 1.

Temminck and Schlegel (1844: 2–4) described this species based on young females, an adult female, and an adult male, all of which agree with the number and composition of the type series.

*Cerchneistinnunculusmanchuricus* Stuart Baker, 1930, is a nomen novum for *Falcotinnunculusjaponicus* Temminck & Schlegel, 1844, and thus relates to the same type specimens.

See also Dekker et al. (2001) and Morioka et al. (2005).

***Falconeglectus*** Schlegel, 1873a: 43.

= *Falcotinnunculusneglectus* Schlegel, 1873.

Holotype, RMNH.AVES.87309, adult female, mounted skin. Loc.: St. Vincent, [Cape Verde], i.1865. Leg.: J.G. Keulemans.

Holotype by monotypy. Schlegel (1873a) only listed the above-mentioned specimen.

***Asturmirandollei*** Schlegel, 1862c: 27.

= *Micrasturmirandollei* (Schlegel, 1862).

Holotype, RMNH.AVES.87314, adult female, skin. Loc.: Suriname. Leg.: Mirandolle.

Holotype by monotypy. Schlegel (1862c: 27) only listed RMNH.AVES.87314 as type. Although Schlegel referred to his 1863e publication as the original description, this article in ‘Nederlandsch Tijdschrift voor Dierkunde’ was published after Schlegel (1862c).

***Micrasturmacrorhynchus*** Von Pelzeln, 1865: 11.

= *Micrasturmirandollei* (Schlegel, 1862).

Syntype, RMNH.AVES.87315, adult female, skin. Loc.: Barra do Rio Negro [= Manaus], Brazil, 4.x.1830. Leg.: J. Natterer. Ex: NMW, 1862.

According to Schifter et al. (2007: 80) the original description was based on three specimens, two of which are in the NMW (NMW 31853 and 39969). The third specimen, a female collected by Natterer on 4 October 1830, now in the RMNH, was sent in exchange in 1862 (in 1864 according to Schlegel 1873a: 68).

***Falcoxanthothorax*** Temminck, 1821: livr. 16, pl. 92.

= *Micrasturgilvicollis* (Vieillot, 1817).

Holotype, RMNH.AVES.87316, adult male, mounted skin. Loc.: Cayenne. Leg.: -.

Following Dickinson et al. (2022) (see also Introduction of this catalogue under “What is new in this 2023 version”: 14) we have selected RMNH.AVES.87316 as the holotype based on pl. 92, since the text that appeared later is irrelevant regarding type status. According to Temminck (1821), the illustrated specimen was in the RMNH. Van den Hoek Ostende et al. (1997: 47) listed this specimen erroneously as syntype. Schifter et al. (2007: 79) erroneously listed four specimens collected by Natterer in Ipanema and Mattodentro in the NMW (NMW 40.002–40.005) as syntypes. For the same reason, five specimens in the MfN (ZMB 885–888, 890, Brazil, leg.: Sellow) should no longer be considered syntypes.

The identification of RMNH.AVES.87316 as *Micrasturgilvicollis* (Vieillot, 1817) has been confirmed by G.F. Mees. See also Ferguson-Lees and Christie (2001: 815).

***Falcoleucauchen*** Temminck, 1824: livr. 52, pl. 306.

= *Micrasturruficollisruficollis* (Vieillot, 1817).

Syntype, RMNH.AVES.90743, adult female, mounted skin. Loc.: Brazil, [1817–1822]. Leg.: Natterer.

Syntype, RMNH.AVES.193032, adult male, mounted skin. Loc.: Brazil, [1817–1822]. Leg.: Natterer.

According to the original description, other syntypes collected by Natterer are in the museums in the MNHN and NMW. Voisin and Voisin (2002: 476) listed two syntypes collected by A.F.C.P. Saint-Hilaire in Brazil in the MNHN (MNHN C.G.2001-393 and C.G.2001-394). A third specimen (MNHN C.G.2001-409) collected by L.C. de S. de Freycinet during the expedition of the ’Uranie’ in Brazil (1820) was probably not part of the type series since Temminck mentioned only specimens collected by A. Saint-Hilaire (Voisin and Voisin 2002: 476). Schifter et al. (2007: 79–80) mentioned that it could not be decided unambiguously which syntypes of *xanthothorax* might also belong to the type series of the name *leucauchen*. However, the type specimens fit the description of *leucauchen* (see above).

Schlegel (1862c: 51) stated that RMNH.AVES.90743 is the depicted specimen on pl. 306.

***Falcobrachypterus*** Temminck, 1822: livr. 20, pl. 116.

= *Micrastursemitorquatussemitorquatus* (Vieillot, 1817).

Holotype, RMNH.AVES.87317, immature male, mounted skin. Loc.: Brazil. Leg.: J. Natterer.

Following Dickinson et al. (2022) (see also Introduction of this catalogue under “What is new in this 2023 version”: 14) we have selected RMNH.AVES.87317 as the holotype based on pl. 116 which was published in March 1822. Van den Hoek Ostende et al. (1997: 47) listed this specimen erroneously as syntype. The text that appeared in August 1822, five months after pl. 116, is irrelevant regarding type status.

An adult male which is depicted on pl. 141 in livraison 24 is in the NMW (NMW 39971; Schifter et al. 2007: 81). However, pl. 141 appeared in July 1822, four months after pl. 116 (March 1822). The specimen illustrated on pl. 141 therefore has no type status.

### ﻿PSITTACIFORMES

#### ﻿Cacatuidae

***LophochroaLeari*** Finsch, 1863: xxiii.

= *Cacatuaducorpsii* Pucheran, 1853.

Holotype, ZMA.AVES.143, sex unknown, skin. Loc.: -. Ex: NAM, died in or before 1871.

Holotype by monotypy. Finsch (1863) based this taxon on a single live bird in the zoological garden of NAM which became part of the collection of the ZMA (Roselaar and Prins 2000: 103) and entered the RMNH after the merger with the ZMA in 2010.

***LophochroaGoffini*** Finsch, 1863: xxiii.

***Cacatuatanimberensis*** Roselaar & Prins, 2000: 104.

= *Cacatuaducorpsii* Pucheran, 1853.

Syntype, ZMA.AVES.141, adult female, skin. Loc.: -. Ex: NAM, died in or before 1871.

Syntype, ZMA.AVES.142, adult, sex unknown, skin. Loc.: -. Ex: NAM, died in or before 1871.

Junior synonym of *Cacatuaducorpsii* Pucheran, 1853.

Van den Hoek Ostende et al. (1997: 108) erroneously listed RMNH.AVES.87994 (adult female, mounted skin, ex Rotterdam Zoo, 08 September 1864) as syntype of *LophochroaGoffini* Finsch, 1863. However, Finsch (1863) based this taxon on two live birds in the zoological garden of NAM which became part of the collection of the ZMA (Roselaar and Prins 2000: 104) and entered the RMNH after the merger with the ZMA in 2010.

***Cacatuaeleonora*** Finsch, 1863: xxi.

= *Cacatuagaleritaeleonora* Finsch, 1863.

Holotype, RMNH.AVES.87980, adult female, mounted skin. Loc.: “Aru”?, [Indonesia]. Ex: NAM, 17.iii.1863.

Holotype by monotypy.

***Kakatoëgaleritaaruensis*** Mathews, 1917: 187.

= *Cacatuagaleritaeleonora* Finsch, 1863.

Syntype, RMNH.AVES.87981, adult female, mounted skin. Loc.: Wammer, Aru Isl., [Indonesia], 15.iii.1864. Leg.: D.S. Hoedt.

Syntype, RMNH.AVES.87982, adult male, mounted skin. Loc.: Wammer, Aru Isl., [Indonesia], 17.iv.1864. Leg.: D.S. Hoedt.

Syntype, RMNH.AVES.87983, adult male, mounted skin. Loc.: Wammer, Aru Isl., [Indonesia], 15.v.1864. Leg.: D.S. Hoedt.

Syntype, RMNH.AVES.87984, adult male, mounted skin. Loc.: Wammer, Aru Isl., [Indonesia], 26.i.1865. Leg.: C.B.H. von Rosenberg.

Syntype, RMNH.AVES.87985, adult female, mounted skin. Loc.: Wokam, Aru Isl., [Indonesia], 01.ii.1865. Leg.: C.B.H. von Rosenberg.

Syntype, RMNH.AVES.87986, adult male, mounted skin. Loc.: Wammer, Aru Isl., [Indonesia], 02.ii.1865. Leg.: C.B.H. von Rosenberg.

Syntype, RMNH.AVES.87987, adult female, mounted skin. Loc.: Wammer, Aru Isl., [Indonesia], 02.ii.1865. Leg.: C.B.H. von Rosenberg, 1866.

Syntype, RMNH.AVES.87988, adult male, mounted skin. Loc.: Wammer, Aru Isl., [Indonesia], 04.ii.1865. Leg.: C.B.H. von Rosenberg.

Syntype, RMNH.AVES.87989, adult male, mounted skin. Loc.: Wammer, Aru Isl., [Indonesia], 06.ii.1865. Leg.: C.B.H. von Rosenberg.

Syntype, RMNH.AVES.87990, adult female, mounted skin. Loc.: Wammer, Aru Isl., [Indonesia], 07.ii.1865. Leg.: C.B.H. von Rosenberg.

Syntype, RMNH.AVES.87991, adult male, mounted skin. Loc.: Wammer, Aru Isl. [Indonesia], 10.ii.1865. Leg.: C.B.H. von Rosenberg.

Syntype, RMNH.AVES.87992, adult female, mounted skin. Loc.: Trangan, Aru Isl. [Indonesia], 07.vii.1865. Leg.: C.B.H.von Rosenberg.

Mathews (1917) based his description on Van Oort (1909a: 70), who referred to 12 specimens in the collection of the RMNH from the Aru Islands which are consistently smaller, with wing lengths between 260–290 mm, compared to *Cacatuagaleritamacrolophu*s.

***Cacatuatriton*** Temminck, 1849: 405.

= *Cacatuagaleritatriton* Temminck, 1849.

Lectotype, RMNH.AVES.87993, adult female, mounted skin. Loc.: Aiduma Island, [Papua], [Indonesia], vii.1828]. Leg.: S. Müller.

Schlegel (1864c: 14) designated the lectotype.

The stand of RMNH.AVES.87993 refers specifically to July (“vii”). Müller visited Aiduma Island between 4 July and 29 August 1828 as part of the ‘Triton’ expedition.

***Cacatuagoffiniana*** Roselaar & Michels, 2004: 186.

= *Cacatuagoffiniana* Roselaar & Michels, 2004.

Holotype, RMNH.AVES.90750 (formerly RMNH.AVES.6620), adult female, skin. Loc.: Saumlaki, Tanimbar, [Indonesia], 22.iv.1923. Leg.: P.F. Kopstein.

Paratype, RMNH.AVES.90751.

Until the publication by Roselaar and Prins (2000), *Lophochroagoffini* Finsch, 1863, was applied to birds from Tanimbar, but the type series in fact represent *Cacatuaducorpsii* Pucheran, 1853 from the Solomon Islands. Finding the Tanimbar Corella nameless, Roselaar and Prins (2000: 104) proposed a substitute name for this taxon, *Cacatuatanimberensis* nom. nov., and designated RMNH.AVES.87994 as the holotype. However, according to Art. 72.7 of “the Code” (ICZN 1999) a replacement name is an objective synonym of the older name and regardless of any additions or restrictions, both names have the same name-bearing type specimens. Roselaar and Michels (2004) recognized and corrected this by describing the Tanimbar Corella under the new name *C.goffiniana* based on newly selected specimens.

***Cacatuapastinatortransfreta*** Mees, 1982: 79.

= *Cacatuasanguineatransfreta* Mees, 1982.

Holotype, RMNH.AVES.42449, adult male, skin. Loc.: Noordpolder, Koerik, [Papua], [Indonesia], 30.vii.1959. Leg.: A. Hoogerwerf, 1960.

Paratypes, RMNH.AVES.42445–42448.

***Plyctolophusparvulus*** Bonaparte, 1850b: 139.

= *Cacatuasulphureaparvula* (Bonaparte, 1850).

Lectotype, RMNH.AVES.87995, adult female, mounted skin. Loc.: Semao, Timor, [Indonesia], [x.1828–xii.1829]. Leg.: S. Müller.

Bonaparte (1850b) gave no indication of the number of specimens available to him, so the listing of RMNH.AVES.87995 as holotype by Van den Hoek Ostende et al. (1997: 121) is erroneous, and constitutes a lectotype designation. Schlegel (1864c: 137) mentioned a specific specimen (cat. 5, collected by Muller on Semao) which was viewed by Bonaparte and used for the species description.

Müller visited Timor from October 1828 until December 1829 during his expedition with the corvette ‘Triton’.

***Cacatuaaequatorialis*** Temminck, 1849: 405.

= *Cacatuasulphureasulphurea* (Gmelin, 1788).

Syntype, RMNH.AVES.87996, adult male, mounted skin. Loc.: Tomini, Celebes [= Sulawesi], [Indonesia], [ix.1841–iv.1842]. Leg.: E.A. Forsten.

Syntype, RMNH.AVES.87997, adult female, mounted skin. Loc.: Tomini, Celebes [= Sulawesi], [Indonesia], [ix.1841–iv.1842]. Leg.: E.A. Forsten.

Van den Hoek Ostende (1997: 118) gave Tomoni [sic] as collecting locality, which is an error for Tomini (Schlegel 1864c: 140). Forsten visited this area between September 1841 and April 1842.

***Psittacusnasicus*** Temminck, 1821: 115.

= *Cacatuatenuirostris* (Kuhl, 1820).

This species was described based on a single specimen collected by Brown in Port Philippe, south coast New Holland (Temminck 1821: 116) which is in the NHM (NHMUK 1863.7.6.3) and listed by Warren (1966: 199) as the holotype.

See Schodde and Mason (1997: 97) for reference to *Psittacusnasicus* Temminck, and Schodde et al. (2010) for nomenclatural issues involved.

***PsittacusLathami*** Temminck, 1807: 21.

***PsittacusTemminckii*** Kuhl, 1820b: 89.

***PsittacusSolandri*** Temminck, 1821: 113.

= *Calyptorhynchuslathamilathami* (Temminck, 1807).

Holotype for *Lathami*, lectotype for *Solandri*, syntype for *Temminckii*, RMNH.AVES.87940, adult male, mounted skin. Loc.: Australia. Ex: Cabinet Temminck.

Jansen (2018: 406) suggested the area near Port Jackson, Botany Bay, New South Wales, as the collecting area, where C.A. Lesueur collected it between 22 August and 13 November 1801.

See Schodde and Bock (1994) for conservation of the name *P.lathami* Temminck, 1807 and suppression of *P.magnificus* Shaw 1790.

Stresemann (1953a: 327) unnecessarily designated the lectotype for *P.Lathami*, which is a holotype by monotypy. Schlegel (1864c: 153) designated the lectotype of *P. Solandri*.

Schifter et al. (2007: 167) stated that two specimens in the NMW collection (NMW 50.025 and 50.027) are syntypes of *P.lathami* Temminck, 1807. However, these specimens are listed by Latham (1790: 107) as “[*Psittacus*] *Banksii* β», not “*Banksii* γ”.= *Lathami* Temminck, 1807.

***Cacatuaintermedia*** Schlegel, 1861: 186.

= *Proboscigeraterrimusaterrimus* (Gmelin, 1788).

Schlegel (1861) referred to specimens collected by Wallace on the Aru Islands. The RMNH has no specimens in the collection.

***PsittacusGoliath*** Kuhl, 1820b: 92.

= *Proboscigeraterrimusgoliath* (Kuhl, 1820).

Syntype, RMNH.AVES.88081, adult, sex unknown, mounted skin. Loc.: New Guinea. Ex: Cabinet Temminck.

***Psittacusalcato*** Temminck, 1828: 74.

***Araalecto*** Temminck, 1835: 17 (amended).

= *Proboscigeraterrimusgoliath* (Kuhl, 1820).

Holotype, RMNH.AVES.88065, adult, sex unknown, skin. Loc.: -. Leg.: -.

Usually Temminck (1835: 17) is given as the authority for this name. However, Temminck already described it in 1828, when he named it *Psittacusalcato*, which he amended to *Araalecto* in 1835.

***Proboscigeraterrimusoorti*** Mathews, 1916: 94.

= *Proboscigeraterrimusgoliath* (Kuhl, 1820).

Syntype, RMNH.AVES.88066, adult female, skin. Loc.: Noordrivier, [Papua], [Indonesia], 18.v.1907. Leg.: Dutch New Guinea Expedition, 1907.

Syntype, RMNH.AVES.88067, adult female, skin. Loc.: Van Weels Kamp, [Papua], [Indonesia], 03.vi.1907. Leg.: Dutch New Guinea Expedition, 1907.

Syntype, RMNH.AVES.88068, adult female, skin. Loc.: Sabang, [Papua], [Indonesia], 20.vii.1907. Leg.: Dutch New Guinea Expedition, 1907.

Syntype, RMNH.AVES.88069, adult female, skin. Loc.: Alkmaar, [Papua], [Indonesia], 12.viii.1907. Leg.: Dutch New Guinea Expedition, 1907.

Mathews (1916) based his description on Van Oort (1909a: 70), who referred to four specimens in the collection of the RMNH. All other specimens listed by Van den Hoek Ostende et al. (1997: 131) are not part of the type series.

***Microglossusaterrimusstenolophus*** van Oort, 1911a: 240.

= *Proboscigeraterrimusstenolophus* (van Oort, 1911).

Syntype, RMNH.AVES.88082 (1609), adult male, skin. Loc.: Humboldt Bay, [Papua], [Indonesia], 23.iii.1903. Leg.: L.F. de Beaufort and H.A. Lorentz, 08.x.1909.

Syntype, RMNH.AVES.88083 (1609), adult female, skin. Loc.: Sentani Lake [Papua], [Indonesia], 28.vi.1903. Leg.: L.F. de Beaufort and H.A. Lorentz, 08.x.1909.

#### ﻿Psittacidae Rafinesque, 1815

***Psittacusroseicollis*** Vieillot, 1818b: 377.

= *Agapornisroseicollisroseicollis* (Vieillot, 1818).

Lectotype, RMNH.AVES.87925, adult, sex unknown, relaxed mount. Loc.: “Pays des Grandes Namaquois”, [South Africa]. Leg.: ?F. Levaillant. Ex: Cabinet Temminck.

Vieillot (1818b) did not mention any specimens. Van den Hoek Ostende et al. (1997: 97) listed RMNH.AVES.87925 as holotype, which is a lectotype designation.

***Psittacuspretrei*** Temminck, 1830: livr. 83, pl. 492.

= *Amazonapretrei* (Temminck, 1830).

Lectotype, RMNH.AVES.87929, adult, sex unknown, mounted skin. Loc.: Mexico, changed to Uruguay. Leg.: -.

Temminck (1830) doubted “Mexico” as the provenance of this specimen. This species originates from southern Brazil. The description gives no indication about the number of specimens nor the whereabouts. Van den Hoek Ostende et al. (1997: 115) listed it as holotype, which constitutes a lectotype designation.

***Chrysotisrhodocorytha*** Salvadori, 1890: 369.

= *Amazonarhodocorytha* (Salvadori, 1890).

Syntype, RMNH.AVES.87928, adult, sex unknown, mounted skin. Loc.: Brazil. Ex: Cabinet Temminck.

Salvadori (1890) introduced *Chrysotisrhodocorytha* as a new name for specimens of *Psittacus Dufresneanus* from Brazil listed in Kuhl, 1820.

***Psittacusauricapillus*** Kuhl, 1820b: 20.

= *Aratingaauricapillusauricapillus* (Kuhl, 1820).

Syntype, RMNH.AVES.87934, adult, sex unknown, mounted skin. Loc.: Brazil. Ex: Cabinet Temminck.

Syntype, RMNH.AVES.87935, adult, sex unknown, mounted skin. Loc.: Brazil. Ex: Cabinet Temminck.

***Psittaculuschrysosema*** “Natterer” Schlegel, 1864c: 28.

= *Brotogerischrysopterachrysosema* (Sclater, 1864).

Holotype, RMNH.AVES.87939, adult female, mounted skin. Loc.: Brazil, 01.x.1829. Leg.: J. Natterer. Ex: NMW, 1864.

According to Von Pelzeln (1871: 261), Natterer collected ten specimens on the Rio Madeira, Cachoeira das Pederneiras, in October 1829.

***Sittacecyanoptera*** “Natterer” Von Pelzeln, 1870: 260.

= *Brotogeriscyanopteracyanoptera* (Pelzeln, 1870).

Syntype, RMNH.AVES.90996, adult male, mounted skin. Loc.: Rio Vaupé [= Rio Uapés], [Brazil], 03.vii.1831. Leg.: J. Natterer. Ex: NMW, 1864.

Syntype, RMNH.AVES.90997, adult female, mounted skin. Loc.: Rio Vaupé [= Rio Uapés], [Brazil], 14.vii.1831. Leg.: J. Natterer. Ex: NMW, 1864.

The type series comprises all specimens available to Von Pelzeln at that time. Although not mentioned in Schifter et al. (2007), four syntypes collected by Natterer near Rio Içanná and Vaupé are in the NMW (NMW 41019–41021, and 84968). According to Natterer’s handwritten register cards the other syntypes went to the RMNH (see specimens listed here), to Hartlaub (male, Rio Vaupé, collected 14.vi.1831), and Salvin (female, Rio Vaupé, collected 1831).

The name *cyanoptera* has long been credited to Salvadori (1891), see e.g., Peters (1937: 207). However, it was already brought into use by Von Pelzeln (1870: 260). Although listed by him in the synonymy of *Brotogerysjugularis* (Deville, 1851), *cyanoptera* was later treated as a valid name and by this became available. It dates from its first publication as a synonym (ICZN 1999, Art. 11.6.1.). It was originally described under the name *Conurusjugularis* Delville, 1851. However, *jugularis* is preoccupied by *Psittacusjugularis* Müller, 1776 and so is *Sittacedevillei* GR Gray, 1870, a new name for *jugularis* Delville, 1851. The next available name is *Sittacecyanoptera* Pelzeln, 1870.

***Psittacusviridissimus*** Kuhl, 1820b: 25.

= *Brotogeristirica* (Gmelin, 1788).

Lectotype, RMNH.AVES.90905, adult, sex unknown, mounted skin. Loc.: Brazil. Ex: Cabinet Temminck.

Schlegel (1864c: 14) designated the lectotype.

***Conurusocularis*** Sclater & Salvin, 1865: 367.

= *Eupsittulapertinaxocularis* (Sclater & Salvin, 1865).

Van den Hoek Ostende et al. (1997: 116) erroneously listed RMNH.AVES.87937 as syntype. Syntypes are in the NHM (Warren 1966: 213).

***Conurusxanthogenius*** Bonaparte, 1850b: 132.

= *Eupsittulapertinaxxanthogenia* (Bonaparte, 1850).

Lectotype, RMNH.AVES.87938, adult, sex unknown, skin. Loc.: [Bonaire], [Antilles], South America. Ex: Cabinet Temminck.

Although Bonaparte (1850b) gave no reference to specimens, we follow Schlegel (1864c: 18) who listed RMNH.AVES.87938 as the type, thus designating it the lectotype.

According to Bonaparte (1850b) this species is from the interior of Brazil, probably an erroneous interpretation of “South America” as on the stand.

***Psittaculacyanochlora*** “Natterer” Schlegel, 1864c: 31.

= *Forpuspasserinuscyanochlorus* (Schlegel, 1864).

Syntype, RMNH.AVES.87960, adult female, mounted skin. Loc.: Forte de São Joaquim do Rio Branco, Brazil, 25.v.1822. Leg.: J. Natterer. Ex: NMW, 1864.

Syntype, RMNH.AVES.87961, adult, sex unknown, mounted skin. Loc.: Forte de São Joaquim do Rio Branco, Brazil, 28.xii.1831. Leg.: J. Natterer. Ex: NMW, 1864.

***Psittacusbrachyurus*** “Temminck” Kuhl, 1820b: 72.

= *Graydidascalusbrachyurus* (Temminck & Kuhl, 1820).

Lectotype, RMNH.AVES.87978, adult, sex unknown, mounted skin. Loc.: [Cayenne]. Ex: Cabinet Temminck.

This publication was authored by Kuhl (1820b), not Temminck & Kuhl as indicated by the IOC. See also *Psittacusbrachyurus* Kuhl, 1820. Kuhl gave no indication of the number of specimens he had seen, so the listing of RMNH.AVES.87978 as holotype by Van den Hoek Ostende et al. (1997: 123) is erroneous but constitutes a lectotype designation.

According to Kuhl this species occurred in Cayenne.

***Brotogeryspanychlorus*** Salvin & Godman, 1883: 211.

= *Nannopsittacapanychlora* (Salvin & Godman, 1883).

Possible syntype, RMNH.AVES.88029, adult female, mounted skin. Loc.: Roraima, British Guyana, 17.xii.1881. Leg.: H. W[hitely]. Ex: G.A. Frank, ii.1883.

Possible syntype, RMNH.AVES.88030, adult male, mounted skin. Loc.: British Guyana. Ex: G.A. Frank, ix.1883.

RMNH.AVES.88029 gives “H.W.” as collector and on the stand is written “*Psittacus* n. sp.”. Whitely is not mentioned on the stand of RMNH.AVES.88030, but it says: “*Brotogeryspanychlorus* n. sp. Salvin”. Whitely travelled and collected in and near Roraima late 1881. It is unknown from which source Frank acquired these specimens and whether they have been seen by Salvin and Godman. Hence we refer to them here as possible syntypes.

Other syntypes are in the NHM (Warren 1966: 220).

***Psittacusleucogaster*** “Illiger” Kuhl, 1820b: 70.

= *Pionitesleucogasterleucogaster* (Kuhl, 1820).

Syntype, RMNH.AVES.88056, adult, sex unknown, mounted skin. Loc.: Cayenne. Ex: Cabinet Temminck.

One syntype was sent to the MfN in December 1818.

***Psittacusmitratus*** Wied, 1820: 260.

= *Pionopsittapileata* (Scopoli, 1769).

Syntype, RMNH.AVES.88057, adult male, mounted skin. Loc.: [Morro d’Arara], Brazil, [1815–1817]. Leg.: A.P.M. zu Wied-Neuwied.

Syntype, RMNH.AVES.88058, adult female, mounted skin. Loc.: [Morro d’Arara], Brazil, [1815–1817]. Leg.: A.P.M. zu Wied-Neuwied.

According to Greenway (1973: 86) two other syntypes are in the AMNH (AMNH 6316 and 6317).

***PsittacusMaximiliani*** Kuhl, 1820b: 72.

= *Pionusmaximilianimaximiliani* (Kuhl, 1820).

Syntype, RMNH.AVES.88059, adult, sex unknown, mounted skin. Loc.: Guyana. Ex: Cabinet Temminck.

According to Kuhl (1820b), other syntypes are in the MNHN, MfN, and in Wied’s private collection.

***Psittacusflavirostris*** Spix, 1824: 42, pl. 31, fig. 2.

= *Pionusmaximilianimaximiliani* (Kuhl, 1820).

Possible syntype, RMNH.AVES.209232, adult male, mounted skin. Loc.: [Piauhy, Maitaca], Brazil [1817–1820]. Leg.: J.B. von Spix.

This could also be a syntype of *Psittacusmaximiliani* Kuhl, 1820 (see entry above) if already in Temminck’s cabinet prior to 1820. Because of a lack of information, we do not list this specimen as such.

***Pionusflavifrons*** Rüppell, 1842: 126.

= *Poicephalusflavifrons* (Rüppell, 1842).

Possible syntype, RMNH.AVES.90929, adult, sex unknown, mounted skin. Loc.: Shoa, Abyssinia. Leg.: W.P.E.S. Rüppell.

Steinheimer (2005a: 242) listed the specimen in the RMNH as an arguable syntype. Another syntype is in the NHM (Warren 1966: 98; NHMUK 1845.6.2.6) and in the SMF. See Steinheimer (2005a: 242) for correction of the publication date and treatment of the lectotype designation by Steinbacher (1949: 119), which was incorrect and should be ignored.

***Pioniascitrinocapillus*** Finsch, 1868: 484.

= *Poicephalusflavifrons* (Rüppell, 1842).

Syntype, RMNH.AVES.88061, adult male, mounted skin. Loc.: Takapé [= Tekezé River], Abyssinie [Ethiopia], 04.i.1862. Leg.: Th. von Heuglin.

***PsittacusMeyeri*** Cretzschmar, 1827: 18, pl. 11.

= *Poicephalusmeyerimeyeri* (Cretzschmar, 1827).

Possible syntype, RMNH.AVES.150537, adult male, mounted skin. Loc.: Abyssinia. Leg.: W.P.E.S. Rüppell

Possible syntype, RMNH.AVES.150538, adult female, mounted skin. Loc.: Abyssinia. Leg.: W.P.E.S. Rüppell.

Steinheimer (2005a: 242) listed both as possible syntypes.

***PoiocephalusVersteri*** “Goffin” Finsch, 1863: xvi.

= *Poicephalussenegalusversteri* Finsch, 1863.

Holotype, RMNH.AVES.88062, adult male, mounted skin. Loc.: [Guinea], West-Afrika. Ex: NAM, 01.vii.1864.

Holotype by monotypy. Finsch (1863) based this taxon on a single living bird in the zoological garden of NAM.

***Sittaceprimoli*** Bonaparte, 1853: 807.

= *Primoliusauricollis* (Cassin, 1853).

Lectotype, RMNH.AVES.87931, adult, sex unknown, mounted skin. Loc.: Bolivia. Leg.: A. d’Orbigny.

Bonaparte (1853) based his description on specimens in the collections of the MNHN and RMNH. Schlegel (1864c: 7) designated the lectotype. The syntype (MNHN C.G.1846-717) listed by Voisin and Voisin (2008a: 474) therefore becomes a paralectotype.

***PsittacusIlligeri*** “Temminck” Kuhl, 1820: 19.

= *Primoliusmaracana* (Vieillot, 1816).

Syntype, RMNH.AVES.87932, adult, sex unknown, mounted skin. Loc.: Brazil. Leg.: -.

Syntype, RMNH.AVES.87933, adult, sex unknown, mounted skin. Loc.: Brazil. Leg.: -.

***Araraaymara*** d’Orbigny, 1841: 376.

***Psittacusmurinoides*** “Temminck” Schlegel, 1864c: 16.

***Psittacusaguava*** Schlegel, 1864a: 81.

= *Psilopsiagonaymara* (d’Orbigny, 1839) (error for 1841).

Syntype for *aymara* and *aguava*, holotype for *murinoides*, RMNH.AVES.87930, adult, sex unknown, mounted skin. Loc.: Chile, changed to Bolivia. Leg.: A. d’Orbigny.

Two other syntypes collected by d’Orbigny in Bolivia are in the MNHN (MNHN 2004-111 and 2004-112; Voisin and Voisin 2008a: 476).

Bonaparte (1854e: 150) published Temminck’s manuscript name *Psittacusmurinoides* as a nomen nudum, as did Gray later (1859: 43). Schlegel (1864c: 16) listed *murinoides* in the synonymy of *aymara* d’Orbigny, 1839; therefore *Psittacusmurinoides* is not an available name.

Dickinson (2019) corrected the year of publication from 1839 to 1841.

***Conurusholochlorus*** Sclater, 1859: 224.

= *Psittacaraholochlorus* (Sclater, 1859).

Syntype, RMNH.AVES.87936, adult, sex unknown, mounted skin. Loc.: Jalapa, Mexico. Leg.: [R. Montes d’Oca]. Ex: P.L. Sclater, 1864.

This is one of four syntypes; Warren (1966: 130) listed a syntype in the NHM (NHMUK 1890.6.1.1935).

***Psittacusvulturinus*** “Illiger” Kuhl, 1820b: 62.

= *Pyriliavulturina* (Kuhl, 1820).

Syntype, RMNH.AVES.87979, adult male, mounted skin. Loc.: South Brazil. Leg.: -.

A specimen was sent by Temminck to the MNHN in May 1844.

***Psittacuscruentatus*** Wied, 1820: 70.

= *Pyrrhuracruentata* (Wied, 1820).

Lectotype, RMNH.AVES.88093, adult, sex unknown, mounted skin. Loc.: Brazil. Leg.: A.P.M. zu Wied-Neuwied.

RMNH.AVES.88094 listed in Van den Hoek Ostende et al. (1997: 133) as syntype is no longer considered a type due to lack of information about the collector. Schlegel (1864c: 23) designated the lectotype. Another paralectotype is in the MLC (MLC.2011.0.1195). No type was found in the AMNH (Allen 1889: 263).

***Conurushoffmanni*** Cabanis, 1861: 6.

= *Pyrrhurahoffmannihoffmanni* (Cabanis, 1861).

Syntype, RMNH.AVES.88095, adult, sex unknown, mounted skin. Loc.: Costa Rica. Ex: MfN, 1864.

***Psittacusleucotis*** “Lichtenstein” Kuhl, 1820b: 21.

***Psittacuslepidus*** “Illiger” Kuhl, 1820b: 21.

= *Pyrrhuraleucotis* (Kuhl, 1820).

Syntype, RMNH.AVES.88096, adult, sex unknown, mounted skin. Loc.: Brazil. Ex: Cabinet Temminck.

Kuhl (1820b) published *lepidus* in the synonymy of his *Psittacusleucotis*. One specimen was exchanged with the MfN in July 1818.

***Conurusphoenicuru****s* “Natterer” Schlegel, 1864c: 26.

= *Pyrrhuramolinaephoenicura* (Schlegel, 1864).

Syntype, RMNH.AVES.88097, adult female, mounted skin. Loc.: [Jauru], Brazil, 03.vii.1826. Leg.: J. Natterer. Ex: NMW, 1864.

Syntype, RMNH.AVES.88098, adult male, mounted skin. Loc.: Mato Grosso, Brazil, x.1825. Leg.: J. Natterer. Ex: NMW, 1864.

RMNH.AVES.88097 is one of the eight specimens Natterer received from captain Peixoto collected on the road from Registo do Jauro to Matogrosso in July 1826 (Von Pelzeln 1870: 260).

***Conurusrhodogaster*** “Natterer” Schlegel, 1864: 27.

= *Pyrrhuraperlata* (Spix, 1824).

Holotype, RMNH.AVES.88099, adult female, mounted skin. Loc.: [Borba], Brazil, 09.xii.1829. Leg.: J. Natterer. Ex: NMW.

Holotype by monotypy.

***Psittacusmicropterus*** Kuhl, 1820b: 67.

= *Touitbatavicus* (Boddaert, 1783).

According to Schlegel (1864c: 69) a specimen in the RMNH (RMNH.AVES.209010) is the type for *P.micropterus* Kuhl, 1820. However, Kuhl (1820b) based his description on pl. 41 in Sonnerat (1776) and did not refer to any specimen(s) or collection(s).

***Psittacushuetii*** Temminck, 1830: livr. 83, pl. 491.

= *Touithuetii* (Temminck, 1830).

Lectotype, RMNH.AVES.88113, adult, sex unknown, mounted skin. Loc.: Peru. Leg.: -.

Schlegel (1864c: 69) designated the lectotype. The description gives neither indication about the number of type specimens nor about their whereabouts.

***Psittacussurdus*** Kuhl, 1820b: 59.

***Psittacusochrurus*** “Prins Maximil.” [= Wied] Kuhl, 1820b: 59.

= *Touitsurdus* (Kuhl, 1820).

Syntype, RMNH.AVES.88114, adult male, mounted skin. Loc.: Brazil. Ex: Cabinet Temminck.

Syntype, RMNH.AVES.88115, adult female, mounted skin. Loc.: Brazil. Ex: Cabinet Temminck.

Kuhl (1820b) published *ochrurus* in the synonymy of his *Psittacussurdus*.

#### ﻿Psittaculidae

**Psittacus (Platycercus) hypophonius** Müller, 1843: 181.

= *Alisterusamboinensishypophonius* (Müller, 1843).

Syntype, RMNH.AVES.87926, adult male, skin. Loc.: Dodingo, Halmahera, [Indonesia]. Leg.: E.A. Forsten.

Syntype, RMNH.AVES.87927, adult male, skin. Loc.: Dodingo, Halmahera, [Indonesia]. Leg.: E.A. Forsten.

During a forced stay due to illness on Ternate from 19 June 1841 until mid September 1841, Forsten sent his hunters to Halmahera to collect skins.

***Chalcopsittaspectabilis*** van Oort, 1908b: 127.

= *Chalcopsittaatrainsignis* Oustalet, 1878.

Holotype, RMNH.AVES.87941, adult male, skin. Loc.: Mamberamo, [Papua], [Indonesia], 13.iii.1876. Leg.: L. Laglaize. Ex: A.A. Bruijn, 1878.

Holotype by monotypy.

***Psittacussintillatus*** Temminck, 1835: livr. 96, pl. 569.

= *Chalcopsittascintillatascintillata* (Temminck, 1835).

Lectotype, RMNH.AVES.87942, immature, relaxed mount. Loc.: Lobo Bay, [Papua], [Indonesia], [vii–viii.1828]. Leg.: S. Müller.

Schlegel (1864c: 122) designated the lectotype.

***CharmosynaStellae*** A.B. Meyer in: Finsch & Meyer, 1886: 1, pl. 2.

= *Charmosynapapoustellae* A.B. Meyer, 1886.

Syntype, RMNH.AVES.87943, adult male, skin. Loc.: Owen Stanley Mountains, Papua New Guinea. Leg.: [K. Huhnstein, 1884]. Ex: O. Finsch, x.1886.

Syntype, RMNH.AVES.87944, immature, skin. Loc.: Owen Stanley Mountains, Papua New Guinea. Leg.: [K. Huhnstein, 1884]. Ex: O. Finsch, x.1886.

Another syntype is in the NMW (NMW 49978; Schifter et al. 2007: 164–165) and two syntypes had been in the MTD but were lost during WWII (MTD C8167 and C8168; Eck and Quaisser 2004: 251).

***Psittacusplacentis*** Temminck, 1835: livr. 93, pl. 553.

= *Charmosynaplacentisplacentis* (Temminck, 1835).

Syntype, RMNH.AVES.87945, adult male, skin. Loc.: Utanata River, [Papua], [Indonesia], [11–22.vi.1828]. Leg.: S. Müller.

Syntype, RMNH.AVES.87946, adult female, skin. Loc.: Utanata River, [Papua], [Indonesia], [11–22.vi.1828]. Leg.: S. Müller.

Syntype, RMNH.AVES.87947, immature male, skin. Loc.: Utanata River, [Papua], [Indonesia], [11–22.vi.1828]. Leg.: S. Müller.

See Mees (1982) for the year of publication.

***Coracopsisbarklyi*** Newton, 1867: 346.

= *Coracopsisbarklyi* Newton, 1867.

Possible syntype, ZMA.AVES.932, adult male, skin. Loc.: Praslin Island, [Seychelles]. Ex: G.A. Frank Jr, London [in or before 1871].

See Roselaar and Prins (2000: 105).

***Coracopsismelanorhyncha*** Finsch, 1863: xx [20].

= *Coracopsis cf vasavasa* (Shaw, 1812).

Finsch (1863) based this new taxon on two live birds in the zoological garden of NAM. The RMNH received a skeleton (RMNH.AVES.253904) from NAM in 1897 that could have been the remains of one of the two living birds Finsch referred to. See Roselaar and Prins (2000: 105) for a discussion on the type specimens and the possible identification as *C.vasavasa* (Shaw, 1812).

***Psittaculadiophthalmaaruensis*** Schlegel, 1874a: 33.

= *Cyclopsittadiophthalmaaruensis* (Schlegel,1874).

Syntype, RMNH.AVES.88035, adult female, skin. Loc.: Wammer, Aru Islands, [Indonesia], 26.iv.1864. Leg.: D.S. Hoedt.

Syntype, RMNH.AVES.88036, adult male, skin. Loc.: Wammer, Aru Islands, [Indonesia], 27.iv.1864. Leg.: D.S. Hoedt.

Syntype, RMNH.AVES.88037, adult male, skin. Loc.: Wammer, Aru Islands, [Indonesia], 28.i.1865. Leg.: C.B.H. von Rosenberg.

Syntype, RMNH.AVES.88038, adult male, skin. Loc.: Wammer, Aru Islands, [Indonesia], 01.ii.1865. Leg.: C.B.H. von Rosenberg.

Syntype, RMNH.AVES.88039, adult male, skin. Loc.: Wammer, Aru Islands, [Indonesia], 09.ii.1865. Leg.: C.B.H. von Rosenberg.

Syntype, RMNH.AVES.88040, adult female, skin. Loc.: Wammer, Aru Islands, [Indonesia], 17.ii.1865. Leg.: C.B.H. von Rosenberg.

Syntype, RMNH.AVES.88041, adult female, skin. Loc.: Wonumbai [= Sungai Manumbai], Aru Islands, [Indonesia], 15.v.1865. Leg.: C.B.H. von Rosenberg.

Syntype, RMNH.AVES.88042, adult female, skin. Loc.: Wonumbai [= Sungai Manumbai], Aru Islands, [Indonesia], 16.v.1865. Leg.: C.B.H. von Rosenberg.

Syntype, RMNH.AVES.88043, adult male, skin. Loc.: Wonumbai [= Sungai Manumbai], Aru Islands, [Indonesia], 21.v.1865. Leg.: C.B.H. von Rosenberg.

Syntype, RMNH.AVES.88044, adult female, skin. Loc.: Wonumbai [= Sungai Manumbai], Aru Islands, [Indonesia], 21.v.1865. Leg.: C.B.H. von Rosenberg.

Syntype, RMNH.AVES.88045, adult male, skin. Loc.: Wonumbai [= Sungai Manumbai], Aru Islands, [Indonesia], 26.vi.1865. Leg.: C.B.H. von Rosenberg.

***Psittaculagulielmi III*** Schlegel, 1866c: 252.

= *Cyclopsittagulielmitertiigulielmitertii* (Schlegel, 1866).

Syntype, RMNH.AVES.88046, adult female, skin. Loc.: Sorong, [Papua], [Indonesia], 25.xi.1864. Leg.: H.A. Bernstein.

Syntype, RMNH.AVES.88047, adult female, skin. Loc.: Sorong, [Papua], [Indonesia], 25.xi.1864. Leg.: H.A. Bernstein.

Syntype, RMNH.AVES.88048, adult male, skin. Loc.: Kalwal, Salawatti, [Indonesia], 05.iii.1865. Leg.: H.A. Bernstein.

Syntype, RMNH.AVES.88049, adult female, skin. Loc.: Kalwal, Salawatti, [Indonesia], 08.iii.1865. Leg.: H.A. Bernstein.

***Psittaculamelanogenia*** Von Rosenberg, 1866: 142.

= *Cyclopsittagulielmitertiimelanogenia* (von Rosenberg, 1866).

Syntype, RMNH.AVES.88050, adult male, skin. Loc.: Wokam, Aru Islands, [Indonesia], 14.iii.1865. Leg.: C.B.H. von Rosenberg (no. 321).

Syntype, RMNH.AVES.88051, adult male, skin. Loc.: Wonumbai [= Sungai Manumbai], Aru Islands, [Indonesia], v.1865. Leg.: C.B.H. von Rosenberg.

Syntype, RMNH.AVES.88052, adult female, skin. Loc.: Wonumbai [= Sungai Manumbai], Aru Islands, [Indonesia], 17.v.1865. Leg.: C.B.H. von Rosenberg (no. 741).

Syntype, RMNH.AVES.88053, adult female, skin. Loc.: Wonumbai [= Sungai Manumbai], Aru Islands, [Indonesia], 24.v.1865. Leg.: C.B.H. von Rosenberg (no. 824).

Syntype, RMNH.AVES.88054, adult female, skin. Loc.: Maikoor, Aru Islands, [Indonesia], 19.vii.1865. Leg.: C.B.H. von Rosenberg (no. 1198).

***EclectusCornelia*** Bonaparte, 1850b: 135.

= *Eclectusroratuscornelia* Bonaparte, 1850.

Possible holotype, ZMA.AVES.261, adult female, relaxed mount. Loc.: -. Ex: NAM.

The description was based on a single bird living in the zoological garden of NAM. However, the type status is uncertain since the original information is lost (see Roselaar and Prins 2000: 102).

***Lariusroratusbiaki*** Hartert, 1932: 448.

= *Eclectusroratuspolychloros* (Scopoli, 1786).

Holotype, RMNH.AVES.14032, adult male, skin. Loc.: Biak, [Indonesia], 1915. Leg.: W.K.H. Feuilleteau de Bruijn. Ex: MZB, 12.i.1950 (MZB 13366).

Holotype by monotypy.

***Psittacodisintermedius*** Bonaparte, 1850a: 4.

= *Eclectusroratusroratus* (P.L.S. Müller, 1776).

Lectotype, RMNH.AVES.88012, adult [male], mounted skin. Loc.: Amboine [= Ambon], [Indonesia]. Ex: NAM.

Schlegel (1864c: 41) designated the lectotype. Bonaparte (1850a) gave no indication of the number of specimens available to him, so the listing of RMNH.AVES.88012 as holotype by Van den Hoek Ostende et al. (1997: 125) was an error.

***Psittacodiswestermani*** Bonaparte, 1850a: 4.

= *Eclectusroratuswestermani* (Bonaparte, 1850).

Lectotype, RMNH.AVES.88013, adult male, mounted skin. Loc.: -. Ex: NAM, 09.xii.1858.

Paralectotype, ZMA.AVES.280.

Bonaparte (1850a) described this species based on living birds in the zoological garden of NAM. It is known that the zoo possessed four specimens around that period, but according to Roselaar and Prins (2000: 102) it is not certain which of the two remaining skins were seen by Bonaparte.

Schlegel (1864c: 42) designated the lectotype. The listing of RMNH.AVES.88013 as holotype by Van den Hoek Ostende et al. (1997: 125) and ZMA.AVES.280 as syntype by Roselaar and Prins (2000: 102) were errors.

According to Peters (1937: 231) this taxon might represent an arrested plumage development of *Loriusroratusriedeli* (A.B. Meyer, 1882). Roselaar and Prins (2000: 103) demonstrated that it represents a separate taxon which has become extinct according to the IOC 10.1 world list.

***Psittacus (Eos) Bernsteini*** Von Rosenberg, 1863: 145.

***DomicellaSchlegeli*** Finsch, 1868: 792 (nomen novum).

= *Eosborneabornea* (Linnaeus, 1758).

Syntype, RMNH.AVES.87955, adult male, mounted skin. Loc.: Great Key Island, [Indonesia], 24.vi.1863. Leg.: D.S. Hoedt.

Syntype, RMNH.AVES.89441, adult female, mounted skin. Loc.: Great Key Island, [Indonesia], 24.vi.1863. Leg.: D.S. Hoedt.

Rosenberg was based in Ambon at the time of writing, together with Hoedt. He did not refer to any specimens, neither in collections nor from his own observations, so must have seen the specimens collected by Hoedt.

Finsch (1868) introduced *DomicellaSchlegeli* as a nomen novum for *PsittacusBernsteini* Rosenberg, 1863, so the type series for *Bernsteini* also applies to *Schlegeli*.

***Eoscyanogenia*** Bonaparte, 1850b: 135.

= *Eoscyanogenia* Bonaparte, 1850.

Lectotype, RMNH.AVES.87956, adult male, relaxed mount. Loc.: -. Ex: NAM.

This specimen, which died in captivity in the zoological garden of NAM, originated from the Moluccas according to Bonaparte (1850b). However, this species is only known from islands in Cenderawasih Bay, West Papua. Schlegel (1864c: 129) listed it as type, hence selecting it as lectotype.

Voisin and Voisin (2008a: 466) referred to a paralectotype in the MNHN (MNHN C.G.2004-127) collected by Dumont d`Urville in Manokwari, Astrolabe Bay, between 26 August and 6 September 1827. Based on Bonaparte’s description there is no reason to assume that any specimens in the MNHN were involved in Bonaparte’s description.

***Psittacusreticulatus*** Müller, 1841: 107.

= *Eosreticulata* (Müller, 1841).

Lectotype, RMNH.AVES.87957, adult, sex unknown, relaxed mount. Loc.: Timorlaoet [= Tanimbar], Amboine [= Ambon], [Indonesia], [29.iii–20.iv.1828]. Leg.: S. Müller.

Müller (1841) gave no indication of the number of specimens available to him, so the listing of RMNH.AVES.87957 as holotype by Van den Hoek Ostende et al. (1997: 121) is erroneous but constitutes a lectotype designation.

*Eosreticulata* is confined to Tanimbar. Ambon probably refers to the locality where Müller, who never visited Tanimbar, obtained this specimen and where he stayed from 29 March to 20 April 1828. The locality Timorlaout (= Tanimbar) could have been added to the label later.

***Eossemilarvata*** Bonaparte, 1850b: 135.

= *Eossemilarvata* Bonaparte, 1850.

Lectotype, RMNH.AVES.87958, adult, sex unknown, mounted skin. Loc.: -. Ex: NAM.

In his original description, Bonaparte (1850b) gave no indication of the number of specimens available to him, but in a publication later that year (1850e: 28) he mentioned having only a single specimen and referred to it as the “typical specimen”, which constitutes a lectotype designation. The listing of RMNH.AVES.87958 as holotype by Van den Hoek Ostende et al. (1997: 121) was erroneous.

***Lorius (Eos) Wallacei*** Finsch, 1864: 411.

= *Eossquamatasquamata* (Boddaert, 1783).

Syntype, RMNH.AVES.87959, adult, sex unknown, mounted skin. Loc.: Waigeu, [Indonesia], [vii–ix.1860]. Leg.: A.R. Wallace. Ex: G.A. Frank, 1862.

Finsch (1864) did not mention the number of syntypes, but referred to specimens in RMNH, NHM, and in the collection of Wallace. However, when he reconsidered and withdrew the name in a later publication (Finsch 1868: 396), he mentioned that he had three specimens: two in the Wallace collection and one in the RMNH. Warren (1966: 309–310) listed no syntypes in the NHM.

Wallace visited Waigeo from July until September 1860 (Baker 2001: 285).

***Psittacuscyanicollis*** Müller, 1841: 108.

= *Geoffroyusgeoffroyicyanicollis* Müller, 1841.

In his description Müller (1841) referred to a single female from Manado collected by Reinwardt. This specimen is not present in the RMNH and not listed by Schlegel (1864c; 1874a) and might therefore be the specimen that was sent to the MNHN in May 1844.

***GeoffroyusLansbergei*** Finsch, 1899a: 225.

= *Geoffroyusgeoffroyifloresianus* Salvadori, 1891.

Holotype, RMNH.AVES.87977, immature, skin. Loc.: Sumbawa, [Indonesia], x.1879. Leg.: J.W. van Lansberge, 1882.

Holotype by monotypy. Finsch (1899a) based his description on a single specimen.

***Pioniasobiensis*** Finsch, 1868: 389.

= *Geoffroyusgeoffroyiobiensis* (Finsch, 1868).

Syntype, RMNH.AVES.87962, adult female, mounted skin. Loc.: Obi-Latoe [= Obilatu], [Indonesia], 23.viii.1862. Leg.: H.A. Bernstein.

Syntype, RMNH.AVES.87963, adult male, mounted skin. Loc.: Obi-Latoe [= Obilatu], [Indonesia], 24.viii.1862. Leg.: H.A. Bernstein.

Syntype, RMNH.AVES.87964, adult male, mounted skin. Loc.: Obi-Latoe [= Obilatu], [Indonesia], 24.viii.1862. Leg.: H.A. Bernstein.

Syntype, RMNH.AVES.87965, adult male, mounted skin. Loc.: Obi-Latoe [= Obilatu], [Indonesia], 24.viii.1862. Leg.: H.A. Bernstein.

Syntype, RMNH.AVES.87966, adult male, mounted skin. Loc.: Obi-Latoe [= Obilatu], [Indonesia], 24.viii.1862. Leg.: H.A. Bernstein.

***Eclectusrhodops*** Schlegel, 1864c: 43.

***Geoffroyusschlegelii*** Salvadori, 1877a: 29 (nomen novum).

= *Geoffroyusgeoffroyirhodops* (Schlegel, 1864).

Syntype, RMNH.AVES.87967, adult male, mounted skin. Loc.: Amboine [= Ambon], [Indonesia], [29.iii–20.iv..1828]. Leg.: S. Müller.

Syntype, RMNH.AVES.87968, adult female, mounted skin. Loc.: Amboine [= Ambon], [Indonesia], [29.iii–20.iv..1828]. Leg.: S. Müller.

Syntype, RMNH.AVES.87969, adult male, mounted skin. Loc.: Amboine [= Ambon], [Indonesia], [1842]. Leg.: E.A. Forsten.

Syntype, RMNH.AVES.87970, adult male, mounted skin. Loc.: Amboine [= Ambon], [Indonesia], 1863. Leg.: D.S. Hoedt.

Syntype, RMNH.AVES.87971, adult male, mounted skin. Loc.: Amboine [= Ambon], [Indonesia], 1863. Leg.: D.S. Hoedt.

Syntype, RMNH.AVES.87972, immature male, mounted skin. Loc.: Amboine [= Ambon], [Indonesia], 1863. Leg.: D.S. Hoedt.

Syntype, RMNH.AVES.87973, immature male, mounted skin. Loc.: Amboine [= Ambon], [Indonesia], 1863. Leg.: D.S. Hoedt.

Syntype, RMNH.AVES.87974, immature male, mounted skin. Loc.: Amboine [= Ambon], [Indonesia], 1863. Leg.: D.S. Hoedt.

Syntype, RMNH.AVES.87975, adult female, mounted skin. Loc.: Amboine [= Ambon], [Indonesia], 1863. Leg.: D.S. Hoedt.

Syntype, RMNH.AVES.87976, adult female, mounted skin. Loc.: Amboine [= Ambon], [Indonesia], 1863. Leg.: D.S. Hoedt.

Syntype, RMNH.AVES.89425, adult male, mounted skin. Loc.: Wahai, Ceram, [Indonesia], 1862. Leg.: J.C.B. Bernelot Moens.

Syntype, RMNH.AVES.89426, adult female, mounted skin. Loc.: Wahai, Ceram, [Indonesia], 1862. Leg.: J.C.B. Bernelot Moens.

Syntype, RMNH.AVES.89427, adult female, mounted skin. Loc.: Wahai, Ceram, [Indonesia], 1862. Leg.: J.C.B. Bernelot Moens.

Syntype, RMNH.AVES.89428, adult male, mounted skin. Loc.: Buru, [Indonesia], 1863. Leg.: D.S. Hoedt.

Syntype, RMNH.AVES.89429, adult male, mounted skin. Loc.: Buru, [Indonesia], 1863. Leg.: D.S. Hoedt.

Syntype, RMNH.AVES.89430, adult female, mounted skin. Loc.: Buru, [Indonesia], 12.viii.1863. Leg.: D.S. Hoedt.

Müller visited Ambon from 29 March to 20 April 1828. Forsten visited Ambon in 1842.

Salvadori (1877a) introduced *Geoffroyusschlegelii* as an unnecessary nomen novum for *Eclectusrhodops* Schlegel, 1864.

***Loriculusaurantiifrons*** Schlegel, 1871a: 9.

= *Loriculusaurantiifronsaurantiifrons* Schlegel, 1871.

Syntype, RMNH.AVES.88002, adult female, mounted skin. Loc.: Kasim, Misol, [Indonesia], 01.vi.1867. Leg.: D.S. Hoedt.

Syntype, RMNH.AVES.88003, adult male, mounted skin. Loc.: Waaigama, Misol, [Indonesia], 28.vi.1867. Leg.: D.S. Hoedt.

***Loriculuscatamene*** Schlegel, 1871a: 7.

= *Loriculuscatamene* Schlegel, 1871.

Holotype, RMNH.AVES.87998, adult male, mounted skin. Loc.: Sanghir, [Indonesia], 29.xi.1865. Leg.: D.S. Hoedt.

Holotype by monotypy. Schlegel (1871a) based his description on a single specimen.

***Loriculusexilis*** Schlegel, 1866b: 185.

= *Loriculusexilis* Schlegel, 1866.

Syntype, RMNH.AVES.88004, adult male, mounted skin. Loc.: Tulabello, Celebes [= Sulawesi], [Indonesia], 09.v.1864. Leg.: C.B.H. von Rosenberg.

Syntype, RMNH.AVES.88005, adult female, mounted skin. Loc.: Tulabello, Celebes [= Sulawesi], [Indonesia], 20.v.1864. Leg.: C.B.H. von Rosenberg.

Syntype, RMNH.AVES.88006, adult male, mounted skin. Loc.: Tagouat, Celebes [= Sulawesi], [Indonesia], 17.vii.1864. Leg.: C.B.H. von Rosenberg.

Syntype, RMNH.AVES.88007, immature male, mounted skin. Loc.: Tulabello, Celebes [= Sulawesi], [Indonesia], 09.v.1864. Leg.: C.B.H. von Rosenberg.

According to the label, RMNH.AVES.88007 was collected on 9 April 1864. The collecting date on the stand is given as 9 May 1864. Von Rosenberg stayed in Tulabello from 14 April to 20 May 1864.

***Loriculussclateriruber*** Meyer & Wiglesworth, 1896: 2.

= *Loriculussclateriruber* Meyer & Wiglesworth, 1896.

Syntype, RMNH.AVES.87999, adult, sex unknown, skin. Loc.: Banggai Island, [Indonesia], v–viii.1895. Leg.: C.W. Cursham. Ex: MTD, 14.ii.1896.

Syntype, RMNH.AVES.88000, adult, sex unknown, skin. Loc.: Banggai Island, [Indonesia], v–viii.1895. Leg.: C.W. Cursham. Ex: MTD, 14.ii.1896.

The entire type series was originally held by the MTD (Eck and Quaisser 2004: 255). Five syntypes collected by C.W. Cursham on Banggai and Peleng are still there (MTD C14638, C14639, C14641, C14518, C14521), one was lost during WWII (MTD C14637), another one went in exchange to the MCZ, Cambridge, Massachusetts (MCZ 9734, formerly MTD C14640) and two specimens collected on Peleng went to Sarasin (MTD C14519) and to the Bureau of Science in Manila (MTD C14520).

***Loriculussclateri*** Wallace, 1863a: 336.

= *Loriculussclaterisclateri* Wallace, 1863.

Syntype, RMNH.AVES.88001, adult male, mounted skin. Loc.: Sula Islands, [Indonesia]. Leg.: A.R. Wallace. Ex: G.A. Frank, 1863.

Another syntype is in the NHM (Warren 1966: 265; NHMUK 1873.5.12.1549).

**Psittacus (Psittacula) stigmatus** Müller, 1843: 182.

= *Loriculusstigmatusstigmatus* (Müller, 1843).

Syntype, RMNH.AVES.88008, adult male, mounted skin. Loc.: Gorontalo, Celebes [= Sulawesi], [Indonesia]. Leg.: E.A. Forsten.

Syntype, RMNH.AVES.88009, adult male, mounted skin. Loc.: Tondano, Celebes [= Sulawesi], [Indonesia]. Leg.: E.A. Forsten.

Syntype, RMNH.AVES.88010, adult male, mounted skin. Loc.: Tondano, Celebes [= Sulawesi], [Indonesia]. Leg.: E.A. Forsten.

Syntype, RMNH.AVES.88011, adult female, mounted skin. Loc.: Gorontalo, Celebes [= Sulawesi], [Indonesia], x.1841. Leg.: E.A. Forsten.

Forsten visited the Tondano area from 22 March to 19 June 1841 and the area near Gorontalo between September 1841 and April 1842.

***Domicellagarrulamorotaiana*** van Bemmel, 1940: 333.

= *Loriusgarrulusmorotaianus* (van Bemmel, 1940).

Holotype, RMNH.AVES.14031, adult male, skin. Loc.: Wajaboela, Morotai, [Indonesia], 10.iii.1938. Leg.: G.A.L. de Haan. Ex: MZB, 12.i.1950 (MZB 13247).

Van Bemmel (1940) nominated RMNH.AVES.14031 the type, but later in the article listed another two “co-types”.

***Psittacuscyanauchen*** Müller, 1841: 107.

= *Loriuslorycyanauchen* (Müller, 1841).

Lectotype, RMNH.AVES.87948, adult male, relaxed mount. Loc.: -. Leg.: -.

Müller (1841) gave no indication of the number of specimens available to him, so the listing as holotype by Van den Hoek Ostende et al. (1997: 120) was an error. Schlegel (1864c: 119) designated the lectotype.

The label does not indicate the locality. The species was described by Müller as originating from the Moluccas. In fact, *L.l.cyanauchen* is restricted to Biak Island, Indonesia.

***Loriuscyanauchenviridicrissalis*** de Beaufort, 1909: 393.

= *Loriusloryviridicrissalis* de Beaufort, 1909.

Syntype, RMNH.AVES.87949 (1609), adult male, skin. Loc.: Tami River, [Papua], [Indonesia], iv–vi.1903. Leg.: L.F. de Beaufort and H.A. Lorentz, 08.x.1909.

Syntype, RMNH.AVES.87950 (1609), adult female, skin. Loc.: Sentani Lake, [Papua], [Indonesia], 01.iv.1903. Leg.: L.F. de Beaufort and H.A. Lorentz, 08.x.1909.

Syntype, RMNH.AVES.87951 (1609), adult female, skin. Loc.: Sentani Lake, [Papua], [Indonesia], 01.iv.1903. Leg.: L.F. de Beaufort and H.A. Lorentz, 08.x.1909.

Syntype, RMNH.AVES.87952 (1609), adult, sex unknown, skin. Loc.: Humboldt Bay, [Papua], [Indonesia], 21.iv.1903. Leg.: L.F. de Beaufort and H.A. Lorentz, 08.x.1909.

Syntype, RMNH.AVES.87953 (1609), adult female, skin. Loc.: Sentani Lake, [Papua], [Indonesia], 19.vi.1903. Leg.: L.F. de Beaufort and H.A. Lorentz, 08.x.1909.

Syntype, RMNH.AVES.87954 (1609), adult male, skin. Loc.: Sentani Lake, [Papua], [Indonesia], 19.vi.1903. Leg.: L.F. de Beaufort and H.A. Lorentz, 08.x.1909.

Syntype, ZMA.AVES.209, adult male, skin. Loc.: Sentani Lake, [Papua], [Indonesia], 08.iv.1903. Leg.: L.F. de Beaufort and H.A. Lorentz.

Syntype, ZMA.AVES.210, adult male, skin. Loc.: Sentani Lake, [Papua], [Indonesia], v.1903. Leg.: L.F. de Beaufort and H.A. Lorentz.

Syntype, RMNH.AVES.207071, adult male, mounted skin. Loc.: Andai, [Papua], [Indonesia], 1.vi.1869. Leg.: C.B.H. von Rosenberg.

Syntype, RMNH.AVES.207072, adult female, mounted skin. Loc.: Andai, [Papua], [Indonesia], 1.vi.1869. Leg.: C.B.H. von Rosenberg.

Although De Beaufort (1909: 403) described this new taxon based on material from the Wichmann Dutch New Guinea Expedition (1903), he also referred to RMNH.AVES.207071 and 207072.

***NasiternapygmaeaGeelvinkiana*** Schlegel, 1871a: 7.

***Nasiternamaforensis*** Salvadori, 1875c: 908.

***Nasiternamisoriensis*** Salvadori, 1875c: 909.

= *Micropsittageelvinkianageelvinkiana* (Schlegel, 1871).

Syntype *geelvinkiana and maforensis*, RMNH.AVES.88014, adult female, skin. Loc.: Numfor Island, [Indonesia], 12.ii.1869. Leg.: C.B.H. von Rosenberg.

Syntype *geelvinkiana and maforensis*, RMNH.AVES.88015, adult male, skin. Loc.: Numfor Island, [Indonesia], 12.ii.1869. Leg.: C.B.H. von Rosenberg.

Syntype *geelvinkiana and maforensis*, RMNH.AVES.88017, adult female, skin. Loc.: Numfor Island, [Indonesia], 14.ii.1869. Leg.: C.B.H. von Rosenberg.

Syntype *geelvinkiana and maforensis*, RMNH.AVES.88018, adult female, skin. Loc.: Numfor Island, [Indonesia], 14.ii.1869. Leg.: C.B.H. von Rosenberg.

Syntype *geelvinkiana and maforensis*, RMNH.AVES.88019, adult male, skin. Loc.: Numfor Island, [Indonesia], 15.ii.1869. Leg.: C.B.H. von Rosenberg.

Syntype *geelvinkiana and maforensis*, RMNH.AVES.88020, adult male, skin. Loc.: Numfor Island, [Indonesia], 15.ii.1869. Leg.: C.B.H. von Rosenberg.

Syntype *geelvinkiana and maforensis*, RMNH.AVES.88021, adult male, skin. Loc.: Numfor Island, [Indonesia], 17.ii.1869. Leg.: C.B.H. von Rosenberg.

Syntype *geelvinkiana and maforensis*, RMNH.AVES.88022, adult male, skin. Loc.: Numfor Island, [Indonesia], 19.ii.1869. Leg.: C.B.H. von Rosenberg.

Syntype *geelvinkiana and maforensis*, RMNH.AVES.88023, adult female, skin. Loc.: Numfor Island, [Indonesia], 19.ii.1869. Leg.: C.B.H. von Rosenberg.

Syntype *geelvinkiana and maforensis*, RMNH.AVES.88024, adult female, skin. Loc.: Numfor Island, [Indonesia], 19.ii.1869. Leg.: C.B.H. von Rosenberg.

Syntype *geelvinkiana and maforensis*, RMNH.AVES.88025, adult female, skin. Loc.: Numfor Island, [Indonesia], 23.ii.1869. Leg.: C.B.H. von Rosenberg.

Syntype *geelvinkiana and maforensis*, RMNH.AVES.88026, adult male, skin. Loc.: Numfor Island, [Indonesia], 26.ii.1869. Leg.: C.B.H. von Rosenberg.

Syntype *geelvinkiana and misoriensis*, RMNH.AVES.207855, adult male, skin. Loc.: Soëk [= Biak], [Indonesia], iii.1869. Leg.: C.B.H. von Rosenberg (= *Micropsittageelvinkianamisoriensis* (Salvadori, 1875).

Syntype *geelvinkiana and misoriensis*, RMNH.AVES.207857, adult female, skin. Loc.: Soëk [= Biak], [Indonesia], iii.1869. Leg.: C.B.H. von Rosenberg (= *Micropsittageelvinkianamisoriensis* (Salvadori, 1875).

Syntype *geelvinkiana and misoriensis*, RMNH.AVES.207859, adult female, skin. Loc.: Soëk [= Biak], [Indonesia], 22.iii.1869. Leg.: C.B.H. von Rosenberg (= *Micropsittageelvinkianamisoriensis* (Salvadori, 1875).

Schlegel (1871a) included specimens from Numfoor and Biak in his description, which was overlooked by Van den Hoek Ostende et al. (1997: 126).

***Nasiternaaruensis*** Salvadori, 1875b: 985.

= *Micropsittakeiensiskeiensis* (Salvadori, 1876).

Syntype, RMNH.AVES.88027, adult male, mounted skin. Loc.: Wokam, Aru Islands, [Indonesia], 30.i.1865. Leg.: C.B.H. von Rosenberg.

Syntype, RMNH.AVES.88028, adult male, mounted skin. Loc.: Wonumbai [= Kobroor], Aru Islands, [Indonesia], 16.v.1865. Leg.: C.B.H. von Rosenberg.

Publication date incorrectly given as 1876 by the IOC.

***Psittacusvenustus*** Temminck, 1821: 121 (nec Kuhl).

= *Neophemachrysostoma* (Kuhl, 1820).

Syntype, RMNH.AVES.88031, adult, sex unknown, mounted skin. Loc.: Tasmania, Australia. Ex: Cabinet Temminck.

Temminck (1821) referred to four specimens of *venustus*: two in his own collection and two in the Linnaean Society in London. Warren (1966: 62) listed a specimen in the NHM (NHMUK 1863.7.6.9) under *Psittacuschrysostomus* Kuhl, 1820, as syntype of *Psittacusvenustus* Temminck, 1821, which she later erroneously listed as holotype under *Psittacusvenustus* Temminck, 1821 (Warren 1966: 304). Because of this ambiguity we do not consider this to be a valid lectotype designation and list RMNH.AVES.88031 as a syntype for *Psittacusvenustus* Temminck, 1821. Temminck sent one specimen to the NMW in 1821 (NMW 1821.LXXIII.43).

***NanodesMusschenbroekii*** “von Rosenberg” Schlegel, 1871b: 34.

= *Neopsittacusmusschenbroekii* (Schlegel, 1871).

Syntype, RMNH.AVES.88032, adult female, skin. Loc.: Hattam, Arfak Mountains, [Papua], [Indonesia], 07.iv.1870. Leg.: C.B.H. von Rosenberg.

Syntype, RMNH.AVES.88033, adult female, skin. Loc.: Hattam, Arfak Mountains, [Papua], [Indonesia], 11.iv.1870. Leg.: C.B.H. von Rosenberg.

Syntype, RMNH.AVES.88034, adult male, skin. Loc.: Hattam, Arfak Mountains, [Papua], [Indonesia], 14.iv.1870. Leg.: C.B.H. von Rosenberg.

***Psittacusvaillanti*** Shaw, 1810: 909

***Psittacusphigy*** Bechstein, 1811: 81.

***Psittacuscoccineus*** Shaw, 1812: 472.

= *Phigyssolitarius* (Suckow, 1800).

Syntype, RMNH.AVES.88055, adult, sex unknown, relaxed mount. Loc.: “New Caledonia” [= Tongatapu], [23.iii–9.iv.1793]. Leg.: [J.-J. de Labilardière]. Ex: Cabinet Temminck.

All authors based their name on Levaillant’s “La Perruche Phigy” (1801b: 125, pl. 64), who based his description on specimens in the MNHN and in the collection of Labillardière. According to Stresemann (1953b: 82) RMNH.AVES.88055 was bought by Temminck in 1805 from the Parisian dealer Bécoeur. This specimen originated from the expedition led by d’Entrecasteaux where Labillardière was the naturalist (1791–1794).

The locality “New Caledonia” is obviously in error, as this parrot is only found in Fiji.

***PsittacusBrownii*** Kuhl, 1820b: 56.

= *Platycercuscaledonicusbrownii* (Kuhl, 1820).

Syntype, RMNH.AVES.88060, adult male, mounted skin. Loc.: Tasmania, Australia. Ex: Cabinet Temminck.

Jansen (2018: 416) suggested that this specimen was collected between 13 January and 16 February 1801, either at Great Taylor Bay or North Bruny, Bruny Island, Tasmania, by C.A. Lesueur or between 20 and 22 May 1802 in Adventure Bay, Bruny Island.

According to Kuhl (1820b), other syntypes were in the MNHN, the Laugier collection, and in London. Warren (1966: 43, 98) listed a syntype in the NHM (NHMUK 1863.7.6.4). Voisin and Voisin (2008a: 494) listed no types for *brownii*, but based on Jansen (2018: 415–416) at least two specimens (MNHN A.C.1388 and 1390; both collected during the Baudin Expedition, 1800–1804) should have been present in the MNHN in 1820.

***Psittacusflavigaster*** Temminck, 1821: 116.

***Psittacusflaviventris*** Temminck, 1821: 117.

= *Platycercuscaledonicus* subsp.

Temminck (1821) used both *flavigaster* (in the caption) and *flaviventris* (in the text) for this taxon. He based his description on specimens in London and on pl. 80 in Levaillant (1805). Salvadori (1891: 546) designated the specimen in the NHM, received from the Linnean Society, as lectotype, a fact overlooked by Warren (1966: 98) who listed this specimen (NHMUK 1863.7.6.4) as syntype. Warren also listed this specimen as syntype for *Psittacus Brownii* Kuhl, 1820. Voisin and Voisin (2008a: 494) listed a specimen in the MNHN (MNHN C.G.2004-68; see Jansen 2018: 415) as the one depicted on pl. 80 by Levaillant, which is thus a paralectotype for both *flaviventris* and *flavigaster*.

***Psittacusicterotis*** “Temminck” Kuhl, 1820b: 54.

= *Platycercusicterotis* Kuhl, 1820.

Kuhl (1820b) based his description on specimens in the RMNH and MNHN. No specimen(s) were found in the RMNH.

***PsittacusBrownii*** Temminck, 1821: 119 (nec Kuhl).

= *Platycercusvenustusvenustus* (Kuhl, 1820).

Temminck (1821) referred to a single specimen collected by Brown in Arnhem’s Land (Northern Territories, Australia) now in the NHM, listed by Warren (1966: 304; NHMUK 1863.7.6.6) as holotype for *Browni* [sic] Temminck.

***Prioniturusmada*** Hartert, 1900: 230.

= *Prioniturusmada* Hartert, 1900.

Paralectotype, RMNH.AVES.90965.

Hartert (1900) did not nominate a type, but described a single specimen in detail. Greenway (1978: 90) considered it the holotype (AMNH 621029). However, Hartert also referred to Schlegel (1874a: 22) and gave all relevant data of a specimen in the RMNH which he recognized as his newly described *P.mada*. According to Art. 72.4.1. (ICZN 1999), this specimen, RMNH.AVES.90965, therefore also belongs to the type series. Greenway’s use of the term “holotype” constitutes a lectotype designation.

A specimen originally in the ZMA collection (ZMA.AVES.56989; collected by Hoedt on Buru in 1870) was not mentioned by Hartert and does not belong to the type series.

***Prioniturusplatenae*** Blasius, 1888: 335.

= *Prioniturusplatenae* Blasius, 1888.

Syntype, RMNH.AVES.88063, adult male, skin. Loc.: Palawan, Philippines, 20.ix.1887. Leg.: C.C. Platen.

***Psittacusplaturus*** “Temminck” Vieillot, 1818b: 314.

= *Prioniturusplaturusplaturus* (Vieillot, 1818).

Vieillot (1818b) based this name on a manuscript name by Temminck. According to Kuhl (1820b: 43), who also attributed this name to Temminck, it is based on a specimen in Temminck’s cabinet from New Caledonia. This must be an error as this species does not occur there. This specimen is not found in the RMNH.

***Psittacussetarius*** Temminck, 1820: livr. 3, pl. 15.

= *Prioniturusplaturusplaturus* (Vieillot, 1818).

Following Dickinson et al. (2022) (see also Introduction of this catalogue under “What is new in this 2023 version”: 14) the specimen illustrated on pl. 15 is the holotype since the text that appeared later is irrelevant regarding type status. According to Temminck (1820), the illustrated specimen was in the RMNH. Temminck had specimens from Indonesia, including Timor (which must be incorrect as *Prioniturus* does not occur there) and the Philippines, including Mindanao. This implies that Temminck had a composite series of at least two, maybe even three or four taxa which he named *setarius*. Pl. 15 could therefore illustrate a composite specimen based on characters of more than one specimen. Temminck also referred to specimens in the MNHN and NHM, but neither Warren (1966) nor Voisin and Voisin (2008a) listed any types.

According to Schlegel (1864c: 45) RMNH.AVES.210650, which is *P.platurusplaturus* (Vieillot, 1818), is the specimen depicted on pl. 15. Although the plate seems to match RMNH.AVES.210650, Schlegel must be wrong as according to handwritten notes under the pedestal it was collected by Müller on Buton on 25 March 1828, eight years after publication of pl. 15. This information might have been added later and prove to be incorrect.

The specimen illustrated on pl. 15 does, however, compares best with *P.p.platurus* from Sulawesi. Sclater (1860b: 223–224) gave Celebes (Sulawesi) as the origin of *setarius* Temminck, but probably based on RMNH.AVES.210650 which Schlegel wrongly considered to be the illustrated specimen and syntype of *setarius*.

***PrioniturusWallacei*** Schlegel, 1864a: 70.

= *Prioniturusplaturusplaturus* (Vieillot, 1818).

Syntype, RMNH.AVES.210650, adult male, mounted skin. Loc.: Buton Island, [Indonesia], 25.iii.1828. Leg.: S. Müller.

Syntype, RMNH.AVES.157200, skull, sex unknown. Loc.: Buton Island, [Indonesia], [22–25.iii.1828]. Leg.: S. Múller.

First published as a nomen nudum by Gray (1859: 18) based on a manuscript name by Gould, Schlegel (1864a) later gave it a formal description. Salvadori (1891: 416) listed two specimens in the NHM as types for *wallacei* Gray, 1859, which Warren (1966: 310) gave no type status as Gray’s publication was a nomen nudum. Warren must have overlooked the valid publication by Schlegel. Schlegel made no direct reference to either Gould or Gray and did not refer to any specimens. However, Schlegel did give Macassar and Buton as type localities. We consider “Macassar” as reference to Gray, making the two specimens in the NHM (NHMUK 1857.11.11.2 and 1857.11.11.18) part of the type series since the RMNH does not have any specimens from Macassar.

Müller visited Buton between 22 and 25 March 1828.

***Psephotuschrysopterygiusblaauwi*** van Oort, 1910a: 71.

= *Psephotellusdissimilis* (Collett, 1898).

Syntype, RMNH.AVES.1577, adult male, skin. Loc.: Port Darwin, Australia. Ex: F.E. Blaauw, 30.viii.1909.

Syntype, RMNH.AVES.2936, adult female, skin. Loc.: Port Darwin, Australia. Ex: F.E. Blaauw, 07.viii.1913.

Syntype, RMNH.AVES.88135 (3708), adult male, skin. Loc.: Port Darwin, Australia. Ex: F.E. Blaauw, 16.xi.1915.

Syntype, RMNH.AVES.88136 (3708), adult female, skin. Loc.: Port Darwin, Australia. Ex: F.E. Blaauw, 16.xi.1915.

Van Oort (1910a) described this race based on one dead (RMNH.AVES.1577) and five living birds in the aviary of F.E. Blaauw. The birds were imported from Port Darwin, Australia, in 1909.

***PsittacellaBrehmii*** Schlegel, 1871b: 35.

= *Psittacellabrehmiibrehmii* Schlegel, 1871.

Holotype, RMNH.AVES.88084, adult female, skin. Loc.: Hattam, Arfak, [Papua], [Indonesia], 11.iv.1870. Leg.: C.B.H. von Rosenberg.

Holotype by monotypy. Schlegel (1871b) wrote having received an adult male from Von Rosenberg. This is an error as the original specimen is a female, which Finsch corrected on the label.

***Psitacella* [sic] *modesta*** Schlegel, 1871b: 36.

= *Psittacellamodestamodesta* Schlegel, 1871.

Holotype, RMNH.AVES.88085, adult male, skin. Loc.: Hattam, Arfak Mountains, [Papua], [Indonesia], 20.iv.1870. Leg.: C.B.H. von Rosenberg.

Holotype by monotypy. Schlegel (1871b) wrote having received an adult male from Von Rosenberg.

***Psittacellalorentzi*** van Oort, 1910e: 212.

= *Psittacellapictalorentzi* van Oort, 1910.

Syntype, RMNH.AVES.88086, adult male, skin. Loc.: Wichmann Mountains, [Papua], [Indonesia], 01.xi.1909. Leg.: H.A. Lorentz, Dutch New Guinea Expedition, 1909.

Syntype, RMNH.AVES.88087, immature male, skin. Loc.: Oranje Mountains, [Papua], [Indonesia], 06.xi.1909. Leg.: H.A. Lorentz, Dutch New Guinea Expedition, 1909.

***Psittaculaalexandridammermani*** Chasen & Kloss, 1932: 8.

= *Psittaculaalexandridammermani* Chasen & Kloss, 1932.

Holotype, RMNH.AVES.14033, adult female, skin. Loc.: Karimondjawa Island, [Indonesia], 15.v.1926. Ex: MZB, 12.i.1950 (MZB 4014).

***Psittaculaalexandrikangeanensis*** Hoogerwerf, 1962: 202.

= *Psittaculaalexandrikangeanensis* Hoogerwerf, 1962.

Hoogerwerf (1962) nominated two specimens in the MZB as types (MZB 23130 and 23134), thereby excluding all other specimens from the type series, including RMNH.AVES.27879, erroneously listed as syntype by Van den Hoek Ostende et al. (1997: 133). Sudaryanti et al. (2006) designated a lectotype (MZB 23130). However, the mere fact that Hoogerwerf did not select a holotype in his description is not a valid reason for a lectotype designation after 1999 (ICZN Recommendation 74G).

***Cyclopsittablythii*** Wallace, 1864: 284.

= *Psittaculirostrisdesmarestiiblythii* (Wallace, 1864).

Possible syntype, RMNH.AVES.90954 (formerly RMNH 88087), adult, sex unknown, skin. Loc.: Misool, [Indonesia], [ii–ix.1860]. Leg.: C. Allen for A.R. Wallace. Ex: G.A. Frank, 1862.

The RMNH received this specimen two years prior to the formal description by Wallace, who referred to six specimens from “Mysol”, making it likely that the specimen in the RMNH was included in the type series.

According to Warren (1966: 37) another syntype is in the NHM (NHMUK 1873.5.12.1543). RMNH.AVES.90954 is a new registration number as the number given in Van den Hoek Ostende et al. (1997: 133; RMNH 88087) was incorrect.

Wallace never visited Misool, where Allen collected for him between February and September 1860 (Baker 2001: 291).

***Cyclopsittadesmarestiiintermedia*** van Oort, 1909b: 229.

= *Psittaculirostrisdesmarestiidesmarestii* (Desmarest, 1826).

Syntype, RMNH.AVES.88088, adult female, skin. Loc.: Sekru, [Papua], [Indonesia], 17.i.1897. Leg.: K. Schädler, 1897.

Syntype, RMNH.AVES.88089, adult male, skin. Loc.: Sekru, [Papua], [Indonesia], 17.i.1897. Leg.: K. Schädler, 1897.

Syntype, RMNH.AVES.88090, adult female, skin. Loc.: Sekru, [Papua], [Indonesia], 26.ii.1897. Leg.: K. Schädler, 1897.

Van Oort (1909b) nominated three specimens as the types, thereby excluding all other specimens from the type series, including RMNH.AVES.89437–89440, erroneously listed as syntype by Van den Hoek Ostende et al. (1997: 133).

***Psittacusiris*** Temminck, 1835: livr. 96, pl. 567.

= *Psitteutelesirisiris* (Temminck, 1835).

Syntype, RMNH.AVES.88091, adult male, relaxed mount. Loc.: [Wienoto], Timor, [Indonesia], [ix.1829]. Leg.: S. Müller.

Syntype, RMNH.AVES.88092, adult male, relaxed mount. Loc.: [Wienoto], Timor, [Indonesia], [ix.1829]. Leg.: S. Müller.

Finsch (1868: 852) referred to one of the RMNH specimens as the type, which constitutes a lectotype designation. He listed the specimens as a male (lectotype) and female; however, both specimens are male. The lectotype designation is therefore not valid, as it is ambiguous.

Müller (1841: 209) described how he encountered this species during his visit to the area of Wienoto and Molo (September 1829).

***Tanygnathusaffinis*** Wallace, 1863b: 20.

***Tanygnathusintermedius*** Schlegel, 1864a: 70.

= *Tanygnathusmegalorynchosaffinis* Wallace, 1863.

Paralectotype for *affinis*, syntype for *intermedius*, RMNH.AVES.88105, adult, sex unknown, mounted skin. Loc.: Buru, [Indonesia], [v.-vi.] 1861. Leg.: A.R. Wallace. Ex: G.A. Frank, 1862.

Syntype for *intermedius*, RMNH.AVES.88106, adult male, mounted skin. Loc.: Buru, [Indonesia], 1863. Leg.: D.S. Hoedt.

Syntype for *intermedius*, RMNH.AVES.88107, adult male, mounted skin. Loc.: Buru, [Indonesia], 1863. Leg.: D.S. Hoedt.

Syntype for *intermedius*, RMNH.AVES.88108, adult female, mounted skin. Loc.: Buru, [Indonesia], 1863. Leg.: D.S. Hoedt.

Syntype for *intermedius*, RMNH.AVES.88109, adult female, mounted skin. Loc.: Buru, [Indonesia], 1863. Leg.: D.S. Hoedt.

Salvadori (1891: 429) designated the lectotype for *affinis* in the NHM (Warren 1966: 5; NHMUK 1873.5.12.1570).

There appears to have been a mix-up of the labels and stands. RMNH.AVES.88106 is a male according to the label, but a female according to the stand. RMNH.AVES.88109 is a female according to the label, but a male according to the stand.

***TanygnathusMorotensis*** Schlegel, 1864a: 70.

= *Tanygnathusmegalorynchosmegalorynchos* (Boddaert, 1783).

Syntype, RMNH.AVES.88100, adult male, mounted skin. Loc.: Morotai, [Indonesia], 27.i.1861. Leg.: H.A. Bernstein.

Syntype, RMNH.AVES.88101, adult female, mounted skin. Loc.: Morotai, [Indonesia], 21.viii.1861. Leg.: H.A. Bernstein.

Syntype, RMNH.AVES.88137, adult male, mounted skin. Loc.: Morotai, [Indonesia], 25.viii.1861. Leg.: H.A. Bernstein.

Syntype, RMNH.AVES.88102, adult female, mounted skin. Loc.: Morotai, [Indonesia], 14.xii.1861. Leg.: H.A. Bernstein.

Syntype, RMNH.AVES.88103, adult female, mounted skin. Loc.: Morotai, [Indonesia], 17.xii.1861. Leg.: H.A. Bernstein.

Syntype, RMNH.AVES.88104, adult female, mounted skin. Loc.: Morotai, [Indonesia], 19.xii.1861. Leg.: H.A. Bernstein.

Schlegel (1864c: 47) and Finsch (1868: 353) only listed one male (RMNH.AVES.88100); they must have both overlooked the second male (RMNH.AVES.88137).

According to the label RMNH.AVES.88101 was collected on 21 August 1861. The stand gives 21 September 1861 as the collecting date.

***PsittacusMulleri*** Müller, 1841: 108.

= *Tanygnathussumatranussumatranus* (Raffles, 1822).

Syntype, RMNH.AVES.88110, adult, sex unknown, mounted skin. Loc.: -. Ex: Cabinet Temminck.

Syntype, RMNH.AVES.88111, adult, sex unknown, skeleton. Loc.: Boeton [= Buton], [Indonesia], [22–25.iii.1828]. Leg.: S. Müller.

Syntype, RMNH.AVES.88112, adult, sex unknown, skeleton. Loc.: Boeton [= Buton], [Indonesia], [22–25.iii.1828]. Leg.: S. Müller.

The locality “Boeton” was written on the stand of RMNH.AVES.88110, but was later erased. Müller (1841) referred to two specimens, RMNH.AVES.88110 and a bird he collected on Buton in 1828. This must be one of the two skeletons. As we are unable to distinguish between them, we list them both as syntypes.

Müller visited Buton between 22 and 25 March 1828.

***Psittacuseuteles*** Temminck, 1835: livr. 96, pl.568.

= *Trichoglossuseuteles* (Temminck, 1835).

Lectotype, RMNH.AVES.88116, adult male, skin. Loc.: [Wienoto], Timor, [Indonesia], [ix.1829]. Leg.: S. Müller.

Paralectotypes, RMNH.AVES.88117 and 88118.

Finsch (1868: 850) referred to the male specimens in the RMNH as the type, which constitutes a lectotype designation.

Müller (1841: 209) described how he encountered this species during his visit to the area of Wienoto and Molo in September 1829.

***Trichoglossusalorensis*** Finsch, 1899a: 226.

= *Trichoglossuseuteles* (Temminck, 1835).

Syntype, RMNH.AVES.88119, adult male, skin. Loc.: Alor, [Indonesia], v.1898. Ex: H. Rolle, 1898.

Syntype, RMNH.AVES.88120, adult male, skin. Loc.: Alor, [Indonesia], v.1898. Ex: H. Rolle, 1898.

***TrichoglossusForsteni*** “Temminck” Bonaparte, 1850a: 3.

= *Trichoglossusforsteniforsteni* Bonaparte, 1850.

Lectotype, RMNH.AVES.88121, adult male, relaxed mount. Loc.: Bima, Sumbawa, [Indonesia], 1842. Leg.: E.A. Forsten.

Paralectotype, RMNH.AVES.88122.

Finsch (1868: 826) referred to the male specimen as the type, which constitutes a lectotype designation.

***TrichoglossusRosenbergii*** Schlegel, 1871a: 9.

= *Trichoglossusrosenbergii* Schlegel, 1871.

Syntype, RMNH.AVES.88123, adult male, skin. Loc.: Soëk [= Biak], Schouten Island, [Indonesia], iii.1869. Leg.: C.B.H. von Rosenberg.

Syntype, RMNH.AVES.88124, adult male, skin. Loc.: Soëk [= Biak], Schouten Island, [Indonesia], iii.1869. Leg.: C.B.H. von Rosenberg.

Syntype, RMNH.AVES.88125, adult male, skin. Loc.: Soëk [= Biak], Schouten Island, [Indonesia], iii.1869. Leg.: C.B.H. von Rosenberg.

Syntype, RMNH.AVES.88126, adult male, skin. Loc.: Soëk [= Biak], Schouten Island, [Indonesia], iii.1869. Leg.: C.B.H. von Rosenberg.

Syntype, RMNH.AVES.88127, adult male, skin. Loc.: Soëk [= Biak], Schouten Island, [Indonesia], iii.1869. Leg.: C.B.H. von Rosenberg.

Syntype, RMNH.AVES.88128, adult male, skin. Loc.: Soëk [= Biak], Schouten Island, [Indonesia], iii.1869. Leg.: C.B.H. von Rosenberg.

Syntype, RMNH.AVES.88129, adult female, skin. Loc.: Soëk [= Biak], Schouten Island, [Indonesia], iii.1869. Leg.: C.B.H. von Rosenberg.

Syntype, RMNH.AVES.88138, adult female, skin. Loc.: Soëk [= Biak], Schouten Island, [Indonesia], iii.1869. Leg.: C.B.H. von Rosenberg.

Syntype, RMNH.AVES.88139, adult female, skin. Loc.: Soëk [= Biak], Schouten Island, [Indonesia], iii.1869. Leg.: C.B.H. von Rosenberg.

***Chalcopsittarubiginosa*** Bonaparte, 1850b: 134.

= *Trichoglossusrubiginosus* (Bonaparte, 1850).

Bonaparte (1850b) referred to a specimen in the Jardin des Plantes (Paris) as the type. The listing of RMNH.AVES.88134 as holotype by Van den Hoek Ostende et al. (1997: 137) is therefore probably an error. Voisin and Voisin (2008a) do not list a type for *rubiginosa*.

***Psitteutelesweberi*** Büttikofer, 1894: 290, taf. XVII, fig. 1.

= *Trichoglossusweberi* (Büttikofer, 1894).

Syntype, RMNH.AVES.88130, adult male, skin and partial skeleton. Loc.: Endeh, Flores, [Indonesia], xii.1888. Leg.: M.W.C. Weber, 1893.

Syntype, RMNH.AVES.88131, adult, sex unknown, skin. Loc.: Reo, North West Flores, [Indonesia], xii.1888. Leg.: M.W.C. Weber, 1893.

Syntype, RMNH.AVES.88132, adult, sex unknown, skin. Loc.: Reo, North West Flores, [Indonesia], xii.1888. Leg.: M.W.C. Weber, 1893.

Syntype, RMNH.AVES.88133, immature, skin. Loc.: Bari, Flores, [Indonesia], xi.1888. Leg.: M.W.C. Weber, 1893.

Syntype, ZMA.AVES.32100, adult female, skin and partial skeleton (RMNH.AVES.255730). Loc.: Endeh, Flores, [Indonesia], 4.i.1889. Leg.: M.W.C. Weber.

The syntype from the former ZMA collection was collected on 4 January 1889. Büttikofer (1894) stated to have specimens from November and December 1888. Since all other data are correct, we assume this to be also a syntype.
